# The monoaminergic system is a bilaterian innovation

**DOI:** 10.1038/s41467-023-39030-2

**Published:** 2023-06-06

**Authors:** Matthew Goulty, Gaelle Botton-Amiot, Ezio Rosato, Simon G. Sprecher, Roberto Feuda

**Affiliations:** 1 Department of Genetics and Genome Biology, University of Leicester, Leicestershire, UK; 2 Department of Biology, Institute of Zoology, University of Fribourg, CH-1700 Fribourg, Switzerland

**Keywords:** Phylogenetics, Evolutionary genetics, Hormones, Synaptic transmission

## Abstract

Monoamines like serotonin, dopamine, and adrenaline/noradrenaline (epinephrine/norepinephrine) act as neuromodulators in the nervous system. They play a role in complex behaviours, cognitive functions such as learning and memory formation, as well as fundamental homeostatic processes such as sleep and feeding. However, the evolutionary origin of the genes required for monoaminergic modulation is uncertain. Using a phylogenomic approach, in this study, we show that most of the genes involved in monoamine production, modulation, and reception originated in the bilaterian stem group. This suggests that the monoaminergic system is a bilaterian novelty and that its evolution may have contributed to the Cambrian diversification.

## Abstract (web summary)

Monoamines act as neuromodulators in the nervous system, but their evolutionary origins are unclear. Here, the authors examine the evolution of genes involved in monoamine production, and processing suggesting that the monoaminergic system evolved in the bilaterian stem-group.

## Introduction

**Fig. 1:**
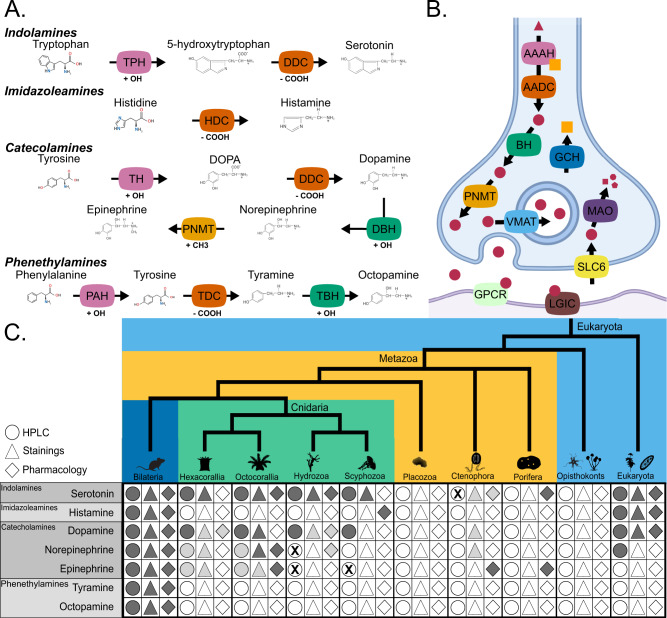
An overview of the monoamine system. **A** Synthesis pathways for key monoamines including the substrate, chemical modification, enzymes, and products. Arrows indicate reactions with the facilitating enzyme overlain. For each enzyme, the label shows the name while the colour indicates the gene family. Chemical modifications are shown next to the enzymes responsible. **B** Cartoon of a synapse with the different enzymes required for the production and detection of monoamines. The red circles represent monoamines and precursor molecules. Yellow squares represent tetrahydrobiopterin co-factor. **C** Current molecular evidence from the literature supporting the presence of monoamines outside Bilaterians in the literature. Dark grey indicates positive results, light grey displays uncertain or partial evidence (e.g., precursors, related compounds) and an X indicates negative results. Blank shapes indicate a lack of evidence. Staining refers to any chemical or immuno-staining experiments; pharmacology refers to evidence-based drug perturbations, adding inhibitors or other chemical interference experiments; HPLC High-Pressure Liquid Chromatography (see Supplementary Data 1 for references and details). PAH phenylalanine hydroxylase, TPH tryptophan hydroxylase, TH tyrosine hydroxylase, DDC dopa decarboxylase, TDC tyrosine decarboxylase, HDC histidine decarboxylase, DBH dopamine beta hydroxylase, TBH tyramine beta hydroxylase, PNMT phenylethanolamine-*N*-methyltransferase, AAAH aromatic amino acid decarboxylase, AADC aromatic amine decarboxylase, BH beta hydroxylase, VMAT vesicular monoamine transporter, GCH GTP cyclo-hydrolase, GPCR g-protein coupled receptor, LGIC ligand gated ion channel, SLC solute ligand carrier, MAO monoamine oxidase. (A) and (B) were made with Biorender. Silhouettes obtained from Phylopic.org. Silhouette images are by Christoph Schomburg (Dendronephthya gigantea); Daniel Jaron (Mus musculus); Emily Jane McTavish, from http://chestofbooks.com/animals/Manual-Of-Zoology/images/I-Order-Ciliata-41.jpg (Ciliophora); Konsta Happonen, from a CC-BY-NC image by sokolkov 2002 on iNaturalist (Geranium sylvaticum); Mali’o Kodis, photograph by Ching (http://www.flickr.com/photos/36302473@N03/) (Chrysaora fuscescens); Noah Schlottman (Pleurobrachia); Oliver Voigt (Trichoplax adhaerens); Steven Traver (Hydra); and Tess Linden (Salpingoeca rosetta).

Monoamines have different biological roles; in the nervous system, they are fundamental neuromodulators^1–5^ required for neuronal plasticity^6,7^. They regulate behaviour and contribute to cognitive functions, including learning and memory formation, emotional states and homeostatic processes such as feeding and sleep^1,3,4,8^. In animals, monoamines are synthesised from aromatic amino acids—primarily tyrosine and tryptophan—using multiple enzymes and co-factors^1^ (see Fig. 1A and Table 1 for definitions). Typically, the first reaction adds a hydroxyl group to the amino acid. Members of the aromatic amino acid hydroxylase (AAAH, Table 1 for definitions) family, phenylalanine hydroxylases (PAHs), tyrosine hydroxylases (THs), and tryptophan hydroxylases (TPHs), mediate this chemical reaction^1,8–11^. Then, enzymes of the amino acid decarboxylase family (AADC), which includes dopa decarboxylases (DDCs), histidine decarboxylases (HDCs), and tyrosine decarboxylases (TDCs), remove a carboxyl group^1,8,12–14^. Some monoamines, such as octopamine, epinephrine and norepinephrine, are modified further by the addition of a second hydroxyl group in a reaction catalysed by Cu(II) monooxygenases, such as dopamine beta-hydroxylases (DBHs), tyramine beta-hydroxylases (TBHs) and monooxygenase DBH-like (MOXDs)^1,13,15,16^. Other monoamines are methylated by phenylethanolamine-*N*-methyltransferases (PNMTs)^1^. Different combinations of these enzymes produce all the major monoamines described in animals (Fig. 1A).

**Table 1:** Definition of key abbreviations used in the paper

Abbreviations	Definition
AAAH	Aromatic Amino Acid Hydroxylase
AADC	Aromatic Amine Decarboxylase
ACM	Acetylcholine Muscarinic Receptor
BUSCO	Benchmarking Universal Single Copy Orthologs
COMT	Catechol-O-Methyltransferase
DAT	Dopamine Transporter
DBH	Dopamine Beta Hydroxylase
DDC	Dopa Decarboxylase
GCH	GTP Cyclo-hydrolase
GPCR	G-protein Coupled Receptor
HDC	Histidine Decarboxylase
HNMT	Histamine-*N*-Methyltransferase
HRH	Histamine Receptor
IDAT	Invertebrate Dopamine Transporter
INE	Drosophila melanogaster transporter Ine
INMT	Indolethylamine-*N*-Methyltransferase
MAO	Monoamine Oxidase
MOXD	Monooxygenase DBH-like
NET	Nor-epinephrine Transporter
NNMT	Nicotinamide-*N*-Methyltransferase
PAH	Phenylalanine Hydroxylase
PNMT	Phenylethanolamine-*N*-Methyltransferase
SAM-MT	S-Adenosyl Methionine Methyltransferase
SERT	Serotonin Transporter
SLC	Solute Ligand Carrier
TBE	Transfer Bootstrap Expectation
TBH	Tyramine Beta Hydroxylase
TDC	Tyrosine Decarboxylase
TH	Tyrosine Hydroxylase
TPH	Tryptophan Hydroxylase
UFB	Ultrafast Bootstrap
VACHT	Vesicular Acetylcholine Transporter
VMAT	Vesicular Monoamine Transporter

However, to function in the nervous system, the production of monoamines is necessary but not sufficient, and additional elements are required. Vesicular monoamine transporters (VMATs) concentrate the monoamines in vesicles before secretion into the synaptic cleft (Fig. 1B)^1,17–19^. Several types of G-protein coupled receptors (GPCRs, e.g., dopaminergic receptors, serotonergic receptors, etc.) detect monoamines on either side of the synapse, triggering signalling cascades^20–25^. Finally, different proteins control the level of monoamines in the synaptic cleft by reuptake (such as transporters of the SLC6 family, like SERTs and DATs)^26–30^ and by degradation (by catabolic enzymes, like monoamine oxidases, MAOs)^31–36^.

Different lines of evidence suggest that monoamines are present in some non-bilaterians animals (Fig. 1C). However, in these animals, the presence of the genes required for the production, modulation, and reception of monoamines remains unclear^20,37–40^. Several studies^11,26,37,39,41–45^ have addressed this problem before, but they were unable to reach a definitive conclusion (summarised in Table 2). A main cause of uncertainty is the limited number of non-bilaterian genomes, such as those of sponges, ctenophores, placozoans, and cnidarians, that have been investigated so far^11,26,37,39,41–45^. Additionally, these studies have focussed on subsets of genes rather than on the monoaminergic system as a whole^11,26,37,39,41–45^. Furthermore, single-gene families have limited phylogenetic signal, making it difficult to resolve their relationships in ‘deep time’^46,47^.

**Table 2:** Summary of previous work and main conclusions

Paper	Genes	Non-Bilaterians	Methods	Conclusions
Iyer et al.^42^	AAAH, AADC, BH, PNMT, COMT, HNMT, MAO	0	Similarity; NJ Tree; ML Tree; Bayesian Tree	DDC and HDC diverge in Metazoa TH sister to TPH and PAH
Caveney et al.^26^	SLC6	0	PCR; Similarity; NJ Tree	3 transporter Clades Predate Bilateria
Anctil^41^	AAAH, AADC, BH, PNMT, MAO, GPCRs, SLC6, VMAT	*Nematostella vectensis* (*Renilla sp*.)	Similarity; Motif; NJ Tree; ML Tree	Homologues of most genes found in *N. vectensis*
Cao et al.^11^	AAAH	2 (*N. vectensis*; *Trichloplax adhaerens)*	Similarity; NJ Tree; ML Tree	TPH and PAH sisters TH and TPH Bilaterian
Siltberg-Liberles et al.^43^	AAAH	0	Bayesian Tree	TPH and TH are sisters Duplications in Metazoa
Kutchko and Siltberg-Liberles^44^	AADC, BH, MAO	0	Similarity; ML Tree	DDC Homologue to TDC and HDC, Metazoan unique TBH homologue of DBH and MOXD PNMT Vertebrate Specific
Krishnan and Schiöth^45^	GPCRs, AAAH, AADC, BH, PNMT	4 (*N. vectensis*; *T. adhaerens*; *Mnemiopsis leidyi*; *Amphimedon queenslandica)*	Similarity	Whole Pathway Present in Cnidaria DDC, DBH and GPCRs in other non-bilaterians
Francis et al.^39^	AADC, BH, MAO	2 Placozoans; 36 Cnidarians; 20 Sponges; 13 Ctenophores	Similarity; ML Tree	DBH Homologues in non-bilaterians DDC Ortholog in Hydrozoa DDC Homologues in non-bilaterians
Moroz et al.^37^	AAAH, AADC, SLC6, BH, PNMT, VMAT, 5HT3R	*T. adhaerens*; 6 Cnidarians; *M. leidyi*; *Pleurobrachia bachei*; *A. queenslandica*	Uncertain	Only DDC and DBH have Orthologs in non-Bilateria

*AAAH* aromatic amino acid hydroxylase, *AADC* aromatic amine decarboxylase, *BH* beta hydroxylase, *PNMT* phenylethanolamine-*N*-methyltransferase, *VMAT* vesicular monoamine transporter, *SLC* solute ligand carrier, *MAO* monoamine oxidase, *COMT* catechol-O-methyltransferase, *HNMT* histamine-*N*-methyltransferase, *GPCR* g-protein coupled receptor, 5HT3R 5-hydroxytryptamine receptor 3, *PAH* phenylalanine hydroxylase, *TPH* tryptophan hydroxylase, *TH* tyrosine hydroxylase, *DDC* dopa decarboxylase, *TDC* tyrosine decarboxylase, *HDC* histidine decarboxylase, *DBH* dopamine beta hydroxylase, *TBH* tyramine beta hydroxylase, *MOXD* monooxygenase DBH-like, *NJ* neighbourhood joining, *ML* maximum likelihood.

Here, we investigate the coordinated evolution of the genes involved in the formation of the monoaminergic system in animals. We study the pattern of duplication of 18 genes that encode the proteins involved in the synthesis, the turnover and the detection of monoamines. We take advantage of modern phylogenomic data covering a wide range of animals, especially non-bilaterians. Moreover, we used methods to minimise the effect of unstable sequences and reconstructed the pattern of gene duplication using recently developed maximum likelihood reconciliation techniques. Our results strongly indicate that many orthologs of the genes involved in the monoaminergic system originated in the bilaterian stem-group. This suggests that the monoaminergic system is a bilaterian innovation and may have contributed to their evolutionary success.

## Results

### The key enzymatic machinery for monoamine synthesis is a bilaterian novelty

Common problems with previous studies were the sparse taxonomical sampling and/or the use of limited phylogenetic methods (see Table 2^11,26,37,39,41–45^). To overcome these issues, we sampled 47 animal species, including 21 non-bilaterians and 18 opisthokonts (Supplementary Data 2 and Methods for further details). To minimise bias associated with poor-quality genomes, we selected species with a high BUSCO completeness score ^48^ (see Supplementary Data 2) while maintaining large taxonomical diversity. After homology and orthogroup identification (see Material and Methods and Supplementary Data 3 and 4), we computed gene trees using maximum likelihood (ML) inference. In addition to ultrafast bootstrap^49^ (UFB), nodal support was estimated using the transferable bootstrap expectation score^50^ (TBE). This method has been designed to identify and account for short and problematic sequences that have limited or conflicting phylogenetic signals^50^. Importantly, the presence of rogue/unstable sequences can affect phylogenetic relationships and the inferred duplication pattern. To identify unstable sequences, we used the Leaf Stability index^51^ (LSI, as implemented in RogueNaRok^52^) and the t-index^50^ (from the TBE analysis). Finally, we used GeneRax^53^ to reconstruct the gene duplication events. In brief, given a gene and species tree, GeneRax uses an ML approach to optimise the duplication and loss events (see^53^ and^54^ for details).

**Fig. 2:**
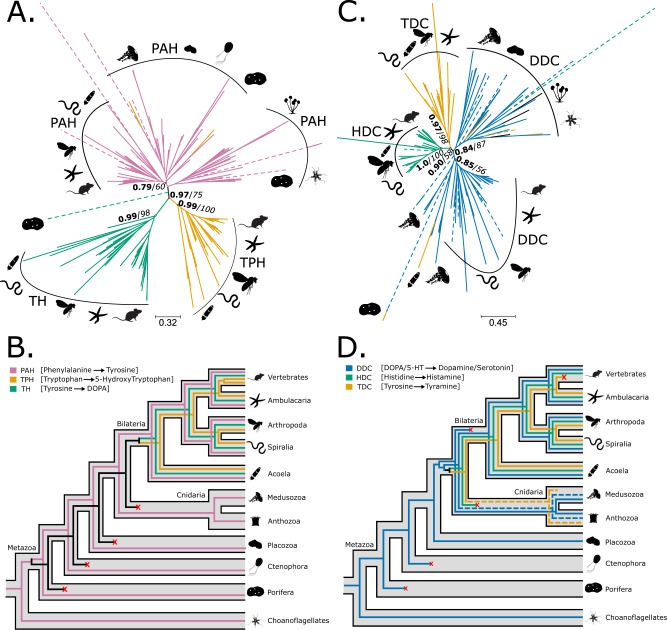
Phylogeny and reconciliation for aromatic amino acid hydroxylases (AAAHs) and amino acid decarboxylases (AADCs). **A** Transfer bootstrap expectation tree and (**B**) simplified illustration of reconciliation calculated using Generax for AAAH sequences. **C** Transfer bootstrap expectation tree and (**D**) simplified illustration of reconciliation calculated using Generax for AADC sequences. The nodal supports shown are transfer bootstrap expectation (TBE) scores (in bold), and ultrafast bootstrap proportion supports (in italic) for key nodes. Dashed lines indicate sequences identified as unstable in the t-index and leaf stability index (LSI) analysis (see Supplementary Data 6 for details). PAH phenylalanine hydroxylase, TPH tryptophan hydroxylase, TH tyrosine hydroxylase, DDC dopa decarboxylase, TDC tyrosine decarboxylase, HDC histidine decarboxylase. Silhouettes obtained from Phylopic.org. Silhouette images are by Andrew R. Gehrke (*Hofstenia miamia*); Daniel Jaron (*Mus musculus*); Mali’o Kodis, photograph by Ching (http://www.flickr.com/photos/36302473@N03/) (*Chrysaora fuscescens*); Mario Quevedo (Asteriidae); Oliver Voigt (Trichoplax adhaerens); Ramiro Morales-Hojas (*Drosophila americana*); Steven Haddock, Jellywatch.org (*Hormiphora californensis*); and Tess Linden (*Salpingoeca rosetta*). Source Data are available at [https://figshare.le.ac.uk/articles/dataset/Monoamine_Neuromodulation_is_a_Bilaterian_Innovation_Results/20391477] in Results/GeneTrees labelled OG_AAAH_TBE.tbe.tree, OG_AAAH_UFB.treefile, OG_AADC_TBE.tbe.tree and OG_AADC_UFB.treefile.

First, we investigated all the sequences encoding the aromatic amino acid hydroxylase (AAAH) family, enzymes that add a hydroxyl group to the aromatic ring of amino acid. This includes three key enzyme types: phenylalanine hydroxylases (PAHs) that synthesise tyrosine from phenylalanine; tyrosine hydroxylases (THs) that mediate the initial step in the synthesis of dopamine, epinephrine, and norepinephrine; and tryptophan hydroxylases (TPHs), which start the synthesis of serotonin (see Fig. 1A). The phylogenetic trees (Fig. 2A, Supplementary 1A–D) recovered the monophyly of TPHs (TBE = 0.99, UFB = 100), THs (TBE = 0.99 and UFB = 98) and ‘TPHs plus THs’ (TBE = 0.97 and UFB = 75). Unlike PAHs, TPHs and THs were unique to Bilateria, apart from a single sequence from the sponge *Amphimedon queenslandica*. However, the results of the LSI and the t-index (see Methods and Supplementary Data 6) suggested that it most likely corresponds to a rogue taxa^55,56^. The absence of TPHs or THs in other sponges supported this conclusion, and we removed the *A. queenslandica* sequence from the dataset. The gene tree to species tree reconciliation corroborated the phylogenetic analyses indicating that THs and TPHs originated in the bilaterian stem group (Fig. 2B, Supplementary Fig. 1F–I).

Amino acid hydroxylases require the co-factor tetrahydrobiopterin to function^1^, which is synthesised by GTP cyclo hydrolases (GCHs), among other enzymes^1^. Unlike AAAH, our phylogenetic tree for GCHs (Supplementary Fig. 3A, B) indicated that these enzymes are found across all animal species and opisthokonts. Phylogeny and reconciliation analyses showed a clear resemblance between gene tree and species tree, suggesting that no major duplication or loss occurred in this gene family (Supplementary Figs. 2, 3D, E). GCHs are not specific to monoaminergic pathways^1^, which may explain their wider distribution.

Next, we investigated the evolution of aromatic amino acid decarboxylase (AADC) enzymes, which remove a carboxyl group as part of monoamine synthesis^1^ (Fig. 1A). Enzymes of the AADC family include dopa decarboxylases (DDCs), histidine decarboxylases (HDCs) and tyrosine decarboxylases (TDCs). The phylogenetic trees (Fig. 2C, Supplementary Fig. 4A–D) recovered the monophyly of HDCs and TDCs (TBE = 0.9 and UFB = 58). Both groups included only bilaterian sequences, except putative TDCs from the cnidarian *Paramuricea biscaya*. In contrast, we identified two clades of DDC encoding genes. The first comprised mainly bilaterians (TBE = 0.86, UFB = 69), while the second included most non-bilaterian and non-metazoan sequences (TBE = 0.84, UFB = 87). In the first DDC clade, the sequences from the sponge *Sycon ciliatum* and the cnidarians *Hydra magnipapillata* and *Clytia hemisphaerica* (TBE = 0.57) were positioned as a sister group to the bilaterian DDCs (TBE = 0.85). LSI and the t-index (see Supplementary Data 6) identified the sequences from *S. ciliatum* as unstable (supported by the alternate positioning in the UFB tree, Supplementary Fig. 4B) but not the sequences from *H. magnipapillata and C. hemisphaerica* (see Supplementary Data 6). This suggests that the latter sequences likely represent putative DDC orthologs, consistent with previous observations^39^. The reconciliation analysis (Fig. 2D, Supplementary Fig. 4F–I) performed after excluding the unstable sequences (see above) suggested that HDCs and TDCs originated in the stem group of Cnidaria and Bilateria. However, such a scenario relies merely on sequences from a single species (the coral *P. biscaya*). For DDCs, the reconciliation suggested a similar pattern: an origin in the stem group of Cnidaria and Bilateria. However, such a hypothesis would require the loss of DDC orthologs in all cnidarians except hydrozoans (Fig. 2D, Supplementary 4F–I). In summary, our results implied that DDCs, HDCs and TDCs are almost exclusive to Bilateria, with the reconciliation analysis placing their origin in the stem lineage of Cnidaria and Bilateria. However, this ‘earlier’ (compared to an origin in the stem lineage of Bilateria) scenario depends upon a few sparsely distributed cnidarian sequences.

**Fig. 3:**
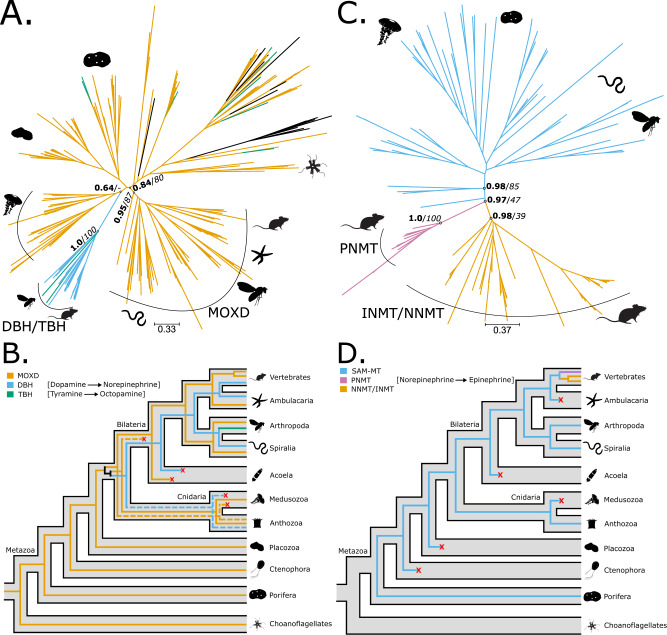
Phylogeny and reconciliation for beta-hydroxylases (BHs) and phenylethanolamine-*N*-methyltransferases (PNMTs). **A** Transfer bootstrap expectation tree and (**B**) simplified illustration of reconciliation calculated using Generax for BH sequences. **C** Transfer bootstrap expectation tree and (**D**) simplified illustration of reconciliation calculated using Generax for PNMT sequences. The nodal supports shown are transfer bootstrap expectation (TBE) scores (in bold), and ultrafast bootstrap proportion supports (in italic) for key nodes. Dashed lines indicate sequences identified as unstable in the t-index and leaf stability index (LSI) analysis (see Supplementary Data 6 for details). DBH dopamine beta hydroxylase, TBH tyramine beta hydroxylase, MOXD monooxygenase DBH-like, INMT indolethylamine-*N*-methyltransferase, NNMT nicotinamide-*N*-methyltransferase, SAM-MT S-adenosyl methionine methyltransferase. Silhouettes obtained from Phylopic.org. Silhouette images are by Andrew R. Gehrke (*Hofstenia miamia*); Daniel Jaron (*Mus musculus*); Mali’o Kodis, photograph by Ching (http://www.flickr.com/photos/36302473@N03/) (*Chrysaora fuscescens*); Mario Quevedo (Asteriidae); Oliver Voigt (*Trichoplax adhaerens*); Ramiro Morales-Hojas (*Drosophila americana*); Steven Haddock, Jellywatch.org (*Hormiphora californensis*); and Tess Linden (Salpingoeca rosetta). Source Data are available at [https://figshare.le.ac.uk/articles/dataset/Monoamine_Neuromodulation_is_a_Bilaterian_Innovation_Results/20391477] in Results/GeneTrees labelled OG_BH_TBE.tbe.tree, OG_BH_UFB.treefile, OG_PNMT_TBE.tbe.tree and OG_PNMT_UFB.treefile.

Dopamine beta hydroxylases (DBHs), tyramine beta hydroxylases (TBHs), and monooxygenase dopamine beta hydroxylase-like (MOXDs) are involved in the synthesis of norepinephrine and octopamine^1^ (Fig. 1A). In vertebrates, DBHs are used to synthesise norepinephrine^1^, while in arthropods TBHs are involved in the production of octopamine^1^. MOXDs are not functionally characterised. The phylogenetic trees (Fig. 3A, Supplementary Fig. 5A, B) supported the monophyly of MOXDs (TBE = 0.95, UFB = 87) and of DBHs and TBHs (TBE = 1.0, UFB = 100), which were almost exclusively limited to bilaterians except for a single sequence from *P. biscaya* in both clades. While the TBE tree placed the non-bilaterian sequences as the sister group of the DBHs/TBHs clade (TBE = 0.64), the UFB tree assigned them as the sister group of the bilaterian MOXDs (UFB = 48) (Supplementary Fig. 5A, B, respectively). To consider these differences, we performed the reconciliation analysis on both trees (Supplementary Fig. 5D–G). When reconciled, both analyses supported the classification of cnidarian sequences as orthologs of DBHs/TBHs (as in the TBE tree, Supplementary Fig. 5A). Furthermore, most reconciliations supported the split between MOXDs and DBHs/TBHs occurring in the ancestor of Cnidaria and Bilateria (Fig. 3B and Supplementary Fig. 5D–F). Interestingly and contrary to previous observations^44^, both trees (Supplementary Fig. 5A, B) indicated that the fruit fly TBH is orthologous (1:1) to human DBH, suggesting that the name distinction is purely semantic (see Supplementary Fig. 5A, B).

Phenylethanolamine-*N*-methyltransferases (PNMTs) catalyse the last step in the production of epinephrine from norepinephrine and belong to the SAM-binding methyltransferase superfamily (SAM-MT)^57^. Our analysis indicated that the enzymes of the SAM-MT family are widely distributed across Metazoa, but PNMTs are uniquely present in vertebrates (TBE = 1, UFB = 100 Fig. 3C, Supplementary 6A, B). Additionally, we showed that these sequences form a monophyletic group with nicotinamide-*N*-methyltransferases (NNMTs) and indolethylamine-*N*-methyltransferases (INMTs), both also unique to vertebrates (Fig. 3C). The reconciliation (Fig. 3D, Supplementary 6D, E) suggested that PNMTs and INMTs/NNMTs are the product of a gene duplication in the ancestor of jawed vertebrates (gnathostomes). A subsequent duplication in mammals gave rise to additional INMT and NNMT paralogs.

### Monoamine transporters orthologs are unique to Bilateria

**Fig. 4:**
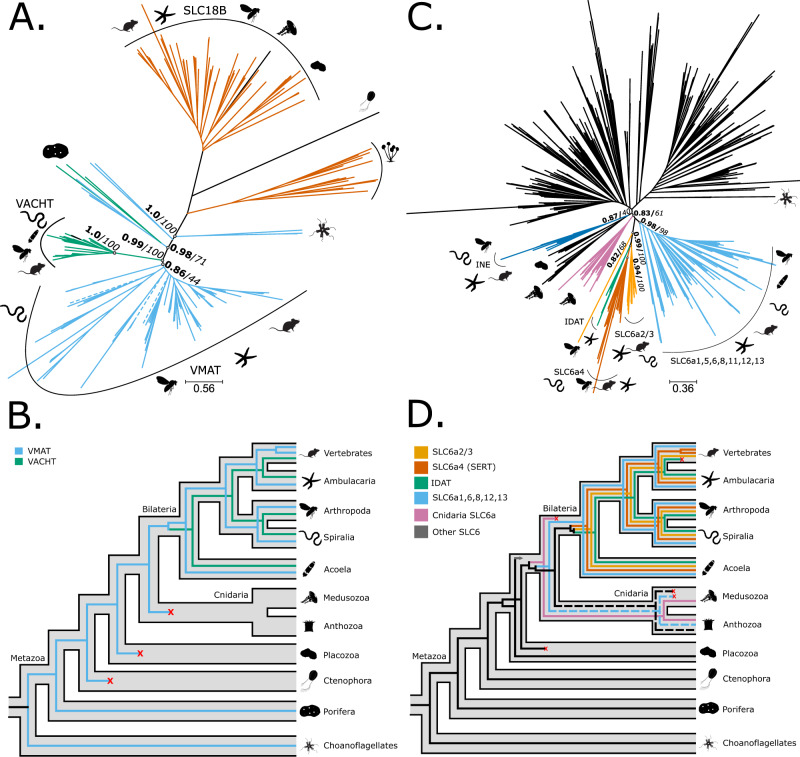
Phylogeny and reconciliation for vesicular monoamine transporters (VMATs) and Solute ligand carrier 6 (SLC6) family members. **A** Transfer bootstrap expectation tree and (**B**) simplified illustration of reconciliation calculated using Generax for VMAT sequences. **C** Transfer bootstrap expectation tree and (**D**) simplified illustration of reconciliation calculated using Generax for SLC6 sequences. The nodal supports shown are transfer bootstrap expectation (TBE) scores (in bold), and ultrafast bootstrap proportion supports (in italic) for key nodes. Dashed lines indicate sequences identified as unstable in the t-index and leaf stability index (LSI) analysis (see Supplementary Data 6 for details). VACHT vesicular acetylcholine transporter, INE *Drosophila melanogaster* transporter Ine, IDAT invertebrate dopamine transporter, SERT serotonin transporter, SLC solute ligand carrier. Silhouettes obtained from Phylopic.org. Silhouette images are by Andrew R. Gehrke (*Hofstenia miamia*); Daniel Jaron (*Mus musculus*); Mali’o Kodis, photograph by Ching (http://www.flickr.com/photos/36302473@N03/) (*Chrysaora fuscescens*); Mario Quevedo (*Asteriidae*); Oliver Voigt (*Trichoplax adhaerens*); Ramiro Morales-Hojas (*Drosophila americana*); Steven Haddock, Jellywatch.org (*Hormiphora californensis*); and Tess Linden (*Salpingoeca rosetta*). Source Data are available at [https://figshare.le.ac.uk/articles/dataset/Monoamine_Neuromodulation_is_a_Bilaterian_Innovation_Results/20391477] in Results/GeneTrees labelled OG_VMAT_TBE.tbe.tree, OG_VMAT_UFB.treefile, OG_SLC6_TBE.tbe.tree and OG_SLC6_UFB.treefile.

Transporters concentrate monoamines inside vesicles for secretion into the synaptic cleft^1^. In Bilateria, vesicular monoamine transporters (VMATs) perform this role. These are part of the solute ligand carrier family 18 (SLC18) and vesicular acetylcholine transporters (VACHTs)^17^. The phylogeny of the SLC18 orthogroup (Fig. 4A, B) supported the monophyly of bilaterian VMATs (TBE = 0.86, UFB = 44) and VACHTs (TBE = 1.0, UFB = 100) (Fig. 4A, Supplementary Fig. 7A, B). Three cnidarian sequences were placed within the VMATs clade; however, LSI and the t-index identified them as unstable (see Supplementary Data 6). Furthermore, our phylogenetic results supported the monophyly of VMATs and VACHTs (TBE = 0.99, UFB = 100, Fig. 4A, Supplementary Fig. 7A, B). Interestingly, the analysis placed some sequences from sponges, choanoflagellates and other holozoans as outgroups of the bilaterian VMAT/VACHT clade (Fig. 4A and Supplementary Fig. 7A, B). After removing the unstable cnidarian sequences, the reconciliation confirmed these findings, namely that VMATs and VACHTs descend from a duplication in the bilaterian stem group (Fig. 4B, Supplementary Fig. 7G, I) and that all non-bilaterians except sponges lack VMAT/VACHT orthologs.

SLC6 is a large family of transporters, including those for serotonin (SERTs/SLC6a4), dopamine in invertebrates (iDATs) and dopamine/epinephrine/norepinephrine in vertebrates (SLC6a2/3s). These proteins regulate the concentration of monoamines in the synaptic cleft^1,26^. All monoamine transporters formed a clade almost exclusive to bilaterians (TBE = 0.99, UFB = 100), apart from a single sequence from the cnidarian *P. biscaya* (Fig. 4C, Supplementary Fig. 8A, B). Our analyses supported the monophyly of SLC6a4 (TBE = 0.94, UFB = 100), iDATs (TBE = 0.98, UFB = 100), and SLC6a2/3s (TBE = 0.96, UFB = 47). Furthermore, we identified a sister group relationship between the transporters for monoamines and a cluster that included transporters for GABA, taurine and creatine (TBE = 0.79, UFB = 50). Such grouping mainly consisted of bilaterian sequences (i.e., SLC6a1s, SLC6a6s and SLC6a8s in Fig. 4C). Both categories of transporters were recovered as a sister group to a large cnidarian-specific clade (TBE = 0.83, UFB = 61). Thus, reconciliation analyses indicated that the transporters for monoamine originated in the stem of Bilateria (Fig. 4D, Supplementary Fig. 8D, E). In addition, based on a single sequence from *P. biscaya* we identified an ortholog to all monoamine transporters in corals. However, evidence from more species is needed to corroborate this finding.

### Evolutionary history of monoamine catabolic enzymes

The correct functioning of neural circuits requires tight control of monoamine levels. Several catabolic enzymes are used in animals for breaking down/inactivating monoamines, which provide a key mechanism of regulation^58^. In bilaterians, this is mediated by catechol-o-methyltransferases (COMTs), histamine-*N*-methyltransferases (HNMTs) and monoamine oxidases (MAOs) (see Fig. 1B).

COMTs add a methyl group and consequently inactivate dopamine, epinephrine, and norepinephrine^32,59^. Phylogenetic trees and reconciliation analyses (Supplementary Figs. 9, 10) identified COMTs as primarily present in deuterostomes. The phylogenetic analyses placed echinoderm COMTs as a sister group to the vertebrate transmembrane-O-methyltransferase clade (TBE = 0.65 and UFB = 60), which also may be able to inactivate dopamine, epinephrine, and norepinephrine^60^. Additionally, this orthogroup included sequences from two corals (*P. biscaya* and *Heliopora coerulea*), two sponges (*S. ciliatum* and *Oscarella pearsei*), a rotifer (*Adineta vaga*) and several non-metazoans (Supplementary Data 3). The reconciliation study inferred many secondary losses across Metazoa (Supplementary Figs. 9, 10D, E). Thus, the phylogenetic distribution of COMTs seem to contrast with the other monoaminergic components that are usually highly conserved across Bilateria (see Supplementary Data 3).

The enzymes of the Histamine-*N*-methyltransferase (HNMTs) family inactivate histamine by adding a methyl group^33,61^. We identified HNMTs in Deuterostomia, Acoela, and Anthozoa (Cnidaria), with sequences present in almost every species from these groups (Supplementary Data 3). Phylogenetic trees and reconciliation analyses (Supplementary Figs. 11, 12A, B) suggested that HNMTs originated in the ancestral group of Cnidaria/Bilateria followed by many secondary losses (Supplementary Figs. 11, 12D, E).

Monoamine oxidases (MAOs) inactivate all monoamines and other amino acid derivatives such as tryptamine, benzylamine and kynuramine^31^. Both the TBE and the UFB trees (Supplementary Figs. 14A, B) supported the monophyly of bilaterian MAOs, including both vertebrate paralogs (TBE = 0.92, UFB = 43). In parallel, we identified a monophyletic clade of cnidarian sequences (TBE = 0.93, UFB = 94) as sister to the bilaterian MAOs. Several choanoflagellate and other non-metazoans contributed to a larger MAO clade that included the metazoan sequences (TBE = 0.99, UFB = 86) (Supplementary Fig. 14A, B). Furthermore, we recognised a MAO-like group that included animal and choanoflagellate sequences related to the large MAO clade (TBE = 0.97, UFB = 91). Interestingly, Medusozoa, Porifera and *D. melanogaster* retain MAO-like sequences only (Supplementary Fig. 13, 14A, B, D, E). We did not identify any MAO in ctenophores or placozoans, while at least one homologue from each clade (MAO and MAO-like) is present in all other animal groups (Supplementary Fig. 13, 14A, B, D, E). The reconciliation study suggested that the two major MAO clades have an ancient origin, significantly predating the origin of animals and neurons (Supplementary Fig. 13, 14D, E).

### Most monoaminergic receptors are specific to bilaterians

Once released in the synaptic cleft, monoamines are detected by specific transmembrane proteins expressed on both sides of the synapse. These receptors are the effectors that trigger signalling cascades inducing a physiological response. Most monoamine receptors are G-protein-coupled receptors (GPCRs) of class A^20,45^. However, serotonin can also be detected by ligand-gated ion channel receptors from the cys-loop repeated gene family^1^ (5HT3Rs).

Our studies revealed that all putative 5HT3R sequences form an orthogroup with the ligand-gated zinc-activated ion channels. This group comprised vertebrates and some urochordates and acoels sequences (see Supplementary Data 3). The phylogenetic tree indicated that most vertebrate 5HT3Rs are monophyletic (TBE = 0.96, UFB = 97; Supplementary Fig. 16A, B). Some fish 5HT3R-annotated sequences clustered with zinc-activated ion channels. Accordingly, the reconciliation study suggested that while several losses occurred in bilaterians, 5HT3Rs underwent an expansion in vertebrate lineages (Supplementary Fig. 15, and 16D, E).

Then, we turned our attention to GPCRs. Our analysis divided the monoaminergic GPCRs into two orthogroups (see Supplementary Data 3). The first, hereafter OG30, contained histamine receptors 1, 3 and 4 (HRH1, HRH3, HRH4) and acetylcholine muscarinic receptors (ACMs). It included bilaterian and non-bilaterian sequences. The second, hereafter OG1, comprised the remaining known monoaminergic GPCRs, including the receptors for serotonin, dopamine, epinephrine/norepinephrine, octopamine/tyramine, trace amines and histamine receptor 2 (HRH2). OG1 included sequences from bilaterians and non-bilaterians (Supplementary Data 3). However, given the taxonomic composition and the lack of outgroups, it was impossible to understand the duplication pattern and the following orthology relationships.

To overcome this limitation, we expanded our study to include all GPCR orthogroups. To reduce the computational burden, we considered non-redundant, seven transmembrane domains GPCRs only (see methods). Thus, we selected 2,837 GPCRs and used CLANs^62^ (a similarity-based method) to identify clusters of related sequences. In brief, CLANs use an all-*vs*.-all BLAST to compute similarity and to cluster sequences at different similarity thresholds^62^ (i.e. different P-values). Applying a threshold including all connections with P-value < 1e^−60^, we observed that most bilaterian sequences from OG1 and OG30 clustered together (Supplementary Fig. 19A). To identify potential outgroups, we relaxed the threshold to include all connections with *P*-value < 1e^−40^ (Supplementary Fig. 19C). At this threshold, we observed that OG1, OG30, adenosine receptors, and melatonin receptors formed a cluster with many interconnected sequences and that such a cluster had sporadic connections with opsins and other GPCRs. To better focus on monoaminergic GPCRs, we increased the P-value threshold to 1e^-42^ (Supplementary Fig. 19B) and identified 1,277 sequences connected to OG1 and OG30.

**Fig. 5:**
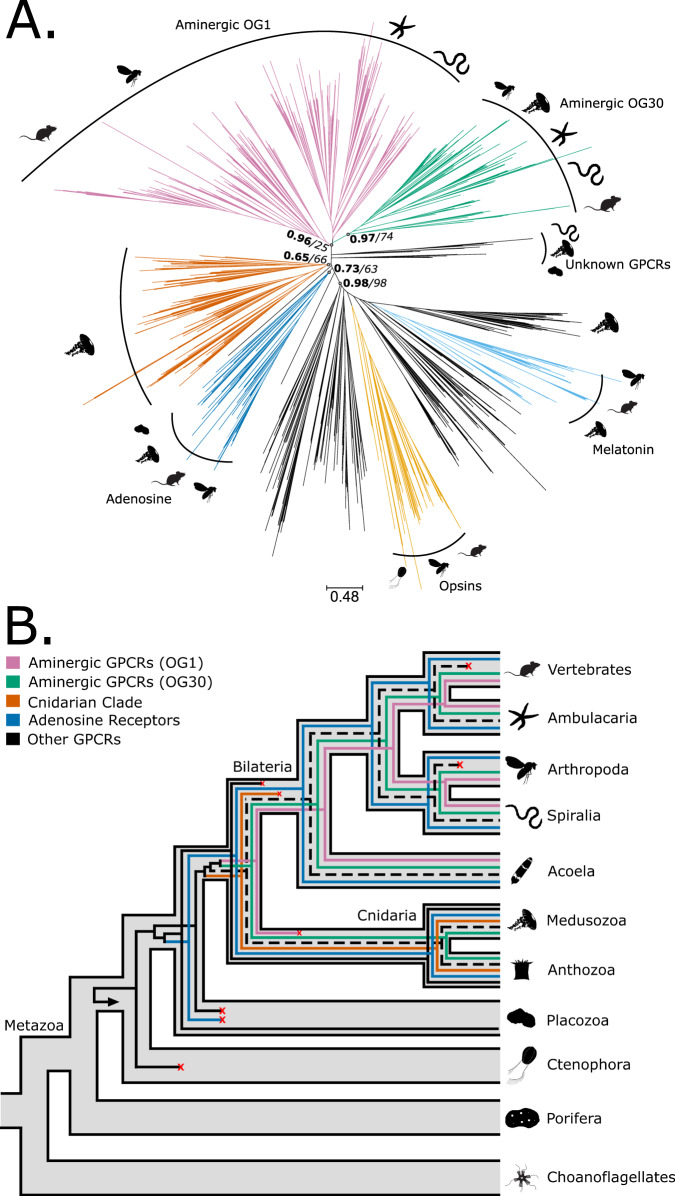
Phylogeny and reconciliation of the monoaminergic GPCRs. **A** Transfer bootstrap expectation tree and (**B**) simplified illustration of reconciliation calculated using Generax for monoaminergic g-protein coupled receptors (GPCRs). The nodal supports shown are transfer bootstrap expectation (TBE) scores (in bold), and ultrafast bootstrap proportion supports (in italic) for key nodes. Dashed lines indicate sequences identified as unstable in the t-index and leaf stability index (LSI) analysis (see Supplementary Data 6 for details). OG orthogroup. Silhouettes obtained from Phylopic.org. Silhouette images are by Andrew R. Gehrke (*Hofstenia miamia*); Daniel Jaron (*Mus musculus*); Mali’o Kodis, photograph by Ching (http://www.flickr.com/photos/36302473@N03/) (*Chrysaora fuscescens*); Mario Quevedo (Asteriidae); Oliver Voigt (Trichoplax adhaerens); Ramiro Morales-Hojas (Drosophila americana); Steven Haddock, Jellywatch.org (*Hormiphora californensis*); and Tess Linden (*Salpingoeca rosetta*). Source Data are available at [https://figshare.le.ac.uk/articles/dataset/Monoamine_Neuromodulation_is_a_Bilaterian_Innovation_Results/20391477] in Results/GPCRAnalysis/GeneTrees labelled GPCR_CLANs_TBE.tbe.tree and GPCR_CLANs_UFB.treefile.

While CLANs is a powerful tool to investigate the relationships between a large number of sequences, it cannot clarify patterns of gene duplication and specific evolutionary histories. Thus, we performed phylogenetic studies using opsin sequences as the outgroup. Both the UFB and the TBE trees (Fig. 5A, Supplementary Fig. 20 A, B) provided support for the monophyly of OG30 (TBE = 0.97 and UFB = 74) and of OG1+OG30 (TBE = 0.96 and UFB = 25). Mainly, these clades are composed by bilaterian sequences, except for a few from cnidarians that are a sister group to the ACM receptors within OG30. Additionally, both trees supported the existence of a large cnidarian-specific monophyletic group (TBE = 0.65, UFB = 66). Additionally, we identified a clade of adenosine receptors (TBE = 0.73, UFB = 63), which included sequences from bilaterians, cnidarians and placozoans. The adenosine receptors clade was placed as sister to OG1 and OG30 and the cnidarian-specific clade. The main disagreement between the UFB and the TBE trees was the position of OG1. The UFB tree recovered OG1 as monophyletic, albeit with low support (UFB = 37). The TBE tree divided OG1 in two clades with OG30 nested within (TBE = 0.70) (Fig. 5A, Supplementary Fig. 20 A, B). Another difference between the trees was the phylogenetic position of two placozoan sequences. These were placed within OG1 by UFB but as a sister group to OG1 and OG30 by TBE. However, the LSI analysis identified these sequences as unstable (Supplementary Data 6).

The reconciliation performed using the TBE and UFB trees supported that OG1, OG30 and the cnidarian-specific clade originated from a duplication that gave rise to the adenosine receptors and to a “proto-monoamine” receptor in the ancestor of placozoans, cnidarians and bilaterian. The “proto-monoamine” receptor subsequently gave rise, in the cnidarian/bilaterian stem group, to the canonical monoamine receptors (OG30 and OG1) and to the cnidarian-specific clade (Fig. 5B, Supplementary Fig. 20C, D–F). Both trees supported OG1 and OG30, originating from a single gene in the cnidarian/bilaterian stem group. Subsequently, OG1 underwent a significant expansion in the bilaterian stem group giving rise to the modern bilaterian monoaminergic receptors. However, the two trees disagree on the precise number of copies of OG1 in the common ancestor of bilaterians, with UFB indicating a single copy (Supplementary Fig. 20D, F) and TBE suggesting multiple copies (Supplementary Fig. 20C, E).

In summary, our results suggest that monoaminergic GPCRs originated from a gene duplication event that also gave rise to the adenosine receptors in the eumetazoan stem-group. Modern monoaminergic receptors can be divided into two subclades. The first is bilaterian specific and contains receptors for serotonin, dopamine, epinephrine/norepinephrine, octopamine/tyramine, trace amines and HRH2. The second includes HRH1, HRH3, HRH4 and ACM receptors comprising both bilaterian and cnidarian sequences. Our reconciliation analyses indicate that the two subclades diverged in the Bilateria/Cnidaria stem group.

## Discussion

**Fig. 6:**
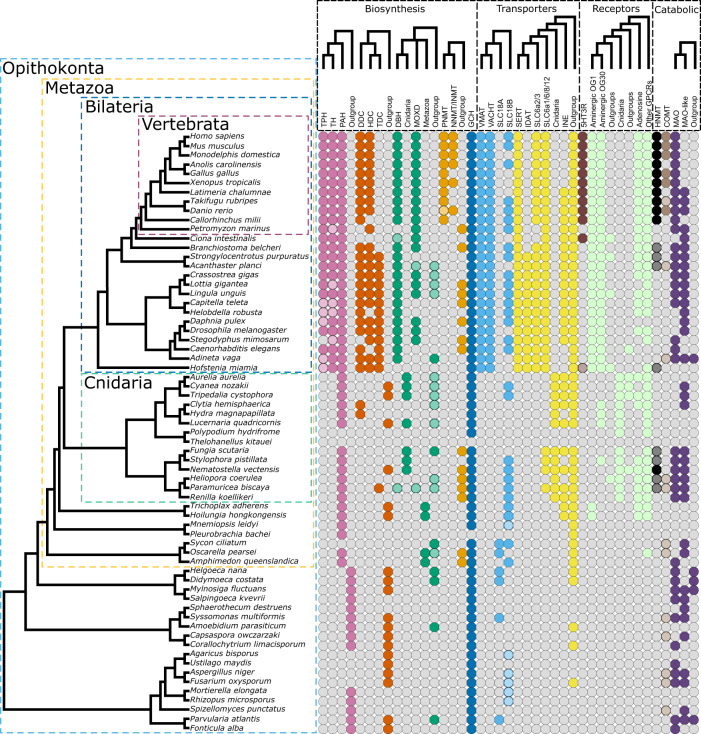
Distribution of monoaminergic system genes. Presence/absence of monoamine pathway genes inferred using the reconciliation analysis. Colours correspond to orthogroups, with darker shades indicating matches to specific InterProScan profiles (Supplementary Data 4). OG orthogroup, PAH phenylalanine hydroxylase, TPH tryptophan hydroxylase, TH tyrosine hydroxylase, DDC dopa decarboxylase, TDC tyrosine decarboxylase, HDC histidine decarboxylase, DBH dopamine beta hydroxylase, TBH tyramine beta hydroxylase, MOXD monooxygenase DBH-like, PNMT phenylethanolamine-*N*-methyltransferase, INMT indolethylamine-*N*-methyltransferase, NNMT nicotinamide-*N*-methyltransferase, VMAT vesicular monoamine transporter, VACHT vesicular acetylcholine transporter, GCH GTP cyclo-hydrolase, SLC solute ligand carrier, MAO monoamine oxidase, HNMT histamine-*N*-methyltransferase, SERT serotonin transporter, IDAT invertebrate dopamine transporter, 5HT3R 5-hydroxytryptamine receptor 3, INE *Drosophila melanogaster* transporter Ine, COMT catechol-O-methyltransferase, GPCR g-protein coupled receptor. Source Data are available at [https://figshare.le.ac.uk/articles/dataset/Monoamine_Neuromodulation_is_a_Bilaterian_Innovation_Results/20391477] in Results/Broccoli labelled table_OGs_protein_names.txt and in Results/InterProScan labelled *_IPS.tsv.

In this study, we have provided strong phylogenetic evidence that the genes that are essential for building a monoaminergic system (i.e., encoding the proteins required for the production, detection, and degradation of monoamines in the nervous system) appear as a whole in bilaterians. Our results (summarised in Fig. 6) allow for a substantial clarification of the evolution of the monoaminergic system.

Compared to previous work^11,26,37,39,41–45^ we have included a larger taxonomic sample, which has provided greater resolution in clarifying the evolution of the different components of the monoaminergic system. We have used statistical methods to pinpoint unstable taxa and to reconcile the gene trees with the species tree, which has improved the robustness of our phylogenetic inferences. We have confirmed previous observations, for instance, that THs and TPHs are present only in Bilateria^11,37,43^. However, in contrast to previous work, we show that most monoaminergic genes evolved through gene duplication in the Bilateria stem group and that others, such as 5HT3Rs and PNMTs, originated in vertebrates. The distribution of orthologs summarised in Fig. 6, suggests a scenario where the monoaminergic system was assembled by combining ancient enzymes with broad functions (such as GCH and MAO) with newly evolved bilaterian-specific genes (such as TPH and TH) into a new functional unit.

The conclusion that the monoaminergic system is a bilaterian innovation may seem in conflict with existing experimental observations reporting the presence of monoamines in non-bilaterian metazoans^38^ (Fig. 1C). How could non-bilaterians produce monoamines? One possible solution is through the functional flexibility of synthetic enzymes. Recently, studies have identified that PAH orthologs produce serotonin in *Drosophila melanogaster, Caenorhabditis elegans*, and mice^9,10,63,64^. Additionally, mutational analyses of mammalian PAH have shown that a few simple mutations can change their substrate specificity from phenylalanine to tryptophan, the key amino acid required for serotonin production^65,66^. Furthermore, alternative pathways could be responsible for the production of monoamines. For example, it has been proposed that the production of L-DOPA (a precursor of dopamine—see Fig. 1A) could be mediated by a tyrosinase in *Hydra*^67^ and in the sea anemone *Metridium senile*^68,69^. There is evidence of a similar pathway functioning in young mice^70,71^. Similarly, *Caenorhabditis elegans* TH-mutants maintain a relatively high dopamine level, suggesting that alternative synthesis pathways exist^5^. Outside animals, other pathways are known, such as cytochrome T5H and other monooxygenases that can produce serotonin and dopamine in plants^72–75^.

Although production is a necessary premise, it is not sufficient to imply function, which depends on the nature (and location) of the receptors involved. Our phylogenetic analysis suggests that the receptors for serotonin, dopamine, epinephrine/norepinephrine, octopamine/tyramine, trace amines and the HRH2s are uniquely present in Bilateria. Thus, they originated from a single gene duplication event in the cnidarian/bilaterian stem group. While the orthologs of histamine receptors 1, 3 and 4 are limited to Bilateria, they appear to be secondarily absent in Cnidaria, with just the ACM receptors conserved across both Cnidaria and Bilateria. We also identified an independent expansion of GPCRs that are unique to Cnidaria but closely related to the canonical monoaminergic receptors. The functional role of these receptors is yet to be determined, but we speculate that they might drive the response to monoamines observed in cnidarians^38^. Despite the considerable progress presented here, we are aware that more remains to be done to unravel the function of monoamine-related enzymes and GPCRs in non-bilaterian animals. Increasing the number of high-quality genomes (e.g., chromosomal level assembly) for many more organisms should enable more precise characterisation of orthology relationships.

**Fig. 7:**
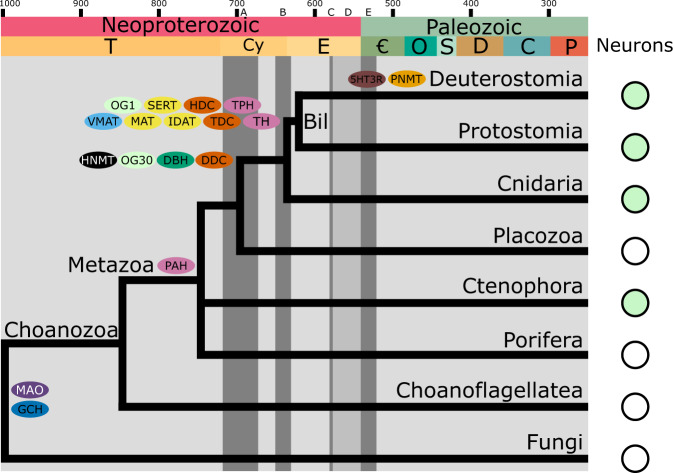
Synopsis of the evolution of the monoaminergic system. A simplified time-calibrated species tree illustrating the origin of the key monoaminergic genes and the presence of neurons. A Sturtian glaciation, B Marinoan glaciation, C Gaskiers glaciation, D Occurrence of Ediacaran Biota/early animal fossils, E the Cambrian explosion. Species tree dated following Dos reis et al.^105^, Geological column is shown in accordance with the ICS International Chronostratigraphic Chart (updated 2017)^106^. Bil Bilateria, OG orthogroup, PAH phenylalanine hydroxylase, TPH tryptophan hydroxylase, TH tyrosine hydroxylase, DDC dopa decarboxylase, TDC tyrosine decarboxylase, HDC histidine decarboxylase, DBH dopamine beta hydroxylase, PNMT phenylethanolamine-*N*-methyltransferase, VMAT vesicular monoamine transporter, GCH GTP cyclo-hydrolase, MAO monoamine oxidase, HNMT histamine-*N*-methyltransferase, SERT serotonin transporter, MAT monoamine transporter, IDAT invertebrate dopamine transporter, 5HT3R 5-hydroxytryptamine receptor 3.

In summary, our data indicate that the key genes required for monoamine production, modulation, and reception emerged in the stem-bilaterians. This discovery suggests that the monoaminergic system evolved to the Cryogenian/Ediacaran boundary, about 650−600 Mya (Fig. 7), pre-dating the Cambrian (~540 Mya). In the transition between Ediacaran and Cambrian, the oceans experienced a progressive, likely non-uniform, increase in oxygen concentration^76–80^. The accepted view is that the increased oxidative capacity of the environment favoured the emergence of more energetically intensive and complex modes of life^71^. Compared to previous epochs, the fossil record shows a shift from filter-feeding and low-motility forms to more elaborated body plans compatible with locomotion and predatory behaviour^81–83^. The origin of the monoaminergic system in the Cryogenian/Ediacaran (Fig. 7), the established roles of monoamines in bilaterians^4–7^, and the morphological changes evident in the fossil record^81–83^, lead us to speculate that it could have played a role in the Cambrian diversification for example by providing flexibility of the neural circuits to facilitate the interaction with the environment.

## Methods

This study utilised wholly computational methods using data sourced from publicly accessible databases, as such ethics approval was not necessary for the methods used.

### Species selection

Whole proteomes were downloaded from NCBI and ENSMBL (see Supplementary Data 2). Additionally, to improve our diversity for Cnidaria, we assembled transcriptomic data for seven species (see Supplementary Data 2) using Trinity^84^ (with–trimmomatic argument)and used Transdecoder^85^ (LongOrfs tool with default options) to infer the amino acid sequences. We used the BUSCO^48^ eukaryota_odb10 database (default options) of 255 single-copy genes to measure the completeness of the proteomes. We selected 101 species (including 47 animals and 54 other eukaryotes) considering both taxonomical diversity and BUSCO completeness score (see Supplementary Data 2).

### Homologues and orthogroup identification

The KEGG pathways^86^ for serotonergic and dopaminergic synapses as well as published literature, were used to identify monoamine pathway genes (See Supplementary Data 4). To identify homologous sequences for each gene of interest, we used BLASTP^87^ (default options with -evalue 1e-10, -outfmt 6) with known sequences from SwissProt^88^, EggNOG^89^ or KEGG^86^ as seeds (detailed in Supplementary Data 4). All BLAST hits with an e-value < 1e^-25^ were extracted and annotated using BLASTP against the SwissProt database. BLAST hits for each species were filtered to remove redundant sequences using CD-HIT (-c 1.0 with default options)^90^.

Starting from the putative homologues to identify the orthogroups, we used Broccoli using default parameters and the maximum likelihood tree reconstruction method^91^. Orthogroups were then annotated based on sequences from *Homo sapiens*, *Mus musculus* and *Drosophila melanogaster* (Supplementary Data 3). Furthermore, orthogroups of interest were analysed using InterProScan^92^ (-f TSV–goterms–pathways options used) and manually inspected for matches to profiles associated with known monoamine genes (details in Supplementary Data 4).

Initially, we performed the BLAST searches and Broccoli orthology inference using all 101 species and constructed phylogenetic trees on orthogroups of interest. However, phylogenetic analysis (see Supplementary Figs. 1E 3C, 4E, 5C, 6C, 7E, 8C, 10C, 12C, 14C, 16C, 17C and 18C) indicates that the vast majority of monoaminergic genes are metazoans-specific (Supplementary Data 3). To reduce the computational burden, we focused on 47 animal and 18 opisthokont species.

### GPCR analysis

We assembled a dataset of monoamine receptors by combing the different Broccoli orthogroups with GPCR annotations (see main text for justification). Phobius^93^ (-s output) was used to estimate the number of transmembrane domains (TMDs) and sequences with 7TMDs were kept, and CD-hit^90^ was used to eliminate homologues with >80% similarity (-c 0.8 with default options). We used CLANs^62^ (all-vs-all BLAST calculated using the online tool: https://toolkit.tuebingen.mpg.de/tools/clans) to cluster sequences based on their similarity across different stringency thresholds/p-values (from 1e^−100^ to 1e^−30^). The clusters were annotated using *Homo sapiens*, *Mus musculus* and *Drosophila melanogaster* sequences. All sequences connecting to the cluster which contained the known aminergic GPCRs at p-value=1e^−42^ were extracted, and opsin sequences were used as the outgroup.

### Phylogenetic analyses

#### Gene trees

Each gene family of interest was aligned using MAFFT^94^ using (–auto option and 1,000 max iterations), and we removed sites with >70% gaps using trimAl^95^ (-gt 0.3 with default options, see Supplementary Data 5 for alignments statistics calculated using PhyKIT^96^. IQ-TREE2^97^ was used to reconstruct the gene trees under the best-fitting model selected with BIC (arguments used: -m MFP -mset Blosum62,cpREV,Dayhoff,DCMut,FLU,HIVb,HIVw,JTT,JTTDCMut,LG,mtART,mtMAM,mtREV,mtZOA,mtMet,mtVer,mtInv,Poisson,PMB,rtREV,VT,WAG,GTR20 -mrate E,I,G,I+G,R -B 1000–wbtl–bnni–alrt 1000–abayes,–alrt 1,000 and–abayes not used in GPCR analyses, Supplementary Data 4). Node support was calculated using 1,000 Ultrafast Bootstrap^49^ (UFB) repeats. We also estimated nodal support using transferable bootstrap expectation (TBE)^50^ scores from 100 non-parametric bootstrap repeats (-b 100 –tbe, see main text for justification).

#### Reconciliation analyses

Generax^53^ was used to reconcile the gene tree with the species tree using the undatedDL model (-r UndatedDL and –unrooted-gene-tree arguments) using the closest proxies to the best-fit models, selected by IQ-TREE2. Specific rate parameters were estimated for each orthogroup (–family-specific-rates option). Reconciliations were visualised using recphyloXML^98^.

To account for the uncertainty in non-bilaterian animal relationships, we performed the reconciliation using the ctenophore-first^99,100^ and sponge-first hypothesis^101–103^. Similarly, we performed a reconciliation including the non-opisthokont species. However, given the lack (or instability - see above) of monoaminergic genes in ctenophores and sponges and in general outside metazoans (see Supplementary Data 3), the differences between the scenarios were marginal (Supplementary Material).

### Rogue taxa analysis

To evaluate the presence of problematic sequences^46,52,55,56^, we used two orthogonal methods the t-index^50^ and the Leaf stability index^51,104^. The t-index evaluates how often the taxa change position across the bootstrap repeats (i.e., how unstable the sequence is). The Leaf stability index (LSI) uses quartet frequencies to estimate sequence stability, and it was calculated using RogueNaRok^52^ (rnr-lsi default options). The LSI was independently estimated using the TBE bootstrap trees and the UFB trees. To identify unstable taxa, we mainly used the t-index and considered sequences with a score > 2 as unstable. We further validated this instability with LSI where, for each sequence, the lower the value, the more unstable it is. Where unstable sequences may have an influence on the topology or reconciliation, we removed them from the alignment and re-ran phylogenetic analyses as described above.

### Reporting summary

Further information on research design is available in the Nature Portfolio Reporting Summary linked to this article.

## Supplementary information

Supplementary information

Description of Additional Supplementary Files

Supplementary Data 1- 6

Reporting Summary

## Data Availability

All data and results underlying this study have been deposited in the figshare repository under accession codes: 20391462; 20391477. Source data trees for Figs. 2A, 2C, 3A, 3C, 4A and 4C can be found in Figshare repository 20391477 in Results/GeneTrees and are labelled *_TBE.tbe.tree and *_UFB.treefile. Source data tree for Fig. 5A can be found in Figshare repository 20391477 in Results/GPCRAnalysis/GeneTrees labelled GPCR_CLANs_TBE.tbe.tree and GPCR_CLANs_UFB.treefile. Source data for Fig. 6 can be found in Figshare repository 20391477 in Results/Broccoli labelled table_OGs_protein_counts.txt and in Results/InterProScan labelled *_IPS.tsv. Source data trees for Supplementary Figures can be found in Figshare repository 20391477 in Results/GeneTrees, Results/GPCRAnalysis/GeneTrees, Results/Reconciliations and Results/RogueTaxaAnalysis/GeneTreesReanalysis. Publicly available data/databases used in this study can be accessed at: NCBI (https://www.ncbi.nlm.nih.gov/); ENSMBL (https://ensemblgenomes.org/); EuckProt (https://evocellbio.com/eukprot/); KEGG pathways (https://www.kegg.jp/kegg/pathway.html); EggNOG (http://eggnog5.embl.de/#/app/home); SwissProt (https://www.uniprot.org/). Accession codes/sources for publicly available original sequence data used in this study are outlined in Supplementary Data 2.

## Code availability

Computation used in this study used published software, references are provided, and necessary options/parameters are outlined in the Methods section.
