# Supplementary material for: The monoaminergic system is a bilaterian innovation
Source: Nat Commun. 2023 Jun 6;14:3284. doi: 10.1038/s41467-023-39030-2 (PMC10244343; doi:10.1038/s41467-023-39030-2)

# Supplementary Information

## The monoaminergic system is a bilaterian innovation

Matthew Goulty[1], Gaelle Botton-Amiot[2], Ezio Rosato[1], Simon Sprecher[2] and Roberto Feuda[1]*

1 Department of Genetics and Genome Biology, University of Leicester, Leicestershire, UK

2 Department of Biology, Institute of Zoology, University of Fribourg, CH-1700 Fribourg, Switzerland

* Corresponding Author

Roberto Feuda: rf190@le.ac.uk

**Supplementary Figures**

Supplementary Figure 1: Analysis of AAAHs. A) TBE tree with TBE score supports. B) Maximum likelihood tree with ultrafast bootstrap proportion supports. C) TBE tree with TBE score supports without rogue sequences. D) Maximum likelihood tree with ultrafast bootstrap proportion supports without rogue sequences. E) Maximum likelihood tree including all eukaryotic sequences with ultrafast bootstrap proportion supports. F) Reconciliation using sponge-first hypothesis. G) Reconciliation without rogue sequences using sponge first hypothesis. H) Reconciliation using ctenophore-first hypothesis. I) Reconciliation without rogue sequences using ctenophore-first hypothesis. J) Reconciliation of the UFB tree including all eukaryotic sequences. Nodes in reconciled trees indicated as speciation events (S) or duplication events (D). All trees shown as rooted, non-reconciled trees rooted arbitrarily for visualisation. AAAH = aromatic amino acid hydroxylase, TBE = transfer bootstrap expectation, UFB = ultrafast bootstrap. Branches colour coded by sequence annotation; orange = tryptophan hydroxylase; green = tyrosine hydroxylase; purple = phenylalanine hydroxylase; and black = others and non-opithokont sequences. Source Data are available at [https://figshare.le.ac.uk/articles/dataset/Monoamine_Neuromodulation_is_a_Bilaterian_Innovation_Results/20391477]; in Results/GeneTrees labelled OG_AAAH_TBE.tbe.tree and OG_AAAH_UFB.treefile; in Results/RogueTaxaAnalysis/GeneTreeReanalysis labelled OG_AAAH_Rogue_TBE.tbe.tree and OG_AAAH_Rogue_UFB.treefile; in Results/GeneTrees/Eukaryotes_GeneTrees labelled OG_AAAH_EUKIQ.treefile; in Results/Reconciliation/SpongeFirst labelled OG_AAAH and OG_AAAH_Rogue; in Results/Reconciliation/CtenophoreFirst labelled OG_AAAH and OG_AAAH_Rogue; and in Results/Reconciliation/Eukaryote labelled OG_AAAH.

1A.

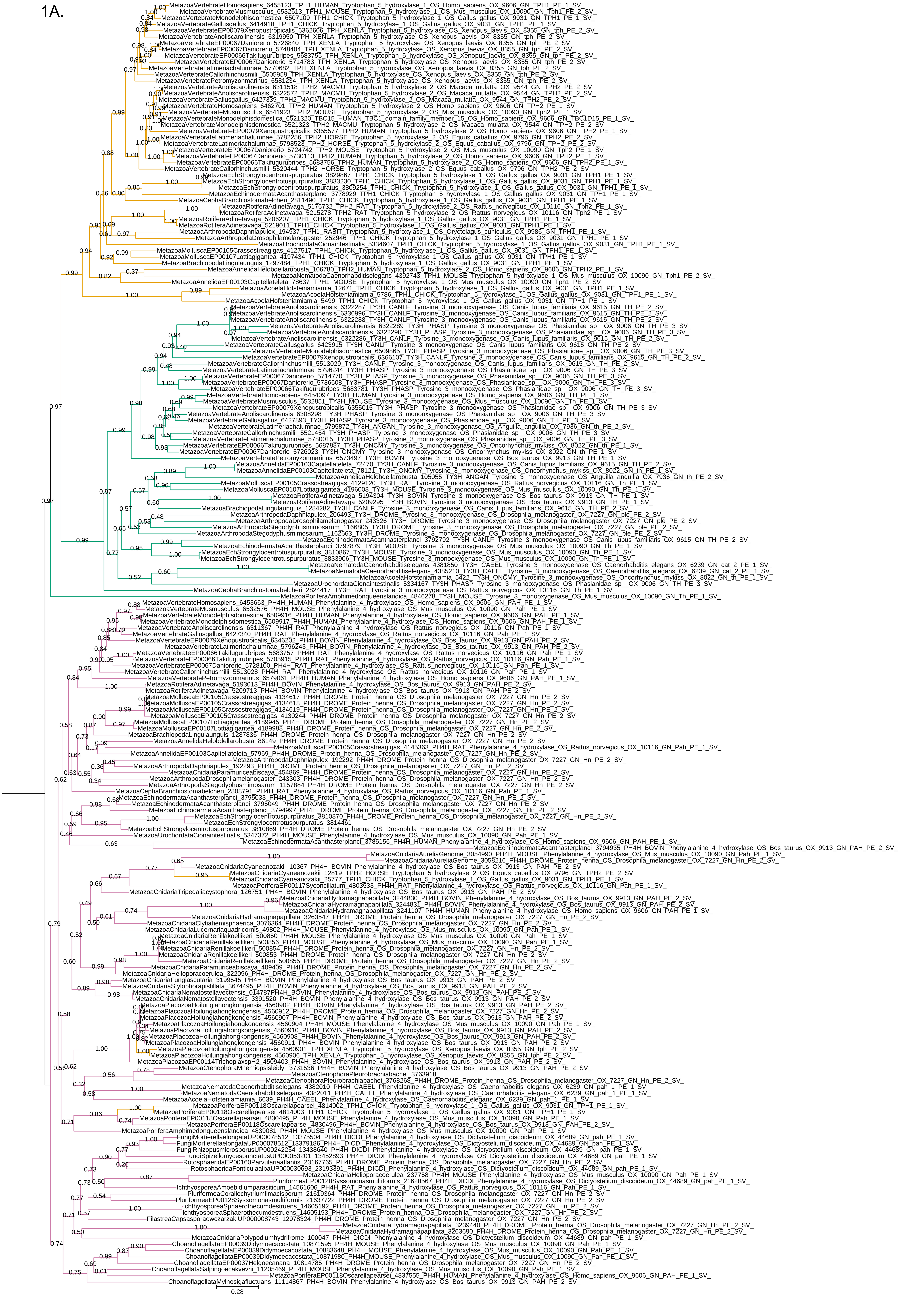

1B.

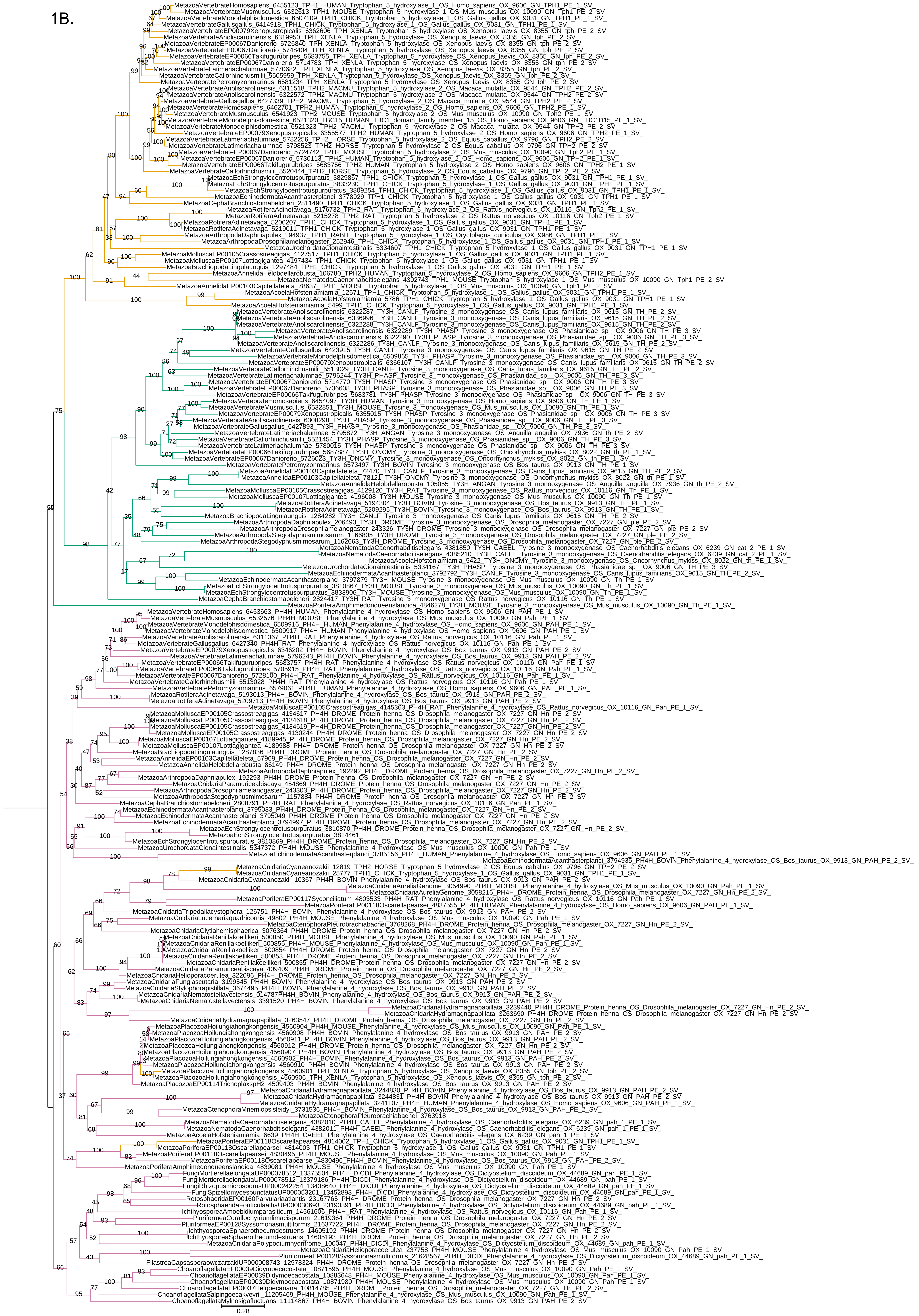

1C.

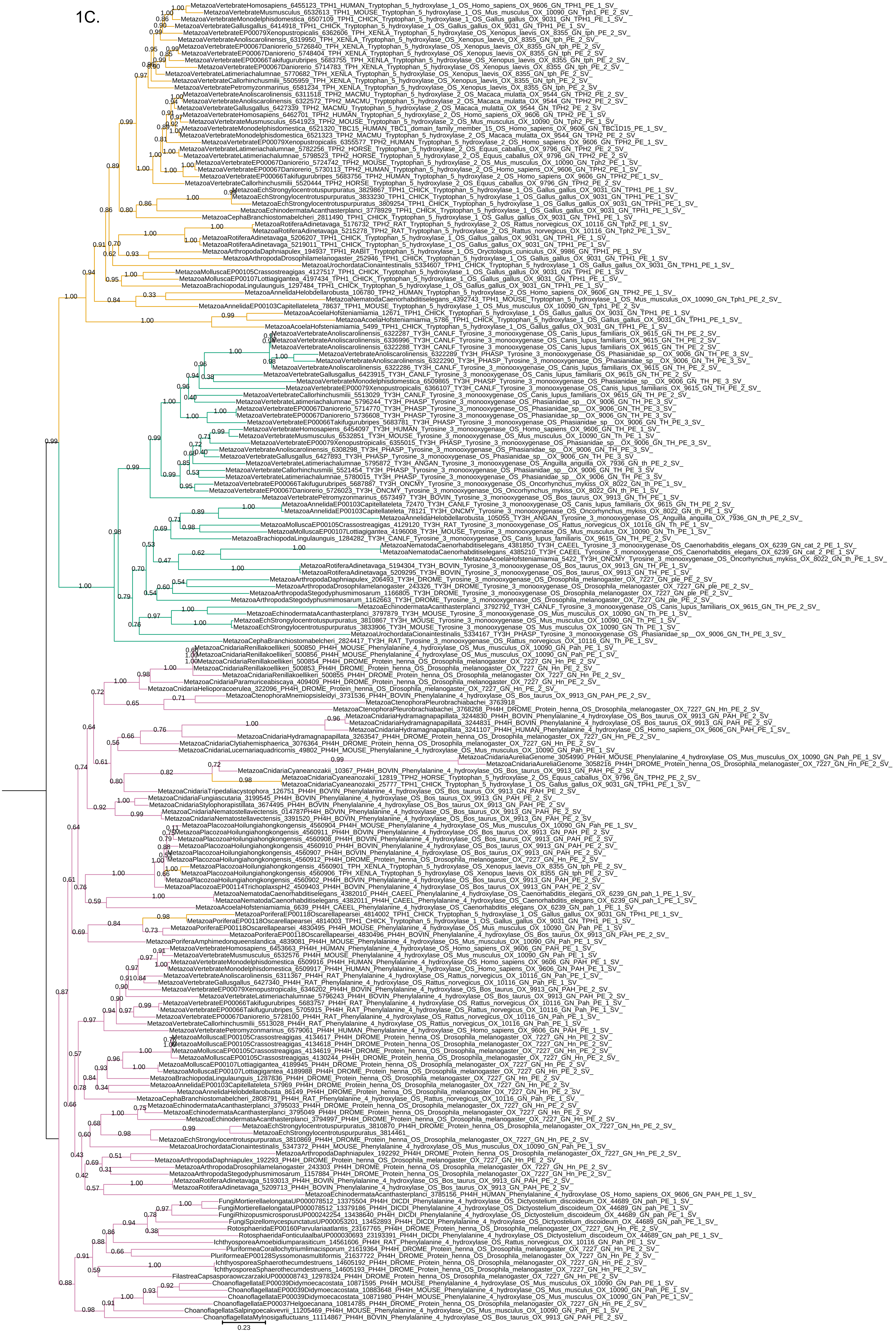

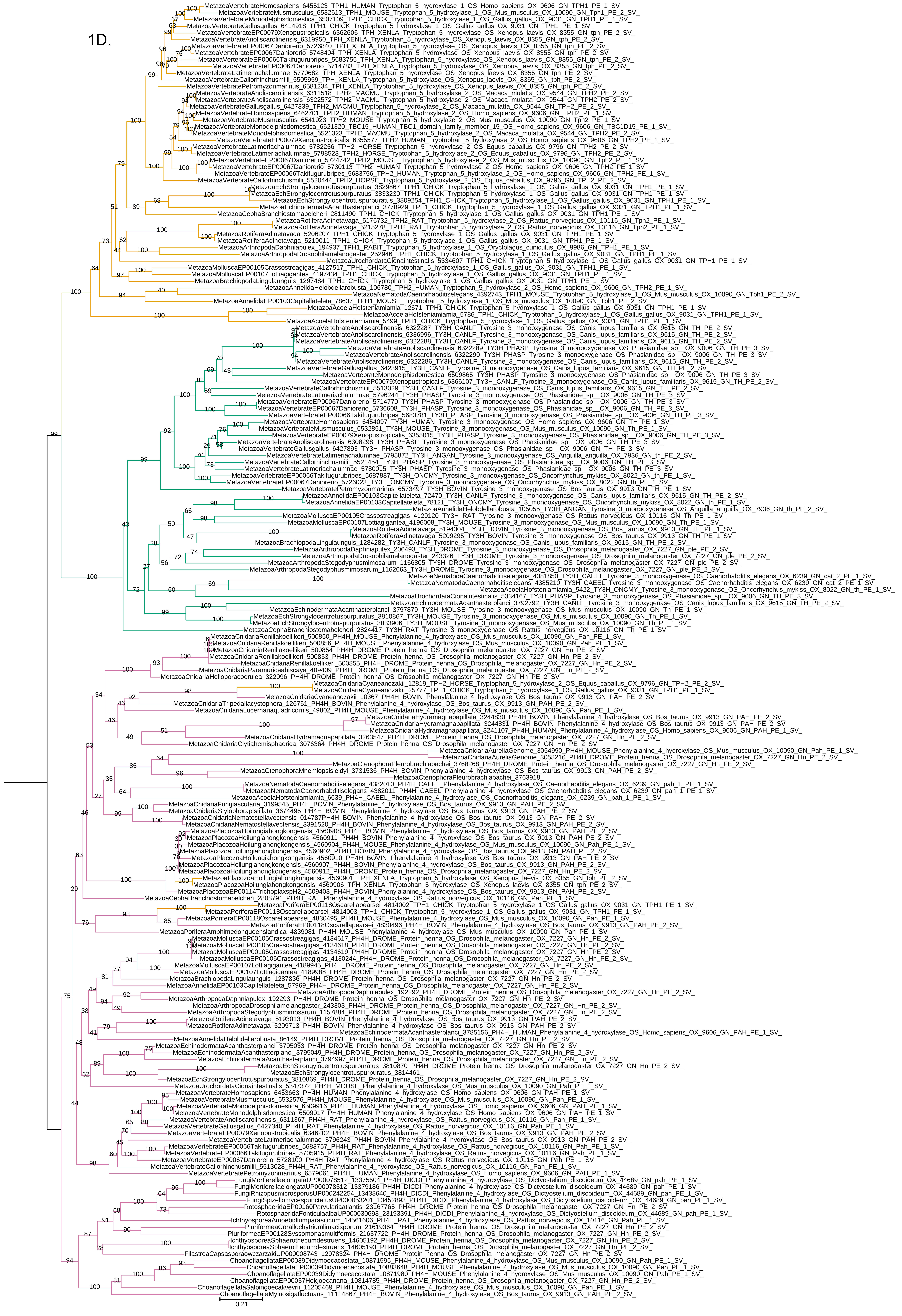
1D.
0.21

1E.

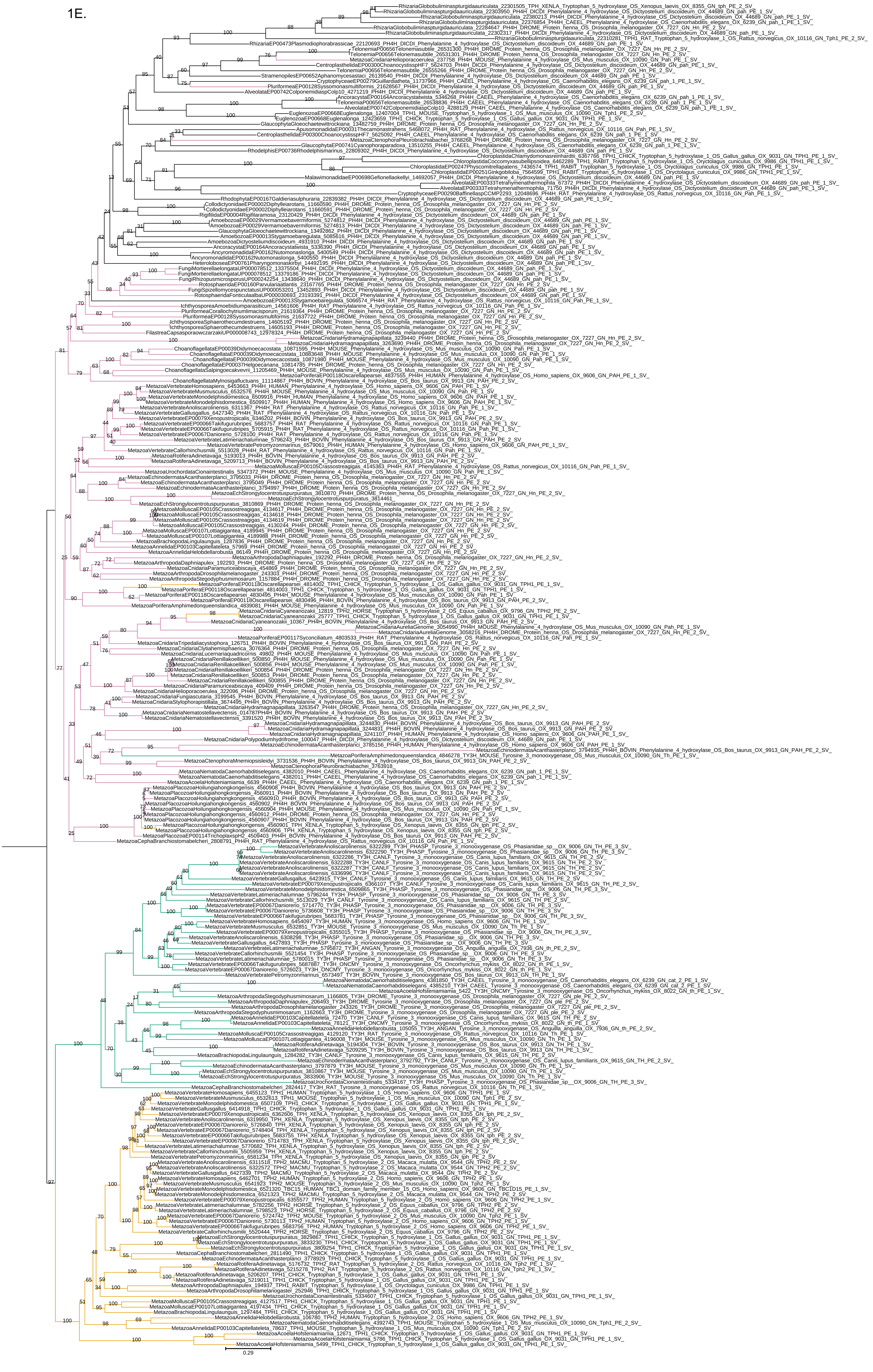

1F.

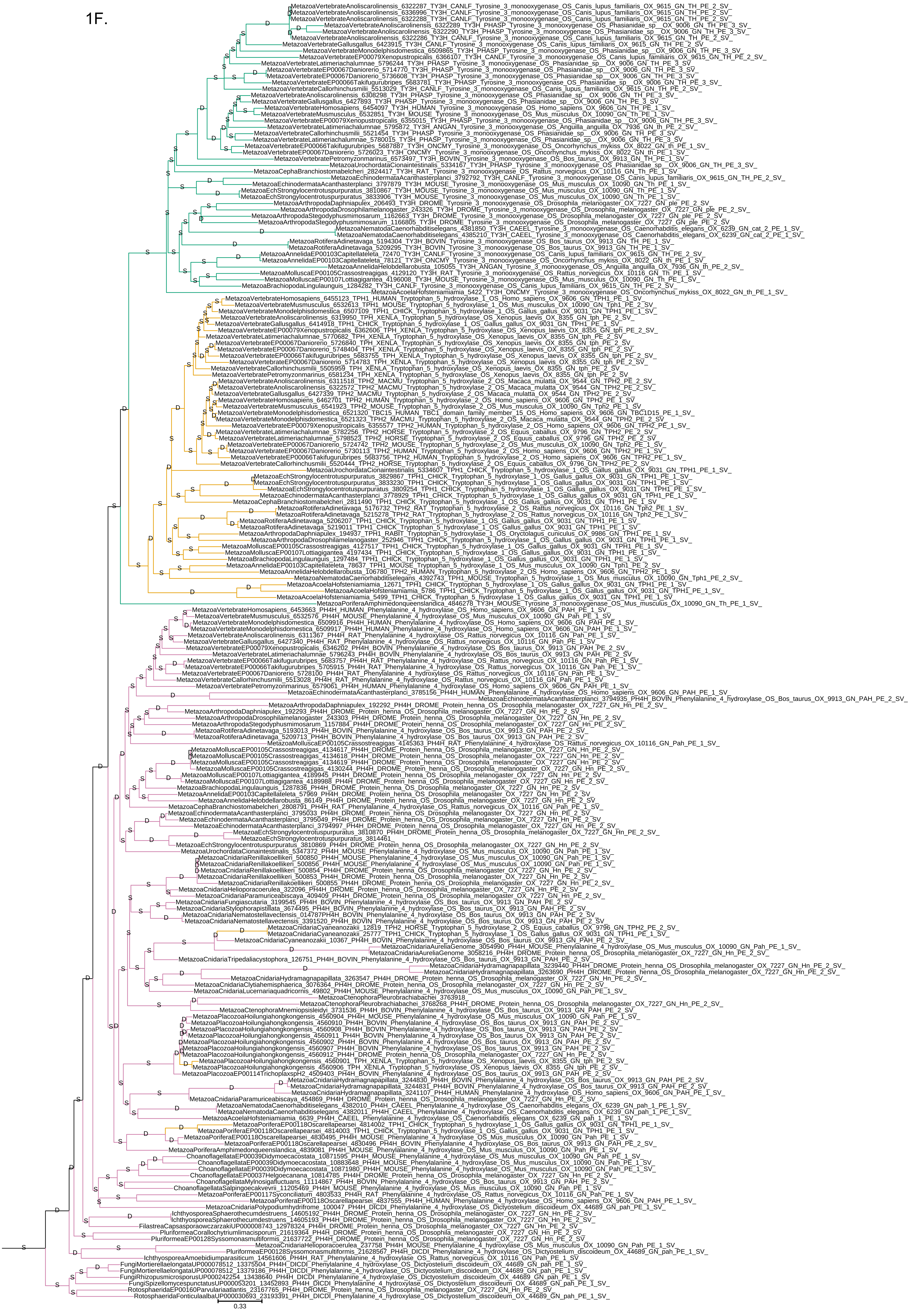

1G.

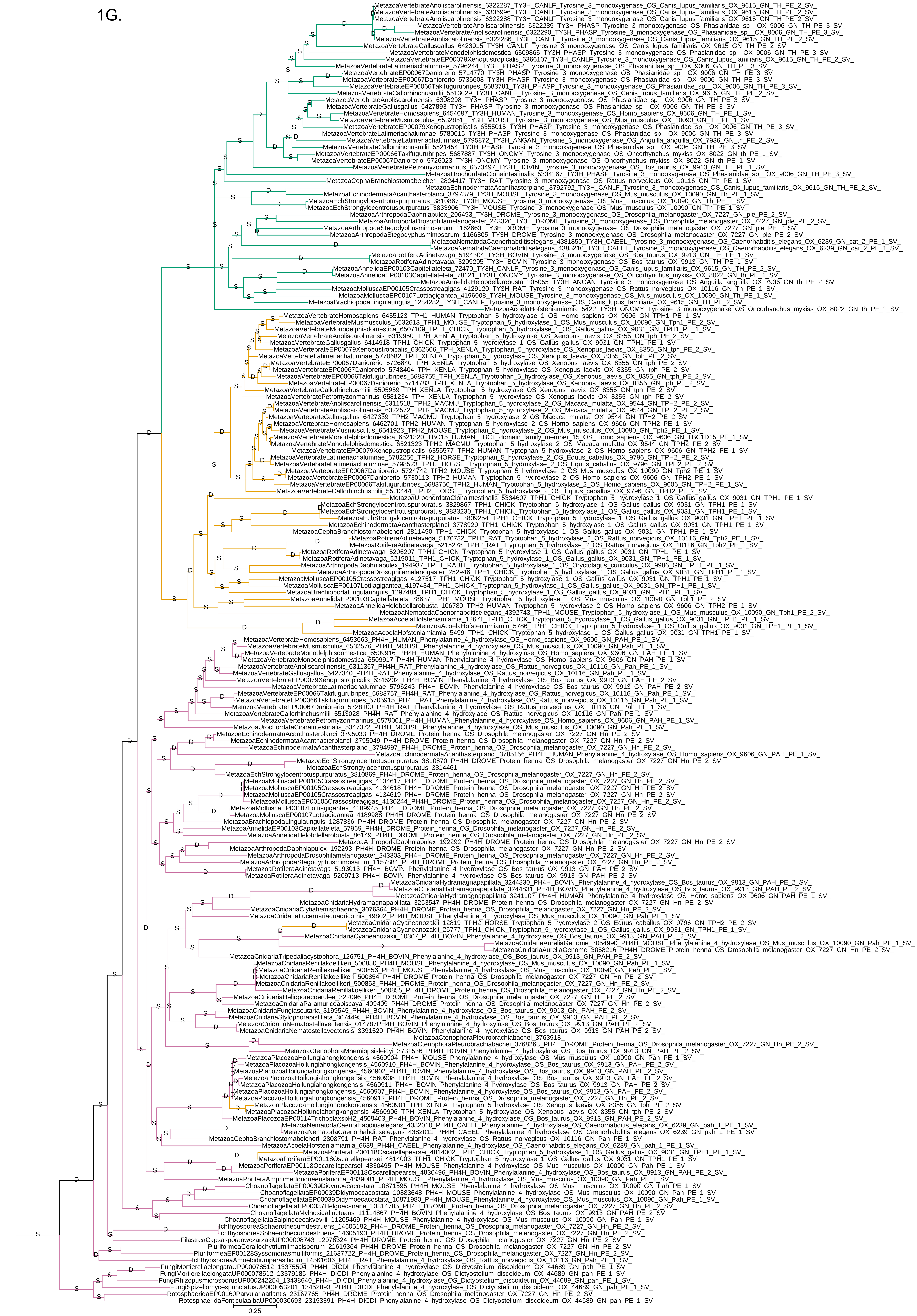

1H.

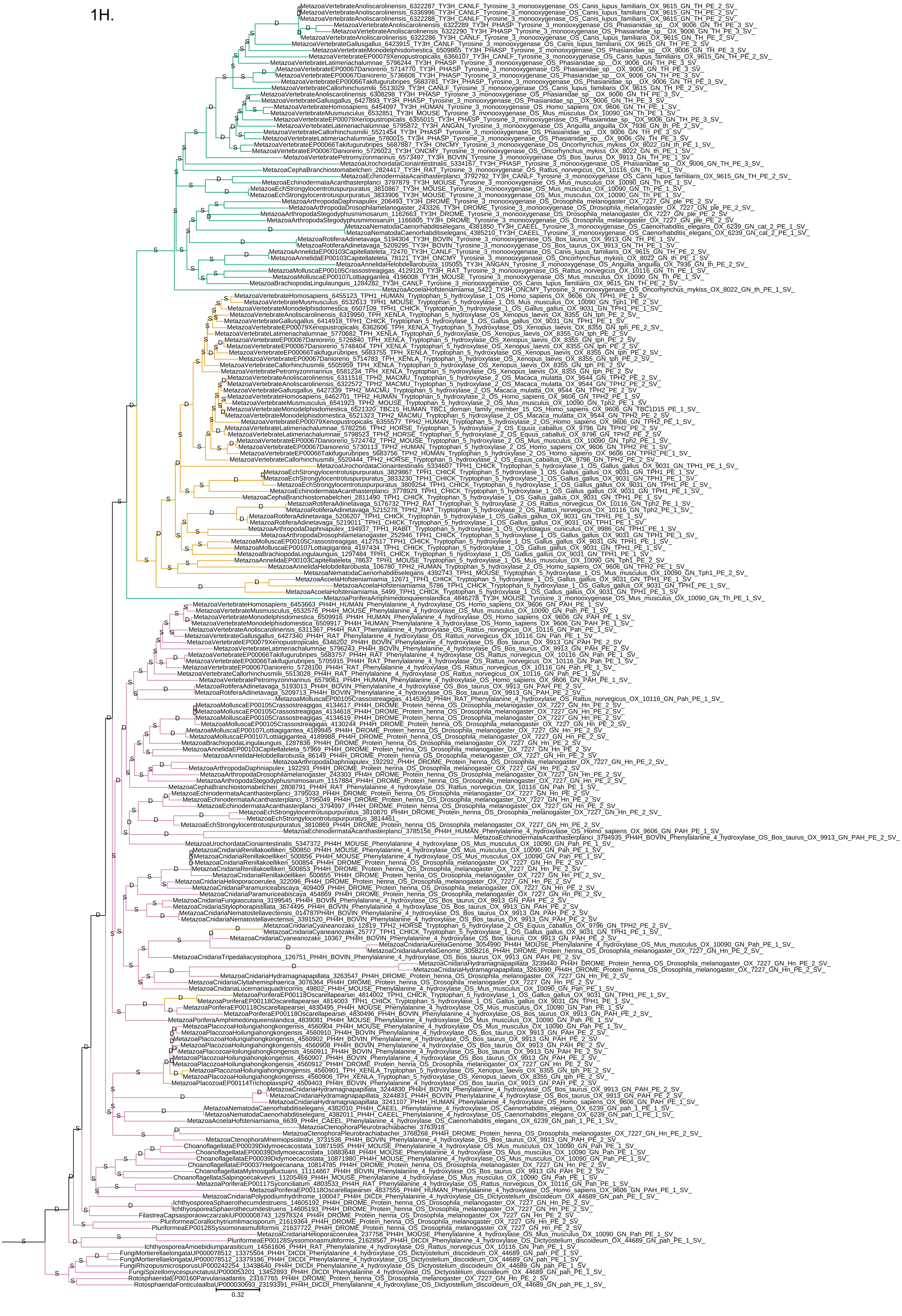

1I.

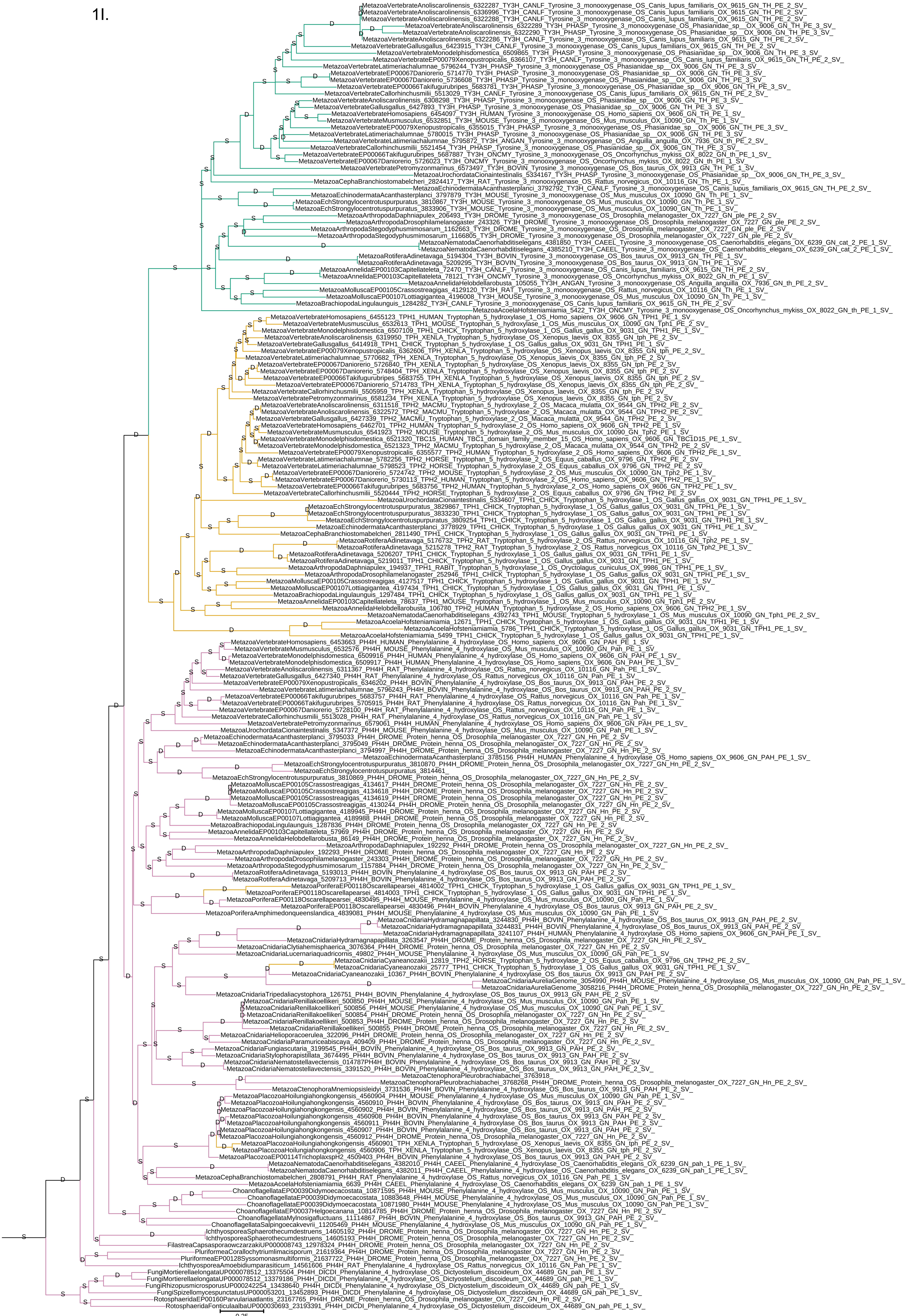

1J.

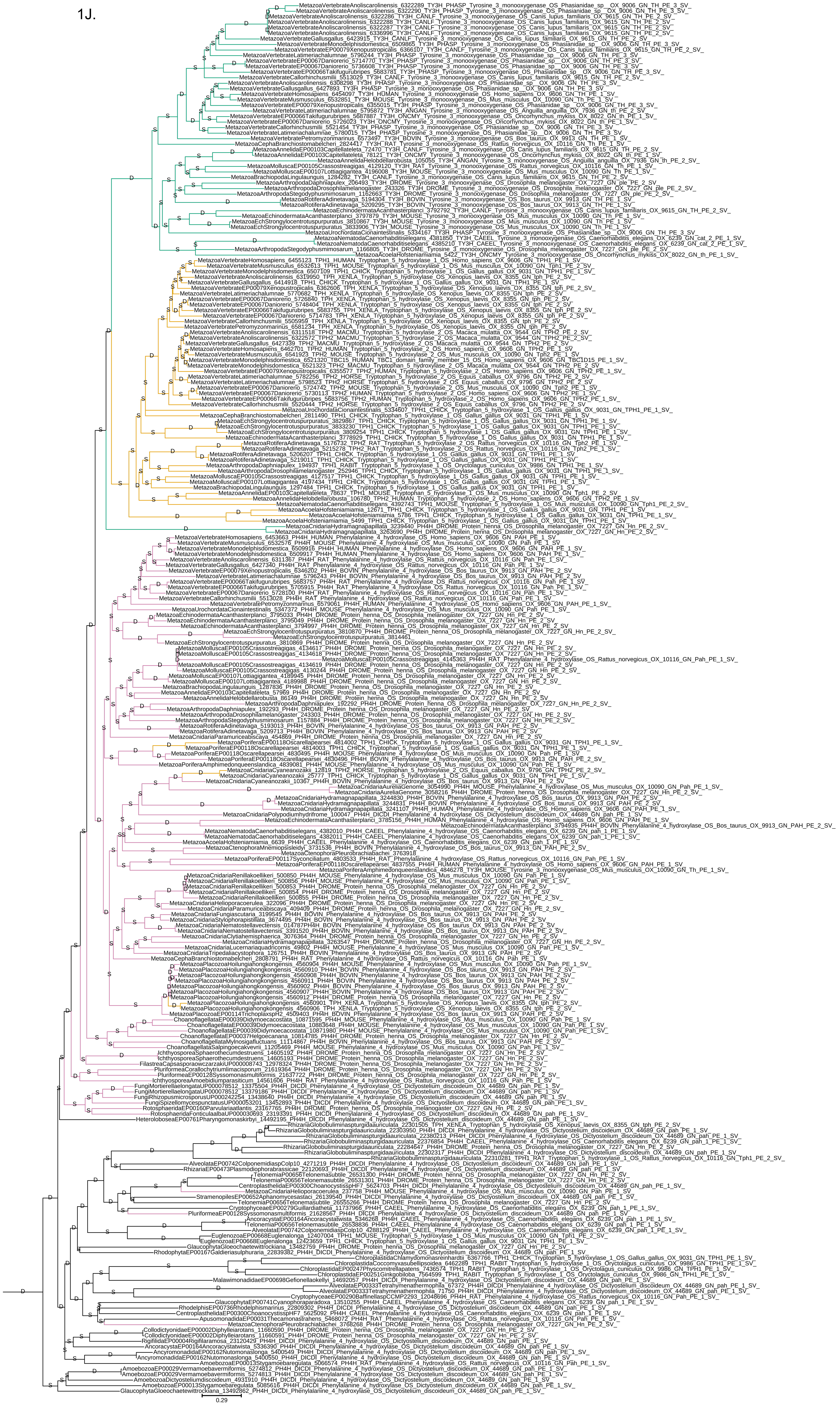

Supplementary Figure 2: Evolution of GCHs. Simplified illustration of gene tree to species tree reconciliation. Silhouettes obtained from Phylopic.org. Silhouette images are by Andrew R. Gehrke (*Hofstenia miamia*); Daniel Jaron (*Mus musculus*); Mali'o Kodis, photograph by Ching (http://www.flickr.com/photos/36302473@N03/) (*Chrysaora fuscescens*); Mario Quevedo (Asteriidae); Oliver Voigt (*Trichoplax adhaerens*); Ramiro Morales-Hojas (*Drosophila americana)*; Steven Haddock, Jellywatch.org (*Hormiphora californensis*); and Tess Linden (*Salpingoeca rosetta*). GCH = GTP cyclohydrolase.

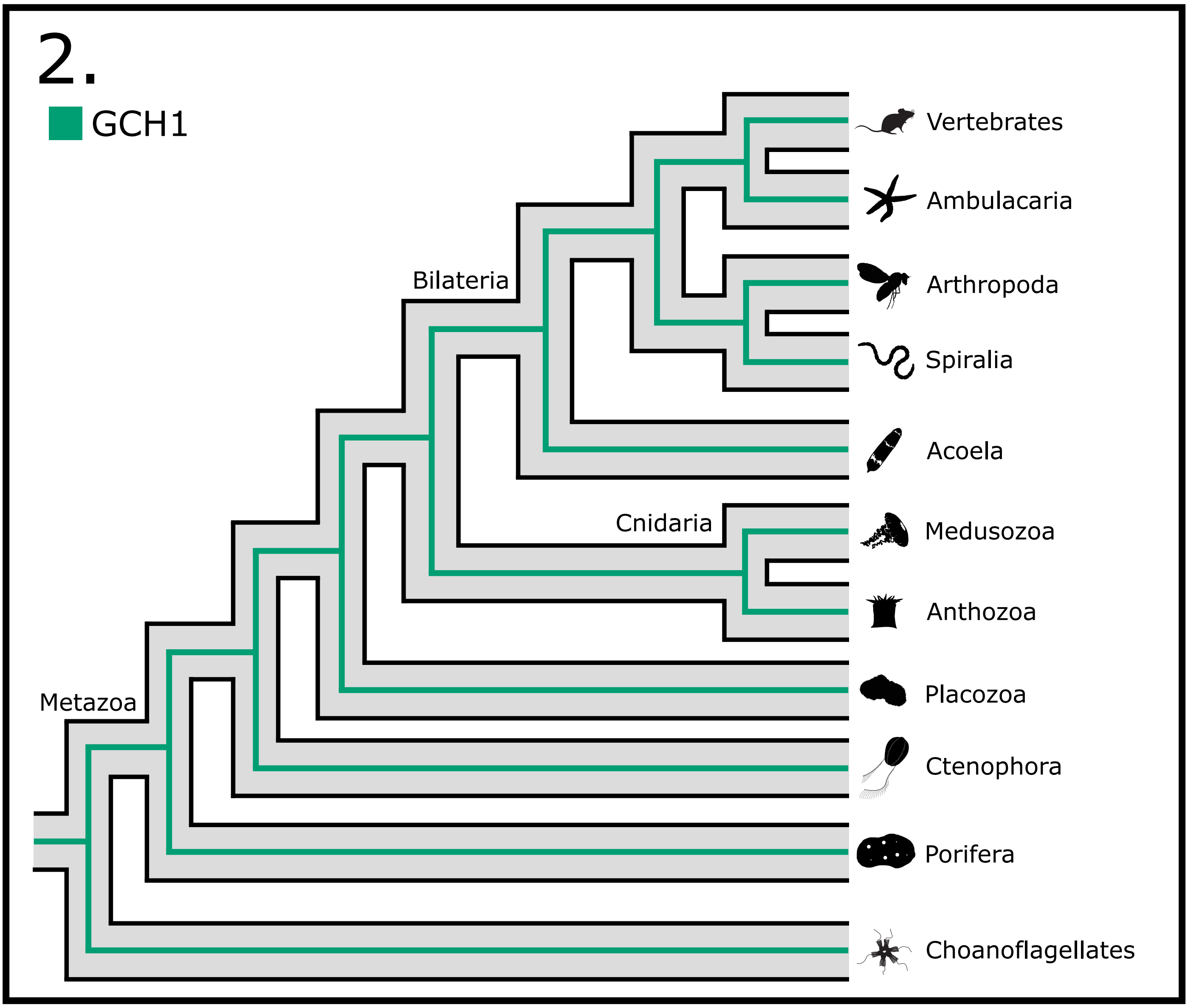
2.
GCH1
Bilateria
Cnidaria
Metazoa
Vertebrates
Ambulacaria
Arthropoda
Spiralia
Acoela
Medusozoa
Anthozoa
Placozoa
Ctenophora
Porifera
Choanoflagellates

Supplementary Figure 3: Analysis of GCHs. A) TBE tree with TBE score supports. B) Maximum likelihood tree with ultrafast bootstrap proportion supports. C) Maximum likelihood tree including all eukaryotic sequences with ultrafast bootstrap proportion supports. D) Reconciliation of using sponge-first hypothesis. E) Reconciliation using ctenophore-first hypothesis. F) Reconciliation of the UFB tree including all eukaryotic sequences. Nodes in reconciled trees indicated as speciation events (S) or duplication events (D). All trees shown as rooted, non-reconciled trees rooted arbitrarily for visualisation. GCH = GTP cyclo-hydrolase, TBE = transfer bootstrap expectation, UFB = ultrafast bootstrap. Branches colour coded by sequence annotation; green = GCH; and black = others and non-opithokont sequences. Source Data are available at [https://figshare.le.ac.uk/articles/dataset/Monoamine_Neuromodulation_is_a_Bilaterian_Innovation_Results/20391477]; in Results/GeneTrees labelled OG_GCH_TBE.tbe.tree and OG_GCH_UFB.treefile; in Results/GeneTrees/Eukaryotes_GeneTrees labelled OG_GCH_EUKIQ.treefile; in Results/Reconciliation/SpongeFirst labelled OG_GCH; in Results/Reconciliation/CtenophoreFirst labelled OG_GCH; and in Results/Reconciliation/Eukaryote labelled OG_GCH.

3A.

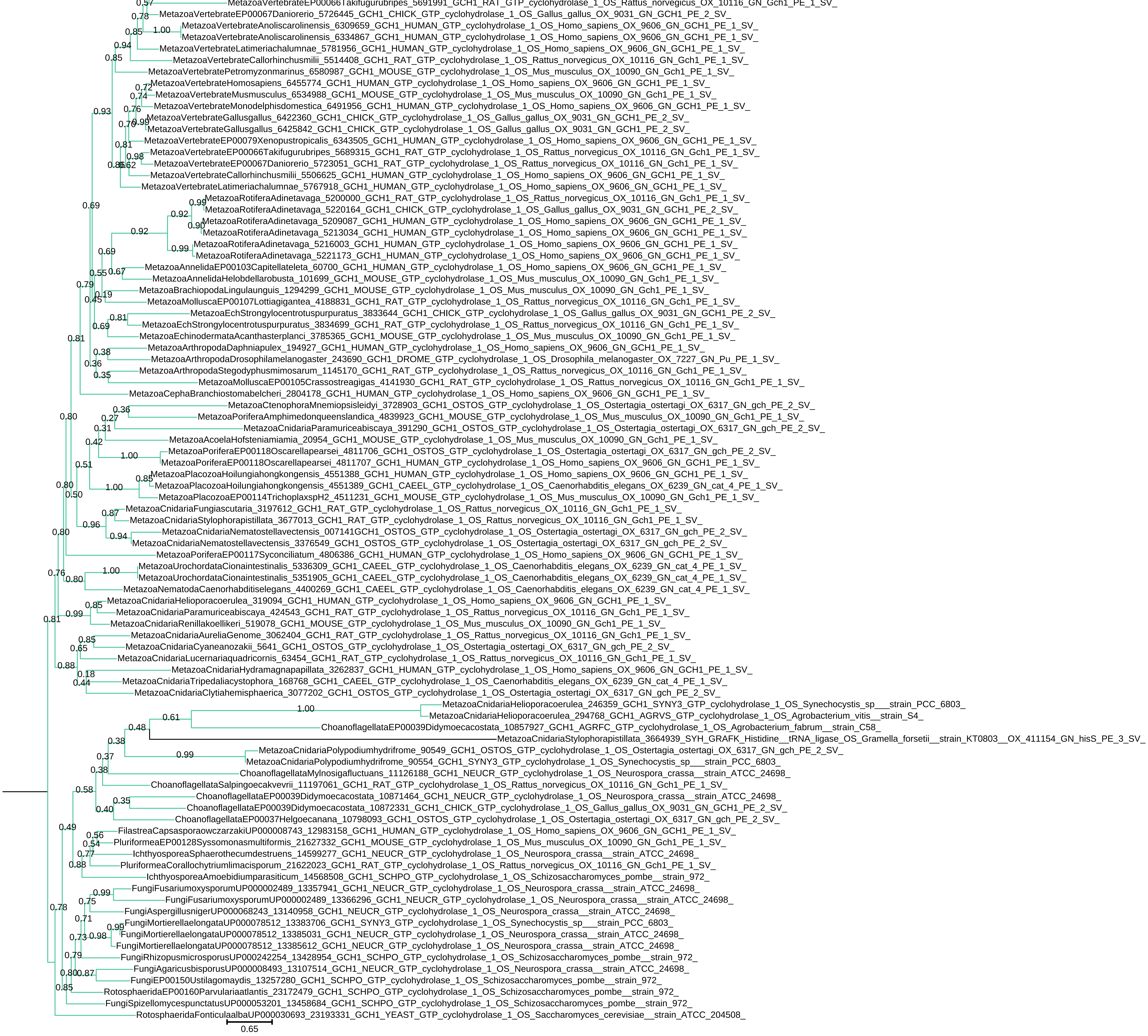
MetazoaVertebrateEP00066Takifugurubripes_5693553_GCH1_CHICK_GTP_cyclohydrolase_1_OS_Gallus_gallus_OX_9031_GN_GCH1_PE_2_SV_
MetazoaVertebrateEP00079Xenopustropicalis_6351672_GCH1_RAT_GTP_cyclohydrolase_1_OS_Rattus_norvegicus_OX_10116_GN_Gch1_PE_1_SV_
MetazoaVertebrateEP00066Takifugurubripes_5691991_GCH1_RAT_GTP_cyclohydrolase_1_OS_Rattus_norvegicus_OX_10116_GN_Gch1_PE_1_SV_
MetazoaVertebrateEP00067Daniorerio_5726445_GCH1_CHICK_GTP_cyclohydrolase_1_OS_Gallus_gallus_OX_9031_GN_GCH1_PE_2_SV_
MetazoaVertebrateAnoliscarolinensis_6309659_GCH1_HUMAN_GTP_cyclohydrolase_1_OS_Homo_sapiens_OX_9606_GN_GCH1_PE_1_SV_
MetazoaVertebrateAnoliscarolinensis_6334867_GCH1_HUMAN_GTP_cyclohydrolase_1_OS_Homo_sapiens_OX_9606_GN_GCH1_PE_1_SV_
MetazoaVertebrateLatimeriachalumnae_5781956_GCH1_HUMAN_GTP_cyclohydrolase_1_OS_Homo_sapiens_OX_9606_GN_GCH1_PE_1_SV_
MetazoaVertebrateCallorhinchusmilii_5514408_GCH1_RAT_GTP_cyclohydrolase_1_OS_Rattus_norvegicus_OX_10116_GN_Gch1_PE_1_SV_
MetazoaVertebratePetromyzonmarinus_6580987_GCH1_MOUSE_GTP_cyclohydrolase_1_OS_Mus_musculus_OX_10090_GN_Gch1_PE_1_SV_
MetazoaVertebrateHomosapiens_6455774_GCH1_HUMAN_GTP_cyclohydrolase_1_OS_Homo_sapiens_OX_9606_GN_GCH1_PE_1_SV_
MetazoaVertebrateMusmusculus_6534988_GCH1_MOUSE_GTP_cyclohydrolase_1_OS_Mus_musculus_OX_10090_GN_Gch1_PE_1_SV_
MetazoaVertebrateMonodelphisdomestica_6491956_GCH1_HUMAN_GTP_cyclohydrolase_1_OS_Homo_sapiens_OX_9606_GN_GCH1_PE_1_SV_
MetazoaVertebrateGallusgallus_6422360_GCH1_CHICK_GTP_cyclohydrolase_1_OS_Gallus_gallus_OX_9031_GN_GCH1_PE_2_SV_
MetazoaVertebrateGallusgallus_6425842_GCH1_CHICK_GTP_cyclohydrolase_1_OS_Gallus_gallus_OX_9031_GN_GCH1_PE_2_SV_
MetazoaVertebrateEP00079Xenopustropicalis_6343505_GCH1_HUMAN_GTP_cyclohydrolase_1_OS_Homo_sapiens_OX_9606_GN_GCH1_PE_1_SV_
MetazoaVertebrateEP00066Takifugurubripes_5689315_GCH1_RAT_GTP_cyclohydrolase_1_OS_Rattus_norvegicus_OX_10116_GN_Gch1_PE_1_SV_
MetazoaVertebrateEP00067Daniorerio_5723051_GCH1_RAT_GTP_cyclohydrolase_1_OS_Rattus_norvegicus_OX_10116_GN_Gch1_PE_1_SV_
MetazoaVertebrateCallorhinchusmilii_5506625_GCH1_HUMAN_GTP_cyclohydrolase_1_OS_Homo_sapiens_OX_9606_GN_GCH1_PE_1_SV_
MetazoaVertebrateLatimeriachalumnae_5767918_GCH1_HUMAN_GTP_cyclohydrolase_1_OS_Homo_sapiens_OX_9606_GN_GCH1_PE_1_SV_
MetazoaRotiferaAdinetavaga_5200000_GCH1_RAT_GTP_cyclohydrolase_1_OS_Rattus_norvegicus_OX_10116_GN_Gch1_PE_1_SV_
MetazoaRotiferaAdinetavaga_5220164_GCH1_CHICK_GTP_cyclohydrolase_1_OS_Gallus_gallus_OX_9031_GN_GCH1_PE_2_SV_
MetazoaRotiferaAdinetavaga_5209087_GCH1_HUMAN_GTP_cyclohydrolase_1_OS_Homo_sapiens_OX_9606_GN_GCH1_PE_1_SV_
MetazoaRotiferaAdinetavaga_5213034_GCH1_HUMAN_GTP_cyclohydrolase_1_OS_Homo_sapiens_OX_9606_GN_GCH1_PE_1_SV_
MetazoaRotiferaAdinetavaga_5216003_GCH1_HUMAN_GTP_cyclohydrolase_1_OS_Homo_sapiens_OX_9606_GN_GCH1_PE_1_SV_
MetazoaRotiferaAdinetavaga_5221173_GCH1_HUMAN_GTP_cyclohydrolase_1_OS_Homo_sapiens_OX_9606_GN_GCH1_PE_1_SV_
MetazoaAnnelidaEP00103Capitellateleta_60700_GCH1_HUMAN_GTP_cyclohydrolase_1_OS_Homo_sapiens_OX_9606_GN_GCH1_PE_1_SV_
MetazoaAnnelidaHelobdellarobusta_101699_GCH1_MOUSE_GTP_cyclohydrolase_1_OS_Mus_musculus_OX_10090_GN_Gch1_PE_1_SV_
MetazoaBrachiopodaLingulaunguis_1294299_GCH1_MOUSE_GTP_cyclohydrolase_1_OS_Mus_musculus_OX_10090_GN_Gch1_PE_1_SV_
MetazoaMolluscaEP00107Lottiagigantea_4188831_GCH1_RAT_GTP_cyclohydrolase_1_OS_Rattus_norvegicus_OX_10116_GN_Gch1_PE_1_SV_
MetazoaEchStrongylocentrotuspurpuratus_3833644_GCH1_CHICK_GTP_cyclohydrolase_1_OS_Gallus_gallus_OX_9031_GN_GCH1_PE_2_SV_
MetazoaEchStrongylocentrotuspurpuratus_3834699_GCH1_RAT_GTP_cyclohydrolase_1_OS_Rattus_norvegicus_OX_10116_GN_Gch1_PE_1_SV_
MetazoaEchinodermataAcanthasterplanci_3785365_GCH1_MOUSE_GTP_cyclohydrolase_1_OS_Mus_musculus_OX_10090_GN_Gch1_PE_1_SV_
MetazoaArthropodaDaphniapulex_194927_GCH1_HUMAN_GTP_cyclohydrolase_1_OS_Homo_sapiens_OX_9606_GN_GCH1_PE_1_SV_
MetazoaArthropodaDrosophilamelanogaster_243690_GCH1_DROME_GTP_cyclohydrolase_1_OS_Drosophila_melanogaster_OX_7227_GN_Pu_PE_1_SV_
MetazoaArthropodaStegodyphusmimosarum_1145170_GCH1_RAT_GTP_cyclohydrolase_1_OS_Rattus_norvegicus_OX_10116_GN_Gch1_PE_1_SV_
MetazoaMolluscaEP00105Crassostreagigas_4141930_GCH1_RAT_GTP_cyclohydrolase_1_OS_Rattus_norvegicus_OX_10116_GN_Gch1_PE_1_SV_
MetazoaCephaBranchiostomabelcheri_2804178_GCH1_HUMAN_GTP_cyclohydrolase_1_OS_Homo_sapiens_OX_9606_GN_GCH1_PE_1_SV_
MetazoaCtenophoraMnemiopsisleidyi_3728903_GCH1_OSTOS_GTP_cyclohydrolase_1_OS_Ostertagia_ostertagi_OX_6317_GN_gch_PE_2_SV_
MetazoaPoriferaAmphimedonqueenslandica_4839923_GCH1_MOUSE_GTP_cyclohydrolase_1_OS_Mus_musculus_OX_10090_GN_Gch1_PE_1_SV_
MetazoaCnidariaParamuriceabiscaya_391290_GCH1_OSTOS_GTP_cyclohydrolase_1_OS_Ostertagia_ostertagi_OX_6317_GN_gch_PE_2_SV_
MetazoaAcoelaHofsteniamiamia_20954_GCH1_MOUSE_GTP_cyclohydrolase_1_OS_Mus_musculus_OX_10090_GN_Gch1_PE_1_SV_
MetazoaPoriferaEP00118Oscarellapearsei_4811706_GCH1_OSTOS_GTP_cyclohydrolase_1_OS_Ostertagia_ostertagi_OX_6317_GN_gch_PE_2_SV_
MetazoaPoriferaEP00118Oscarellapearsei_4811707_GCH1_HUMAN_GTP_cyclohydrolase_1_OS_Homo_sapiens_OX_9606_GN_GCH1_PE_1_SV_
MetazoaPlacozoaHoilungiahongkongensis_4551388_GCH1_HUMAN_GTP_cyclohydrolase_1_OS_Homo_sapiens_OX_9606_GN_GCH1_PE_1_SV_
MetazoaPlacozoaHoilungiahongkongensis_4551389_GCH1_CAEEL_GTP_cyclohydrolase_1_OS_Caenorhabditis_elegans_OX_6239_GN_cat_4_PE_1_SV_
MetazoaPlacozoaEP00114TrichoplaxspH2_4511231_GCH1_MOUSE_GTP_cyclohydrolase_1_OS_Mus_musculus_OX_10090_GN_Gch1_PE_1_SV_
MetazoaCnidariaFungiascutaria_3197612_GCH1_RAT_GTP_cyclohydrolase_1_OS_Rattus_norvegicus_OX_10116_GN_Gch1_PE_1_SV_
MetazoaCnidariaStylophorapistillata_3677013_GCH1_RAT_GTP_cyclohydrolase_1_OS_Rattus_norvegicus_OX_10116_GN_Gch1_PE_1_SV_
MetazoaCnidariaNematostellavectensis_007141GCH1_OSTOS_GTP_cyclohydrolase_1_OS_Ostertagia_ostertagi_OX_6317_GN_gch_PE_2_SV_
MetazoaCnidariaNematostellavectensis_3376549_GCH1_OSTOS_GTP_cyclohydrolase_1_OS_Ostertagia_ostertagi_OX_6317_GN_gch_PE_2_SV_
MetazoaPoriferaEP00117Syconciliatum_4806386_GCH1_HUMAN_GTP_cyclohydrolase_1_OS_Homo_sapiens_OX_9606_GN_GCH1_PE_1_SV_
MetazoaUrochordataCionaintestinalis_5336309_GCH1_CAEEL_GTP_cyclohydrolase_1_OS_Caenorhabditis_elegans_OX_6239_GN_cat_4_PE_1_SV_
MetazoaUrochordataCionaintestinalis_5351905_GCH1_CAEEL_GTP_cyclohydrolase_1_OS_Caenorhabditis_elegans_OX_6239_GN_cat_4_PE_1_SV_
MetazoaNematodaCaenorhabditiselegans_4400269_GCH1_CAEEL_GTP_cyclohydrolase_1_OS_Caenorhabditis_elegans_OX_6239_GN_cat_4_PE_1_SV_
MetazoaCnidariaHelioporacoerulea_319094_GCH1_HUMAN_GTP_cyclohydrolase_1_OS_Homo_sapiens_OX_9606_GN_GCH1_PE_1_SV_
MetazoaCnidariaParamuriceabiscaya_424543_GCH1_RAT_GTP_cyclohydrolase_1_OS_Rattus_norvegicus_OX_10116_GN_Gch1_PE_1_SV_
MetazoaCnidariaRenillakoellikeri_519078_GCH1_MOUSE_GTP_cyclohydrolase_1_OS_Mus_musculus_OX_10090_GN_Gch1_PE_1_SV_
MetazoaCnidariaAureliaGenome_3062404_GCH1_RAT_GTP_cyclohydrolase_1_OS_Rattus_norvegicus_OX_10116_GN_Gch1_PE_1_SV_
MetazoaCnidariaCyaneanozakii_5641_GCH1_OSTOS_GTP_cyclohydrolase_1_OS_Ostertagia_ostertagi_OX_6317_GN_gch_PE_2_SV_
MetazoaCnidariaLucernariaquadricornis_63454_GCH1_RAT_GTP_cyclohydrolase_1_OS_Rattus_norvegicus_OX_10116_GN_Gch1_PE_1_SV_
MetazoaCnidariaHydramagnapapillata_3262837_GCH1_HUMAN_GTP_cyclohydrolase_1_OS_Homo_sapiens_OX_9606_GN_GCH1_PE_1_SV_
MetazoaCnidariaTripedaliacystophora_168768_GCH1_CAEEL_GTP_cyclohydrolase_1_OS_Caenorhabditis_elegans_OX_6239_GN_cat_4_PE_1_SV_
MetazoaCnidariaClytiahemisphaerica_3077202_GCH1_OSTOS_GTP_cyclohydrolase_1_OS_Ostertagia_ostertagi_OX_6317_GN_gch_PE_2_SV_
MetazoaCnidariaHelioporacoerulea_246359_GCH1_SYNY3_GTP_cyclohydrolase_1_OS_Synechocystis_sp___strain_PCC_6803_
MetazoaCnidariaHelioporacoerulea_294768_GCH1_AGRVS_GTP_cyclohydrolase_1_OS_Agrobacterium_vitis__strain_S4_
ChoanoflagellataEP00039Didymoecacostata_10857927_GCH1_AGRFC_GTP_cyclohydrolase_1_OS_Agrobacterium_fabrum__strain_C58_
MetazoaCnidariaStylophorapistillata_3664939_SYH_GRAFK_Histidine__tRNA_ligase_OS_Gramella_forsetii__strain_KT0803__OX_411154_GN_hisS_PE_3_SV_
MetazoaCnidariaPolypodiumhydrifrome_90549_GCH1_OSTOS_GTP_cyclohydrolase_1_OS_Ostertagia_ostertagi_OX_6317_GN_gch_PE_2_SV_
MetazoaCnidariaPolypodiumhydrifrome_90554_GCH1_SYNY3_GTP_cyclohydrolase_1_OS_Synechocystis_sp___strain_PCC_6803_
ChoanoflagellataMylnosigafluctuans_11126188_GCH1_NEUCR_GTP_cyclohydrolase_1_OS_Neurospora_crassa__strain_ATCC_24698_
ChoanoflagellataSalpingoecakvevrii_11197061_GCH1_RAT_GTP_cyclohydrolase_1_OS_Rattus_norvegicus_OX_10116_GN_Gch1_PE_1_SV_
ChoanoflagellataEP00039Didymoecacostata_10871464_GCH1_NEUCR_GTP_cyclohydrolase_1_OS_Neurospora_crassa__strain_ATCC_24698_
ChoanoflagellataEP00039Didymoecacostata_10872331_GCH1_CHICK_GTP_cyclohydrolase_1_OS_Gallus_gallus_OX_9031_GN_GCH1_PE_2_SV_
ChoanoflagellataEP00037Helgoecanana_10798093_GCH1_OSTOS_GTP_cyclohydrolase_1_OS_Ostertagia_ostertagi_OX_6317_GN_gch_PE_2_SV_
FilastreaCapsasporaowczarzakiUP000008743_12983158_GCH1_HUMAN_GTP_cyclohydrolase_1_OS_Homo_sapiens_OX_9606_GN_GCH1_PE_1_SV_
PluriformeaEP00128Syssomonasmultiformis_21627332_GCH1_MOUSE_GTP_cyclohydrolase_1_OS_Mus_musculus_OX_10090_GN_Gch1_PE_1_SV_
IchthyosporeaSphaerothecumdestruens_14599277_GCH1_NEUCR_GTP_cyclohydrolase_1_OS_Neurospora_crassa__strain_ATCC_24698_
PluriformeaCorallochytriumlimacisporum_21622023_GCH1_RAT_GTP_cyclohydrolase_1_OS_Rattus_norvegicus_OX_10116_GN_Gch1_PE_1_SV_
IchthyosporeaAmoebidiumparasiticum_14568508_GCH1_SCHPO_GTP_cyclohydrolase_1_OS_Schizosaccharomyces_pombe__strain_972_
FungiFusariumoxysporumUP000002489_13357941_GCH1_NEUCR_GTP_cyclohydrolase_1_OS_Neurospora_crassa__strain_ATCC_24698_
FungiFusariumoxysporumUP000002489_13366296_GCH1_NEUCR_GTP_cyclohydrolase_1_OS_Neurospora_crassa__strain_ATCC_24698_
FungiAspergillusnigerUP000068243_13140958_GCH1_NEUCR_GTP_cyclohydrolase_1_OS_Neurospora_crassa__strain_ATCC_24698_
FungiMortierellaelongataUP000078512_13383706_GCH1_SYNY3_GTP_cyclohydrolase_1_OS_Synechocystis_sp___strain_PCC_6803_
FungiMortierellaelongataUP000078512_13385031_GCH1_NEUCR_GTP_cyclohydrolase_1_OS_Neurospora_crassa__strain_ATCC_24698_
FungiMortierellaelongataUP000078512_13385612_GCH1_NEUCR_GTP_cyclohydrolase_1_OS_Neurospora_crassa__strain_ATCC_24698_
FungiRhizopusmicrosporusUP000242254_13428954_GCH1_SCHPO_GTP_cyclohydrolase_1_OS_Schizosaccharomyces_pombe__strain_972_
FungiAgaricusbisporusUP000008493_13107514_GCH1_NEUCR_GTP_cyclohydrolase_1_OS_Neurospora_crassa__strain_ATCC_24698_
FungiEP00150Ustilagomaydis_13257280_GCH1_SCHPO_GTP_cyclohydrolase_1_OS_Schizosaccharomyces_pombe__strain_972_
RotosphaeridaEP00160Parvulariaatlantis_23172479_GCH1_SCHPO_GTP_cyclohydrolase_1_OS_Schizosaccharomyces_pombe__strain_972_
FungiSpizellomycespunctatusUP000053201_13458684_GCH1_SCHPO_GTP_cyclohydrolase_1_OS_Schizosaccharomyces_pombe__strain_972_
RotosphaeridaFonticulaalbaUP000030693_23193331_GCH1_YEAST_GTP_cyclohydrolase_1_OS_Saccharomyces_cerevisiae__strain_ATCC_204508_
0.65

3B.

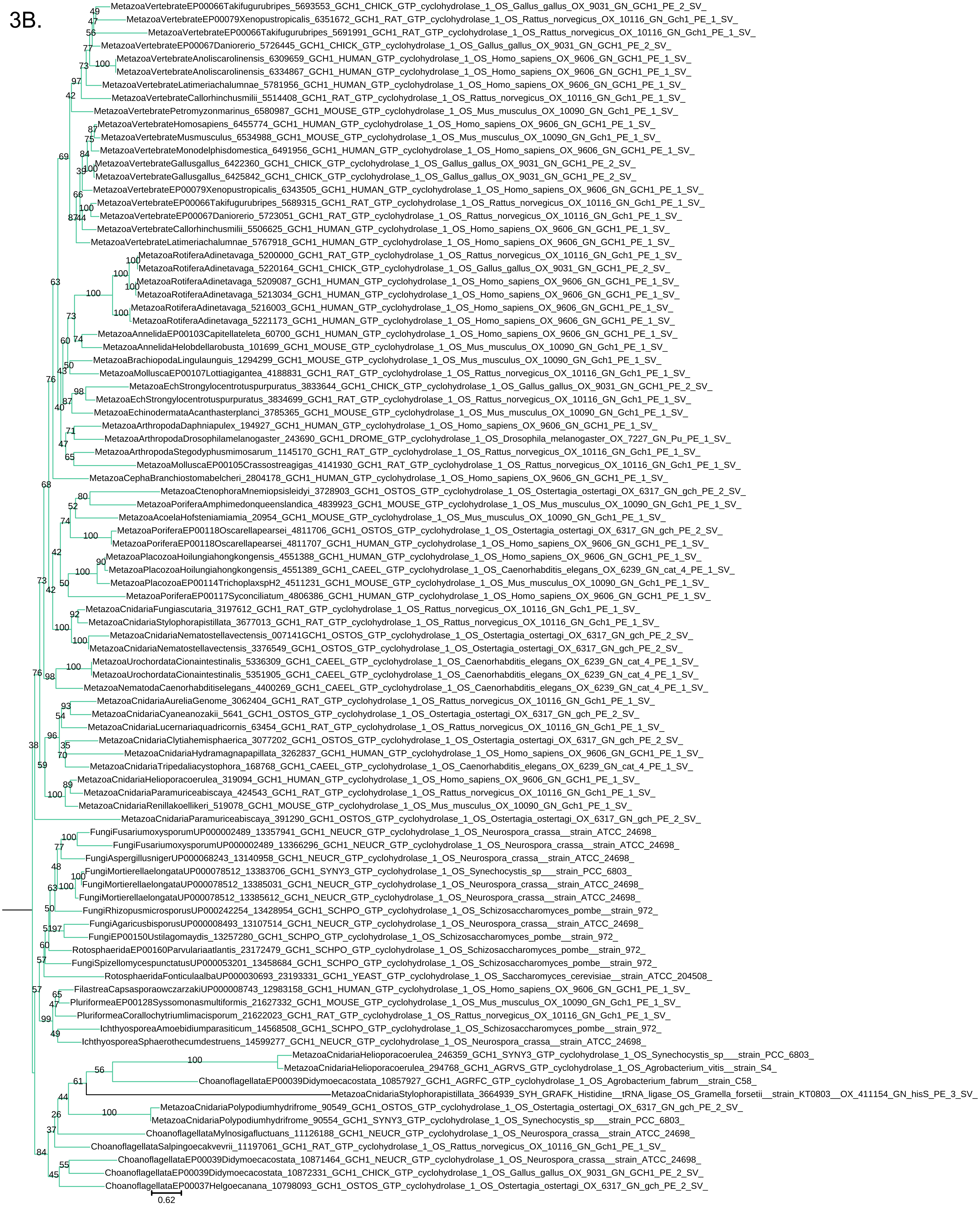
MetazoaVertebrateEP00066Takifugurubripes_5693553_GCH1_CHICK_GTP_cyclohydrolase_1_OS_Gallus_gallus_OX_9031_GN_GCH1_PE_2_SV_
MetazoaVertebrateEP00079Xenopustropicalis_6351672_GCH1_RAT_GTP_cyclohydrolase_1_OS_Rattus_norvegicus_OX_10116_GN_Gch1_PE_1_SV_
MetazoaVertebrateEP00066Takifugurubripes_5691991_GCH1_RAT_GTP_cyclohydrolase_1_OS_Rattus_norvegicus_OX_10116_GN_Gch1_PE_1_SV_
MetazoaVertebrateEP00067Daniorerio_5726445_GCH1_CHICK_GTP_cyclohydrolase_1_OS_Gallus_gallus_OX_9031_GN_GCH1_PE_2_SV_
MetazoaVertebrateAnoliscarolinensis_6309659_GCH1_HUMAN_GTP_cyclohydrolase_1_OS_Homo_sapiens_OX_9606_GN_GCH1_PE_1_SV_
MetazoaVertebrateAnoliscarolinensis_6334867_GCH1_HUMAN_GTP_cyclohydrolase_1_OS_Homo_sapiens_OX_9606_GN_GCH1_PE_1_SV_
MetazoaVertebrateLatimeriachalumnae_5781956_GCH1_HUMAN_GTP_cyclohydrolase_1_OS_Homo_sapiens_OX_9606_GN_GCH1_PE_1_SV_
MetazoaVertebrateCallorhinchusmilii_5514408_GCH1_RAT_GTP_cyclohydrolase_1_OS_Rattus_norvegicus_OX_10116_GN_Gch1_PE_1_SV_
MetazoaVertebratePetromyzonmarinus_6580987_GCH1_MOUSE_GTP_cyclohydrolase_1_OS_Mus_musculus_OX_10090_GN_Gch1_PE_1_SV_
MetazoaVertebrateHomosapiens_6455774_GCH1_HUMAN_GTP_cyclohydrolase_1_OS_Homo_sapiens_OX_9606_GN_GCH1_PE_1_SV_
MetazoaVertebrateMusmusculus_6534988_GCH1_MOUSE_GTP_cyclohydrolase_1_OS_Mus_musculus_OX_10090_GN_Gch1_PE_1_SV_
MetazoaVertebrateMonodelphisdomestica_6491956_GCH1_HUMAN_GTP_cyclohydrolase_1_OS_Homo_sapiens_OX_9606_GN_GCH1_PE_1_SV_
MetazoaVertebrateGallusgallus_6422360_GCH1_CHICK_GTP_cyclohydrolase_1_OS_Gallus_gallus_OX_9031_GN_GCH1_PE_2_SV_
MetazoaVertebrateGallusgallus_6425842_GCH1_CHICK_GTP_cyclohydrolase_1_OS_Gallus_gallus_OX_9031_GN_GCH1_PE_2_SV_
MetazoaVertebrateEP00079Xenopustropicalis_6343505_GCH1_HUMAN_GTP_cyclohydrolase_1_OS_Homo_sapiens_OX_9606_GN_GCH1_PE_1_SV_
MetazoaVertebrateEP00066Takifugurubripes_5689315_GCH1_RAT_GTP_cyclohydrolase_1_OS_Rattus_norvegicus_OX_10116_GN_Gch1_PE_1_SV_
MetazoaVertebrateEP00067Daniorerio_5723051_GCH1_RAT_GTP_cyclohydrolase_1_OS_Rattus_norvegicus_OX_10116_GN_Gch1_PE_1_SV_
MetazoaVertebrateCallorhinchusmilii_5506625_GCH1_HUMAN_GTP_cyclohydrolase_1_OS_Homo_sapiens_OX_9606_GN_GCH1_PE_1_SV_
MetazoaVertebrateLatimeriachalumnae_5767918_GCH1_HUMAN_GTP_cyclohydrolase_1_OS_Homo_sapiens_OX_9606_GN_GCH1_PE_1_SV_
MetazoaRotiferaAdinetavaga_5200000_GCH1_RAT_GTP_cyclohydrolase_1_OS_Rattus_norvegicus_OX_10116_GN_Gch1_PE_1_SV_
MetazoaRotiferaAdinetavaga_5220164_GCH1_CHICK_GTP_cyclohydrolase_1_OS_Gallus_gallus_OX_9031_GN_GCH1_PE_2_SV_
MetazoaRotiferaAdinetavaga_5209087_GCH1_HUMAN_GTP_cyclohydrolase_1_OS_Homo_sapiens_OX_9606_GN_GCH1_PE_1_SV_
MetazoaRotiferaAdinetavaga_5213034_GCH1_HUMAN_GTP_cyclohydrolase_1_OS_Homo_sapiens_OX_9606_GN_GCH1_PE_1_SV_
MetazoaRotiferaAdinetavaga_5216003_GCH1_HUMAN_GTP_cyclohydrolase_1_OS_Homo_sapiens_OX_9606_GN_GCH1_PE_1_SV_
MetazoaRotiferaAdinetavaga_5221173_GCH1_HUMAN_GTP_cyclohydrolase_1_OS_Homo_sapiens_OX_9606_GN_GCH1_PE_1_SV_
MetazoaAnnelidaEP00103Capitellateleta_60700_GCH1_HUMAN_GTP_cyclohydrolase_1_OS_Homo_sapiens_OX_9606_GN_GCH1_PE_1_SV_
MetazoaAnnelidaHelobdellarobusta_101699_GCH1_MOUSE_GTP_cyclohydrolase_1_OS_Mus_musculus_OX_10090_GN_Gch1_PE_1_SV_
MetazoaBrachiopodaLingulaunguis_1294299_GCH1_MOUSE_GTP_cyclohydrolase_1_OS_Mus_musculus_OX_10090_GN_Gch1_PE_1_SV_
MetazoaMolluscaEP00107Lottiagigantea_4188831_GCH1_RAT_GTP_cyclohydrolase_1_OS_Rattus_norvegicus_OX_10116_GN_Gch1_PE_1_SV_
MetazoaEchStrongylocentrotuspurpuratus_3833644_GCH1_CHICK_GTP_cyclohydrolase_1_OS_Gallus_gallus_OX_9031_GN_GCH1_PE_2_SV_
MetazoaEchStrongylocentrotuspurpuratus_3834699_GCH1_RAT_GTP_cyclohydrolase_1_OS_Rattus_norvegicus_OX_10116_GN_Gch1_PE_1_SV_
MetazoaEchinodermataAcanthasterplanci_3785365_GCH1_MOUSE_GTP_cyclohydrolase_1_OS_Mus_musculus_OX_10090_GN_Gch1_PE_1_SV_
MetazoaArthropodaDaphniapulex_194927_GCH1_HUMAN_GTP_cyclohydrolase_1_OS_Homo_sapiens_OX_9606_GN_GCH1_PE_1_SV_
MetazoaArthropodaDrosophilamelanogaster_243690_GCH1_DROME_GTP_cyclohydrolase_1_OS_Drosophila_melanogaster_OX_7227_GN_Pu_PE_1_SV_
MetazoaArthropodaStegodyphusmimosarum_1145170_GCH1_RAT_GTP_cyclohydrolase_1_OS_Rattus_norvegicus_OX_10116_GN_Gch1_PE_1_SV_
MetazoaMolluscaEP00105Crassostreagigas_4141930_GCH1_RAT_GTP_cyclohydrolase_1_OS_Rattus_norvegicus_OX_10116_GN_Gch1_PE_1_SV_
MetazoaCephaBranchiostomabelcheri_2804178_GCH1_HUMAN_GTP_cyclohydrolase_1_OS_Homo_sapiens_OX_9606_GN_GCH1_PE_1_SV_
MetazoaCtenophoraMnemiopsisleidyi_3728903_GCH1_OSTOS_GTP_cyclohydrolase_1_OS_Ostertagia_ostertagi_OX_6317_GN_gch_PE_2_SV_
MetazoaPoriferaAmphimedonqueenslandica_4839923_GCH1_MOUSE_GTP_cyclohydrolase_1_OS_Mus_musculus_OX_10090_GN_Gch1_PE_1_SV_
MetazoaAcoelaHofsteniamiamia_20954_GCH1_MOUSE_GTP_cyclohydrolase_1_OS_Mus_musculus_OX_10090_GN_Gch1_PE_1_SV_
MetazoaPoriferaEP00118Oscarellapearsei_4811706_GCH1_OSTOS_GTP_cyclohydrolase_1_OS_Ostertagia_ostertagi_OX_6317_GN_gch_PE_2_SV_
MetazoaPoriferaEP00118Oscarellapearsei_4811707_GCH1_HUMAN_GTP_cyclohydrolase_1_OS_Homo_sapiens_OX_9606_GN_GCH1_PE_1_SV_
MetazoaPlacozoaHoilungiahongkongensis_4551388_GCH1_HUMAN_GTP_cyclohydrolase_1_OS_Homo_sapiens_OX_9606_GN_GCH1_PE_1_SV_
MetazoaPlacozoaHoilungiahongkongensis_4551389_GCH1_CAEEL_GTP_cyclohydrolase_1_OS_Caenorhabditis_elegans_OX_6239_GN_cat_4_PE_1_SV_
MetazoaPlacozoaEP00114TrichoplaxspH2_4511231_GCH1_MOUSE_GTP_cyclohydrolase_1_OS_Mus_musculus_OX_10090_GN_Gch1_PE_1_SV_
MetazoaPoriferaEP00117Syconciliatum_4806386_GCH1_HUMAN_GTP_cyclohydrolase_1_OS_Homo_sapiens_OX_9606_GN_GCH1_PE_1_SV_
MetazoaCnidariaFungiascutaria_3197612_GCH1_RAT_GTP_cyclohydrolase_1_OS_Rattus_norvegicus_OX_10116_GN_Gch1_PE_1_SV_
MetazoaCnidariaStylophorapistillata_3677013_GCH1_RAT_GTP_cyclohydrolase_1_OS_Rattus_norvegicus_OX_10116_GN_Gch1_PE_1_SV_
MetazoaCnidariaNematostellavectensis_007141GCH1_OSTOS_GTP_cyclohydrolase_1_OS_Ostertagia_ostertagi_OX_6317_GN_gch_PE_2_SV_
MetazoaCnidariaNematostellavectensis_3376549_GCH1_OSTOS_GTP_cyclohydrolase_1_OS_Ostertagia_ostertagi_OX_6317_GN_gch_PE_2_SV_
MetazoaUrochordataCionaintestinalis_5336309_GCH1_CAEEL_GTP_cyclohydrolase_1_OS_Caenorhabditis_elegans_OX_6239_GN_cat_4_PE_1_SV_
MetazoaUrochordataCionaintestinalis_5351905_GCH1_CAEEL_GTP_cyclohydrolase_1_OS_Caenorhabditis_elegans_OX_6239_GN_cat_4_PE_1_SV_
MetazoaNematodaCaenorhabditiselegans_4400269_GCH1_CAEEL_GTP_cyclohydrolase_1_OS_Caenorhabditis_elegans_OX_6239_GN_cat_4_PE_1_SV_
MetazoaCnidariaAureliaGenome_3062404_GCH1_RAT_GTP_cyclohydrolase_1_OS_Rattus_norvegicus_OX_10116_GN_Gch1_PE_1_SV_
MetazoaCnidariaCyaneanozakii_5641_GCH1_OSTOS_GTP_cyclohydrolase_1_OS_Ostertagia_ostertagi_OX_6317_GN_gch_PE_2_SV_
MetazoaCnidariaLucernariaquadricornis_63454_GCH1_RAT_GTP_cyclohydrolase_1_OS_Rattus_norvegicus_OX_10116_GN_Gch1_PE_1_SV_
MetazoaCnidariaClytiahemisphaerica_3077202_GCH1_OSTOS_GTP_cyclohydrolase_1_OS_Ostertagia_ostertagi_OX_6317_GN_gch_PE_2_SV_
MetazoaCnidariaHydramagnapapillata_3262837_GCH1_HUMAN_GTP_cyclohydrolase_1_OS_Homo_sapiens_OX_9606_GN_GCH1_PE_1_SV_
MetazoaCnidariaTripedaliacystophora_168768_GCH1_CAEEL_GTP_cyclohydrolase_1_OS_Caenorhabditis_elegans_OX_6239_GN_cat_4_PE_1_SV_
MetazoaCnidariaHelioporacoerulea_319094_GCH1_HUMAN_GTP_cyclohydrolase_1_OS_Homo_sapiens_OX_9606_GN_GCH1_PE_1_SV_
MetazoaCnidariaParamuriceabiscaya_424543_GCH1_RAT_GTP_cyclohydrolase_1_OS_Rattus_norvegicus_OX_10116_GN_Gch1_PE_1_SV_
MetazoaCnidariaRenillakoellikeri_519078_GCH1_MOUSE_GTP_cyclohydrolase_1_OS_Mus_musculus_OX_10090_GN_Gch1_PE_1_SV_
MetazoaCnidariaParamuriceabiscaya_391290_GCH1_OSTOS_GTP_cyclohydrolase_1_OS_Ostertagia_ostertagi_OX_6317_GN_gch_PE_2_SV_
FungiFusariumoxysporumUP000002489_13357941_GCH1_NEUCR_GTP_cyclohydrolase_1_OS_Neurospora_crassa__strain_ATCC_24698_
FungiFusariumoxysporumUP000002489_13366296_GCH1_NEUCR_GTP_cyclohydrolase_1_OS_Neurospora_crassa__strain_ATCC_24698_
FungiAspergillusnigerUP000068243_13140958_GCH1_NEUCR_GTP_cyclohydrolase_1_OS_Neurospora_crassa__strain_ATCC_24698_
FungiMortierellaelongataUP000078512_13383706_GCH1_SYNY3_GTP_cyclohydrolase_1_OS_Synechocystis_sp___strain_PCC_6803_
FungiMortierellaelongataUP000078512_13385031_GCH1_NEUCR_GTP_cyclohydrolase_1_OS_Neurospora_crassa__strain_ATCC_24698_
FungiMortierellaelongataUP000078512_13385612_GCH1_NEUCR_GTP_cyclohydrolase_1_OS_Neurospora_crassa__strain_ATCC_24698_
FungiRhizopusmicrosporusUP000242254_13428954_GCH1_SCHPO_GTP_cyclohydrolase_1_OS_Schizosaccharomyces_pombe__strain_972_
FungiAgaricusbisporusUP000008493_13107514_GCH1_NEUCR_GTP_cyclohydrolase_1_OS_Neurospora_crassa__strain_ATCC_24698_
FungiEP00150Ustilagomaydis_13257280_GCH1_SCHPO_GTP_cyclohydrolase_1_OS_Schizosaccharomyces_pombe__strain_972_
RotosphaeridaEP00160Parvulariaatlantis_23172479_GCH1_SCHPO_GTP_cyclohydrolase_1_OS_Schizosaccharomyces_pombe__strain_972_
FungiSpizellomycespunctatusUP000053201_13458684_GCH1_SCHPO_GTP_cyclohydrolase_1_OS_Schizosaccharomyces_pombe__strain_972_
RotosphaeridaFonticulaalbaUP000030693_23193331_GCH1_YEAST_GTP_cyclohydrolase_1_OS_Saccharomyces_cerevisiae__strain_ATCC_204508_
FilastreaCapsasporaowczarzakiUP000008743_12983158_GCH1_HUMAN_GTP_cyclohydrolase_1_OS_Homo_sapiens_OX_9606_GN_GCH1_PE_1_SV_
PluriformeaEP00128Syssomonasmultiformis_21627332_GCH1_MOUSE_GTP_cyclohydrolase_1_OS_Mus_musculus_OX_10090_GN_Gch1_PE_1_SV_
PluriformeaCorallochytriumlimacisporum_21622023_GCH1_RAT_GTP_cyclohydrolase_1_OS_Rattus_norvegicus_OX_10116_GN_Gch1_PE_1_SV_
IchthyosporeaAmoebidiumparasiticum_14568508_GCH1_SCHPO_GTP_cyclohydrolase_1_OS_Schizosaccharomyces_pombe__strain_972_
IchthyosporeaSphaerothecumdestruens_14599277_GCH1_NEUCR_GTP_cyclohydrolase_1_OS_Neurospora_crassa__strain_ATCC_24698_
MetazoaCnidariaHelioporacoerulea_246359_GCH1_SYNY3_GTP_cyclohydrolase_1_OS_Synechocystis_sp___strain_PCC_6803_
MetazoaCnidariaHelioporacoerulea_294768_GCH1_AGRVS_GTP_cyclohydrolase_1_OS_Agrobacterium_vitis__strain_S4_
ChoanoflagellataEP00039Didymoecacostata_10857927_GCH1_AGRFC_GTP_cyclohydrolase_1_OS_Agrobacterium_fabrum__strain_C58_
MetazoaCnidariaStylophorapistillata_3664939_SYH_GRAFK_Histidine__tRNA_ligase_OS_Gramella_forsetii__strain_KT0803__OX_411154_GN_hisS_PE_3_SV_
MetazoaCnidariaPolypodiumhydrifrome_90549_GCH1_OSTOS_GTP_cyclohydrolase_1_OS_Ostertagia_ostertagi_OX_6317_GN_gch_PE_2_SV_
MetazoaCnidariaPolypodiumhydrifrome_90554_GCH1_SYNY3_GTP_cyclohydrolase_1_OS_Synechocystis_sp___strain_PCC_6803_
ChoanoflagellataMylnosigafluctuans_11126188_GCH1_NEUCR_GTP_cyclohydrolase_1_OS_Neurospora_crassa__strain_ATCC_24698_
ChoanoflagellataSalpingoecakvevrii_11197061_GCH1_RAT_GTP_cyclohydrolase_1_OS_Rattus_norvegicus_OX_10116_GN_Gch1_PE_1_SV_
ChoanoflagellataEP00039Didymoecacostata_10871464_GCH1_NEUCR_GTP_cyclohydrolase_1_OS_Neurospora_crassa__strain_ATCC_24698_
ChoanoflagellataEP00039Didymoecacostata_10872331_GCH1_CHICK_GTP_cyclohydrolase_1_OS_Gallus_gallus_OX_9031_GN_GCH1_PE_2_SV_
ChoanoflagellataEP00037Helgoecanana_10798093_GCH1_OSTOS_GTP_cyclohydrolase_1_OS_Ostertagia_ostertagi_OX_6317_GN_gch_PE_2_SV_
0.62

3C.

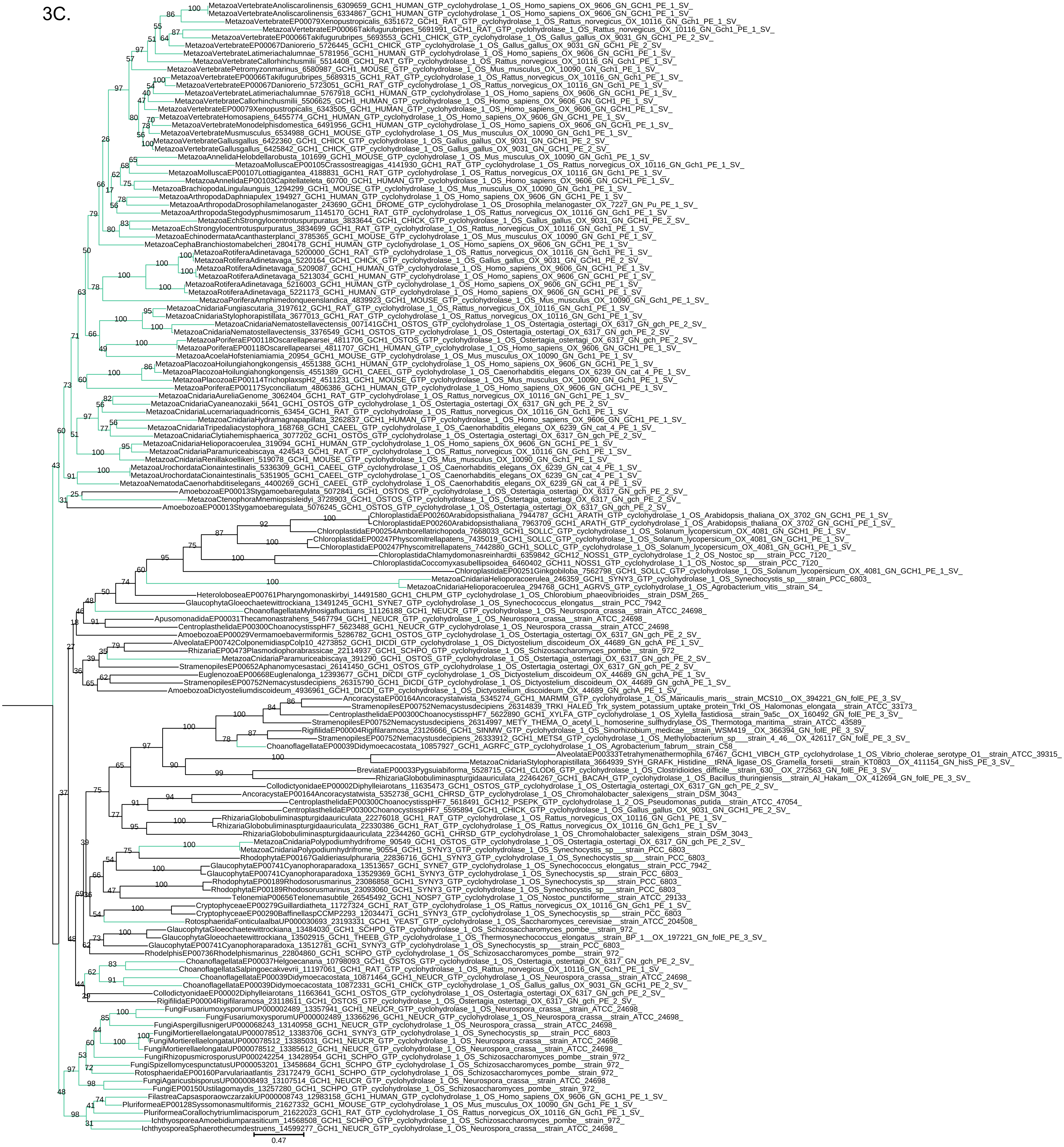

MetazoaVertebrateAnoliscarolinensis_6309659_GCH1_HUMAN_GTP_cyclohydrolase_1_OS_Homo_sapiens_OX_9606_GN_GCH1_PE_1_SV_
MetazoaVertebrateAnoliscarolinensis_6334867_GCH1_HUMAN_GTP_cyclohydrolase_1_OS_Homo_sapiens_OX_9606_GN_GCH1_PE_1_SV_
MetazoaVertebrateEP00079Xenopustropicalis_6351672_GCH1_RAT_GTP_cyclohydrolase_1_OS_Rattus_norvegicus_OX_10116_GN_Gch1_PE_1_SV_
MetazoaVertebrateEP00066Takifugurubripes_5691991_GCH1_RAT_GTP_cyclohydrolase_1_OS_Rattus_norvegicus_OX_10116_GN_Gch1_PE_1_SV_
MetazoaVertebrateEP00066Takifugurubripes_5693553_GCH1_CHICK_GTP_cyclohydrolase_1_OS_Gallus_gallus_OX_9031_GN_GCH1_PE_2_SV_
MetazoaVertebrateEP00067Daniorerio_5726445_GCH1_CHICK_GTP_cyclohydrolase_1_OS_Gallus_gallus_OX_9031_GN_GCH1_PE_2_SV_
MetazoaVertebrateLatimeriachalumnae_5781956_GCH1_HUMAN_GTP_cyclohydrolase_1_OS_Homo_sapiens_OX_9606_GN_GCH1_PE_1_SV_
MetazoaVertebrateCallorhinchusmilii_5514408_GCH1_RAT_GTP_cyclohydrolase_1_OS_Rattus_norvegicus_OX_10116_GN_Gch1_PE_1_SV_
MetazoaVertebratePetromyzonmarinus_6580987_GCH1_MOUSE_GTP_cyclohydrolase_1_OS_Mus_musculus_OX_10090_GN_Gch1_PE_1_SV_
MetazoaVertebrateEP00066Takifugurubripes_5689315_GCH1_RAT_GTP_cyclohydrolase_1_OS_Rattus_norvegicus_OX_10116_GN_Gch1_PE_1_SV_
MetazoaVertebrateEP00067Daniorerio_5723051_GCH1_RAT_GTP_cyclohydrolase_1_OS_Rattus_norvegicus_OX_10116_GN_Gch1_PE_1_SV_
MetazoaVertebrateLatimeriachalumnae_5767918_GCH1_HUMAN_GTP_cyclohydrolase_1_OS_Homo_sapiens_OX_9606_GN_GCH1_PE_1_SV_
MetazoaVertebrateCallorhinchusmilii_5506625_GCH1_HUMAN_GTP_cyclohydrolase_1_OS_Homo_sapiens_OX_9606_GN_GCH1_PE_1_SV_
MetazoaVertebrateEP00079Xenopustropicalis_6343505_GCH1_HUMAN_GTP_cyclohydrolase_1_OS_Homo_sapiens_OX_9606_GN_GCH1_PE_1_SV_
MetazoaVertebrateHomosapiens_6455774_GCH1_HUMAN_GTP_cyclohydrolase_1_OS_Homo_sapiens_OX_9606_GN_GCH1_PE_1_SV_
MetazoaVertebrateMonodelphisdomestica_6491956_GCH1_HUMAN_GTP_cyclohydrolase_1_OS_Homo_sapiens_OX_9606_GN_GCH1_PE_1_SV_
MetazoaVertebrateMusmusculus_6534988_GCH1_MOUSE_GTP_cyclohydrolase_1_OS_Mus_musculus_OX_10090_GN_Gch1_PE_1_SV_
MetazoaVertebrateGallusgallus_6422360_GCH1_CHICK_GTP_cyclohydrolase_1_OS_Gallus_gallus_OX_9031_GN_GCH1_PE_2_SV_
MetazoaVertebrateGallusgallus_6425842_GCH1_CHICK_GTP_cyclohydrolase_1_OS_Gallus_gallus_OX_9031_GN_GCH1_PE_2_SV_
MetazoaAnnelidaHelobdellarobusta_101699_GCH1_MOUSE_GTP_cyclohydrolase_1_OS_Mus_musculus_OX_10090_GN_Gch1_PE_1_SV_
MetazoaMolluscaEP00105Crassostreagigas_4141930_GCH1_RAT_GTP_cyclohydrolase_1_OS_Rattus_norvegicus_OX_10116_GN_Gch1_PE_1_SV_
MetazoaMolluscaEP00107Lottiagigantea_4188831_GCH1_RAT_GTP_cyclohydrolase_1_OS_Rattus_norvegicus_OX_10116_GN_Gch1_PE_1_SV_
MetazoaAnnelidaEP00103Capitellateleta_60700_GCH1_HUMAN_GTP_cyclohydrolase_1_OS_Homo_sapiens_OX_9606_GN_GCH1_PE_1_SV_
MetazoaBrachiopodaLingulaunguis_1294299_GCH1_MOUSE_GTP_cyclohydrolase_1_OS_Mus_musculus_OX_10090_GN_Gch1_PE_1_SV_
MetazoaArthropodaDaphniapulex_194927_GCH1_HUMAN_GTP_cyclohydrolase_1_OS_Homo_sapiens_OX_9606_GN_GCH1_PE_1_SV_
MetazoaArthropodaDrosophilamelanogaster_243690_GCH1_DROME_GTP_cyclohydrolase_1_OS_Drosophila_melanogaster_OX_7227_GN_Pu_PE_1_SV_
MetazoaArthropodaStegodyphusmimosarum_1145170_GCH1_RAT_GTP_cyclohydrolase_1_OS_Rattus_norvegicus_OX_10116_GN_Gch1_PE_1_SV_
MetazoaEchStrongylocentrotuspurpuratus_3833644_GCH1_CHICK_GTP_cyclohydrolase_1_OS_Gallus_gallus_OX_9031_GN_GCH1_PE_2_SV_
MetazoaEchStrongylocentrotuspurpuratus_3834699_GCH1_RAT_GTP_cyclohydrolase_1_OS_Rattus_norvegicus_OX_10116_GN_Gch1_PE_1_SV_
MetazoaEchinodermataAcanthasterplanci_3785365_GCH1_MOUSE_GTP_cyclohydrolase_1_OS_Mus_musculus_OX_10090_GN_Gch1_PE_1_SV_
MetazoaCephaBranchiostomabelcheri_2804178_GCH1_HUMAN_GTP_cyclohydrolase_1_OS_Homo_sapiens_OX_9606_GN_GCH1_PE_1_SV_
MetazoaRotiferaAdinetavaga_5200000_GCH1_RAT_GTP_cyclohydrolase_1_OS_Rattus_norvegicus_OX_10116_GN_Gch1_PE_1_SV_
MetazoaRotiferaAdinetavaga_5220164_GCH1_CHICK_GTP_cyclohydrolase_1_OS_Gallus_gallus_OX_9031_GN_GCH1_PE_2_SV_
MetazoaRotiferaAdinetavaga_5209087_GCH1_HUMAN_GTP_cyclohydrolase_1_OS_Homo_sapiens_OX_9606_GN_GCH1_PE_1_SV_
MetazoaRotiferaAdinetavaga_5213034_GCH1_HUMAN_GTP_cyclohydrolase_1_OS_Homo_sapiens_OX_9606_GN_GCH1_PE_1_SV_
MetazoaRotiferaAdinetavaga_5216003_GCH1_HUMAN_GTP_cyclohydrolase_1_OS_Homo_sapiens_OX_9606_GN_GCH1_PE_1_SV_
MetazoaRotiferaAdinetavaga_5221173_GCH1_HUMAN_GTP_cyclohydrolase_1_OS_Homo_sapiens_OX_9606_GN_GCH1_PE_1_SV_
MetazoaPoriferaAmphimedonqueenslandica_4839923_GCH1_MOUSE_GTP_cyclohydrolase_1_OS_Mus_musculus_OX_10090_GN_Gch1_PE_1_SV_
MetazoaCnidariaFungiascutaria_3197612_GCH1_RAT_GTP_cyclohydrolase_1_OS_Rattus_norvegicus_OX_10116_GN_Gch1_PE_1_SV_
MetazoaCnidariaStylophorapistillata_3677013_GCH1_RAT_GTP_cyclohydrolase_1_OS_Rattus_norvegicus_OX_10116_GN_Gch1_PE_1_SV_
MetazoaCnidariaNematostellavectensis_007141GCH1_OSTOS_GTP_cyclohydrolase_1_OS_Ostertagia_ostertagi_OX_6317_GN_gch_PE_2_SV_
MetazoaCnidariaNematostellavectensis_3376549_GCH1_OSTOS_GTP_cyclohydrolase_1_OS_Ostertagia_ostertagi_OX_6317_GN_gch_PE_2_SV_
MetazoaPoriferaEP00118Oscarellapearsei_4811706_GCH1_OSTOS_GTP_cyclohydrolase_1_OS_Ostertagia_ostertagi_OX_6317_GN_gch_PE_2_SV_
MetazoaPoriferaEP00118Oscarellapearsei_4811707_GCH1_HUMAN_GTP_cyclohydrolase_1_OS_Homo_sapiens_OX_9606_GN_GCH1_PE_1_SV_
MetazoaAcoelaHofsteniamiamia_20954_GCH1_MOUSE_GTP_cyclohydrolase_1_OS_Mus_musculus_OX_10090_GN_Gch1_PE_1_SV_
MetazoaPlacozoaHoilungiahongkongensis_4551388_GCH1_HUMAN_GTP_cyclohydrolase_1_OS_Homo_sapiens_OX_9606_GN_GCH1_PE_1_SV_
MetazoaPlacozoaHoilungiahongkongensis_4551389_GCH1_CAEEL_GTP_cyclohydrolase_1_OS_Caenorhabditis_elegans_OX_6239_GN_cat_4_PE_1_SV_
MetazoaPlacozoaEP00114TrichoplaxspH2_4511231_GCH1_MOUSE_GTP_cyclohydrolase_1_OS_Mus_musculus_OX_10090_GN_Gch1_PE_1_SV_
MetazoaPoriferaEP00117Syconciliatum_4806386_GCH1_HUMAN_GTP_cyclohydrolase_1_OS_Homo_sapiens_OX_9606_GN_GCH1_PE_1_SV_
MetazoaCnidariaAureliaGenome_3062404_GCH1_RAT_GTP_cyclohydrolase_1_OS_Rattus_norvegicus_OX_10116_GN_Gch1_PE_1_SV_
MetazoaCnidariaCyaneanozakii_5641_GCH1_OSTOS_GTP_cyclohydrolase_1_OS_Ostertagia_ostertagi_OX_6317_GN_gch_PE_2_SV_
MetazoaCnidariaLucernariaquadricornis_63454_GCH1_RAT_GTP_cyclohydrolase_1_OS_Rattus_norvegicus_OX_10116_GN_Gch1_PE_1_SV_
MetazoaCnidariaHydramagnapapillata_3262837_GCH1_HUMAN_GTP_cyclohydrolase_1_OS_Homo_sapiens_OX_9606_GN_GCH1_PE_1_SV_
MetazoaCnidariaTripedaliacystophora_168768_GCH1_CAEEL_GTP_cyclohydrolase_1_OS_Caenorhabditis_elegans_OX_6239_GN_cat_4_PE_1_SV_
MetazoaCnidariaClytiahemisphaerica_3077202_GCH1_OSTOS_GTP_cyclohydrolase_1_OS_Ostertagia_ostertagi_OX_6317_GN_gch_PE_2_SV_
MetazoaCnidariaHelioporacoerulea_319094_GCH1_HUMAN_GTP_cyclohydrolase_1_OS_Homo_sapiens_OX_9606_GN_GCH1_PE_1_SV_
MetazoaCnidariaParamuriceabiscaya_424543_GCH1_RAT_GTP_cyclohydrolase_1_OS_Rattus_norvegicus_OX_10116_GN_Gch1_PE_1_SV_
MetazoaCnidariaRenillakoellikeri_519078_GCH1_MOUSE_GTP_cyclohydrolase_1_OS_Mus_musculus_OX_10090_GN_Gch1_PE_1_SV_
MetazoaUrochordataCionaintestinalis_5336309_GCH1_CAEEL_GTP_cyclohydrolase_1_OS_Caenorhabditis_elegans_OX_6239_GN_cat_4_PE_1_SV_
MetazoaUrochordataCionaintestinalis_5351905_GCH1_CAEEL_GTP_cyclohydrolase_1_OS_Caenorhabditis_elegans_OX_6239_GN_cat_4_PE_1_SV_
MetazoaNematodaCaenorhabditiselegans_4400269_GCH1_CAEEL_GTP_cyclohydrolase_1_OS_Caenorhabditis_elegans_OX_6239_GN_cat_4_PE_1_SV_
AmoebozoaEP00013Stygamoebaregulata_5072841_GCH1_OSTOS_GTP_cyclohydrolase_1_OS_Ostertagia_ostertagi_OX_6317_GN_gch_PE_2_SV_
MetazoaCtenophoraMnemiopsisleidyi_3728903_GCH1_OSTOS_GTP_cyclohydrolase_1_OS_Ostertagia_ostertagi_OX_6317_GN_gch_PE_2_SV_
AmoebozoaEP00013Stygamoebaregulata_5076245_GCH1_OSTOS_GTP_cyclohydrolase_1_OS_Ostertagia_ostertagi_OX_6317_GN_gch_PE_2_SV_
ChloroplastidaEP00260Arabidopsisthaliana_7944787_GCH1_ARATH_GTP_cyclohydrolase_1_OS_Arabidopsis_thaliana_OX_3702_GN_GCH1_PE_1_SV_
ChloroplastidaEP00260Arabidopsisthaliana_7963709_GCH1_ARATH_GTP_cyclohydrolase_1_OS_Arabidopsis_thaliana_OX_3702_GN_GCH1_PE_1_SV_
ChloroplastidaEP00254Amborellatrichopoda_7668033_GCH1_SOLLC_GTP_cyclohydrolase_1_OS_Solanum_lycopersicum_OX_4081_GN_GCH1_PE_1_SV_
ChloroplastidaEP00247Physcomitrellapatens_7435019_GCH1_SOLLC_GTP_cyclohydrolase_1_OS_Solanum_lycopersicum_OX_4081_GN_GCH1_PE_1_SV_
ChloroplastidaEP00247Physcomitrellapatens_7442880_GCH1_SOLLC_GTP_cyclohydrolase_1_OS_Solanum_lycopersicum_OX_4081_GN_GCH1_PE_1_SV_
ChloroplastidaChlamydomonasreinhardtii_6359842_GCH12_NOSS1_GTP_cyclohydrolase_1_2_OS_Nostoc_sp___strain_PCC_7120_
ChloroplastidaCoccomyxasubellipsoidea_6460402_GCH11_NOSS1_GTP_cyclohydrolase_1_1_OS_Nostoc_sp___strain_PCC_7120_
ChloroplastidaEP00251Ginkgobiloba_7562798_GCH1_SOLLC_GTP_cyclohydrolase_1_OS_Solanum_lycopersicum_OX_4081_GN_GCH1_PE_1_SV_
MetazoaCnidariaHelioporacoerulea_246359_GCH1_SYNY3_GTP_cyclohydrolase_1_OS_Synechocystis_sp___strain_PCC_6803_
MetazoaCnidariaHelioporacoerulea_294768_GCH1_AGRVS_GTP_cyclohydrolase_1_OS_Agrobacterium_vitis__strain_S4_
HeteroloboseaEP00761Pharyngomonaskirbyi_14491580_GCH1_CHLPM_GTP_cyclohydrolase_1_OS_Chlorobium_phaeovibrioides__strain_DSM_265_
GlaucophytaGloeochaetewittrockiana_13491245_GCH1_SYNE7_GTP_cyclohydrolase_1_OS_Synechococcus_elongatus__strain_PCC_7942_
ChoanoflagellataMylnosigafluctuans_11126188_GCH1_NEUCR_GTP_cyclohydrolase_1_OS_Neurospora_crassa__strain_ATCC_24698_
ApusomonadidaEP00031Thecamonastrahens_5467794_GCH1_NEUCR_GTP_cyclohydrolase_1_OS_Neurospora_crassa__strain_ATCC_24698_
CentroplasthelidaEP00300ChoanocystisspHF7_5623488_GCH1_NEUCR_GTP_cyclohydrolase_1_OS_Neurospora_crassa__strain_ATCC_24698_
AmoebozoaEP00029Vermamoebavermiformis_5286782_GCH1_OSTOS_GTP_cyclohydrolase_1_OS_Ostertagia_ostertagi_OX_6317_GN_gch_PE_2_SV_
AlveolataEP00742ColponemidiaspColp10_4273852_GCH1_DICDI_GTP_cyclohydrolase_1_OS_Dictyostelium_discoideum_OX_44689_GN_gchA_PE_1_SV_
RhizariaEP00473Plasmodiophorabrassicae_22114937_GCH1_SCHPO_GTP_cyclohydrolase_1_OS_Schizosaccharomyces_pombe__strain_972_
MetazoaCnidariaParamuriceabiscaya_391290_GCH1_OSTOS_GTP_cyclohydrolase_1_OS_Ostertagia_ostertagi_OX_6317_GN_gch_PE_2_SV_
StramenopilesEP00652Aphanomycesastaci_26141450_GCH1_OSTOS_GTP_cyclohydrolase_1_OS_Ostertagia_ostertagi_OX_6317_GN_gch_PE_2_SV_
EuglenozoaEP00668Euglenalonga_12393677_GCH1_DICDI_GTP_cyclohydrolase_1_OS_Dictyostelium_discoideum_OX_44689_GN_gchA_PE_1_SV_
StramenopilesEP00752Nemacystusdecipiens_26315790_GCH1_DICDI_GTP_cyclohydrolase_1_OS_Dictyostelium_discoideum_OX_44689_GN_gchA_PE_1_SV_
AmoebozoaDictyosteliumdiscoideum_4936961_GCH1_DICDI_GTP_cyclohydrolase_1_OS_Dictyostelium_discoideum_OX_44689_GN_gchA_PE_1_SV_
AncoracystaEP00164Ancoracystatwista_5345274_GCH1_MARMM_GTP_cyclohydrolase_1_OS_Maricaulis_maris__strain_MCS10__OX_394221_GN_folE_PE_3_SV_
StramenopilesEP00752Nemacystusdecipiens_26314839_TRKI_HALED_Trk_system_potassium_uptake_protein_TrkI_OS_Halomonas_elongata__strain_ATCC_33173_
CentroplasthelidaEP00300ChoanocystisspHF7_5622890_GCH1_XYLFA_GTP_cyclohydrolase_1_OS_Xylella_fastidiosa__strain_9a5c__OX_160492_GN_folE_PE_3_SV_
StramenopilesEP00752Nemacystusdecipiens_26314997_METY_THEMA_O_acetyl_L_homoserine_sulfhydrylase_OS_Thermotoga_maritima__strain_ATCC_43589_
RigifilidaEP00004Rigifilaramosa_23126666_GCH1_SINMW_GTP_cyclohydrolase_1_OS_Sinorhizobium_medicae__strain_WSM419__OX_366394_GN_folE_PE_3_SV_
StramenopilesEP00752Nemacystusdecipiens_26333912_GCH1_METS4_GTP_cyclohydrolase_1_OS_Methylobacterium_sp___strain_4_46__OX_426117_GN_folE_PE_3_SV_
ChoanoflagellataEP00039Didymoecacostata_10857927_GCH1_AGRFC_GTP_cyclohydrolase_1_OS_Agrobacterium_fabrum__strain_C58_
AlveolataEP00333Tetrahymenathermophila_67467_GCH1_VIBCH_GTP_cyclohydrolase_1_OS_Vibrio_cholerae_serotype_O1__strain_ATCC_39315_
MetazoaCnidariaStylophorapistillata_3664939_SYH_GRAFK_Histidine__tRNA_ligase_OS_Gramella_forsetii__strain_KT0803__OX_411154_GN_hisS_PE_3_SV_
BreviataEP00033Pygsuiabiforma_5528715_GCH1_CLOD6_GTP_cyclohydrolase_1_OS_Clostridioides_difficile__strain_630__OX_272563_GN_folE_PE_3_SV_
RhizariaGlobobuliminaspturgidaauriculata_22464267_GCH1_BACAH_GTP_cyclohydrolase_1_OS_Bacillus_thuringiensis__strain_Al_Hakam__OX_412694_GN_folE_PE_3_SV_
CollodictyonidaeEP00002Diphylleiarotans_11635473_GCH1_OSTOS_GTP_cyclohydrolase_1_OS_Ostertagia_ostertagi_OX_6317_GN_gch_PE_2_SV_
AncoracystaEP00164Ancoracystatwista_5352738_GCH1_CHRSD_GTP_cyclohydrolase_1_OS_Chromohalobacter_salexigens__strain_DSM_3043_
CentroplasthelidaEP00300ChoanocystisspHF7_5618491_GCH12_PSEPK_GTP_cyclohydrolase_1_2_OS_Pseudomonas_putida__strain_ATCC_47054_
CentroplasthelidaEP00300ChoanocystisspHF7_5595894_GCH1_CHICK_GTP_cyclohydrolase_1_OS_Gallus_gallus_OX_9031_GN_GCH1_PE_2_SV_
RhizariaGlobobuliminaspturgidaauriculata_22276018_GCH1_RAT_GTP_cyclohydrolase_1_OS_Rattus_norvegicus_OX_10116_GN_Gch1_PE_1_SV_
RhizariaGlobobuliminaspturgidaauriculata_22330386_GCH1_RAT_GTP_cyclohydrolase_1_OS_Rattus_norvegicus_OX_10116_GN_Gch1_PE_1_SV_
RhizariaGlobobuliminaspturgidaauriculata_22344260_GCH1_CHRSD_GTP_cyclohydrolase_1_OS_Chromohalobacter_salexigens__strain_DSM_3043_
MetazoaCnidariaPolypodiumhydrifrome_90549_GCH1_OSTOS_GTP_cyclohydrolase_1_OS_Ostertagia_ostertagi_OX_6317_GN_gch_PE_2_SV_
MetazoaCnidariaPolypodiumhydrifrome_90554_GCH1_SYNY3_GTP_cyclohydrolase_1_OS_Synechocystis_sp___strain_PCC_6803_
RhodophytaEP00167Galdieriasulphuraria_22836716_GCH1_SYNY3_GTP_cyclohydrolase_1_OS_Synechocystis_sp___strain_PCC_6803_
GlaucophytaEP00741Cyanophoraparadoxa_13513657_GCH1_SYNE7_GTP_cyclohydrolase_1_OS_Synechococcus_elongatus__strain_PCC_7942_
GlaucophytaEP00741Cyanophoraparadoxa_13529369_GCH1_SYNY3_GTP_cyclohydrolase_1_OS_Synechocystis_sp___strain_PCC_6803_
RhodophytaEP00189Rhodosorusmarinus_23086858_GCH1_SYNY3_GTP_cyclohydrolase_1_OS_Synechocystis_sp___strain_PCC_6803_
RhodophytaEP00189Rhodosorusmarinus_23093060_GCH1_SYNY3_GTP_cyclohydrolase_1_OS_Synechocystis_sp___strain_PCC_6803_
TelonemiaP00656Telonemasubtile_26545492_GCH1_NOSP7_GTP_cyclohydrolase_1_OS_Nostoc_punctiforme__strain_ATCC_29133_
CryptophyceaeEP00279Guillardiatheta_11727324_GCH1_RAT_GTP_cyclohydrolase_1_OS_Rattus_norvegicus_OX_10116_GN_Gch1_PE_1_SV_
CryptophyceaeEP00290BaffinellaspCCMP2293_12034471_GCH1_SYNY3_GTP_cyclohydrolase_1_OS_Synechocystis_sp___strain_PCC_6803_
RotosphaeridaFonticulaalbaUP000030693_23193331_GCH1_YEAST_GTP_cyclohydrolase_1_OS_Saccharomyces_cerevisiae__strain_ATCC_204508_
GlaucophytaGloeochaetewittrockiana_13484030_GCH1_SCHPO_GTP_cyclohydrolase_1_OS_Schizosaccharomyces_pombe__strain_972_
GlaucophytaGloeochaetewittrockiana_13502915_GCH1_THEEB_GTP_cyclohydrolase_1_OS_Thermosynechococcus_elongatus__strain_BP_1__OX_197221_GN_folE_PE_3_SV_
GlaucophytaEP00741Cyanophoraparadoxa_13512781_GCH1_SYNY3_GTP_cyclohydrolase_1_OS_Synechocystis_sp___strain_PCC_6803_
RhodelphisEP00736Rhodelphismarinus_22804860_GCH1_SCHPO_GTP_cyclohydrolase_1_OS_Schizosaccharomyces_pombe__strain_972_
ChoanoflagellataEP00037Helgoecanana_10798093_GCH1_OSTOS_GTP_cyclohydrolase_1_OS_Ostertagia_ostertagi_OX_6317_GN_gch_PE_2_SV_
ChoanoflagellataSalpingoecakvevrii_11197061_GCH1_RAT_GTP_cyclohydrolase_1_OS_Rattus_norvegicus_OX_10116_GN_Gch1_PE_1_SV_
ChoanoflagellataEP00039Didymoecacostata_10871464_GCH1_NEUCR_GTP_cyclohydrolase_1_OS_Neurospora_crassa__strain_ATCC_24698_
ChoanoflagellataEP00039Didymoecacostata_10872331_GCH1_CHICK_GTP_cyclohydrolase_1_OS_Gallus_gallus_OX_9031_GN_GCH1_PE_2_SV_
CollodictyonidaeEP00002Diphylleiarotans_11663641_GCH1_OSTOS_GTP_cyclohydrolase_1_OS_Ostertagia_ostertagi_OX_6317_GN_gch_PE_2_SV_
RigifilidaEP00004Rigifilaramosa_23118611_GCH1_OSTOS_GTP_cyclohydrolase_1_OS_Ostertagia_ostertagi_OX_6317_GN_gch_PE_2_SV_
FungiFusariumoxysporumUP000002489_13357941_GCH1_NEUCR_GTP_cyclohydrolase_1_OS_Neurospora_crassa__strain_ATCC_24698_
FungiFusariumoxysporumUP000002489_13366296_GCH1_NEUCR_GTP_cyclohydrolase_1_OS_Neurospora_crassa__strain_ATCC_24698_
FungiAspergillusnigerUP000068243_13140958_GCH1_NEUCR_GTP_cyclohydrolase_1_OS_Neurospora_crassa__strain_ATCC_24698_
FungiMortierellaelongataUP000078512_13383706_GCH1_SYNY3_GTP_cyclohydrolase_1_OS_Synechocystis_sp___strain_PCC_6803_
FungiMortierellaelongataUP000078512_13385031_GCH1_NEUCR_GTP_cyclohydrolase_1_OS_Neurospora_crassa__strain_ATCC_24698_
FungiMortierellaelongataUP000078512_13385612_GCH1_NEUCR_GTP_cyclohydrolase_1_OS_Neurospora_crassa__strain_ATCC_24698_
FungiRhizopusmicrosporusUP000242254_13428954_GCH1_SCHPO_GTP_cyclohydrolase_1_OS_Schizosaccharomyces_pombe__strain_972_
FungiSpizellomycespunctatusUP000053201_13458684_GCH1_SCHPO_GTP_cyclohydrolase_1_OS_Schizosaccharomyces_pombe__strain_972_
RotosphaeridaEP00160Parvulariaatlantis_23172479_GCH1_SCHPO_GTP_cyclohydrolase_1_OS_Schizosaccharomyces_pombe__strain_972_
FungiAgaricusbisporusUP000008493_13107514_GCH1_NEUCR_GTP_cyclohydrolase_1_OS_Neurospora_crassa__strain_ATCC_24698_
FungiEP00150Ustilagomaydis_13257280_GCH1_SCHPO_GTP_cyclohydrolase_1_OS_Schizosaccharomyces_pombe__strain_972_
FilastreaCapsasporaowczarzakiUP000008743_12983158_GCH1_HUMAN_GTP_cyclohydrolase_1_OS_Homo_sapiens_OX_9606_GN_GCH1_PE_1_SV_
PluriformeaEP00128Syssomonasmultiformis_21627332_GCH1_MOUSE_GTP_cyclohydrolase_1_OS_Mus_musculus_OX_10090_GN_Gch1_PE_1_SV_
PluriformeaCorallochytriumlimacisporum_21622023_GCH1_RAT_GTP_cyclohydrolase_1_OS_Rattus_norvegicus_OX_10116_GN_Gch1_PE_1_SV_
IchthyosporeaAmoebidiumparasiticum_14568508_GCH1_SCHPO_GTP_cyclohydrolase_1_OS_Schizosaccharomyces_pombe__strain_972_
IchthyosporeaSphaerothecumdestruens_14599277_GCH1_NEUCR_GTP_cyclohydrolase_1_OS_Neurospora_crassa__strain_ATCC_24698_
0.47

3D.

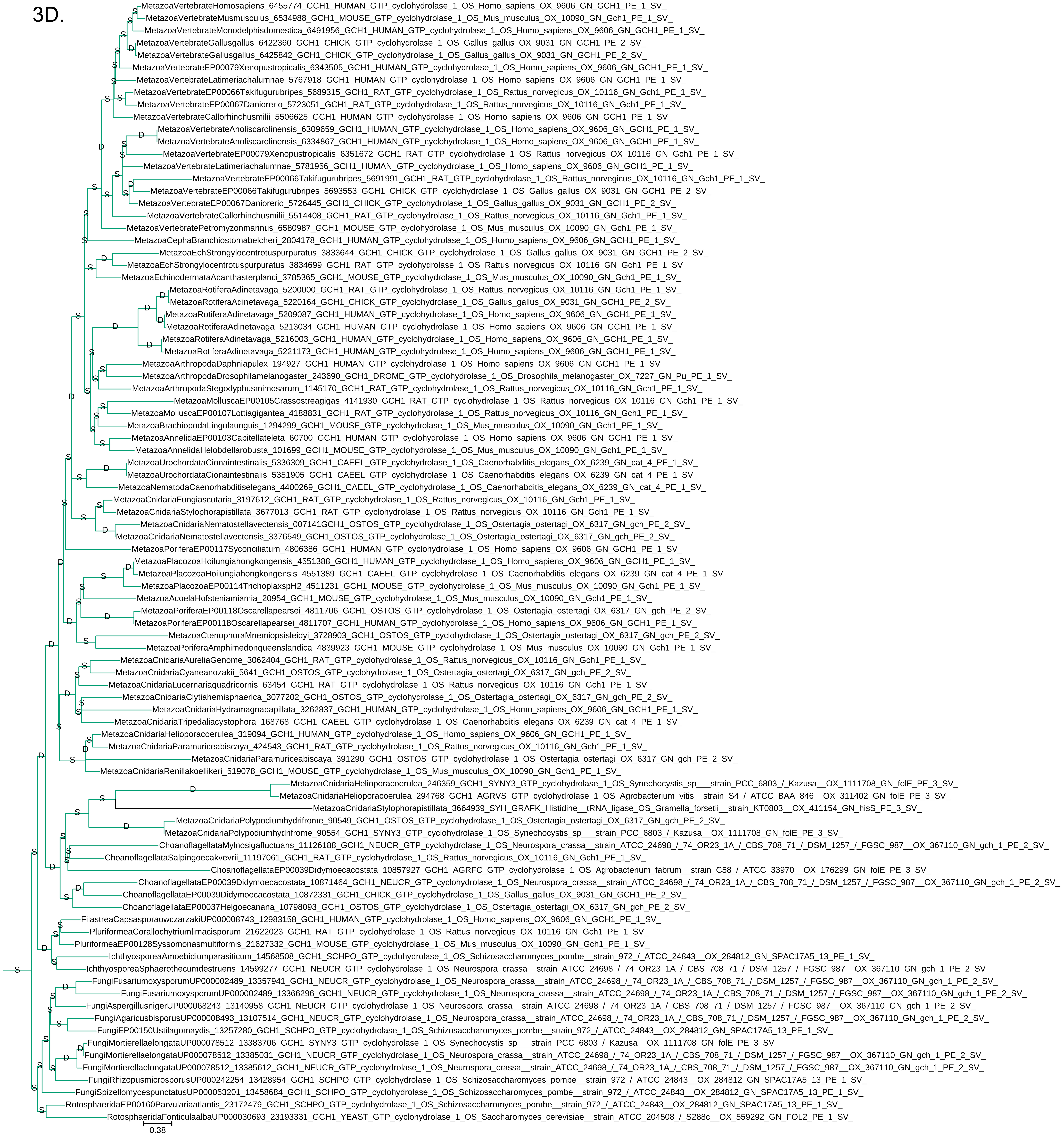
MetazoaVertebrateHomosapiens_6455774_GCH1_HUMAN_GTP_cyclohydrolase_1_OS_Homo_sapiens_OX_9606_GN_GCH1_PE_1_SV_
MetazoaVertebrateMusmusculus_6534988_GCH1_MOUSE_GTP_cyclohydrolase_1_OS_Mus_musculus_OX_10090_GN_Gch1_PE_1_SV_
MetazoaVertebrateMonodelphisdomestica_6491956_GCH1_HUMAN_GTP_cyclohydrolase_1_OS_Homo_sapiens_OX_9606_GN_GCH1_PE_1_SV_
MetazoaVertebrateGallusgallus_6422360_GCH1_CHICK_GTP_cyclohydrolase_1_OS_Gallus_gallus_OX_9031_GN_GCH1_PE_2_SV_
MetazoaVertebrateGallusgallus_6425842_GCH1_CHICK_GTP_cyclohydrolase_1_OS_Gallus_gallus_OX_9031_GN_GCH1_PE_2_SV_
MetazoaVertebrateEP00079Xenopustropicalis_6343505_GCH1_HUMAN_GTP_cyclohydrolase_1_OS_Homo_sapiens_OX_9606_GN_GCH1_PE_1_SV_
MetazoaVertebrateLatimeriachalumnae_5767918_GCH1_HUMAN_GTP_cyclohydrolase_1_OS_Homo_sapiens_OX_9606_GN_GCH1_PE_1_SV_
MetazoaVertebrateEP00066Takifugurubripes_5689315_GCH1_RAT_GTP_cyclohydrolase_1_OS_Rattus_norvegicus_OX_10116_GN_Gch1_PE_1_SV_
MetazoaVertebrateEP00067Daniorerio_5723051_GCH1_RAT_GTP_cyclohydrolase_1_OS_Rattus_norvegicus_OX_10116_GN_Gch1_PE_1_SV_
MetazoaVertebrateCallorhinchusmilii_5506625_GCH1_HUMAN_GTP_cyclohydrolase_1_OS_Homo_sapiens_OX_9606_GN_GCH1_PE_1_SV_
MetazoaVertebrateAnoliscarolinensis_6309659_GCH1_HUMAN_GTP_cyclohydrolase_1_OS_Homo_sapiens_OX_9606_GN_GCH1_PE_1_SV_
MetazoaVertebrateAnoliscarolinensis_6334867_GCH1_HUMAN_GTP_cyclohydrolase_1_OS_Homo_sapiens_OX_9606_GN_GCH1_PE_1_SV_
MetazoaVertebrateEP00079Xenopustropicalis_6351672_GCH1_RAT_GTP_cyclohydrolase_1_OS_Rattus_norvegicus_OX_10116_GN_Gch1_PE_1_SV_
MetazoaVertebrateLatimeriachalumnae_5781956_GCH1_HUMAN_GTP_cyclohydrolase_1_OS_Homo_sapiens_OX_9606_GN_GCH1_PE_1_SV_
MetazoaVertebrateEP00066Takifugurubripes_5691991_GCH1_RAT_GTP_cyclohydrolase_1_OS_Rattus_norvegicus_OX_10116_GN_Gch1_PE_1_SV_
MetazoaVertebrateEP00066Takifugurubripes_5693553_GCH1_CHICK_GTP_cyclohydrolase_1_OS_Gallus_gallus_OX_9031_GN_GCH1_PE_2_SV_
MetazoaVertebrateEP00067Daniorerio_5726445_GCH1_CHICK_GTP_cyclohydrolase_1_OS_Gallus_gallus_OX_9031_GN_GCH1_PE_2_SV_
MetazoaVertebrateCallorhinchusmilii_5514408_GCH1_RAT_GTP_cyclohydrolase_1_OS_Rattus_norvegicus_OX_10116_GN_Gch1_PE_1_SV_
MetazoaVertebratePetromyzonmarinus_6580987_GCH1_MOUSE_GTP_cyclohydrolase_1_OS_Mus_musculus_OX_10090_GN_Gch1_PE_1_SV_
MetazoaCephaBranchiostomabelcheri_2804178_GCH1_HUMAN_GTP_cyclohydrolase_1_OS_Homo_sapiens_OX_9606_GN_GCH1_PE_1_SV_
MetazoaEchStrongylocentrotuspurpuratus_3833644_GCH1_CHICK_GTP_cyclohydrolase_1_OS_Gallus_gallus_OX_9031_GN_GCH1_PE_2_SV_
MetazoaEchStrongylocentrotuspurpuratus_3834699_GCH1_RAT_GTP_cyclohydrolase_1_OS_Rattus_norvegicus_OX_10116_GN_Gch1_PE_1_SV_
MetazoaEchinodermataAcanthasterplanci_3785365_GCH1_MOUSE_GTP_cyclohydrolase_1_OS_Mus_musculus_OX_10090_GN_Gch1_PE_1_SV_
MetazoaRotiferaAdinetavaga_5200000_GCH1_RAT_GTP_cyclohydrolase_1_OS_Rattus_norvegicus_OX_10116_GN_Gch1_PE_1_SV_
MetazoaRotiferaAdinetavaga_5220164_GCH1_CHICK_GTP_cyclohydrolase_1_OS_Gallus_gallus_OX_9031_GN_GCH1_PE_2_SV_
MetazoaRotiferaAdinetavaga_5209087_GCH1_HUMAN_GTP_cyclohydrolase_1_OS_Homo_sapiens_OX_9606_GN_GCH1_PE_1_SV_
MetazoaRotiferaAdinetavaga_5213034_GCH1_HUMAN_GTP_cyclohydrolase_1_OS_Homo_sapiens_OX_9606_GN_GCH1_PE_1_SV_
MetazoaRotiferaAdinetavaga_5216003_GCH1_HUMAN_GTP_cyclohydrolase_1_OS_Homo_sapiens_OX_9606_GN_GCH1_PE_1_SV_
MetazoaRotiferaAdinetavaga_5221173_GCH1_HUMAN_GTP_cyclohydrolase_1_OS_Homo_sapiens_OX_9606_GN_GCH1_PE_1_SV_
MetazoaArthropodaDaphniapulex_194927_GCH1_HUMAN_GTP_cyclohydrolase_1_OS_Homo_sapiens_OX_9606_GN_GCH1_PE_1_SV_
MetazoaArthropodaDrosophilamelanogaster_243690_GCH1_DROME_GTP_cyclohydrolase_1_OS_Drosophila_melanogaster_OX_7227_GN_Pu_PE_1_SV_
MetazoaArthropodaStegodyphusmimosarum_1145170_GCH1_RAT_GTP_cyclohydrolase_1_OS_Rattus_norvegicus_OX_10116_GN_Gch1_PE_1_SV_
MetazoaMolluscaEP00105Crassostreagigas_4141930_GCH1_RAT_GTP_cyclohydrolase_1_OS_Rattus_norvegicus_OX_10116_GN_Gch1_PE_1_SV_
MetazoaMolluscaEP00107Lottiagigantea_4188831_GCH1_RAT_GTP_cyclohydrolase_1_OS_Rattus_norvegicus_OX_10116_GN_Gch1_PE_1_SV_
MetazoaBrachiopodaLingulaunguis_1294299_GCH1_MOUSE_GTP_cyclohydrolase_1_OS_Mus_musculus_OX_10090_GN_Gch1_PE_1_SV_
MetazoaAnnelidaEP00103Capitellateleta_60700_GCH1_HUMAN_GTP_cyclohydrolase_1_OS_Homo_sapiens_OX_9606_GN_GCH1_PE_1_SV_
MetazoaAnnelidaHelobdellarobusta_101699_GCH1_MOUSE_GTP_cyclohydrolase_1_OS_Mus_musculus_OX_10090_GN_Gch1_PE_1_SV_
MetazoaUrochordataCionaintestinalis_5336309_GCH1_CAEEL_GTP_cyclohydrolase_1_OS_Caenorhabditis_elegans_OX_6239_GN_cat_4_PE_1_SV_
MetazoaUrochordataCionaintestinalis_5351905_GCH1_CAEEL_GTP_cyclohydrolase_1_OS_Caenorhabditis_elegans_OX_6239_GN_cat_4_PE_1_SV_
MetazoaNematodaCaenorhabditiselegans_4400269_GCH1_CAEEL_GTP_cyclohydrolase_1_OS_Caenorhabditis_elegans_OX_6239_GN_cat_4_PE_1_SV_
MetazoaCnidariaFungiascutaria_3197612_GCH1_RAT_GTP_cyclohydrolase_1_OS_Rattus_norvegicus_OX_10116_GN_Gch1_PE_1_SV_
MetazoaCnidariaStylophorapistillata_3677013_GCH1_RAT_GTP_cyclohydrolase_1_OS_Rattus_norvegicus_OX_10116_GN_Gch1_PE_1_SV_
MetazoaCnidariaNematostellavectensis_007141GCH1_OSTOS_GTP_cyclohydrolase_1_OS_Ostertagia_ostertagi_OX_6317_GN_gch_PE_2_SV_
MetazoaCnidariaNematostellavectensis_3376549_GCH1_OSTOS_GTP_cyclohydrolase_1_OS_Ostertagia_ostertagi_OX_6317_GN_gch_PE_2_SV_
MetazoaPoriferaEP00117Syconciliatum_4806386_GCH1_HUMAN_GTP_cyclohydrolase_1_OS_Homo_sapiens_OX_9606_GN_GCH1_PE_1_SV_
MetazoaPlacozoaHoilungiahongkongensis_4551388_GCH1_HUMAN_GTP_cyclohydrolase_1_OS_Homo_sapiens_OX_9606_GN_GCH1_PE_1_SV_
MetazoaPlacozoaHoilungiahongkongensis_4551389_GCH1_CAEEL_GTP_cyclohydrolase_1_OS_Caenorhabditis_elegans_OX_6239_GN_cat_4_PE_1_SV_
MetazoaPlacozoaEP00114TrichoplaxspH2_4511231_GCH1_MOUSE_GTP_cyclohydrolase_1_OS_Mus_musculus_OX_10090_GN_Gch1_PE_1_SV_
MetazoaAcoelaHofsteniamiamia_20954_GCH1_MOUSE_GTP_cyclohydrolase_1_OS_Mus_musculus_OX_10090_GN_Gch1_PE_1_SV_
MetazoaPoriferaEP00118Oscarellapearsei_4811706_GCH1_OSTOS_GTP_cyclohydrolase_1_OS_Ostertagia_ostertagi_OX_6317_GN_gch_PE_2_SV_
MetazoaPoriferaEP00118Oscarellapearsei_4811707_GCH1_HUMAN_GTP_cyclohydrolase_1_OS_Homo_sapiens_OX_9606_GN_GCH1_PE_1_SV_
MetazoaCtenophoraMnemiopsisleidyi_3728903_GCH1_OSTOS_GTP_cyclohydrolase_1_OS_Ostertagia_ostertagi_OX_6317_GN_gch_PE_2_SV_
MetazoaPoriferaAmphimedonqueenslandica_4839923_GCH1_MOUSE_GTP_cyclohydrolase_1_OS_Mus_musculus_OX_10090_GN_Gch1_PE_1_SV_
MetazoaCnidariaAureliaGenome_3062404_GCH1_RAT_GTP_cyclohydrolase_1_OS_Rattus_norvegicus_OX_10116_GN_Gch1_PE_1_SV_
MetazoaCnidariaCyaneanozakii_5641_GCH1_OSTOS_GTP_cyclohydrolase_1_OS_Ostertagia_ostertagi_OX_6317_GN_gch_PE_2_SV_
MetazoaCnidariaLucernariaquadricornis_63454_GCH1_RAT_GTP_cyclohydrolase_1_OS_Rattus_norvegicus_OX_10116_GN_Gch1_PE_1_SV_
MetazoaCnidariaClytiahemisphaerica_3077202_GCH1_OSTOS_GTP_cyclohydrolase_1_OS_Ostertagia_ostertagi_OX_6317_GN_gch_PE_2_SV_
MetazoaCnidariaHydramagnapapillata_3262837_GCH1_HUMAN_GTP_cyclohydrolase_1_OS_Homo_sapiens_OX_9606_GN_GCH1_PE_1_SV_
MetazoaCnidariaTripedaliacystophora_168768_GCH1_CAEEL_GTP_cyclohydrolase_1_OS_Caenorhabditis_elegans_OX_6239_GN_cat_4_PE_1_SV_
MetazoaCnidariaHelioporacoerulea_319094_GCH1_HUMAN_GTP_cyclohydrolase_1_OS_Homo_sapiens_OX_9606_GN_GCH1_PE_1_SV_
MetazoaCnidariaParamuriceabiscaya_424543_GCH1_RAT_GTP_cyclohydrolase_1_OS_Rattus_norvegicus_OX_10116_GN_Gch1_PE_1_SV_
MetazoaCnidariaParamuriceabiscaya_391290_GCH1_OSTOS_GTP_cyclohydrolase_1_OS_Ostertagia_ostertagi_OX_6317_GN_gch_PE_2_SV_
MetazoaCnidariaRenillakoellikeri_519078_GCH1_MOUSE_GTP_cyclohydrolase_1_OS_Mus_musculus_OX_10090_GN_Gch1_PE_1_SV_
MetazoaCnidariaHelioporacoerulea_246359_GCH1_SYNY3_GTP_cyclohydrolase_1_OS_Synechocystis_sp___strain_PCC_6803_/_Kazusa__OX_1111708_GN_folE_PE_3_SV_
MetazoaCnidariaHelioporacoerulea_294768_GCH1_AGRVS_GTP_cyclohydrolase_1_OS_Agrobacterium_vitis__strain_S4_/_ATCC_BAA_846__OX_311402_GN_folE_PE_3_SV_
MetazoaCnidariaStylophorapistillata_3664939_SYH_GRAFK_Histidine__tRNA_ligase_OS_Gramella_forsetii__strain_KT0803__OX_411154_GN_hisS_PE_3_SV_
MetazoaCnidariaPolypodiumhydrifrome_90549_GCH1_OSTOS_GTP_cyclohydrolase_1_OS_Ostertagia_ostertagi_OX_6317_GN_gch_PE_2_SV_
MetazoaCnidariaPolypodiumhydrifrome_90554_GCH1_SYNY3_GTP_cyclohydrolase_1_OS_Synechocystis_sp___strain_PCC_6803_/_Kazusa__OX_1111708_GN_folE_PE_3_SV_
ChoanoflagellataMylnosigafluctuans_11126188_GCH1_NEUCR_GTP_cyclohydrolase_1_OS_Neurospora_crassa__strain_ATCC_24698_/_74_OR23_1A_/_CBS_708_71_/_DSM_1257_/_FGSC_987__OX_367110_GN_gch_1_PE_2_SV_
ChoanoflagellataSalpingoecakvevrii_11197061_GCH1_RAT_GTP_cyclohydrolase_1_OS_Rattus_norvegicus_OX_10116_GN_Gch1_PE_1_SV_
ChoanoflagellataEP00039Didymoecacostata_10857927_GCH1_AGRFC_GTP_cyclohydrolase_1_OS_Agrobacterium_fabrum__strain_C58_/_ATCC_33970__OX_176299_GN_folE_PE_3_SV_
ChoanoflagellataEP00039Didymoecacostata_10871464_GCH1_NEUCR_GTP_cyclohydrolase_1_OS_Neurospora_crassa__strain_ATCC_24698_/_74_OR23_1A_/_CBS_708_71_/_DSM_1257_/_FGSC_987__OX_367110_GN_gch_1_PE_2_SV_
ChoanoflagellataEP00039Didymoecacostata_10872331_GCH1_CHICK_GTP_cyclohydrolase_1_OS_Gallus_gallus_OX_9031_GN_GCH1_PE_2_SV_
ChoanoflagellataEP00037Helgoecanana_10798093_GCH1_OSTOS_GTP_cyclohydrolase_1_OS_Ostertagia_ostertagi_OX_6317_GN_gch_PE_2_SV_
FilastreaCapsasporaowczarzakiUP000008743_12983158_GCH1_HUMAN_GTP_cyclohydrolase_1_OS_Homo_sapiens_OX_9606_GN_GCH1_PE_1_SV_
PluriformeaCorallochytriumlimacisporum_21622023_GCH1_RAT_GTP_cyclohydrolase_1_OS_Rattus_norvegicus_OX_10116_GN_Gch1_PE_1_SV_
PluriformeaEP00128Syssomonasmultiformis_21627332_GCH1_MOUSE_GTP_cyclohydrolase_1_OS_Mus_musculus_OX_10090_GN_Gch1_PE_1_SV_
IchthyosporeaAmoebidiumparasiticum_14568508_GCH1_SCHPO_GTP_cyclohydrolase_1_OS_Schizosaccharomyces_pombe__strain_972_/_ATCC_24843__OX_284812_GN_SPAC17A5_13_PE_1_SV_
IchthyosporeaSphaerothecumdestruens_14599277_GCH1_NEUCR_GTP_cyclohydrolase_1_OS_Neurospora_crassa__strain_ATCC_24698_/_74_OR23_1A_/_CBS_708_71_/_DSM_1257_/_FGSC_987__OX_367110_GN_gch_1_PE_2_SV_
FungiFusariumoxysporumUP000002489_13357941_GCH1_NEUCR_GTP_cyclohydrolase_1_OS_Neurospora_crassa__strain_ATCC_24698_/_74_OR23_1A_/_CBS_708_71_/_DSM_1257_/_FGSC_987__OX_367110_GN_gch_1_PE_2_SV_
FungiFusariumoxysporumUP000002489_13366296_GCH1_NEUCR_GTP_cyclohydrolase_1_OS_Neurospora_crassa__strain_ATCC_24698_/_74_OR23_1A_/_CBS_708_71_/_DSM_1257_/_FGSC_987__OX_367110_GN_gch_1_PE_2_SV_
FungiAspergillusnigerUP000068243_13140958_GCH1_NEUCR_GTP_cyclohydrolase_1_OS_Neurospora_crassa__strain_ATCC_24698_/_74_OR23_1A_/_CBS_708_71_/_DSM_1257_/_FGSC_987__OX_367110_GN_gch_1_PE_2_SV_
FungiAgaricusbisporusUP000008493_13107514_GCH1_NEUCR_GTP_cyclohydrolase_1_OS_Neurospora_crassa__strain_ATCC_24698_/_74_OR23_1A_/_CBS_708_71_/_DSM_1257_/_FGSC_987__OX_367110_GN_gch_1_PE_2_SV_
FungiEP00150Ustilagomaydis_13257280_GCH1_SCHPO_GTP_cyclohydrolase_1_OS_Schizosaccharomyces_pombe__strain_972_/_ATCC_24843__OX_284812_GN_SPAC17A5_13_PE_1_SV_
FungiMortierellaelongataUP000078512_13383706_GCH1_SYNY3_GTP_cyclohydrolase_1_OS_Synechocystis_sp___strain_PCC_6803_/_Kazusa__OX_1111708_GN_folE_PE_3_SV_
FungiMortierellaelongataUP000078512_13385031_GCH1_NEUCR_GTP_cyclohydrolase_1_OS_Neurospora_crassa__strain_ATCC_24698_/_74_OR23_1A_/_CBS_708_71_/_DSM_1257_/_FGSC_987__OX_367110_GN_gch_1_PE_2_SV_
FungiMortierellaelongataUP000078512_13385612_GCH1_NEUCR_GTP_cyclohydrolase_1_OS_Neurospora_crassa__strain_ATCC_24698_/_74_OR23_1A_/_CBS_708_71_/_DSM_1257_/_FGSC_987__OX_367110_GN_gch_1_PE_2_SV_
FungiRhizopusmicrosporusUP000242254_13428954_GCH1_SCHPO_GTP_cyclohydrolase_1_OS_Schizosaccharomyces_pombe__strain_972_/_ATCC_24843__OX_284812_GN_SPAC17A5_13_PE_1_SV_
FungiSpizellomycespunctatusUP000053201_13458684_GCH1_SCHPO_GTP_cyclohydrolase_1_OS_Schizosaccharomyces_pombe__strain_972_/_ATCC_24843__OX_284812_GN_SPAC17A5_13_PE_1_SV_
RotosphaeridaEP00160Parvulariaatlantis_23172479_GCH1_SCHPO_GTP_cyclohydrolase_1_OS_Schizosaccharomyces_pombe__strain_972_/_ATCC_24843__OX_284812_GN_SPAC17A5_13_PE_1_SV_
RotosphaeridaFonticulaalbaUP000030693_23193331_GCH1_YEAST_GTP_cyclohydrolase_1_OS_Saccharomyces_cerevisiae__strain_ATCC_204508_/_S288c__OX_559292_GN_FOL2_PE_1_SV_
0.38

3E.

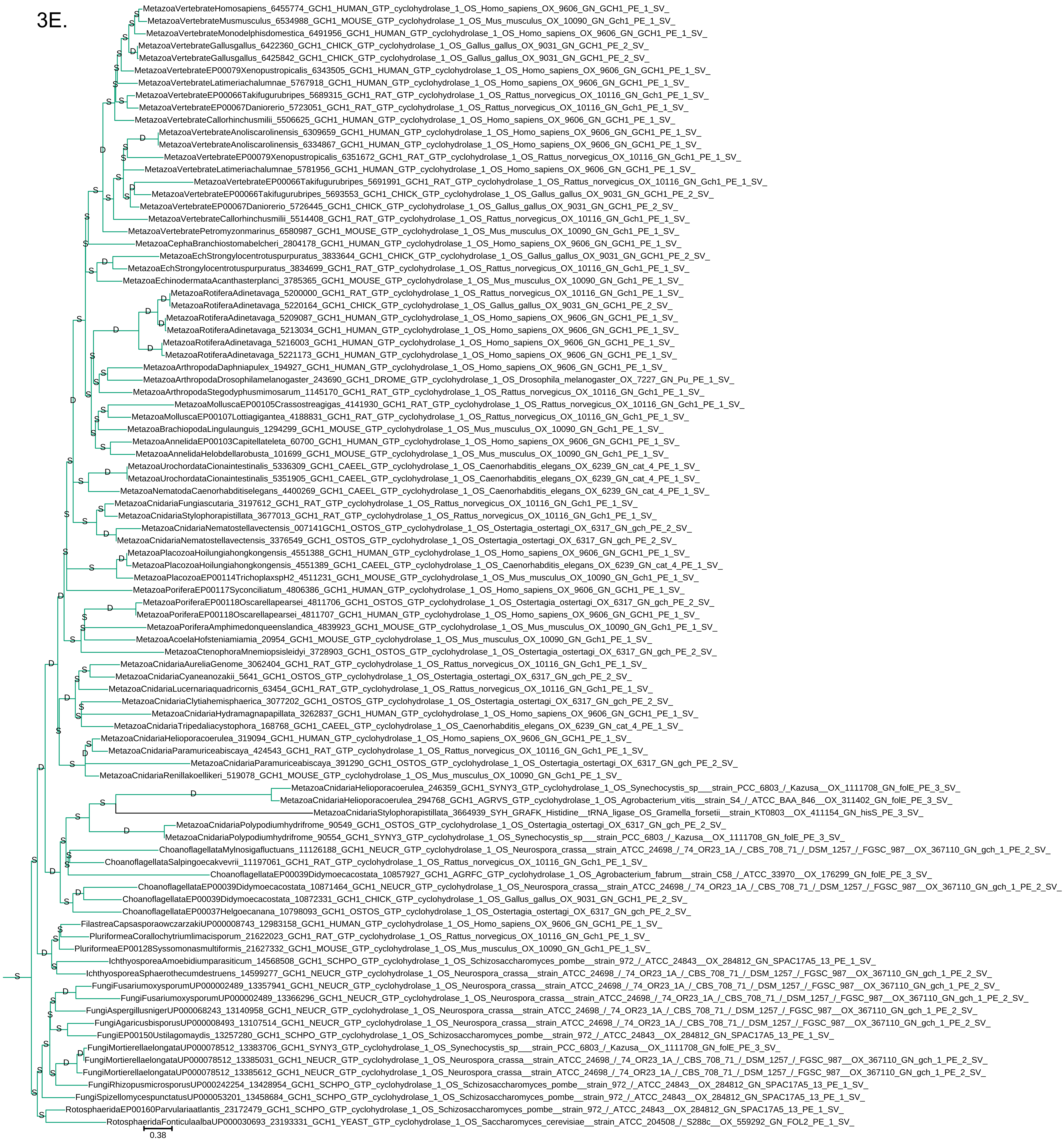
MetazoaVertebrateHomosapiens_6455774_GCH1_HUMAN_GTP_cyclohydrolase_1_OS_Homo_sapiens_OX_9606_GN_GCH1_PE_1_SV_
MetazoaVertebrateMusmusculus_6534988_GCH1_MOUSE_GTP_cyclohydrolase_1_OS_Mus_musculus_OX_10090_GN_Gch1_PE_1_SV_
MetazoaVertebrateMonodelphisdomestica_6491956_GCH1_HUMAN_GTP_cyclohydrolase_1_OS_Homo_sapiens_OX_9606_GN_GCH1_PE_1_SV_
MetazoaVertebrateGallusgallus_6422360_GCH1_CHICK_GTP_cyclohydrolase_1_OS_Gallus_gallus_OX_9031_GN_GCH1_PE_2_SV_
MetazoaVertebrateGallusgallus_6425842_GCH1_CHICK_GTP_cyclohydrolase_1_OS_Gallus_gallus_OX_9031_GN_GCH1_PE_2_SV_
MetazoaVertebrateEP00079Xenopustropicalis_6343505_GCH1_HUMAN_GTP_cyclohydrolase_1_OS_Homo_sapiens_OX_9606_GN_GCH1_PE_1_SV_
MetazoaVertebrateLatimeriachalumnae_5767918_GCH1_HUMAN_GTP_cyclohydrolase_1_OS_Homo_sapiens_OX_9606_GN_GCH1_PE_1_SV_
MetazoaVertebrateEP00066Takifugurubripes_5689315_GCH1_RAT_GTP_cyclohydrolase_1_OS_Rattus_norvegicus_OX_10116_GN_Gch1_PE_1_SV_
MetazoaVertebrateEP00067Daniorerio_5723051_GCH1_RAT_GTP_cyclohydrolase_1_OS_Rattus_norvegicus_OX_10116_GN_Gch1_PE_1_SV_
MetazoaVertebrateCallorhinchusmilii_5506625_GCH1_HUMAN_GTP_cyclohydrolase_1_OS_Homo_sapiens_OX_9606_GN_GCH1_PE_1_SV_
MetazoaVertebrateAnoliscarolinensis_6309659_GCH1_HUMAN_GTP_cyclohydrolase_1_OS_Homo_sapiens_OX_9606_GN_GCH1_PE_1_SV_
MetazoaVertebrateAnoliscarolinensis_6334867_GCH1_HUMAN_GTP_cyclohydrolase_1_OS_Homo_sapiens_OX_9606_GN_GCH1_PE_1_SV_
MetazoaVertebrateEP00079Xenopustropicalis_6351672_GCH1_RAT_GTP_cyclohydrolase_1_OS_Rattus_norvegicus_OX_10116_GN_Gch1_PE_1_SV_
MetazoaVertebrateLatimeriachalumnae_5781956_GCH1_HUMAN_GTP_cyclohydrolase_1_OS_Homo_sapiens_OX_9606_GN_GCH1_PE_1_SV_
MetazoaVertebrateEP00066Takifugurubripes_5691991_GCH1_RAT_GTP_cyclohydrolase_1_OS_Rattus_norvegicus_OX_10116_GN_Gch1_PE_1_SV_
MetazoaVertebrateEP00066Takifugurubripes_5693553_GCH1_CHICK_GTP_cyclohydrolase_1_OS_Gallus_gallus_OX_9031_GN_GCH1_PE_2_SV_
MetazoaVertebrateEP00067Daniorerio_5726445_GCH1_CHICK_GTP_cyclohydrolase_1_OS_Gallus_gallus_OX_9031_GN_GCH1_PE_2_SV_
MetazoaVertebrateCallorhinchusmilii_5514408_GCH1_RAT_GTP_cyclohydrolase_1_OS_Rattus_norvegicus_OX_10116_GN_Gch1_PE_1_SV_
MetazoaVertebratePetromyzonmarinus_6580987_GCH1_MOUSE_GTP_cyclohydrolase_1_OS_Mus_musculus_OX_10090_GN_Gch1_PE_1_SV_
MetazoaCephaBranchiostomabelcheri_2804178_GCH1_HUMAN_GTP_cyclohydrolase_1_OS_Homo_sapiens_OX_9606_GN_GCH1_PE_1_SV_
MetazoaEchStrongylocentrotuspurpuratus_3833644_GCH1_CHICK_GTP_cyclohydrolase_1_OS_Gallus_gallus_OX_9031_GN_GCH1_PE_2_SV_
MetazoaEchStrongylocentrotuspurpuratus_3834699_GCH1_RAT_GTP_cyclohydrolase_1_OS_Rattus_norvegicus_OX_10116_GN_Gch1_PE_1_SV_
MetazoaEchinodermataAcanthasterplanci_3785365_GCH1_MOUSE_GTP_cyclohydrolase_1_OS_Mus_musculus_OX_10090_GN_Gch1_PE_1_SV_
MetazoaRotiferaAdinetavaga_5200000_GCH1_RAT_GTP_cyclohydrolase_1_OS_Rattus_norvegicus_OX_10116_GN_Gch1_PE_1_SV_
MetazoaRotiferaAdinetavaga_5220164_GCH1_CHICK_GTP_cyclohydrolase_1_OS_Gallus_gallus_OX_9031_GN_GCH1_PE_2_SV_
MetazoaRotiferaAdinetavaga_5209087_GCH1_HUMAN_GTP_cyclohydrolase_1_OS_Homo_sapiens_OX_9606_GN_GCH1_PE_1_SV_
MetazoaRotiferaAdinetavaga_5213034_GCH1_HUMAN_GTP_cyclohydrolase_1_OS_Homo_sapiens_OX_9606_GN_GCH1_PE_1_SV_
MetazoaRotiferaAdinetavaga_5216003_GCH1_HUMAN_GTP_cyclohydrolase_1_OS_Homo_sapiens_OX_9606_GN_GCH1_PE_1_SV_
MetazoaRotiferaAdinetavaga_5221173_GCH1_HUMAN_GTP_cyclohydrolase_1_OS_Homo_sapiens_OX_9606_GN_GCH1_PE_1_SV_
MetazoaArthropodaDaphniapulex_194927_GCH1_HUMAN_GTP_cyclohydrolase_1_OS_Homo_sapiens_OX_9606_GN_GCH1_PE_1_SV_
MetazoaArthropodaDrosophilamelanogaster_243690_GCH1_DROME_GTP_cyclohydrolase_1_OS_Drosophila_melanogaster_OX_7227_GN_Pu_PE_1_SV_
MetazoaArthropodaStegodyphusmimosarum_1145170_GCH1_RAT_GTP_cyclohydrolase_1_OS_Rattus_norvegicus_OX_10116_GN_Gch1_PE_1_SV_
MetazoaMolluscaEP00105Crassostreagigas_4141930_GCH1_RAT_GTP_cyclohydrolase_1_OS_Rattus_norvegicus_OX_10116_GN_Gch1_PE_1_SV_
MetazoaMolluscaEP00107Lottiagigantea_4188831_GCH1_RAT_GTP_cyclohydrolase_1_OS_Rattus_norvegicus_OX_10116_GN_Gch1_PE_1_SV_
MetazoaBrachiopodaLingulaunguis_1294299_GCH1_MOUSE_GTP_cyclohydrolase_1_OS_Mus_musculus_OX_10090_GN_Gch1_PE_1_SV_
MetazoaAnnelidaEP00103Capitellateleta_60700_GCH1_HUMAN_GTP_cyclohydrolase_1_OS_Homo_sapiens_OX_9606_GN_GCH1_PE_1_SV_
MetazoaAnnelidaHelobdellarobusta_101699_GCH1_MOUSE_GTP_cyclohydrolase_1_OS_Mus_musculus_OX_10090_GN_Gch1_PE_1_SV_
MetazoaUrochordataCionaintestinalis_5336309_GCH1_CAEEL_GTP_cyclohydrolase_1_OS_Caenorhabditis_elegans_OX_6239_GN_cat_4_PE_1_SV_
MetazoaUrochordataCionaintestinalis_5351905_GCH1_CAEEL_GTP_cyclohydrolase_1_OS_Caenorhabditis_elegans_OX_6239_GN_cat_4_PE_1_SV_
MetazoaNematodaCaenorhabditiselegans_4400269_GCH1_CAEEL_GTP_cyclohydrolase_1_OS_Caenorhabditis_elegans_OX_6239_GN_cat_4_PE_1_SV_
MetazoaCnidariaFungiascutaria_3197612_GCH1_RAT_GTP_cyclohydrolase_1_OS_Rattus_norvegicus_OX_10116_GN_Gch1_PE_1_SV_
MetazoaCnidariaStylophorapistillata_3677013_GCH1_RAT_GTP_cyclohydrolase_1_OS_Rattus_norvegicus_OX_10116_GN_Gch1_PE_1_SV_
MetazoaCnidariaNematostellavectensis_007141GCH1_OSTOS_GTP_cyclohydrolase_1_OS_Ostertagia_ostertagi_OX_6317_GN_gch_PE_2_SV_
MetazoaCnidariaNematostellavectensis_3376549_GCH1_OSTOS_GTP_cyclohydrolase_1_OS_Ostertagia_ostertagi_OX_6317_GN_gch_PE_2_SV_
MetazoaPlacozoaHoilungiahongkongensis_4551388_GCH1_HUMAN_GTP_cyclohydrolase_1_OS_Homo_sapiens_OX_9606_GN_GCH1_PE_1_SV_
MetazoaPlacozoaHoilungiahongkongensis_4551389_GCH1_CAEEL_GTP_cyclohydrolase_1_OS_Caenorhabditis_elegans_OX_6239_GN_cat_4_PE_1_SV_
MetazoaPlacozoaEP00114TrichoplaxspH2_4511231_GCH1_MOUSE_GTP_cyclohydrolase_1_OS_Mus_musculus_OX_10090_GN_Gch1_PE_1_SV_
MetazoaPoriferaEP00117Syconciliatum_4806386_GCH1_HUMAN_GTP_cyclohydrolase_1_OS_Homo_sapiens_OX_9606_GN_GCH1_PE_1_SV_
MetazoaPoriferaEP00118Oscarellapearsei_4811706_GCH1_OSTOS_GTP_cyclohydrolase_1_OS_Ostertagia_ostertagi_OX_6317_GN_gch_PE_2_SV_
MetazoaPoriferaEP00118Oscarellapearsei_4811707_GCH1_HUMAN_GTP_cyclohydrolase_1_OS_Homo_sapiens_OX_9606_GN_GCH1_PE_1_SV_
MetazoaPoriferaAmphimedonqueenslandica_4839923_GCH1_MOUSE_GTP_cyclohydrolase_1_OS_Mus_musculus_OX_10090_GN_Gch1_PE_1_SV_
MetazoaAcoelaHofsteniamiamia_20954_GCH1_MOUSE_GTP_cyclohydrolase_1_OS_Mus_musculus_OX_10090_GN_Gch1_PE_1_SV_
MetazoaCtenophoraMnemiopsisleidyi_3728903_GCH1_OSTOS_GTP_cyclohydrolase_1_OS_Ostertagia_ostertagi_OX_6317_GN_gch_PE_2_SV_
MetazoaCnidariaAureliaGenome_3062404_GCH1_RAT_GTP_cyclohydrolase_1_OS_Rattus_norvegicus_OX_10116_GN_Gch1_PE_1_SV_
MetazoaCnidariaCyaneanozakii_5641_GCH1_OSTOS_GTP_cyclohydrolase_1_OS_Ostertagia_ostertagi_OX_6317_GN_gch_PE_2_SV_
MetazoaCnidariaLucernariaquadricornis_63454_GCH1_RAT_GTP_cyclohydrolase_1_OS_Rattus_norvegicus_OX_10116_GN_Gch1_PE_1_SV_
MetazoaCnidariaClytiahemisphaerica_3077202_GCH1_OSTOS_GTP_cyclohydrolase_1_OS_Ostertagia_ostertagi_OX_6317_GN_gch_PE_2_SV_
MetazoaCnidariaHydramagnapapillata_3262837_GCH1_HUMAN_GTP_cyclohydrolase_1_OS_Homo_sapiens_OX_9606_GN_GCH1_PE_1_SV_
MetazoaCnidariaTripedaliacystophora_168768_GCH1_CAEEL_GTP_cyclohydrolase_1_OS_Caenorhabditis_elegans_OX_6239_GN_cat_4_PE_1_SV_
MetazoaCnidariaHelioporacoerulea_319094_GCH1_HUMAN_GTP_cyclohydrolase_1_OS_Homo_sapiens_OX_9606_GN_GCH1_PE_1_SV_
MetazoaCnidariaParamuriceabiscaya_424543_GCH1_RAT_GTP_cyclohydrolase_1_OS_Rattus_norvegicus_OX_10116_GN_Gch1_PE_1_SV_
MetazoaCnidariaParamuriceabiscaya_391290_GCH1_OSTOS_GTP_cyclohydrolase_1_OS_Ostertagia_ostertagi_OX_6317_GN_gch_PE_2_SV_
MetazoaCnidariaRenillakoellikeri_519078_GCH1_MOUSE_GTP_cyclohydrolase_1_OS_Mus_musculus_OX_10090_GN_Gch1_PE_1_SV_
MetazoaCnidariaHelioporacoerulea_246359_GCH1_SYNY3_GTP_cyclohydrolase_1_OS_Synechocystis_sp___strain_PCC_6803_/_Kazusa__OX_1111708_GN_folE_PE_3_SV_
MetazoaCnidariaHelioporacoerulea_294768_GCH1_AGRVS_GTP_cyclohydrolase_1_OS_Agrobacterium_vitis__strain_S4_/_ATCC_BAA_846__OX_311402_GN_folE_PE_3_SV_
MetazoaCnidariaStylophorapistillata_3664939_SYH_GRAFK_Histidine__tRNA_ligase_OS_Gramella_forsetii__strain_KT0803__OX_411154_GN_hisS_PE_3_SV_
MetazoaCnidariaPolypodiumhydrifrome_90549_GCH1_OSTOS_GTP_cyclohydrolase_1_OS_Ostertagia_ostertagi_OX_6317_GN_gch_PE_2_SV_
MetazoaCnidariaPolypodiumhydrifrome_90554_GCH1_SYNY3_GTP_cyclohydrolase_1_OS_Synechocystis_sp___strain_PCC_6803_/_Kazusa__OX_1111708_GN_folE_PE_3_SV_
ChoanoflagellataMylnosigafluctuans_11126188_GCH1_NEUCR_GTP_cyclohydrolase_1_OS_Neurospora_crassa__strain_ATCC_24698_/_74_OR23_1A_/_CBS_708_71_/_DSM_1257_/_FGSC_987__OX_367110_GN_gch_1_PE_2_SV_
ChoanoflagellataSalpingoecakvevrii_11197061_GCH1_RAT_GTP_cyclohydrolase_1_OS_Rattus_norvegicus_OX_10116_GN_Gch1_PE_1_SV_
ChoanoflagellataEP00039Didymoecacostata_10857927_GCH1_AGRFC_GTP_cyclohydrolase_1_OS_Agrobacterium_fabrum__strain_C58_/_ATCC_33970__OX_176299_GN_folE_PE_3_SV_
ChoanoflagellataEP00039Didymoecacostata_10871464_GCH1_NEUCR_GTP_cyclohydrolase_1_OS_Neurospora_crassa__strain_ATCC_24698_/_74_OR23_1A_/_CBS_708_71_/_DSM_1257_/_FGSC_987__OX_367110_GN_gch_1_PE_2_SV_
ChoanoflagellataEP00039Didymoecacostata_10872331_GCH1_CHICK_GTP_cyclohydrolase_1_OS_Gallus_gallus_OX_9031_GN_GCH1_PE_2_SV_
ChoanoflagellataEP00037Helgoecanana_10798093_GCH1_OSTOS_GTP_cyclohydrolase_1_OS_Ostertagia_ostertagi_OX_6317_GN_gch_PE_2_SV_
FilastreaCapsasporaowczarzakiUP000008743_12983158_GCH1_HUMAN_GTP_cyclohydrolase_1_OS_Homo_sapiens_OX_9606_GN_GCH1_PE_1_SV_
PluriformeaCorallochytriumlimacisporum_21622023_GCH1_RAT_GTP_cyclohydrolase_1_OS_Rattus_norvegicus_OX_10116_GN_Gch1_PE_1_SV_
PluriformeaEP00128Syssomonasmultiformis_21627332_GCH1_MOUSE_GTP_cyclohydrolase_1_OS_Mus_musculus_OX_10090_GN_Gch1_PE_1_SV_
IchthyosporeaAmoebidiumparasiticum_14568508_GCH1_SCHPO_GTP_cyclohydrolase_1_OS_Schizosaccharomyces_pombe__strain_972_/_ATCC_24843__OX_284812_GN_SPAC17A5_13_PE_1_SV_
IchthyosporeaSphaerothecumdestruens_14599277_GCH1_NEUCR_GTP_cyclohydrolase_1_OS_Neurospora_crassa__strain_ATCC_24698_/_74_OR23_1A_/_CBS_708_71_/_DSM_1257_/_FGSC_987__OX_367110_GN_gch_1_PE_2_SV_
FungiFusariumoxysporumUP000002489_13357941_GCH1_NEUCR_GTP_cyclohydrolase_1_OS_Neurospora_crassa__strain_ATCC_24698_/_74_OR23_1A_/_CBS_708_71_/_DSM_1257_/_FGSC_987__OX_367110_GN_gch_1_PE_2_SV_
FungiFusariumoxysporumUP000002489_13366296_GCH1_NEUCR_GTP_cyclohydrolase_1_OS_Neurospora_crassa__strain_ATCC_24698_/_74_OR23_1A_/_CBS_708_71_/_DSM_1257_/_FGSC_987__OX_367110_GN_gch_1_PE_2_SV_
FungiAspergillusnigerUP000068243_13140958_GCH1_NEUCR_GTP_cyclohydrolase_1_OS_Neurospora_crassa__strain_ATCC_24698_/_74_OR23_1A_/_CBS_708_71_/_DSM_1257_/_FGSC_987__OX_367110_GN_gch_1_PE_2_SV_
FungiAgaricusbisporusUP000008493_13107514_GCH1_NEUCR_GTP_cyclohydrolase_1_OS_Neurospora_crassa__strain_ATCC_24698_/_74_OR23_1A_/_CBS_708_71_/_DSM_1257_/_FGSC_987__OX_367110_GN_gch_1_PE_2_SV_
FungiEP00150Ustilagomaydis_13257280_GCH1_SCHPO_GTP_cyclohydrolase_1_OS_Schizosaccharomyces_pombe__strain_972_/_ATCC_24843__OX_284812_GN_SPAC17A5_13_PE_1_SV_
FungiMortierellaelongataUP000078512_13383706_GCH1_SYNY3_GTP_cyclohydrolase_1_OS_Synechocystis_sp___strain_PCC_6803_/_Kazusa__OX_1111708_GN_folE_PE_3_SV_
FungiMortierellaelongataUP000078512_13385031_GCH1_NEUCR_GTP_cyclohydrolase_1_OS_Neurospora_crassa__strain_ATCC_24698_/_74_OR23_1A_/_CBS_708_71_/_DSM_1257_/_FGSC_987__OX_367110_GN_gch_1_PE_2_SV_
FungiMortierellaelongataUP000078512_13385612_GCH1_NEUCR_GTP_cyclohydrolase_1_OS_Neurospora_crassa__strain_ATCC_24698_/_74_OR23_1A_/_CBS_708_71_/_DSM_1257_/_FGSC_987__OX_367110_GN_gch_1_PE_2_SV_
FungiRhizopusmicrosporusUP000242254_13428954_GCH1_SCHPO_GTP_cyclohydrolase_1_OS_Schizosaccharomyces_pombe__strain_972_/_ATCC_24843__OX_284812_GN_SPAC17A5_13_PE_1_SV_
FungiSpizellomycespunctatusUP000053201_13458684_GCH1_SCHPO_GTP_cyclohydrolase_1_OS_Schizosaccharomyces_pombe__strain_972_/_ATCC_24843__OX_284812_GN_SPAC17A5_13_PE_1_SV_
RotosphaeridaEP00160Parvulariaatlantis_23172479_GCH1_SCHPO_GTP_cyclohydrolase_1_OS_Schizosaccharomyces_pombe__strain_972_/_ATCC_24843__OX_284812_GN_SPAC17A5_13_PE_1_SV_
RotosphaeridaFonticulaalbaUP000030693_23193331_GCH1_YEAST_GTP_cyclohydrolase_1_OS_Saccharomyces_cerevisiae__strain_ATCC_204508_/_S288c__OX_559292_GN_FOL2_PE_1_SV_
0.38

3F.

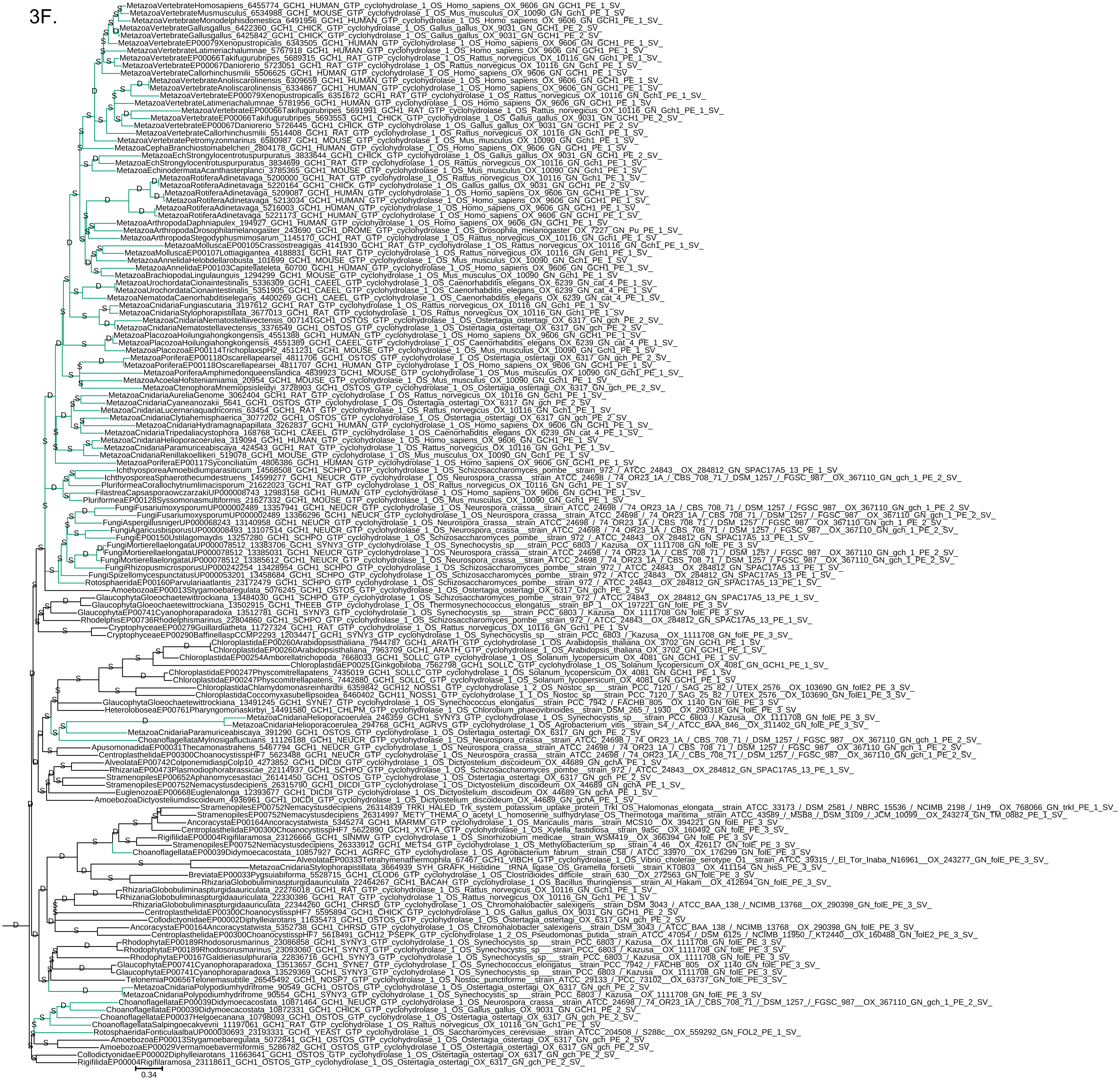
MetazoaVertebrateHomosapiens_6455774_GCH1_HUMAN_GTP_cyclohydrolase_1_OS_Homo_sapiens_OX_9606_GN_GCH1_PE_1_SV_
MetazoaVertebrateMusmusculus_6534988_GCH1_MOUSE_GTP_cyclohydrolase_1_OS_Mus_musculus_OX_10090_GN_Gch1_PE_1_SV_
MetazoaVertebrateMonodelphisdomestica_6491956_GCH1_HUMAN_GTP_cyclohydrolase_1_OS_Homo_sapiens_OX_9606_GN_GCH1_PE_1_SV_
MetazoaVertebrateGallusgallus_6422360_GCH1_CHICK_GTP_cyclohydrolase_1_OS_Gallus_gallus_OX_9031_GN_GCH1_PE_2_SV_
MetazoaVertebrateGallusgallus_6425842_GCH1_CHICK_GTP_cyclohydrolase_1_OS_Gallus_gallus_OX_9031_GN_GCH1_PE_2_SV_
MetazoaVertebrateEP00079Xenopustropicalis_6343505_GCH1_HUMAN_GTP_cyclohydrolase_1_OS_Homo_sapiens_OX_9606_GN_GCH1_PE_1_SV_
MetazoaVertebrateLatimeriachalumnae_5767918_GCH1_HUMAN_GTP_cyclohydrolase_1_OS_Homo_sapiens_OX_9606_GN_GCH1_PE_1_SV_
MetazoaVertebrateEP00066Takifugurubripes_5689315_GCH1_RAT_GTP_cyclohydrolase_1_OS_Rattus_norvegicus_OX_10116_GN_Gch1_PE_1_SV_
MetazoaVertebrateEP00067Daniorerio_5723051_GCH1_RAT_GTP_cyclohydrolase_1_OS_Rattus_norvegicus_OX_10116_GN_Gch1_PE_1_SV_
MetazoaVertebrateCallorhinchusmilii_5506625_GCH1_HUMAN_GTP_cyclohydrolase_1_OS_Homo_sapiens_OX_9606_GN_GCH1_PE_1_SV_
MetazoaVertebrateAnoliscarolinensis_6309659_GCH1_HUMAN_GTP_cyclohydrolase_1_OS_Homo_sapiens_OX_9606_GN_GCH1_PE_1_SV_
MetazoaVertebrateAnoliscarolinensis_6334867_GCH1_HUMAN_GTP_cyclohydrolase_1_OS_Homo_sapiens_OX_9606_GN_GCH1_PE_1_SV_
MetazoaVertebrateEP00079Xenopustropicalis_6351672_GCH1_RAT_GTP_cyclohydrolase_1_OS_Rattus_norvegicus_OX_10116_GN_Gch1_PE_1_SV_
MetazoaVertebrateLatimeriachalumnae_5781956_GCH1_HUMAN_GTP_cyclohydrolase_1_OS_Homo_sapiens_OX_9606_GN_GCH1_PE_1_SV_
MetazoaVertebrateEP00066Takifugurubripes_5691991_GCH1_RAT_GTP_cyclohydrolase_1_OS_Rattus_norvegicus_OX_10116_GN_Gch1_PE_1_SV_
MetazoaVertebrateEP00066Takifugurubripes_5693553_GCH1_CHICK_GTP_cyclohydrolase_1_OS_Gallus_gallus_OX_9031_GN_GCH1_PE_2_SV_
MetazoaVertebrateEP00067Daniorerio_5726445_GCH1_CHICK_GTP_cyclohydrolase_1_OS_Gallus_gallus_OX_9031_GN_GCH1_PE_2_SV_
MetazoaVertebrateCallorhinchusmilii_5514408_GCH1_RAT_GTP_cyclohydrolase_1_OS_Rattus_norvegicus_OX_10116_GN_Gch1_PE_1_SV_
MetazoaVertebratePetromyzonmarinus_6580987_GCH1_MOUSE_GTP_cyclohydrolase_1_OS_Mus_musculus_OX_10090_GN_Gch1_PE_1_SV_
MetazoaCephaBranchiostomabelcheri_2804178_GCH1_HUMAN_GTP_cyclohydrolase_1_OS_Homo_sapiens_OX_9606_GN_GCH1_PE_1_SV_
MetazoaEchStrongylocentrotuspurpuratus_3833644_GCH1_CHICK_GTP_cyclohydrolase_1_OS_Gallus_gallus_OX_9031_GN_GCH1_PE_2_SV_
MetazoaEchStrongylocentrotuspurpuratus_3834699_GCH1_RAT_GTP_cyclohydrolase_1_OS_Rattus_norvegicus_OX_10116_GN_Gch1_PE_1_SV_
MetazoaEchinodermataAcanthasterplanci_3785365_GCH1_MOUSE_GTP_cyclohydrolase_1_OS_Mus_musculus_OX_10090_GN_Gch1_PE_1_SV_
MetazoaRotiferaAdinetavaga_5200000_GCH1_RAT_GTP_cyclohydrolase_1_OS_Rattus_norvegicus_OX_10116_GN_Gch1_PE_1_SV_
MetazoaRotiferaAdinetavaga_5220164_GCH1_CHICK_GTP_cyclohydrolase_1_OS_Gallus_gallus_OX_9031_GN_GCH1_PE_2_SV_
MetazoaRotiferaAdinetavaga_5209087_GCH1_HUMAN_GTP_cyclohydrolase_1_OS_Homo_sapiens_OX_9606_GN_GCH1_PE_1_SV_
MetazoaRotiferaAdinetavaga_5213034_GCH1_HUMAN_GTP_cyclohydrolase_1_OS_Homo_sapiens_OX_9606_GN_GCH1_PE_1_SV_
MetazoaRotiferaAdinetavaga_5216003_GCH1_HUMAN_GTP_cyclohydrolase_1_OS_Homo_sapiens_OX_9606_GN_GCH1_PE_1_SV_
MetazoaRotiferaAdinetavaga_5221173_GCH1_HUMAN_GTP_cyclohydrolase_1_OS_Homo_sapiens_OX_9606_GN_GCH1_PE_1_SV_
MetazoaArthropodaDaphniapulex_194927_GCH1_HUMAN_GTP_cyclohydrolase_1_OS_Homo_sapiens_OX_9606_GN_GCH1_PE_1_SV_
MetazoaArthropodaDrosophilamelanogaster_243690_GCH1_DROME_GTP_cyclohydrolase_1_OS_Drosophila_melanogaster_OX_7227_GN_Pu_PE_1_SV_
MetazoaArthropodaStegodyphusmimosarum_1145170_GCH1_RAT_GTP_cyclohydrolase_1_OS_Rattus_norvegicus_OX_10116_GN_Gch1_PE_1_SV_
MetazoaMolluscaEP00105Crassostreagigas_4141930_GCH1_RAT_GTP_cyclohydrolase_1_OS_Rattus_norvegicus_OX_10116_GN_Gch1_PE_1_SV_
MetazoaMolluscaEP00107Lottiagigantea_4188831_GCH1_RAT_GTP_cyclohydrolase_1_OS_Rattus_norvegicus_OX_10116_GN_Gch1_PE_1_SV_
MetazoaAnnelidaHelobdellarobusta_101699_GCH1_MOUSE_GTP_cyclohydrolase_1_OS_Mus_musculus_OX_10090_GN_Gch1_PE_1_SV_
MetazoaAnnelidaEP00103Capitellateleta_60700_GCH1_HUMAN_GTP_cyclohydrolase_1_OS_Homo_sapiens_OX_9606_GN_GCH1_PE_1_SV_
MetazoaBrachiopodaLingulaunguis_1294299_GCH1_MOUSE_GTP_cyclohydrolase_1_OS_Mus_musculus_OX_10090_GN_Gch1_PE_1_SV_
MetazoaUrochordataCionaintestinalis_5336309_GCH1_CAEEL_GTP_cyclohydrolase_1_OS_Caenorhabditis_elegans_OX_6239_GN_cat_4_PE_1_SV_
MetazoaUrochordataCionaintestinalis_5351905_GCH1_CAEEL_GTP_cyclohydrolase_1_OS_Caenorhabditis_elegans_OX_6239_GN_cat_4_PE_1_SV_
MetazoaNematodaCaenorhabditiselegans_4400269_GCH1_CAEEL_GTP_cyclohydrolase_1_OS_Caenorhabditis_elegans_OX_6239_GN_cat_4_PE_1_SV_
MetazoaCnidariaFungiascutaria_3197612_GCH1_RAT_GTP_cyclohydrolase_1_OS_Rattus_norvegicus_OX_10116_GN_Gch1_PE_1_SV_
MetazoaCnidariaStylophorapistillata_3677013_GCH1_RAT_GTP_cyclohydrolase_1_OS_Rattus_norvegicus_OX_10116_GN_Gch1_PE_1_SV_
MetazoaCnidariaNematostellavectensis_007141GCH1_OSTOS_GTP_cyclohydrolase_1_OS_Ostertagia_ostertagi_OX_6317_GN_gch_PE_2_SV_
MetazoaCnidariaNematostellavectensis_3376549_GCH1_OSTOS_GTP_cyclohydrolase_1_OS_Ostertagia_ostertagi_OX_6317_GN_gch_PE_2_SV_
MetazoaPlacozoaHoilungiahongkongensis_4551388_GCH1_HUMAN_GTP_cyclohydrolase_1_OS_Homo_sapiens_OX_9606_GN_GCH1_PE_1_SV_
MetazoaPlacozoaHoilungiahongkongensis_4551389_GCH1_CAEEL_GTP_cyclohydrolase_1_OS_Caenorhabditis_elegans_OX_6239_GN_cat_4_PE_1_SV_
MetazoaPlacozoaEP00114TrichoplaxspH2_4511231_GCH1_MOUSE_GTP_cyclohydrolase_1_OS_Mus_musculus_OX_10090_GN_Gch1_PE_1_SV_
MetazoaPoriferaEP00118Oscarellapearsei_4811706_GCH1_OSTOS_GTP_cyclohydrolase_1_OS_Ostertagia_ostertagi_OX_6317_GN_gch_PE_2_SV_
MetazoaPoriferaEP00118Oscarellapearsei_4811707_GCH1_HUMAN_GTP_cyclohydrolase_1_OS_Homo_sapiens_OX_9606_GN_GCH1_PE_1_SV_
MetazoaPoriferaAmphimedonqueenslandica_4839923_GCH1_MOUSE_GTP_cyclohydrolase_1_OS_Mus_musculus_OX_10090_GN_Gch1_PE_1_SV_
MetazoaAcoelaHofsteniamiamia_20954_GCH1_MOUSE_GTP_cyclohydrolase_1_OS_Mus_musculus_OX_10090_GN_Gch1_PE_1_SV_
MetazoaCtenophoraMnemiopsisleidyi_3728903_GCH1_OSTOS_GTP_cyclohydrolase_1_OS_Ostertagia_ostertagi_OX_6317_GN_gch_PE_2_SV_
MetazoaCnidariaAureliaGenome_3062404_GCH1_RAT_GTP_cyclohydrolase_1_OS_Rattus_norvegicus_OX_10116_GN_Gch1_PE_1_SV_
MetazoaCnidariaCyaneanozakii_5641_GCH1_OSTOS_GTP_cyclohydrolase_1_OS_Ostertagia_ostertagi_OX_6317_GN_gch_PE_2_SV_
MetazoaCnidariaLucernariaquadricornis_63454_GCH1_RAT_GTP_cyclohydrolase_1_OS_Rattus_norvegicus_OX_10116_GN_Gch1_PE_1_SV_
MetazoaCnidariaClytiahemisphaerica_3077202_GCH1_OSTOS_GTP_cyclohydrolase_1_OS_Ostertagia_ostertagi_OX_6317_GN_gch_PE_2_SV_
MetazoaCnidariaHydramagnapapillata_3262837_GCH1_HUMAN_GTP_cyclohydrolase_1_OS_Homo_sapiens_OX_9606_GN_GCH1_PE_1_SV_
MetazoaCnidariaTripedaliacystophora_168768_GCH1_CAEEL_GTP_cyclohydrolase_1_OS_Caenorhabditis_elegans_OX_6239_GN_cat_4_PE_1_SV_
MetazoaCnidariaHelioporacoerulea_319094_GCH1_HUMAN_GTP_cyclohydrolase_1_OS_Homo_sapiens_OX_9606_GN_GCH1_PE_1_SV_
MetazoaCnidariaParamuriceabiscaya_424543_GCH1_RAT_GTP_cyclohydrolase_1_OS_Rattus_norvegicus_OX_10116_GN_Gch1_PE_1_SV_
MetazoaCnidariaRenillakoellikeri_519078_GCH1_MOUSE_GTP_cyclohydrolase_1_OS_Mus_musculus_OX_10090_GN_Gch1_PE_1_SV_
MetazoaPoriferaEP00117Syconciliatum_4806386_GCH1_HUMAN_GTP_cyclohydrolase_1_OS_Homo_sapiens_OX_9606_GN_GCH1_PE_1_SV_
IchthyosporeaAmoebidiumparasiticum_14568508_GCH1_SCHPO_GTP_cyclohydrolase_1_OS_Schizosaccharomyces_pombe__strain_972_/_ATCC_24843__OX_284812_GN_SPAC17A5_13_PE_1_SV_
IchthyosporeaSphaerothecumdestruens_14599277_GCH1_NEUCR_GTP_cyclohydrolase_1_OS_Neurospora_crassa__strain_ATCC_24698_/_74_OR23_1A_/_CBS_708_71_/_DSM_1257_/_FGSC_987__OX_367110_GN_gch_1_PE_2_SV_
PluriformeaCorallochytriumlimacisporum_21622023_GCH1_RAT_GTP_cyclohydrolase_1_OS_Rattus_norvegicus_OX_10116_GN_Gch1_PE_1_SV_
FilastreaCapsasporaowczarzakiUP000008743_12983158_GCH1_HUMAN_GTP_cyclohydrolase_1_OS_Homo_sapiens_OX_9606_GN_GCH1_PE_1_SV_
PluriformeaEP00128Syssomonasmultiformis_21627332_GCH1_MOUSE_GTP_cyclohydrolase_1_OS_Mus_musculus_OX_10090_GN_Gch1_PE_1_SV_
FungiFusariumoxysporumUP000002489_13357941_GCH1_NEUCR_GTP_cyclohydrolase_1_OS_Neurospora_crassa__strain_ATCC_24698_/_74_OR23_1A_/_CBS_708_71_/_DSM_1257_/_FGSC_987__OX_367110_GN_gch_1_PE_2_SV_
FungiFusariumoxysporumUP000002489_13366296_GCH1_NEUCR_GTP_cyclohydrolase_1_OS_Neurospora_crassa__strain_ATCC_24698_/_74_OR23_1A_/_CBS_708_71_/_DSM_1257_/_FGSC_987__OX_367110_GN_gch_1_PE_2_SV_
FungiAspergillusnigerUP000068243_13140958_GCH1_NEUCR_GTP_cyclohydrolase_1_OS_Neurospora_crassa__strain_ATCC_24698_/_74_OR23_1A_/_CBS_708_71_/_DSM_1257_/_FGSC_987__OX_367110_GN_gch_1_PE_2_SV_
FungiAgaricusbisporusUP000008493_13107514_GCH1_NEUCR_GTP_cyclohydrolase_1_OS_Neurospora_crassa__strain_ATCC_24698_/_74_OR23_1A_/_CBS_708_71_/_DSM_1257_/_FGSC_987__OX_367110_GN_gch_1_PE_2_SV_
FungiEP00150Ustilagomaydis_13257280_GCH1_SCHPO_GTP_cyclohydrolase_1_OS_Schizosaccharomyces_pombe__strain_972_/_ATCC_24843__OX_284812_GN_SPAC17A5_13_PE_1_SV_
FungiMortierellaelongataUP000078512_13383706_GCH1_SYNY3_GTP_cyclohydrolase_1_OS_Synechocystis_sp___strain_PCC_6803_/_Kazusa__OX_1111708_GN_folE_PE_3_SV_
FungiMortierellaelongataUP000078512_13385031_GCH1_NEUCR_GTP_cyclohydrolase_1_OS_Neurospora_crassa__strain_ATCC_24698_/_74_OR23_1A_/_CBS_708_71_/_DSM_1257_/_FGSC_987__OX_367110_GN_gch_1_PE_2_SV_
FungiMortierellaelongataUP000078512_13385612_GCH1_NEUCR_GTP_cyclohydrolase_1_OS_Neurospora_crassa__strain_ATCC_24698_/_74_OR23_1A_/_CBS_708_71_/_DSM_1257_/_FGSC_987__OX_367110_GN_gch_1_PE_2_SV_
FungiRhizopusmicrosporusUP000242254_13428954_GCH1_SCHPO_GTP_cyclohydrolase_1_OS_Schizosaccharomyces_pombe__strain_972_/_ATCC_24843__OX_284812_GN_SPAC17A5_13_PE_1_SV_
FungiSpizellomycespunctatusUP000053201_13458684_GCH1_SCHPO_GTP_cyclohydrolase_1_OS_Schizosaccharomyces_pombe__strain_972_/_ATCC_24843__OX_284812_GN_SPAC17A5_13_PE_1_SV_
RotosphaeridaEP00160Parvulariaatlantis_23172479_GCH1_SCHPO_GTP_cyclohydrolase_1_OS_Schizosaccharomyces_pombe__strain_972_/_ATCC_24843__OX_284812_GN_SPAC17A5_13_PE_1_SV_
AmoebozoaEP00013Stygamoebaregulata_5076245_GCH1_OSTOS_GTP_cyclohydrolase_1_OS_Ostertagia_ostertagi_OX_6317_GN_gch_PE_2_SV_
GlaucophytaGloeochaetewittrockiana_13484030_GCH1_SCHPO_GTP_cyclohydrolase_1_OS_Schizosaccharomyces_pombe__strain_972_/_ATCC_24843__OX_284812_GN_SPAC17A5_13_PE_1_SV_
GlaucophytaGloeochaetewittrockiana_13502915_GCH1_THEEB_GTP_cyclohydrolase_1_OS_Thermosynechococcus_elongatus__strain_BP_1__OX_197221_GN_folE_PE_3_SV_
GlaucophytaEP00741Cyanophoraparadoxa_13512781_GCH1_SYNY3_GTP_cyclohydrolase_1_OS_Synechocystis_sp___strain_PCC_6803_/_Kazusa__OX_1111708_GN_folE_PE_3_SV_
RhodelphisEP00736Rhodelphismarinus_22804860_GCH1_SCHPO_GTP_cyclohydrolase_1_OS_Schizosaccharomyces_pombe__strain_972_/_ATCC_24843__OX_284812_GN_SPAC17A5_13_PE_1_SV_
CryptophyceaeEP00279Guillardiatheta_11727324_GCH1_RAT_GTP_cyclohydrolase_1_OS_Rattus_norvegicus_OX_10116_GN_Gch1_PE_1_SV_
CryptophyceaeEP00290BaffinellasCCMP2293_13044471_GCH1_SYNY3_GTP_cyclohydrolase_1_OS_Synechocystis_sp___strain_PCC_6803_/_Kazusa__OX_1111708_GN_folE_PE_3_SV_
ChloroplastidaEP00260Arabidopsisthaliana_7944787_GCH1_ARATH_GTP_cyclohydrolase_1_OS_Arabidopsis_thaliana_OX_3702_GN_GCH1_PE_1_SV_
ChloroplastidaEP00260Arabidopsisthaliana_7963709_GCH1_ARATH_GTP_cyclohydrolase_1_OS_Arabidopsis_thaliana_OX_3702_GN_GCH1_PE_1_SV_
ChloroplastidaEP00254Amborellatrichopoda_7668033_GCH1_SOLLC_GTP_cyclohydrolase_1_OS_Solanum_lycopersicum_OX_4081_GN_GCH1_PE_1_SV_
ChloroplastidaEP00251Ginkgobiloba_7562798_GCH1_SOLLC_GTP_cyclohydrolase_1_OS_Solanum_lycopersicum_OX_4081_GN_GCH1_PE_1_SV_
ChloroplastidaEP00247Physcomitrellapatens_7435019_GCH1_SOLLC_GTP_cyclohydrolase_1_OS_Solanum_lycopersicum_OX_4081_GN_GCH1_PE_1_SV_
ChloroplastidaEP00247Physcomitrellapatens_7442880_GCH1_SOLLC_GTP_cyclohydrolase_1_OS_Solanum_lycopersicum_OX_4081_GN_GCH1_PE_1_SV_
ChloroplastidaChlamydomonasreinhardtii_6359842_GCH12_NOSS1_GTP_cyclohydrolase_1_2_OS_Nostoc_sp___strain_PCC_7120_/_SAG_25_82_/_UTEX_2576__OX_103690_GN_folE2_PE_3_SV_
ChloroplastidaCoccomyxasubellipsoidea_6460402_GCH11_NOSS1_GTP_cyclohydrolase_1_1_OS_Nostoc_sp___strain_PCC_7120_/_SAG_25_82_/_UTEX_2576__OX_103690_GN_folE1_PE_3_SV_
GlaucophytaGloeochaetewittrockiana_13491245_GCH1_SYNE7_GTP_cyclohydrolase_1_OS_Synechococcus_elongatus__strain_PCC_7942_/_FACHB_805__OX_1140_GN_folE_PE_3_SV_
HeteroloboseaEP00761Pharyngomonaskirbyi_14491580_GCH1_CHLPM_GTP_cyclohydrolase_1_OS_Chlorobium_phaeovibrioides__strain_DSM_265_/_1930__OX_290318_GN_folE_PE_3_SV_
MetazoaCnidariaHelioporacoerulea_246359_GCH1_SYNY3_GTP_cyclohydrolase_1_OS_Synechocystis_sp___strain_PCC_6803_/_Kazusa__OX_1111708_GN_folE_PE_3_SV_
MetazoaCnidariaHelioporacoerulea_294768_GCH1_AGRVS_GTP_cyclohydrolase_1_OS_Agrobacterium_vitis__strain_S4_/_ATCC_BAA_846__OX_311402_GN_folE_PE_3_SV_
MetazoaCnidariaParamuriceabiscaya_391290_GCH1_OSTOS_GTP_cyclohydrolase_1_OS_Ostertagia_ostertagi_OX_6317_GN_gch_PE_2_SV_
ChoanoflagellataMylnosigafluctuans_11126188_GCH1_NEUCR_GTP_cyclohydrolase_1_OS_Neurospora_crassa__strain_ATCC_24698_/_74_OR23_1A_/_CBS_708_71_/_DSM_1257_/_FGSC_987__OX_367110_GN_gch_1_PE_2_SV_
ApusomonadidaEP00031Thecamonastrahens_5467794_GCH1_NEUCR_GTP_cyclohydrolase_1_OS_Neurospora_crassa__strain_ATCC_24698_/_74_OR23_1A_/_CBS_708_71_/_DSM_1257_/_FGSC_987__OX_367110_GN_gch_1_PE_2_SV_
CentroplasthelidaEP00300ChoanocystisspHF7_5623488_GCH1_NEUCR_GTP_cyclohydrolase_1_OS_Neurospora_crassa__strain_ATCC_24698_/_74_OR23_1A_/_CBS_708_71_/_DSM_1257_/_FGSC_987__OX_367110_GN_gch_1_PE_2_SV_
AlveolataEP00742Colponemidiasp Colp10_4273852_GCH1_DICDI_GTP_cyclohydrolase_1_OS_Dictyostelium_discoideum_OX_44689_GN_gchA_PE_1_SV_
RhizariaEP00473Plasmodiophorabrassicae_22114937_GCH1_SCHPO_GTP_cyclohydrolase_1_OS_Schizosaccharomyces_pombe__strain_972_/_ATCC_24843__OX_284812_GN_SPAC17A5_13_PE_1_SV_
StramenopilesEP00652Aphanomycesastaci_26141450_GCH1_OSTOS_GTP_cyclohydrolase_1_OS_Ostertagia_ostertagi_OX_6317_GN_gch_PE_2_SV_
StramenopilesEP00752Nemacystusdecipiens_26315790_GCH1_DICDI_GTP_cyclohydrolase_1_OS_Dictyostelium_discoideum_OX_44689_GN_gchA_PE_1_SV_
EuglenozoaEP00668Euglenalonga_12393677_GCH1_DICDI_GTP_cyclohydrolase_1_OS_Dictyostelium_discoideum_OX_44689_GN_gchA_PE_1_SV_
AmoebozoaDictyosteliumdiscoideum_4936961_GCH1_DICDI_GTP_cyclohydrolase_1_OS_Dictyostelium_discoideum_OX_44689_GN_gchA_PE_1_SV_
StramenopilesEP00752Nemacystusdecipiens_26314839_TRKI_HALED_Trk_system_potassium_uptake_protein_TrkI_OS_Halomonas_elongata__strain_ATCC_33173_/_DSM_2581_/_NBRC_15536_/_NCIMB_2198_/_1H9__OX_768066_GN_trkI_PE_1_SV_
StramenopilesEP00752Nemacystusdecipiens_26314997_METY_THEMA_O_acetyl_L_homoserine_sulfhydrylase_OS_Thermotoga_maritima__strain_ATCC_43589_/_MSB8_/_DSM_3109_/_JCM_10099__OX_243274_GN_TM_0882_PE_1_SV_
AncoracystaEP00164Ancoracystatwista_5345274_GCH1_MARMM_GTP_cyclohydrolase_1_OS_Maricaulis_maris__strain_MCS10__OX_394221_GN_folE_PE_3_SV_
CentroplasthelidaEP00300ChoanocystisspHF7_5622890_GCH1_XYLFA_GTP_cyclohydrolase_1_OS_Xylella_fastidiosa__strain_9a5c__OX_160492_GN_folE_PE_3_SV_
RigifilidaEP00004Rigifilaramosa_23126666_GCH1_SINMW_GTP_cyclohydrolase_1_OS_Sinorhizobium_medicae__strain_WSM419__OX_366394_GN_folE_PE_3_SV_
StramenopilesEP00752Nemacystusdecipiens_26333912_GCH1_METS4_GTP_cyclohydrolase_1_OS_Methylobacterium_sp___strain_4_46__OX_426117_GN_folE_PE_3_SV_
ChoanoflagellataEP00039Didymoecacostata_10857927_GCH1_AGRFC_GTP_cyclohydrolase_1_OS_Agrobacterium_fabrum__strain_C58_/_ATCC_33970__OX_176299_GN_folE_PE_3_SV_
AlveolataEP00333Tetrahymenathermophila_67467_GCH1_VIBCH_GTP_cyclohydrolase_1_OS_Vibrio_cholerae_serotype_O1__strain_ATCC_39315_/_El_Tor_Inaba_N16961__OX_243277_GN_folE_PE_3_SV_
MetazoaCnidariaStylophorapistillata_3664939_SYH_GRAFK_Histidine__tRNA_ligase_OS_Gramella_forsetii__strain_KT0803__OX_411154_GN_hisS_PE_3_SV_
BreviataEP00033Pygsuiabiforma_5528715_GCH1_CLOD6_GTP_cyclohydrolase_1_OS_Clostridioides_difficile__strain_630__OX_272563_GN_folE_PE_3_SV_
RhizariaGlobobuliminaspturgidaauriculata_22464267_GCH1_BACAH_GTP_cyclohydrolase_1_OS_Bacillus_thuringiensis__strain_Al_Hakam__OX_412694_GN_folE_PE_3_SV_
RhizariaGlobobuliminaspturgidaauriculata_22276018_GCH1_RAT_GTP_cyclohydrolase_1_OS_Rattus_norvegicus_OX_10116_GN_Gch1_PE_1_SV_
RhizariaGlobobuliminaspturgidaauriculata_22330386_GCH1_RAT_GTP_cyclohydrolase_1_OS_Rattus_norvegicus_OX_10116_GN_Gch1_PE_1_SV_
RhizariaGlobobuliminaspturgidaauriculata_22344260_GCH1_CHRSD_GTP_cyclohydrolase_1_OS_Chromohalobacter_salexigens__strain_DSM_3043_/_ATCC_BAA_138_/_NCIMB_13768__OX_290398_GN_folE_PE_3_SV_
CentroplasthelidaEP00300ChoanocystisspHF7_5595894_GCH1_CHICK_GTP_cyclohydrolase_1_OS_Gallus_gallus_OX_9031_GN_GCH1_PE_2_SV_
CollodictyonidaeEP00002Diphylleiarotans_11635473_GCH1_OSTOS_GTP_cyclohydrolase_1_OS_Ostertagia_ostertagi_OX_6317_GN_gch_PE_2_SV_
AncoracystaEP00164Ancoracystatwista_5352738_GCH1_CHRSD_GTP_cyclohydrolase_1_OS_Chromohalobacter_salexigens__strain_DSM_3043_/_ATCC_BAA_138_/_NCIMB_13768__OX_290398_GN_folE_PE_3_SV_
CentroplasthelidaEP00300ChoanocystisspHF7_5618491_GCH12_PSEPK_GTP_cyclohydrolase_1_2_OS_Pseudomonas_putida__strain_ATCC_47054_/_DSM_6125_/_NCIMB_11950_/_KT2440__OX_160488_GN_folE2_PE_3_SV_
RhodophytaEP00189Rhodosorusmarinus_23086858_GCH1_SYNY3_GTP_cyclohydrolase_1_OS_Synechocystis_sp___strain_PCC_6803_/_Kazusa__OX_1111708_GN_folE_PE_3_SV_
RhodophytaEP00189Rhodosorusmarinus_23093060_GCH1_SYNY3_GTP_cyclohydrolase_1_OS_Synechocystis_sp___strain_PCC_6803_/_Kazusa__OX_1111708_GN_folE_PE_3_SV_
RhodophytaEP00167Galdieriasulphuraria_22836716_GCH1_SYNY3_GTP_cyclohydrolase_1_OS_Synechocystis_sp___strain_PCC_6803_/_Kazusa__OX_1111708_GN_folE_PE_3_SV_
GlaucophytaEP00741Cyanophoraparadoxa_13513657_GCH1_SYNE7_GTP_cyclohydrolase_1_OS_Synechococcus_elongatus__strain_PCC_7942_/_FACHB_805__OX_1140_GN_folE_PE_3_SV_
GlaucophytaEP00741Cyanophoraparadoxa_13529369_GCH1_SYNY3_GTP_cyclohydrolase_1_OS_Synechocystis_sp___strain_PCC_6803_/_Kazusa__OX_1111708_GN_folE_PE_3_SV_
TelonemiaP00656Telonemasubtile_26545492_GCH1_NOSP7_GTP_cyclohydrolase_1_OS_Nostoc_punctiforme__strain_ATCC_29133_/_PCC_73102__OX_63737_GN_folE_PE_3_SV_
MetazoaCnidariaPolypodiumhydriforme_90549_GCH1_OSTOS_GTP_cyclohydrolase_1_OS_Ostertagia_ostertagi_OX_6317_GN_gch_PE_2_SV_
MetazoaCnidariaPolypodiumhydriforme_90554_GCH1_SYNY3_GTP_cyclohydrolase_1_OS_Synechocystis_sp___strain_PCC_6803_/_Kazusa__OX_1111708_GN_folE_PE_3_SV_
ChoanoflagellataEP00039Didymoecacostata_10871464_GCH1_NEUCR_GTP_cyclohydrolase_1_OS_Neurospora_crassa__strain_ATCC_24698_/_74_OR23_1A_/_CBS_708_71_/_DSM_1257_/_FGSC_987__OX_367110_GN_gch_1_PE_2_SV_
ChoanoflagellataEP00039Didymoecacostata_10872331_GCH1_CHICK_GTP_cyclohydrolase_1_OS_Gallus_gallus_OX_9031_GN_GCH1_PE_2_SV_
ChoanoflagellataEP00037Helgoecanana_10798093_GCH1_OSTOS_GTP_cyclohydrolase_1_OS_Ostertagia_ostertagi_OX_6317_GN_gch_PE_2_SV_
ChoanoflagellataSalpingoecakvevrii_11197061_GCH1_RAT_GTP_cyclohydrolase_1_OS_Rattus_norvegicus_OX_10116_GN_Gch1_PE_1_SV_
RotosphaeridaFonticulaalbaUP000030693_23193331_GCH1_YEAST_GTP_cyclohydrolase_1_OS_Saccharomyces_cerevisiae__strain_ATCC_204508_/_S288c__OX_559292_GN_FOL2_PE_1_SV_
AmoebozoaEP00013Stygamoebaregulata_5072841_GCH1_OSTOS_GTP_cyclohydrolase_1_OS_Ostertagia_ostertagi_OX_6317_GN_gch_PE_2_SV_
AmoebozoaEP00029Vermamoebavermiformis_5286782_GCH1_OSTOS_GTP_cyclohydrolase_1_OS_Ostertagia_ostertagi_OX_6317_GN_gch_PE_2_SV_
CollodictyonidaeEP00002Diphylleiarotans_11663641_GCH1_OSTOS_GTP_cyclohydrolase_1_OS_Ostertagia_ostertagi_OX_6317_GN_gch_PE_2_SV_
RigifilidaEP00004Rigifilaramosa_23118611_GCH1_OSTOS_GTP_cyclohydrolase_1_OS_Ostertagia_ostertagi_OX_6317_GN_gch_PE_2_SV_
0.34

Supplementary Figure 4: Analysis of AADCs. A) TBE tree with TBE score supports. B) Maximum likelihood tree with ultrafast bootstrap proportion supports. C) TBE tree with TBE score supports without rogue sequences. D) Maximum likelihood tree with ultrafast bootstrap proportion supports without rogue sequences. E) Maximum likelihood tree including all eukaryotic sequences with ultrafast bootstrap proportion supports. F) Reconciliation using sponge-first hypothesis. G) Reconciliation without rogue sequences using sponge first hypothesis. H) Reconciliation using ctenophore-first hypothesis. I) Reconciliation without rogue sequences using ctenophore-first hypothesis. J) Reconciliation of the UFB tree including all eukaryotic sequences. Nodes in reconciled trees indicated as speciation events (S) or duplication events (D). All trees shown as rooted, non-reconciled trees rooted arbitrarily for visualisation. AADC = aromatic amine decarboxylase, TBE = transfer bootstrap expectation, UFB = ultrafast bootstrap. Branches colour coded by sequence annotation; blue = dopa decarboxylase; green = histidine decarboxylase; orange = tyrosine decarboxylase; and black = others and non-opithokont sequences. Source Data are available at [https://figshare.le.ac.uk/articles/dataset/Monoamine_Neuromodulation_is_a_Bilaterian_Innovation_Results/20391477]; in Results/GeneTrees labelled OG_AADC_TBE.tbe.tree and OG_AADC_UFB.treefile; in Results/RogueTaxaAnalysis/GeneTreeReanalysis labelled OG_AADC_Rogue_TBE.tbe.tree and OG_AADC_Rogue_UFB.treefile; in Results/GeneTrees/Eukaryotes_GeneTrees labelled OG_AADC_EUKIQ.treefile; in Results/Reconciliation/SpongeFirst labelled OG_AADC and OG_AADC_Rogue; in Results/Reconciliation/CtenophoreFirst labelled OG_AADC and OG_AADC_Rogue; and in Results/Reconciliation/Eukaryote labelled OG_AADC.

4A.

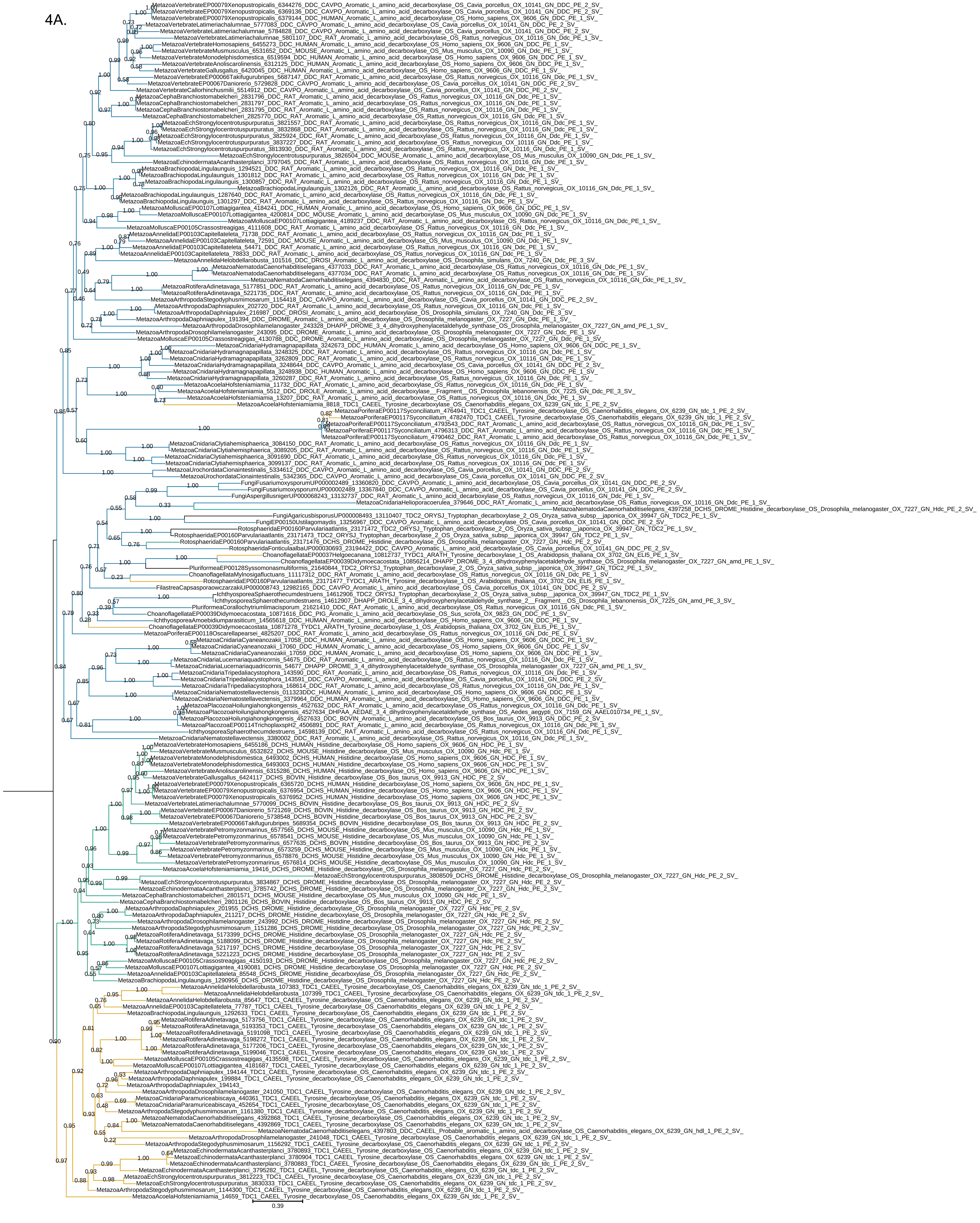

4B.

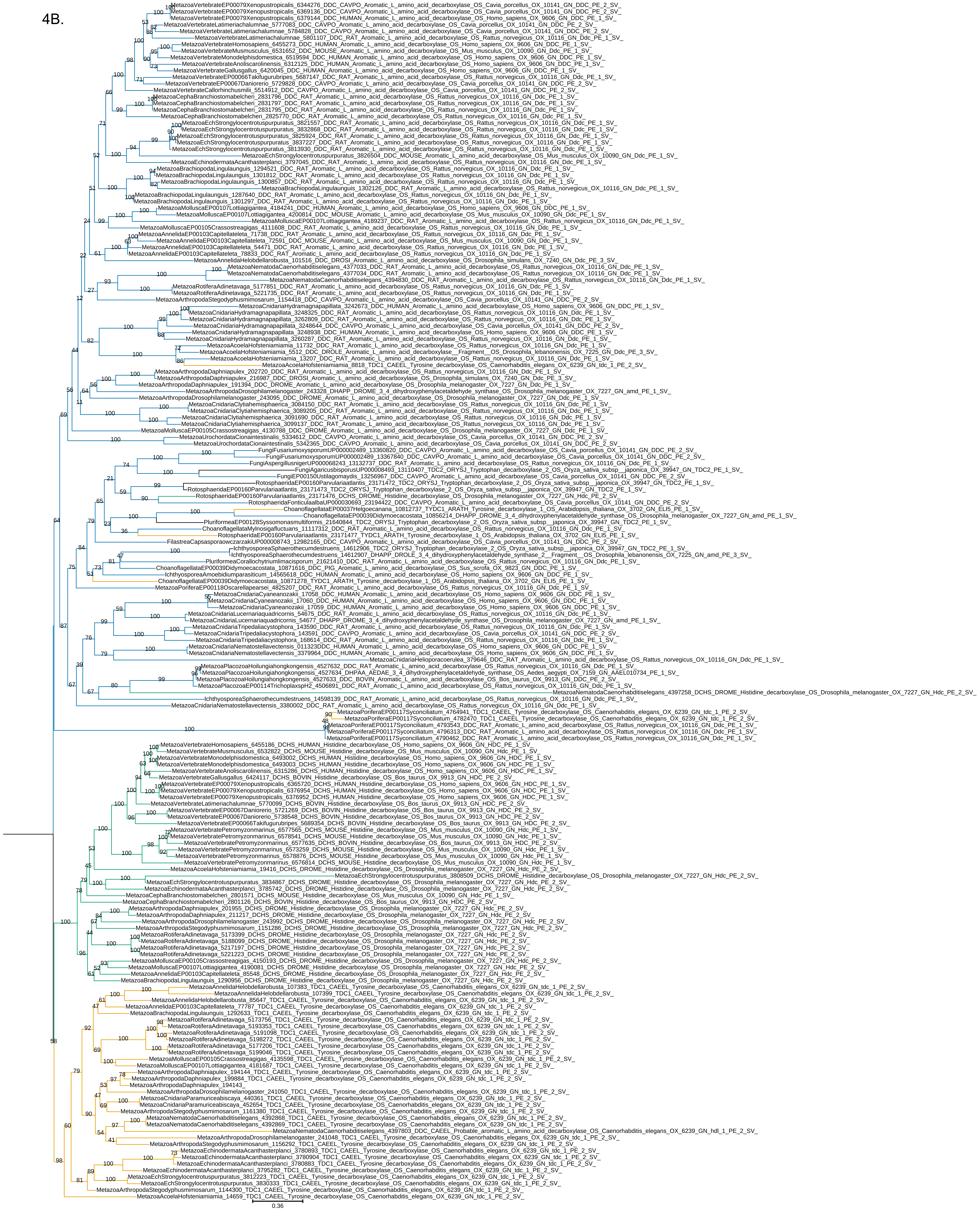

4C.

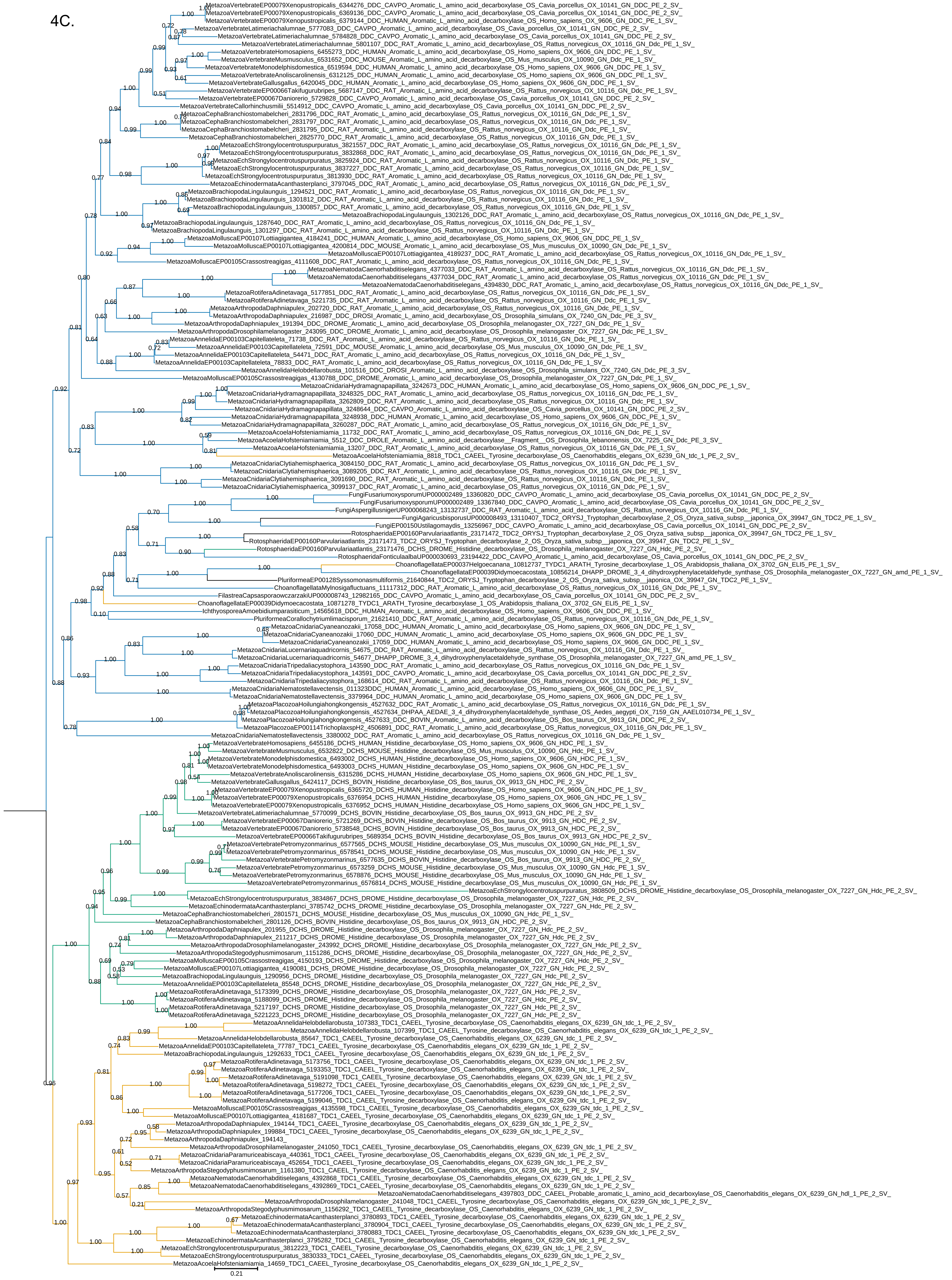

4D.

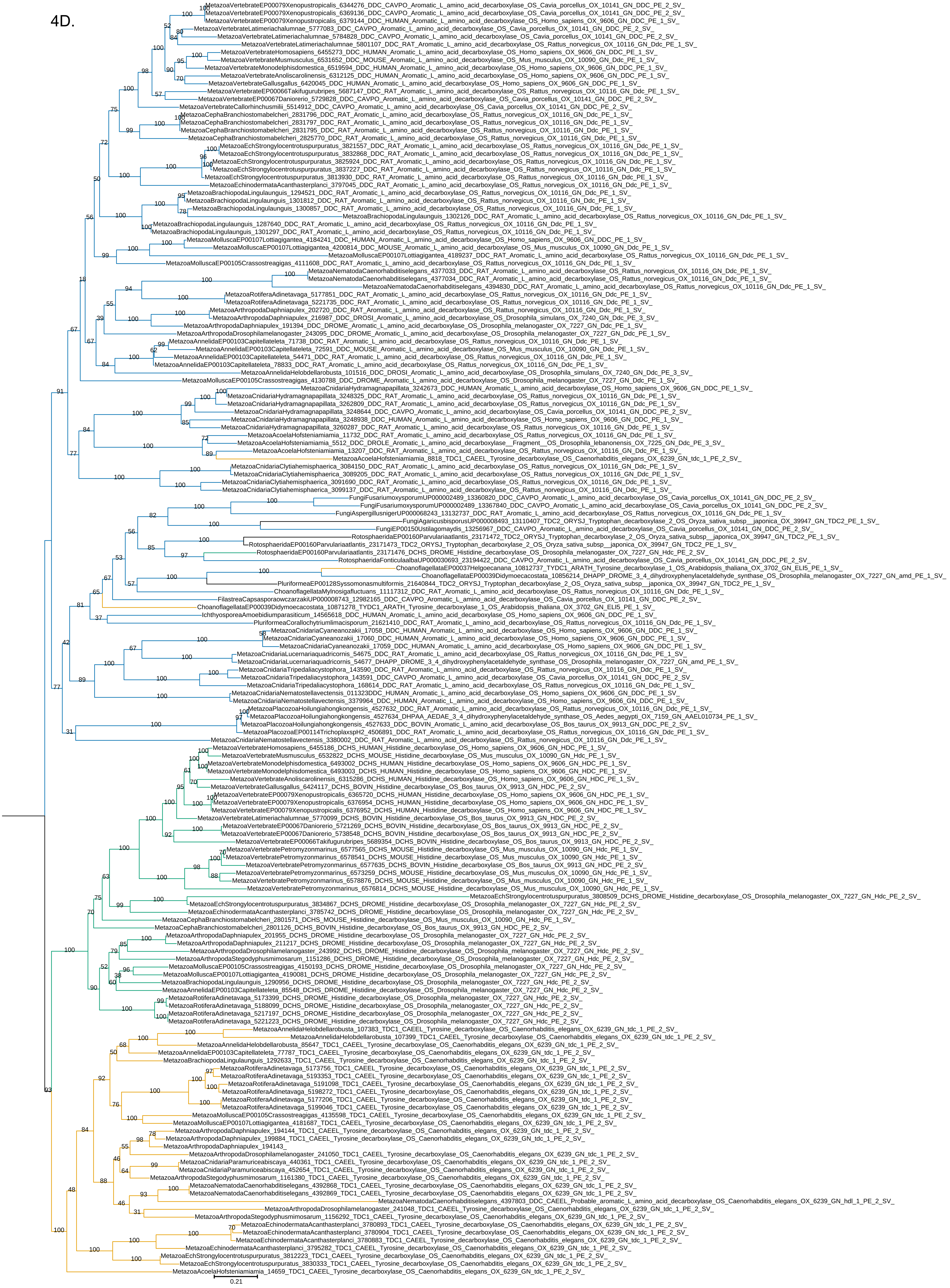

MetazoaVertebrateEP00079Xenopustropicalis_6344276_DDC_CAVPO_Aromatic_L_amino_acid_decarboxylase_OS_Cavia_porcellus_OX_10141_GN_DDC_PE_2_SV_
MetazoaVertebrateEP00079Xenopustropicalis_6369136_DDC_CAVPO_Aromatic_L_amino_acid_decarboxylase_OS_Cavia_porcellus_OX_10141_GN_DDC_PE_2_SV_
MetazoaVertebrateEP00079Xenopustropicalis_6379144_DDC_HUMAN_Aromatic_L_amino_acid_decarboxylase_OS_Homo_sapiens_OX_9606_GN_DDC_PE_1_SV_
MetazoaVertebrateLatimeriachalumnae_5777083_DDC_CAVPO_Aromatic_L_amino_acid_decarboxylase_OS_Cavia_porcellus_OX_10141_GN_DDC_PE_2_SV_
MetazoaVertebrateLatimeriachalumnae_5784828_DDC_CAVPO_Aromatic_L_amino_acid_decarboxylase_OS_Cavia_porcellus_OX_10141_GN_DDC_PE_2_SV_
MetazoaVertebrateLatimeriachalumnae_5801107_DDC_RAT_Aromatic_L_amino_acid_decarboxylase_OS_Rattus_norvegicus_OX_10116_GN_Ddc_PE_1_SV_
MetazoaVertebrateHomosapiens_6455273_DDC_HUMAN_Aromatic_L_amino_acid_decarboxylase_OS_Homo_sapiens_OX_9606_GN_DDC_PE_1_SV_
MetazoaVertebrateMusmusculus_6531652_DDC_MOUSE_Aromatic_L_amino_acid_decarboxylase_OS_Mus_musculus_OX_10090_GN_Ddc_PE_1_SV_
MetazoaVertebrateMonodelphisdomestica_6519594_DDC_HUMAN_Aromatic_L_amino_acid_decarboxylase_OS_Homo_sapiens_OX_9606_GN_DDC_PE_1_SV_
MetazoaVertebrateAnoliscarolinensis_6312125_DDC_HUMAN_Aromatic_L_amino_acid_decarboxylase_OS_Homo_sapiens_OX_9606_GN_DDC_PE_1_SV_
MetazoaVertebrateGallusgallus_6420045_DDC_HUMAN_Aromatic_L_amino_acid_decarboxylase_OS_Homo_sapiens_OX_9606_GN_DDC_PE_1_SV_
MetazoaVertebrateEP00066Takifugurubripes_5687147_DDC_RAT_Aromatic_L_amino_acid_decarboxylase_OS_Rattus_norvegicus_OX_10116_GN_Ddc_PE_1_SV_
MetazoaVertebrateEP00067Daniorerio_5729828_DDC_CAVPO_Aromatic_L_amino_acid_decarboxylase_OS_Cavia_porcellus_OX_10141_GN_DDC_PE_2_SV_
MetazoaVertebrateCallorhinchusmilii_5514912_DDC_CAVPO_Aromatic_L_amino_acid_decarboxylase_OS_Cavia_porcellus_OX_10141_GN_DDC_PE_2_SV_
MetazoaCephaBranchiostomabelcheri_2831796_DDC_RAT_Aromatic_L_amino_acid_decarboxylase_OS_Rattus_norvegicus_OX_10116_GN_Ddc_PE_1_SV_
MetazoaCephaBranchiostomabelcheri_2831797_DDC_RAT_Aromatic_L_amino_acid_decarboxylase_OS_Rattus_norvegicus_OX_10116_GN_Ddc_PE_1_SV_
MetazoaCephaBranchiostomabelcheri_2831795_DDC_RAT_Aromatic_L_amino_acid_decarboxylase_OS_Rattus_norvegicus_OX_10116_GN_Ddc_PE_1_SV_
MetazoaCephaBranchiostomabelcheri_2825770_DDC_RAT_Aromatic_L_amino_acid_decarboxylase_OS_Rattus_norvegicus_OX_10116_GN_Ddc_PE_1_SV_
MetazoaEchStrongylocentrotuspurpuratus_3821557_DDC_RAT_Aromatic_L_amino_acid_decarboxylase_OS_Rattus_norvegicus_OX_10116_GN_Ddc_PE_1_SV_
MetazoaEchStrongylocentrotuspurpuratus_3832868_DDC_RAT_Aromatic_L_amino_acid_decarboxylase_OS_Rattus_norvegicus_OX_10116_GN_Ddc_PE_1_SV_
MetazoaEchStrongylocentrotuspurpuratus_3825924_DDC_RAT_Aromatic_L_amino_acid_decarboxylase_OS_Rattus_norvegicus_OX_10116_GN_Ddc_PE_1_SV_
MetazoaEchStrongylocentrotuspurpuratus_3837227_DDC_RAT_Aromatic_L_amino_acid_decarboxylase_OS_Rattus_norvegicus_OX_10116_GN_Ddc_PE_1_SV_
MetazoaEchStrongylocentrotuspurpuratus_3813930_DDC_RAT_Aromatic_L_amino_acid_decarboxylase_OS_Rattus_norvegicus_OX_10116_GN_Ddc_PE_1_SV_
MetazoaEchinodermataAcanthasterplanci_3797045_DDC_RAT_Aromatic_L_amino_acid_decarboxylase_OS_Rattus_norvegicus_OX_10116_GN_Ddc_PE_1_SV_
MetazoaBrachiopodaLingulaunguis_1294521_DDC_RAT_Aromatic_L_amino_acid_decarboxylase_OS_Rattus_norvegicus_OX_10116_GN_Ddc_PE_1_SV_
MetazoaBrachiopodaLingulaunguis_1301812_DDC_RAT_Aromatic_L_amino_acid_decarboxylase_OS_Rattus_norvegicus_OX_10116_GN_Ddc_PE_1_SV_
MetazoaBrachiopodaLingulaunguis_1300857_DDC_RAT_Aromatic_L_amino_acid_decarboxylase_OS_Rattus_norvegicus_OX_10116_GN_Ddc_PE_1_SV_
MetazoaBrachiopodaLingulaunguis_1302126_DDC_RAT_Aromatic_L_amino_acid_decarboxylase_OS_Rattus_norvegicus_OX_10116_GN_Ddc_PE_1_SV_
MetazoaBrachiopodaLingulaunguis_1287640_DDC_RAT_Aromatic_L_amino_acid_decarboxylase_OS_Rattus_norvegicus_OX_10116_GN_Ddc_PE_1_SV_
MetazoaBrachiopodaLingulaunguis_1301297_DDC_RAT_Aromatic_L_amino_acid_decarboxylase_OS_Rattus_norvegicus_OX_10116_GN_Ddc_PE_1_SV_
MetazoaMolluscaEP00107Lottiagigantea_4184241_DDC_HUMAN_Aromatic_L_amino_acid_decarboxylase_OS_Homo_sapiens_OX_9606_GN_DDC_PE_1_SV_
MetazoaMolluscaEP00107Lottiagigantea_4200814_DDC_MOUSE_Aromatic_L_amino_acid_decarboxylase_OS_Mus_musculus_OX_10090_GN_Ddc_PE_1_SV_
MetazoaMolluscaEP00107Lottiagigantea_4189237_DDC_RAT_Aromatic_L_amino_acid_decarboxylase_OS_Rattus_norvegicus_OX_10116_GN_Ddc_PE_1_SV_
MetazoaMolluscaEP00105Crassostreagigas_4111608_DDC_RAT_Aromatic_L_amino_acid_decarboxylase_OS_Rattus_norvegicus_OX_10116_GN_Ddc_PE_1_SV_
MetazoaNematodaCaenorhabditiselegans_4377033_DDC_RAT_Aromatic_L_amino_acid_decarboxylase_OS_Rattus_norvegicus_OX_10116_GN_Ddc_PE_1_SV_
MetazoaNematodaCaenorhabditiselegans_4377034_DDC_RAT_Aromatic_L_amino_acid_decarboxylase_OS_Rattus_norvegicus_OX_10116_GN_Ddc_PE_1_SV_
MetazoaNematodaCaenorhabditiselegans_4394830_DDC_RAT_Aromatic_L_amino_acid_decarboxylase_OS_Rattus_norvegicus_OX_10116_GN_Ddc_PE_1_SV_
MetazoaRotiferaAdinetavaga_5177851_DDC_RAT_Aromatic_L_amino_acid_decarboxylase_OS_Rattus_norvegicus_OX_10116_GN_Ddc_PE_1_SV_
MetazoaRotiferaAdinetavaga_5221735_DDC_RAT_Aromatic_L_amino_acid_decarboxylase_OS_Rattus_norvegicus_OX_10116_GN_Ddc_PE_1_SV_
MetazoaArthropodaDaphniapulex_202720_DDC_RAT_Aromatic_L_amino_acid_decarboxylase_OS_Rattus_norvegicus_OX_10116_GN_Ddc_PE_1_SV_
MetazoaArthropodaDaphniapulex_216987_DDC_DROSI_Aromatic_L_amino_acid_decarboxylase_OS_Drosophila_simulans_OX_7240_GN_Ddc_PE_3_SV_
MetazoaArthropodaDaphniapulex_191394_DDC_DROME_Aromatic_L_amino_acid_decarboxylase_OS_Drosophila_melanogaster_OX_7227_GN_Ddc_PE_1_SV_
MetazoaArthropodaDrosophilamelanogaster_243095_DDC_DROME_Aromatic_L_amino_acid_decarboxylase_OS_Drosophila_melanogaster_OX_7227_GN_Ddc_PE_1_SV_
MetazoaAnnelidaEP00103Capitellateleta_71738_DDC_RAT_Aromatic_L_amino_acid_decarboxylase_OS_Rattus_norvegicus_OX_10116_GN_Ddc_PE_1_SV_
MetazoaAnnelidaEP00103Capitellateleta_72591_DDC_MOUSE_Aromatic_L_amino_acid_decarboxylase_OS_Mus_musculus_OX_10090_GN_Ddc_PE_1_SV_
MetazoaAnnelidaEP00103Capitellateleta_54471_DDC_RAT_Aromatic_L_amino_acid_decarboxylase_OS_Rattus_norvegicus_OX_10116_GN_Ddc_PE_1_SV_
MetazoaAnnelidaEP00103Capitellateleta_78833_DDC_RAT_Aromatic_L_amino_acid_decarboxylase_OS_Rattus_norvegicus_OX_10116_GN_Ddc_PE_1_SV_
MetazoaAnnelidaHelobdellarobusta_101516_DDC_DROSI_Aromatic_L_amino_acid_decarboxylase_OS_Drosophila_simulans_OX_7240_GN_Ddc_PE_3_SV_
MetazoaMolluscaEP00105Crassostreagigas_4130788_DDC_DROME_Aromatic_L_amino_acid_decarboxylase_OS_Drosophila_melanogaster_OX_7227_GN_Ddc_PE_1_SV_
MetazoaCnidariaHydramagnapapillata_3242673_DDC_HUMAN_Aromatic_L_amino_acid_decarboxylase_OS_Homo_sapiens_OX_9606_GN_DDC_PE_1_SV_
MetazoaCnidariaHydramagnapapillata_3248325_DDC_RAT_Aromatic_L_amino_acid_decarboxylase_OS_Rattus_norvegicus_OX_10116_GN_Ddc_PE_1_SV_
MetazoaCnidariaHydramagnapapillata_3262809_DDC_RAT_Aromatic_L_amino_acid_decarboxylase_OS_Rattus_norvegicus_OX_10116_GN_Ddc_PE_1_SV_
MetazoaCnidariaHydramagnapapillata_3248644_DDC_CAVPO_Aromatic_L_amino_acid_decarboxylase_OS_Cavia_porcellus_OX_10141_GN_DDC_PE_2_SV_
MetazoaCnidariaHydramagnapapillata_3248938_DDC_HUMAN_Aromatic_L_amino_acid_decarboxylase_OS_Homo_sapiens_OX_9606_GN_DDC_PE_1_SV_
MetazoaCnidariaHydramagnapapillata_3260287_DDC_RAT_Aromatic_L_amino_acid_decarboxylase_OS_Rattus_norvegicus_OX_10116_GN_Ddc_PE_1_SV_
MetazoaAcoelaHofsteniamiamia_11732_DDC_RAT_Aromatic_L_amino_acid_decarboxylase_OS_Rattus_norvegicus_OX_10116_GN_Ddc_PE_1_SV_
MetazoaAcoelaHofsteniamiamia_5512_DDC_DROLE_Aromatic_L_amino_acid_decarboxylase__Fragment__OS_Drosophila_lebanonensis_OX_7225_GN_Ddc_PE_3_SV_
MetazoaAcoelaHofsteniamiamia_13207_DDC_RAT_Aromatic_L_amino_acid_decarboxylase_OS_Rattus_norvegicus_OX_10116_GN_Ddc_PE_1_SV_
MetazoaAcoelaHofsteniamiamia_8818_TDC1_CAEEL_Tyrosine_decarboxylase_OS_Caenorhabditis_elegans_OX_6239_GN_tdc_1_PE_2_SV_
MetazoaCnidariaClytiahemisphaerica_3084150_DDC_RAT_Aromatic_L_amino_acid_decarboxylase_OS_Rattus_norvegicus_OX_10116_GN_Ddc_PE_1_SV_
MetazoaCnidariaClytiahemisphaerica_3089205_DDC_RAT_Aromatic_L_amino_acid_decarboxylase_OS_Rattus_norvegicus_OX_10116_GN_Ddc_PE_1_SV_
MetazoaCnidariaClytiahemisphaerica_3091690_DDC_RAT_Aromatic_L_amino_acid_decarboxylase_OS_Rattus_norvegicus_OX_10116_GN_Ddc_PE_1_SV_
MetazoaCnidariaClytiahemisphaerica_3099137_DDC_RAT_Aromatic_L_amino_acid_decarboxylase_OS_Rattus_norvegicus_OX_10116_GN_Ddc_PE_1_SV_
FungiFusariumoxysporumUP000002489_13360820_DDC_CAVPO_Aromatic_L_amino_acid_decarboxylase_OS_Cavia_porcellus_OX_10141_GN_DDC_PE_2_SV_
FungiFusariumoxysporumUP000002489_13367840_DDC_CAVPO_Aromatic_L_amino_acid_decarboxylase_OS_Cavia_porcellus_OX_10141_GN_DDC_PE_2_SV_
FungiAspergillusnigerUP000068243_13132737_DDC_RAT_Aromatic_L_amino_acid_decarboxylase_OS_Rattus_norvegicus_OX_10116_GN_Ddc_PE_1_SV_
FungiAgaricusbisporusUP000008493_13110407_TDC2_ORYSJ_Tryptophan_decarboxylase_2_OS_Oryza_sativa_subsp__japonica_OX_39947_GN_TDC2_PE_1_SV_
FungiEP00150Ustilagomaydis_13256967_DDC_CAVPO_Aromatic_L_amino_acid_decarboxylase_OS_Cavia_porcellus_OX_10141_GN_DDC_PE_2_SV_
RotosphaeridaEP00160Parvulariaatlantis_23171472_TDC2_ORYSJ_Tryptophan_decarboxylase_2_OS_Oryza_sativa_subsp__japonica_OX_39947_GN_TDC2_PE_1_SV_
RotosphaeridaEP00160Parvulariaatlantis_23171473_TDC2_ORYSJ_Tryptophan_decarboxylase_2_OS_Oryza_sativa_subsp__japonica_OX_39947_GN_TDC2_PE_1_SV_
RotosphaeridaEP00160Parvulariaatlantis_23171476_DCHS_DROME_Histidine_decarboxylase_OS_Drosophila_melanogaster_OX_7227_GN_Hdc_PE_2_SV_
RotosphaeridaFonticulaalbaUP000030693_23194422_DDC_CAVPO_Aromatic_L_amino_acid_decarboxylase_OS_Cavia_porcellus_OX_10141_GN_DDC_PE_2_SV_
ChoanoflagellataEP00037Helgoecanana_10812737_TYDC1_ARATH_Tyrosine_decarboxylase_1_OS_Arabidopsis_thaliana_OX_3702_GN_ELI5_PE_1_SV_
ChoanoflagellataEP00039Didymoecacostata_10856214_DHAPP_DROME_3_4_dihydroxyphenylacetaldehyde_synthase_OS_Drosophila_melanogaster_OX_7227_GN_amd_PE_1_SV_
PluriformeaEP00128Syssomonasmultiformis_21640844_TDC2_ORYSJ_Tryptophan_decarboxylase_2_OS_Oryza_sativa_subsp__japonica_OX_39947_GN_TDC2_PE_1_SV_
ChoanoflagellataMylnosigafluctuans_11117312_DDC_RAT_Aromatic_L_amino_acid_decarboxylase_OS_Rattus_norvegicus_OX_10116_GN_Ddc_PE_1_SV_
FilastreaCapsasporaowczarzakiUP000008743_12982165_DDC_CAVPO_Aromatic_L_amino_acid_decarboxylase_OS_Cavia_porcellus_OX_10141_GN_DDC_PE_2_SV_
ChoanoflagellataEP00039Didymoecacostata_10871278_TYDC1_ARATH_Tyrosine_decarboxylase_1_OS_Arabidopsis_thaliana_OX_3702_GN_ELI5_PE_1_SV_
IchthyosporeaAmoebidiumparasiticum_14565618_DDC_HUMAN_Aromatic_L_amino_acid_decarboxylase_OS_Homo_sapiens_OX_9606_GN_DDC_PE_1_SV_
PluriformeaCorallochytriumlimacisporum_21621410_DDC_RAT_Aromatic_L_amino_acid_decarboxylase_OS_Rattus_norvegicus_OX_10116_GN_Ddc_PE_1_SV_
MetazoaCnidariaCyaneanozakii_17058_DDC_HUMAN_Aromatic_L_amino_acid_decarboxylase_OS_Homo_sapiens_OX_9606_GN_DDC_PE_1_SV_
MetazoaCnidariaCyaneanozakii_17060_DDC_HUMAN_Aromatic_L_amino_acid_decarboxylase_OS_Homo_sapiens_OX_9606_GN_DDC_PE_1_SV_
MetazoaCnidariaCyaneanozakii_17059_DDC_HUMAN_Aromatic_L_amino_acid_decarboxylase_OS_Homo_sapiens_OX_9606_GN_DDC_PE_1_SV_
MetazoaCnidariaLucernariaquadricornis_54675_DDC_RAT_Aromatic_L_amino_acid_decarboxylase_OS_Rattus_norvegicus_OX_10116_GN_Ddc_PE_1_SV_
MetazoaCnidariaLucernariaquadricornis_54677_DHAPP_DROME_3_4_dihydroxyphenylacetaldehyde_synthase_OS_Drosophila_melanogaster_OX_7227_GN_amd_PE_1_SV_
MetazoaCnidariaTripedaliacystophora_143590_DDC_RAT_Aromatic_L_amino_acid_decarboxylase_OS_Rattus_norvegicus_OX_10116_GN_Ddc_PE_1_SV_
MetazoaCnidariaTripedaliacystophora_143591_DDC_CAVPO_Aromatic_L_amino_acid_decarboxylase_OS_Cavia_porcellus_OX_10141_GN_DDC_PE_2_SV_
MetazoaCnidariaTripedaliacystophora_168614_DDC_RAT_Aromatic_L_amino_acid_decarboxylase_OS_Rattus_norvegicus_OX_10116_GN_Ddc_PE_1_SV_
MetazoaCnidariaNematostellavectensis_011323DDC_HUMAN_Aromatic_L_amino_acid_decarboxylase_OS_Homo_sapiens_OX_9606_GN_DDC_PE_1_SV_
MetazoaCnidariaNematostellavectensis_3379964_DDC_HUMAN_Aromatic_L_amino_acid_decarboxylase_OS_Homo_sapiens_OX_9606_GN_DDC_PE_1_SV_
MetazoaPlacozoaHoilungiahongkongensis_4527632_DDC_RAT_Aromatic_L_amino_acid_decarboxylase_OS_Rattus_norvegicus_OX_10116_GN_Ddc_PE_1_SV_
MetazoaPlacozoaHoilungiahongkongensis_4527634_DHPAA_AEDAE_3_4_dihydroxyphenylacetaldehyde_synthase_OS_Aedes_aegypti_OX_7159_GN_AAEL010734_PE_1_SV_
MetazoaPlacozoaHoilungiahongkongensis_4527633_DDC_BOVIN_Aromatic_L_amino_acid_decarboxylase_OS_Bos_taurus_OX_9913_GN_DDC_PE_2_SV_
MetazoaPlacozoaEP00114Trichoplaxsp.H2_4506891_DDC_RAT_Aromatic_L_amino_acid_decarboxylase_OS_Rattus_norvegicus_OX_10116_GN_Ddc_PE_1_SV_
MetazoaCnidariaNematostellavectensis_3380002_DDC_RAT_Aromatic_L_amino_acid_decarboxylase_OS_Rattus_norvegicus_OX_10116_GN_Ddc_PE_1_SV_
MetazoaVertebrateHomosapiens_6455186_DCHS_HUMAN_Histidine_decarboxylase_OS_Homo_sapiens_OX_9606_GN_HDC_PE_1_SV_
MetazoaVertebrateMusmusculus_6532822_DCHS_MOUSE_Histidine_decarboxylase_OS_Mus_musculus_OX_10090_GN_Hdc_PE_1_SV_
MetazoaVertebrateMonodelphisdomestica_6493002_DCHS_HUMAN_Histidine_decarboxylase_OS_Homo_sapiens_OX_9606_GN_HDC_PE_1_SV_
MetazoaVertebrateMonodelphisdomestica_6493003_DCHS_HUMAN_Histidine_decarboxylase_OS_Homo_sapiens_OX_9606_GN_HDC_PE_1_SV_
MetazoaVertebrateAnoliscarolinensis_6315286_DCHS_HUMAN_Histidine_decarboxylase_OS_Homo_sapiens_OX_9606_GN_HDC_PE_1_SV_
MetazoaVertebrateGallusgallus_6424117_DCHS_BOVIN_Histidine_decarboxylase_OS_Bos_taurus_OX_9913_GN_HDC_PE_2_SV_
MetazoaVertebrateEP00079Xenopustropicalis_6365720_DCHS_HUMAN_Histidine_decarboxylase_OS_Homo_sapiens_OX_9606_GN_HDC_PE_1_SV_
MetazoaVertebrateEP00079Xenopustropicalis_6376954_DCHS_HUMAN_Histidine_decarboxylase_OS_Homo_sapiens_OX_9606_GN_HDC_PE_1_SV_
MetazoaVertebrateEP00079Xenopustropicalis_6376952_DCHS_HUMAN_Histidine_decarboxylase_OS_Homo_sapiens_OX_9606_GN_HDC_PE_1_SV_
MetazoaVertebrateLatimeriachalumnae_5770099_DCHS_BOVIN_Histidine_decarboxylase_OS_Bos_taurus_OX_9913_GN_HDC_PE_2_SV_
MetazoaVertebrateEP00067Daniorerio_5721269_DCHS_BOVIN_Histidine_decarboxylase_OS_Bos_taurus_OX_9913_GN_HDC_PE_2_SV_
MetazoaVertebrateEP00067Daniorerio_5738548_DCHS_BOVIN_Histidine_decarboxylase_OS_Bos_taurus_OX_9913_GN_HDC_PE_2_SV_
MetazoaVertebrateEP00066Takifugurubripes_5689354_DCHS_BOVIN_Histidine_decarboxylase_OS_Bos_taurus_OX_9913_GN_HDC_PE_2_SV_
MetazoaVertebratePetromyzonmarinus_6577565_DCHS_MOUSE_Histidine_decarboxylase_OS_Mus_musculus_OX_10090_GN_Hdc_PE_1_SV_
MetazoaVertebratePetromyzonmarinus_6578541_DCHS_MOUSE_Histidine_decarboxylase_OS_Mus_musculus_OX_10090_GN_Hdc_PE_1_SV_
MetazoaVertebratePetromyzonmarinus_6577635_DCHS_BOVIN_Histidine_decarboxylase_OS_Bos_taurus_OX_9913_GN_HDC_PE_2_SV_
MetazoaVertebratePetromyzonmarinus_6573259_DCHS_MOUSE_Histidine_decarboxylase_OS_Mus_musculus_OX_10090_GN_Hdc_PE_1_SV_
MetazoaVertebratePetromyzonmarinus_6578876_DCHS_MOUSE_Histidine_decarboxylase_OS_Mus_musculus_OX_10090_GN_Hdc_PE_1_SV_
MetazoaVertebratePetromyzonmarinus_6576814_DCHS_MOUSE_Histidine_decarboxylase_OS_Mus_musculus_OX_10090_GN_Hdc_PE_1_SV_
MetazoaEchStrongylocentrotuspurpuratus_3808509_DCHS_DROME_Histidine_decarboxylase_OS_Drosophila_melanogaster_OX_7227_GN_Hdc_PE_2_SV_
MetazoaEchStrongylocentrotuspurpuratus_3834867_DCHS_DROME_Histidine_decarboxylase_OS_Drosophila_melanogaster_OX_7227_GN_Hdc_PE_2_SV_
MetazoaEchinodermataAcanthasterplanci_3785742_DCHS_DROME_Histidine_decarboxylase_OS_Drosophila_melanogaster_OX_7227_GN_Hdc_PE_2_SV_
MetazoaCephaBranchiostomabelcheri_2801571_DCHS_MOUSE_Histidine_decarboxylase_OS_Mus_musculus_OX_10090_GN_Hdc_PE_1_SV_
MetazoaCephaBranchiostomabelcheri_2801126_DCHS_BOVIN_Histidine_decarboxylase_OS_Bos_taurus_OX_9913_GN_HDC_PE_2_SV_
MetazoaArthropodaDaphniapulex_201955_DCHS_DROME_Histidine_decarboxylase_OS_Drosophila_melanogaster_OX_7227_GN_Hdc_PE_2_SV_
MetazoaArthropodaDaphniapulex_211217_DCHS_DROME_Histidine_decarboxylase_OS_Drosophila_melanogaster_OX_7227_GN_Hdc_PE_2_SV_
MetazoaArthropodaDrosophilamelanogaster_243992_DCHS_DROME_Histidine_decarboxylase_OS_Drosophila_melanogaster_OX_7227_GN_Hdc_PE_2_SV_
MetazoaArthropodaStegodyphusmimosarum_1151286_DCHS_DROME_Histidine_decarboxylase_OS_Drosophila_melanogaster_OX_7227_GN_Hdc_PE_2_SV_
MetazoaMolluscaEP00105Crassostreagigas_4150193_DCHS_DROME_Histidine_decarboxylase_OS_Drosophila_melanogaster_OX_7227_GN_Hdc_PE_2_SV_
MetazoaMolluscaEP00107Lottiagigantea_4190081_DCHS_DROME_Histidine_decarboxylase_OS_Drosophila_melanogaster_OX_7227_GN_Hdc_PE_2_SV_
MetazoaBrachiopodaLingulaunguis_1290956_DCHS_DROME_Histidine_decarboxylase_OS_Drosophila_melanogaster_OX_7227_GN_Hdc_PE_2_SV_
MetazoaAnnelidaEP00103Capitellateleta_85548_DCHS_DROME_Histidine_decarboxylase_OS_Drosophila_melanogaster_OX_7227_GN_Hdc_PE_2_SV_
MetazoaRotiferaAdinetavaga_5173399_DCHS_DROME_Histidine_decarboxylase_OS_Drosophila_melanogaster_OX_7227_GN_Hdc_PE_2_SV_
MetazoaRotiferaAdinetavaga_5188099_DCHS_DROME_Histidine_decarboxylase_OS_Drosophila_melanogaster_OX_7227_GN_Hdc_PE_2_SV_
MetazoaRotiferaAdinetavaga_5217197_DCHS_DROME_Histidine_decarboxylase_OS_Drosophila_melanogaster_OX_7227_GN_Hdc_PE_2_SV_
MetazoaRotiferaAdinetavaga_5221223_DCHS_DROME_Histidine_decarboxylase_OS_Drosophila_melanogaster_OX_7227_GN_Hdc_PE_2_SV_
MetazoaAnnelidaHelobdellarobusta_107383_TDC1_CAEEL_Tyrosine_decarboxylase_OS_Caenorhabditis_elegans_OX_6239_GN_tdc_1_PE_2_SV_
MetazoaAnnelidaHelobdellarobusta_107399_TDC1_CAEEL_Tyrosine_decarboxylase_OS_Caenorhabditis_elegans_OX_6239_GN_tdc_1_PE_2_SV_
MetazoaAnnelidaHelobdellarobusta_85647_TDC1_CAEEL_Tyrosine_decarboxylase_OS_Caenorhabditis_elegans_OX_6239_GN_tdc_1_PE_2_SV_
MetazoaAnnelidaEP00103Capitellateleta_77787_TDC1_CAEEL_Tyrosine_decarboxylase_OS_Caenorhabditis_elegans_OX_6239_GN_tdc_1_PE_2_SV_
MetazoaBrachiopodaLingulaunguis_1292633_TDC1_CAEEL_Tyrosine_decarboxylase_OS_Caenorhabditis_elegans_OX_6239_GN_tdc_1_PE_2_SV_
MetazoaRotiferaAdinetavaga_5173756_TDC1_CAEEL_Tyrosine_decarboxylase_OS_Caenorhabditis_elegans_OX_6239_GN_tdc_1_PE_2_SV_
MetazoaRotiferaAdinetavaga_5193353_TDC1_CAEEL_Tyrosine_decarboxylase_OS_Caenorhabditis_elegans_OX_6239_GN_tdc_1_PE_2_SV_
MetazoaRotiferaAdinetavaga_5191098_TDC1_CAEEL_Tyrosine_decarboxylase_OS_Caenorhabditis_elegans_OX_6239_GN_tdc_1_PE_2_SV_
MetazoaRotiferaAdinetavaga_5198272_TDC1_CAEEL_Tyrosine_decarboxylase_OS_Caenorhabditis_elegans_OX_6239_GN_tdc_1_PE_2_SV_
MetazoaRotiferaAdinetavaga_5177206_TDC1_CAEEL_Tyrosine_decarboxylase_OS_Caenorhabditis_elegans_OX_6239_GN_tdc_1_PE_2_SV_
MetazoaRotiferaAdinetavaga_5199046_TDC1_CAEEL_Tyrosine_decarboxylase_OS_Caenorhabditis_elegans_OX_6239_GN_tdc_1_PE_2_SV_
MetazoaMolluscaEP00105Crassostreagigas_4135598_TDC1_CAEEL_Tyrosine_decarboxylase_OS_Caenorhabditis_elegans_OX_6239_GN_tdc_1_PE_2_SV_
MetazoaMolluscaEP00107Lottiagigantea_4181687_TDC1_CAEEL_Tyrosine_decarboxylase_OS_Caenorhabditis_elegans_OX_6239_GN_tdc_1_PE_2_SV_
MetazoaArthropodaDaphniapulex_194144_TDC1_CAEEL_Tyrosine_decarboxylase_OS_Caenorhabditis_elegans_OX_6239_GN_tdc_1_PE_2_SV_
MetazoaArthropodaDaphniapulex_199884_TDC1_CAEEL_Tyrosine_decarboxylase_OS_Caenorhabditis_elegans_OX_6239_GN_tdc_1_PE_2_SV_
MetazoaArthropodaDaphniapulex_194143_
MetazoaArthropodaDrosophilamelanogaster_241050_TDC1_CAEEL_Tyrosine_decarboxylase_OS_Caenorhabditis_elegans_OX_6239_GN_tdc_1_PE_2_SV_
MetazoaCnidariaParamuriceabiscaya_440361_TDC1_CAEEL_Tyrosine_decarboxylase_OS_Caenorhabditis_elegans_OX_6239_GN_tdc_1_PE_2_SV_
MetazoaCnidariaParamuriceabiscaya_452654_TDC1_CAEEL_Tyrosine_decarboxylase_OS_Caenorhabditis_elegans_OX_6239_GN_tdc_1_PE_2_SV_
MetazoaArthropodaStegodyphusmimosarum_1161380_TDC1_CAEEL_Tyrosine_decarboxylase_OS_Caenorhabditis_elegans_OX_6239_GN_tdc_1_PE_2_SV_
MetazoaNematodaCaenorhabditiselegans_4392868_TDC1_CAEEL_Tyrosine_decarboxylase_OS_Caenorhabditis_elegans_OX_6239_GN_tdc_1_PE_2_SV_
MetazoaNematodaCaenorhabditiselegans_4392869_TDC1_CAEEL_Tyrosine_decarboxylase_OS_Caenorhabditis_elegans_OX_6239_GN_tdc_1_PE_2_SV_
MetazoaNematodaCaenorhabditiselegans_4397803_DDC_CAEEL_Probable_aromatic_L_amino_acid_decarboxylase_OS_Caenorhabditis_elegans_OX_6239_GN_hdl_1_PE_2_SV_
MetazoaArthropodaDrosophilamelanogaster_241048_TDC1_CAEEL_Tyrosine_decarboxylase_OS_Caenorhabditis_elegans_OX_6239_GN_tdc_1_PE_2_SV_
MetazoaArthropodaStegodyphusmimosarum_1156292_TDC1_CAEEL_Tyrosine_decarboxylase_OS_Caenorhabditis_elegans_OX_6239_GN_tdc_1_PE_2_SV_
MetazoaEchinodermataAcanthasterplanci_3780893_TDC1_CAEEL_Tyrosine_decarboxylase_OS_Caenorhabditis_elegans_OX_6239_GN_tdc_1_PE_2_SV_
MetazoaEchinodermataAcanthasterplanci_3780904_TDC1_CAEEL_Tyrosine_decarboxylase_OS_Caenorhabditis_elegans_OX_6239_GN_tdc_1_PE_2_SV_
MetazoaEchinodermataAcanthasterplanci_3780883_TDC1_CAEEL_Tyrosine_decarboxylase_OS_Caenorhabditis_elegans_OX_6239_GN_tdc_1_PE_2_SV_
MetazoaEchinodermataAcanthasterplanci_3795282_TDC1_CAEEL_Tyrosine_decarboxylase_OS_Caenorhabditis_elegans_OX_6239_GN_tdc_1_PE_2_SV_
MetazoaEchStrongylocentrotuspurpuratus_3812223_TDC1_CAEEL_Tyrosine_decarboxylase_OS_Caenorhabditis_elegans_OX_6239_GN_tdc_1_PE_2_SV_
MetazoaEchStrongylocentrotuspurpuratus_3830333_TDC1_CAEEL_Tyrosine_decarboxylase_OS_Caenorhabditis_elegans_OX_6239_GN_tdc_1_PE_2_SV_
MetazoaAcoelaHofsteniamiamia_14659_TDC1_CAEEL_Tyrosine_decarboxylase_OS_Caenorhabditis_elegans_OX_6239_GN_tdc_1_PE_2_SV_
0.21

4E.

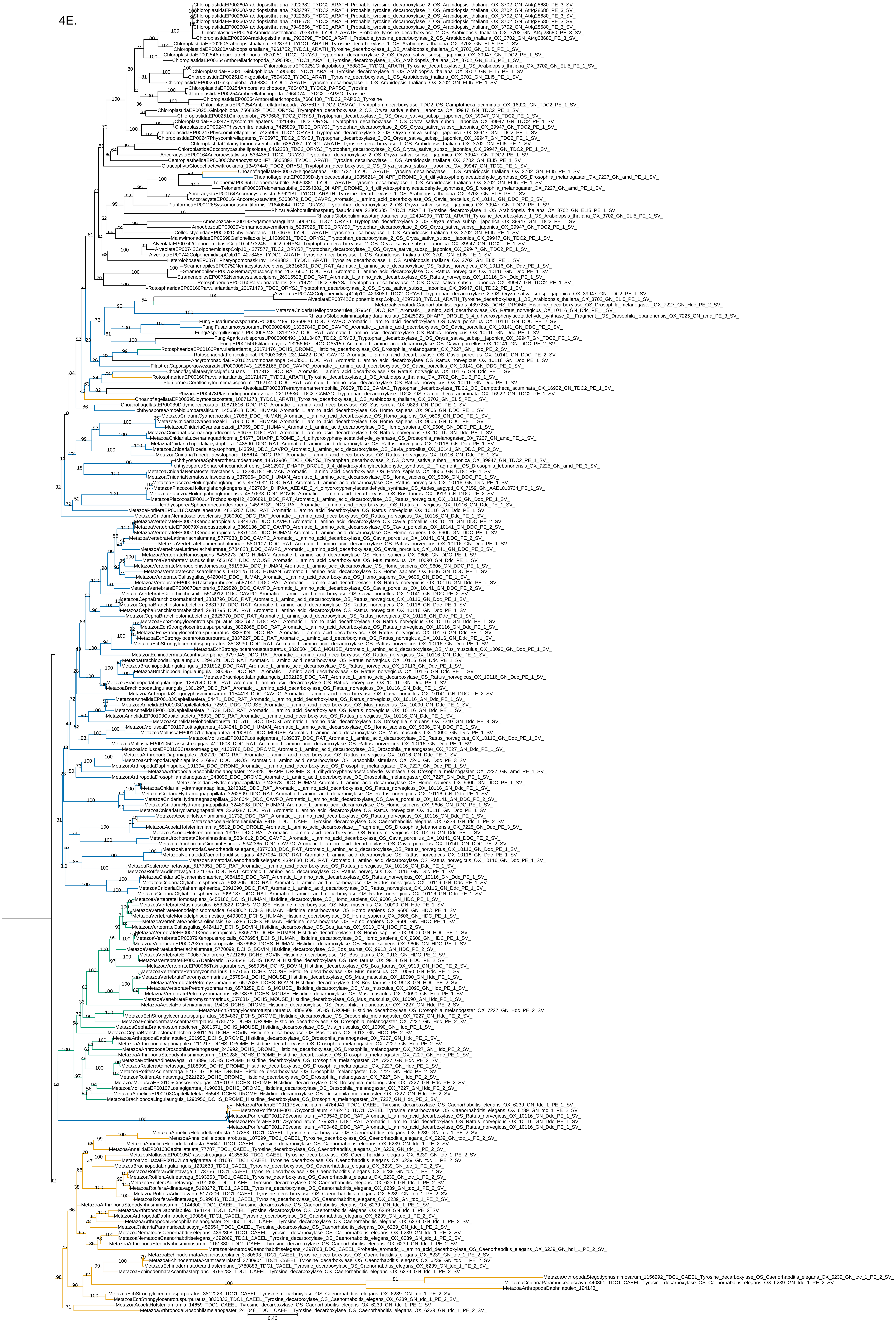

4F.

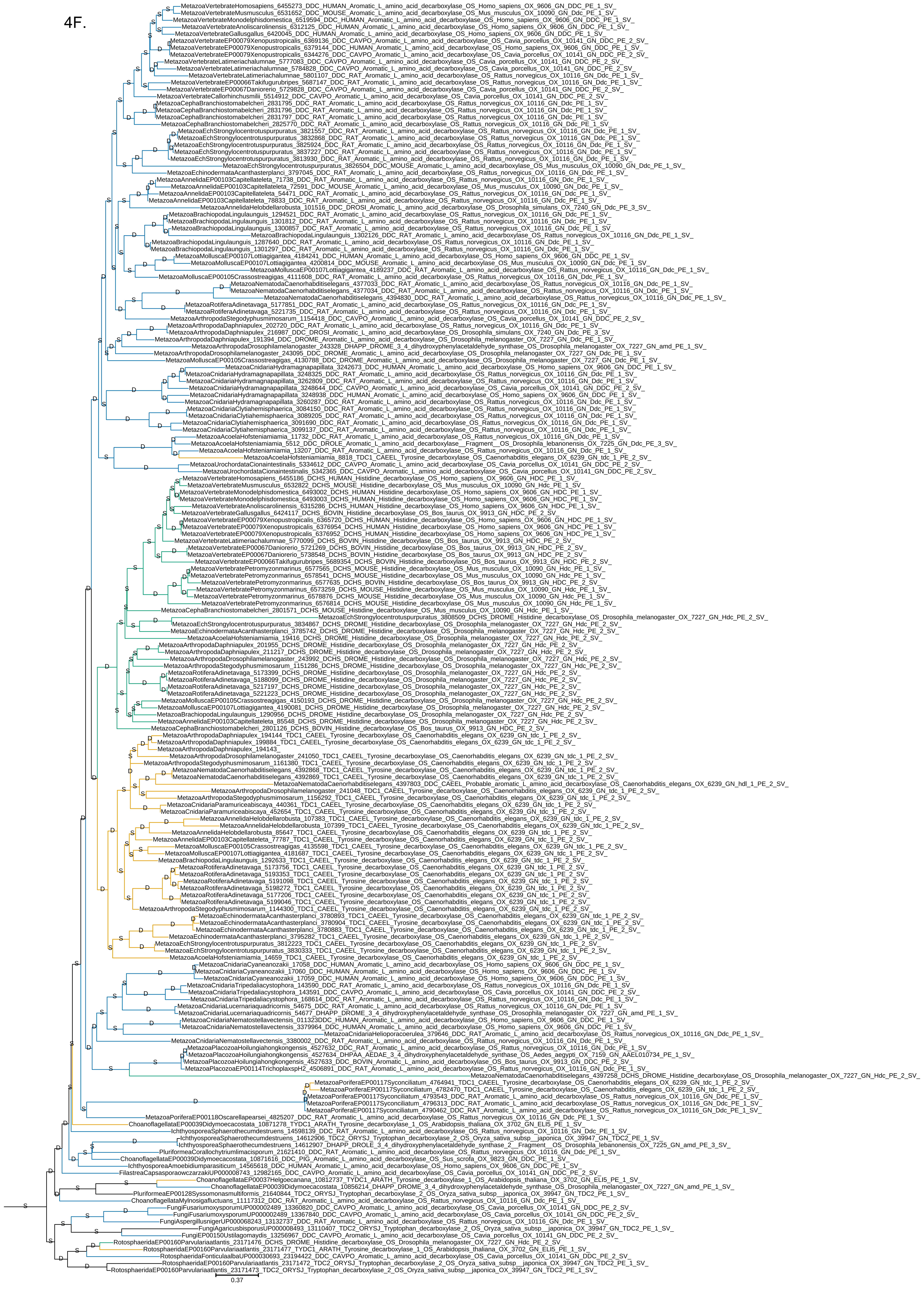

4G.

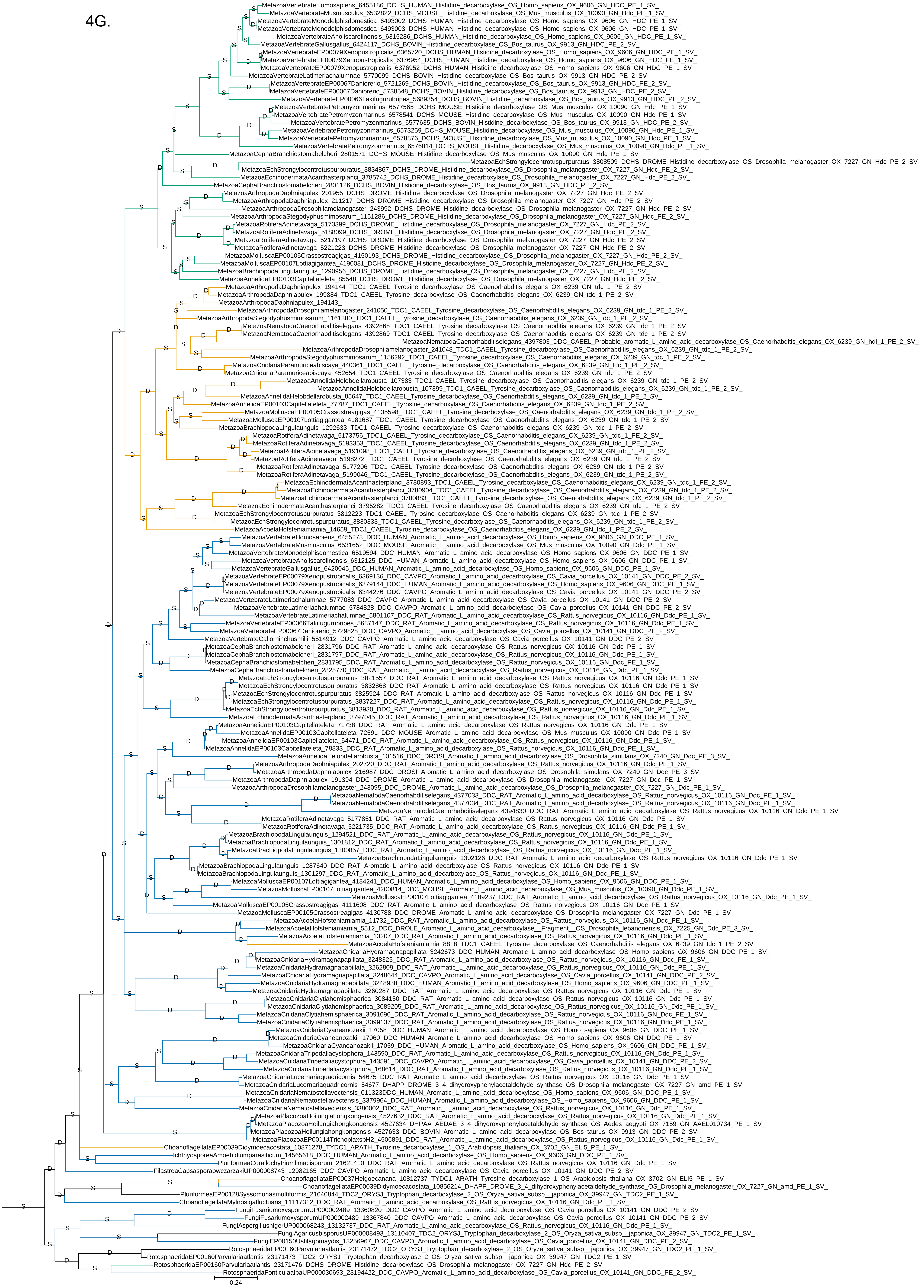
MetazoaVertebrateHomosapiens_6455186_DCHS_HUMAN_Histidine_decarboxylase_OS_Homo_sapiens_OX_9606_GN_HDC_PE_1_SV_
MetazoaVertebrateMusmusculus_6532822_DCHS_MOUSE_Histidine_decarboxylase_OS_Mus_musculus_OX_10090_GN_Hdc_PE_1_SV_
MetazoaVertebrateMonodelphisdomestica_6493002_DCHS_HUMAN_Histidine_decarboxylase_OS_Homo_sapiens_OX_9606_GN_HDC_PE_1_SV_
MetazoaVertebrateMonodelphisdomestica_6493003_DCHS_HUMAN_Histidine_decarboxylase_OS_Homo_sapiens_OX_9606_GN_HDC_PE_1_SV_
MetazoaVertebrateAnoliscarolinensis_6315286_DCHS_HUMAN_Histidine_decarboxylase_OS_Homo_sapiens_OX_9606_GN_HDC_PE_1_SV_
MetazoaVertebrateGallusgallus_6424117_DCHS_BOVIN_Histidine_decarboxylase_OS_Bos_taurus_OX_9913_GN_HDC_PE_2_SV_
MetazoaVertebrateEP00079Xenopustropicalis_6365720_DCHS_HUMAN_Histidine_decarboxylase_OS_Homo_sapiens_OX_9606_GN_HDC_PE_1_SV_
MetazoaVertebrateEP00079Xenopustropicalis_6376954_DCHS_HUMAN_Histidine_decarboxylase_OS_Homo_sapiens_OX_9606_GN_HDC_PE_1_SV_
MetazoaVertebrateEP00079Xenopustropicalis_6376952_DCHS_HUMAN_Histidine_decarboxylase_OS_Homo_sapiens_OX_9606_GN_HDC_PE_1_SV_
MetazoaVertebrateLatimeriachalumnae_5770099_DCHS_BOVIN_Histidine_decarboxylase_OS_Bos_taurus_OX_9913_GN_HDC_PE_2_SV_
MetazoaVertebrateEP00067Daniorerio_5721269_DCHS_BOVIN_Histidine_decarboxylase_OS_Bos_taurus_OX_9913_GN_HDC_PE_2_SV_
MetazoaVertebrateEP00067Daniorerio_5738548_DCHS_BOVIN_Histidine_decarboxylase_OS_Bos_taurus_OX_9913_GN_HDC_PE_2_SV_
MetazoaVertebrateEP00066Takifugurubripes_5689354_DCHS_BOVIN_Histidine_decarboxylase_OS_Bos_taurus_OX_9913_GN_HDC_PE_2_SV_
MetazoaVertebratePetromyzonmarinus_6577565_DCHS_MOUSE_Histidine_decarboxylase_OS_Mus_musculus_OX_10090_GN_Hdc_PE_1_SV_
MetazoaVertebratePetromyzonmarinus_6578541_DCHS_MOUSE_Histidine_decarboxylase_OS_Mus_musculus_OX_10090_GN_Hdc_PE_1_SV_
MetazoaVertebratePetromyzonmarinus_6577635_DCHS_BOVIN_Histidine_decarboxylase_OS_Bos_taurus_OX_9913_GN_HDC_PE_2_SV_
MetazoaVertebratePetromyzonmarinus_6573259_DCHS_MOUSE_Histidine_decarboxylase_OS_Mus_musculus_OX_10090_GN_Hdc_PE_1_SV_
MetazoaVertebratePetromyzonmarinus_6578876_DCHS_MOUSE_Histidine_decarboxylase_OS_Mus_musculus_OX_10090_GN_Hdc_PE_1_SV_
MetazoaVertebratePetromyzonmarinus_6576814_DCHS_MOUSE_Histidine_decarboxylase_OS_Mus_musculus_OX_10090_GN_Hdc_PE_1_SV_
MetazoaCephaBranchiostomabelcheri_2801571_DCHS_MOUSE_Histidine_decarboxylase_OS_Mus_musculus_OX_10090_GN_Hdc_PE_1_SV_
MetazoaEchStrongylocentrotuspurpuratus_3808509_DCHS_DROME_Histidine_decarboxylase_OS_Drosophila_melanogaster_OX_7227_GN_Hdc_PE_2_SV_
MetazoaEchStrongylocentrotuspurpuratus_3834867_DCHS_DROME_Histidine_decarboxylase_OS_Drosophila_melanogaster_OX_7227_GN_Hdc_PE_2_SV_
MetazoaEchinodermataAcanthasterplanci_3785742_DCHS_DROME_Histidine_decarboxylase_OS_Drosophila_melanogaster_OX_7227_GN_Hdc_PE_2_SV_
MetazoaCephaBranchiostomabelcheri_2801126_DCHS_BOVIN_Histidine_decarboxylase_OS_Bos_taurus_OX_9913_GN_HDC_PE_2_SV_
MetazoaArthropodaDaphniapulex_201955_DCHS_DROME_Histidine_decarboxylase_OS_Drosophila_melanogaster_OX_7227_GN_Hdc_PE_2_SV_
MetazoaArthropodaDaphniapulex_211217_DCHS_DROME_Histidine_decarboxylase_OS_Drosophila_melanogaster_OX_7227_GN_Hdc_PE_2_SV_
MetazoaArthropodaDrosophilamelanogaster_243992_DCHS_DROME_Histidine_decarboxylase_OS_Drosophila_melanogaster_OX_7227_GN_Hdc_PE_2_SV_
MetazoaArthropodaStegodyphusmimosarum_1151286_DCHS_DROME_Histidine_decarboxylase_OS_Drosophila_melanogaster_OX_7227_GN_Hdc_PE_2_SV_
MetazoaRotiferaAdinetavaga_5173399_DCHS_DROME_Histidine_decarboxylase_OS_Drosophila_melanogaster_OX_7227_GN_Hdc_PE_2_SV_
MetazoaRotiferaAdinetavaga_5188099_DCHS_DROME_Histidine_decarboxylase_OS_Drosophila_melanogaster_OX_7227_GN_Hdc_PE_2_SV_
MetazoaRotiferaAdinetavaga_5217197_DCHS_DROME_Histidine_decarboxylase_OS_Drosophila_melanogaster_OX_7227_GN_Hdc_PE_2_SV_
MetazoaRotiferaAdinetavaga_5221223_DCHS_DROME_Histidine_decarboxylase_OS_Drosophila_melanogaster_OX_7227_GN_Hdc_PE_2_SV_
MetazoaMolluscaEP00105Crassostreagigas_4150193_DCHS_DROME_Histidine_decarboxylase_OS_Drosophila_melanogaster_OX_7227_GN_Hdc_PE_2_SV_
MetazoaMolluscaEP00107Lottiagigantea_4190081_DCHS_DROME_Histidine_decarboxylase_OS_Drosophila_melanogaster_OX_7227_GN_Hdc_PE_2_SV_
MetazoaBrachiopodaLingulaunguis_1290956_DCHS_DROME_Histidine_decarboxylase_OS_Drosophila_melanogaster_OX_7227_GN_Hdc_PE_2_SV_
MetazoaAnnelidaEP00103Capitellateleta_85548_DCHS_DROME_Histidine_decarboxylase_OS_Drosophila_melanogaster_OX_7227_GN_Hdc_PE_2_SV_
MetazoaArthropodaDaphniapulex_194144_TDC1_CAEEL_Tyrosine_decarboxylase_OS_Caenorhabditis_elegans_OX_6239_GN_tdc_1_PE_2_SV_
MetazoaArthropodaDaphniapulex_199884_TDC1_CAEEL_Tyrosine_decarboxylase_OS_Caenorhabditis_elegans_OX_6239_GN_tdc_1_PE_2_SV_
MetazoaArthropodaDaphniapulex_194143_
MetazoaArthropodaDrosophilamelanogaster_241050_TDC1_CAEEL_Tyrosine_decarboxylase_OS_Caenorhabditis_elegans_OX_6239_GN_tdc_1_PE_2_SV_
MetazoaArthropodaStegodyphusmimosarum_1161380_TDC1_CAEEL_Tyrosine_decarboxylase_OS_Caenorhabditis_elegans_OX_6239_GN_tdc_1_PE_2_SV_
MetazoaNematodaCaenorhabditiselegans_4392868_TDC1_CAEEL_Tyrosine_decarboxylase_OS_Caenorhabditis_elegans_OX_6239_GN_tdc_1_PE_2_SV_
MetazoaNematodaCaenorhabditiselegans_4392869_TDC1_CAEEL_Tyrosine_decarboxylase_OS_Caenorhabditis_elegans_OX_6239_GN_tdc_1_PE_2_SV_
MetazoaNematodaCaenorhabditiselegans_4397803_DDC_CAEEL_Probable_aromatic_L_amino_acid_decarboxylase_OS_Caenorhabditis_elegans_OX_6239_GN_hdl_1_PE_2_SV_
MetazoaArthropodaDrosophilamelanogaster_241048_TDC1_CAEEL_Tyrosine_decarboxylase_OS_Caenorhabditis_elegans_OX_6239_GN_tdc_1_PE_2_SV_
MetazoaArthropodaStegodyphusmimosarum_1156292_TDC1_CAEEL_Tyrosine_decarboxylase_OS_Caenorhabditis_elegans_OX_6239_GN_tdc_1_PE_2_SV_
MetazoaCnidariaParamuriceabiscaya_440361_TDC1_CAEEL_Tyrosine_decarboxylase_OS_Caenorhabditis_elegans_OX_6239_GN_tdc_1_PE_2_SV_
MetazoaCnidariaParamuriceabiscaya_452654_TDC1_CAEEL_Tyrosine_decarboxylase_OS_Caenorhabditis_elegans_OX_6239_GN_tdc_1_PE_2_SV_
MetazoaAnnelidaHelobdellarobusta_107383_TDC1_CAEEL_Tyrosine_decarboxylase_OS_Caenorhabditis_elegans_OX_6239_GN_tdc_1_PE_2_SV_
MetazoaAnnelidaHelobdellarobusta_107399_TDC1_CAEEL_Tyrosine_decarboxylase_OS_Caenorhabditis_elegans_OX_6239_GN_tdc_1_PE_2_SV_
MetazoaAnnelidaHelobdellarobusta_85647_TDC1_CAEEL_Tyrosine_decarboxylase_OS_Caenorhabditis_elegans_OX_6239_GN_tdc_1_PE_2_SV_
MetazoaAnnelidaEP00103Capitellateleta_77787_TDC1_CAEEL_Tyrosine_decarboxylase_OS_Caenorhabditis_elegans_OX_6239_GN_tdc_1_PE_2_SV_
MetazoaMolluscaEP00105Crassostreagigas_4135598_TDC1_CAEEL_Tyrosine_decarboxylase_OS_Caenorhabditis_elegans_OX_6239_GN_tdc_1_PE_2_SV_
MetazoaMolluscaEP00107Lottiagigantea_4181687_TDC1_CAEEL_Tyrosine_decarboxylase_OS_Caenorhabditis_elegans_OX_6239_GN_tdc_1_PE_2_SV_
MetazoaBrachiopodaLingulaunguis_1292633_TDC1_CAEEL_Tyrosine_decarboxylase_OS_Caenorhabditis_elegans_OX_6239_GN_tdc_1_PE_2_SV_
MetazoaRotiferaAdinetavaga_5173756_TDC1_CAEEL_Tyrosine_decarboxylase_OS_Caenorhabditis_elegans_OX_6239_GN_tdc_1_PE_2_SV_
MetazoaRotiferaAdinetavaga_5193353_TDC1_CAEEL_Tyrosine_decarboxylase_OS_Caenorhabditis_elegans_OX_6239_GN_tdc_1_PE_2_SV_
MetazoaRotiferaAdinetavaga_5191098_TDC1_CAEEL_Tyrosine_decarboxylase_OS_Caenorhabditis_elegans_OX_6239_GN_tdc_1_PE_2_SV_
MetazoaRotiferaAdinetavaga_5198272_TDC1_CAEEL_Tyrosine_decarboxylase_OS_Caenorhabditis_elegans_OX_6239_GN_tdc_1_PE_2_SV_
MetazoaRotiferaAdinetavaga_5177206_TDC1_CAEEL_Tyrosine_decarboxylase_OS_Caenorhabditis_elegans_OX_6239_GN_tdc_1_PE_2_SV_
MetazoaRotiferaAdinetavaga_5199046_TDC1_CAEEL_Tyrosine_decarboxylase_OS_Caenorhabditis_elegans_OX_6239_GN_tdc_1_PE_2_SV_
MetazoaEchinodermataAcanthasterplanci_3780893_TDC1_CAEEL_Tyrosine_decarboxylase_OS_Caenorhabditis_elegans_OX_6239_GN_tdc_1_PE_2_SV_
MetazoaEchinodermataAcanthasterplanci_3780904_TDC1_CAEEL_Tyrosine_decarboxylase_OS_Caenorhabditis_elegans_OX_6239_GN_tdc_1_PE_2_SV_
MetazoaEchinodermataAcanthasterplanci_3780883_TDC1_CAEEL_Tyrosine_decarboxylase_OS_Caenorhabditis_elegans_OX_6239_GN_tdc_1_PE_2_SV_
MetazoaEchinodermataAcanthasterplanci_3795282_TDC1_CAEEL_Tyrosine_decarboxylase_OS_Caenorhabditis_elegans_OX_6239_GN_tdc_1_PE_2_SV_
MetazoaEchStrongylocentrotuspurpuratus_3812223_TDC1_CAEEL_Tyrosine_decarboxylase_OS_Caenorhabditis_elegans_OX_6239_GN_tdc_1_PE_2_SV_
MetazoaEchStrongylocentrotuspurpuratus_3830333_TDC1_CAEEL_Tyrosine_decarboxylase_OS_Caenorhabditis_elegans_OX_6239_GN_tdc_1_PE_2_SV_
MetazoaAcoelaHofsteniamiamia_14659_TDC1_CAEEL_Tyrosine_decarboxylase_OS_Caenorhabditis_elegans_OX_6239_GN_tdc_1_PE_2_SV_
MetazoaVertebrateHomosapiens_6455273_DDC_HUMAN_Aromatic_L_amino_acid_decarboxylase_OS_Homo_sapiens_OX_9606_GN_DDC_PE_1_SV_
MetazoaVertebrateMusmusculus_6531652_DDC_MOUSE_Aromatic_L_amino_acid_decarboxylase_OS_Mus_musculus_OX_10090_GN_Ddc_PE_1_SV_
MetazoaVertebrateMonodelphisdomestica_6519594_DDC_HUMAN_Aromatic_L_amino_acid_decarboxylase_OS_Homo_sapiens_OX_9606_GN_DDC_PE_1_SV_
MetazoaVertebrateAnoliscarolinensis_6312125_DDC_HUMAN_Aromatic_L_amino_acid_decarboxylase_OS_Homo_sapiens_OX_9606_GN_DDC_PE_1_SV_
MetazoaVertebrateGallusgallus_6420045_DDC_HUMAN_Aromatic_L_amino_acid_decarboxylase_OS_Homo_sapiens_OX_9606_GN_DDC_PE_1_SV_
MetazoaVertebrateEP00079Xenopustropicalis_6369136_DDC_CAVPO_Aromatic_L_amino_acid_decarboxylase_OS_Cavia_porcellus_OX_10141_GN_DDC_PE_2_SV_
MetazoaVertebrateEP00079Xenopustropicalis_6379144_DDC_HUMAN_Aromatic_L_amino_acid_decarboxylase_OS_Homo_sapiens_OX_9606_GN_DDC_PE_1_SV_
MetazoaVertebrateEP00079Xenopustropicalis_6344276_DDC_CAVPO_Aromatic_L_amino_acid_decarboxylase_OS_Cavia_porcellus_OX_10141_GN_DDC_PE_2_SV_
MetazoaVertebrateLatimeriachalumnae_5777083_DDC_CAVPO_Aromatic_L_amino_acid_decarboxylase_OS_Cavia_porcellus_OX_10141_GN_DDC_PE_2_SV_
MetazoaVertebrateLatimeriachalumnae_5784828_DDC_CAVPO_Aromatic_L_amino_acid_decarboxylase_OS_Cavia_porcellus_OX_10141_GN_DDC_PE_2_SV_
MetazoaVertebrateLatimeriachalumnae_5801107_DDC_RAT_Aromatic_L_amino_acid_decarboxylase_OS_Rattus_norvegicus_OX_10116_GN_Ddc_PE_1_SV_
MetazoaVertebrateEP00066Takifugurubripes_5687147_DDC_RAT_Aromatic_L_amino_acid_decarboxylase_OS_Rattus_norvegicus_OX_10116_GN_Ddc_PE_1_SV_
MetazoaVertebrateEP00067Daniorerio_5729828_DDC_CAVPO_Aromatic_L_amino_acid_decarboxylase_OS_Cavia_porcellus_OX_10141_GN_DDC_PE_2_SV_
MetazoaVertebrateCallorhinchusmilii_5514912_DDC_CAVPO_Aromatic_L_amino_acid_decarboxylase_OS_Cavia_porcellus_OX_10141_GN_DDC_PE_2_SV_
MetazoaCephaBranchiostomabelcheri_2831796_DDC_RAT_Aromatic_L_amino_acid_decarboxylase_OS_Rattus_norvegicus_OX_10116_GN_Ddc_PE_1_SV_
MetazoaCephaBranchiostomabelcheri_2831797_DDC_RAT_Aromatic_L_amino_acid_decarboxylase_OS_Rattus_norvegicus_OX_10116_GN_Ddc_PE_1_SV_
MetazoaCephaBranchiostomabelcheri_2831795_DDC_RAT_Aromatic_L_amino_acid_decarboxylase_OS_Rattus_norvegicus_OX_10116_GN_Ddc_PE_1_SV_
MetazoaCephaBranchiostomabelcheri_2825770_DDC_RAT_Aromatic_L_amino_acid_decarboxylase_OS_Rattus_norvegicus_OX_10116_GN_Ddc_PE_1_SV_
MetazoaEchStrongylocentrotuspurpuratus_3821557_DDC_RAT_Aromatic_L_amino_acid_decarboxylase_OS_Rattus_norvegicus_OX_10116_GN_Ddc_PE_1_SV_
MetazoaEchStrongylocentrotuspurpuratus_3832868_DDC_RAT_Aromatic_L_amino_acid_decarboxylase_OS_Rattus_norvegicus_OX_10116_GN_Ddc_PE_1_SV_
MetazoaEchStrongylocentrotuspurpuratus_3825924_DDC_RAT_Aromatic_L_amino_acid_decarboxylase_OS_Rattus_norvegicus_OX_10116_GN_Ddc_PE_1_SV_
MetazoaEchStrongylocentrotuspurpuratus_3837227_DDC_RAT_Aromatic_L_amino_acid_decarboxylase_OS_Rattus_norvegicus_OX_10116_GN_Ddc_PE_1_SV_
MetazoaEchStrongylocentrotuspurpuratus_3813930_DDC_RAT_Aromatic_L_amino_acid_decarboxylase_OS_Rattus_norvegicus_OX_10116_GN_Ddc_PE_1_SV_
MetazoaEchinodermataAcanthasterplanci_3797045_DDC_RAT_Aromatic_L_amino_acid_decarboxylase_OS_Rattus_norvegicus_OX_10116_GN_Ddc_PE_1_SV_
MetazoaAnnelidaEP00103Capitellateleta_71738_DDC_RAT_Aromatic_L_amino_acid_decarboxylase_OS_Rattus_norvegicus_OX_10116_GN_Ddc_PE_1_SV_
MetazoaAnnelidaEP00103Capitellateleta_72591_DDC_MOUSE_Aromatic_L_amino_acid_decarboxylase_OS_Mus_musculus_OX_10090_GN_Ddc_PE_1_SV_
MetazoaAnnelidaEP00103Capitellateleta_54471_DDC_RAT_Aromatic_L_amino_acid_decarboxylase_OS_Rattus_norvegicus_OX_10116_GN_Ddc_PE_1_SV_
MetazoaAnnelidaEP00103Capitellateleta_78833_DDC_RAT_Aromatic_L_amino_acid_decarboxylase_OS_Rattus_norvegicus_OX_10116_GN_Ddc_PE_1_SV_
MetazoaAnnelidaHelobdellarobusta_101516_DDC_DROSI_Aromatic_L_amino_acid_decarboxylase_OS_Drosophila_simulans_OX_7240_GN_Ddc_PE_3_SV_
MetazoaArthropodaDaphniapulex_202720_DDC_RAT_Aromatic_L_amino_acid_decarboxylase_OS_Rattus_norvegicus_OX_10116_GN_Ddc_PE_1_SV_
MetazoaArthropodaDaphniapulex_216987_DDC_DROSI_Aromatic_L_amino_acid_decarboxylase_OS_Drosophila_simulans_OX_7240_GN_Ddc_PE_3_SV_
MetazoaArthropodaDaphniapulex_191394_DDC_DROME_Aromatic_L_amino_acid_decarboxylase_OS_Drosophila_melanogaster_OX_7227_GN_Ddc_PE_1_SV_
MetazoaArthropodaDrosophilamelanogaster_243095_DDC_DROME_Aromatic_L_amino_acid_decarboxylase_OS_Drosophila_melanogaster_OX_7227_GN_Ddc_PE_1_SV_
MetazoaNematodaCaenorhabditiselegans_4377033_DDC_RAT_Aromatic_L_amino_acid_decarboxylase_OS_Rattus_norvegicus_OX_10116_GN_Ddc_PE_1_SV_
MetazoaNematodaCaenorhabditiselegans_4377034_DDC_RAT_Aromatic_L_amino_acid_decarboxylase_OS_Rattus_norvegicus_OX_10116_GN_Ddc_PE_1_SV_
MetazoaNematodaCaenorhabditiselegans_4394830_DDC_RAT_Aromatic_L_amino_acid_decarboxylase_OS_Rattus_norvegicus_OX_10116_GN_Ddc_PE_1_SV_
MetazoaRotiferaAdinetavaga_5177851_DDC_RAT_Aromatic_L_amino_acid_decarboxylase_OS_Rattus_norvegicus_OX_10116_GN_Ddc_PE_1_SV_
MetazoaRotiferaAdinetavaga_5221735_DDC_RAT_Aromatic_L_amino_acid_decarboxylase_OS_Rattus_norvegicus_OX_10116_GN_Ddc_PE_1_SV_
MetazoaBrachiopodaLingulaunguis_1294521_DDC_RAT_Aromatic_L_amino_acid_decarboxylase_OS_Rattus_norvegicus_OX_10116_GN_Ddc_PE_1_SV_
MetazoaBrachiopodaLingulaunguis_1301812_DDC_RAT_Aromatic_L_amino_acid_decarboxylase_OS_Rattus_norvegicus_OX_10116_GN_Ddc_PE_1_SV_
MetazoaBrachiopodaLingulaunguis_1300857_DDC_RAT_Aromatic_L_amino_acid_decarboxylase_OS_Rattus_norvegicus_OX_10116_GN_Ddc_PE_1_SV_
MetazoaBrachiopodaLingulaunguis_1302126_DDC_RAT_Aromatic_L_amino_acid_decarboxylase_OS_Rattus_norvegicus_OX_10116_GN_Ddc_PE_1_SV_
MetazoaBrachiopodaLingulaunguis_1287640_DDC_RAT_Aromatic_L_amino_acid_decarboxylase_OS_Rattus_norvegicus_OX_10116_GN_Ddc_PE_1_SV_
MetazoaBrachiopodaLingulaunguis_1301297_DDC_RAT_Aromatic_L_amino_acid_decarboxylase_OS_Rattus_norvegicus_OX_10116_GN_Ddc_PE_1_SV_
MetazoaMolluscaEP00107Lottiagigantea_4184241_DDC_HUMAN_Aromatic_L_amino_acid_decarboxylase_OS_Homo_sapiens_OX_9606_GN_DDC_PE_1_SV_
MetazoaMolluscaEP00107Lottiagigantea_4200814_DDC_MOUSE_Aromatic_L_amino_acid_decarboxylase_OS_Mus_musculus_OX_10090_GN_Ddc_PE_1_SV_
MetazoaMolluscaEP00107Lottiagigantea_4189237_DDC_RAT_Aromatic_L_amino_acid_decarboxylase_OS_Rattus_norvegicus_OX_10116_GN_Ddc_PE_1_SV_
MetazoaMolluscaEP00105Crassostreagigas_4111608_DDC_RAT_Aromatic_L_amino_acid_decarboxylase_OS_Rattus_norvegicus_OX_10116_GN_Ddc_PE_1_SV_
MetazoaMolluscaEP00105Crassostreagigas_4130788_DDC_DROME_Aromatic_L_amino_acid_decarboxylase_OS_Drosophila_melanogaster_OX_7227_GN_Ddc_PE_1_SV_
MetazoaAcoelaHofsteniamiamia_11732_DDC_RAT_Aromatic_L_amino_acid_decarboxylase_OS_Rattus_norvegicus_OX_10116_GN_Ddc_PE_1_SV_
MetazoaAcoelaHofsteniamiamia_5512_DDC_DROLE_Aromatic_L_amino_acid_decarboxylase__Fragment__OS_Drosophila_lebanonensis_OX_7225_GN_Ddc_PE_3_SV_
MetazoaAcoelaHofsteniamiamia_13207_DDC_RAT_Aromatic_L_amino_acid_decarboxylase_OS_Rattus_norvegicus_OX_10116_GN_Ddc_PE_1_SV_
MetazoaAcoelaHofsteniamiamia_8818_TDC1_CAEEL_Tyrosine_decarboxylase_OS_Caenorhabditis_elegans_OX_6239_GN_tdc_1_PE_2_SV_
MetazoaCnidariaHydramagnapapillata_3242673_DDC_HUMAN_Aromatic_L_amino_acid_decarboxylase_OS_Homo_sapiens_OX_9606_GN_DDC_PE_1_SV_
MetazoaCnidariaHydramagnapapillata_3248325_DDC_RAT_Aromatic_L_amino_acid_decarboxylase_OS_Rattus_norvegicus_OX_10116_GN_Ddc_PE_1_SV_
MetazoaCnidariaHydramagnapapillata_3262809_DDC_RAT_Aromatic_L_amino_acid_decarboxylase_OS_Rattus_norvegicus_OX_10116_GN_Ddc_PE_1_SV_
MetazoaCnidariaHydramagnapapillata_3248644_DDC_CAVPO_Aromatic_L_amino_acid_decarboxylase_OS_Cavia_porcellus_OX_10141_GN_DDC_PE_2_SV_
MetazoaCnidariaHydramagnapapillata_3248938_DDC_HUMAN_Aromatic_L_amino_acid_decarboxylase_OS_Homo_sapiens_OX_9606_GN_DDC_PE_1_SV_
MetazoaCnidariaHydramagnapapillata_3260287_DDC_RAT_Aromatic_L_amino_acid_decarboxylase_OS_Rattus_norvegicus_OX_10116_GN_Ddc_PE_1_SV_
MetazoaCnidariaClytiahemisphaerica_3084150_DDC_RAT_Aromatic_L_amino_acid_decarboxylase_OS_Rattus_norvegicus_OX_10116_GN_Ddc_PE_1_SV_
MetazoaCnidariaClytiahemisphaerica_3089205_DDC_RAT_Aromatic_L_amino_acid_decarboxylase_OS_Rattus_norvegicus_OX_10116_GN_Ddc_PE_1_SV_
MetazoaCnidariaClytiahemisphaerica_3091690_DDC_RAT_Aromatic_L_amino_acid_decarboxylase_OS_Rattus_norvegicus_OX_10116_GN_Ddc_PE_1_SV_
MetazoaCnidariaClytiahemisphaerica_3099137_DDC_RAT_Aromatic_L_amino_acid_decarboxylase_OS_Rattus_norvegicus_OX_10116_GN_Ddc_PE_1_SV_
MetazoaCnidariaCyaneanozakii_17058_DDC_HUMAN_Aromatic_L_amino_acid_decarboxylase_OS_Homo_sapiens_OX_9606_GN_DDC_PE_1_SV_
MetazoaCnidariaCyaneanozakii_17060_DDC_HUMAN_Aromatic_L_amino_acid_decarboxylase_OS_Homo_sapiens_OX_9606_GN_DDC_PE_1_SV_
MetazoaCnidariaCyaneanozakii_17059_DDC_HUMAN_Aromatic_L_amino_acid_decarboxylase_OS_Homo_sapiens_OX_9606_GN_DDC_PE_1_SV_
MetazoaCnidariaTripedaliacystophora_143590_DDC_RAT_Aromatic_L_amino_acid_decarboxylase_OS_Rattus_norvegicus_OX_10116_GN_Ddc_PE_1_SV_
MetazoaCnidariaTripedaliacystophora_143591_DDC_CAVPO_Aromatic_L_amino_acid_decarboxylase_OS_Cavia_porcellus_OX_10141_GN_DDC_PE_2_SV_
MetazoaCnidariaTripedaliacystophora_168614_DDC_RAT_Aromatic_L_amino_acid_decarboxylase_OS_Rattus_norvegicus_OX_10116_GN_Ddc_PE_1_SV_
MetazoaCnidariaLucernariaquadricornis_54675_DDC_RAT_Aromatic_L_amino_acid_decarboxylase_OS_Rattus_norvegicus_OX_10116_GN_Ddc_PE_1_SV_
MetazoaCnidariaLucernariaquadricornis_54677_DHAPP_DROME_3_4_dihydroxyphenylacetaldehyde_synthase_OS_Drosophila_melanogaster_OX_7227_GN_amd_PE_1_SV_
MetazoaCnidariaNematostellavectensis_011323DDC_HUMAN_Aromatic_L_amino_acid_decarboxylase_OS_Homo_sapiens_OX_9606_GN_DDC_PE_1_SV_
MetazoaCnidariaNematostellavectensis_3379964_DDC_HUMAN_Aromatic_L_amino_acid_decarboxylase_OS_Homo_sapiens_OX_9606_GN_DDC_PE_1_SV_
MetazoaCnidariaNematostellavectensis_3380002_DDC_RAT_Aromatic_L_amino_acid_decarboxylase_OS_Rattus_norvegicus_OX_10116_GN_Ddc_PE_1_SV_
MetazoaPlacozoaHoilungiahongkongensis_4527632_DDC_RAT_Aromatic_L_amino_acid_decarboxylase_OS_Rattus_norvegicus_OX_10116_GN_Ddc_PE_1_SV_
MetazoaPlacozoaHoilungiahongkongensis_4527634_DHPAA_AEDAE_3_4_dihydroxyphenylacetaldehyde_synthase_OS_Aedes_aegypti_OX_7159_GN_AAEL010734_PE_1_SV_
MetazoaPlacozoaHoilungiahongkongensis_4527633_DDC_BOVIN_Aromatic_L_amino_acid_decarboxylase_OS_Bos_taurus_OX_9913_GN_DDC_PE_2_SV_
MetazoaPlacozoaEP00114TrichoplaxspH2_4506891_DDC_RAT_Aromatic_L_amino_acid_decarboxylase_OS_Rattus_norvegicus_OX_10116_GN_Ddc_PE_1_SV_
ChoanoflagellataEP00039Didymoecacostata_10871278_TYDC1_ARATH_Tyrosine_decarboxylase_1_OS_Arabidopsis_thaliana_OX_3702_GN_ELI5_PE_1_SV_
IchthyosporeaAmoebidiumparasiticum_14565618_DDC_HUMAN_Aromatic_L_amino_acid_decarboxylase_OS_Homo_sapiens_OX_9606_GN_DDC_PE_1_SV_
PluriformeaCorallochytriumlimacisporum_21621410_DDC_RAT_Aromatic_L_amino_acid_decarboxylase_OS_Rattus_norvegicus_OX_10116_GN_Ddc_PE_1_SV_
FilastreaCapsasporaowczarzakiUP000008743_12982165_DDC_CAVPO_Aromatic_L_amino_acid_decarboxylase_OS_Cavia_porcellus_OX_10141_GN_DDC_PE_2_SV_
ChoanoflagellataEP00037Helgoecanana_10812737_TYDC1_ARATH_Tyrosine_decarboxylase_1_OS_Arabidopsis_thaliana_OX_3702_GN_ELI5_PE_1_SV_
ChoanoflagellataEP00039Didymoecacostata_10856214_DHAPP_DROME_3_4_dihydroxyphenylacetaldehyde_synthase_OS_Drosophila_melanogaster_OX_7227_GN_amd_PE_1_SV_
PluriformeaEP00128Syssomonasmultiformis_21640844_TDC2_ORYSJ_Tryptophan_decarboxylase_2_OS_Oryza_sativa_subsp__japonica_OX_39947_GN_TDC2_PE_1_SV_
ChoanoflagellataMylnosigafluctuans_11117312_DDC_RAT_Aromatic_L_amino_acid_decarboxylase_OS_Rattus_norvegicus_OX_10116_GN_Ddc_PE_1_SV_
FungiFusariumoxysporumUP000002489_13360820_DDC_CAVPO_Aromatic_L_amino_acid_decarboxylase_OS_Cavia_porcellus_OX_10141_GN_DDC_PE_2_SV_
FungiFusariumoxysporumUP000002489_13367840_DDC_CAVPO_Aromatic_L_amino_acid_decarboxylase_OS_Cavia_porcellus_OX_10141_GN_DDC_PE_2_SV_
FungiAspergillusnigerUP000068243_13132737_DDC_RAT_Aromatic_L_amino_acid_decarboxylase_OS_Rattus_norvegicus_OX_10116_GN_Ddc_PE_1_SV_
FungiAgaricusbisporusUP000008493_13110407_TDC2_ORYSJ_Tryptophan_decarboxylase_2_OS_Oryza_sativa_subsp__japonica_OX_39947_GN_TDC2_PE_1_SV_
FungiEP00150Ustilagomaydis_13256967_DDC_CAVPO_Aromatic_L_amino_acid_decarboxylase_OS_Cavia_porcellus_OX_10141_GN_DDC_PE_2_SV_
RotosphaeridaEP00160Parvulariaatlantis_23171472_TDC2_ORYSJ_Tryptophan_decarboxylase_2_OS_Oryza_sativa_subsp__japonica_OX_39947_GN_TDC2_PE_1_SV_
RotosphaeridaEP00160Parvulariaatlantis_23171473_TDC2_ORYSJ_Tryptophan_decarboxylase_2_OS_Oryza_sativa_subsp__japonica_OX_39947_GN_TDC2_PE_1_SV_
RotosphaeridaEP00160Parvulariaatlantis_23171476_DCHS_DROME_Histidine_decarboxylase_OS_Drosophila_melanogaster_OX_7227_GN_Hdc_PE_2_SV_
RotosphaeridaFonticulaalbaUP000030693_23194422_DDC_CAVPO_Aromatic_L_amino_acid_decarboxylase_OS_Cavia_porcellus_OX_10141_GN_DDC_PE_2_SV_
0.24

4H.

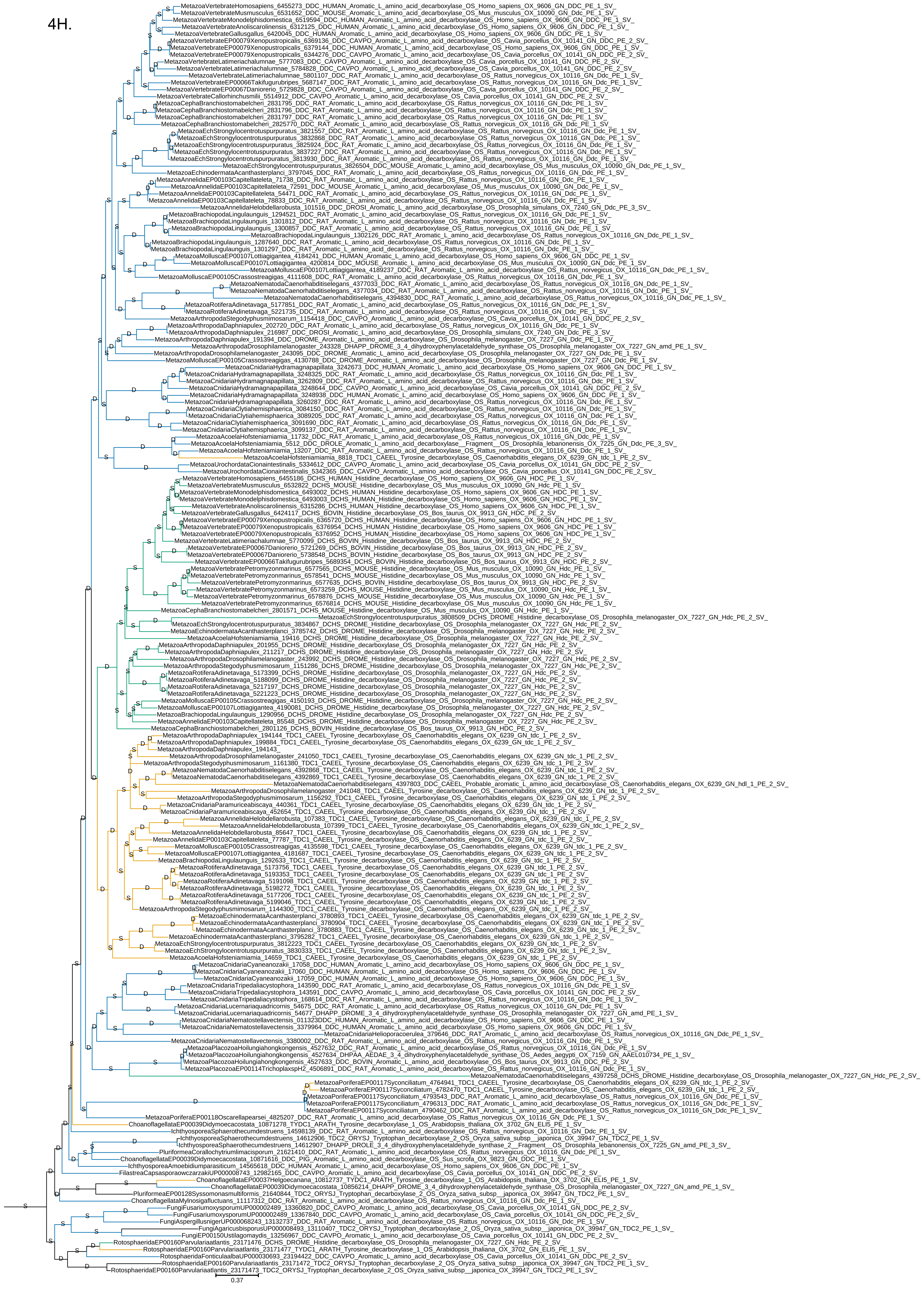
MetazoaVertebrateHomosapiens_6455273_DDC_HUMAN_Aromatic_L_amino_acid_decarboxylase_OS_Homo_sapiens_OX_9606_GN_DDC_PE_1_SV_
MetazoaVertebrateMusmusculus_6531652_DDC_MOUSE_Aromatic_L_amino_acid_decarboxylase_OS_Mus_musculus_OX_10090_GN_Ddc_PE_1_SV_
MetazoaVertebrateMonodelphisdomestica_6519594_DDC_HUMAN_Aromatic_L_amino_acid_decarboxylase_OS_Homo_sapiens_OX_9606_GN_DDC_PE_1_SV_
MetazoaVertebrateAnoliscarolinensis_6312125_DDC_HUMAN_Aromatic_L_amino_acid_decarboxylase_OS_Homo_sapiens_OX_9606_GN_DDC_PE_1_SV_
MetazoaVertebrateGallusgallus_6420045_DDC_HUMAN_Aromatic_L_amino_acid_decarboxylase_OS_Homo_sapiens_OX_9606_GN_DDC_PE_1_SV_
MetazoaVertebrateEP00079Xenopustropicalis_6369136_DDC_CAVPO_Aromatic_L_amino_acid_decarboxylase_OS_Cavia_porcellus_OX_10141_GN_DDC_PE_2_SV_
MetazoaVertebrateEP00079Xenopustropicalis_6379144_DDC_HUMAN_Aromatic_L_amino_acid_decarboxylase_OS_Homo_sapiens_OX_9606_GN_DDC_PE_1_SV_
MetazoaVertebrateEP00079Xenopustropicalis_6344276_DDC_CAVPO_Aromatic_L_amino_acid_decarboxylase_OS_Cavia_porcellus_OX_10141_GN_DDC_PE_2_SV_
MetazoaVertebrateLatimeriachalumnae_5777083_DDC_CAVPO_Aromatic_L_amino_acid_decarboxylase_OS_Cavia_porcellus_OX_10141_GN_DDC_PE_2_SV_
MetazoaVertebrateLatimeriachalumnae_5784828_DDC_CAVPO_Aromatic_L_amino_acid_decarboxylase_OS_Cavia_porcellus_OX_10141_GN_DDC_PE_2_SV_
MetazoaVertebrateLatimeriachalumnae_5801107_DDC_RAT_Aromatic_L_amino_acid_decarboxylase_OS_Rattus_norvegicus_OX_10116_GN_Ddc_PE_1_SV_
MetazoaVertebrateEP00066Takifugurubripes_5687147_DDC_RAT_Aromatic_L_amino_acid_decarboxylase_OS_Rattus_norvegicus_OX_10116_GN_Ddc_PE_1_SV_
MetazoaVertebrateEP00067Daniorerio_5729828_DDC_CAVPO_Aromatic_L_amino_acid_decarboxylase_OS_Cavia_porcellus_OX_10141_GN_DDC_PE_2_SV_
MetazoaVertebrateCallorhinchusmilii_5514912_DDC_CAVPO_Aromatic_L_amino_acid_decarboxylase_OS_Cavia_porcellus_OX_10141_GN_DDC_PE_2_SV_
MetazoaCephaBranchiostomabelcheri_2831795_DDC_RAT_Aromatic_L_amino_acid_decarboxylase_OS_Rattus_norvegicus_OX_10116_GN_Ddc_PE_1_SV_
MetazoaCephaBranchiostomabelcheri_2831796_DDC_RAT_Aromatic_L_amino_acid_decarboxylase_OS_Rattus_norvegicus_OX_10116_GN_Ddc_PE_1_SV_
MetazoaCephaBranchiostomabelcheri_2831797_DDC_RAT_Aromatic_L_amino_acid_decarboxylase_OS_Rattus_norvegicus_OX_10116_GN_Ddc_PE_1_SV_
MetazoaCephaBranchiostomabelcheri_2825770_DDC_RAT_Aromatic_L_amino_acid_decarboxylase_OS_Rattus_norvegicus_OX_10116_GN_Ddc_PE_1_SV_
MetazoaEchStrongylocentrotuspurpuratus_3821557_DDC_RAT_Aromatic_L_amino_acid_decarboxylase_OS_Rattus_norvegicus_OX_10116_GN_Ddc_PE_1_SV_
MetazoaEchStrongylocentrotuspurpuratus_3832868_DDC_RAT_Aromatic_L_amino_acid_decarboxylase_OS_Rattus_norvegicus_OX_10116_GN_Ddc_PE_1_SV_
MetazoaEchStrongylocentrotuspurpuratus_3825924_DDC_RAT_Aromatic_L_amino_acid_decarboxylase_OS_Rattus_norvegicus_OX_10116_GN_Ddc_PE_1_SV_
MetazoaEchStrongylocentrotuspurpuratus_3837227_DDC_RAT_Aromatic_L_amino_acid_decarboxylase_OS_Rattus_norvegicus_OX_10116_GN_Ddc_PE_1_SV_
MetazoaEchStrongylocentrotuspurpuratus_3813930_DDC_RAT_Aromatic_L_amino_acid_decarboxylase_OS_Rattus_norvegicus_OX_10116_GN_Ddc_PE_1_SV_
MetazoaEchStrongylocentrotuspurpuratus_3826504_DDC_MOUSE_Aromatic_L_amino_acid_decarboxylase_OS_Mus_musculus_OX_10090_GN_Ddc_PE_1_SV_
MetazoaEchinodermataAcanthasterplanci_3797045_DDC_RAT_Aromatic_L_amino_acid_decarboxylase_OS_Rattus_norvegicus_OX_10116_GN_Ddc_PE_1_SV_
MetazoaAnnelidaEP00103Capitellateleta_71738_DDC_RAT_Aromatic_L_amino_acid_decarboxylase_OS_Rattus_norvegicus_OX_10116_GN_Ddc_PE_1_SV_
MetazoaAnnelidaEP00103Capitellateleta_72591_DDC_MOUSE_Aromatic_L_amino_acid_decarboxylase_OS_Mus_musculus_OX_10090_GN_Ddc_PE_1_SV_
MetazoaAnnelidaEP00103Capitellateleta_54471_DDC_RAT_Aromatic_L_amino_acid_decarboxylase_OS_Rattus_norvegicus_OX_10116_GN_Ddc_PE_1_SV_
MetazoaAnnelidaEP00103Capitellateleta_78833_DDC_RAT_Aromatic_L_amino_acid_decarboxylase_OS_Rattus_norvegicus_OX_10116_GN_Ddc_PE_1_SV_
MetazoaAnnelidaHelobdellarobusta_101516_DDC_DROSI_Aromatic_L_amino_acid_decarboxylase_OS_Drosophila_simulans_OX_7240_GN_Ddc_PE_3_SV_
MetazoaBrachiopodaLingulaunguis_1294521_DDC_RAT_Aromatic_L_amino_acid_decarboxylase_OS_Rattus_norvegicus_OX_10116_GN_Ddc_PE_1_SV_
MetazoaBrachiopodaLingulaunguis_1301812_DDC_RAT_Aromatic_L_amino_acid_decarboxylase_OS_Rattus_norvegicus_OX_10116_GN_Ddc_PE_1_SV_
MetazoaBrachiopodaLingulaunguis_1300857_DDC_RAT_Aromatic_L_amino_acid_decarboxylase_OS_Rattus_norvegicus_OX_10116_GN_Ddc_PE_1_SV_
MetazoaBrachiopodaLingulaunguis_1302126_DDC_RAT_Aromatic_L_amino_acid_decarboxylase_OS_Rattus_norvegicus_OX_10116_GN_Ddc_PE_1_SV_
MetazoaBrachiopodaLingulaunguis_1287640_DDC_RAT_Aromatic_L_amino_acid_decarboxylase_OS_Rattus_norvegicus_OX_10116_GN_Ddc_PE_1_SV_
MetazoaBrachiopodaLingulaunguis_1301297_DDC_RAT_Aromatic_L_amino_acid_decarboxylase_OS_Rattus_norvegicus_OX_10116_GN_Ddc_PE_1_SV_
MetazoaMolluscaEP00107Lottiagigantea_4184241_DDC_HUMAN_Aromatic_L_amino_acid_decarboxylase_OS_Homo_sapiens_OX_9606_GN_DDC_PE_1_SV_
MetazoaMolluscaEP00107Lottiagigantea_4200814_DDC_MOUSE_Aromatic_L_amino_acid_decarboxylase_OS_Mus_musculus_OX_10090_GN_Ddc_PE_1_SV_
MetazoaMolluscaEP00107Lottiagigantea_4189237_DDC_RAT_Aromatic_L_amino_acid_decarboxylase_OS_Rattus_norvegicus_OX_10116_GN_Ddc_PE_1_SV_
MetazoaMolluscaEP00105Crassostreagigas_4111608_DDC_RAT_Aromatic_L_amino_acid_decarboxylase_OS_Rattus_norvegicus_OX_10116_GN_Ddc_PE_1_SV_
MetazoaNematodaCaenorhabditiselegans_4377033_DDC_RAT_Aromatic_L_amino_acid_decarboxylase_OS_Rattus_norvegicus_OX_10116_GN_Ddc_PE_1_SV_
MetazoaNematodaCaenorhabditiselegans_4377034_DDC_RAT_Aromatic_L_amino_acid_decarboxylase_OS_Rattus_norvegicus_OX_10116_GN_Ddc_PE_1_SV_
MetazoaNematodaCaenorhabditiselegans_4394830_DDC_RAT_Aromatic_L_amino_acid_decarboxylase_OS_Rattus_norvegicus_OX_10116_GN_Ddc_PE_1_SV_
MetazoaRotiferaAdinetavaga_5177851_DDC_RAT_Aromatic_L_amino_acid_decarboxylase_OS_Rattus_norvegicus_OX_10116_GN_Ddc_PE_1_SV_
MetazoaRotiferaAdinetavaga_5221735_DDC_RAT_Aromatic_L_amino_acid_decarboxylase_OS_Rattus_norvegicus_OX_10116_GN_Ddc_PE_1_SV_
MetazoaArthropodaStegodyphusmimosarum_1154418_DDC_CAVPO_Aromatic_L_amino_acid_decarboxylase_OS_Cavia_porcellus_OX_10141_GN_DDC_PE_2_SV_
MetazoaArthropodaDaphniapulex_202720_DDC_RAT_Aromatic_L_amino_acid_decarboxylase_OS_Rattus_norvegicus_OX_10116_GN_Ddc_PE_1_SV_
MetazoaArthropodaDaphniapulex_216987_DDC_DROSI_Aromatic_L_amino_acid_decarboxylase_OS_Drosophila_simulans_OX_7240_GN_Ddc_PE_3_SV_
MetazoaArthropodaDaphniapulex_191394_DDC_DROME_Aromatic_L_amino_acid_decarboxylase_OS_Drosophila_melanogaster_OX_7227_GN_Ddc_PE_1_SV_
MetazoaArthropodaDrosophilamelanogaster_243328_DHAPP_DROME_3_4_dihydroxyphenylacetaldehyde_synthase_OS_Drosophila_melanogaster_OX_7227_GN_amd_PE_1_SV_
MetazoaArthropodaDrosophilamelanogaster_243095_DDC_DROME_Aromatic_L_amino_acid_decarboxylase_OS_Drosophila_melanogaster_OX_7227_GN_Ddc_PE_1_SV_
MetazoaMolluscaEP00105Crassostreagigas_4130788_DDC_DROME_Aromatic_L_amino_acid_decarboxylase_OS_Drosophila_melanogaster_OX_7227_GN_Ddc_PE_1_SV_
MetazoaCnidariaHydramagnapapillata_3242673_DDC_HUMAN_Aromatic_L_amino_acid_decarboxylase_OS_Homo_sapiens_OX_9606_GN_DDC_PE_1_SV_
MetazoaCnidariaHydramagnapapillata_3248325_DDC_RAT_Aromatic_L_amino_acid_decarboxylase_OS_Rattus_norvegicus_OX_10116_GN_Ddc_PE_1_SV_
MetazoaCnidariaHydramagnapapillata_3262809_DDC_RAT_Aromatic_L_amino_acid_decarboxylase_OS_Rattus_norvegicus_OX_10116_GN_Ddc_PE_1_SV_
MetazoaCnidariaHydramagnapapillata_3248644_DDC_CAVPO_Aromatic_L_amino_acid_decarboxylase_OS_Cavia_porcellus_OX_10141_GN_DDC_PE_2_SV_
MetazoaCnidariaHydramagnapapillata_3248938_DDC_HUMAN_Aromatic_L_amino_acid_decarboxylase_OS_Homo_sapiens_OX_9606_GN_DDC_PE_1_SV_
MetazoaCnidariaHydramagnapapillata_3260287_DDC_RAT_Aromatic_L_amino_acid_decarboxylase_OS_Rattus_norvegicus_OX_10116_GN_Ddc_PE_1_SV_
MetazoaCnidariaClytiahemisphaerica_3084150_DDC_RAT_Aromatic_L_amino_acid_decarboxylase_OS_Rattus_norvegicus_OX_10116_GN_Ddc_PE_1_SV_
MetazoaCnidariaClytiahemisphaerica_3089205_DDC_RAT_Aromatic_L_amino_acid_decarboxylase_OS_Rattus_norvegicus_OX_10116_GN_Ddc_PE_1_SV_
MetazoaCnidariaClytiahemisphaerica_3091690_DDC_RAT_Aromatic_L_amino_acid_decarboxylase_OS_Rattus_norvegicus_OX_10116_GN_Ddc_PE_1_SV_
MetazoaCnidariaClytiahemisphaerica_3099137_DDC_RAT_Aromatic_L_amino_acid_decarboxylase_OS_Rattus_norvegicus_OX_10116_GN_Ddc_PE_1_SV_
MetazoaAcoelaHofsteniamiamia_11732_DDC_RAT_Aromatic_L_amino_acid_decarboxylase_OS_Rattus_norvegicus_OX_10116_GN_Ddc_PE_1_SV_
MetazoaAcoelaHofsteniamiamia_5512_DDC_DROLE_Aromatic_L_amino_acid_decarboxylase__Fragment__OS_Drosophila_lebanonensis_OX_7225_GN_Ddc_PE_3_SV_
MetazoaAcoelaHofsteniamiamia_13207_DDC_RAT_Aromatic_L_amino_acid_decarboxylase_OS_Rattus_norvegicus_OX_10116_GN_Ddc_PE_1_SV_
MetazoaAcoelaHofsteniamiamia_8818_TDC1_CAEEL_Tyrosine_decarboxylase_OS_Caenorhabditis_elegans_OX_6239_GN_tdc_1_PE_2_SV_
MetazoaUrochordataCionaintestinalis_5334612_DDC_CAVPO_Aromatic_L_amino_acid_decarboxylase_OS_Cavia_porcellus_OX_10141_GN_DDC_PE_2_SV_
MetazoaUrochordataCionaintestinalis_5342365_DDC_CAVPO_Aromatic_L_amino_acid_decarboxylase_OS_Cavia_porcellus_OX_10141_GN_DDC_PE_2_SV_
MetazoaVertebrateHomosapiens_6455186_DCHS_HUMAN_Histidine_decarboxylase_OS_Homo_sapiens_OX_9606_GN_HDC_PE_1_SV_
MetazoaVertebrateMusmusculus_6532822_DCHS_MOUSE_Histidine_decarboxylase_OS_Mus_musculus_OX_10090_GN_Hdc_PE_1_SV_
MetazoaVertebrateMonodelphisdomestica_6493002_DCHS_HUMAN_Histidine_decarboxylase_OS_Homo_sapiens_OX_9606_GN_HDC_PE_1_SV_
MetazoaVertebrateMonodelphisdomestica_6493003_DCHS_HUMAN_Histidine_decarboxylase_OS_Homo_sapiens_OX_9606_GN_HDC_PE_1_SV_
MetazoaVertebrateAnoliscarolinensis_6315286_DCHS_HUMAN_Histidine_decarboxylase_OS_Homo_sapiens_OX_9606_GN_HDC_PE_1_SV_
MetazoaVertebrateGallusgallus_6424117_DCHS_BOVIN_Histidine_decarboxylase_OS_Bos_taurus_OX_9913_GN_HDC_PE_2_SV_
MetazoaVertebrateEP00079Xenopustropicalis_6365720_DCHS_HUMAN_Histidine_decarboxylase_OS_Homo_sapiens_OX_9606_GN_HDC_PE_1_SV_
MetazoaVertebrateEP00079Xenopustropicalis_6376954_DCHS_HUMAN_Histidine_decarboxylase_OS_Homo_sapiens_OX_9606_GN_HDC_PE_1_SV_
MetazoaVertebrateEP00079Xenopustropicalis_6376952_DCHS_HUMAN_Histidine_decarboxylase_OS_Homo_sapiens_OX_9606_GN_HDC_PE_1_SV_
MetazoaVertebrateLatimeriachalumnae_5770099_DCHS_BOVIN_Histidine_decarboxylase_OS_Bos_taurus_OX_9913_GN_HDC_PE_2_SV_
MetazoaVertebrateEP00067Daniorerio_5721269_DCHS_BOVIN_Histidine_decarboxylase_OS_Bos_taurus_OX_9913_GN_HDC_PE_2_SV_
MetazoaVertebrateEP00067Daniorerio_5738548_DCHS_BOVIN_Histidine_decarboxylase_OS_Bos_taurus_OX_9913_GN_HDC_PE_2_SV_
MetazoaVertebrateEP00066Takifugurubripes_5689354_DCHS_BOVIN_Histidine_decarboxylase_OS_Bos_taurus_OX_9913_GN_HDC_PE_2_SV_
MetazoaVertebratePetromyzonmarinus_6577565_DCHS_MOUSE_Histidine_decarboxylase_OS_Mus_musculus_OX_10090_GN_Hdc_PE_1_SV_
MetazoaVertebratePetromyzonmarinus_6578541_DCHS_MOUSE_Histidine_decarboxylase_OS_Mus_musculus_OX_10090_GN_Hdc_PE_1_SV_
MetazoaVertebratePetromyzonmarinus_6577635_DCHS_BOVIN_Histidine_decarboxylase_OS_Bos_taurus_OX_9913_GN_HDC_PE_2_SV_
MetazoaVertebratePetromyzonmarinus_6573259_DCHS_MOUSE_Histidine_decarboxylase_OS_Mus_musculus_OX_10090_GN_Hdc_PE_1_SV_
MetazoaVertebratePetromyzonmarinus_6578876_DCHS_MOUSE_Histidine_decarboxylase_OS_Mus_musculus_OX_10090_GN_Hdc_PE_1_SV_
MetazoaVertebratePetromyzonmarinus_6576814_DCHS_MOUSE_Histidine_decarboxylase_OS_Mus_musculus_OX_10090_GN_Hdc_PE_1_SV_
MetazoaCephaBranchiostomabelcheri_2801571_DCHS_MOUSE_Histidine_decarboxylase_OS_Mus_musculus_OX_10090_GN_Hdc_PE_1_SV_
MetazoaEchStrongylocentrotuspurpuratus_3808509_DCHS_DROME_Histidine_decarboxylase_OS_Drosophila_melanogaster_OX_7227_GN_Hdc_PE_2_SV_
MetazoaEchStrongylocentrotuspurpuratus_3834867_DCHS_DROME_Histidine_decarboxylase_OS_Drosophila_melanogaster_OX_7227_GN_Hdc_PE_2_SV_
MetazoaEchinodermataAcanthasterplanci_3785742_DCHS_DROME_Histidine_decarboxylase_OS_Drosophila_melanogaster_OX_7227_GN_Hdc_PE_2_SV_
MetazoaAcoelaHofsteniamiamia_19416_DCHS_DROME_Histidine_decarboxylase_OS_Drosophila_melanogaster_OX_7227_GN_Hdc_PE_2_SV_
MetazoaArthropodaDaphniapulex_201955_DCHS_DROME_Histidine_decarboxylase_OS_Drosophila_melanogaster_OX_7227_GN_Hdc_PE_2_SV_
MetazoaArthropodaDaphniapulex_211217_DCHS_DROME_Histidine_decarboxylase_OS_Drosophila_melanogaster_OX_7227_GN_Hdc_PE_2_SV_
MetazoaArthropodaDrosophilamelanogaster_243992_DCHS_DROME_Histidine_decarboxylase_OS_Drosophila_melanogaster_OX_7227_GN_Hdc_PE_2_SV_
MetazoaArthropodaStegodyphusmimosarum_1151286_DCHS_DROME_Histidine_decarboxylase_OS_Drosophila_melanogaster_OX_7227_GN_Hdc_PE_2_SV_
MetazoaRotiferaAdinetavaga_5173399_DCHS_DROME_Histidine_decarboxylase_OS_Drosophila_melanogaster_OX_7227_GN_Hdc_PE_2_SV_
MetazoaRotiferaAdinetavaga_5188099_DCHS_DROME_Histidine_decarboxylase_OS_Drosophila_melanogaster_OX_7227_GN_Hdc_PE_2_SV_
MetazoaRotiferaAdinetavaga_5217197_DCHS_DROME_Histidine_decarboxylase_OS_Drosophila_melanogaster_OX_7227_GN_Hdc_PE_2_SV_
MetazoaRotiferaAdinetavaga_5221283_DCHS_DROME_Histidine_decarboxylase_OS_Drosophila_melanogaster_OX_7227_GN_Hdc_PE_2_SV_
MetazoaMolluscaEP00105Crassostreagigas_4150193_DCHS_DROME_Histidine_decarboxylase_OS_Drosophila_melanogaster_OX_7227_GN_Hdc_PE_2_SV_
MetazoaMolluscaEP00107Lottiagigantea_4190081_DCHS_DROME_Histidine_decarboxylase_OS_Drosophila_melanogaster_OX_7227_GN_Hdc_PE_2_SV_
MetazoaBrachiopodaLingulaunguis_1290956_DCHS_DROME_Histidine_decarboxylase_OS_Drosophila_melanogaster_OX_7227_GN_Hdc_PE_2_SV_
MetazoaAnnelidaEP00103Capitellateleta_85548_DCHS_DROME_Histidine_decarboxylase_OS_Drosophila_melanogaster_OX_7227_GN_Hdc_PE_2_SV_
MetazoaCephaBranchiostomabelcheri_2801126_DCHS_BOVIN_Histidine_decarboxylase_OS_Bos_taurus_OX_9913_GN_HDC_PE_2_SV_
MetazoaArthropodaDaphniapulex_194144_TDC1_CAEEL_Tyrosine_decarboxylase_OS_Caenorhabditis_elegans_OX_6239_GN_tdc_1_PE_2_SV_
MetazoaArthropodaDaphniapulex_199884_TDC1_CAEEL_Tyrosine_decarboxylase_OS_Caenorhabditis_elegans_OX_6239_GN_tdc_1_PE_2_SV_
MetazoaArthropodaDaphniapulex_194143_
MetazoaArthropodaDrosophilamelanogaster_241050_TDC1_CAEEL_Tyrosine_decarboxylase_OS_Caenorhabditis_elegans_OX_6239_GN_tdc_1_PE_2_SV_
MetazoaArthropodaStegodyphusmimosarum_1161380_TDC1_CAEEL_Tyrosine_decarboxylase_OS_Caenorhabditis_elegans_OX_6239_GN_tdc_1_PE_2_SV_
MetazoaNematodaCaenorhabditiselegans_4392868_TDC1_CAEEL_Tyrosine_decarboxylase_OS_Caenorhabditis_elegans_OX_6239_GN_tdc_1_PE_2_SV_
MetazoaNematodaCaenorhabditiselegans_4392869_TDC1_CAEEL_Tyrosine_decarboxylase_OS_Caenorhabditis_elegans_OX_6239_GN_tdc_1_PE_2_SV_
MetazoaNematodaCaenorhabditiselegans_4397803_DDC_CAEEL_Probable_aromatic_L_amino_acid_decarboxylase_OS_Caenorhabditis_elegans_OX_6239_GN_hdl_1_PE_2_SV_
MetazoaArthropodaDrosophilamelanogaster_241048_TDC1_CAEEL_Tyrosine_decarboxylase_OS_Caenorhabditis_elegans_OX_6239_GN_tdc_1_PE_2_SV_
MetazoaArthropodaStegodyphusmimosarum_1156292_TDC1_CAEEL_Tyrosine_decarboxylase_OS_Caenorhabditis_elegans_OX_6239_GN_tdc_1_PE_2_SV_
MetazoaCnidariaParamuriceabiscaya_440361_TDC1_CAEEL_Tyrosine_decarboxylase_OS_Caenorhabditis_elegans_OX_6239_GN_tdc_1_PE_2_SV_
MetazoaCnidariaParamuriceabiscaya_452654_TDC1_CAEEL_Tyrosine_decarboxylase_OS_Caenorhabditis_elegans_OX_6239_GN_tdc_1_PE_2_SV_
MetazoaAnnelidaHelobdellarobusta_107383_TDC1_CAEEL_Tyrosine_decarboxylase_OS_Caenorhabditis_elegans_OX_6239_GN_tdc_1_PE_2_SV_
MetazoaAnnelidaHelobdellarobusta_107399_TDC1_CAEEL_Tyrosine_decarboxylase_OS_Caenorhabditis_elegans_OX_6239_GN_tdc_1_PE_2_SV_
MetazoaAnnelidaHelobdellarobusta_85647_TDC1_CAEEL_Tyrosine_decarboxylase_OS_Caenorhabditis_elegans_OX_6239_GN_tdc_1_PE_2_SV_
MetazoaAnnelidaEP00103Capitellateleta_77787_TDC1_CAEEL_Tyrosine_decarboxylase_OS_Caenorhabditis_elegans_OX_6239_GN_tdc_1_PE_2_SV_
MetazoaMolluscaEP00105Crassostreagigas_4135598_TDC1_CAEEL_Tyrosine_decarboxylase_OS_Caenorhabditis_elegans_OX_6239_GN_tdc_1_PE_2_SV_
MetazoaMolluscaEP00107Lottiagigantea_4181687_TDC1_CAEEL_Tyrosine_decarboxylase_OS_Caenorhabditis_elegans_OX_6239_GN_tdc_1_PE_2_SV_
MetazoaBrachiopodaLingulaunguis_1292633_TDC1_CAEEL_Tyrosine_decarboxylase_OS_Caenorhabditis_elegans_OX_6239_GN_tdc_1_PE_2_SV_
MetazoaRotiferaAdinetavaga_5173756_TDC1_CAEEL_Tyrosine_decarboxylase_OS_Caenorhabditis_elegans_OX_6239_GN_tdc_1_PE_2_SV_
MetazoaRotiferaAdinetavaga_5193353_TDC1_CAEEL_Tyrosine_decarboxylase_OS_Caenorhabditis_elegans_OX_6239_GN_tdc_1_PE_2_SV_
MetazoaRotiferaAdinetavaga_5191098_TDC1_CAEEL_Tyrosine_decarboxylase_OS_Caenorhabditis_elegans_OX_6239_GN_tdc_1_PE_2_SV_
MetazoaRotiferaAdinetavaga_5198272_TDC1_CAEEL_Tyrosine_decarboxylase_OS_Caenorhabditis_elegans_OX_6239_GN_tdc_1_PE_2_SV_
MetazoaRotiferaAdinetavaga_5177206_TDC1_CAEEL_Tyrosine_decarboxylase_OS_Caenorhabditis_elegans_OX_6239_GN_tdc_1_PE_2_SV_
MetazoaRotiferaAdinetavaga_5199046_TDC1_CAEEL_Tyrosine_decarboxylase_OS_Caenorhabditis_elegans_OX_6239_GN_tdc_1_PE_2_SV_
MetazoaArthropodaStegodyphusmimosarum_1144300_TDC1_CAEEL_Tyrosine_decarboxylase_OS_Caenorhabditis_elegans_OX_6239_GN_tdc_1_PE_2_SV_
MetazoaEchinodermataAcanthasterplanci_3780893_TDC1_CAEEL_Tyrosine_decarboxylase_OS_Caenorhabditis_elegans_OX_6239_GN_tdc_1_PE_2_SV_
MetazoaEchinodermataAcanthasterplanci_3780904_TDC1_CAEEL_Tyrosine_decarboxylase_OS_Caenorhabditis_elegans_OX_6239_GN_tdc_1_PE_2_SV_
MetazoaEchinodermataAcanthasterplanci_3780883_TDC1_CAEEL_Tyrosine_decarboxylase_OS_Caenorhabditis_elegans_OX_6239_GN_tdc_1_PE_2_SV_
MetazoaEchinodermataAcanthasterplanci_3795282_TDC1_CAEEL_Tyrosine_decarboxylase_OS_Caenorhabditis_elegans_OX_6239_GN_tdc_1_PE_2_SV_
MetazoaEchStrongylocentrotuspurpuratus_3812223_TDC1_CAEEL_Tyrosine_decarboxylase_OS_Caenorhabditis_elegans_OX_6239_GN_tdc_1_PE_2_SV_
MetazoaEchStrongylocentrotuspurpuratus_3830333_TDC1_CAEEL_Tyrosine_decarboxylase_OS_Caenorhabditis_elegans_OX_6239_GN_tdc_1_PE_2_SV_
MetazoaAcoelaHofsteniamiamia_14659_TDC1_CAEEL_Tyrosine_decarboxylase_OS_Caenorhabditis_elegans_OX_6239_GN_tdc_1_PE_2_SV_
MetazoaCnidariaCyaneanozakii_17058_DDC_HUMAN_Aromatic_L_amino_acid_decarboxylase_OS_Homo_sapiens_OX_9606_GN_DDC_PE_1_SV_
MetazoaCnidariaCyaneanozakii_17060_DDC_HUMAN_Aromatic_L_amino_acid_decarboxylase_OS_Homo_sapiens_OX_9606_GN_DDC_PE_1_SV_
MetazoaCnidariaCyaneanozakii_17059_DDC_HUMAN_Aromatic_L_amino_acid_decarboxylase_OS_Homo_sapiens_OX_9606_GN_DDC_PE_1_SV_
MetazoaCnidariaTripedaliacystophora_143590_DDC_RAT_Aromatic_L_amino_acid_decarboxylase_OS_Rattus_norvegicus_OX_10116_GN_Ddc_PE_1_SV_
MetazoaCnidariaTripedaliacystophora_143591_DDC_CAVPO_Aromatic_L_amino_acid_decarboxylase_OS_Cavia_porcellus_OX_10141_GN_DDC_PE_2_SV_
MetazoaCnidariaTripedaliacystophora_168614_DDC_RAT_Aromatic_L_amino_acid_decarboxylase_OS_Rattus_norvegicus_OX_10116_GN_Ddc_PE_1_SV_
MetazoaCnidariaLucernariaquadricornis_54675_DDC_RAT_Aromatic_L_amino_acid_decarboxylase_OS_Rattus_norvegicus_OX_10116_GN_Ddc_PE_1_SV_
MetazoaCnidariaLucernariaquadricornis_54677_DHAPP_DROME_3_4_dihydroxyphenylacetaldehyde_synthase_OS_Drosophila_melanogaster_OX_7227_GN_amd_PE_1_SV_
MetazoaCnidariaNematostellavectensis_011323DDC_HUMAN_Aromatic_L_amino_acid_decarboxylase_OS_Homo_sapiens_OX_9606_GN_DDC_PE_1_SV_
MetazoaCnidariaNematostellavectensis_3379964_DDC_HUMAN_Aromatic_L_amino_acid_decarboxylase_OS_Homo_sapiens_OX_9606_GN_DDC_PE_1_SV_
MetazoaCnidariaHelioporacoerulea_379646_DDC_RAT_Aromatic_L_amino_acid_decarboxylase_OS_Rattus_norvegicus_OX_10116_GN_Ddc_PE_1_SV_
MetazoaCnidariaNematostellavectensis_3380002_DDC_RAT_Aromatic_L_amino_acid_decarboxylase_OS_Rattus_norvegicus_OX_10116_GN_Ddc_PE_1_SV_
MetazoaPlacozoaHoilungiahongkongensis_4527632_DDC_RAT_Aromatic_L_amino_acid_decarboxylase_OS_Rattus_norvegicus_OX_10116_GN_Ddc_PE_1_SV_
MetazoaPlacozoaHoilungiahongkongensis_4527634_DHPAA_AEDAE_3_4_dihydroxyphenylacetaldehyde_synthase_OS_Aedes_aegypti_OX_7159_GN_AAEL010734_PE_1_SV_
MetazoaPlacozoaHoilungiahongkongensis_4527633_DDC_BOVIN_Aromatic_L_amino_acid_decarboxylase_OS_Bos_taurus_OX_9913_GN_DDC_PE_2_SV_
MetazoaPlacozoaEP00114TrichoplaxspH2_4506891_DDC_RAT_Aromatic_L_amino_acid_decarboxylase_OS_Rattus_norvegicus_OX_10116_GN_Ddc_PE_1_SV_
MetazoaNematodaCaenorhabditiselegans_4397258_DCHS_DROME_Histidine_decarboxylase_OS_Drosophila_melanogaster_OX_7227_GN_Hdc_PE_2_SV_
MetazoaPoriferaEP00117Syconciliatum_4764941_TDC1_CAEEL_Tyrosine_decarboxylase_OS_Caenorhabditis_elegans_OX_6239_GN_tdc_1_PE_2_SV_
MetazoaPoriferaEP00117Syconciliatum_4782470_TDC1_CAEEL_Tyrosine_decarboxylase_OS_Caenorhabditis_elegans_OX_6239_GN_tdc_1_PE_2_SV_
MetazoaPoriferaEP00117Syconciliatum_4793543_DDC_RAT_Aromatic_L_amino_acid_decarboxylase_OS_Rattus_norvegicus_OX_10116_GN_Ddc_PE_1_SV_
MetazoaPoriferaEP00117Syconciliatum_4796313_DDC_RAT_Aromatic_L_amino_acid_decarboxylase_OS_Rattus_norvegicus_OX_10116_GN_Ddc_PE_1_SV_
MetazoaPoriferaEP00117Syconciliatum_4790462_DDC_RAT_Aromatic_L_amino_acid_decarboxylase_OS_Rattus_norvegicus_OX_10116_GN_Ddc_PE_1_SV_
MetazoaPoriferaEP00118Oscarellapearsei_4825207_DDC_RAT_Aromatic_L_amino_acid_decarboxylase_OS_Rattus_norvegicus_OX_10116_GN_Ddc_PE_1_SV_
ChoanoflagellataEP00039Didymoecacostata_10871278_TYDC1_ARATH_Tyrosine_decarboxylase_1_OS_Arabidopsis_thaliana_OX_3702_GN_ELI5_PE_1_SV_
IchthyosporeaSphaerothecumdestruens_14598139_DDC_RAT_Aromatic_L_amino_acid_decarboxylase_OS_Rattus_norvegicus_OX_10116_GN_Ddc_PE_1_SV_
IchthyosporeaSphaerothecumdestruens_14612906_TDC2_ORYSJ_Tryptophan_decarboxylase_2_OS_Oryza_sativa_subsp__japonica_OX_39947_GN_TDC2_PE_1_SV_
IchthyosporeaSphaerothecumdestruens_14612907_DHAPP_DROLE_3_4_dihydroxyphenylacetaldehyde_synthase_2__Fragment__OS_Drosophila_lebanonensis_OX_7225_GN_amd_PE_3_SV_
PluriformeaCorallochytriumlimacisporum_21621410_DDC_RAT_Aromatic_L_amino_acid_decarboxylase_OS_Rattus_norvegicus_OX_10116_GN_Ddc_PE_1_SV_
ChoanoflagellataEP00039Didymoecacostata_10871616_DDC_PIG_Aromatic_L_amino_acid_decarboxylase_OS_Sus_scrofa_OX_9823_GN_DDC_PE_1_SV_
IchthyosporeaAmoebidiumparasiticum_14565618_DDC_HUMAN_Aromatic_L_amino_acid_decarboxylase_OS_Homo_sapiens_OX_9606_GN_DDC_PE_1_SV_
FilastreaCapsasporaowczarzakiUP000008743_12982165_DDC_CAVPO_Aromatic_L_amino_acid_decarboxylase_OS_Cavia_porcellus_OX_10141_GN_DDC_PE_2_SV_
ChoanoflagellataEP00037Helgoecanana_10812737_TYDC1_ARATH_Tyrosine_decarboxylase_1_OS_Arabidopsis_thaliana_OX_3702_GN_ELI5_PE_1_SV_
ChoanoflagellataEP00039Didymoecacostata_10856214_DHAPP_DROME_3_4_dihydroxyphenylacetaldehyde_synthase_OS_Drosophila_melanogaster_OX_7227_GN_amd_PE_1_SV_
PluriformeaEP00128Syssomonasmultiformis_21640844_TDC2_ORYSJ_Tryptophan_decarboxylase_2_OS_Oryza_sativa_subsp__japonica_OX_39947_GN_TDC2_PE_1_SV_
ChoanoflagellataMylnosigafluctuans_11117312_DDC_RAT_Aromatic_L_amino_acid_decarboxylase_OS_Rattus_norvegicus_OX_10116_GN_Ddc_PE_1_SV_
FungiFusariumoxysporumUP000002489_13360820_DDC_CAVPO_Aromatic_L_amino_acid_decarboxylase_OS_Cavia_porcellus_OX_10141_GN_DDC_PE_2_SV_
FungiFusariumoxysporumUP000002489_13367840_DDC_CAVPO_Aromatic_L_amino_acid_decarboxylase_OS_Cavia_porcellus_OX_10141_GN_DDC_PE_2_SV_
FungiAspergillusnigerUP000068243_13132737_DDC_RAT_Aromatic_L_amino_acid_decarboxylase_OS_Rattus_norvegicus_OX_10116_GN_Ddc_PE_1_SV_
FungiAgaricusbisporusUP000008493_13110407_TDC2_ORYSJ_Tryptophan_decarboxylase_2_OS_Oryza_sativa_subsp__japonica_OX_39947_GN_TDC2_PE_1_SV_
FungiEP00150Ustilagomaydis_13256967_DDC_CAVPO_Aromatic_L_amino_acid_decarboxylase_OS_Cavia_porcellus_OX_10141_GN_DDC_PE_2_SV_
RotosphaeridaEP00160Parvulariaatlantis_23171476_DCHS_DROME_Histidine_decarboxylase_OS_Drosophila_melanogaster_OX_7227_GN_Hdc_PE_2_SV_
RotosphaeridaEP00160Parvulariaatlantis_23171477_TYDC1_ARATH_Tyrosine_decarboxylase_1_OS_Arabidopsis_thaliana_OX_3702_GN_ELI5_PE_1_SV_
RotosphaeridaFonticulaalbaUP000030693_23194422_DDC_CAVPO_Aromatic_L_amino_acid_decarboxylase_OS_Cavia_porcellus_OX_10141_GN_DDC_PE_2_SV_
RotosphaeridaEP00160Parvulariaatlantis_23171472_TDC2_ORYSJ_Tryptophan_decarboxylase_2_OS_Oryza_sativa_subsp__japonica_OX_39947_GN_TDC2_PE_1_SV_
RotosphaeridaEP00160Parvulariaatlantis_23171473_TDC2_ORYSJ_Tryptophan_decarboxylase_2_OS_Oryza_sativa_subsp__japonica_OX_39947_GN_TDC2_PE_1_SV_
0.37

4I.

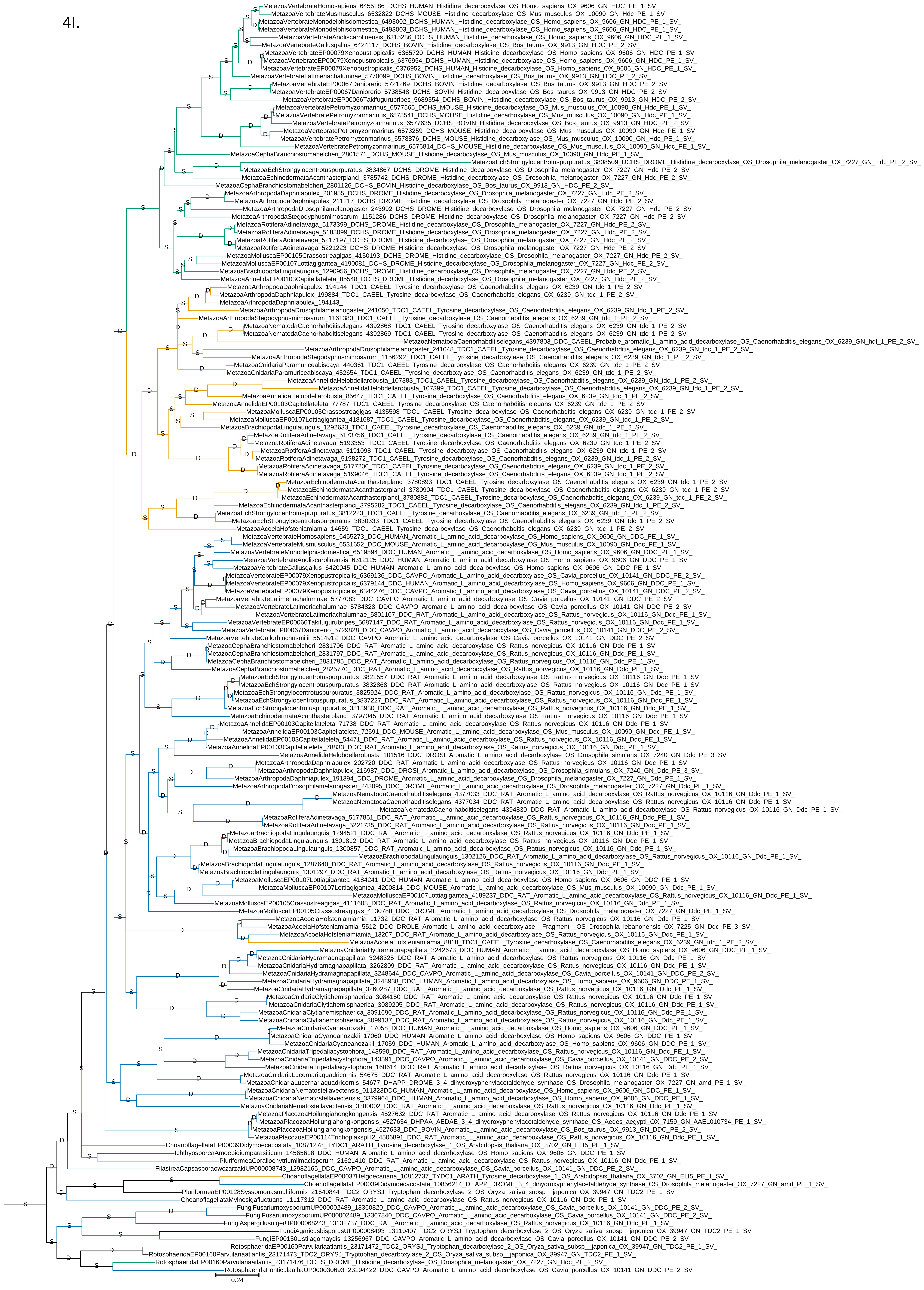

MetazoaVertebrateHomosapiens_6455186_DCHS_HUMAN_Histidine_decarboxylase_OS_Homo_sapiens_OX_9606_GN_HDC_PE_1_SV_
MetazoaVertebrateMusmusculus_6532822_DCHS_MOUSE_Histidine_decarboxylase_OS_Mus_musculus_OX_10090_GN_Hdc_PE_1_SV_
MetazoaVertebrateMonodelphisdomestica_6493002_DCHS_HUMAN_Histidine_decarboxylase_OS_Homo_sapiens_OX_9606_GN_HDC_PE_1_SV_
MetazoaVertebrateMonodelphisdomestica_6493003_DCHS_HUMAN_Histidine_decarboxylase_OS_Homo_sapiens_OX_9606_GN_HDC_PE_1_SV_
MetazoaVertebrateAnoliscarolinensis_6315286_DCHS_HUMAN_Histidine_decarboxylase_OS_Homo_sapiens_OX_9606_GN_HDC_PE_1_SV_
MetazoaVertebrateGallusgallus_6424117_DCHS_BOVIN_Histidine_decarboxylase_OS_Bos_taurus_OX_9913_GN_HDC_PE_2_SV_
MetazoaVertebrateEP00079Xenopustropicalis_6365720_DCHS_HUMAN_Histidine_decarboxylase_OS_Homo_sapiens_OX_9606_GN_HDC_PE_1_SV_
MetazoaVertebrateEP00079Xenopustropicalis_6376954_DCHS_HUMAN_Histidine_decarboxylase_OS_Homo_sapiens_OX_9606_GN_HDC_PE_1_SV_
MetazoaVertebrateEP00079Xenopustropicalis_6376952_DCHS_HUMAN_Histidine_decarboxylase_OS_Homo_sapiens_OX_9606_GN_HDC_PE_1_SV_
MetazoaVertebrateLatimeriachalumnae_5770099_DCHS_BOVIN_Histidine_decarboxylase_OS_Bos_taurus_OX_9913_GN_HDC_PE_2_SV_
MetazoaVertebrateEP00067Daniorerio_5721269_DCHS_BOVIN_Histidine_decarboxylase_OS_Bos_taurus_OX_9913_GN_HDC_PE_2_SV_
MetazoaVertebrateEP00067Daniorerio_5738548_DCHS_BOVIN_Histidine_decarboxylase_OS_Bos_taurus_OX_9913_GN_HDC_PE_2_SV_
MetazoaVertebrateEP00066Takifugurubripes_5689354_DCHS_BOVIN_Histidine_decarboxylase_OS_Bos_taurus_OX_9913_GN_HDC_PE_2_SV_
MetazoaVertebratePetromyzonmarinus_6577565_DCHS_MOUSE_Histidine_decarboxylase_OS_Mus_musculus_OX_10090_GN_Hdc_PE_1_SV_
MetazoaVertebratePetromyzonmarinus_6578541_DCHS_MOUSE_Histidine_decarboxylase_OS_Mus_musculus_OX_10090_GN_Hdc_PE_1_SV_
MetazoaVertebratePetromyzonmarinus_6577635_DCHS_BOVIN_Histidine_decarboxylase_OS_Bos_taurus_OX_9913_GN_HDC_PE_2_SV_
MetazoaVertebratePetromyzonmarinus_6573259_DCHS_MOUSE_Histidine_decarboxylase_OS_Mus_musculus_OX_10090_GN_Hdc_PE_1_SV_
MetazoaVertebratePetromyzonmarinus_6578876_DCHS_MOUSE_Histidine_decarboxylase_OS_Mus_musculus_OX_10090_GN_Hdc_PE_1_SV_
MetazoaVertebratePetromyzonmarinus_6576814_DCHS_MOUSE_Histidine_decarboxylase_OS_Mus_musculus_OX_10090_GN_Hdc_PE_1_SV_
MetazoaCephaBranchiostomabelcheri_2801571_DCHS_MOUSE_Histidine_decarboxylase_OS_Mus_musculus_OX_10090_GN_Hdc_PE_1_SV_
MetazoaEchStrongylocentrotuspurpuratus_3808509_DCHS_DROME_Histidine_decarboxylase_OS_Drosophila_melanogaster_OX_7227_GN_Hdc_PE_2_SV_
MetazoaEchStrongylocentrotuspurpuratus_3834867_DCHS_DROME_Histidine_decarboxylase_OS_Drosophila_melanogaster_OX_7227_GN_Hdc_PE_2_SV_
MetazoaEchinodermataAcanthasterplanci_3785742_DCHS_DROME_Histidine_decarboxylase_OS_Drosophila_melanogaster_OX_7227_GN_Hdc_PE_2_SV_
MetazoaCephaBranchiostomabelcheri_2801126_DCHS_BOVIN_Histidine_decarboxylase_OS_Bos_taurus_OX_9913_GN_HDC_PE_2_SV_
MetazoaArthropodaDaphniapulex_201955_DCHS_DROME_Histidine_decarboxylase_OS_Drosophila_melanogaster_OX_7227_GN_Hdc_PE_2_SV_
MetazoaArthropodaDaphniapulex_211217_DCHS_DROME_Histidine_decarboxylase_OS_Drosophila_melanogaster_OX_7227_GN_Hdc_PE_2_SV_
MetazoaArthropodaDrosophilamelanogaster_243992_DCHS_DROME_Histidine_decarboxylase_OS_Drosophila_melanogaster_OX_7227_GN_Hdc_PE_2_SV_
MetazoaArthropodaStegodyphusmimosarum_1151286_DCHS_DROME_Histidine_decarboxylase_OS_Drosophila_melanogaster_OX_7227_GN_Hdc_PE_2_SV_
MetazoaRotiferaAdinetavaga_5173399_DCHS_DROME_Histidine_decarboxylase_OS_Drosophila_melanogaster_OX_7227_GN_Hdc_PE_2_SV_
MetazoaRotiferaAdinetavaga_5188099_DCHS_DROME_Histidine_decarboxylase_OS_Drosophila_melanogaster_OX_7227_GN_Hdc_PE_2_SV_
MetazoaRotiferaAdinetavaga_5217197_DCHS_DROME_Histidine_decarboxylase_OS_Drosophila_melanogaster_OX_7227_GN_Hdc_PE_2_SV_
MetazoaRotiferaAdinetavaga_5221223_DCHS_DROME_Histidine_decarboxylase_OS_Drosophila_melanogaster_OX_7227_GN_Hdc_PE_2_SV_
MetazoaMolluscaEP00105Crassostreagigas_4150193_DCHS_DROME_Histidine_decarboxylase_OS_Drosophila_melanogaster_OX_7227_GN_Hdc_PE_2_SV_
MetazoaMolluscaEP00107Lottiagigantea_4190081_DCHS_DROME_Histidine_decarboxylase_OS_Drosophila_melanogaster_OX_7227_GN_Hdc_PE_2_SV_
MetazoaBrachiopodaLingulaunguis_1290956_DCHS_DROME_Histidine_decarboxylase_OS_Drosophila_melanogaster_OX_7227_GN_Hdc_PE_2_SV_
MetazoaAnnelidaEP00103Capitellateleta_85548_DCHS_DROME_Histidine_decarboxylase_OS_Drosophila_melanogaster_OX_7227_GN_Hdc_PE_2_SV_
MetazoaArthropodaDaphniapulex_194144_TDC1_CAEEL_Tyrosine_decarboxylase_OS_Caenorhabditis_elegans_OX_6239_GN_tdc_1_PE_2_SV_
MetazoaArthropodaDaphniapulex_199884_TDC1_CAEEL_Tyrosine_decarboxylase_OS_Caenorhabditis_elegans_OX_6239_GN_tdc_1_PE_2_SV_
MetazoaArthropodaDaphniapulex_194143_
MetazoaArthropodaDrosophilamelanogaster_241050_TDC1_CAEEL_Tyrosine_decarboxylase_OS_Caenorhabditis_elegans_OX_6239_GN_tdc_1_PE_2_SV_
MetazoaArthropodaStegodyphusmimosarum_1161380_TDC1_CAEEL_Tyrosine_decarboxylase_OS_Caenorhabditis_elegans_OX_6239_GN_tdc_1_PE_2_SV_
MetazoaNematodaCaenorhabditiselegans_4392868_TDC1_CAEEL_Tyrosine_decarboxylase_OS_Caenorhabditis_elegans_OX_6239_GN_tdc_1_PE_2_SV_
MetazoaNematodaCaenorhabditiselegans_4392869_TDC1_CAEEL_Tyrosine_decarboxylase_OS_Caenorhabditis_elegans_OX_6239_GN_tdc_1_PE_2_SV_
MetazoaNematodaCaenorhabditiselegans_4397803_DDC_CAEEL_Probable_aromatic_L_amino_acid_decarboxylase_OS_Caenorhabditis_elegans_OX_6239_GN_hdl_1_PE_2_SV_
MetazoaArthropodaDrosophilamelanogaster_241048_TDC1_CAEEL_Tyrosine_decarboxylase_OS_Caenorhabditis_elegans_OX_6239_GN_tdc_1_PE_2_SV_
MetazoaArthropodaStegodyphusmimosarum_1156292_TDC1_CAEEL_Tyrosine_decarboxylase_OS_Caenorhabditis_elegans_OX_6239_GN_tdc_1_PE_2_SV_
MetazoaCnidariaParamuriceabiscaya_440361_TDC1_CAEEL_Tyrosine_decarboxylase_OS_Caenorhabditis_elegans_OX_6239_GN_tdc_1_PE_2_SV_
MetazoaCnidariaParamuriceabiscaya_452654_TDC1_CAEEL_Tyrosine_decarboxylase_OS_Caenorhabditis_elegans_OX_6239_GN_tdc_1_PE_2_SV_
MetazoaAnnelidaHelobdellarobusta_107383_TDC1_CAEEL_Tyrosine_decarboxylase_OS_Caenorhabditis_elegans_OX_6239_GN_tdc_1_PE_2_SV_
MetazoaAnnelidaHelobdellarobusta_107399_TDC1_CAEEL_Tyrosine_decarboxylase_OS_Caenorhabditis_elegans_OX_6239_GN_tdc_1_PE_2_SV_
MetazoaAnnelidaHelobdellarobusta_85647_TDC1_CAEEL_Tyrosine_decarboxylase_OS_Caenorhabditis_elegans_OX_6239_GN_tdc_1_PE_2_SV_
MetazoaAnnelidaEP00103Capitellateleta_77787_TDC1_CAEEL_Tyrosine_decarboxylase_OS_Caenorhabditis_elegans_OX_6239_GN_tdc_1_PE_2_SV_
MetazoaMolluscaEP00105Crassostreagigas_4135598_TDC1_CAEEL_Tyrosine_decarboxylase_OS_Caenorhabditis_elegans_OX_6239_GN_tdc_1_PE_2_SV_
MetazoaMolluscaEP00107Lottiagigantea_4181687_TDC1_CAEEL_Tyrosine_decarboxylase_OS_Caenorhabditis_elegans_OX_6239_GN_tdc_1_PE_2_SV_
MetazoaBrachiopodaLingulaunguis_1292633_TDC1_CAEEL_Tyrosine_decarboxylase_OS_Caenorhabditis_elegans_OX_6239_GN_tdc_1_PE_2_SV_
MetazoaRotiferaAdinetavaga_5173756_TDC1_CAEEL_Tyrosine_decarboxylase_OS_Caenorhabditis_elegans_OX_6239_GN_tdc_1_PE_2_SV_
MetazoaRotiferaAdinetavaga_5193353_TDC1_CAEEL_Tyrosine_decarboxylase_OS_Caenorhabditis_elegans_OX_6239_GN_tdc_1_PE_2_SV_
MetazoaRotiferaAdinetavaga_5191098_TDC1_CAEEL_Tyrosine_decarboxylase_OS_Caenorhabditis_elegans_OX_6239_GN_tdc_1_PE_2_SV_
MetazoaRotiferaAdinetavaga_5198272_TDC1_CAEEL_Tyrosine_decarboxylase_OS_Caenorhabditis_elegans_OX_6239_GN_tdc_1_PE_2_SV_
MetazoaRotiferaAdinetavaga_5177206_TDC1_CAEEL_Tyrosine_decarboxylase_OS_Caenorhabditis_elegans_OX_6239_GN_tdc_1_PE_2_SV_
MetazoaRotiferaAdinetavaga_5199046_TDC1_CAEEL_Tyrosine_decarboxylase_OS_Caenorhabditis_elegans_OX_6239_GN_tdc_1_PE_2_SV_
MetazoaEchinodermataAcanthasterplanci_3780893_TDC1_CAEEL_Tyrosine_decarboxylase_OS_Caenorhabditis_elegans_OX_6239_GN_tdc_1_PE_2_SV_
MetazoaEchinodermataAcanthasterplanci_3780904_TDC1_CAEEL_Tyrosine_decarboxylase_OS_Caenorhabditis_elegans_OX_6239_GN_tdc_1_PE_2_SV_
MetazoaEchinodermataAcanthasterplanci_3780883_TDC1_CAEEL_Tyrosine_decarboxylase_OS_Caenorhabditis_elegans_OX_6239_GN_tdc_1_PE_2_SV_
MetazoaEchinodermataAcanthasterplanci_3795282_TDC1_CAEEL_Tyrosine_decarboxylase_OS_Caenorhabditis_elegans_OX_6239_GN_tdc_1_PE_2_SV_
MetazoaEchStrongylocentrotuspurpuratus_3812223_TDC1_CAEEL_Tyrosine_decarboxylase_OS_Caenorhabditis_elegans_OX_6239_GN_tdc_1_PE_2_SV_
MetazoaEchStrongylocentrotuspurpuratus_3830333_TDC1_CAEEL_Tyrosine_decarboxylase_OS_Caenorhabditis_elegans_OX_6239_GN_tdc_1_PE_2_SV_
MetazoaAcoelaHofsteniamiamia_14659_TDC1_CAEEL_Tyrosine_decarboxylase_OS_Caenorhabditis_elegans_OX_6239_GN_tdc_1_PE_2_SV_
MetazoaVertebrateHomosapiens_6455273_DDC_HUMAN_Aromatic_L_amino_acid_decarboxylase_OS_Homo_sapiens_OX_9606_GN_DDC_PE_1_SV_
MetazoaVertebrateMusmusculus_6531652_DDC_MOUSE_Aromatic_L_amino_acid_decarboxylase_OS_Mus_musculus_OX_10090_GN_Ddc_PE_1_SV_
MetazoaVertebrateMonodelphisdomestica_6519594_DDC_HUMAN_Aromatic_L_amino_acid_decarboxylase_OS_Homo_sapiens_OX_9606_GN_DDC_PE_1_SV_
MetazoaVertebrateAnoliscarolinensis_6312125_DDC_HUMAN_Aromatic_L_amino_acid_decarboxylase_OS_Homo_sapiens_OX_9606_GN_DDC_PE_1_SV_
MetazoaVertebrateGallusgallus_6420045_DDC_HUMAN_Aromatic_L_amino_acid_decarboxylase_OS_Homo_sapiens_OX_9606_GN_DDC_PE_1_SV_
MetazoaVertebrateEP00079Xenopustropicalis_6369136_DDC_CAVPO_Aromatic_L_amino_acid_decarboxylase_OS_Cavia_porcellus_OX_10141_GN_DDC_PE_2_SV_
MetazoaVertebrateEP00079Xenopustropicalis_6379144_DDC_HUMAN_Aromatic_L_amino_acid_decarboxylase_OS_Homo_sapiens_OX_9606_GN_DDC_PE_1_SV_
MetazoaVertebrateEP00079Xenopustropicalis_6344276_DDC_CAVPO_Aromatic_L_amino_acid_decarboxylase_OS_Cavia_porcellus_OX_10141_GN_DDC_PE_2_SV_
MetazoaVertebrateLatimeriachalumnae_5777083_DDC_CAVPO_Aromatic_L_amino_acid_decarboxylase_OS_Cavia_porcellus_OX_10141_GN_DDC_PE_2_SV_
MetazoaVertebrateLatimeriachalumnae_5784828_DDC_CAVPO_Aromatic_L_amino_acid_decarboxylase_OS_Cavia_porcellus_OX_10141_GN_DDC_PE_2_SV_
MetazoaVertebrateLatimeriachalumnae_5801107_DDC_RAT_Aromatic_L_amino_acid_decarboxylase_OS_Rattus_norvegicus_OX_10116_GN_Ddc_PE_1_SV_
MetazoaVertebrateEP00066Takifugurubripes_5687147_DDC_RAT_Aromatic_L_amino_acid_decarboxylase_OS_Rattus_norvegicus_OX_10116_GN_Ddc_PE_1_SV_
MetazoaVertebrateEP00067Daniorerio_5729828_DDC_CAVPO_Aromatic_L_amino_acid_decarboxylase_OS_Cavia_porcellus_OX_10141_GN_DDC_PE_2_SV_
MetazoaVertebrateCallorhinchusmilii_5514912_DDC_CAVPO_Aromatic_L_amino_acid_decarboxylase_OS_Cavia_porcellus_OX_10141_GN_DDC_PE_2_SV_
MetazoaCephaBranchiostomabelcheri_2831796_DDC_RAT_Aromatic_L_amino_acid_decarboxylase_OS_Rattus_norvegicus_OX_10116_GN_Ddc_PE_1_SV_
MetazoaCephaBranchiostomabelcheri_2831797_DDC_RAT_Aromatic_L_amino_acid_decarboxylase_OS_Rattus_norvegicus_OX_10116_GN_Ddc_PE_1_SV_
MetazoaCephaBranchiostomabelcheri_2831795_DDC_RAT_Aromatic_L_amino_acid_decarboxylase_OS_Rattus_norvegicus_OX_10116_GN_Ddc_PE_1_SV_
MetazoaCephaBranchiostomabelcheri_2825770_DDC_RAT_Aromatic_L_amino_acid_decarboxylase_OS_Rattus_norvegicus_OX_10116_GN_Ddc_PE_1_SV_
MetazoaEchStrongylocentrotuspurpuratus_3821557_DDC_RAT_Aromatic_L_amino_acid_decarboxylase_OS_Rattus_norvegicus_OX_10116_GN_Ddc_PE_1_SV_
MetazoaEchStrongylocentrotuspurpuratus_3832868_DDC_RAT_Aromatic_L_amino_acid_decarboxylase_OS_Rattus_norvegicus_OX_10116_GN_Ddc_PE_1_SV_
MetazoaEchStrongylocentrotuspurpuratus_3825924_DDC_RAT_Aromatic_L_amino_acid_decarboxylase_OS_Rattus_norvegicus_OX_10116_GN_Ddc_PE_1_SV_
MetazoaEchStrongylocentrotuspurpuratus_3837227_DDC_RAT_Aromatic_L_amino_acid_decarboxylase_OS_Rattus_norvegicus_OX_10116_GN_Ddc_PE_1_SV_
MetazoaEchStrongylocentrotuspurpuratus_3813930_DDC_RAT_Aromatic_L_amino_acid_decarboxylase_OS_Rattus_norvegicus_OX_10116_GN_Ddc_PE_1_SV_
MetazoaEchinodermataAcanthasterplanci_3797045_DDC_RAT_Aromatic_L_amino_acid_decarboxylase_OS_Rattus_norvegicus_OX_10116_GN_Ddc_PE_1_SV_
MetazoaAnnelidaEP00103Capitellateleta_71738_DDC_RAT_Aromatic_L_amino_acid_decarboxylase_OS_Rattus_norvegicus_OX_10116_GN_Ddc_PE_1_SV_
MetazoaAnnelidaEP00103Capitellateleta_72591_DDC_MOUSE_Aromatic_L_amino_acid_decarboxylase_OS_Mus_musculus_OX_10090_GN_Ddc_PE_1_SV_
MetazoaAnnelidaEP00103Capitellateleta_54471_DDC_RAT_Aromatic_L_amino_acid_decarboxylase_OS_Rattus_norvegicus_OX_10116_GN_Ddc_PE_1_SV_
MetazoaAnnelidaEP00103Capitellateleta_78833_DDC_RAT_Aromatic_L_amino_acid_decarboxylase_OS_Rattus_norvegicus_OX_10116_GN_Ddc_PE_1_SV_
MetazoaAnnelidaHelobdellarobusta_101516_DDC_DROSI_Aromatic_L_amino_acid_decarboxylase_OS_Drosophila_simulans_OX_7240_GN_Ddc_PE_3_SV_
MetazoaArthropodaDaphniapulex_202720_DDC_RAT_Aromatic_L_amino_acid_decarboxylase_OS_Rattus_norvegicus_OX_10116_GN_Ddc_PE_1_SV_
MetazoaArthropodaDaphniapulex_216987_DDC_DROSI_Aromatic_L_amino_acid_decarboxylase_OS_Drosophila_simulans_OX_7240_GN_Ddc_PE_3_SV_
MetazoaArthropodaDaphniapulex_191394_DDC_DROME_Aromatic_L_amino_acid_decarboxylase_OS_Drosophila_melanogaster_OX_7227_GN_Ddc_PE_1_SV_
MetazoaArthropodaDrosophilamelanogaster_243095_DDC_DROME_Aromatic_L_amino_acid_decarboxylase_OS_Drosophila_melanogaster_OX_7227_GN_Ddc_PE_1_SV_
MetazoaNematodaCaenorhabditiselegans_4377033_DDC_RAT_Aromatic_L_amino_acid_decarboxylase_OS_Rattus_norvegicus_OX_10116_GN_Ddc_PE_1_SV_
MetazoaNematodaCaenorhabditiselegans_4377034_DDC_RAT_Aromatic_L_amino_acid_decarboxylase_OS_Rattus_norvegicus_OX_10116_GN_Ddc_PE_1_SV_
MetazoaNematodaCaenorhabditiselegans_4394830_DDC_RAT_Aromatic_L_amino_acid_decarboxylase_OS_Rattus_norvegicus_OX_10116_GN_Ddc_PE_1_SV_
MetazoaRotiferaAdinetavaga_5177851_DDC_RAT_Aromatic_L_amino_acid_decarboxylase_OS_Rattus_norvegicus_OX_10116_GN_Ddc_PE_1_SV_
MetazoaRotiferaAdinetavaga_5221735_DDC_RAT_Aromatic_L_amino_acid_decarboxylase_OS_Rattus_norvegicus_OX_10116_GN_Ddc_PE_1_SV_
MetazoaBrachiopodaLingulaunguis_1294521_DDC_RAT_Aromatic_L_amino_acid_decarboxylase_OS_Rattus_norvegicus_OX_10116_GN_Ddc_PE_1_SV_
MetazoaBrachiopodaLingulaunguis_1301812_DDC_RAT_Aromatic_L_amino_acid_decarboxylase_OS_Rattus_norvegicus_OX_10116_GN_Ddc_PE_1_SV_
MetazoaBrachiopodaLingulaunguis_1300857_DDC_RAT_Aromatic_L_amino_acid_decarboxylase_OS_Rattus_norvegicus_OX_10116_GN_Ddc_PE_1_SV_
MetazoaBrachiopodaLingulaunguis_1302126_DDC_RAT_Aromatic_L_amino_acid_decarboxylase_OS_Rattus_norvegicus_OX_10116_GN_Ddc_PE_1_SV_
MetazoaBrachiopodaLingulaunguis_1287640_DDC_RAT_Aromatic_L_amino_acid_decarboxylase_OS_Rattus_norvegicus_OX_10116_GN_Ddc_PE_1_SV_
MetazoaBrachiopodaLingulaunguis_1301297_DDC_RAT_Aromatic_L_amino_acid_decarboxylase_OS_Rattus_norvegicus_OX_10116_GN_Ddc_PE_1_SV_
MetazoaMolluscaEP00107Lottiagigantea_4184241_DDC_HUMAN_Aromatic_L_amino_acid_decarboxylase_OS_Homo_sapiens_OX_9606_GN_DDC_PE_1_SV_
MetazoaMolluscaEP00107Lottiagigantea_4200814_DDC_MOUSE_Aromatic_L_amino_acid_decarboxylase_OS_Mus_musculus_OX_10090_GN_Ddc_PE_1_SV_
MetazoaMolluscaEP00107Lottiagigantea_4189237_DDC_RAT_Aromatic_L_amino_acid_decarboxylase_OS_Rattus_norvegicus_OX_10116_GN_Ddc_PE_1_SV_
MetazoaMolluscaEP00105Crassostreagigas_4111608_DDC_RAT_Aromatic_L_amino_acid_decarboxylase_OS_Rattus_norvegicus_OX_10116_GN_Ddc_PE_1_SV_
MetazoaMolluscaEP00105Crassostreagigas_4130788_DDC_DROME_Aromatic_L_amino_acid_decarboxylase_OS_Drosophila_melanogaster_OX_7227_GN_Ddc_PE_1_SV_
MetazoaAcoelaHofsteniamiamia_11732_DDC_RAT_Aromatic_L_amino_acid_decarboxylase_OS_Rattus_norvegicus_OX_10116_GN_Ddc_PE_1_SV_
MetazoaAcoelaHofsteniamiamia_5512_DDC_DROLE_Aromatic_L_amino_acid_decarboxylase__Fragment__OS_Drosophila_lebanonensis_OX_7225_GN_Ddc_PE_3_SV_
MetazoaAcoelaHofsteniamiamia_13207_DDC_RAT_Aromatic_L_amino_acid_decarboxylase_OS_Rattus_norvegicus_OX_10116_GN_Ddc_PE_1_SV_
MetazoaAcoelaHofsteniamiamia_8818_TDC1_CAEEL_Tyrosine_decarboxylase_OS_Caenorhabditis_elegans_OX_6239_GN_tdc_1_PE_2_SV_
MetazoaCnidariaHydramagnapapillata_3242673_DDC_HUMAN_Aromatic_L_amino_acid_decarboxylase_OS_Homo_sapiens_OX_9606_GN_DDC_PE_1_SV_
MetazoaCnidariaHydramagnapapillata_3248325_DDC_RAT_Aromatic_L_amino_acid_decarboxylase_OS_Rattus_norvegicus_OX_10116_GN_Ddc_PE_1_SV_
MetazoaCnidariaHydramagnapapillata_3262809_DDC_RAT_Aromatic_L_amino_acid_decarboxylase_OS_Rattus_norvegicus_OX_10116_GN_Ddc_PE_1_SV_
MetazoaCnidariaHydramagnapapillata_3248644_DDC_CAVPO_Aromatic_L_amino_acid_decarboxylase_OS_Cavia_porcellus_OX_10141_GN_DDC_PE_2_SV_
MetazoaCnidariaHydramagnapapillata_3248938_DDC_HUMAN_Aromatic_L_amino_acid_decarboxylase_OS_Homo_sapiens_OX_9606_GN_DDC_PE_1_SV_
MetazoaCnidariaHydramagnapapillata_3260287_DDC_RAT_Aromatic_L_amino_acid_decarboxylase_OS_Rattus_norvegicus_OX_10116_GN_Ddc_PE_1_SV_
MetazoaCnidariaClytiahemisphaerica_3084150_DDC_RAT_Aromatic_L_amino_acid_decarboxylase_OS_Rattus_norvegicus_OX_10116_GN_Ddc_PE_1_SV_
MetazoaCnidariaClytiahemisphaerica_3089205_DDC_RAT_Aromatic_L_amino_acid_decarboxylase_OS_Rattus_norvegicus_OX_10116_GN_Ddc_PE_1_SV_
MetazoaCnidariaClytiahemisphaerica_3091690_DDC_RAT_Aromatic_L_amino_acid_decarboxylase_OS_Rattus_norvegicus_OX_10116_GN_Ddc_PE_1_SV_
MetazoaCnidariaClytiahemisphaerica_3099137_DDC_RAT_Aromatic_L_amino_acid_decarboxylase_OS_Rattus_norvegicus_OX_10116_GN_Ddc_PE_1_SV_
MetazoaCnidariaCyaneanozakii_17058_DDC_HUMAN_Aromatic_L_amino_acid_decarboxylase_OS_Homo_sapiens_OX_9606_GN_DDC_PE_1_SV_
MetazoaCnidariaCyaneanozakii_17060_DDC_HUMAN_Aromatic_L_amino_acid_decarboxylase_OS_Homo_sapiens_OX_9606_GN_DDC_PE_1_SV_
MetazoaCnidariaCyaneanozakii_17059_DDC_HUMAN_Aromatic_L_amino_acid_decarboxylase_OS_Homo_sapiens_OX_9606_GN_DDC_PE_1_SV_
MetazoaCnidariaTripedaliacystophora_143590_DDC_RAT_Aromatic_L_amino_acid_decarboxylase_OS_Rattus_norvegicus_OX_10116_GN_Ddc_PE_1_SV_
MetazoaCnidariaTripedaliacystophora_143591_DDC_CAVPO_Aromatic_L_amino_acid_decarboxylase_OS_Cavia_porcellus_OX_10141_GN_DDC_PE_2_SV_
MetazoaCnidariaTripedaliacystophora_168614_DDC_RAT_Aromatic_L_amino_acid_decarboxylase_OS_Rattus_norvegicus_OX_10116_GN_Ddc_PE_1_SV_
MetazoaCnidariaLucernariaquadricornis_54675_DDC_RAT_Aromatic_L_amino_acid_decarboxylase_OS_Rattus_norvegicus_OX_10116_GN_Ddc_PE_1_SV_
MetazoaCnidariaLucernariaquadricornis_54677_DHAPP_DROME_3_4_dihydroxyphenylacetaldehyde_synthase_OS_Drosophila_melanogaster_OX_7227_GN_amd_PE_1_SV_
MetazoaCnidariaNematostellavectensis_011323DDC_HUMAN_Aromatic_L_amino_acid_decarboxylase_OS_Homo_sapiens_OX_9606_GN_DDC_PE_1_SV_
MetazoaCnidariaNematostellavectensis_3379964_DDC_HUMAN_Aromatic_L_amino_acid_decarboxylase_OS_Homo_sapiens_OX_9606_GN_DDC_PE_1_SV_
MetazoaCnidariaNematostellavectensis_3380002_DDC_RAT_Aromatic_L_amino_acid_decarboxylase_OS_Rattus_norvegicus_OX_10116_GN_Ddc_PE_1_SV_
MetazoaPlacozoaHoilungiahongkongensis_4527632_DDC_RAT_Aromatic_L_amino_acid_decarboxylase_OS_Rattus_norvegicus_OX_10116_GN_Ddc_PE_1_SV_
MetazoaPlacozoaHoilungiahongkongensis_4527634_DHPAA_AEDAE_3_4_dihydroxyphenylacetaldehyde_synthase_OS_Aedes_aegypti_OX_7159_GN_AAEL010734_PE_1_SV_
MetazoaPlacozoaHoilungiahongkongensis_4527633_DDC_BOVIN_Aromatic_L_amino_acid_decarboxylase_OS_Bos_taurus_OX_9913_GN_DDC_PE_2_SV_
MetazoaPlacozoaEP00114TrichoplaxspH2_4506891_DDC_RAT_Aromatic_L_amino_acid_decarboxylase_OS_Rattus_norvegicus_OX_10116_GN_Ddc_PE_1_SV_
ChoanoflagellataEP00039Didymoecacostata_10871278_TYDC1_ARATH_Tyrosine_decarboxylase_1_OS_Arabidopsis_thaliana_OX_3702_GN_ELI5_PE_1_SV_
IchthyosporeaAmoebidiumparasiticum_14565618_DDC_HUMAN_Aromatic_L_amino_acid_decarboxylase_OS_Homo_sapiens_OX_9606_GN_DDC_PE_1_SV_
PluriformeaCorallochytriumlimacisporum_21621410_DDC_RAT_Aromatic_L_amino_acid_decarboxylase_OS_Rattus_norvegicus_OX_10116_GN_Ddc_PE_1_SV_
FilastreaCapsasporaowczarzakiUP000008743_12982165_DDC_CAVPO_Aromatic_L_amino_acid_decarboxylase_OS_Cavia_porcellus_OX_10141_GN_DDC_PE_2_SV_
ChoanoflagellataEP00037Helgoecanana_10812737_TYDC1_ARATH_Tyrosine_decarboxylase_1_OS_Arabidopsis_thaliana_OX_3702_GN_ELI5_PE_1_SV_
ChoanoflagellataEP00039Didymoecacostata_10856214_DHAPP_DROME_3_4_dihydroxyphenylacetaldehyde_synthase_OS_Drosophila_melanogaster_OX_7227_GN_amd_PE_1_SV_
PluriformeaEP00128Syssomonasmultiformis_21640844_TDC2_ORYSJ_Tryptophan_decarboxylase_2_OS_Oryza_sativa_subsp__japonica_OX_39947_GN_TDC2_PE_1_SV_
ChoanoflagellataMylnosigafluctuans_11117312_DDC_RAT_Aromatic_L_amino_acid_decarboxylase_OS_Rattus_norvegicus_OX_10116_GN_Ddc_PE_1_SV_
FungiFusariumoxysporumUP000002489_13360820_DDC_CAVPO_Aromatic_L_amino_acid_decarboxylase_OS_Cavia_porcellus_OX_10141_GN_DDC_PE_2_SV_
FungiFusariumoxysporumUP000002489_13367840_DDC_CAVPO_Aromatic_L_amino_acid_decarboxylase_OS_Cavia_porcellus_OX_10141_GN_DDC_PE_2_SV_
FungiAspergillusnigerUP000068243_13132737_DDC_RAT_Aromatic_L_amino_acid_decarboxylase_OS_Rattus_norvegicus_OX_10116_GN_Ddc_PE_1_SV_
FungiAgaricusbisporusUP000008493_13110407_TDC2_ORYSJ_Tryptophan_decarboxylase_2_OS_Oryza_sativa_subsp__japonica_OX_39947_GN_TDC2_PE_1_SV_
FungiEP00150Ustilagomaydis_13256967_DDC_CAVPO_Aromatic_L_amino_acid_decarboxylase_OS_Cavia_porcellus_OX_10141_GN_DDC_PE_2_SV_
RotosphaeridaEP00160Parvulariaatlantis_23171472_TDC2_ORYSJ_Tryptophan_decarboxylase_2_OS_Oryza_sativa_subsp__japonica_OX_39947_GN_TDC2_PE_1_SV_
RotosphaeridaEP00160Parvulariaatlantis_23171473_TDC2_ORYSJ_Tryptophan_decarboxylase_2_OS_Oryza_sativa_subsp__japonica_OX_39947_GN_TDC2_PE_1_SV_
RotosphaeridaEP00160Parvulariaatlantis_23171476_DCHS_DROME_Histidine_decarboxylase_OS_Drosophila_melanogaster_OX_7227_GN_Hdc_PE_2_SV_
RotosphaeridaFonticulaalbaUP000030693_23194422_DDC_CAVPO_Aromatic_L_amino_acid_decarboxylase_OS_Cavia_porcellus_OX_10141_GN_DDC_PE_2_SV_
0.24

4J.

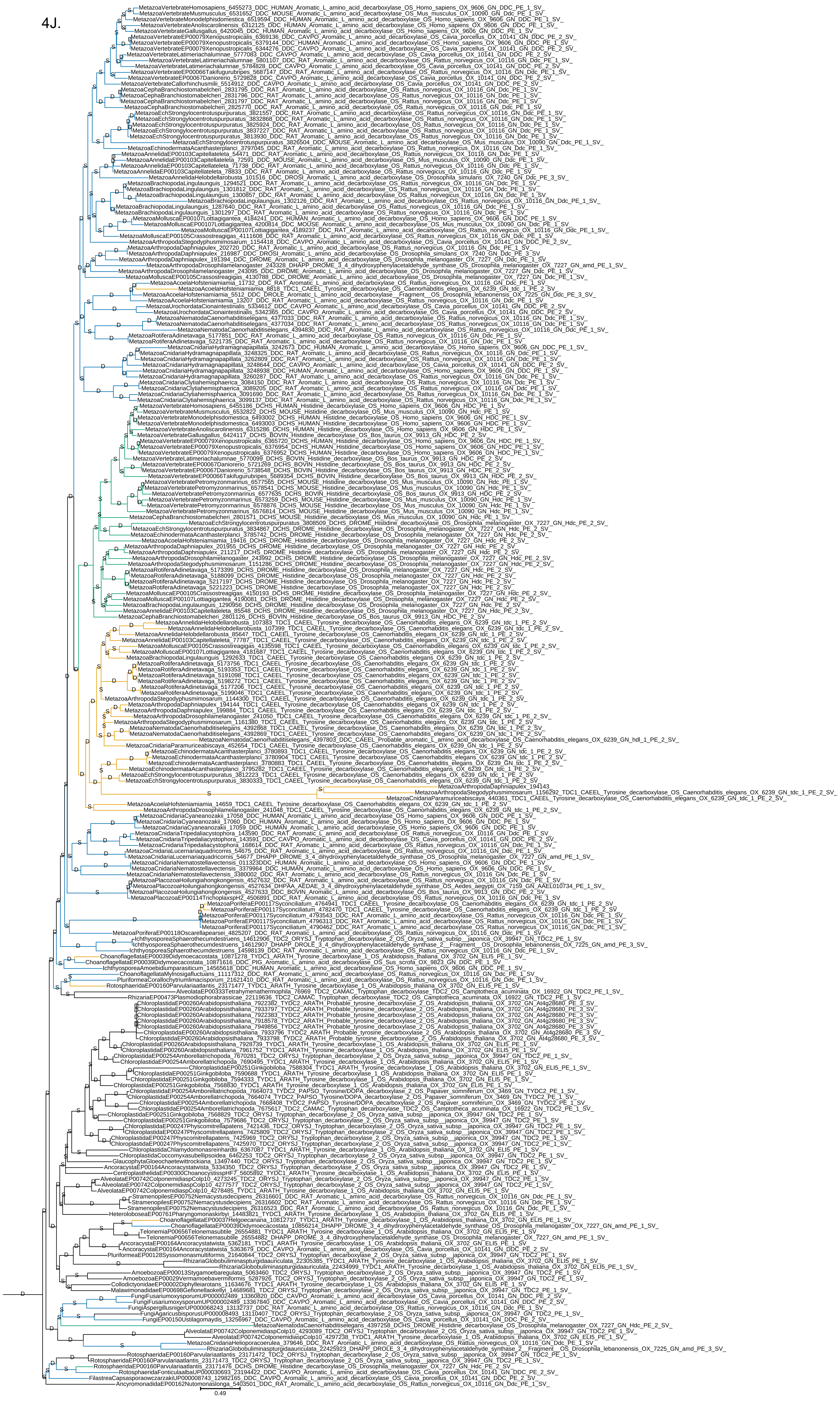

Supplementary Figure 5: Analysis of BHs. A) TBE tree with TBE score supports. B) Maximum likelihood tree with ultrafast bootstrap proportion supports. C) Maximum likelihood tree including all eukaryotic sequences with ultrafast bootstrap proportion supports. D) TBE tree reconciliation using sponge-first hypothesis. E) UFB tree reconciliation using sponge-first hypothesis. F) TBE tree reconciliation using ctenophore-first hypothesis G) UFB tree reconciliation using ctenophore-first hypothesis. H) Reconciliation of UFB tree including all eukaryotic sequences. Nodes in reconciled trees indicated as speciation events (S) or duplication events (D). All trees shown as rooted, non-reconciled trees rooted arbitrarily for visualisation. BH = beta hydroxylase, TBE = transfer bootstrap expectation, UFB = ultrafast bootstrap. Branches colour coded by sequence annotation; blue = dopamine beta hydroxylase; green = tyramine beta hydroxylase; orange = monooxygenase DBH-like; and black = others and non-opithokont sequences. Source Data are available at [https://figshare.le.ac.uk/articles/dataset/Monoamine_Neuromodulation_is_a_Bilaterian_Innovation_Results/20391477]; in Results/GeneTrees labelled OG_BH_TBE.tbe.tree and OG_BH_UFB.treefile; in Results/GeneTrees/Eukaryotes_GeneTrees labelled OG_BH_EUKIQ.treefile; in Results/Reconciliation/SpongeFirst labelled OG_BH_TBE and OG_BH_UFB; in Results/Reconciliation/CtenophoreFirst labelled OG_BH_TBE and OG_BH_UFB; and in Results/Reconciliation/Eukaryote labelled OG_BH.

5A.

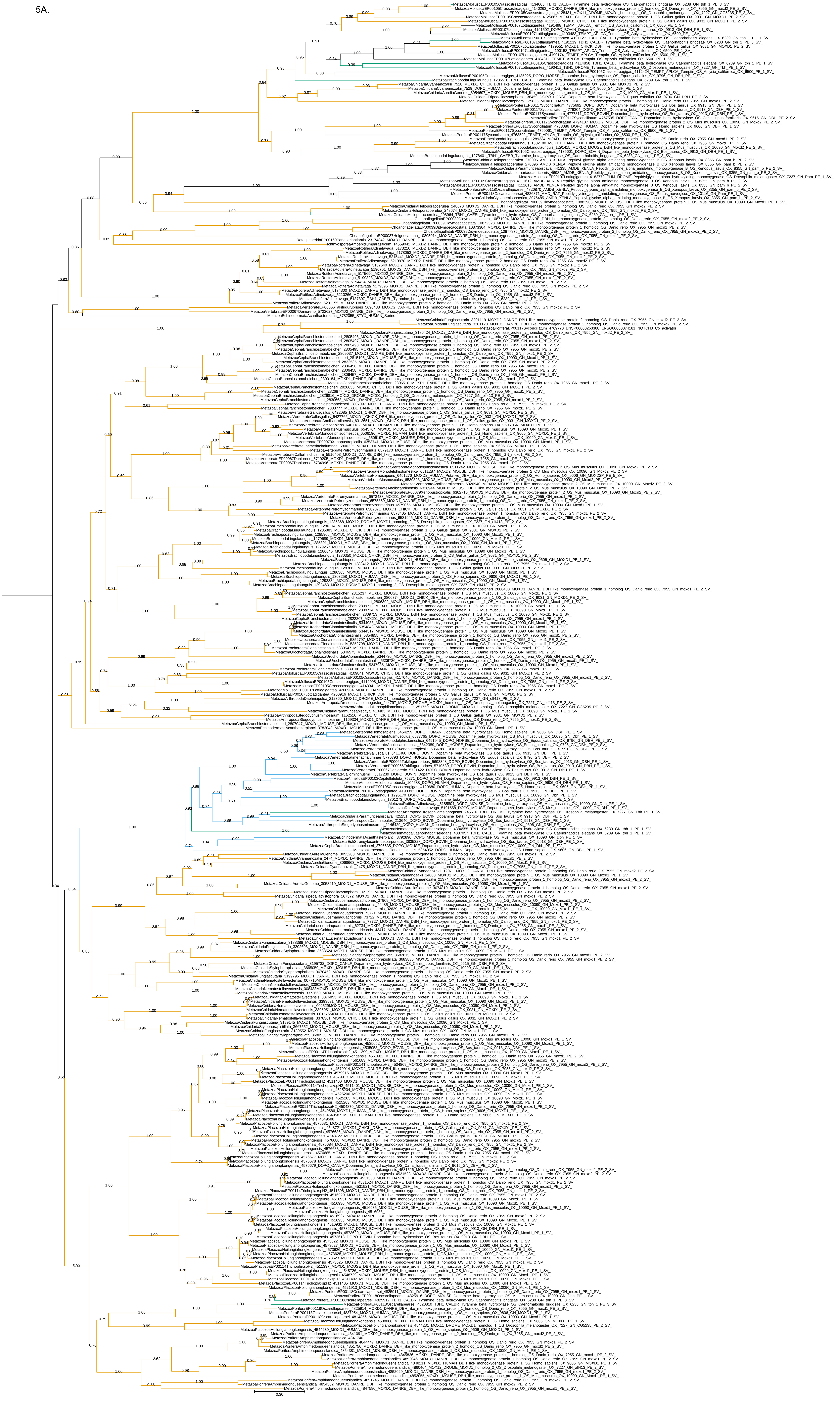

5B.

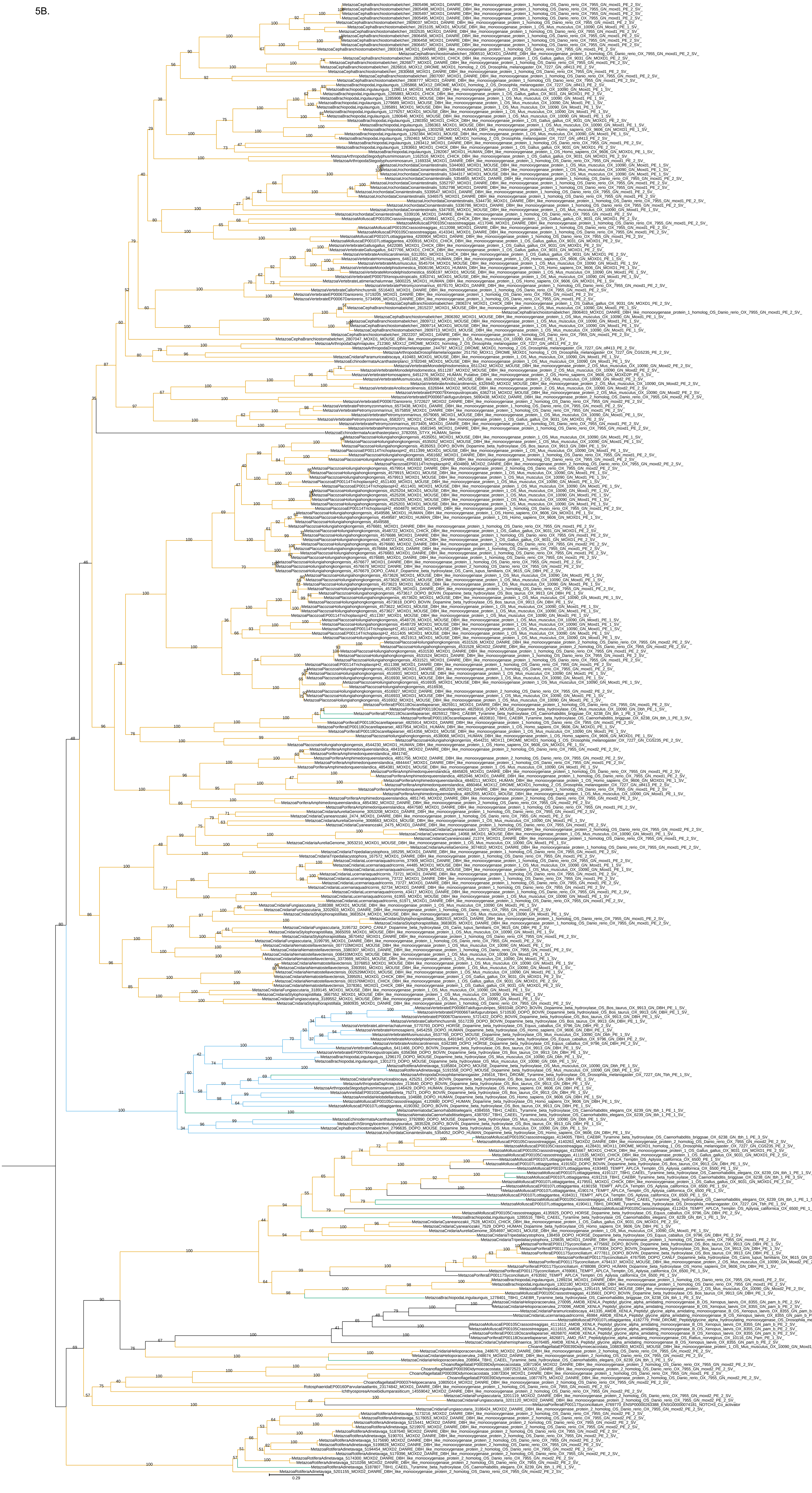

5C.

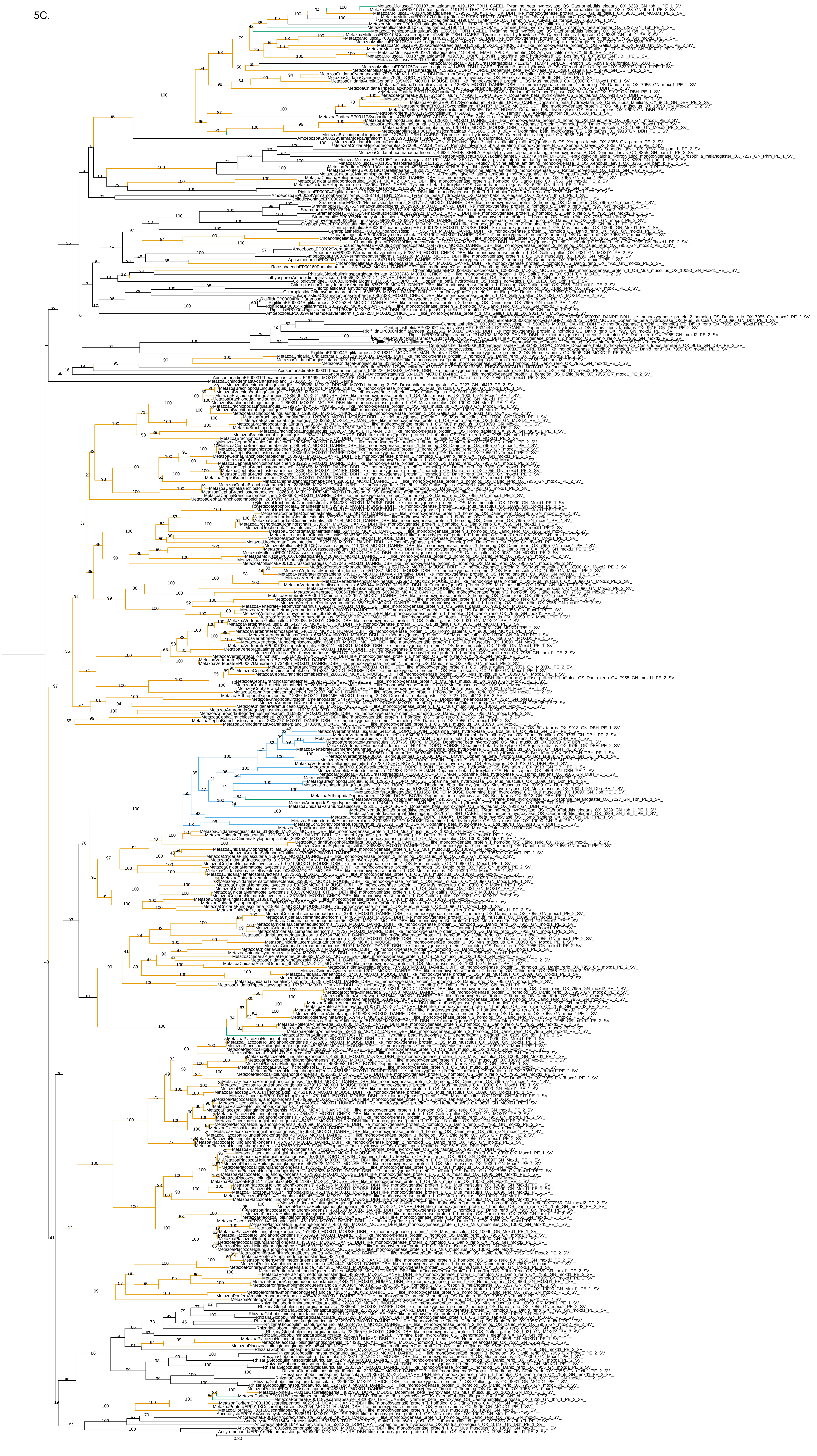

5D.

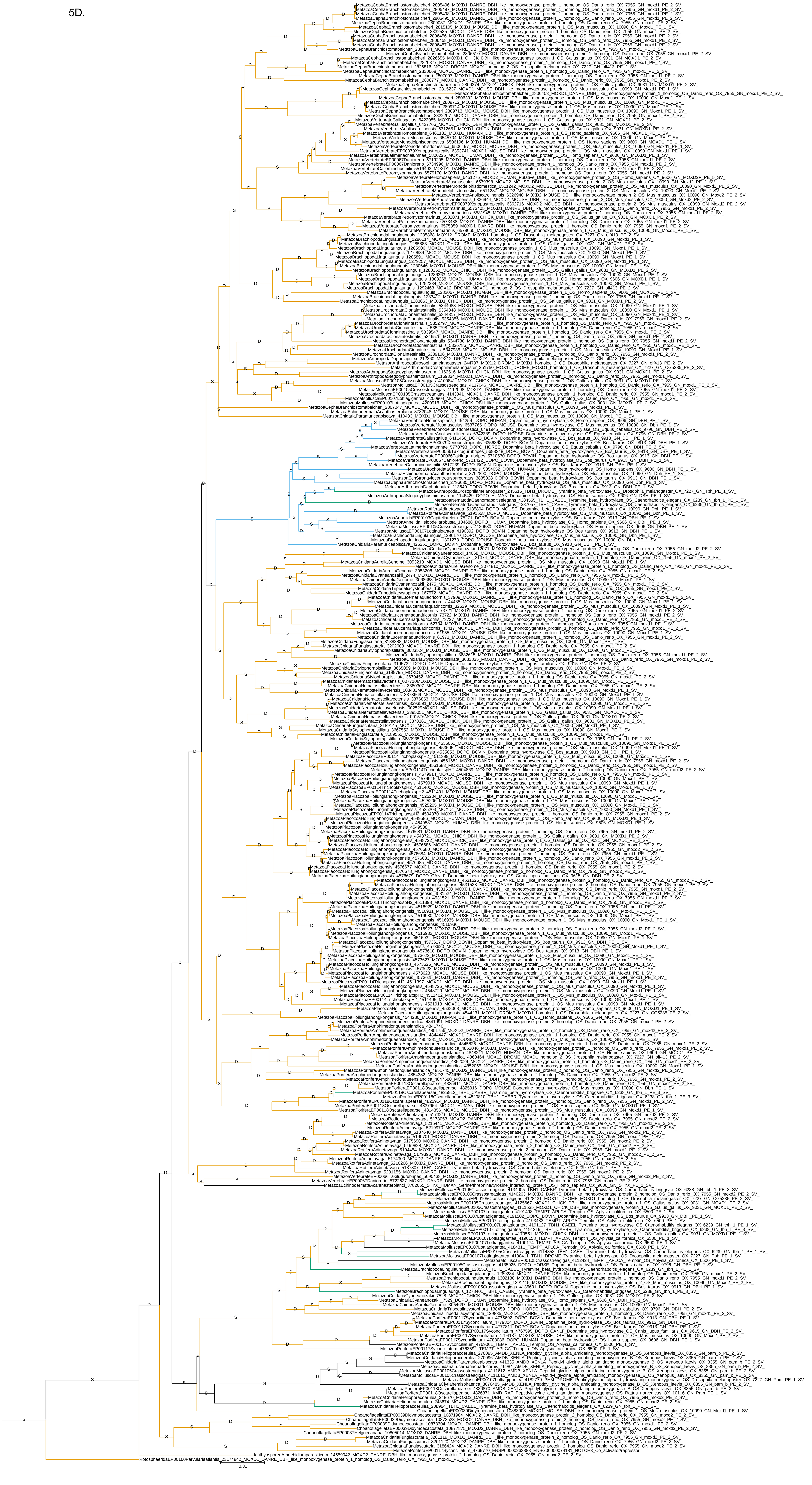

5E.

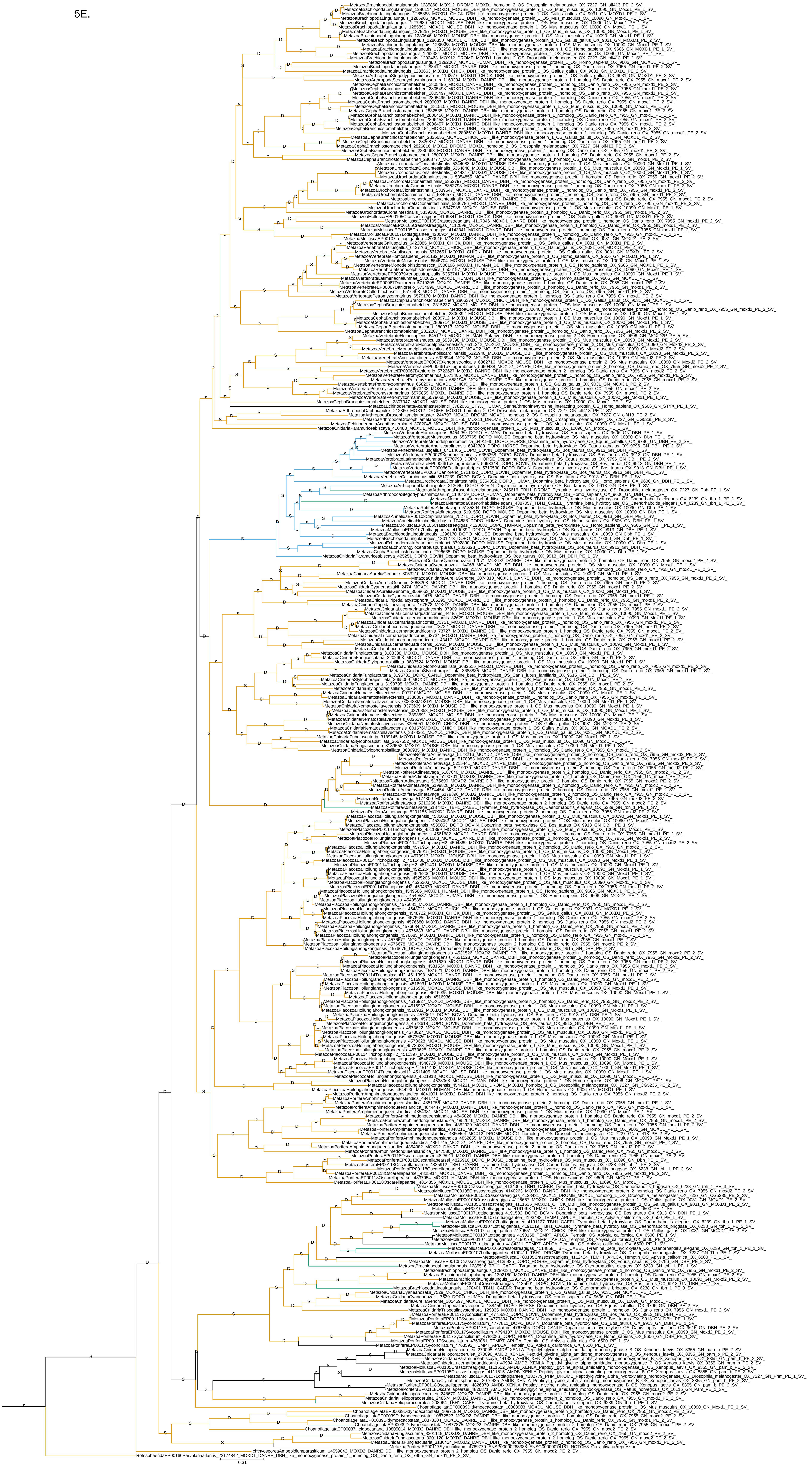

5F.

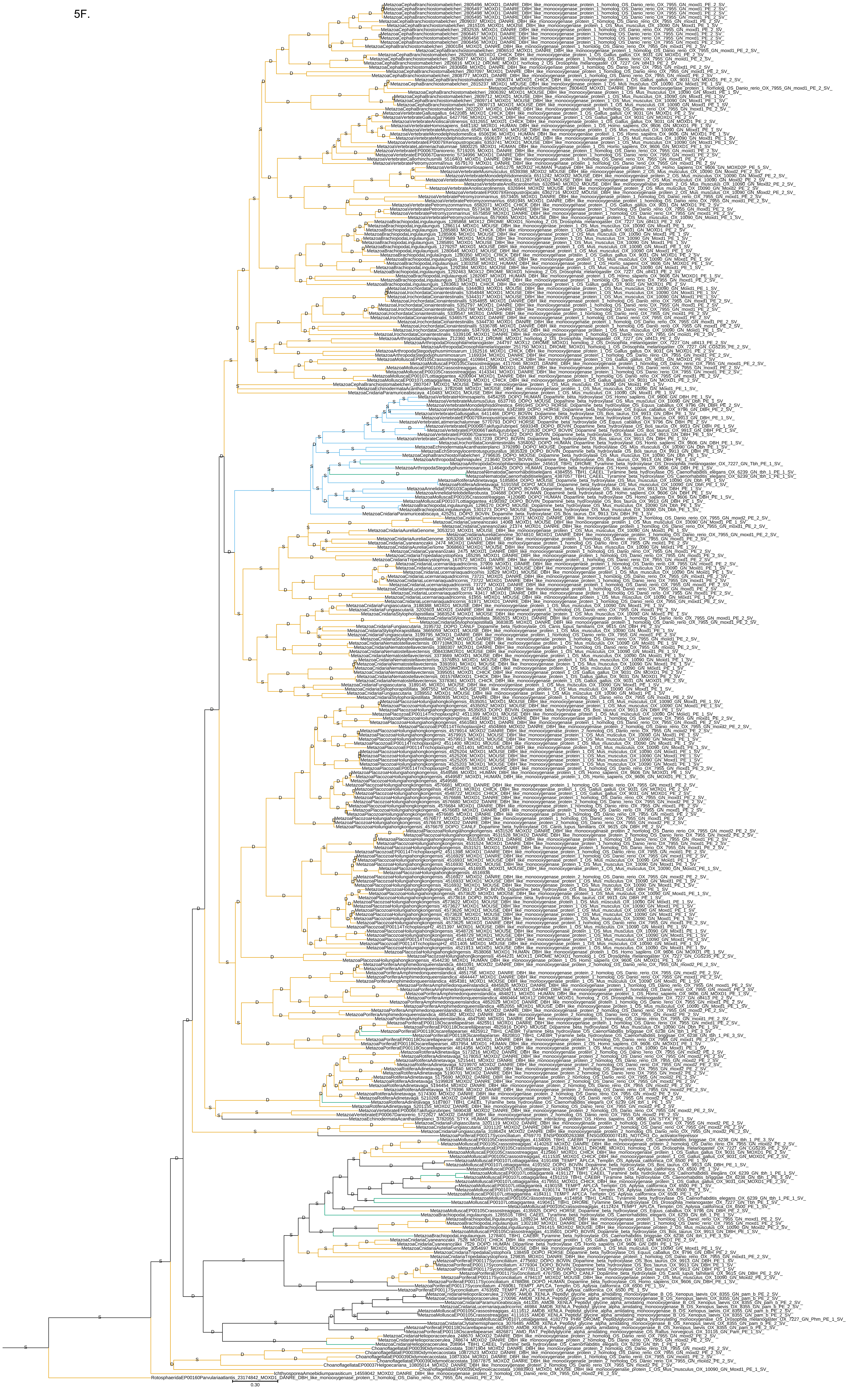

5G.

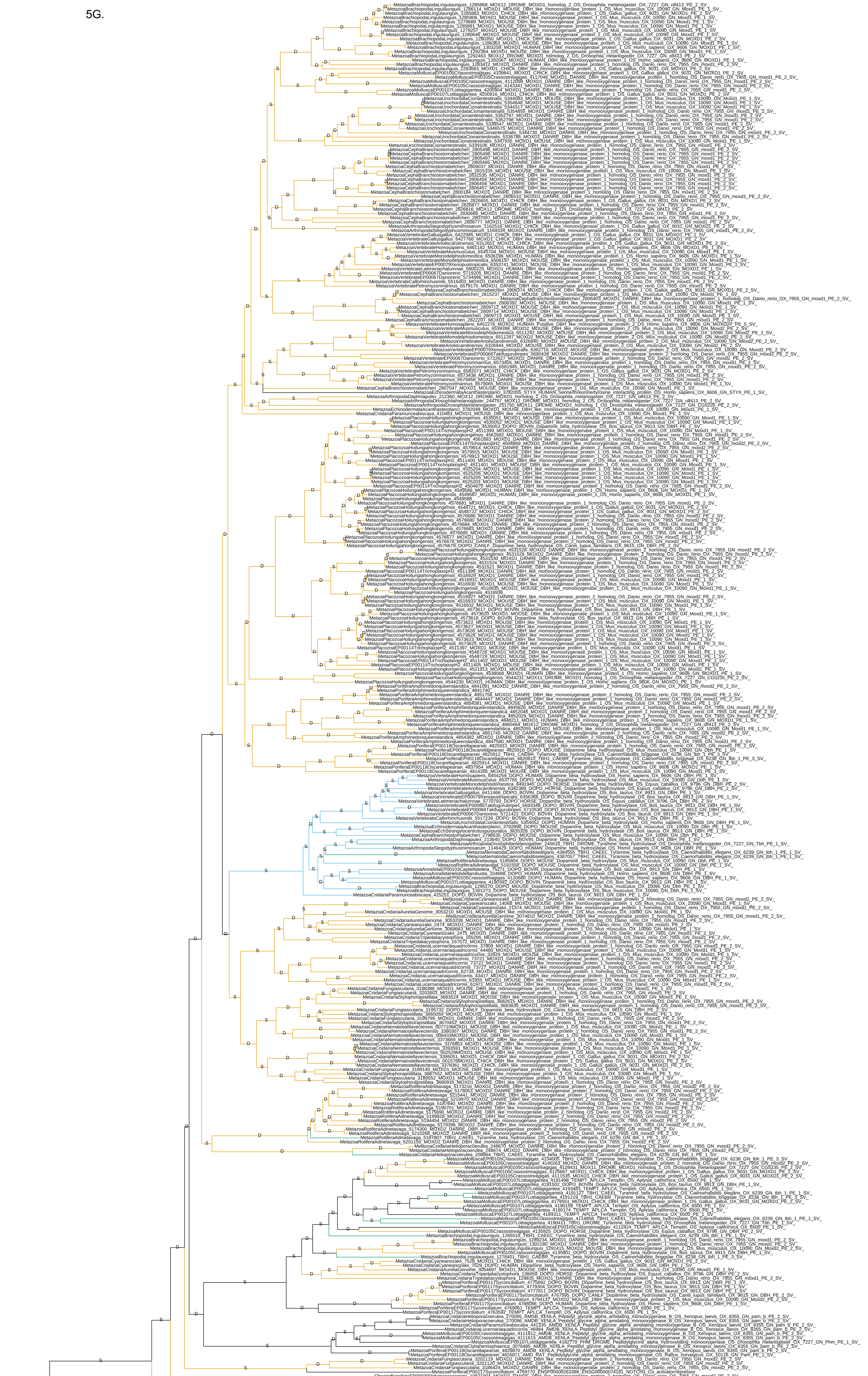

5H.

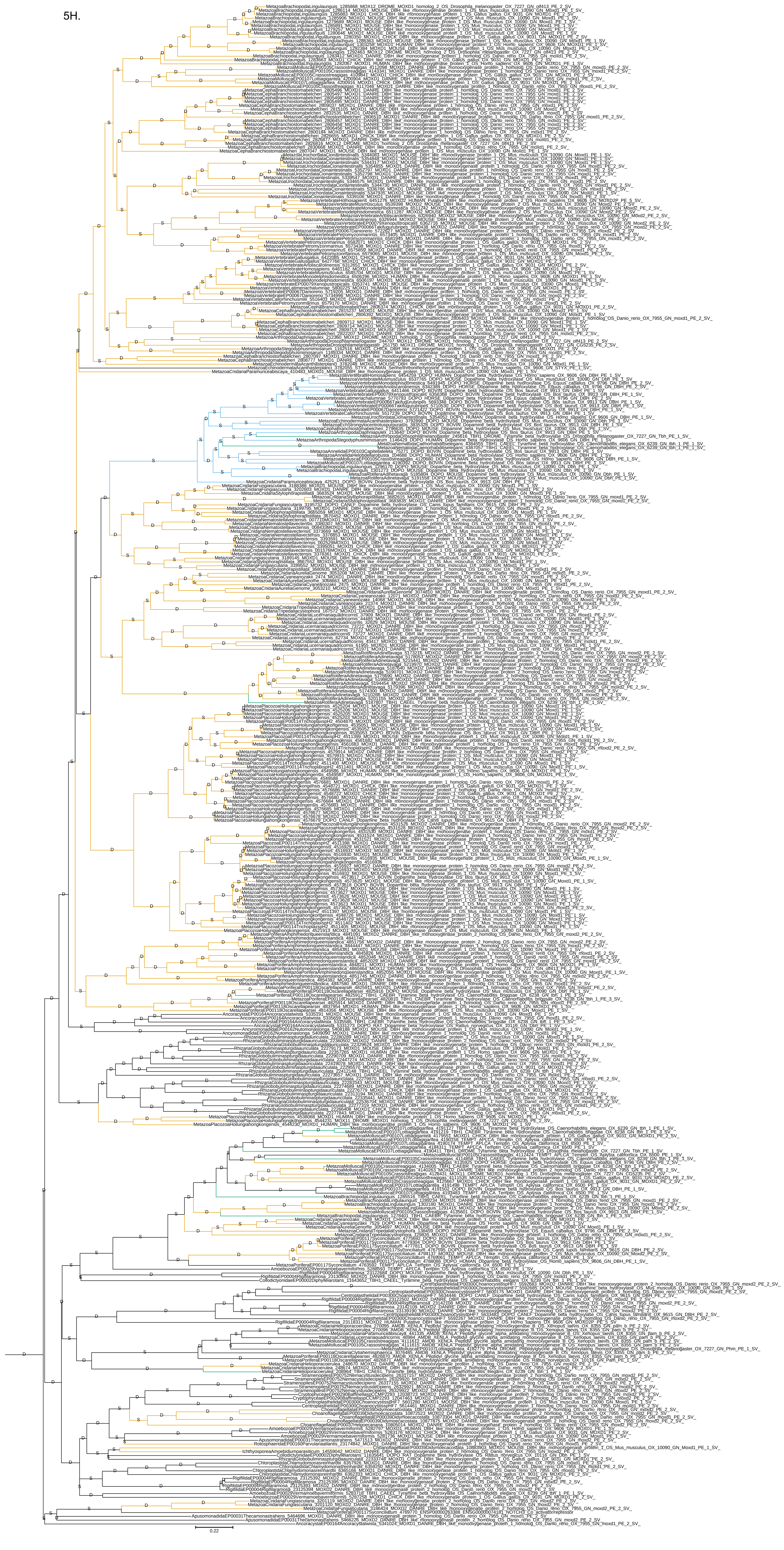

Supplementary Figure 6: Analysis of PNMTs. A) TBE tree with TBE score supports. B) Maximum likelihood tree with ultrafast bootstrap proportion supports. C) Maximum likelihood tree including all eukaryotic sequences with ultrafast bootstrap proportion supports. D) Reconciliation using sponge-first hypothesis. E) Reconciliation using ctenophore-first hypothesis. F) Reconciliation of the UFB tree including all eukaryotic sequences. Nodes in reconciled trees indicated as speciation events (S) or duplication events (D). All trees shown as rooted, non-reconciled trees rooted arbitrarily for visualisation. PNMT = phenylethanolamine-N-methyltransferase, TBE = transfer bootstrap expectation, UFB = ultrafast bootstrap. Branches colour coded by sequence annotation; blue = S-adenosyl methionine methyltransferase; purple = phenylethanolamine-N-methyltransferase; orange = indolethylamine-N-methyltransferase/nicotinamide-N-methyltransferase; and black = others and non-opithokont sequences. Source Data are available at [https://figshare.le.ac.uk/articles/dataset/Monoamine_Neuromodulation_is_a_Bilaterian_Innovation_Results/20391477]; in Results/GeneTrees labelled OG_PNMT_TBE.tbe.tree and OG_PNMT_UFB.treefile; in Results/GeneTrees/Eukaryotes_GeneTrees labelled OG_PNMT_EUKIQ.treefile; in Results/Reconciliation/SpongeFirst labelled OG_PNMT; in Results/Reconciliation/CtenophoreFirst labelled OG_PNMT; and in Results/Reconciliation/Eukaryote labelled OG_PNMT.

6A.

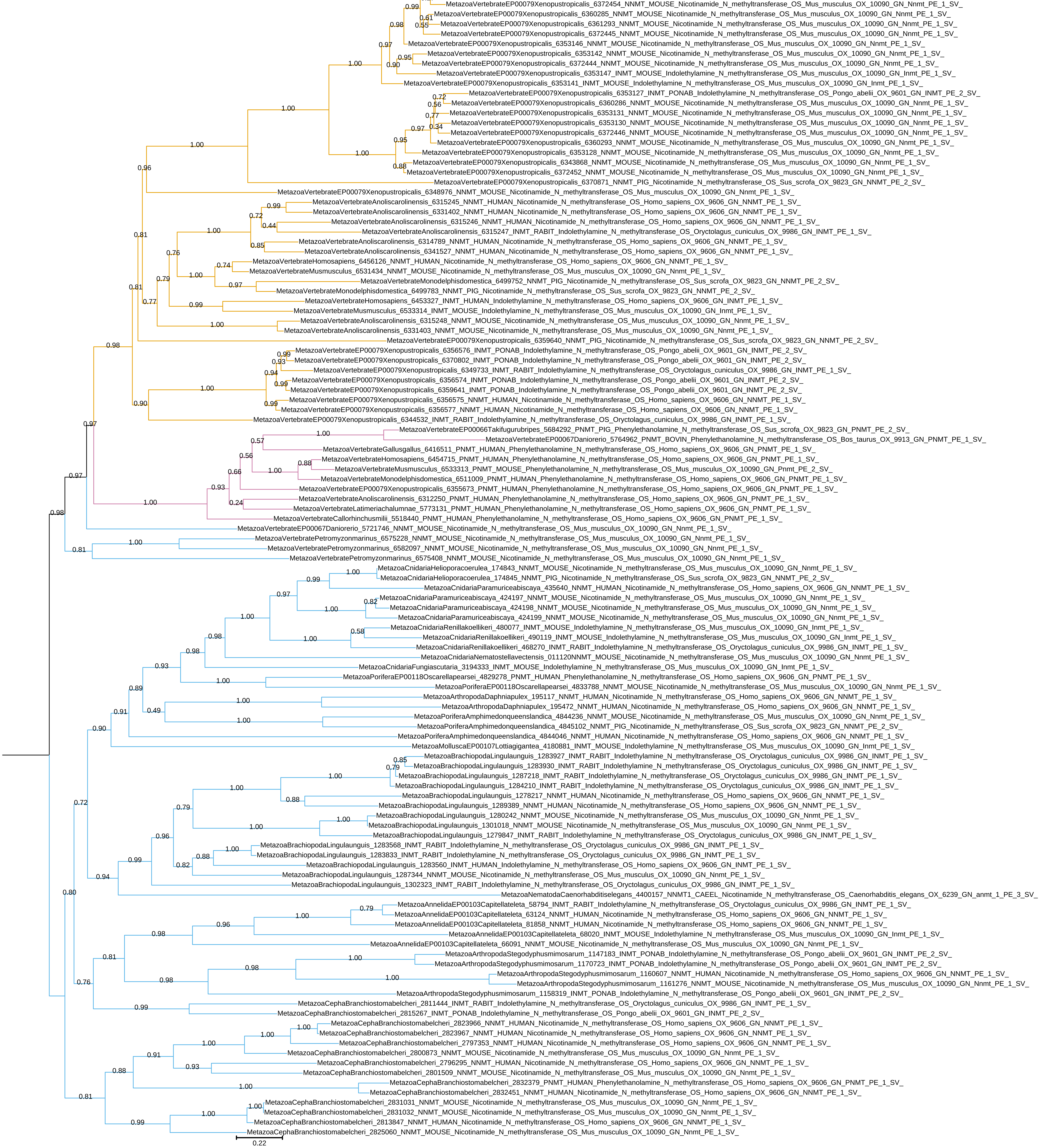

MetazoaVertebrateEP00079Xenopustropicalis_6348369_NNMT_HUMAN_Nicotinamide_N_methyltransferase_OS_Homo_sapiens_OX_9606_GN_NNMT_PE_1_SV_
MetazoaVertebrateEP00079Xenopustropicalis_6372449_NNMT_MOUSE_Nicotinamide_N_methyltransferase_OS_Mus_musculus_OX_10090_GN_Nnmt_PE_1_SV_
MetazoaVertebrateEP00079Xenopustropicalis_6372453_NNMT_HUMAN_Nicotinamide_N_methyltransferase_OS_Homo_sapiens_OX_9606_GN_NNMT_PE_1_SV_
MetazoaVertebrateEP00079Xenopustropicalis_6372454_NNMT_MOUSE_Nicotinamide_N_methyltransferase_OS_Mus_musculus_OX_10090_GN_Nnmt_PE_1_SV_
MetazoaVertebrateEP00079Xenopustropicalis_6360285_NNMT_MOUSE_Nicotinamide_N_methyltransferase_OS_Mus_musculus_OX_10090_GN_Nnmt_PE_1_SV_
MetazoaVertebrateEP00079Xenopustropicalis_6361293_NNMT_MOUSE_Nicotinamide_N_methyltransferase_OS_Mus_musculus_OX_10090_GN_Nnmt_PE_1_SV_
MetazoaVertebrateEP00079Xenopustropicalis_6372445_NNMT_MOUSE_Nicotinamide_N_methyltransferase_OS_Mus_musculus_OX_10090_GN_Nnmt_PE_1_SV_
MetazoaVertebrateEP00079Xenopustropicalis_6353146_NNMT_MOUSE_Nicotinamide_N_methyltransferase_OS_Mus_musculus_OX_10090_GN_Nnmt_PE_1_SV_
MetazoaVertebrateEP00079Xenopustropicalis_6353142_NNMT_MOUSE_Nicotinamide_N_methyltransferase_OS_Mus_musculus_OX_10090_GN_Nnmt_PE_1_SV_
MetazoaVertebrateEP00079Xenopustropicalis_6372444_NNMT_MOUSE_Nicotinamide_N_methyltransferase_OS_Mus_musculus_OX_10090_GN_Nnmt_PE_1_SV_
MetazoaVertebrateEP00079Xenopustropicalis_6353147_INMT_MOUSE_Indolethylamine_N_methyltransferase_OS_Mus_musculus_OX_10090_GN_Inmt_PE_1_SV_
MetazoaVertebrateEP00079Xenopustropicalis_6353141_INMT_MOUSE_Indolethylamine_N_methyltransferase_OS_Mus_musculus_OX_10090_GN_Inmt_PE_1_SV_
MetazoaVertebrateEP00079Xenopustropicalis_6353127_INMT_PONAB_Indolethylamine_N_methyltransferase_OS_Pongo_abelii_OX_9601_GN_INMT_PE_2_SV_
MetazoaVertebrateEP00079Xenopustropicalis_6360286_NNMT_MOUSE_Nicotinamide_N_methyltransferase_OS_Mus_musculus_OX_10090_GN_Nnmt_PE_1_SV_
MetazoaVertebrateEP00079Xenopustropicalis_6353131_NNMT_MOUSE_Nicotinamide_N_methyltransferase_OS_Mus_musculus_OX_10090_GN_Nnmt_PE_1_SV_
MetazoaVertebrateEP00079Xenopustropicalis_6353130_NNMT_MOUSE_Nicotinamide_N_methyltransferase_OS_Mus_musculus_OX_10090_GN_Nnmt_PE_1_SV_
MetazoaVertebrateEP00079Xenopustropicalis_6372446_NNMT_MOUSE_Nicotinamide_N_methyltransferase_OS_Mus_musculus_OX_10090_GN_Nnmt_PE_1_SV_
MetazoaVertebrateEP00079Xenopustropicalis_6360293_NNMT_MOUSE_Nicotinamide_N_methyltransferase_OS_Mus_musculus_OX_10090_GN_Nnmt_PE_1_SV_
MetazoaVertebrateEP00079Xenopustropicalis_6353128_NNMT_MOUSE_Nicotinamide_N_methyltransferase_OS_Mus_musculus_OX_10090_GN_Nnmt_PE_1_SV_
MetazoaVertebrateEP00079Xenopustropicalis_6343868_NNMT_MOUSE_Nicotinamide_N_methyltransferase_OS_Mus_musculus_OX_10090_GN_Nnmt_PE_1_SV_
MetazoaVertebrateEP00079Xenopustropicalis_6372452_NNMT_MOUSE_Nicotinamide_N_methyltransferase_OS_Mus_musculus_OX_10090_GN_Nnmt_PE_1_SV_
MetazoaVertebrateEP00079Xenopustropicalis_6370871_NNMT_PIG_Nicotinamide_N_methyltransferase_OS_Sus_scrofa_OX_9823_GN_NNMT_PE_2_SV_
MetazoaVertebrateEP00079Xenopustropicalis_6348976_NNMT_MOUSE_Nicotinamide_N_methyltransferase_OS_Mus_musculus_OX_10090_GN_Nnmt_PE_1_SV_
MetazoaVertebrateAnoliscarolinensis_6315245_NNMT_HUMAN_Nicotinamide_N_methyltransferase_OS_Homo_sapiens_OX_9606_GN_NNMT_PE_1_SV_
MetazoaVertebrateAnoliscarolinensis_6331402_NNMT_HUMAN_Nicotinamide_N_methyltransferase_OS_Homo_sapiens_OX_9606_GN_NNMT_PE_1_SV_
MetazoaVertebrateAnoliscarolinensis_6315246_NNMT_HUMAN_Nicotinamide_N_methyltransferase_OS_Homo_sapiens_OX_9606_GN_NNMT_PE_1_SV_
MetazoaVertebrateAnoliscarolinensis_6315247_INMT_RABIT_Indolethylamine_N_methyltransferase_OS_Oryctolagus_cuniculus_OX_9986_GN_INMT_PE_1_SV_
MetazoaVertebrateAnoliscarolinensis_6314789_NNMT_HUMAN_Nicotinamide_N_methyltransferase_OS_Homo_sapiens_OX_9606_GN_NNMT_PE_1_SV_
MetazoaVertebrateAnoliscarolinensis_6341527_NNMT_HUMAN_Nicotinamide_N_methyltransferase_OS_Homo_sapiens_OX_9606_GN_NNMT_PE_1_SV_
MetazoaVertebrateHomosapiens_6456126_NNMT_HUMAN_Nicotinamide_N_methyltransferase_OS_Homo_sapiens_OX_9606_GN_NNMT_PE_1_SV_
MetazoaVertebrateMusmusculus_6531434_NNMT_MOUSE_Nicotinamide_N_methyltransferase_OS_Mus_musculus_OX_10090_GN_Nnmt_PE_1_SV_
MetazoaVertebrateMonodelphisdomestica_6499752_NNMT_PIG_Nicotinamide_N_methyltransferase_OS_Sus_scrofa_OX_9823_GN_NNMT_PE_2_SV_
MetazoaVertebrateMonodelphisdomestica_6499783_NNMT_PIG_Nicotinamide_N_methyltransferase_OS_Sus_scrofa_OX_9823_GN_NNMT_PE_2_SV_
MetazoaVertebrateHomosapiens_6453327_INMT_HUMAN_Indolethylamine_N_methyltransferase_OS_Homo_sapiens_OX_9606_GN_INMT_PE_1_SV_
MetazoaVertebrateMusmusculus_6533314_INMT_MOUSE_Indolethylamine_N_methyltransferase_OS_Mus_musculus_OX_10090_GN_Inmt_PE_1_SV_
MetazoaVertebrateAnoliscarolinensis_6315248_NNMT_MOUSE_Nicotinamide_N_methyltransferase_OS_Mus_musculus_OX_10090_GN_Nnmt_PE_1_SV_
MetazoaVertebrateAnoliscarolinensis_6331403_NNMT_MOUSE_Nicotinamide_N_methyltransferase_OS_Mus_musculus_OX_10090_GN_Nnmt_PE_1_SV_
MetazoaVertebrateEP00079Xenopustropicalis_6359640_NNMT_PIG_Nicotinamide_N_methyltransferase_OS_Sus_scrofa_OX_9823_GN_NNMT_PE_2_SV_
MetazoaVertebrateEP00079Xenopustropicalis_6356576_INMT_PONAB_Indolethylamine_N_methyltransferase_OS_Pongo_abelii_OX_9601_GN_INMT_PE_2_SV_
MetazoaVertebrateEP00079Xenopustropicalis_6370802_INMT_PONAB_Indolethylamine_N_methyltransferase_OS_Pongo_abelii_OX_9601_GN_INMT_PE_2_SV_
MetazoaVertebrateEP00079Xenopustropicalis_6349733_INMT_RABIT_Indolethylamine_N_methyltransferase_OS_Oryctolagus_cuniculus_OX_9986_GN_INMT_PE_1_SV_
MetazoaVertebrateEP00079Xenopustropicalis_6356574_INMT_PONAB_Indolethylamine_N_methyltransferase_OS_Pongo_abelii_OX_9601_GN_INMT_PE_2_SV_
MetazoaVertebrateEP00079Xenopustropicalis_6359641_INMT_PONAB_Indolethylamine_N_methyltransferase_OS_Pongo_abelii_OX_9601_GN_INMT_PE_2_SV_
MetazoaVertebrateEP00079Xenopustropicalis_6356575_NNMT_HUMAN_Nicotinamide_N_methyltransferase_OS_Homo_sapiens_OX_9606_GN_NNMT_PE_1_SV_
MetazoaVertebrateEP00079Xenopustropicalis_6356577_NNMT_HUMAN_Nicotinamide_N_methyltransferase_OS_Homo_sapiens_OX_9606_GN_NNMT_PE_1_SV_
MetazoaVertebrateEP00079Xenopustropicalis_6344532_INMT_RABIT_Indolethylamine_N_methyltransferase_OS_Oryctolagus_cuniculus_OX_9986_GN_INMT_PE_1_SV_
MetazoaVertebrateEP00066Takifugurubripes_5684292_PNMT_PIG_Phenylethanolamine_N_methyltransferase_OS_Sus_scrofa_OX_9823_GN_PNMT_PE_2_SV_
MetazoaVertebrateEP00067Daniorerio_5764962_PNMT_BOVIN_Phenylethanolamine_N_methyltransferase_OS_Bos_taurus_OX_9913_GN_PNMT_PE_1_SV_
MetazoaVertebrateGallusgallus_6416511_PNMT_HUMAN_Phenylethanolamine_N_methyltransferase_OS_Homo_sapiens_OX_9606_GN_PNMT_PE_1_SV_
MetazoaVertebrateHomosapiens_6454715_PNMT_HUMAN_Phenylethanolamine_N_methyltransferase_OS_Homo_sapiens_OX_9606_GN_PNMT_PE_1_SV_
MetazoaVertebrateMusmusculus_6533313_PNMT_MOUSE_Phenylethanolamine_N_methyltransferase_OS_Mus_musculus_OX_10090_GN_Pnmt_PE_2_SV_
MetazoaVertebrateMonodelphisdomestica_6511009_PNMT_HUMAN_Phenylethanolamine_N_methyltransferase_OS_Homo_sapiens_OX_9606_GN_PNMT_PE_1_SV_
MetazoaVertebrateEP00079Xenopustropicalis_6355673_PNMT_HUMAN_Phenylethanolamine_N_methyltransferase_OS_Homo_sapiens_OX_9606_GN_PNMT_PE_1_SV_
MetazoaVertebrateAnoliscarolinensis_6312250_PNMT_HUMAN_Phenylethanolamine_N_methyltransferase_OS_Homo_sapiens_OX_9606_GN_PNMT_PE_1_SV_
MetazoaVertebrateLatimeriachalumnae_5773131_PNMT_HUMAN_Phenylethanolamine_N_methyltransferase_OS_Homo_sapiens_OX_9606_GN_PNMT_PE_1_SV_
MetazoaVertebrateCallorhinchusmilii_5518440_PNMT_HUMAN_Phenylethanolamine_N_methyltransferase_OS_Homo_sapiens_OX_9606_GN_PNMT_PE_1_SV_
MetazoaVertebrateEP00067Daniorerio_5721746_NNMT_MOUSE_Nicotinamide_N_methyltransferase_OS_Mus_musculus_OX_10090_GN_Nnmt_PE_1_SV_
MetazoaVertebratePetromyzonmarinus_6575228_NNMT_MOUSE_Nicotinamide_N_methyltransferase_OS_Mus_musculus_OX_10090_GN_Nnmt_PE_1_SV_
MetazoaVertebratePetromyzonmarinus_6582097_NNMT_MOUSE_Nicotinamide_N_methyltransferase_OS_Mus_musculus_OX_10090_GN_Nnmt_PE_1_SV_
MetazoaVertebratePetromyzonmarinus_6575408_NNMT_MOUSE_Nicotinamide_N_methyltransferase_OS_Mus_musculus_OX_10090_GN_Nnmt_PE_1_SV_
MetazoaCnidariaHelioporacoerulea_174843_NNMT_MOUSE_Nicotinamide_N_methyltransferase_OS_Mus_musculus_OX_10090_GN_Nnmt_PE_1_SV_
MetazoaCnidariaHelioporacoerulea_174845_NNMT_PIG_Nicotinamide_N_methyltransferase_OS_Sus_scrofa_OX_9823_GN_NNMT_PE_2_SV_
MetazoaCnidariaParamuriceabiscaya_435640_NNMT_HUMAN_Nicotinamide_N_methyltransferase_OS_Homo_sapiens_OX_9606_GN_NNMT_PE_1_SV_
MetazoaCnidariaParamuriceabiscaya_424197_NNMT_MOUSE_Nicotinamide_N_methyltransferase_OS_Mus_musculus_OX_10090_GN_Nnmt_PE_1_SV_
MetazoaCnidariaParamuriceabiscaya_424198_NNMT_MOUSE_Nicotinamide_N_methyltransferase_OS_Mus_musculus_OX_10090_GN_Nnmt_PE_1_SV_
MetazoaCnidariaParamuriceabiscaya_424199_NNMT_MOUSE_Nicotinamide_N_methyltransferase_OS_Mus_musculus_OX_10090_GN_Nnmt_PE_1_SV_
MetazoaCnidariaRenillakoellikeri_480077_INMT_MOUSE_Indolethylamine_N_methyltransferase_OS_Mus_musculus_OX_10090_GN_Inmt_PE_1_SV_
MetazoaCnidariaRenillakoellikeri_490119_INMT_MOUSE_Indolethylamine_N_methyltransferase_OS_Mus_musculus_OX_10090_GN_Inmt_PE_1_SV_
MetazoaCnidariaRenillakoellikeri_468270_INMT_RABIT_Indolethylamine_N_methyltransferase_OS_Oryctolagus_cuniculus_OX_9986_GN_INMT_PE_1_SV_
MetazoaCnidariaNematostellavectensis_011120NNMT_MOUSE_Nicotinamide_N_methyltransferase_OS_Mus_musculus_OX_10090_GN_Nnmt_PE_1_SV_
MetazoaCnidariaFungiascutaria_3194333_INMT_MOUSE_Indolethylamine_N_methyltransferase_OS_Mus_musculus_OX_10090_GN_Inmt_PE_1_SV_
MetazoaPoriferaEP00118Oscarellapearsei_4829278_PNMT_HUMAN_Phenylethanolamine_N_methyltransferase_OS_Homo_sapiens_OX_9606_GN_PNMT_PE_1_SV_
MetazoaPoriferaEP00118Oscarellapearsei_4833788_NNMT_MOUSE_Nicotinamide_N_methyltransferase_OS_Mus_musculus_OX_10090_GN_Nnmt_PE_1_SV_
MetazoaArthropodaDaphniapulex_195117_NNMT_HUMAN_Nicotinamide_N_methyltransferase_OS_Homo_sapiens_OX_9606_GN_NNMT_PE_1_SV_
MetazoaArthropodaDaphniapulex_195472_NNMT_HUMAN_Nicotinamide_N_methyltransferase_OS_Homo_sapiens_OX_9606_GN_NNMT_PE_1_SV_
MetazoaPoriferaAmphimedonqueenslandica_4844236_NNMT_MOUSE_Nicotinamide_N_methyltransferase_OS_Mus_musculus_OX_10090_GN_Nnmt_PE_1_SV_
MetazoaPoriferaAmphimedonqueenslandica_4845102_NNMT_PIG_Nicotinamide_N_methyltransferase_OS_Sus_scrofa_OX_9823_GN_NNMT_PE_2_SV_
MetazoaPoriferaAmphimedonqueenslandica_4844046_NNMT_HUMAN_Nicotinamide_N_methyltransferase_OS_Homo_sapiens_OX_9606_GN_NNMT_PE_1_SV_
MetazoaMolluscaEP00107Lottiagigantea_4180881_INMT_MOUSE_Indolethylamine_N_methyltransferase_OS_Mus_musculus_OX_10090_GN_Inmt_PE_1_SV_
MetazoaBrachiopodaLingulaunguis_1283927_INMT_RABIT_Indolethylamine_N_methyltransferase_OS_Oryctolagus_cuniculus_OX_9986_GN_INMT_PE_1_SV_
MetazoaBrachiopodaLingulaunguis_1283930_INMT_RABIT_Indolethylamine_N_methyltransferase_OS_Oryctolagus_cuniculus_OX_9986_GN_INMT_PE_1_SV_
MetazoaBrachiopodaLingulaunguis_1287218_INMT_RABIT_Indolethylamine_N_methyltransferase_OS_Oryctolagus_cuniculus_OX_9986_GN_INMT_PE_1_SV_
MetazoaBrachiopodaLingulaunguis_1284210_INMT_RABIT_Indolethylamine_N_methyltransferase_OS_Oryctolagus_cuniculus_OX_9986_GN_INMT_PE_1_SV_
MetazoaBrachiopodaLingulaunguis_1278217_NNMT_HUMAN_Nicotinamide_N_methyltransferase_OS_Homo_sapiens_OX_9606_GN_NNMT_PE_1_SV_
MetazoaBrachiopodaLingulaunguis_1289389_NNMT_HUMAN_Nicotinamide_N_methyltransferase_OS_Homo_sapiens_OX_9606_GN_NNMT_PE_1_SV_
MetazoaBrachiopodaLingulaunguis_1280242_NNMT_MOUSE_Nicotinamide_N_methyltransferase_OS_Mus_musculus_OX_10090_GN_Nnmt_PE_1_SV_
MetazoaBrachiopodaLingulaunguis_1301018_NNMT_MOUSE_Nicotinamide_N_methyltransferase_OS_Mus_musculus_OX_10090_GN_Nnmt_PE_1_SV_
MetazoaBrachiopodaLingulaunguis_1279847_INMT_RABIT_Indolethylamine_N_methyltransferase_OS_Oryctolagus_cuniculus_OX_9986_GN_INMT_PE_1_SV_
MetazoaBrachiopodaLingulaunguis_1283568_INMT_RABIT_Indolethylamine_N_methyltransferase_OS_Oryctolagus_cuniculus_OX_9986_GN_INMT_PE_1_SV_
MetazoaBrachiopodaLingulaunguis_1283833_INMT_RABIT_Indolethylamine_N_methyltransferase_OS_Oryctolagus_cuniculus_OX_9986_GN_INMT_PE_1_SV_
MetazoaBrachiopodaLingulaunguis_1283560_INMT_HUMAN_Indolethylamine_N_methyltransferase_OS_Homo_sapiens_OX_9606_GN_INMT_PE_1_SV_
MetazoaBrachiopodaLingulaunguis_1287344_NNMT_MOUSE_Nicotinamide_N_methyltransferase_OS_Mus_musculus_OX_10090_GN_Nnmt_PE_1_SV_
MetazoaBrachiopodaLingulaunguis_1302323_INMT_RABIT_Indolethylamine_N_methyltransferase_OS_Oryctolagus_cuniculus_OX_9986_GN_INMT_PE_1_SV_
MetazoaNematodaCaenorhabditiselegans_4400157_NNMT1_CAEEL_Nicotinamide_N_methyltransferase_OS_Caenorhabditis_elegans_OX_6239_GN_anmt_1_PE_3_SV_
MetazoaAnnelidaEP00103Capitellateleta_58794_INMT_RABIT_Indolethylamine_N_methyltransferase_OS_Oryctolagus_cuniculus_OX_9986_GN_INMT_PE_1_SV_
MetazoaAnnelidaEP00103Capitellateleta_63124_NNMT_HUMAN_Nicotinamide_N_methyltransferase_OS_Homo_sapiens_OX_9606_GN_NNMT_PE_1_SV_
MetazoaAnnelidaEP00103Capitellateleta_81858_NNMT_HUMAN_Nicotinamide_N_methyltransferase_OS_Homo_sapiens_OX_9606_GN_NNMT_PE_1_SV_
MetazoaAnnelidaEP00103Capitellateleta_68020_INMT_MOUSE_Indolethylamine_N_methyltransferase_OS_Mus_musculus_OX_10090_GN_Inmt_PE_1_SV_
MetazoaAnnelidaEP00103Capitellateleta_66091_NNMT_MOUSE_Nicotinamide_N_methyltransferase_OS_Mus_musculus_OX_10090_GN_Nnmt_PE_1_SV_
MetazoaArthropodaStegodyphusmimosarum_1147183_INMT_PONAB_Indolethylamine_N_methyltransferase_OS_Pongo_abelii_OX_9601_GN_INMT_PE_2_SV_
MetazoaArthropodaStegodyphusmimosarum_1170723_INMT_PONAB_Indolethylamine_N_methyltransferase_OS_Pongo_abelii_OX_9601_GN_INMT_PE_2_SV_
MetazoaArthropodaStegodyphusmimosarum_1160607_NNMT_HUMAN_Nicotinamide_N_methyltransferase_OS_Homo_sapiens_OX_9606_GN_NNMT_PE_1_SV_
MetazoaArthropodaStegodyphusmimosarum_1161276_NNMT_MOUSE_Nicotinamide_N_methyltransferase_OS_Mus_musculus_OX_10090_GN_Nnmt_PE_1_SV_
MetazoaArthropodaStegodyphusmimosarum_1158319_INMT_PONAB_Indolethylamine_N_methyltransferase_OS_Pongo_abelii_OX_9601_GN_INMT_PE_2_SV_
MetazoaCephaBranchiostomabelcheri_2811444_INMT_RABIT_Indolethylamine_N_methyltransferase_OS_Oryctolagus_cuniculus_OX_9986_GN_INMT_PE_1_SV_
MetazoaCephaBranchiostomabelcheri_2815267_INMT_PONAB_Indolethylamine_N_methyltransferase_OS_Pongo_abelii_OX_9601_GN_INMT_PE_2_SV_
MetazoaCephaBranchiostomabelcheri_2823966_NNMT_HUMAN_Nicotinamide_N_methyltransferase_OS_Homo_sapiens_OX_9606_GN_NNMT_PE_1_SV_
MetazoaCephaBranchiostomabelcheri_2823967_NNMT_HUMAN_Nicotinamide_N_methyltransferase_OS_Homo_sapiens_OX_9606_GN_NNMT_PE_1_SV_
MetazoaCephaBranchiostomabelcheri_2797353_NNMT_HUMAN_Nicotinamide_N_methyltransferase_OS_Homo_sapiens_OX_9606_GN_NNMT_PE_1_SV_
MetazoaCephaBranchiostomabelcheri_2800873_NNMT_MOUSE_Nicotinamide_N_methyltransferase_OS_Mus_musculus_OX_10090_GN_Nnmt_PE_1_SV_
MetazoaCephaBranchiostomabelcheri_2796295_NNMT_HUMAN_Nicotinamide_N_methyltransferase_OS_Homo_sapiens_OX_9606_GN_NNMT_PE_1_SV_
MetazoaCephaBranchiostomabelcheri_2801509_NNMT_MOUSE_Nicotinamide_N_methyltransferase_OS_Mus_musculus_OX_10090_GN_Nnmt_PE_1_SV_
MetazoaCephaBranchiostomabelcheri_2832379_PNMT_HUMAN_Phenylethanolamine_N_methyltransferase_OS_Homo_sapiens_OX_9606_GN_PNMT_PE_1_SV_
MetazoaCephaBranchiostomabelcheri_2832451_NNMT_HUMAN_Nicotinamide_N_methyltransferase_OS_Homo_sapiens_OX_9606_GN_NNMT_PE_1_SV_
MetazoaCephaBranchiostomabelcheri_2831031_NNMT_MOUSE_Nicotinamide_N_methyltransferase_OS_Mus_musculus_OX_10090_GN_Nnmt_PE_1_SV_
MetazoaCephaBranchiostomabelcheri_2831032_NNMT_MOUSE_Nicotinamide_N_methyltransferase_OS_Mus_musculus_OX_10090_GN_Nnmt_PE_1_SV_
MetazoaCephaBranchiostomabelcheri_2813847_NNMT_HUMAN_Nicotinamide_N_methyltransferase_OS_Homo_sapiens_OX_9606_GN_NNMT_PE_1_SV_
MetazoaCephaBranchiostomabelcheri_2825060_NNMT_MOUSE_Nicotinamide_N_methyltransferase_OS_Mus_musculus_OX_10090_GN_Nnmt_PE_1_SV_
0.22

6B.

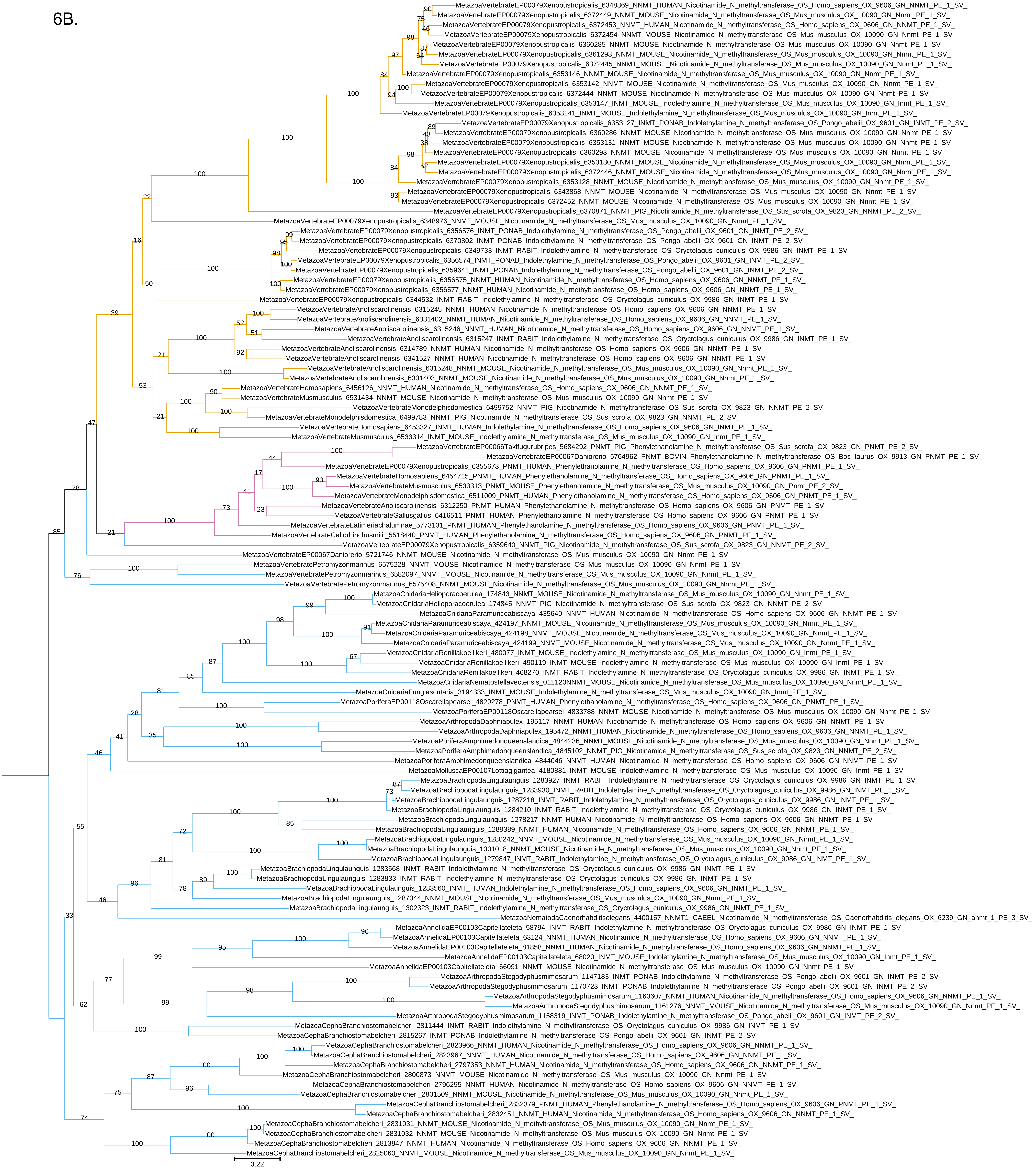

MetazoaVertebrateEP00079Xenopustropicalis_6348369_NNMT_HUMAN_Nicotinamide_N_methyltransferase_OS_Homo_sapiens_OX_9606_GN_NNMT_PE_1_SV_
MetazoaVertebrateEP00079Xenopustropicalis_6372449_NNMT_MOUSE_Nicotinamide_N_methyltransferase_OS_Mus_musculus_OX_10090_GN_Nnmt_PE_1_SV_
MetazoaVertebrateEP00079Xenopustropicalis_6372453_NNMT_HUMAN_Nicotinamide_N_methyltransferase_OS_Homo_sapiens_OX_9606_GN_NNMT_PE_1_SV_
MetazoaVertebrateEP00079Xenopustropicalis_6372454_NNMT_MOUSE_Nicotinamide_N_methyltransferase_OS_Mus_musculus_OX_10090_GN_Nnmt_PE_1_SV_
MetazoaVertebrateEP00079Xenopustropicalis_6360285_NNMT_MOUSE_Nicotinamide_N_methyltransferase_OS_Mus_musculus_OX_10090_GN_Nnmt_PE_1_SV_
MetazoaVertebrateEP00079Xenopustropicalis_6361293_NNMT_MOUSE_Nicotinamide_N_methyltransferase_OS_Mus_musculus_OX_10090_GN_Nnmt_PE_1_SV_
MetazoaVertebrateEP00079Xenopustropicalis_6372445_NNMT_MOUSE_Nicotinamide_N_methyltransferase_OS_Mus_musculus_OX_10090_GN_Nnmt_PE_1_SV_
MetazoaVertebrateEP00079Xenopustropicalis_6353146_NNMT_MOUSE_Nicotinamide_N_methyltransferase_OS_Mus_musculus_OX_10090_GN_Nnmt_PE_1_SV_
MetazoaVertebrateEP00079Xenopustropicalis_6353142_NNMT_MOUSE_Nicotinamide_N_methyltransferase_OS_Mus_musculus_OX_10090_GN_Nnmt_PE_1_SV_
MetazoaVertebrateEP00079Xenopustropicalis_6372444_NNMT_MOUSE_Nicotinamide_N_methyltransferase_OS_Mus_musculus_OX_10090_GN_Nnmt_PE_1_SV_
MetazoaVertebrateEP00079Xenopustropicalis_6353147_INMT_MOUSE_Indolethylamine_N_methyltransferase_OS_Mus_musculus_OX_10090_GN_Inmt_PE_1_SV_
MetazoaVertebrateEP00079Xenopustropicalis_6353141_INMT_MOUSE_Indolethylamine_N_methyltransferase_OS_Mus_musculus_OX_10090_GN_Inmt_PE_1_SV_
MetazoaVertebrateEP00079Xenopustropicalis_6353127_INMT_PONAB_Indolethylamine_N_methyltransferase_OS_Pongo_abelii_OX_9601_GN_INMT_PE_2_SV_
MetazoaVertebrateEP00079Xenopustropicalis_6360286_NNMT_MOUSE_Nicotinamide_N_methyltransferase_OS_Mus_musculus_OX_10090_GN_Nnmt_PE_1_SV_
MetazoaVertebrateEP00079Xenopustropicalis_6353131_NNMT_MOUSE_Nicotinamide_N_methyltransferase_OS_Mus_musculus_OX_10090_GN_Nnmt_PE_1_SV_
MetazoaVertebrateEP00079Xenopustropicalis_6360293_NNMT_MOUSE_Nicotinamide_N_methyltransferase_OS_Mus_musculus_OX_10090_GN_Nnmt_PE_1_SV_
MetazoaVertebrateEP00079Xenopustropicalis_6353130_NNMT_MOUSE_Nicotinamide_N_methyltransferase_OS_Mus_musculus_OX_10090_GN_Nnmt_PE_1_SV_
MetazoaVertebrateEP00079Xenopustropicalis_6372446_NNMT_MOUSE_Nicotinamide_N_methyltransferase_OS_Mus_musculus_OX_10090_GN_Nnmt_PE_1_SV_
MetazoaVertebrateEP00079Xenopustropicalis_6353128_NNMT_MOUSE_Nicotinamide_N_methyltransferase_OS_Mus_musculus_OX_10090_GN_Nnmt_PE_1_SV_
MetazoaVertebrateEP00079Xenopustropicalis_6343868_NNMT_MOUSE_Nicotinamide_N_methyltransferase_OS_Mus_musculus_OX_10090_GN_Nnmt_PE_1_SV_
MetazoaVertebrateEP00079Xenopustropicalis_6372452_NNMT_MOUSE_Nicotinamide_N_methyltransferase_OS_Mus_musculus_OX_10090_GN_Nnmt_PE_1_SV_
MetazoaVertebrateEP00079Xenopustropicalis_6370871_NNMT_PIG_Nicotinamide_N_methyltransferase_OS_Sus_scrofa_OX_9823_GN_NNMT_PE_2_SV_
MetazoaVertebrateEP00079Xenopustropicalis_6348976_NNMT_MOUSE_Nicotinamide_N_methyltransferase_OS_Mus_musculus_OX_10090_GN_Nnmt_PE_1_SV_
MetazoaVertebrateEP00079Xenopustropicalis_6356576_INMT_PONAB_Indolethylamine_N_methyltransferase_OS_Pongo_abelii_OX_9601_GN_INMT_PE_2_SV_
MetazoaVertebrateEP00079Xenopustropicalis_6370802_INMT_PONAB_Indolethylamine_N_methyltransferase_OS_Pongo_abelii_OX_9601_GN_INMT_PE_2_SV_
MetazoaVertebrateEP00079Xenopustropicalis_6349733_INMT_RABIT_Indolethylamine_N_methyltransferase_OS_Oryctolagus_cuniculus_OX_9986_GN_INMT_PE_1_SV_
MetazoaVertebrateEP00079Xenopustropicalis_6356574_INMT_PONAB_Indolethylamine_N_methyltransferase_OS_Pongo_abelii_OX_9601_GN_INMT_PE_2_SV_
MetazoaVertebrateEP00079Xenopustropicalis_6359641_INMT_PONAB_Indolethylamine_N_methyltransferase_OS_Pongo_abelii_OX_9601_GN_INMT_PE_2_SV_
MetazoaVertebrateEP00079Xenopustropicalis_6356575_NNMT_HUMAN_Nicotinamide_N_methyltransferase_OS_Homo_sapiens_OX_9606_GN_NNMT_PE_1_SV_
MetazoaVertebrateEP00079Xenopustropicalis_6356577_NNMT_HUMAN_Nicotinamide_N_methyltransferase_OS_Homo_sapiens_OX_9606_GN_NNMT_PE_1_SV_
MetazoaVertebrateEP00079Xenopustropicalis_6344532_INMT_RABIT_Indolethylamine_N_methyltransferase_OS_Oryctolagus_cuniculus_OX_9986_GN_INMT_PE_1_SV_
MetazoaVertebrateAnoliscarolinensis_6315245_NNMT_HUMAN_Nicotinamide_N_methyltransferase_OS_Homo_sapiens_OX_9606_GN_NNMT_PE_1_SV_
MetazoaVertebrateAnoliscarolinensis_6331402_NNMT_HUMAN_Nicotinamide_N_methyltransferase_OS_Homo_sapiens_OX_9606_GN_NNMT_PE_1_SV_
MetazoaVertebrateAnoliscarolinensis_6315246_NNMT_HUMAN_Nicotinamide_N_methyltransferase_OS_Homo_sapiens_OX_9606_GN_NNMT_PE_1_SV_
MetazoaVertebrateAnoliscarolinensis_6315247_INMT_RABIT_Indolethylamine_N_methyltransferase_OS_Oryctolagus_cuniculus_OX_9986_GN_INMT_PE_1_SV_
MetazoaVertebrateAnoliscarolinensis_6314789_NNMT_HUMAN_Nicotinamide_N_methyltransferase_OS_Homo_sapiens_OX_9606_GN_NNMT_PE_1_SV_
MetazoaVertebrateAnoliscarolinensis_6341527_NNMT_HUMAN_Nicotinamide_N_methyltransferase_OS_Homo_sapiens_OX_9606_GN_NNMT_PE_1_SV_
MetazoaVertebrateAnoliscarolinensis_6315248_NNMT_MOUSE_Nicotinamide_N_methyltransferase_OS_Mus_musculus_OX_10090_GN_Nnmt_PE_1_SV_
MetazoaVertebrateAnoliscarolinensis_6331403_NNMT_MOUSE_Nicotinamide_N_methyltransferase_OS_Mus_musculus_OX_10090_GN_Nnmt_PE_1_SV_
MetazoaVertebrateHomosapiens_6456126_NNMT_HUMAN_Nicotinamide_N_methyltransferase_OS_Homo_sapiens_OX_9606_GN_NNMT_PE_1_SV_
MetazoaVertebrateMusmusculus_6531434_NNMT_MOUSE_Nicotinamide_N_methyltransferase_OS_Mus_musculus_OX_10090_GN_Nnmt_PE_1_SV_
MetazoaVertebrateMonodelphisdomestica_6499752_NNMT_PIG_Nicotinamide_N_methyltransferase_OS_Sus_scrofa_OX_9823_GN_NNMT_PE_2_SV_
MetazoaVertebrateMonodelphisdomestica_6499783_NNMT_PIG_Nicotinamide_N_methyltransferase_OS_Sus_scrofa_OX_9823_GN_NNMT_PE_2_SV_
MetazoaVertebrateHomosapiens_6453327_INMT_HUMAN_Indolethylamine_N_methyltransferase_OS_Homo_sapiens_OX_9606_GN_INMT_PE_1_SV_
MetazoaVertebrateMusmusculus_6533314_INMT_MOUSE_Indolethylamine_N_methyltransferase_OS_Mus_musculus_OX_10090_GN_Inmt_PE_1_SV_
MetazoaVertebrateEP00066Takifugurubripes_5684292_PNMT_PIG_Phenylethanolamine_N_methyltransferase_OS_Sus_scrofa_OX_9823_GN_PNMT_PE_2_SV_
MetazoaVertebrateEP00067Daniorerio_5764962_PNMT_BOVIN_Phenylethanolamine_N_methyltransferase_OS_Bos_taurus_OX_9913_GN_PNMT_PE_1_SV_
MetazoaVertebrateEP00079Xenopustropicalis_6355673_PNMT_HUMAN_Phenylethanolamine_N_methyltransferase_OS_Homo_sapiens_OX_9606_GN_PNMT_PE_1_SV_
MetazoaVertebrateHomosapiens_6454715_PNMT_HUMAN_Phenylethanolamine_N_methyltransferase_OS_Homo_sapiens_OX_9606_GN_PNMT_PE_1_SV_
MetazoaVertebrateMusmusculus_6533313_PNMT_MOUSE_Phenylethanolamine_N_methyltransferase_OS_Mus_musculus_OX_10090_GN_Pnmt_PE_2_SV_
MetazoaVertebrateMonodelphisdomestica_6511009_PNMT_HUMAN_Phenylethanolamine_N_methyltransferase_OS_Homo_sapiens_OX_9606_GN_PNMT_PE_1_SV_
MetazoaVertebrateAnoliscarolinensis_6312250_PNMT_HUMAN_Phenylethanolamine_N_methyltransferase_OS_Homo_sapiens_OX_9606_GN_PNMT_PE_1_SV_
MetazoaVertebrateGallusgallus_6416511_PNMT_HUMAN_Phenylethanolamine_N_methyltransferase_OS_Homo_sapiens_OX_9606_GN_PNMT_PE_1_SV_
MetazoaVertebrateLatimeriachalumnae_5773131_PNMT_HUMAN_Phenylethanolamine_N_methyltransferase_OS_Homo_sapiens_OX_9606_GN_PNMT_PE_1_SV_
MetazoaVertebrateCallorhinchusmilii_5518440_PNMT_HUMAN_Phenylethanolamine_N_methyltransferase_OS_Homo_sapiens_OX_9606_GN_PNMT_PE_1_SV_
MetazoaVertebrateEP00079Xenopustropicalis_6359640_NNMT_PIG_Nicotinamide_N_methyltransferase_OS_Sus_scrofa_OX_9823_GN_NNMT_PE_2_SV_
MetazoaVertebrateEP00067Daniorerio_5721746_NNMT_MOUSE_Nicotinamide_N_methyltransferase_OS_Mus_musculus_OX_10090_GN_Nnmt_PE_1_SV_
MetazoaVertebratePetromyzonmarinus_6575228_NNMT_MOUSE_Nicotinamide_N_methyltransferase_OS_Mus_musculus_OX_10090_GN_Nnmt_PE_1_SV_
MetazoaVertebratePetromyzonmarinus_6582097_NNMT_MOUSE_Nicotinamide_N_methyltransferase_OS_Mus_musculus_OX_10090_GN_Nnmt_PE_1_SV_
MetazoaVertebratePetromyzonmarinus_6575408_NNMT_MOUSE_Nicotinamide_N_methyltransferase_OS_Mus_musculus_OX_10090_GN_Nnmt_PE_1_SV_
MetazoaCnidariaHelioporacoerulea_174843_NNMT_MOUSE_Nicotinamide_N_methyltransferase_OS_Mus_musculus_OX_10090_GN_Nnmt_PE_1_SV_
MetazoaCnidariaHelioporacoerulea_174845_NNMT_PIG_Nicotinamide_N_methyltransferase_OS_Sus_scrofa_OX_9823_GN_NNMT_PE_2_SV_
MetazoaCnidariaParamuriceabiscaya_435640_NNMT_HUMAN_Nicotinamide_N_methyltransferase_OS_Homo_sapiens_OX_9606_GN_NNMT_PE_1_SV_
MetazoaCnidariaParamuriceabiscaya_424197_NNMT_MOUSE_Nicotinamide_N_methyltransferase_OS_Mus_musculus_OX_10090_GN_Nnmt_PE_1_SV_
MetazoaCnidariaParamuriceabiscaya_424198_NNMT_MOUSE_Nicotinamide_N_methyltransferase_OS_Mus_musculus_OX_10090_GN_Nnmt_PE_1_SV_
MetazoaCnidariaParamuriceabiscaya_424199_NNMT_MOUSE_Nicotinamide_N_methyltransferase_OS_Mus_musculus_OX_10090_GN_Nnmt_PE_1_SV_
MetazoaCnidariaRenillakoellikeri_480077_INMT_MOUSE_Indolethylamine_N_methyltransferase_OS_Mus_musculus_OX_10090_GN_Inmt_PE_1_SV_
MetazoaCnidariaRenillakoellikeri_490119_INMT_MOUSE_Indolethylamine_N_methyltransferase_OS_Mus_musculus_OX_10090_GN_Inmt_PE_1_SV_
MetazoaCnidariaRenillakoellikeri_468270_INMT_RABIT_Indolethylamine_N_methyltransferase_OS_Oryctolagus_cuniculus_OX_9986_GN_INMT_PE_1_SV_
MetazoaCnidariaNematostellavectensis_011120NNMT_MOUSE_Nicotinamide_N_methyltransferase_OS_Mus_musculus_OX_10090_GN_Nnmt_PE_1_SV_
MetazoaCnidariaFungiascutaria_3194333_INMT_MOUSE_Indolethylamine_N_methyltransferase_OS_Mus_musculus_OX_10090_GN_Inmt_PE_1_SV_
MetazoaPoriferaEP00118Oscarellapearsei_4829278_PNMT_HUMAN_Phenylethanolamine_N_methyltransferase_OS_Homo_sapiens_OX_9606_GN_PNMT_PE_1_SV_
MetazoaPoriferaEP00118Oscarellapearsei_4833788_NNMT_MOUSE_Nicotinamide_N_methyltransferase_OS_Mus_musculus_OX_10090_GN_Nnmt_PE_1_SV_
MetazoaArthropodaDaphniapulex_195117_NNMT_HUMAN_Nicotinamide_N_methyltransferase_OS_Homo_sapiens_OX_9606_GN_NNMT_PE_1_SV_
MetazoaArthropodaDaphniapulex_195472_NNMT_HUMAN_Nicotinamide_N_methyltransferase_OS_Homo_sapiens_OX_9606_GN_NNMT_PE_1_SV_
MetazoaPoriferaAmphimedonqueenslandica_4844236_NNMT_MOUSE_Nicotinamide_N_methyltransferase_OS_Mus_musculus_OX_10090_GN_Nnmt_PE_1_SV_
MetazoaPoriferaAmphimedonqueenslandica_4845102_NNMT_PIG_Nicotinamide_N_methyltransferase_OS_Sus_scrofa_OX_9823_GN_NNMT_PE_2_SV_
MetazoaPoriferaAmphimedonqueenslandica_4844046_NNMT_HUMAN_Nicotinamide_N_methyltransferase_OS_Homo_sapiens_OX_9606_GN_NNMT_PE_1_SV_
MetazoaMolluscaEP00107Lottiagigantea_4180881_INMT_MOUSE_Indolethylamine_N_methyltransferase_OS_Mus_musculus_OX_10090_GN_Inmt_PE_1_SV_
MetazoaBrachiopodaLingulaunguis_1283927_INMT_RABIT_Indolethylamine_N_methyltransferase_OS_Oryctolagus_cuniculus_OX_9986_GN_INMT_PE_1_SV_
MetazoaBrachiopodaLingulaunguis_1283930_INMT_RABIT_Indolethylamine_N_methyltransferase_OS_Oryctolagus_cuniculus_OX_9986_GN_INMT_PE_1_SV_
MetazoaBrachiopodaLingulaunguis_1287218_INMT_RABIT_Indolethylamine_N_methyltransferase_OS_Oryctolagus_cuniculus_OX_9986_GN_INMT_PE_1_SV_
MetazoaBrachiopodaLingulaunguis_1284210_INMT_RABIT_Indolethylamine_N_methyltransferase_OS_Oryctolagus_cuniculus_OX_9986_GN_INMT_PE_1_SV_
MetazoaBrachiopodaLingulaunguis_1278217_NNMT_HUMAN_Nicotinamide_N_methyltransferase_OS_Homo_sapiens_OX_9606_GN_NNMT_PE_1_SV_
MetazoaBrachiopodaLingulaunguis_1289389_NNMT_HUMAN_Nicotinamide_N_methyltransferase_OS_Homo_sapiens_OX_9606_GN_NNMT_PE_1_SV_
MetazoaBrachiopodaLingulaunguis_1280242_NNMT_MOUSE_Nicotinamide_N_methyltransferase_OS_Mus_musculus_OX_10090_GN_Nnmt_PE_1_SV_
MetazoaBrachiopodaLingulaunguis_1301018_NNMT_MOUSE_Nicotinamide_N_methyltransferase_OS_Mus_musculus_OX_10090_GN_Nnmt_PE_1_SV_
MetazoaBrachiopodaLingulaunguis_1279847_INMT_RABIT_Indolethylamine_N_methyltransferase_OS_Oryctolagus_cuniculus_OX_9986_GN_INMT_PE_1_SV_
MetazoaBrachiopodaLingulaunguis_1283568_INMT_RABIT_Indolethylamine_N_methyltransferase_OS_Oryctolagus_cuniculus_OX_9986_GN_INMT_PE_1_SV_
MetazoaBrachiopodaLingulaunguis_1283833_INMT_RABIT_Indolethylamine_N_methyltransferase_OS_Oryctolagus_cuniculus_OX_9986_GN_INMT_PE_1_SV_
MetazoaBrachiopodaLingulaunguis_1283560_INMT_HUMAN_Indolethylamine_N_methyltransferase_OS_Homo_sapiens_OX_9606_GN_INMT_PE_1_SV_
MetazoaBrachiopodaLingulaunguis_1287344_NNMT_MOUSE_Nicotinamide_N_methyltransferase_OS_Mus_musculus_OX_10090_GN_Nnmt_PE_1_SV_
MetazoaBrachiopodaLingulaunguis_1302323_INMT_RABIT_Indolethylamine_N_methyltransferase_OS_Oryctolagus_cuniculus_OX_9986_GN_INMT_PE_1_SV_
MetazoaNematodaCaenorhabditiselegans_4400157_NNMT1_CAEEL_Nicotinamide_N_methyltransferase_OS_Caenorhabditis_elegans_OX_6239_GN_anmt_1_PE_3_SV_
MetazoaAnnelidaEP00103Capitellateleta_58794_INMT_RABIT_Indolethylamine_N_methyltransferase_OS_Oryctolagus_cuniculus_OX_9986_GN_INMT_PE_1_SV_
MetazoaAnnelidaEP00103Capitellateleta_63124_NNMT_HUMAN_Nicotinamide_N_methyltransferase_OS_Homo_sapiens_OX_9606_GN_NNMT_PE_1_SV_
MetazoaAnnelidaEP00103Capitellateleta_81858_NNMT_HUMAN_Nicotinamide_N_methyltransferase_OS_Homo_sapiens_OX_9606_GN_NNMT_PE_1_SV_
MetazoaAnnelidaEP00103Capitellateleta_68020_INMT_MOUSE_Indolethylamine_N_methyltransferase_OS_Mus_musculus_OX_10090_GN_Inmt_PE_1_SV_
MetazoaAnnelidaEP00103Capitellateleta_66091_NNMT_MOUSE_Nicotinamide_N_methyltransferase_OS_Mus_musculus_OX_10090_GN_Nnmt_PE_1_SV_
MetazoaArthropodaStegodyphusmimosarum_1147183_INMT_PONAB_Indolethylamine_N_methyltransferase_OS_Pongo_abelii_OX_9601_GN_INMT_PE_2_SV_
MetazoaArthropodaStegodyphusmimosarum_1170723_INMT_PONAB_Indolethylamine_N_methyltransferase_OS_Pongo_abelii_OX_9601_GN_INMT_PE_2_SV_
MetazoaArthropodaStegodyphusmimosarum_1160607_NNMT_HUMAN_Nicotinamide_N_methyltransferase_OS_Homo_sapiens_OX_9606_GN_NNMT_PE_1_SV_
MetazoaArthropodaStegodyphusmimosarum_1161276_NNMT_MOUSE_Nicotinamide_N_methyltransferase_OS_Mus_musculus_OX_10090_GN_Nnmt_PE_1_SV_
MetazoaArthropodaStegodyphusmimosarum_1158319_INMT_PONAB_Indolethylamine_N_methyltransferase_OS_Pongo_abelii_OX_9601_GN_INMT_PE_2_SV_
MetazoaCephaBranchiostomabelcheri_2811444_INMT_RABIT_Indolethylamine_N_methyltransferase_OS_Oryctolagus_cuniculus_OX_9986_GN_INMT_PE_1_SV_
MetazoaCephaBranchiostomabelcheri_2815267_INMT_PONAB_Indolethylamine_N_methyltransferase_OS_Pongo_abelii_OX_9601_GN_INMT_PE_2_SV_
MetazoaCephaBranchiostomabelcheri_2823966_NNMT_HUMAN_Nicotinamide_N_methyltransferase_OS_Homo_sapiens_OX_9606_GN_NNMT_PE_1_SV_
MetazoaCephaBranchiostomabelcheri_2823967_NNMT_HUMAN_Nicotinamide_N_methyltransferase_OS_Homo_sapiens_OX_9606_GN_NNMT_PE_1_SV_
MetazoaCephaBranchiostomabelcheri_2797353_NNMT_HUMAN_Nicotinamide_N_methyltransferase_OS_Homo_sapiens_OX_9606_GN_NNMT_PE_1_SV_
MetazoaCephaBranchiostomabelcheri_2800873_NNMT_MOUSE_Nicotinamide_N_methyltransferase_OS_Mus_musculus_OX_10090_GN_Nnmt_PE_1_SV_
MetazoaCephaBranchiostomabelcheri_2796295_NNMT_HUMAN_Nicotinamide_N_methyltransferase_OS_Homo_sapiens_OX_9606_GN_NNMT_PE_1_SV_
MetazoaCephaBranchiostomabelcheri_2801509_NNMT_MOUSE_Nicotinamide_N_methyltransferase_OS_Mus_musculus_OX_10090_GN_Nnmt_PE_1_SV_
MetazoaCephaBranchiostomabelcheri_2832379_PNMT_HUMAN_Phenylethanolamine_N_methyltransferase_OS_Homo_sapiens_OX_9606_GN_PNMT_PE_1_SV_
MetazoaCephaBranchiostomabelcheri_2832451_NNMT_HUMAN_Nicotinamide_N_methyltransferase_OS_Homo_sapiens_OX_9606_GN_NNMT_PE_1_SV_
MetazoaCephaBranchiostomabelcheri_2831031_NNMT_MOUSE_Nicotinamide_N_methyltransferase_OS_Mus_musculus_OX_10090_GN_Nnmt_PE_1_SV_
MetazoaCephaBranchiostomabelcheri_2831032_NNMT_MOUSE_Nicotinamide_N_methyltransferase_OS_Mus_musculus_OX_10090_GN_Nnmt_PE_1_SV_
MetazoaCephaBranchiostomabelcheri_2813847_NNMT_HUMAN_Nicotinamide_N_methyltransferase_OS_Homo_sapiens_OX_9606_GN_NNMT_PE_1_SV_
MetazoaCephaBranchiostomabelcheri_2825060_NNMT_MOUSE_Nicotinamide_N_methyltransferase_OS_Mus_musculus_OX_10090_GN_Nnmt_PE_1_SV_
0.22

6C.

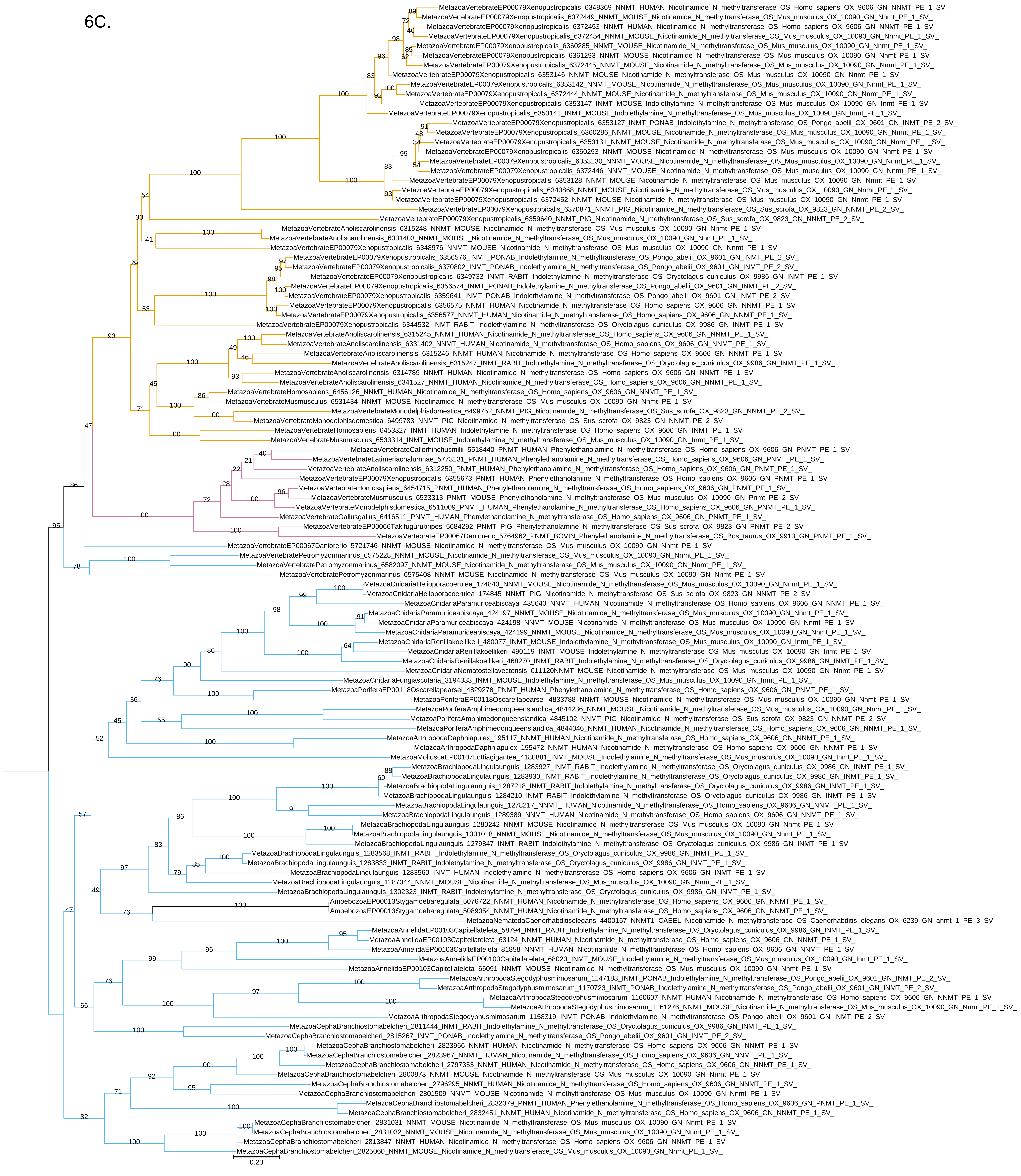

MetazoaVertebrateEP00079Xenopustropicalis_6348369_NNMT_HUMAN_Nicotinamide_N_methyltransferase_OS_Homo_sapiens_OX_9606_GN_NNMT_PE_1_SV_
MetazoaVertebrateEP00079Xenopustropicalis_6372449_NNMT_MOUSE_Nicotinamide_N_methyltransferase_OS_Mus_musculus_OX_10090_GN_Nnmt_PE_1_SV_
MetazoaVertebrateEP00079Xenopustropicalis_6372453_NNMT_HUMAN_Nicotinamide_N_methyltransferase_OS_Homo_sapiens_OX_9606_GN_NNMT_PE_1_SV_
MetazoaVertebrateEP00079Xenopustropicalis_6372454_NNMT_MOUSE_Nicotinamide_N_methyltransferase_OS_Mus_musculus_OX_10090_GN_Nnmt_PE_1_SV_
MetazoaVertebrateEP00079Xenopustropicalis_6360285_NNMT_MOUSE_Nicotinamide_N_methyltransferase_OS_Mus_musculus_OX_10090_GN_Nnmt_PE_1_SV_
MetazoaVertebrateEP00079Xenopustropicalis_6361293_NNMT_MOUSE_Nicotinamide_N_methyltransferase_OS_Mus_musculus_OX_10090_GN_Nnmt_PE_1_SV_
MetazoaVertebrateEP00079Xenopustropicalis_6372445_NNMT_MOUSE_Nicotinamide_N_methyltransferase_OS_Mus_musculus_OX_10090_GN_Nnmt_PE_1_SV_
MetazoaVertebrateEP00079Xenopustropicalis_6353146_NNMT_MOUSE_Nicotinamide_N_methyltransferase_OS_Mus_musculus_OX_10090_GN_Nnmt_PE_1_SV_
MetazoaVertebrateEP00079Xenopustropicalis_6353142_NNMT_MOUSE_Nicotinamide_N_methyltransferase_OS_Mus_musculus_OX_10090_GN_Nnmt_PE_1_SV_
MetazoaVertebrateEP00079Xenopustropicalis_6372444_NNMT_MOUSE_Nicotinamide_N_methyltransferase_OS_Mus_musculus_OX_10090_GN_Nnmt_PE_1_SV_
MetazoaVertebrateEP00079Xenopustropicalis_6353147_INMT_MOUSE_Indolethylamine_N_methyltransferase_OS_Mus_musculus_OX_10090_GN_Inmt_PE_1_SV_
MetazoaVertebrateEP00079Xenopustropicalis_6353141_INMT_MOUSE_Indolethylamine_N_methyltransferase_OS_Mus_musculus_OX_10090_GN_Inmt_PE_1_SV_
MetazoaVertebrateEP00079Xenopustropicalis_6353127_INMT_PONAB_Indolethylamine_N_methyltransferase_OS_Pongo_abelii_OX_9601_GN_INMT_PE_2_SV_
MetazoaVertebrateEP00079Xenopustropicalis_6360286_NNMT_MOUSE_Nicotinamide_N_methyltransferase_OS_Mus_musculus_OX_10090_GN_Nnmt_PE_1_SV_
MetazoaVertebrateEP00079Xenopustropicalis_6353131_NNMT_MOUSE_Nicotinamide_N_methyltransferase_OS_Mus_musculus_OX_10090_GN_Nnmt_PE_1_SV_
MetazoaVertebrateEP00079Xenopustropicalis_6360293_NNMT_MOUSE_Nicotinamide_N_methyltransferase_OS_Mus_musculus_OX_10090_GN_Nnmt_PE_1_SV_
MetazoaVertebrateEP00079Xenopustropicalis_6353130_NNMT_MOUSE_Nicotinamide_N_methyltransferase_OS_Mus_musculus_OX_10090_GN_Nnmt_PE_1_SV_
MetazoaVertebrateEP00079Xenopustropicalis_6372446_NNMT_MOUSE_Nicotinamide_N_methyltransferase_OS_Mus_musculus_OX_10090_GN_Nnmt_PE_1_SV_
MetazoaVertebrateEP00079Xenopustropicalis_6353128_NNMT_MOUSE_Nicotinamide_N_methyltransferase_OS_Mus_musculus_OX_10090_GN_Nnmt_PE_1_SV_
MetazoaVertebrateEP00079Xenopustropicalis_6343868_NNMT_MOUSE_Nicotinamide_N_methyltransferase_OS_Mus_musculus_OX_10090_GN_Nnmt_PE_1_SV_
MetazoaVertebrateEP00079Xenopustropicalis_6372452_NNMT_MOUSE_Nicotinamide_N_methyltransferase_OS_Mus_musculus_OX_10090_GN_Nnmt_PE_1_SV_
MetazoaVertebrateEP00079Xenopustropicalis_6370871_NNMT_PIG_Nicotinamide_N_methyltransferase_OS_Sus_scrofa_OX_9823_GN_NNMT_PE_2_SV_
MetazoaVertebrateEP00079Xenopustropicalis_6359640_NNMT_PIG_Nicotinamide_N_methyltransferase_OS_Sus_scrofa_OX_9823_GN_NNMT_PE_2_SV_
MetazoaVertebrateAnoliscarolinensis_6315248_NNMT_MOUSE_Nicotinamide_N_methyltransferase_OS_Mus_musculus_OX_10090_GN_Nnmt_PE_1_SV_
MetazoaVertebrateAnoliscarolinensis_6331403_NNMT_MOUSE_Nicotinamide_N_methyltransferase_OS_Mus_musculus_OX_10090_GN_Nnmt_PE_1_SV_
MetazoaVertebrateEP00079Xenopustropicalis_6348976_NNMT_MOUSE_Nicotinamide_N_methyltransferase_OS_Mus_musculus_OX_10090_GN_Nnmt_PE_1_SV_
MetazoaVertebrateEP00079Xenopustropicalis_6356576_INMT_PONAB_Indolethylamine_N_methyltransferase_OS_Pongo_abelii_OX_9601_GN_INMT_PE_2_SV_
MetazoaVertebrateEP00079Xenopustropicalis_6370802_INMT_PONAB_Indolethylamine_N_methyltransferase_OS_Pongo_abelii_OX_9601_GN_INMT_PE_2_SV_
MetazoaVertebrateEP00079Xenopustropicalis_6349733_INMT_RABIT_Indolethylamine_N_methyltransferase_OS_Oryctolagus_cuniculus_OX_9986_GN_INMT_PE_1_SV_
MetazoaVertebrateEP00079Xenopustropicalis_6356574_INMT_PONAB_Indolethylamine_N_methyltransferase_OS_Pongo_abelii_OX_9601_GN_INMT_PE_2_SV_
MetazoaVertebrateEP00079Xenopustropicalis_6359641_INMT_PONAB_Indolethylamine_N_methyltransferase_OS_Pongo_abelii_OX_9601_GN_INMT_PE_2_SV_
MetazoaVertebrateEP00079Xenopustropicalis_6356575_NNMT_HUMAN_Nicotinamide_N_methyltransferase_OS_Homo_sapiens_OX_9606_GN_NNMT_PE_1_SV_
MetazoaVertebrateEP00079Xenopustropicalis_6356577_NNMT_HUMAN_Nicotinamide_N_methyltransferase_OS_Homo_sapiens_OX_9606_GN_NNMT_PE_1_SV_
MetazoaVertebrateEP00079Xenopustropicalis_6344532_INMT_RABIT_Indolethylamine_N_methyltransferase_OS_Oryctolagus_cuniculus_OX_9986_GN_INMT_PE_1_SV_
MetazoaVertebrateAnoliscarolinensis_6315245_NNMT_HUMAN_Nicotinamide_N_methyltransferase_OS_Homo_sapiens_OX_9606_GN_NNMT_PE_1_SV_
MetazoaVertebrateAnoliscarolinensis_6331402_NNMT_HUMAN_Nicotinamide_N_methyltransferase_OS_Homo_sapiens_OX_9606_GN_NNMT_PE_1_SV_
MetazoaVertebrateAnoliscarolinensis_6315246_NNMT_HUMAN_Nicotinamide_N_methyltransferase_OS_Homo_sapiens_OX_9606_GN_NNMT_PE_1_SV_
MetazoaVertebrateAnoliscarolinensis_6315247_INMT_RABIT_Indolethylamine_N_methyltransferase_OS_Oryctolagus_cuniculus_OX_9986_GN_INMT_PE_1_SV_
MetazoaVertebrateAnoliscarolinensis_6314789_NNMT_HUMAN_Nicotinamide_N_methyltransferase_OS_Homo_sapiens_OX_9606_GN_NNMT_PE_1_SV_
MetazoaVertebrateAnoliscarolinensis_6341527_NNMT_HUMAN_Nicotinamide_N_methyltransferase_OS_Homo_sapiens_OX_9606_GN_NNMT_PE_1_SV_
MetazoaVertebrateHomosapiens_6456126_NNMT_HUMAN_Nicotinamide_N_methyltransferase_OS_Homo_sapiens_OX_9606_GN_NNMT_PE_1_SV_
MetazoaVertebrateMusmusculus_6531434_NNMT_MOUSE_Nicotinamide_N_methyltransferase_OS_Mus_musculus_OX_10090_GN_Nnmt_PE_1_SV_
MetazoaVertebrateMonodelphisdomestica_6499752_NNMT_PIG_Nicotinamide_N_methyltransferase_OS_Sus_scrofa_OX_9823_GN_NNMT_PE_2_SV_
MetazoaVertebrateMonodelphisdomestica_6499783_NNMT_PIG_Nicotinamide_N_methyltransferase_OS_Sus_scrofa_OX_9823_GN_NNMT_PE_2_SV_
MetazoaVertebrateHomosapiens_6453327_INMT_HUMAN_Indolethylamine_N_methyltransferase_OS_Homo_sapiens_OX_9606_GN_INMT_PE_1_SV_
MetazoaVertebrateMusmusculus_6533314_INMT_MOUSE_Indolethylamine_N_methyltransferase_OS_Mus_musculus_OX_10090_GN_Inmt_PE_1_SV_
MetazoaVertebrateCallorhinchusmilii_5518440_PNMT_HUMAN_Phenylethanolamine_N_methyltransferase_OS_Homo_sapiens_OX_9606_GN_PNMT_PE_1_SV_
MetazoaVertebrateLatimeriachalumnae_5773131_PNMT_HUMAN_Phenylethanolamine_N_methyltransferase_OS_Homo_sapiens_OX_9606_GN_PNMT_PE_1_SV_
MetazoaVertebrateAnoliscarolinensis_6312250_PNMT_HUMAN_Phenylethanolamine_N_methyltransferase_OS_Homo_sapiens_OX_9606_GN_PNMT_PE_1_SV_
MetazoaVertebrateEP00079Xenopustropicalis_6355673_PNMT_HUMAN_Phenylethanolamine_N_methyltransferase_OS_Homo_sapiens_OX_9606_GN_PNMT_PE_1_SV_
MetazoaVertebrateHomosapiens_6454715_PNMT_HUMAN_Phenylethanolamine_N_methyltransferase_OS_Homo_sapiens_OX_9606_GN_PNMT_PE_1_SV_
MetazoaVertebrateMusmusculus_6533313_PNMT_MOUSE_Phenylethanolamine_N_methyltransferase_OS_Mus_musculus_OX_10090_GN_Pnmt_PE_2_SV_
MetazoaVertebrateMonodelphisdomestica_6511009_PNMT_HUMAN_Phenylethanolamine_N_methyltransferase_OS_Homo_sapiens_OX_9606_GN_PNMT_PE_1_SV_
MetazoaVertebrateGallusgallus_6416511_PNMT_HUMAN_Phenylethanolamine_N_methyltransferase_OS_Homo_sapiens_OX_9606_GN_PNMT_PE_1_SV_
MetazoaVertebrateEP00066Takifugurubripes_5684292_PNMT_PIG_Phenylethanolamine_N_methyltransferase_OS_Sus_scrofa_OX_9823_GN_PNMT_PE_2_SV_
MetazoaVertebrateEP00067Daniorerio_5764962_PNMT_BOVIN_Phenylethanolamine_N_methyltransferase_OS_Bos_taurus_OX_9913_GN_PNMT_PE_1_SV_
MetazoaVertebrateEP00067Daniorerio_5721746_NNMT_MOUSE_Nicotinamide_N_methyltransferase_OS_Mus_musculus_OX_10090_GN_Nnmt_PE_1_SV_
MetazoaVertebratePetromyzonmarinus_6575228_NNMT_MOUSE_Nicotinamide_N_methyltransferase_OS_Mus_musculus_OX_10090_GN_Nnmt_PE_1_SV_
MetazoaVertebratePetromyzonmarinus_6582097_NNMT_MOUSE_Nicotinamide_N_methyltransferase_OS_Mus_musculus_OX_10090_GN_Nnmt_PE_1_SV_
MetazoaVertebratePetromyzonmarinus_6575408_NNMT_MOUSE_Nicotinamide_N_methyltransferase_OS_Mus_musculus_OX_10090_GN_Nnmt_PE_1_SV_
MetazoaCnidariaHelioporacoerulea_174843_NNMT_MOUSE_Nicotinamide_N_methyltransferase_OS_Mus_musculus_OX_10090_GN_Nnmt_PE_1_SV_
MetazoaCnidariaHelioporacoerulea_174845_NNMT_PIG_Nicotinamide_N_methyltransferase_OS_Sus_scrofa_OX_9823_GN_NNMT_PE_2_SV_
MetazoaCnidariaParamuriceabiscaya_435640_NNMT_HUMAN_Nicotinamide_N_methyltransferase_OS_Homo_sapiens_OX_9606_GN_NNMT_PE_1_SV_
MetazoaCnidariaParamuriceabiscaya_424197_NNMT_MOUSE_Nicotinamide_N_methyltransferase_OS_Mus_musculus_OX_10090_GN_Nnmt_PE_1_SV_
MetazoaCnidariaParamuriceabiscaya_424198_NNMT_MOUSE_Nicotinamide_N_methyltransferase_OS_Mus_musculus_OX_10090_GN_Nnmt_PE_1_SV_
MetazoaCnidariaParamuriceabiscaya_424199_NNMT_MOUSE_Nicotinamide_N_methyltransferase_OS_Mus_musculus_OX_10090_GN_Nnmt_PE_1_SV_
MetazoaCnidariaRenillakoellikeri_480077_INMT_MOUSE_Indolethylamine_N_methyltransferase_OS_Mus_musculus_OX_10090_GN_Inmt_PE_1_SV_
MetazoaCnidariaRenillakoellikeri_490119_INMT_MOUSE_Indolethylamine_N_methyltransferase_OS_Mus_musculus_OX_10090_GN_Inmt_PE_1_SV_
MetazoaCnidariaRenillakoellikeri_468270_INMT_RABIT_Indolethylamine_N_methyltransferase_OS_Oryctolagus_cuniculus_OX_9986_GN_INMT_PE_1_SV_
MetazoaCnidariaNematostellavectensis_011120NNMT_MOUSE_Nicotinamide_N_methyltransferase_OS_Mus_musculus_OX_10090_GN_Nnmt_PE_1_SV_
MetazoaCnidariaFungiascutaria_3194333_INMT_MOUSE_Indolethylamine_N_methyltransferase_OS_Mus_musculus_OX_10090_GN_Inmt_PE_1_SV_
MetazoaPoriferaEP00118Oscarellapearsei_4829278_PNMT_HUMAN_Phenylethanolamine_N_methyltransferase_OS_Homo_sapiens_OX_9606_GN_PNMT_PE_1_SV_
MetazoaPoriferaEP00118Oscarellapearsei_4833788_NNMT_MOUSE_Nicotinamide_N_methyltransferase_OS_Mus_musculus_OX_10090_GN_Nnmt_PE_1_SV_
MetazoaPoriferaAmphimedonqueenslandica_4844236_NNMT_MOUSE_Nicotinamide_N_methyltransferase_OS_Mus_musculus_OX_10090_GN_Nnmt_PE_1_SV_
MetazoaPoriferaAmphimedonqueenslandica_4845102_NNMT_PIG_Nicotinamide_N_methyltransferase_OS_Sus_scrofa_OX_9823_GN_NNMT_PE_2_SV_
MetazoaPoriferaAmphimedonqueenslandica_4844046_NNMT_HUMAN_Nicotinamide_N_methyltransferase_OS_Homo_sapiens_OX_9606_GN_NNMT_PE_1_SV_
MetazoaArthropodaDaphniapulex_195117_NNMT_HUMAN_Nicotinamide_N_methyltransferase_OS_Homo_sapiens_OX_9606_GN_NNMT_PE_1_SV_
MetazoaArthropodaDaphniapulex_195472_NNMT_HUMAN_Nicotinamide_N_methyltransferase_OS_Homo_sapiens_OX_9606_GN_NNMT_PE_1_SV_
MetazoaMolluscaEP00107Lottiagigantea_4180881_INMT_MOUSE_Indolethylamine_N_methyltransferase_OS_Mus_musculus_OX_10090_GN_Inmt_PE_1_SV_
MetazoaBrachiopodaLingulaunguis_1283927_INMT_RABIT_Indolethylamine_N_methyltransferase_OS_Oryctolagus_cuniculus_OX_9986_GN_INMT_PE_1_SV_
MetazoaBrachiopodaLingulaunguis_1283930_INMT_RABIT_Indolethylamine_N_methyltransferase_OS_Oryctolagus_cuniculus_OX_9986_GN_INMT_PE_1_SV_
MetazoaBrachiopodaLingulaunguis_1287218_INMT_RABIT_Indolethylamine_N_methyltransferase_OS_Oryctolagus_cuniculus_OX_9986_GN_INMT_PE_1_SV_
MetazoaBrachiopodaLingulaunguis_1284210_INMT_RABIT_Indolethylamine_N_methyltransferase_OS_Oryctolagus_cuniculus_OX_9986_GN_INMT_PE_1_SV_
MetazoaBrachiopodaLingulaunguis_1278217_NNMT_HUMAN_Nicotinamide_N_methyltransferase_OS_Homo_sapiens_OX_9606_GN_NNMT_PE_1_SV_
MetazoaBrachiopodaLingulaunguis_1289389_NNMT_HUMAN_Nicotinamide_N_methyltransferase_OS_Homo_sapiens_OX_9606_GN_NNMT_PE_1_SV_
MetazoaBrachiopodaLingulaunguis_1280242_NNMT_MOUSE_Nicotinamide_N_methyltransferase_OS_Mus_musculus_OX_10090_GN_Nnmt_PE_1_SV_
MetazoaBrachiopodaLingulaunguis_1301018_NNMT_MOUSE_Nicotinamide_N_methyltransferase_OS_Mus_musculus_OX_10090_GN_Nnmt_PE_1_SV_
MetazoaBrachiopodaLingulaunguis_1279847_INMT_RABIT_Indolethylamine_N_methyltransferase_OS_Oryctolagus_cuniculus_OX_9986_GN_INMT_PE_1_SV_
MetazoaBrachiopodaLingulaunguis_1283568_INMT_RABIT_Indolethylamine_N_methyltransferase_OS_Oryctolagus_cuniculus_OX_9986_GN_INMT_PE_1_SV_
MetazoaBrachiopodaLingulaunguis_1283833_INMT_RABIT_Indolethylamine_N_methyltransferase_OS_Oryctolagus_cuniculus_OX_9986_GN_INMT_PE_1_SV_
MetazoaBrachiopodaLingulaunguis_1283560_INMT_HUMAN_Indolethylamine_N_methyltransferase_OS_Homo_sapiens_OX_9606_GN_INMT_PE_1_SV_
MetazoaBrachiopodaLingulaunguis_1287344_NNMT_MOUSE_Nicotinamide_N_methyltransferase_OS_Mus_musculus_OX_10090_GN_Nnmt_PE_1_SV_
MetazoaBrachiopodaLingulaunguis_1302323_INMT_RABIT_Indolethylamine_N_methyltransferase_OS_Oryctolagus_cuniculus_OX_9986_GN_INMT_PE_1_SV_
AmoebozoaEP00013Stygamoebaregulata_5076722_NNMT_HUMAN_Nicotinamide_N_methyltransferase_OS_Homo_sapiens_OX_9606_GN_NNMT_PE_1_SV_
AmoebozoaEP00013Stygamoebaregulata_5089054_NNMT_HUMAN_Nicotinamide_N_methyltransferase_OS_Homo_sapiens_OX_9606_GN_NNMT_PE_1_SV_
MetazoaNematodaCaenorhabditiselegans_4400157_NNMT1_CAEEL_Nicotinamide_N_methyltransferase_OS_Caenorhabditis_elegans_OX_6239_GN_anmt_1_PE_3_SV_
MetazoaAnnelidaEP00103Capitellateleta_58794_INMT_RABIT_Indolethylamine_N_methyltransferase_OS_Oryctolagus_cuniculus_OX_9986_GN_INMT_PE_1_SV_
MetazoaAnnelidaEP00103Capitellateleta_63124_NNMT_HUMAN_Nicotinamide_N_methyltransferase_OS_Homo_sapiens_OX_9606_GN_NNMT_PE_1_SV_
MetazoaAnnelidaEP00103Capitellateleta_81858_NNMT_HUMAN_Nicotinamide_N_methyltransferase_OS_Homo_sapiens_OX_9606_GN_NNMT_PE_1_SV_
MetazoaAnnelidaEP00103Capitellateleta_68020_INMT_MOUSE_Indolethylamine_N_methyltransferase_OS_Mus_musculus_OX_10090_GN_Inmt_PE_1_SV_
MetazoaAnnelidaEP00103Capitellateleta_66091_NNMT_MOUSE_Nicotinamide_N_methyltransferase_OS_Mus_musculus_OX_10090_GN_Nnmt_PE_1_SV_
MetazoaArthropodaStegodyphusmimosarum_1147183_INMT_PONAB_Indolethylamine_N_methyltransferase_OS_Pongo_abelii_OX_9601_GN_INMT_PE_2_SV_
MetazoaArthropodaStegodyphusmimosarum_1170723_INMT_PONAB_Indolethylamine_N_methyltransferase_OS_Pongo_abelii_OX_9601_GN_INMT_PE_2_SV_
MetazoaArthropodaStegodyphusmimosarum_1160607_NNMT_HUMAN_Nicotinamide_N_methyltransferase_OS_Homo_sapiens_OX_9606_GN_NNMT_PE_1_SV_
MetazoaArthropodaStegodyphusmimosarum_1161276_NNMT_MOUSE_Nicotinamide_N_methyltransferase_OS_Mus_musculus_OX_10090_GN_Nnmt_PE_1_SV_
MetazoaArthropodaStegodyphusmimosarum_1158319_INMT_PONAB_Indolethylamine_N_methyltransferase_OS_Pongo_abelii_OX_9601_GN_INMT_PE_2_SV_
MetazoaCephaBranchiostomabelcheri_2811444_INMT_RABIT_Indolethylamine_N_methyltransferase_OS_Oryctolagus_cuniculus_OX_9986_GN_INMT_PE_1_SV_
MetazoaCephaBranchiostomabelcheri_2815267_INMT_PONAB_Indolethylamine_N_methyltransferase_OS_Pongo_abelii_OX_9601_GN_INMT_PE_2_SV_
MetazoaCephaBranchiostomabelcheri_2823966_NNMT_HUMAN_Nicotinamide_N_methyltransferase_OS_Homo_sapiens_OX_9606_GN_NNMT_PE_1_SV_
MetazoaCephaBranchiostomabelcheri_2823967_NNMT_HUMAN_Nicotinamide_N_methyltransferase_OS_Homo_sapiens_OX_9606_GN_NNMT_PE_1_SV_
MetazoaCephaBranchiostomabelcheri_2797353_NNMT_HUMAN_Nicotinamide_N_methyltransferase_OS_Homo_sapiens_OX_9606_GN_NNMT_PE_1_SV_
MetazoaCephaBranchiostomabelcheri_2800873_NNMT_MOUSE_Nicotinamide_N_methyltransferase_OS_Mus_musculus_OX_10090_GN_Nnmt_PE_1_SV_
MetazoaCephaBranchiostomabelcheri_2796295_NNMT_HUMAN_Nicotinamide_N_methyltransferase_OS_Homo_sapiens_OX_9606_GN_NNMT_PE_1_SV_
MetazoaCephaBranchiostomabelcheri_2801509_NNMT_MOUSE_Nicotinamide_N_methyltransferase_OS_Mus_musculus_OX_10090_GN_Nnmt_PE_1_SV_
MetazoaCephaBranchiostomabelcheri_2832379_PNMT_HUMAN_Phenylethanolamine_N_methyltransferase_OS_Homo_sapiens_OX_9606_GN_PNMT_PE_1_SV_
MetazoaCephaBranchiostomabelcheri_2832451_NNMT_HUMAN_Nicotinamide_N_methyltransferase_OS_Homo_sapiens_OX_9606_GN_NNMT_PE_1_SV_
MetazoaCephaBranchiostomabelcheri_2831031_NNMT_MOUSE_Nicotinamide_N_methyltransferase_OS_Mus_musculus_OX_10090_GN_Nnmt_PE_1_SV_
MetazoaCephaBranchiostomabelcheri_2831032_NNMT_MOUSE_Nicotinamide_N_methyltransferase_OS_Mus_musculus_OX_10090_GN_Nnmt_PE_1_SV_
MetazoaCephaBranchiostomabelcheri_2813847_NNMT_HUMAN_Nicotinamide_N_methyltransferase_OS_Homo_sapiens_OX_9606_GN_NNMT_PE_1_SV_
MetazoaCephaBranchiostomabelcheri_2825060_NNMT_MOUSE_Nicotinamide_N_methyltransferase_OS_Mus_musculus_OX_10090_GN_Nnmt_PE_1_SV_
0.23

6D.

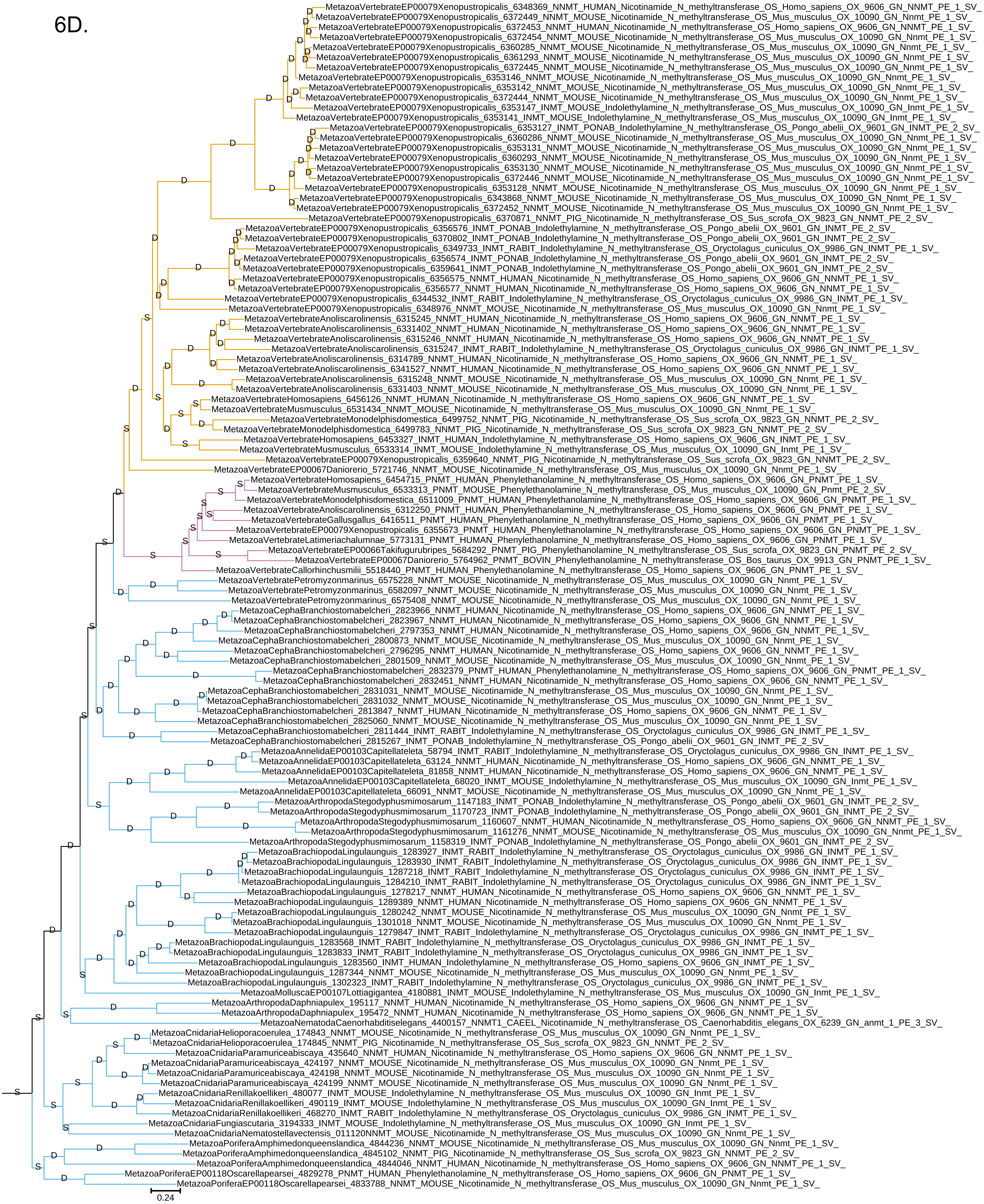

MetazoaVertebrateEP00079Xenopustropicalis_6348369_NNMT_HUMAN_Nicotinamide_N_methyltransferase_OS_Homo_sapiens_OX_9606_GN_NNMT_PE_1_SV_
MetazoaVertebrateEP00079Xenopustropicalis_6372449_NNMT_MOUSE_Nicotinamide_N_methyltransferase_OS_Mus_musculus_OX_10090_GN_Nnmt_PE_1_SV_
MetazoaVertebrateEP00079Xenopustropicalis_6372453_NNMT_HUMAN_Nicotinamide_N_methyltransferase_OS_Homo_sapiens_OX_9606_GN_NNMT_PE_1_SV_
MetazoaVertebrateEP00079Xenopustropicalis_6372454_NNMT_MOUSE_Nicotinamide_N_methyltransferase_OS_Mus_musculus_OX_10090_GN_Nnmt_PE_1_SV_
MetazoaVertebrateEP00079Xenopustropicalis_6360285_NNMT_MOUSE_Nicotinamide_N_methyltransferase_OS_Mus_musculus_OX_10090_GN_Nnmt_PE_1_SV_
MetazoaVertebrateEP00079Xenopustropicalis_6361293_NNMT_MOUSE_Nicotinamide_N_methyltransferase_OS_Mus_musculus_OX_10090_GN_Nnmt_PE_1_SV_
MetazoaVertebrateEP00079Xenopustropicalis_6372445_NNMT_MOUSE_Nicotinamide_N_methyltransferase_OS_Mus_musculus_OX_10090_GN_Nnmt_PE_1_SV_
MetazoaVertebrateEP00079Xenopustropicalis_6353146_NNMT_MOUSE_Nicotinamide_N_methyltransferase_OS_Mus_musculus_OX_10090_GN_Nnmt_PE_1_SV_
MetazoaVertebrateEP00079Xenopustropicalis_6353142_NNMT_MOUSE_Nicotinamide_N_methyltransferase_OS_Mus_musculus_OX_10090_GN_Nnmt_PE_1_SV_
MetazoaVertebrateEP00079Xenopustropicalis_6372444_NNMT_MOUSE_Nicotinamide_N_methyltransferase_OS_Mus_musculus_OX_10090_GN_Nnmt_PE_1_SV_
MetazoaVertebrateEP00079Xenopustropicalis_6353147_INMT_MOUSE_Indolethylamine_N_methyltransferase_OS_Mus_musculus_OX_10090_GN_Inmt_PE_1_SV_
MetazoaVertebrateEP00079Xenopustropicalis_6353141_INMT_MOUSE_Indolethylamine_N_methyltransferase_OS_Mus_musculus_OX_10090_GN_Inmt_PE_1_SV_
MetazoaVertebrateEP00079Xenopustropicalis_6353127_INMT_PONAB_Indolethylamine_N_methyltransferase_OS_Pongo_abelii_OX_9601_GN_INMT_PE_2_SV_
MetazoaVertebrateEP00079Xenopustropicalis_6360286_NNMT_MOUSE_Nicotinamide_N_methyltransferase_OS_Mus_musculus_OX_10090_GN_Nnmt_PE_1_SV_
MetazoaVertebrateEP00079Xenopustropicalis_6353131_NNMT_MOUSE_Nicotinamide_N_methyltransferase_OS_Mus_musculus_OX_10090_GN_Nnmt_PE_1_SV_
MetazoaVertebrateEP00079Xenopustropicalis_6360293_NNMT_MOUSE_Nicotinamide_N_methyltransferase_OS_Mus_musculus_OX_10090_GN_Nnmt_PE_1_SV_
MetazoaVertebrateEP00079Xenopustropicalis_6353130_NNMT_MOUSE_Nicotinamide_N_methyltransferase_OS_Mus_musculus_OX_10090_GN_Nnmt_PE_1_SV_
MetazoaVertebrateEP00079Xenopustropicalis_6372446_NNMT_MOUSE_Nicotinamide_N_methyltransferase_OS_Mus_musculus_OX_10090_GN_Nnmt_PE_1_SV_
MetazoaVertebrateEP00079Xenopustropicalis_6353128_NNMT_MOUSE_Nicotinamide_N_methyltransferase_OS_Mus_musculus_OX_10090_GN_Nnmt_PE_1_SV_
MetazoaVertebrateEP00079Xenopustropicalis_6343868_NNMT_MOUSE_Nicotinamide_N_methyltransferase_OS_Mus_musculus_OX_10090_GN_Nnmt_PE_1_SV_
MetazoaVertebrateEP00079Xenopustropicalis_6372452_NNMT_MOUSE_Nicotinamide_N_methyltransferase_OS_Mus_musculus_OX_10090_GN_Nnmt_PE_1_SV_
MetazoaVertebrateEP00079Xenopustropicalis_6370871_NNMT_PIG_Nicotinamide_N_methyltransferase_OS_Sus_scrofa_OX_9823_GN_NNMT_PE_2_SV_
MetazoaVertebrateEP00079Xenopustropicalis_6356576_INMT_PONAB_Indolethylamine_N_methyltransferase_OS_Pongo_abelii_OX_9601_GN_INMT_PE_2_SV_
MetazoaVertebrateEP00079Xenopustropicalis_6370802_INMT_PONAB_Indolethylamine_N_methyltransferase_OS_Pongo_abelii_OX_9601_GN_INMT_PE_2_SV_
MetazoaVertebrateEP00079Xenopustropicalis_6349733_INMT_RABIT_Indolethylamine_N_methyltransferase_OS_Oryctolagus_cuniculus_OX_9986_GN_INMT_PE_1_SV_
MetazoaVertebrateEP00079Xenopustropicalis_6356574_INMT_PONAB_Indolethylamine_N_methyltransferase_OS_Pongo_abelii_OX_9601_GN_INMT_PE_2_SV_
MetazoaVertebrateEP00079Xenopustropicalis_6359641_INMT_PONAB_Indolethylamine_N_methyltransferase_OS_Pongo_abelii_OX_9601_GN_INMT_PE_2_SV_
MetazoaVertebrateEP00079Xenopustropicalis_6356575_NNMT_HUMAN_Nicotinamide_N_methyltransferase_OS_Homo_sapiens_OX_9606_GN_NNMT_PE_1_SV_
MetazoaVertebrateEP00079Xenopustropicalis_6356577_NNMT_HUMAN_Nicotinamide_N_methyltransferase_OS_Homo_sapiens_OX_9606_GN_NNMT_PE_1_SV_
MetazoaVertebrateEP00079Xenopustropicalis_6344532_INMT_RABIT_Indolethylamine_N_methyltransferase_OS_Oryctolagus_cuniculus_OX_9986_GN_INMT_PE_1_SV_
MetazoaVertebrateEP00079Xenopustropicalis_6348976_NNMT_MOUSE_Nicotinamide_N_methyltransferase_OS_Mus_musculus_OX_10090_GN_Nnmt_PE_1_SV_
MetazoaVertebrateAnoliscarolinensis_6315245_NNMT_HUMAN_Nicotinamide_N_methyltransferase_OS_Homo_sapiens_OX_9606_GN_NNMT_PE_1_SV_
MetazoaVertebrateAnoliscarolinensis_6331402_NNMT_HUMAN_Nicotinamide_N_methyltransferase_OS_Homo_sapiens_OX_9606_GN_NNMT_PE_1_SV_
MetazoaVertebrateAnoliscarolinensis_6315246_NNMT_HUMAN_Nicotinamide_N_methyltransferase_OS_Homo_sapiens_OX_9606_GN_NNMT_PE_1_SV_
MetazoaVertebrateAnoliscarolinensis_6315247_INMT_RABIT_Indolethylamine_N_methyltransferase_OS_Oryctolagus_cuniculus_OX_9986_GN_INMT_PE_1_SV_
MetazoaVertebrateAnoliscarolinensis_6314789_NNMT_HUMAN_Nicotinamide_N_methyltransferase_OS_Homo_sapiens_OX_9606_GN_NNMT_PE_1_SV_
MetazoaVertebrateAnoliscarolinensis_6341527_NNMT_HUMAN_Nicotinamide_N_methyltransferase_OS_Homo_sapiens_OX_9606_GN_NNMT_PE_1_SV_
MetazoaVertebrateAnoliscarolinensis_6315248_NNMT_MOUSE_Nicotinamide_N_methyltransferase_OS_Mus_musculus_OX_10090_GN_Nnmt_PE_1_SV_
MetazoaVertebrateAnoliscarolinensis_6331403_NNMT_MOUSE_Nicotinamide_N_methyltransferase_OS_Mus_musculus_OX_10090_GN_Nnmt_PE_1_SV_
MetazoaVertebrateHomosapiens_6456126_NNMT_HUMAN_Nicotinamide_N_methyltransferase_OS_Homo_sapiens_OX_9606_GN_NNMT_PE_1_SV_
MetazoaVertebrateMusmusculus_6531434_NNMT_MOUSE_Nicotinamide_N_methyltransferase_OS_Mus_musculus_OX_10090_GN_Nnmt_PE_1_SV_
MetazoaVertebrateMonodelphisdomestica_6499752_NNMT_PIG_Nicotinamide_N_methyltransferase_OS_Sus_scrofa_OX_9823_GN_NNMT_PE_2_SV_
MetazoaVertebrateMonodelphisdomestica_6499783_NNMT_PIG_Nicotinamide_N_methyltransferase_OS_Sus_scrofa_OX_9823_GN_NNMT_PE_2_SV_
MetazoaVertebrateHomosapiens_6453327_INMT_HUMAN_Indolethylamine_N_methyltransferase_OS_Homo_sapiens_OX_9606_GN_INMT_PE_1_SV_
MetazoaVertebrateMusmusculus_6533314_INMT_MOUSE_Indolethylamine_N_methyltransferase_OS_Mus_musculus_OX_10090_GN_Inmt_PE_1_SV_
MetazoaVertebrateEP00079Xenopustropicalis_6359640_NNMT_PIG_Nicotinamide_N_methyltransferase_OS_Sus_scrofa_OX_9823_GN_NNMT_PE_2_SV_
MetazoaVertebrateEP00067Daniorerio_5721746_NNMT_MOUSE_Nicotinamide_N_methyltransferase_OS_Mus_musculus_OX_10090_GN_Nnmt_PE_1_SV_
MetazoaVertebrateHomosapiens_6454715_PNMT_HUMAN_Phenylethanolamine_N_methyltransferase_OS_Homo_sapiens_OX_9606_GN_PNMT_PE_1_SV_
MetazoaVertebrateMusmusculus_6533313_PNMT_MOUSE_Phenylethanolamine_N_methyltransferase_OS_Mus_musculus_OX_10090_GN_Pnmt_PE_2_SV_
MetazoaVertebrateMonodelphisdomestica_6511009_PNMT_HUMAN_Phenylethanolamine_N_methyltransferase_OS_Homo_sapiens_OX_9606_GN_PNMT_PE_1_SV_
MetazoaVertebrateAnoliscarolinensis_6312250_PNMT_HUMAN_Phenylethanolamine_N_methyltransferase_OS_Homo_sapiens_OX_9606_GN_PNMT_PE_1_SV_
MetazoaVertebrateGallusgallus_6416511_PNMT_HUMAN_Phenylethanolamine_N_methyltransferase_OS_Homo_sapiens_OX_9606_GN_PNMT_PE_1_SV_
MetazoaVertebrateEP00079Xenopustropicalis_6355673_PNMT_HUMAN_Phenylethanolamine_N_methyltransferase_OS_Homo_sapiens_OX_9606_GN_PNMT_PE_1_SV_
MetazoaVertebrateLatimeriachalumnae_5773131_PNMT_HUMAN_Phenylethanolamine_N_methyltransferase_OS_Homo_sapiens_OX_9606_GN_PNMT_PE_1_SV_
MetazoaVertebrateEP00066Takifugurubripes_5684292_PNMT_PIG_Phenylethanolamine_N_methyltransferase_OS_Sus_scrofa_OX_9823_GN_PNMT_PE_2_SV_
MetazoaVertebrateEP00067Daniorerio_5764962_PNMT_BOVIN_Phenylethanolamine_N_methyltransferase_OS_Bos_taurus_OX_9913_GN_PNMT_PE_1_SV_
MetazoaVertebrateCallorhinchusmilii_5518440_PNMT_HUMAN_Phenylethanolamine_N_methyltransferase_OS_Homo_sapiens_OX_9606_GN_PNMT_PE_1_SV_
MetazoaVertebratePetromyzonmarinus_6575228_NNMT_MOUSE_Nicotinamide_N_methyltransferase_OS_Mus_musculus_OX_10090_GN_Nnmt_PE_1_SV_
MetazoaVertebratePetromyzonmarinus_6582097_NNMT_MOUSE_Nicotinamide_N_methyltransferase_OS_Mus_musculus_OX_10090_GN_Nnmt_PE_1_SV_
MetazoaVertebratePetromyzonmarinus_6575408_NNMT_MOUSE_Nicotinamide_N_methyltransferase_OS_Mus_musculus_OX_10090_GN_Nnmt_PE_1_SV_
MetazoaCephaBranchiostomabelcheri_2823966_NNMT_HUMAN_Nicotinamide_N_methyltransferase_OS_Homo_sapiens_OX_9606_GN_NNMT_PE_1_SV_
MetazoaCephaBranchiostomabelcheri_2823967_NNMT_HUMAN_Nicotinamide_N_methyltransferase_OS_Homo_sapiens_OX_9606_GN_NNMT_PE_1_SV_
MetazoaCephaBranchiostomabelcheri_2797353_NNMT_HUMAN_Nicotinamide_N_methyltransferase_OS_Homo_sapiens_OX_9606_GN_NNMT_PE_1_SV_
MetazoaCephaBranchiostomabelcheri_2800873_NNMT_MOUSE_Nicotinamide_N_methyltransferase_OS_Mus_musculus_OX_10090_GN_Nnmt_PE_1_SV_
MetazoaCephaBranchiostomabelcheri_2796295_NNMT_HUMAN_Nicotinamide_N_methyltransferase_OS_Homo_sapiens_OX_9606_GN_NNMT_PE_1_SV_
MetazoaCephaBranchiostomabelcheri_2801509_NNMT_MOUSE_Nicotinamide_N_methyltransferase_OS_Mus_musculus_OX_10090_GN_Nnmt_PE_1_SV_
MetazoaCephaBranchiostomabelcheri_2832379_PNMT_HUMAN_Phenylethanolamine_N_methyltransferase_OS_Homo_sapiens_OX_9606_GN_PNMT_PE_1_SV_
MetazoaCephaBranchiostomabelcheri_2832451_NNMT_HUMAN_Nicotinamide_N_methyltransferase_OS_Homo_sapiens_OX_9606_GN_NNMT_PE_1_SV_
MetazoaCephaBranchiostomabelcheri_2831031_NNMT_MOUSE_Nicotinamide_N_methyltransferase_OS_Mus_musculus_OX_10090_GN_Nnmt_PE_1_SV_
MetazoaCephaBranchiostomabelcheri_2831032_NNMT_MOUSE_Nicotinamide_N_methyltransferase_OS_Mus_musculus_OX_10090_GN_Nnmt_PE_1_SV_
MetazoaCephaBranchiostomabelcheri_2813847_NNMT_HUMAN_Nicotinamide_N_methyltransferase_OS_Homo_sapiens_OX_9606_GN_NNMT_PE_1_SV_
MetazoaCephaBranchiostomabelcheri_2825060_NNMT_MOUSE_Nicotinamide_N_methyltransferase_OS_Mus_musculus_OX_10090_GN_Nnmt_PE_1_SV_
MetazoaCephaBranchiostomabelcheri_2811444_INMT_RABIT_Indolethylamine_N_methyltransferase_OS_Oryctolagus_cuniculus_OX_9986_GN_INMT_PE_1_SV_
MetazoaCephaBranchiostomabelcheri_2815267_INMT_PONAB_Indolethylamine_N_methyltransferase_OS_Pongo_abelii_OX_9601_GN_INMT_PE_2_SV_
MetazoaAnnelidaEP00103Capitellateleta_58794_INMT_RABIT_Indolethylamine_N_methyltransferase_OS_Oryctolagus_cuniculus_OX_9986_GN_INMT_PE_1_SV_
MetazoaAnnelidaEP00103Capitellateleta_63124_NNMT_HUMAN_Nicotinamide_N_methyltransferase_OS_Homo_sapiens_OX_9606_GN_NNMT_PE_1_SV_
MetazoaAnnelidaEP00103Capitellateleta_81858_NNMT_HUMAN_Nicotinamide_N_methyltransferase_OS_Homo_sapiens_OX_9606_GN_NNMT_PE_1_SV_
MetazoaAnnelidaEP00103Capitellateleta_68020_INMT_MOUSE_Indolethylamine_N_methyltransferase_OS_Mus_musculus_OX_10090_GN_Inmt_PE_1_SV_
MetazoaAnnelidaEP00103Capitellateleta_66091_NNMT_MOUSE_Nicotinamide_N_methyltransferase_OS_Mus_musculus_OX_10090_GN_Nnmt_PE_1_SV_
MetazoaArthropodaStegodyphusmimosarum_1147183_INMT_PONAB_Indolethylamine_N_methyltransferase_OS_Pongo_abelii_OX_9601_GN_INMT_PE_2_SV_
MetazoaArthropodaStegodyphusmimosarum_1170723_INMT_PONAB_Indolethylamine_N_methyltransferase_OS_Pongo_abelii_OX_9601_GN_INMT_PE_2_SV_
MetazoaArthropodaStegodyphusmimosarum_1160607_NNMT_HUMAN_Nicotinamide_N_methyltransferase_OS_Homo_sapiens_OX_9606_GN_NNMT_PE_1_SV_
MetazoaArthropodaStegodyphusmimosarum_1161276_NNMT_MOUSE_Nicotinamide_N_methyltransferase_OS_Mus_musculus_OX_10090_GN_Nnmt_PE_1_SV_
MetazoaArthropodaStegodyphusmimosarum_1158319_INMT_PONAB_Indolethylamine_N_methyltransferase_OS_Pongo_abelii_OX_9601_GN_INMT_PE_2_SV_
MetazoaBrachiopodaLingulaunguis_1283927_INMT_RABIT_Indolethylamine_N_methyltransferase_OS_Oryctolagus_cuniculus_OX_9986_GN_INMT_PE_1_SV_
MetazoaBrachiopodaLingulaunguis_1283930_INMT_RABIT_Indolethylamine_N_methyltransferase_OS_Oryctolagus_cuniculus_OX_9986_GN_INMT_PE_1_SV_
MetazoaBrachiopodaLingulaunguis_1287218_INMT_RABIT_Indolethylamine_N_methyltransferase_OS_Oryctolagus_cuniculus_OX_9986_GN_INMT_PE_1_SV_
MetazoaBrachiopodaLingulaunguis_1284210_INMT_RABIT_Indolethylamine_N_methyltransferase_OS_Oryctolagus_cuniculus_OX_9986_GN_INMT_PE_1_SV_
MetazoaBrachiopodaLingulaunguis_1278217_NNMT_HUMAN_Nicotinamide_N_methyltransferase_OS_Homo_sapiens_OX_9606_GN_NNMT_PE_1_SV_
MetazoaBrachiopodaLingulaunguis_1289389_NNMT_HUMAN_Nicotinamide_N_methyltransferase_OS_Homo_sapiens_OX_9606_GN_NNMT_PE_1_SV_
MetazoaBrachiopodaLingulaunguis_1280242_NNMT_MOUSE_Nicotinamide_N_methyltransferase_OS_Mus_musculus_OX_10090_GN_Nnmt_PE_1_SV_
MetazoaBrachiopodaLingulaunguis_1301018_NNMT_MOUSE_Nicotinamide_N_methyltransferase_OS_Mus_musculus_OX_10090_GN_Nnmt_PE_1_SV_
MetazoaBrachiopodaLingulaunguis_1279847_INMT_RABIT_Indolethylamine_N_methyltransferase_OS_Oryctolagus_cuniculus_OX_9986_GN_INMT_PE_1_SV_
MetazoaBrachiopodaLingulaunguis_1283568_INMT_RABIT_Indolethylamine_N_methyltransferase_OS_Oryctolagus_cuniculus_OX_9986_GN_INMT_PE_1_SV_
MetazoaBrachiopodaLingulaunguis_1283833_INMT_RABIT_Indolethylamine_N_methyltransferase_OS_Oryctolagus_cuniculus_OX_9986_GN_INMT_PE_1_SV_
MetazoaBrachiopodaLingulaunguis_1283560_INMT_HUMAN_Indolethylamine_N_methyltransferase_OS_Homo_sapiens_OX_9606_GN_INMT_PE_1_SV_
MetazoaBrachiopodaLingulaunguis_1287344_NNMT_MOUSE_Nicotinamide_N_methyltransferase_OS_Mus_musculus_OX_10090_GN_Nnmt_PE_1_SV_
MetazoaBrachiopodaLingulaunguis_1302323_INMT_RABIT_Indolethylamine_N_methyltransferase_OS_Oryctolagus_cuniculus_OX_9986_GN_INMT_PE_1_SV_
MetazoaMolluscaEP00107Lottiagigantea_4180881_INMT_MOUSE_Indolethylamine_N_methyltransferase_OS_Mus_musculus_OX_10090_GN_Inmt_PE_1_SV_
MetazoaArthropodaDaphniapulex_195117_NNMT_HUMAN_Nicotinamide_N_methyltransferase_OS_Homo_sapiens_OX_9606_GN_NNMT_PE_1_SV_
MetazoaArthropodaDaphniapulex_195472_NNMT_HUMAN_Nicotinamide_N_methyltransferase_OS_Homo_sapiens_OX_9606_GN_NNMT_PE_1_SV_
MetazoaNematodaCaenorhabditiselegans_4400157_NNMT1_CAEEL_Nicotinamide_N_methyltransferase_OS_Caenorhabditis_elegans_OX_6239_GN_anmt_1_PE_3_SV_
MetazoaCnidariaHelioporacoerulea_174843_NNMT_MOUSE_Nicotinamide_N_methyltransferase_OS_Mus_musculus_OX_10090_GN_Nnmt_PE_1_SV_
MetazoaCnidariaHelioporacoerulea_174845_NNMT_PIG_Nicotinamide_N_methyltransferase_OS_Sus_scrofa_OX_9823_GN_NNMT_PE_2_SV_
MetazoaCnidariaParamuriceabiscaya_435640_NNMT_HUMAN_Nicotinamide_N_methyltransferase_OS_Homo_sapiens_OX_9606_GN_NNMT_PE_1_SV_
MetazoaCnidariaParamuriceabiscaya_424197_NNMT_MOUSE_Nicotinamide_N_methyltransferase_OS_Mus_musculus_OX_10090_GN_Nnmt_PE_1_SV_
MetazoaCnidariaParamuriceabiscaya_424198_NNMT_MOUSE_Nicotinamide_N_methyltransferase_OS_Mus_musculus_OX_10090_GN_Nnmt_PE_1_SV_
MetazoaCnidariaParamuriceabiscaya_424199_NNMT_MOUSE_Nicotinamide_N_methyltransferase_OS_Mus_musculus_OX_10090_GN_Nnmt_PE_1_SV_
MetazoaCnidariaRenillakoellikeri_480077_INMT_MOUSE_Indolethylamine_N_methyltransferase_OS_Mus_musculus_OX_10090_GN_Inmt_PE_1_SV_
MetazoaCnidariaRenillakoellikeri_490119_INMT_MOUSE_Indolethylamine_N_methyltransferase_OS_Mus_musculus_OX_10090_GN_Inmt_PE_1_SV_
MetazoaCnidariaRenillakoellikeri_468270_INMT_RABIT_Indolethylamine_N_methyltransferase_OS_Oryctolagus_cuniculus_OX_9986_GN_INMT_PE_1_SV_
MetazoaCnidariaFungiascutaria_3194333_INMT_MOUSE_Indolethylamine_N_methyltransferase_OS_Mus_musculus_OX_10090_GN_Inmt_PE_1_SV_
MetazoaCnidariaNematostellavectensis_011120NNMT_MOUSE_Nicotinamide_N_methyltransferase_OS_Mus_musculus_OX_10090_GN_Nnmt_PE_1_SV_
MetazoaPoriferaAmphimedonqueenslandica_4844236_NNMT_MOUSE_Nicotinamide_N_methyltransferase_OS_Mus_musculus_OX_10090_GN_Nnmt_PE_1_SV_
MetazoaPoriferaAmphimedonqueenslandica_4845102_NNMT_PIG_Nicotinamide_N_methyltransferase_OS_Sus_scrofa_OX_9823_GN_NNMT_PE_2_SV_
MetazoaPoriferaAmphimedonqueenslandica_4844046_NNMT_HUMAN_Nicotinamide_N_methyltransferase_OS_Homo_sapiens_OX_9606_GN_NNMT_PE_1_SV_
MetazoaPoriferaEP00118Oscarellapearsei_4829278_PNMT_HUMAN_Phenylethanolamine_N_methyltransferase_OS_Homo_sapiens_OX_9606_GN_PNMT_PE_1_SV_
MetazoaPoriferaEP00118Oscarellapearsei_4833788_NNMT_MOUSE_Nicotinamide_N_methyltransferase_OS_Mus_musculus_OX_10090_GN_Nnmt_PE_1_SV_
0.24

6E.

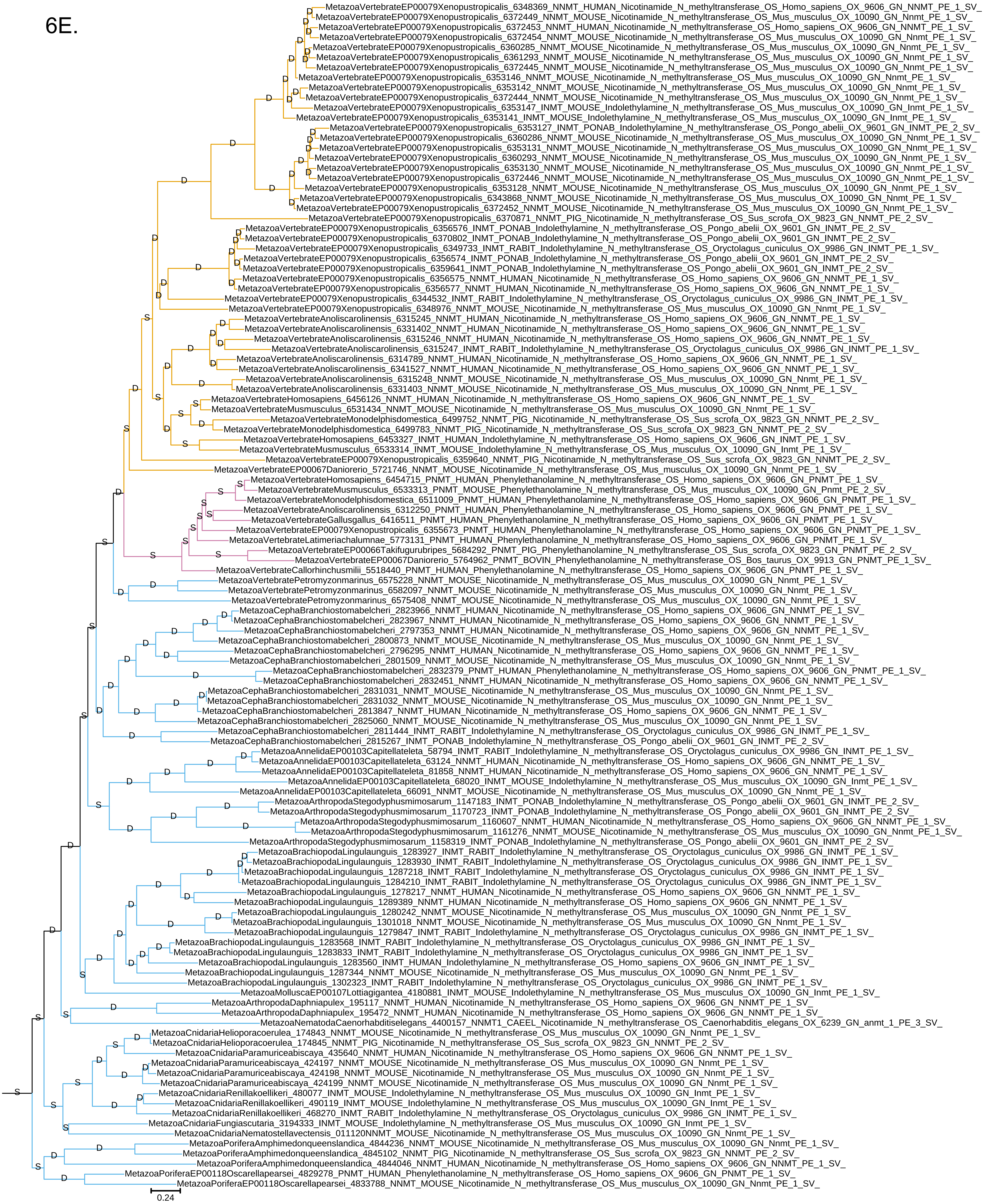

MetazoaVertebrateEP00079Xenopustropicalis_6348369_NNMT_HUMAN_Nicotinamide_N_methyltransferase_OS_Homo_sapiens_OX_9606_GN_NNMT_PE_1_SV_
MetazoaVertebrateEP00079Xenopustropicalis_6372449_NNMT_MOUSE_Nicotinamide_N_methyltransferase_OS_Mus_musculus_OX_10090_GN_Nnmt_PE_1_SV_
MetazoaVertebrateEP00079Xenopustropicalis_6372453_NNMT_HUMAN_Nicotinamide_N_methyltransferase_OS_Homo_sapiens_OX_9606_GN_NNMT_PE_1_SV_
MetazoaVertebrateEP00079Xenopustropicalis_6372454_NNMT_MOUSE_Nicotinamide_N_methyltransferase_OS_Mus_musculus_OX_10090_GN_Nnmt_PE_1_SV_
MetazoaVertebrateEP00079Xenopustropicalis_6360285_NNMT_MOUSE_Nicotinamide_N_methyltransferase_OS_Mus_musculus_OX_10090_GN_Nnmt_PE_1_SV_
MetazoaVertebrateEP00079Xenopustropicalis_6361293_NNMT_MOUSE_Nicotinamide_N_methyltransferase_OS_Mus_musculus_OX_10090_GN_Nnmt_PE_1_SV_
MetazoaVertebrateEP00079Xenopustropicalis_6372445_NNMT_MOUSE_Nicotinamide_N_methyltransferase_OS_Mus_musculus_OX_10090_GN_Nnmt_PE_1_SV_
MetazoaVertebrateEP00079Xenopustropicalis_6353146_NNMT_MOUSE_Nicotinamide_N_methyltransferase_OS_Mus_musculus_OX_10090_GN_Nnmt_PE_1_SV_
MetazoaVertebrateEP00079Xenopustropicalis_6353142_NNMT_MOUSE_Nicotinamide_N_methyltransferase_OS_Mus_musculus_OX_10090_GN_Nnmt_PE_1_SV_
MetazoaVertebrateEP00079Xenopustropicalis_6372444_NNMT_MOUSE_Nicotinamide_N_methyltransferase_OS_Mus_musculus_OX_10090_GN_Nnmt_PE_1_SV_
MetazoaVertebrateEP00079Xenopustropicalis_6353147_INMT_MOUSE_Indolethylamine_N_methyltransferase_OS_Mus_musculus_OX_10090_GN_Inmt_PE_1_SV_
MetazoaVertebrateEP00079Xenopustropicalis_6353141_INMT_MOUSE_Indolethylamine_N_methyltransferase_OS_Mus_musculus_OX_10090_GN_Inmt_PE_1_SV_
MetazoaVertebrateEP00079Xenopustropicalis_6353127_INMT_PONAB_Indolethylamine_N_methyltransferase_OS_Pongo_abelii_OX_9601_GN_INMT_PE_2_SV_
MetazoaVertebrateEP00079Xenopustropicalis_6360286_NNMT_MOUSE_Nicotinamide_N_methyltransferase_OS_Mus_musculus_OX_10090_GN_Nnmt_PE_1_SV_
MetazoaVertebrateEP00079Xenopustropicalis_6353131_NNMT_MOUSE_Nicotinamide_N_methyltransferase_OS_Mus_musculus_OX_10090_GN_Nnmt_PE_1_SV_
MetazoaVertebrateEP00079Xenopustropicalis_6360293_NNMT_MOUSE_Nicotinamide_N_methyltransferase_OS_Mus_musculus_OX_10090_GN_Nnmt_PE_1_SV_
MetazoaVertebrateEP00079Xenopustropicalis_6353130_NNMT_MOUSE_Nicotinamide_N_methyltransferase_OS_Mus_musculus_OX_10090_GN_Nnmt_PE_1_SV_
MetazoaVertebrateEP00079Xenopustropicalis_6372446_NNMT_MOUSE_Nicotinamide_N_methyltransferase_OS_Mus_musculus_OX_10090_GN_Nnmt_PE_1_SV_
MetazoaVertebrateEP00079Xenopustropicalis_6353128_NNMT_MOUSE_Nicotinamide_N_methyltransferase_OS_Mus_musculus_OX_10090_GN_Nnmt_PE_1_SV_
MetazoaVertebrateEP00079Xenopustropicalis_6343868_NNMT_MOUSE_Nicotinamide_N_methyltransferase_OS_Mus_musculus_OX_10090_GN_Nnmt_PE_1_SV_
MetazoaVertebrateEP00079Xenopustropicalis_6372452_NNMT_MOUSE_Nicotinamide_N_methyltransferase_OS_Mus_musculus_OX_10090_GN_Nnmt_PE_1_SV_
MetazoaVertebrateEP00079Xenopustropicalis_6370871_NNMT_PIG_Nicotinamide_N_methyltransferase_OS_Sus_scrofa_OX_9823_GN_NNMT_PE_2_SV_
MetazoaVertebrateEP00079Xenopustropicalis_6356576_INMT_PONAB_Indolethylamine_N_methyltransferase_OS_Pongo_abelii_OX_9601_GN_INMT_PE_2_SV_
MetazoaVertebrateEP00079Xenopustropicalis_6370802_INMT_PONAB_Indolethylamine_N_methyltransferase_OS_Pongo_abelii_OX_9601_GN_INMT_PE_2_SV_
MetazoaVertebrateEP00079Xenopustropicalis_6349733_INMT_RABIT_Indolethylamine_N_methyltransferase_OS_Oryctolagus_cuniculus_OX_9986_GN_INMT_PE_1_SV_
MetazoaVertebrateEP00079Xenopustropicalis_6356574_INMT_PONAB_Indolethylamine_N_methyltransferase_OS_Pongo_abelii_OX_9601_GN_INMT_PE_2_SV_
MetazoaVertebrateEP00079Xenopustropicalis_6359641_INMT_PONAB_Indolethylamine_N_methyltransferase_OS_Pongo_abelii_OX_9601_GN_INMT_PE_2_SV_
MetazoaVertebrateEP00079Xenopustropicalis_6356575_NNMT_HUMAN_Nicotinamide_N_methyltransferase_OS_Homo_sapiens_OX_9606_GN_NNMT_PE_1_SV_
MetazoaVertebrateEP00079Xenopustropicalis_6356577_NNMT_HUMAN_Nicotinamide_N_methyltransferase_OS_Homo_sapiens_OX_9606_GN_NNMT_PE_1_SV_
MetazoaVertebrateEP00079Xenopustropicalis_6344532_INMT_RABIT_Indolethylamine_N_methyltransferase_OS_Oryctolagus_cuniculus_OX_9986_GN_INMT_PE_1_SV_
MetazoaVertebrateEP00079Xenopustropicalis_6348976_NNMT_MOUSE_Nicotinamide_N_methyltransferase_OS_Mus_musculus_OX_10090_GN_Nnmt_PE_1_SV_
MetazoaVertebrateAnoliscarolinensis_6315245_NNMT_HUMAN_Nicotinamide_N_methyltransferase_OS_Homo_sapiens_OX_9606_GN_NNMT_PE_1_SV_
MetazoaVertebrateAnoliscarolinensis_6331402_NNMT_HUMAN_Nicotinamide_N_methyltransferase_OS_Homo_sapiens_OX_9606_GN_NNMT_PE_1_SV_
MetazoaVertebrateAnoliscarolinensis_6315246_NNMT_HUMAN_Nicotinamide_N_methyltransferase_OS_Homo_sapiens_OX_9606_GN_NNMT_PE_1_SV_
MetazoaVertebrateAnoliscarolinensis_6315247_INMT_RABIT_Indolethylamine_N_methyltransferase_OS_Oryctolagus_cuniculus_OX_9986_GN_INMT_PE_1_SV_
MetazoaVertebrateAnoliscarolinensis_6314789_NNMT_HUMAN_Nicotinamide_N_methyltransferase_OS_Homo_sapiens_OX_9606_GN_NNMT_PE_1_SV_
MetazoaVertebrateAnoliscarolinensis_6341527_NNMT_HUMAN_Nicotinamide_N_methyltransferase_OS_Homo_sapiens_OX_9606_GN_NNMT_PE_1_SV_
MetazoaVertebrateAnoliscarolinensis_6315248_NNMT_MOUSE_Nicotinamide_N_methyltransferase_OS_Mus_musculus_OX_10090_GN_Nnmt_PE_1_SV_
MetazoaVertebrateAnoliscarolinensis_6331403_NNMT_MOUSE_Nicotinamide_N_methyltransferase_OS_Mus_musculus_OX_10090_GN_Nnmt_PE_1_SV_
MetazoaVertebrateHomosapiens_6456126_NNMT_HUMAN_Nicotinamide_N_methyltransferase_OS_Homo_sapiens_OX_9606_GN_NNMT_PE_1_SV_
MetazoaVertebrateMusmusculus_6531434_NNMT_MOUSE_Nicotinamide_N_methyltransferase_OS_Mus_musculus_OX_10090_GN_Nnmt_PE_1_SV_
MetazoaVertebrateMonodelphisdomestica_6499752_NNMT_PIG_Nicotinamide_N_methyltransferase_OS_Sus_scrofa_OX_9823_GN_NNMT_PE_2_SV_
MetazoaVertebrateMonodelphisdomestica_6499783_NNMT_PIG_Nicotinamide_N_methyltransferase_OS_Sus_scrofa_OX_9823_GN_NNMT_PE_2_SV_
MetazoaVertebrateHomosapiens_6453327_INMT_HUMAN_Indolethylamine_N_methyltransferase_OS_Homo_sapiens_OX_9606_GN_INMT_PE_1_SV_
MetazoaVertebrateMusmusculus_6533314_INMT_MOUSE_Indolethylamine_N_methyltransferase_OS_Mus_musculus_OX_10090_GN_Inmt_PE_1_SV_
MetazoaVertebrateEP00079Xenopustropicalis_6359640_NNMT_PIG_Nicotinamide_N_methyltransferase_OS_Sus_scrofa_OX_9823_GN_NNMT_PE_2_SV_
MetazoaVertebrateEP00067Daniorerio_5721746_NNMT_MOUSE_Nicotinamide_N_methyltransferase_OS_Mus_musculus_OX_10090_GN_Nnmt_PE_1_SV_
MetazoaVertebrateHomosapiens_6454715_PNMT_HUMAN_Phenylethanolamine_N_methyltransferase_OS_Homo_sapiens_OX_9606_GN_PNMT_PE_1_SV_
MetazoaVertebrateMusmusculus_6533313_PNMT_MOUSE_Phenylethanolamine_N_methyltransferase_OS_Mus_musculus_OX_10090_GN_Pnmt_PE_2_SV_
MetazoaVertebrateMonodelphisdomestica_6511009_PNMT_HUMAN_Phenylethanolamine_N_methyltransferase_OS_Homo_sapiens_OX_9606_GN_PNMT_PE_1_SV_
MetazoaVertebrateAnoliscarolinensis_6312250_PNMT_HUMAN_Phenylethanolamine_N_methyltransferase_OS_Homo_sapiens_OX_9606_GN_PNMT_PE_1_SV_
MetazoaVertebrateGallusgallus_6416511_PNMT_HUMAN_Phenylethanolamine_N_methyltransferase_OS_Homo_sapiens_OX_9606_GN_PNMT_PE_1_SV_
MetazoaVertebrateEP00079Xenopustropicalis_6355673_PNMT_HUMAN_Phenylethanolamine_N_methyltransferase_OS_Homo_sapiens_OX_9606_GN_PNMT_PE_1_SV_
MetazoaVertebrateLatimeriachalumnae_5773131_PNMT_HUMAN_Phenylethanolamine_N_methyltransferase_OS_Homo_sapiens_OX_9606_GN_PNMT_PE_1_SV_
MetazoaVertebrateEP00066Takifugurubripes_5684292_PNMT_PIG_Phenylethanolamine_N_methyltransferase_OS_Sus_scrofa_OX_9823_GN_PNMT_PE_2_SV_
MetazoaVertebrateEP00067Daniorerio_5764962_PNMT_BOVIN_Phenylethanolamine_N_methyltransferase_OS_Bos_taurus_OX_9913_GN_PNMT_PE_1_SV_
MetazoaVertebrateCallorhinchusmilii_5518440_PNMT_HUMAN_Phenylethanolamine_N_methyltransferase_OS_Homo_sapiens_OX_9606_GN_PNMT_PE_1_SV_
MetazoaVertebratePetromyzonmarinus_6575228_NNMT_MOUSE_Nicotinamide_N_methyltransferase_OS_Mus_musculus_OX_10090_GN_Nnmt_PE_1_SV_
MetazoaVertebratePetromyzonmarinus_6582097_NNMT_MOUSE_Nicotinamide_N_methyltransferase_OS_Mus_musculus_OX_10090_GN_Nnmt_PE_1_SV_
MetazoaVertebratePetromyzonmarinus_6575408_NNMT_MOUSE_Nicotinamide_N_methyltransferase_OS_Mus_musculus_OX_10090_GN_Nnmt_PE_1_SV_
MetazoaCephaBranchiostomabelcheri_2823966_NNMT_HUMAN_Nicotinamide_N_methyltransferase_OS_Homo_sapiens_OX_9606_GN_NNMT_PE_1_SV_
MetazoaCephaBranchiostomabelcheri_2823967_NNMT_HUMAN_Nicotinamide_N_methyltransferase_OS_Homo_sapiens_OX_9606_GN_NNMT_PE_1_SV_
MetazoaCephaBranchiostomabelcheri_2797353_NNMT_HUMAN_Nicotinamide_N_methyltransferase_OS_Homo_sapiens_OX_9606_GN_NNMT_PE_1_SV_
MetazoaCephaBranchiostomabelcheri_2800873_NNMT_MOUSE_Nicotinamide_N_methyltransferase_OS_Mus_musculus_OX_10090_GN_Nnmt_PE_1_SV_
MetazoaCephaBranchiostomabelcheri_2796295_NNMT_HUMAN_Nicotinamide_N_methyltransferase_OS_Homo_sapiens_OX_9606_GN_NNMT_PE_1_SV_
MetazoaCephaBranchiostomabelcheri_2801509_NNMT_MOUSE_Nicotinamide_N_methyltransferase_OS_Mus_musculus_OX_10090_GN_Nnmt_PE_1_SV_
MetazoaCephaBranchiostomabelcheri_2832379_PNMT_HUMAN_Phenylethanolamine_N_methyltransferase_OS_Homo_sapiens_OX_9606_GN_PNMT_PE_1_SV_
MetazoaCephaBranchiostomabelcheri_2832451_NNMT_HUMAN_Nicotinamide_N_methyltransferase_OS_Homo_sapiens_OX_9606_GN_NNMT_PE_1_SV_
MetazoaCephaBranchiostomabelcheri_2831031_NNMT_MOUSE_Nicotinamide_N_methyltransferase_OS_Mus_musculus_OX_10090_GN_Nnmt_PE_1_SV_
MetazoaCephaBranchiostomabelcheri_2831032_NNMT_MOUSE_Nicotinamide_N_methyltransferase_OS_Mus_musculus_OX_10090_GN_Nnmt_PE_1_SV_
MetazoaCephaBranchiostomabelcheri_2813847_NNMT_HUMAN_Nicotinamide_N_methyltransferase_OS_Homo_sapiens_OX_9606_GN_NNMT_PE_1_SV_
MetazoaCephaBranchiostomabelcheri_2825060_NNMT_MOUSE_Nicotinamide_N_methyltransferase_OS_Mus_musculus_OX_10090_GN_Nnmt_PE_1_SV_
MetazoaCephaBranchiostomabelcheri_2811444_INMT_RABIT_Indolethylamine_N_methyltransferase_OS_Oryctolagus_cuniculus_OX_9986_GN_INMT_PE_1_SV_
MetazoaCephaBranchiostomabelcheri_2815267_INMT_PONAB_Indolethylamine_N_methyltransferase_OS_Pongo_abelii_OX_9601_GN_INMT_PE_2_SV_
MetazoaAnnelidaEP00103Capitellateleta_58794_INMT_RABIT_Indolethylamine_N_methyltransferase_OS_Oryctolagus_cuniculus_OX_9986_GN_INMT_PE_1_SV_
MetazoaAnnelidaEP00103Capitellateleta_63124_NNMT_HUMAN_Nicotinamide_N_methyltransferase_OS_Homo_sapiens_OX_9606_GN_NNMT_PE_1_SV_
MetazoaAnnelidaEP00103Capitellateleta_81858_NNMT_HUMAN_Nicotinamide_N_methyltransferase_OS_Homo_sapiens_OX_9606_GN_NNMT_PE_1_SV_
MetazoaAnnelidaEP00103Capitellateleta_68020_INMT_MOUSE_Indolethylamine_N_methyltransferase_OS_Mus_musculus_OX_10090_GN_Inmt_PE_1_SV_
MetazoaAnnelidaEP00103Capitellateleta_66091_NNMT_MOUSE_Nicotinamide_N_methyltransferase_OS_Mus_musculus_OX_10090_GN_Nnmt_PE_1_SV_
MetazoaArthropodaStegodyphusmimosarum_1147183_INMT_PONAB_Indolethylamine_N_methyltransferase_OS_Pongo_abelii_OX_9601_GN_INMT_PE_2_SV_
MetazoaArthropodaStegodyphusmimosarum_1170723_INMT_PONAB_Indolethylamine_N_methyltransferase_OS_Pongo_abelii_OX_9601_GN_INMT_PE_2_SV_
MetazoaArthropodaStegodyphusmimosarum_1160607_NNMT_HUMAN_Nicotinamide_N_methyltransferase_OS_Homo_sapiens_OX_9606_GN_NNMT_PE_1_SV_
MetazoaArthropodaStegodyphusmimosarum_1161276_NNMT_MOUSE_Nicotinamide_N_methyltransferase_OS_Mus_musculus_OX_10090_GN_Nnmt_PE_1_SV_
MetazoaArthropodaStegodyphusmimosarum_1158319_INMT_PONAB_Indolethylamine_N_methyltransferase_OS_Pongo_abelii_OX_9601_GN_INMT_PE_2_SV_
MetazoaBrachiopodaLingulaunguis_1283927_INMT_RABIT_Indolethylamine_N_methyltransferase_OS_Oryctolagus_cuniculus_OX_9986_GN_INMT_PE_1_SV_
MetazoaBrachiopodaLingulaunguis_1283930_INMT_RABIT_Indolethylamine_N_methyltransferase_OS_Oryctolagus_cuniculus_OX_9986_GN_INMT_PE_1_SV_
MetazoaBrachiopodaLingulaunguis_1287218_INMT_RABIT_Indolethylamine_N_methyltransferase_OS_Oryctolagus_cuniculus_OX_9986_GN_INMT_PE_1_SV_
MetazoaBrachiopodaLingulaunguis_1284210_INMT_RABIT_Indolethylamine_N_methyltransferase_OS_Oryctolagus_cuniculus_OX_9986_GN_INMT_PE_1_SV_
MetazoaBrachiopodaLingulaunguis_1278217_NNMT_HUMAN_Nicotinamide_N_methyltransferase_OS_Homo_sapiens_OX_9606_GN_NNMT_PE_1_SV_
MetazoaBrachiopodaLingulaunguis_1289389_NNMT_HUMAN_Nicotinamide_N_methyltransferase_OS_Homo_sapiens_OX_9606_GN_NNMT_PE_1_SV_
MetazoaBrachiopodaLingulaunguis_1280242_NNMT_MOUSE_Nicotinamide_N_methyltransferase_OS_Mus_musculus_OX_10090_GN_Nnmt_PE_1_SV_
MetazoaBrachiopodaLingulaunguis_1301018_NNMT_MOUSE_Nicotinamide_N_methyltransferase_OS_Mus_musculus_OX_10090_GN_Nnmt_PE_1_SV_
MetazoaBrachiopodaLingulaunguis_1279847_INMT_RABIT_Indolethylamine_N_methyltransferase_OS_Oryctolagus_cuniculus_OX_9986_GN_INMT_PE_1_SV_
MetazoaBrachiopodaLingulaunguis_1283568_INMT_RABIT_Indolethylamine_N_methyltransferase_OS_Oryctolagus_cuniculus_OX_9986_GN_INMT_PE_1_SV_
MetazoaBrachiopodaLingulaunguis_1283833_INMT_RABIT_Indolethylamine_N_methyltransferase_OS_Oryctolagus_cuniculus_OX_9986_GN_INMT_PE_1_SV_
MetazoaBrachiopodaLingulaunguis_1283560_INMT_HUMAN_Indolethylamine_N_methyltransferase_OS_Homo_sapiens_OX_9606_GN_INMT_PE_1_SV_
MetazoaBrachiopodaLingulaunguis_1287344_NNMT_MOUSE_Nicotinamide_N_methyltransferase_OS_Mus_musculus_OX_10090_GN_Nnmt_PE_1_SV_
MetazoaBrachiopodaLingulaunguis_1302323_INMT_RABIT_Indolethylamine_N_methyltransferase_OS_Oryctolagus_cuniculus_OX_9986_GN_INMT_PE_1_SV_
MetazoaMolluscaEP00107Lottiagigantea_4180881_INMT_MOUSE_Indolethylamine_N_methyltransferase_OS_Mus_musculus_OX_10090_GN_Inmt_PE_1_SV_
MetazoaArthropodaDaphniapulex_195117_NNMT_HUMAN_Nicotinamide_N_methyltransferase_OS_Homo_sapiens_OX_9606_GN_NNMT_PE_1_SV_
MetazoaArthropodaDaphniapulex_195472_NNMT_HUMAN_Nicotinamide_N_methyltransferase_OS_Homo_sapiens_OX_9606_GN_NNMT_PE_1_SV_
MetazoaNematodaCaenorhabditiselegans_4400157_NNMT1_CAEEL_Nicotinamide_N_methyltransferase_OS_Caenorhabditis_elegans_OX_6239_GN_anmt_1_PE_3_SV_
MetazoaCnidariaHelioporacoerulea_174843_NNMT_MOUSE_Nicotinamide_N_methyltransferase_OS_Mus_musculus_OX_10090_GN_Nnmt_PE_1_SV_
MetazoaCnidariaHelioporacoerulea_174845_NNMT_PIG_Nicotinamide_N_methyltransferase_OS_Sus_scrofa_OX_9823_GN_NNMT_PE_2_SV_
MetazoaCnidariaParamuriceabiscaya_435640_NNMT_HUMAN_Nicotinamide_N_methyltransferase_OS_Homo_sapiens_OX_9606_GN_NNMT_PE_1_SV_
MetazoaCnidariaParamuriceabiscaya_424197_NNMT_MOUSE_Nicotinamide_N_methyltransferase_OS_Mus_musculus_OX_10090_GN_Nnmt_PE_1_SV_
MetazoaCnidariaParamuriceabiscaya_424198_NNMT_MOUSE_Nicotinamide_N_methyltransferase_OS_Mus_musculus_OX_10090_GN_Nnmt_PE_1_SV_
MetazoaCnidariaParamuriceabiscaya_424199_NNMT_MOUSE_Nicotinamide_N_methyltransferase_OS_Mus_musculus_OX_10090_GN_Nnmt_PE_1_SV_
MetazoaCnidariaRenillakoellikeri_480077_INMT_MOUSE_Indolethylamine_N_methyltransferase_OS_Mus_musculus_OX_10090_GN_Inmt_PE_1_SV_
MetazoaCnidariaRenillakoellikeri_490119_INMT_MOUSE_Indolethylamine_N_methyltransferase_OS_Mus_musculus_OX_10090_GN_Inmt_PE_1_SV_
MetazoaCnidariaRenillakoellikeri_468270_INMT_RABIT_Indolethylamine_N_methyltransferase_OS_Oryctolagus_cuniculus_OX_9986_GN_INMT_PE_1_SV_
MetazoaCnidariaFungiascutaria_3194333_INMT_MOUSE_Indolethylamine_N_methyltransferase_OS_Mus_musculus_OX_10090_GN_Inmt_PE_1_SV_
MetazoaCnidariaNematostellavectensis_011120NNMT_MOUSE_Nicotinamide_N_methyltransferase_OS_Mus_musculus_OX_10090_GN_Nnmt_PE_1_SV_
MetazoaPoriferaAmphimedonqueenslandica_4844236_NNMT_MOUSE_Nicotinamide_N_methyltransferase_OS_Mus_musculus_OX_10090_GN_Nnmt_PE_1_SV_
MetazoaPoriferaAmphimedonqueenslandica_4845102_NNMT_PIG_Nicotinamide_N_methyltransferase_OS_Sus_scrofa_OX_9823_GN_NNMT_PE_2_SV_
MetazoaPoriferaAmphimedonqueenslandica_4844046_NNMT_HUMAN_Nicotinamide_N_methyltransferase_OS_Homo_sapiens_OX_9606_GN_NNMT_PE_1_SV_
MetazoaPoriferaEP00118Oscarellapearsei_4829278_PNMT_HUMAN_Phenylethanolamine_N_methyltransferase_OS_Homo_sapiens_OX_9606_GN_PNMT_PE_1_SV_
MetazoaPoriferaEP00118Oscarellapearsei_4833788_NNMT_MOUSE_Nicotinamide_N_methyltransferase_OS_Mus_musculus_OX_10090_GN_Nnmt_PE_1_SV_
0.24

6F.

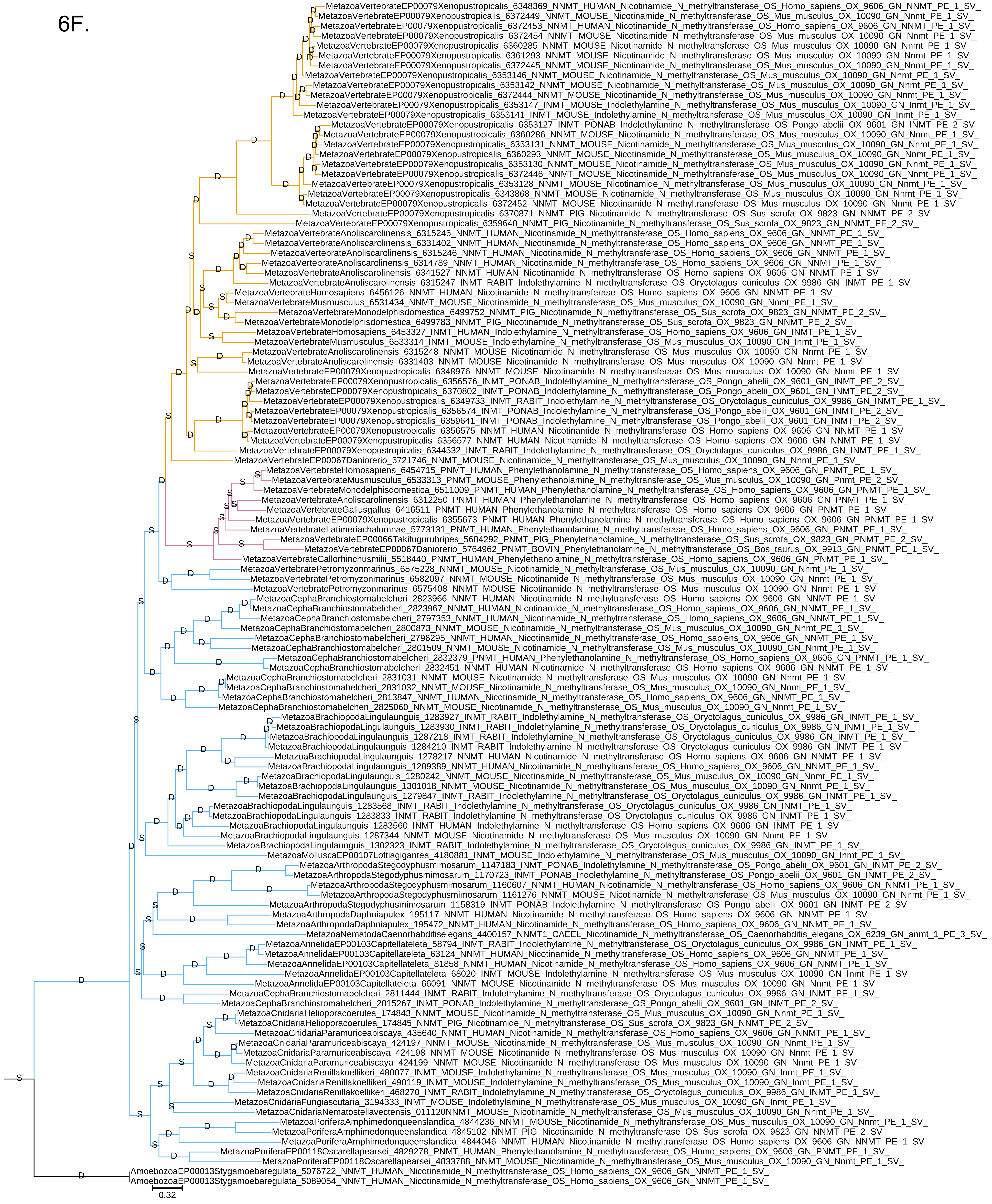

Supplementary Figure 7: Analysis of VMATs. A) TBE tree with TBE score supports. B) Maximum likelihood tree with ultrafast bootstrap proportion supports. C) TBE tree with TBE score supports without rogue sequences. D) Maximum likelihood tree with ultrafast bootstrap proportion supports without rogue sequences. E) Maximum likelihood tree including all eukaryotic sequences with ultrafast bootstrap proportion supports. F) Reconciliation using sponge-first hypothesis. G) Reconciliation without rogue sequences using sponge first hypothesis. H) Reconciliation using ctenophore-first hypothesis. I) Reconciliation without rogue sequences using ctenophore-first hypothesis. J) Reconciliation of the UFB tree including all eukaryotic sequences. Nodes in reconciled trees indicated as speciation events (S) or duplication events (D). All trees shown as rooted, non-reconciled trees rooted arbitrarily for visualisation. VMAT = vesicular monoamine transporter, TBE = transfer bootstrap expectation, UFB = ultrafast bootstrap. Branches colour coded by sequence annotation; blue = vesicular monoamine transporter; green = vesicular acetylcholine transporter; dark orange = other solute ligand carrier 18 sequences; and black = others and non-opithokont sequences. Source Data are available at [https://figshare.le.ac.uk/articles/dataset/Monoamine_Neuromodulation_is_a_Bilaterian_Innovation_Results/20391477]; in Results/GeneTrees labelled OG_VMAT_TBE.tbe.tree and OG_VMAT_UFB.treefile; in Results/RogueTaxaAnalysis/GeneTreeReanalysis labelled OG_VMAT_Rogue_TBE.tbe.tree and OG_VMAT_Rogue_UFB.treefile; in Results/GeneTrees/Eukaryotes_GeneTrees labelled OG_VMAT_EUKIQ.treefile; in Results/Reconciliation/SpongeFirst labelled OG_VMAT and OG_VMAT_Rogue; in Results/Reconciliation/CtenophoreFirst labelled OG_VMAT and OG_VMAT_Rogue; and in Results/Reconciliation/Eukaryote labelled OG_VMAT.

7A.

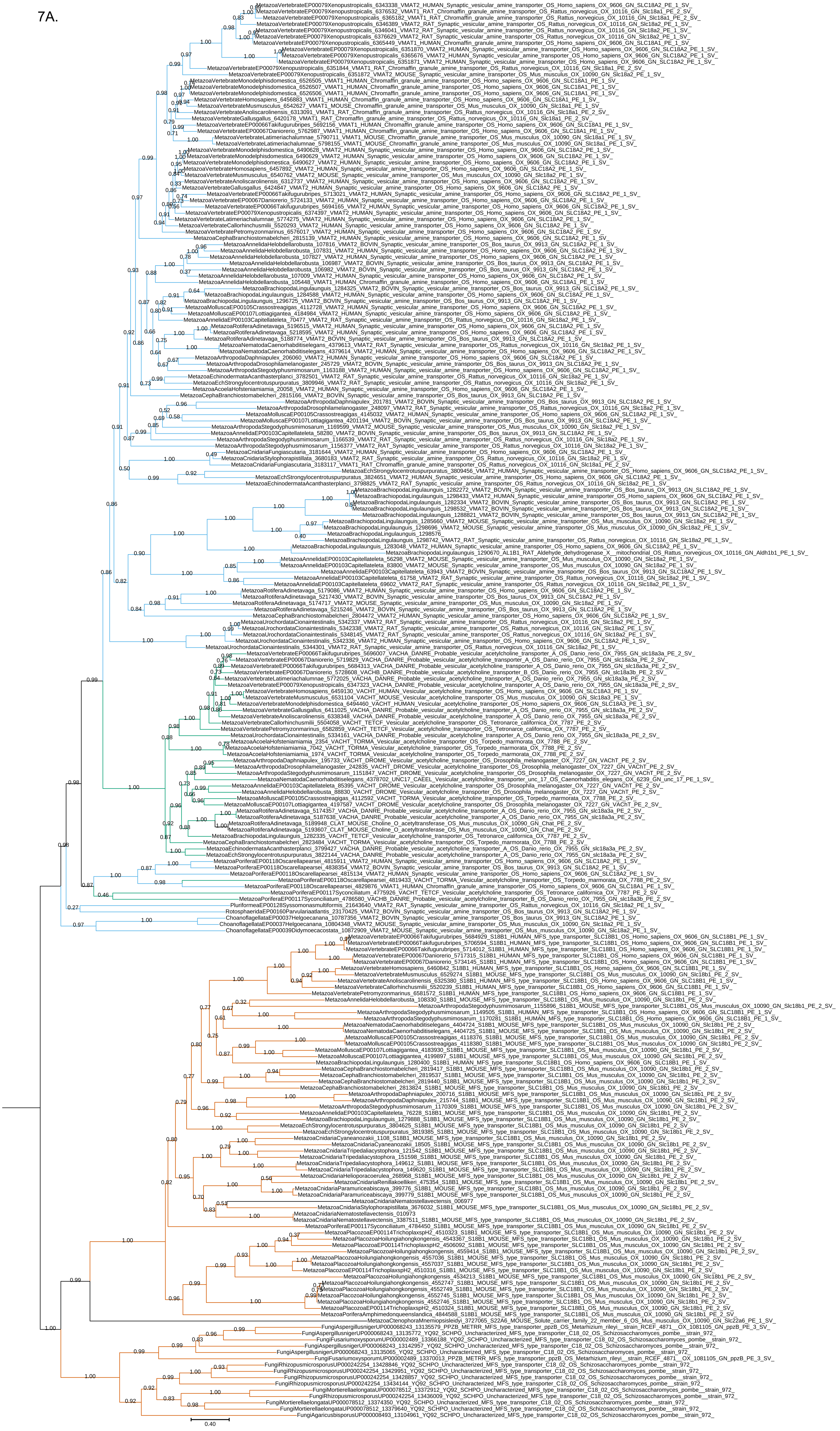

7B.

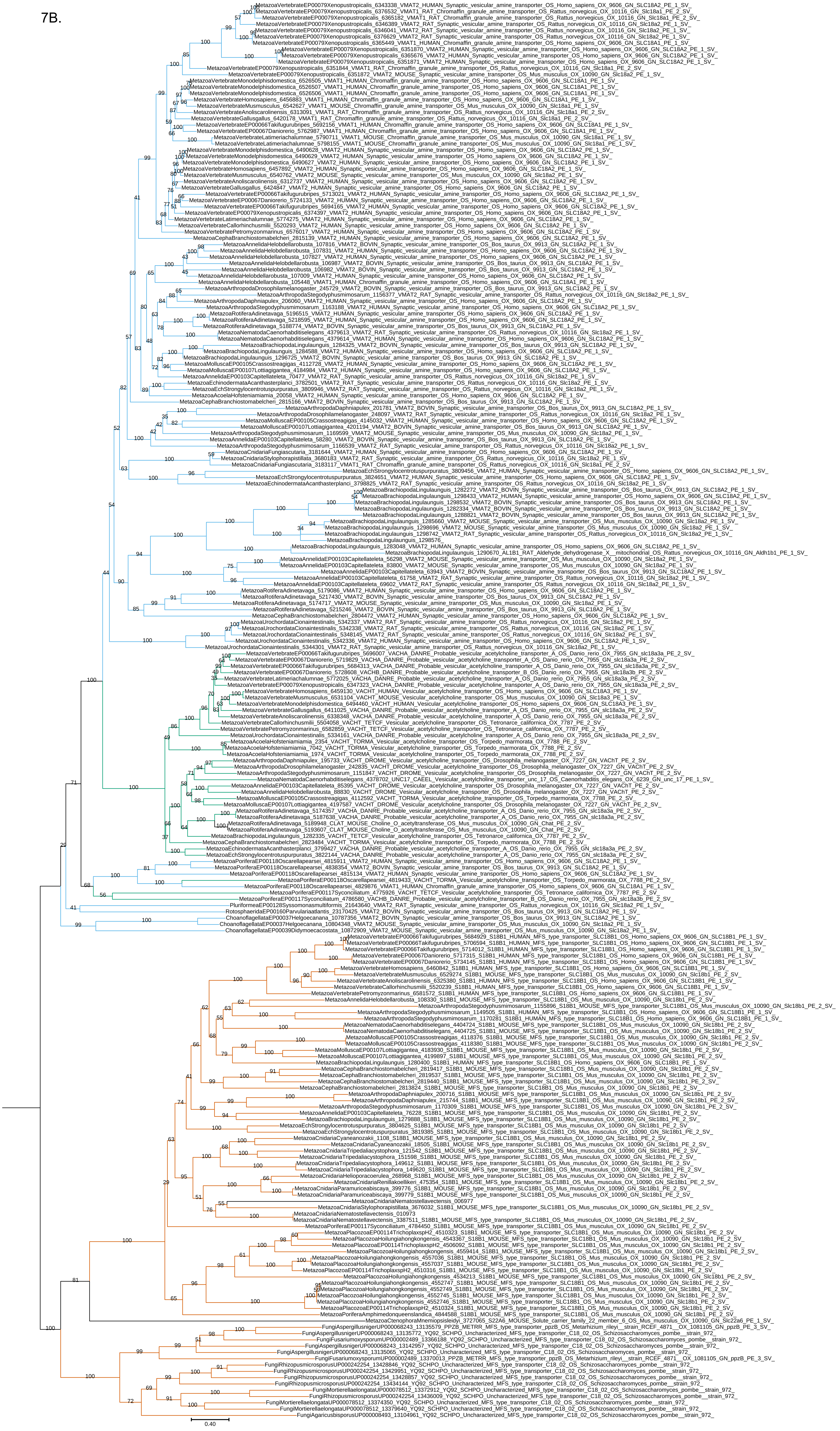

7C.

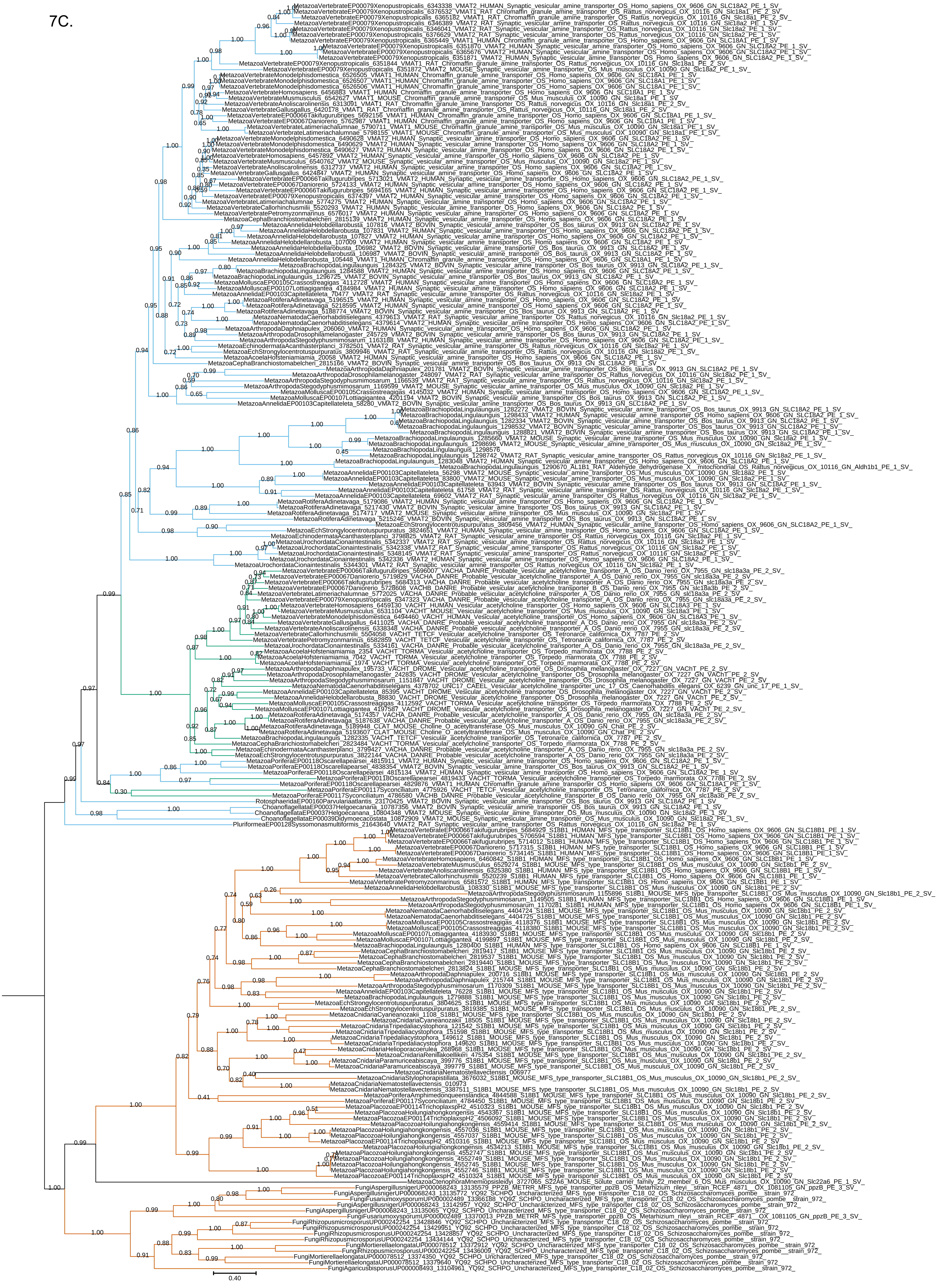

7D.

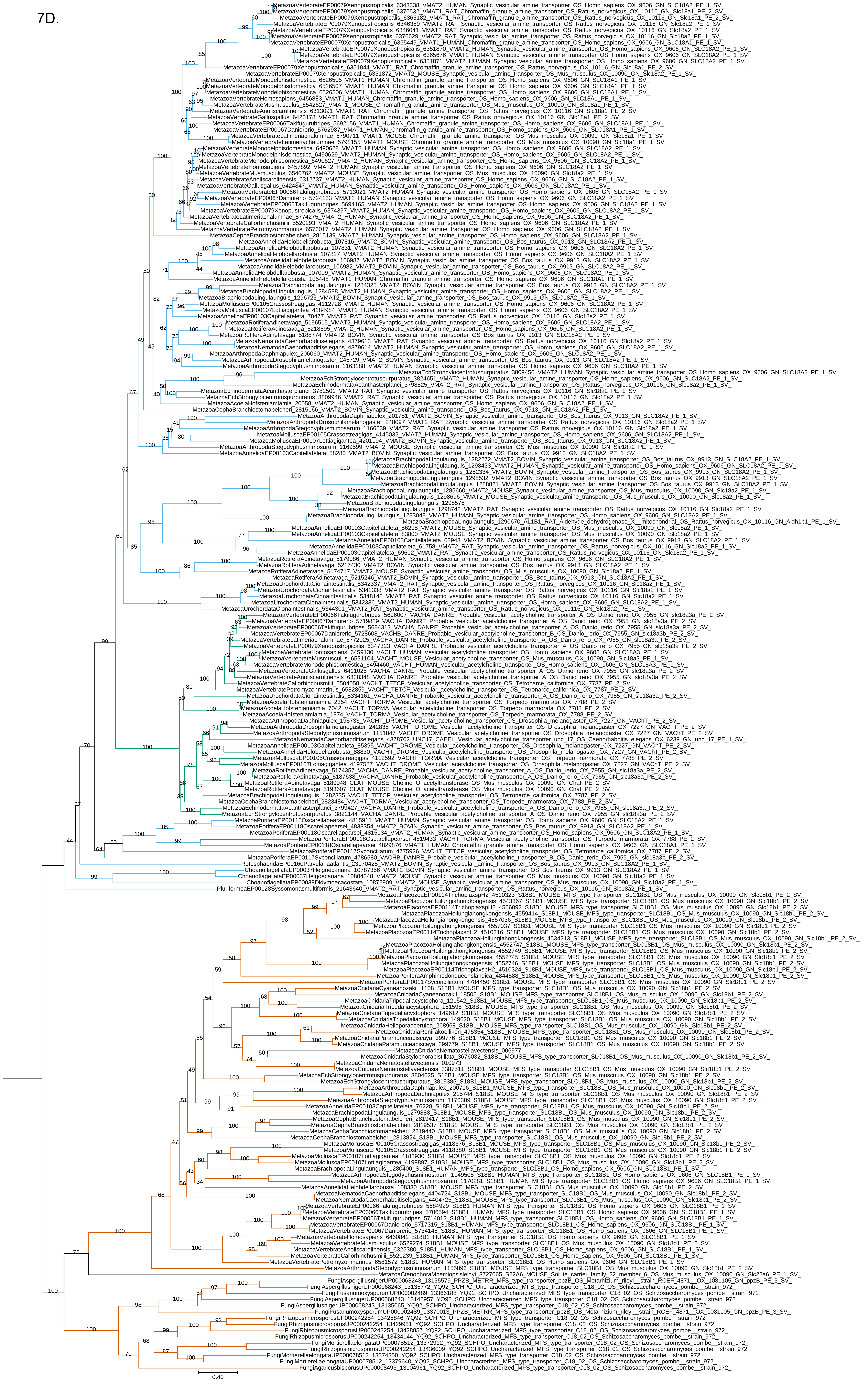

7E.

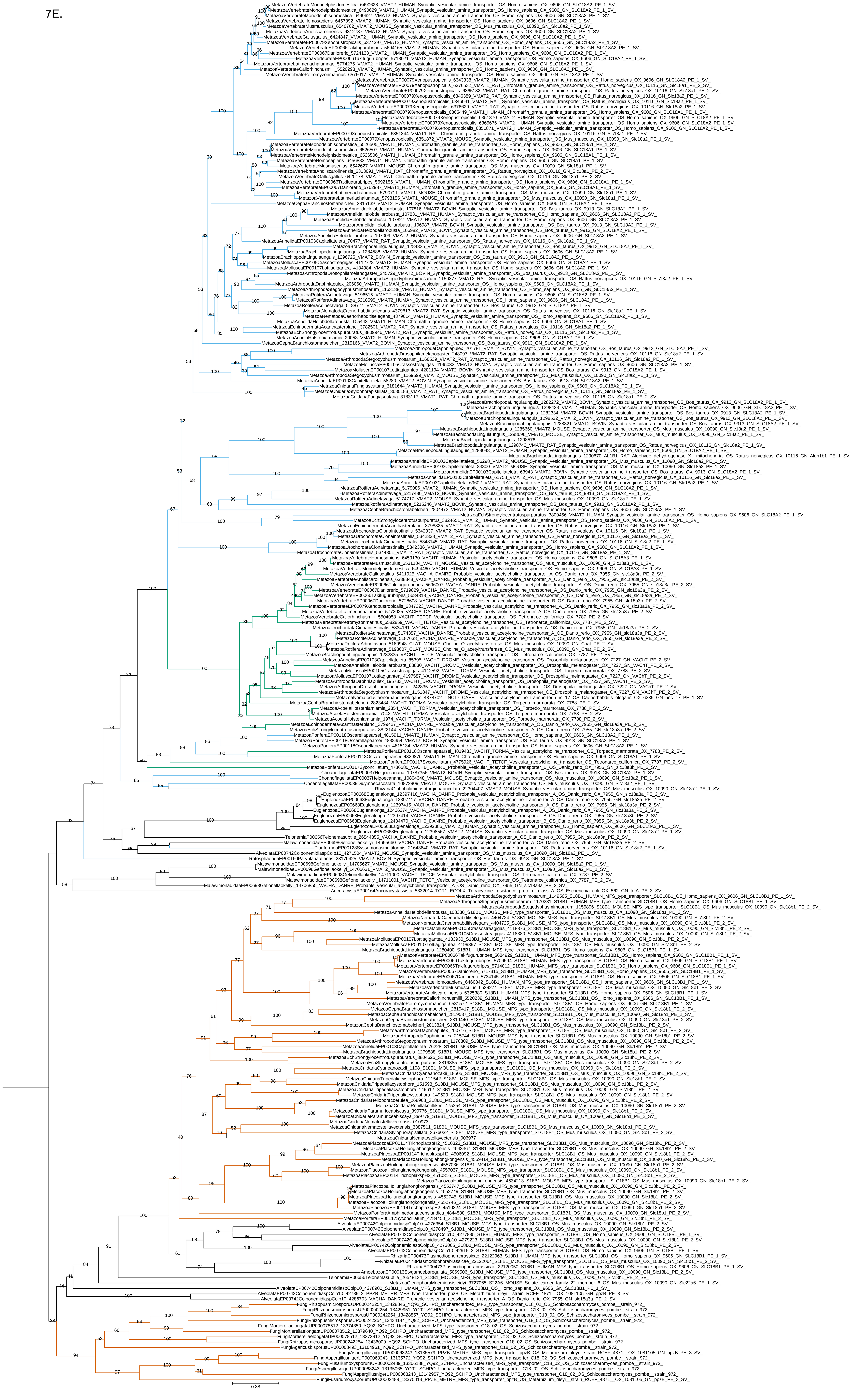

7F.

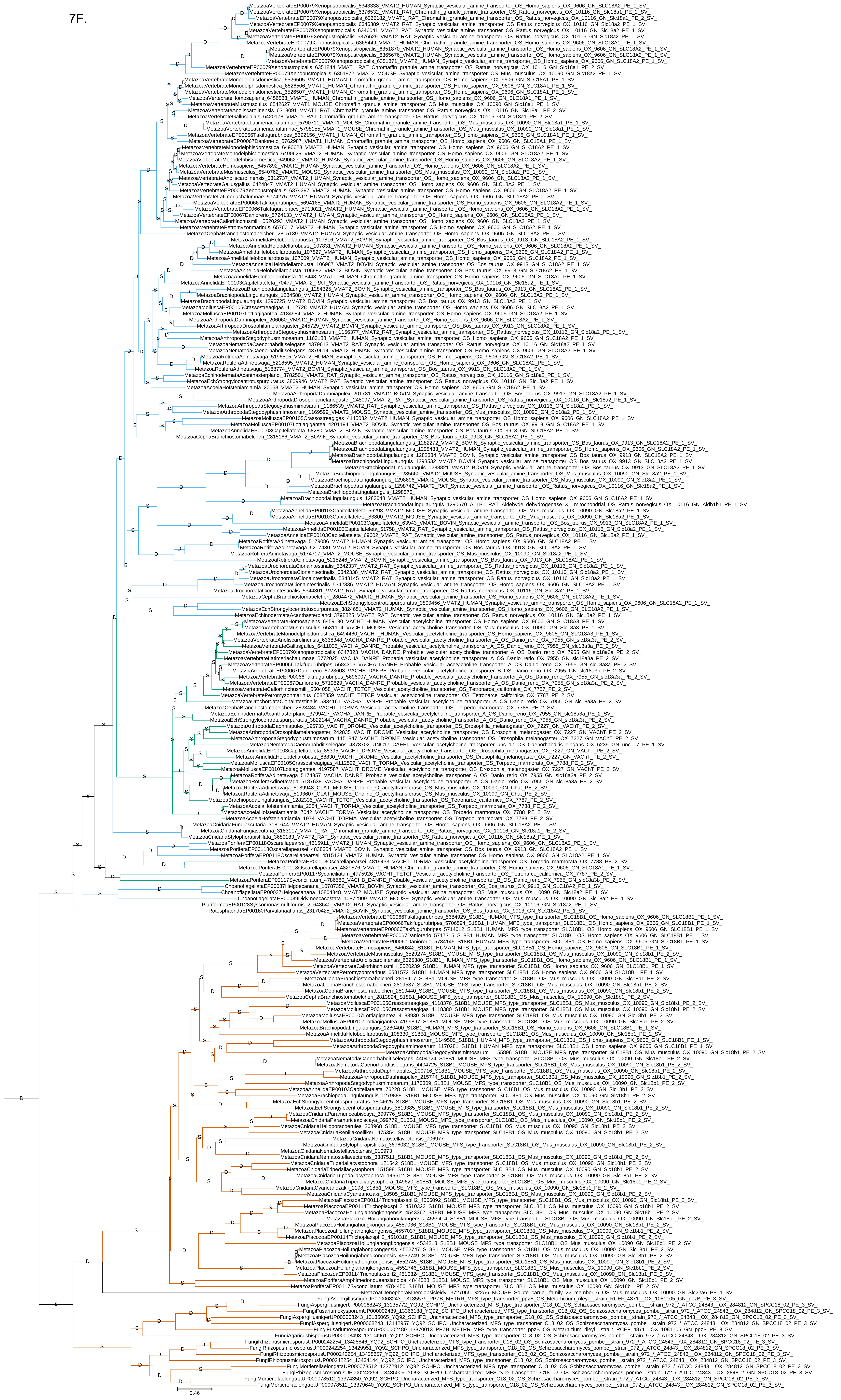

7G.

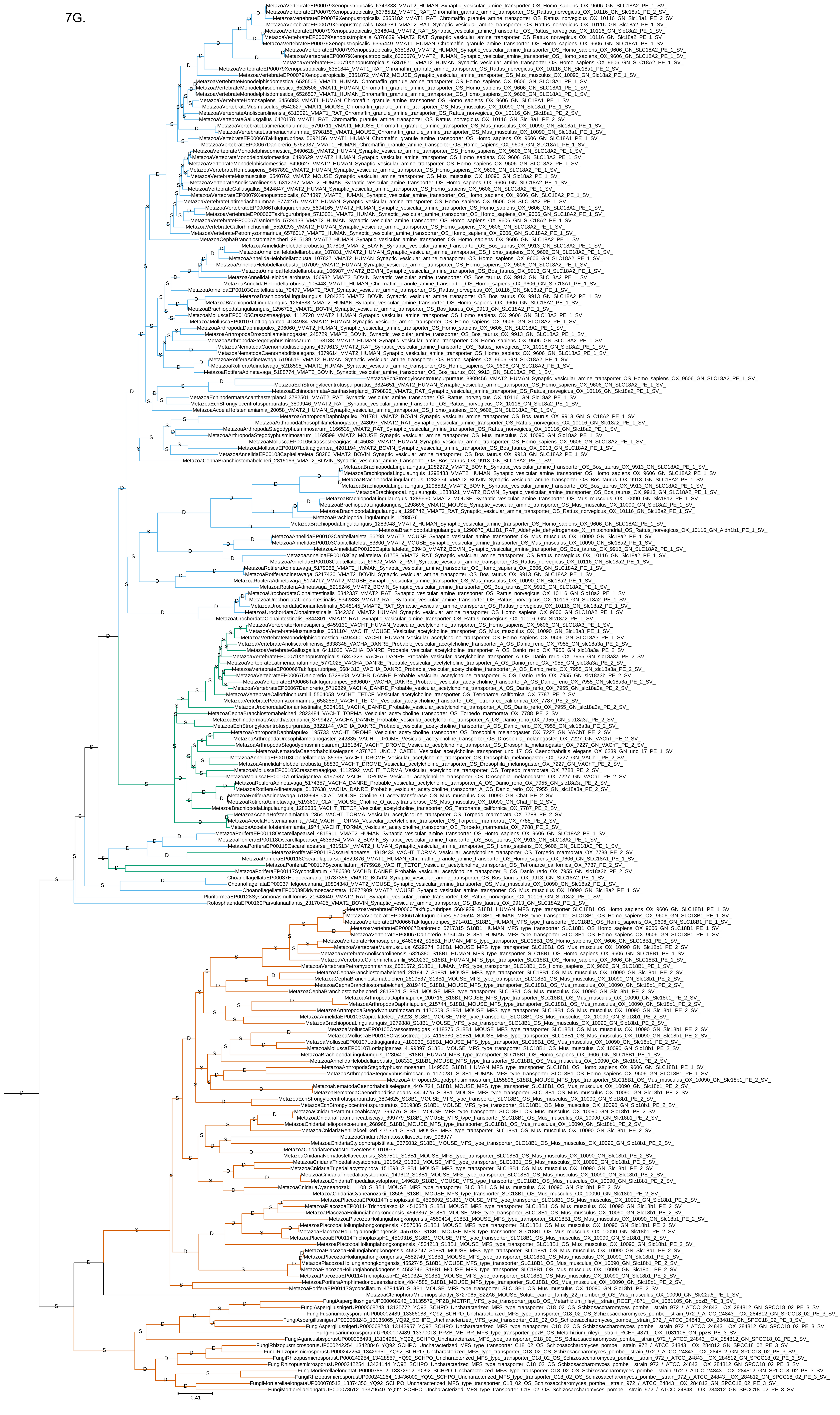

7H.

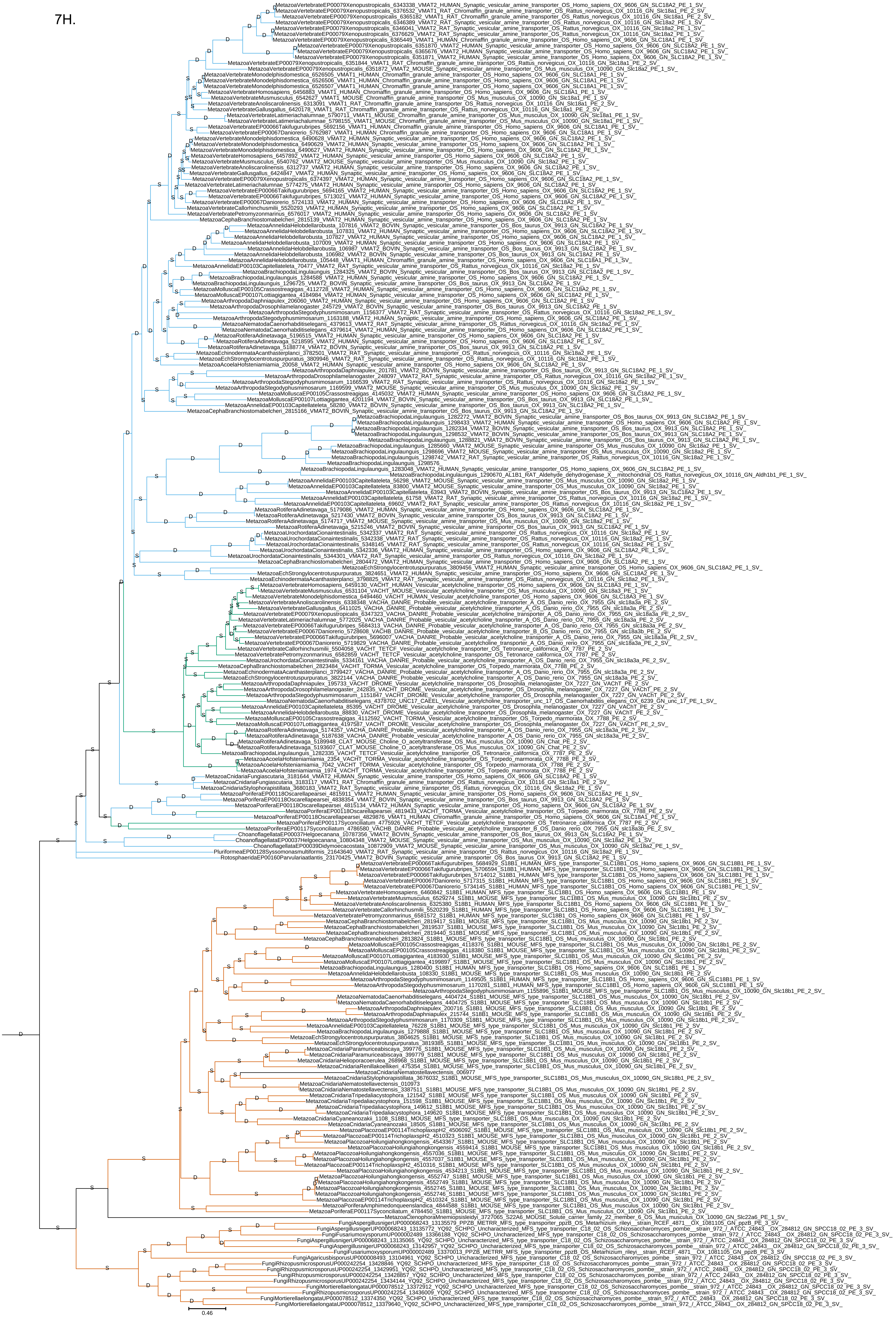

7I.

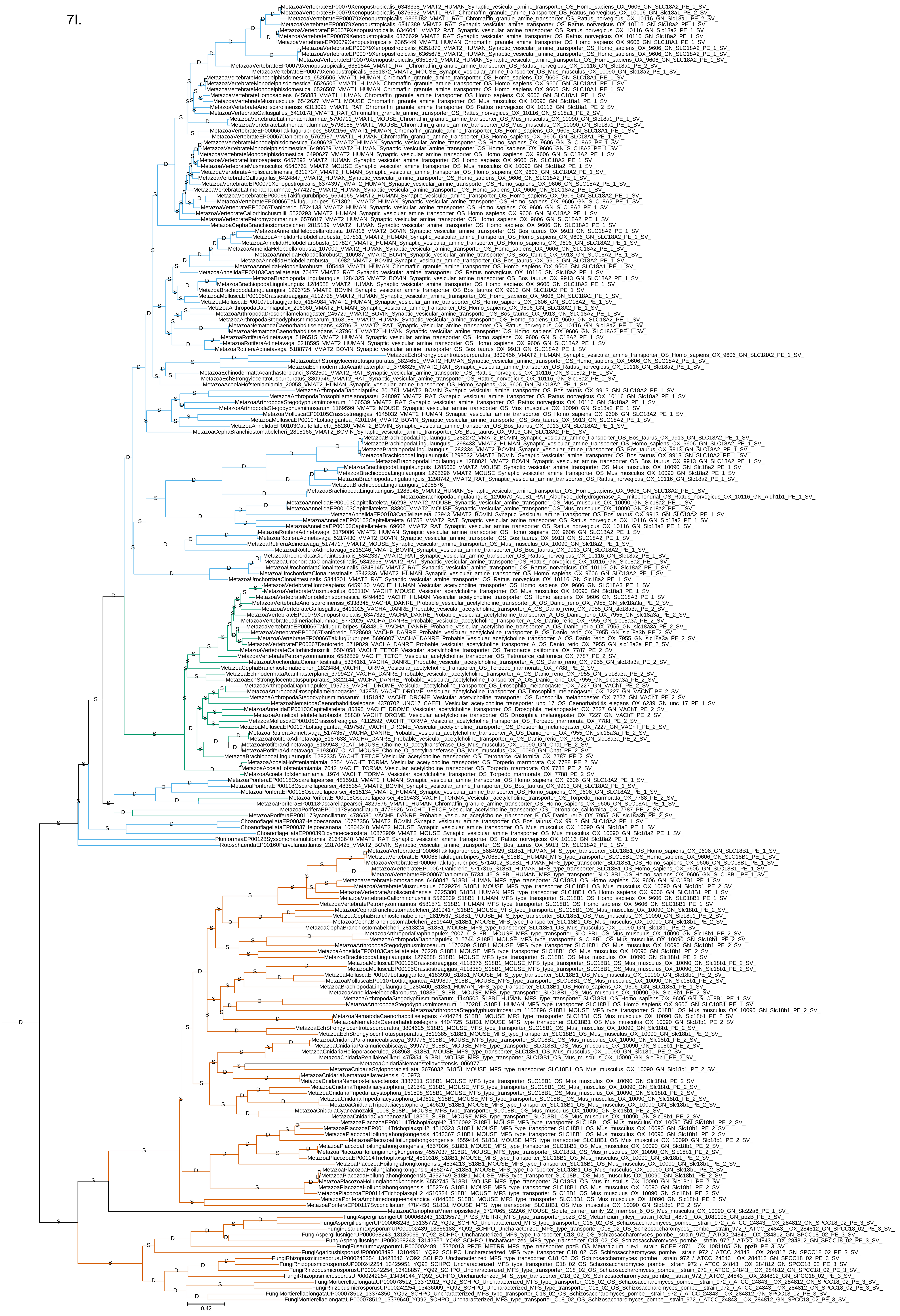

7J.

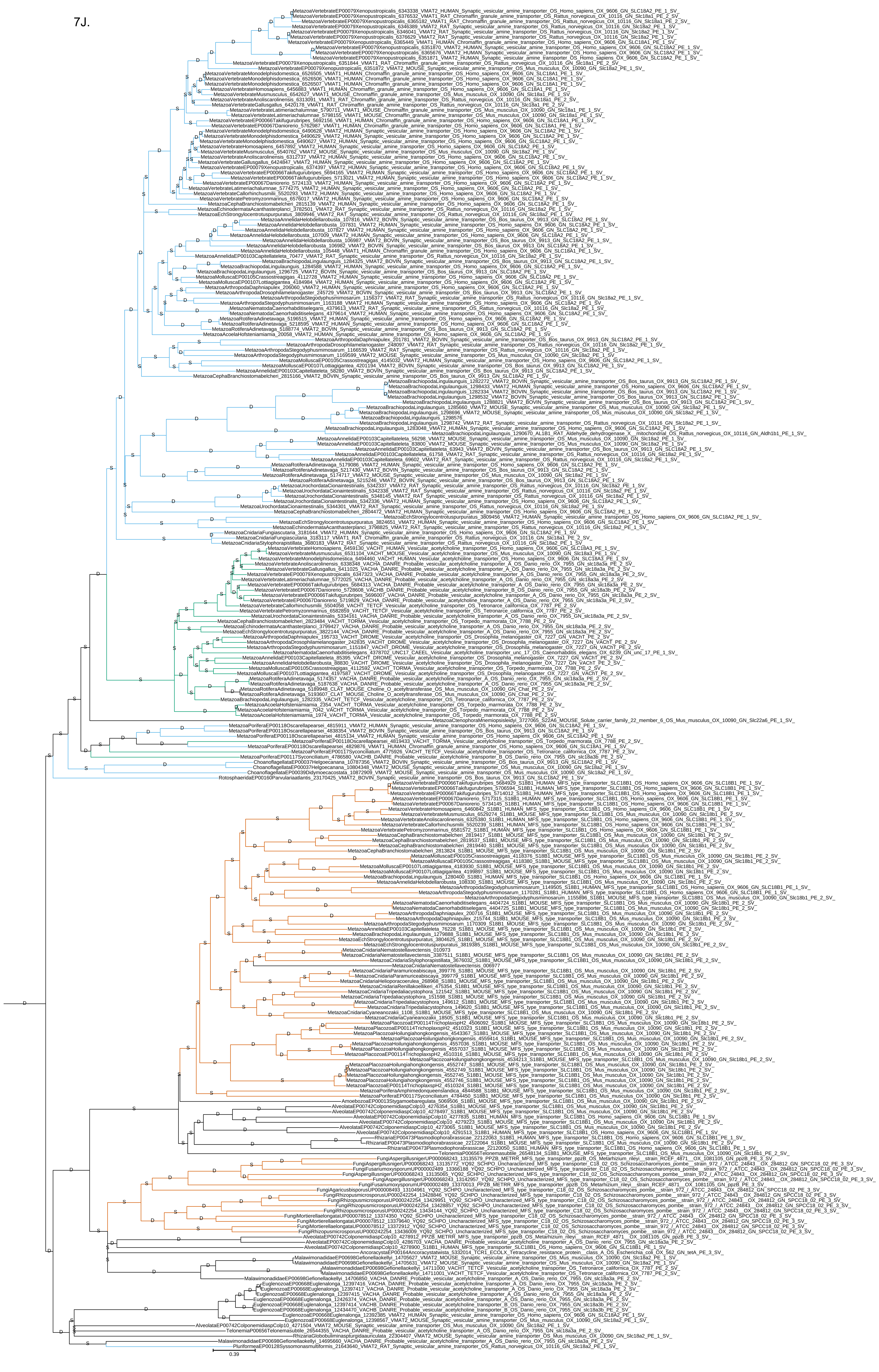

Supplementary Figure 8: Analysis of SLC6 sequences. A) TBE tree with TBE score supports. B) Maximum likelihood tree with ultrafast bootstrap proportion supports. C) Maximum likelihood tree including all eukaryotic sequences with ultrafast bootstrap proportion supports. D) Reconciliation using sponge-first hypothesis. E) Reconciliation using ctenophore-first hypothesis. F) Reconciliation of the UFB tree including all eukaryotic sequences. Nodes in reconciled trees indicated as speciation events (S) or duplication events (D). All trees shown as rooted, non-reconciled trees rooted arbitrarily for visualisation. SLC = solute ligand carrier, TBE = transfer bootstrap expectation, UFB = ultrafast bootstrap. Branches colour coded by sequence annotation or clade; dark blue = clade annotated as *Drosophila melanogaster* ine transporter; green = invertebrate dopamine transporter; purple = cnidarian specific clade; light orange = solute ligand carrier 6a2/6a3; light blue = other solute ligand carrier 6 sequences; dark orange = serotonin transporter; and black = others and non-opithokont sequences. Source Data are available at [https://figshare.le.ac.uk/articles/dataset/Monoamine_Neuromodulation_is_a_Bilaterian_Innovation_Results/20391477]; in Results/GeneTrees labelled OG_SLC6_TBE.tbe.tree and OG_SLC6_UFB.treefile; in Results/GeneTrees/Eukaryotes_GeneTrees labelled OG_SLC6_EUKIQ.treefile; in Results/Reconciliation/SpongeFirst labelled OG_SLC6; in Results/Reconciliation/CtenophoreFirst labelled OG_SLC6; and in Results/Reconciliation/Eukaryote labelled OG_SLC6.

8A.

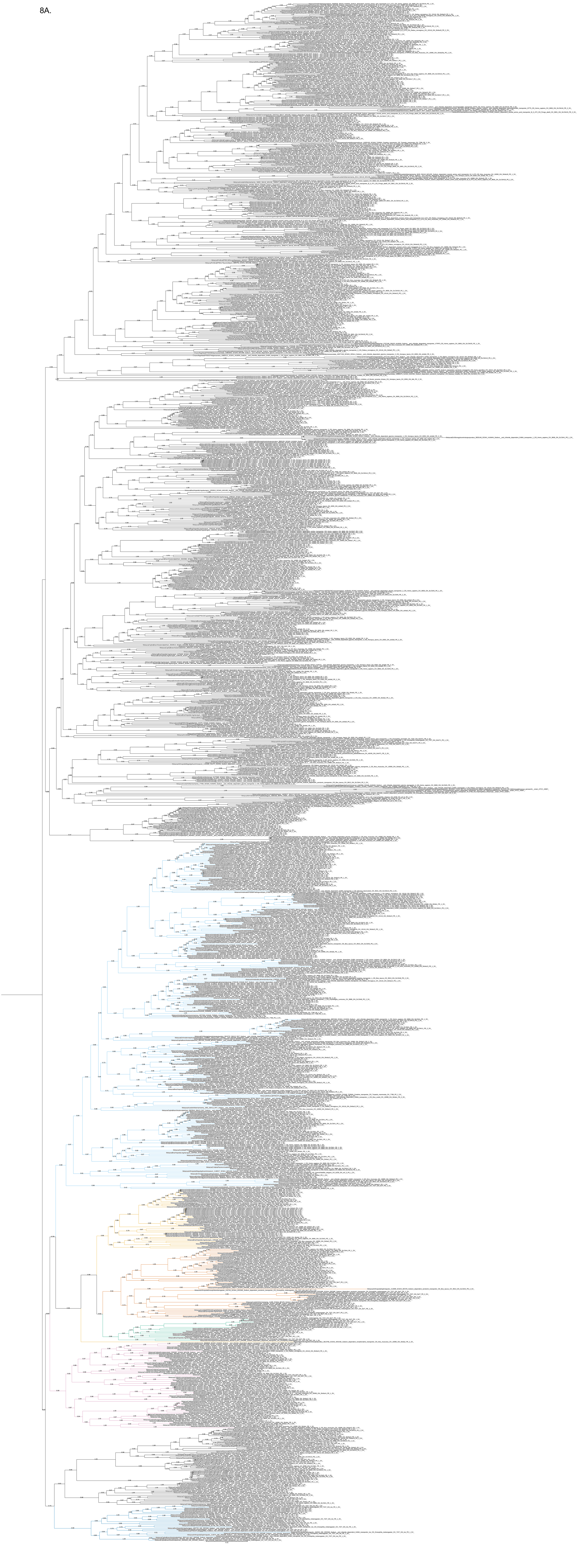

8B.

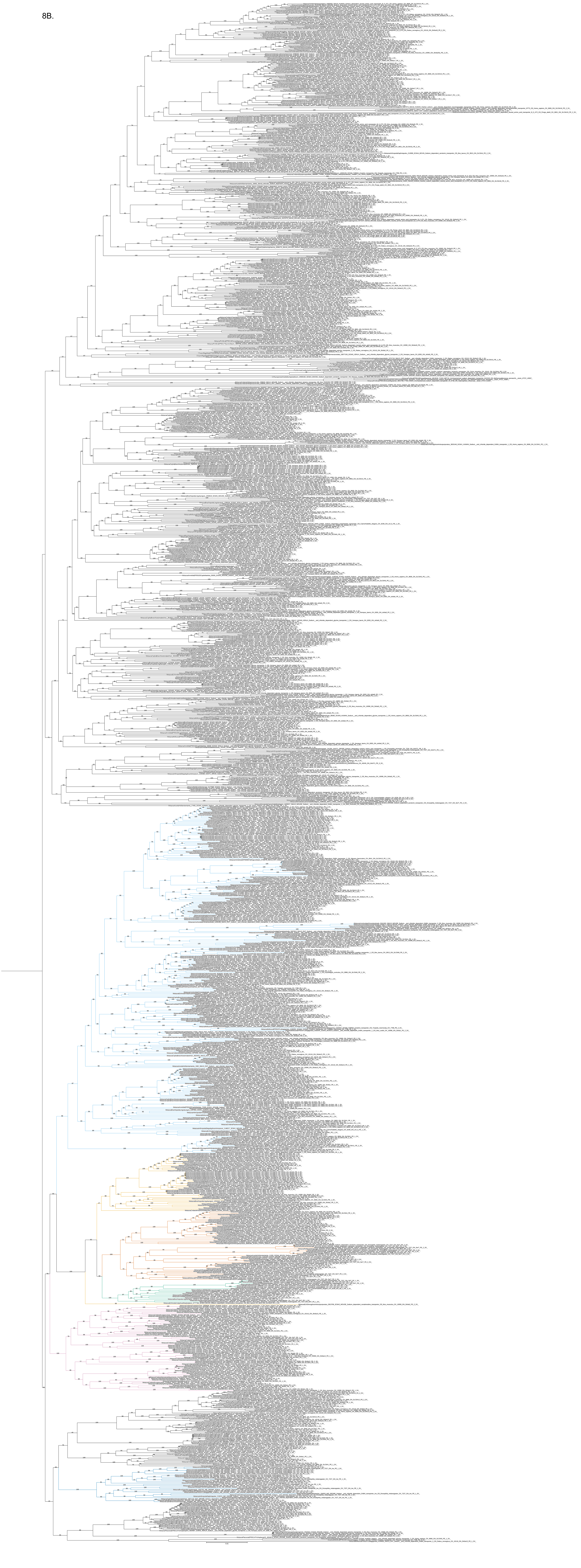

8C.

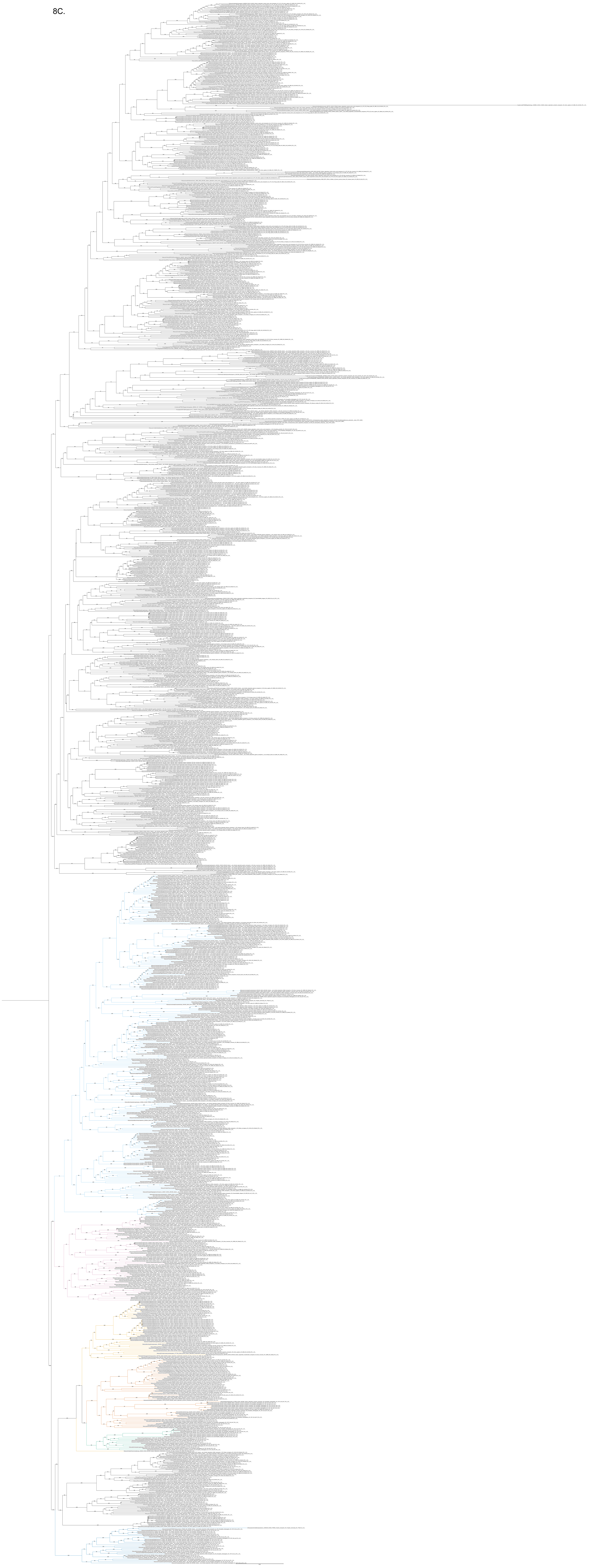

8D.

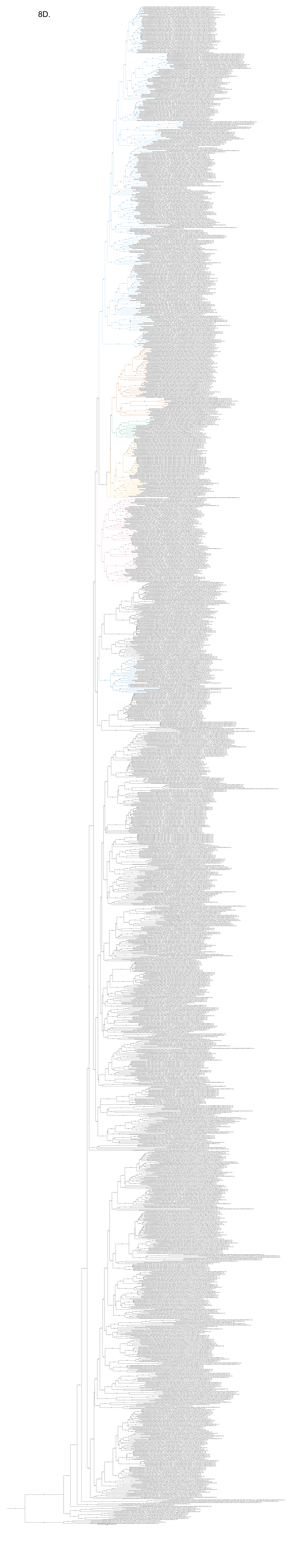

8E.

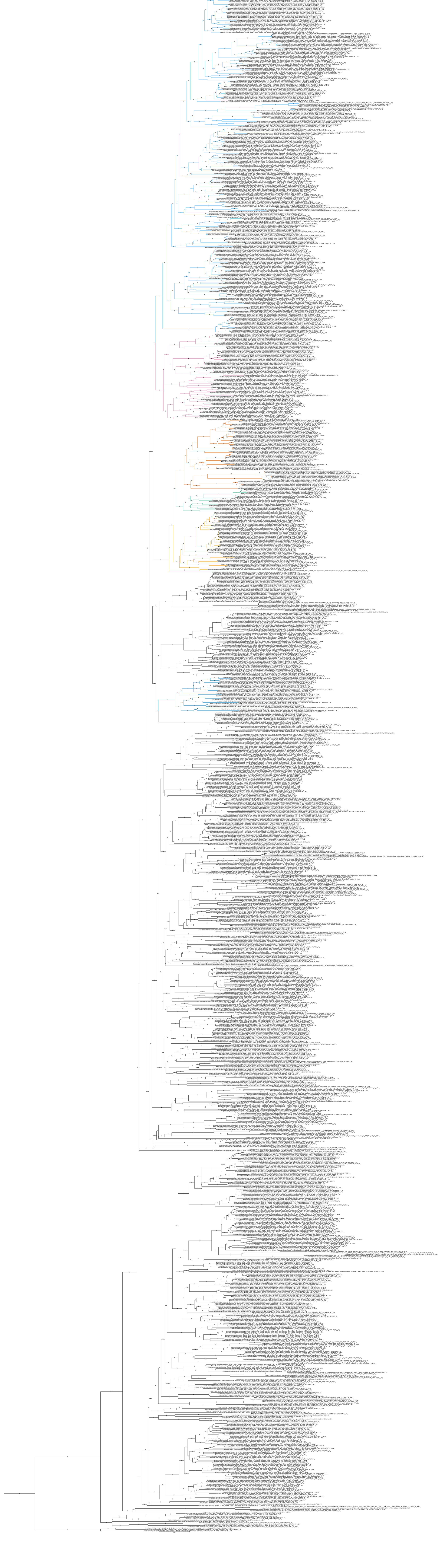

8F.

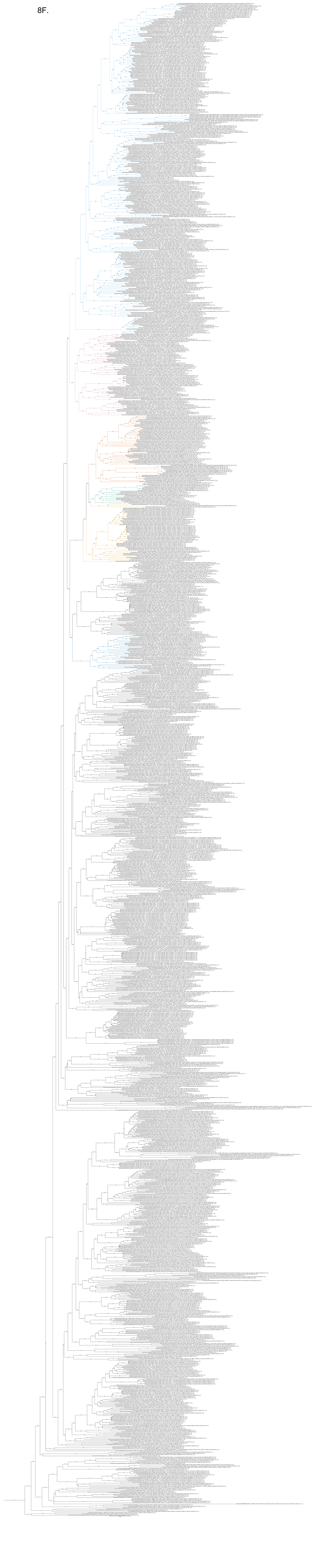

Supplementary Figure 9: Evolution of COMTs. Simplified illustration of the gene tree to species tree reconciliation. Silhouettes obtained from Phylopic.org. Silhouette images are by Andrew R. Gehrke (*Hofstenia miamia*); Daniel Jaron (*Mus musculus*); Mali'o Kodis, photograph by Ching (http://www.flickr.com/photos/36302473@N03/) (*Chrysaora fuscescens*); Mario Quevedo (Asteriidae); Oliver Voigt (*Trichoplax adhaerens*); Ramiro Morales-Hojas (*Drosophila americana*); Steven Haddock, Jellywatch.org (*Hormiphora californensis*); and Tess Linden (*Salpingoeca rosetta*). COMT = catechol-O-methyltransferase.

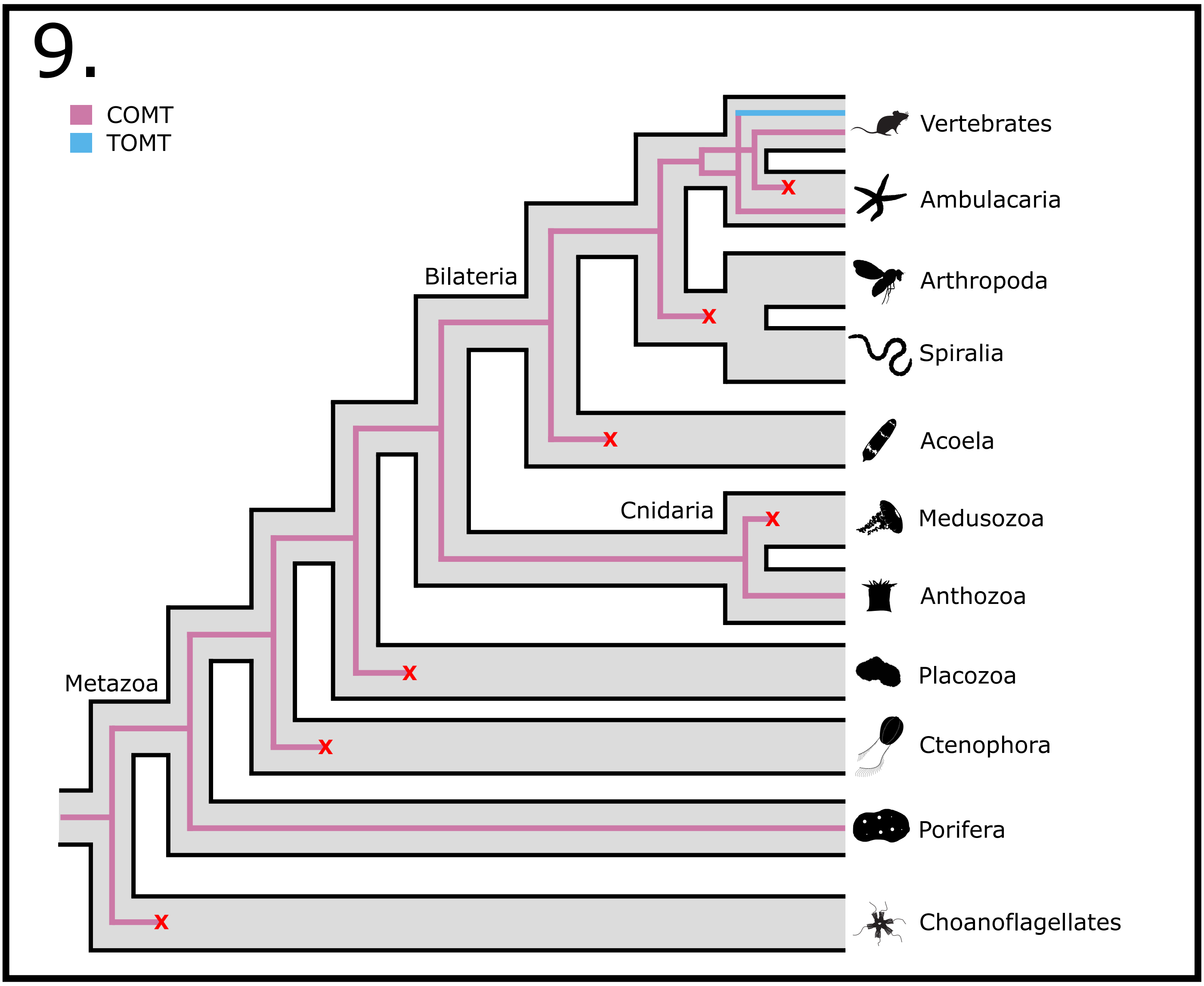

9.
COMT
TOMT
Vertebrates
Ambulacaria
Arthropoda
Spiralia
Acoela
Medusozoa
Anthozoa
Placozoa
Ctenophora
Porifera
Choanoflagellates
Bilateria
Cnidaria
Metazoa

Supplementary Figure 10: Analysis of COMTs. A) TBE tree with TBE score supports. B) Maximum likelihood tree with ultrafast bootstrap proportion supports. C) Maximum likelihood tree including all eukaryotic sequences with ultrafast bootstrap proportion supports. D) Reconciliation using sponge-first hypothesis. E) Reconciliation using ctenophore-first hypothesis. F) Reconciliation of the UFB tree including all eukaryotic sequences. Nodes in reconciled trees indicated as speciation events (S) or duplication events (D). All trees shown as rooted, non-reconciled trees rooted arbitrarily for visualisation. COMT = catechol-O-methyltransferase, TBE = transfer bootstrap expectation, UFB = ultrafast bootstrap. Branches colour coded by sequence annotation or clade; blue = transmembrane-O-methyltransferase; purple = catechol-O-methyltransferase; and black = others and non-opithokont sequences. Source Data are available at [https://figshare.le.ac.uk/articles/dataset/Monoamine_Neuromodulation_is_a_Bilaterian_Innovation_Results/20391477]; in Results/GeneTrees labelled OG_COMT_TBE.tbe.tree and OG_COMT_UFB.treefile; in Results/GeneTrees/Eukaryotes_GeneTrees labelled OG_COMT_EUKIQ.treefile; in Results/Reconciliation/SpongeFirst labelled OG_COMT; in Results/Reconciliation/CtenophoreFirst labelled OG_COMT; and in Results/Reconciliation/Eukaryote labelled OG_COMT.

10A.

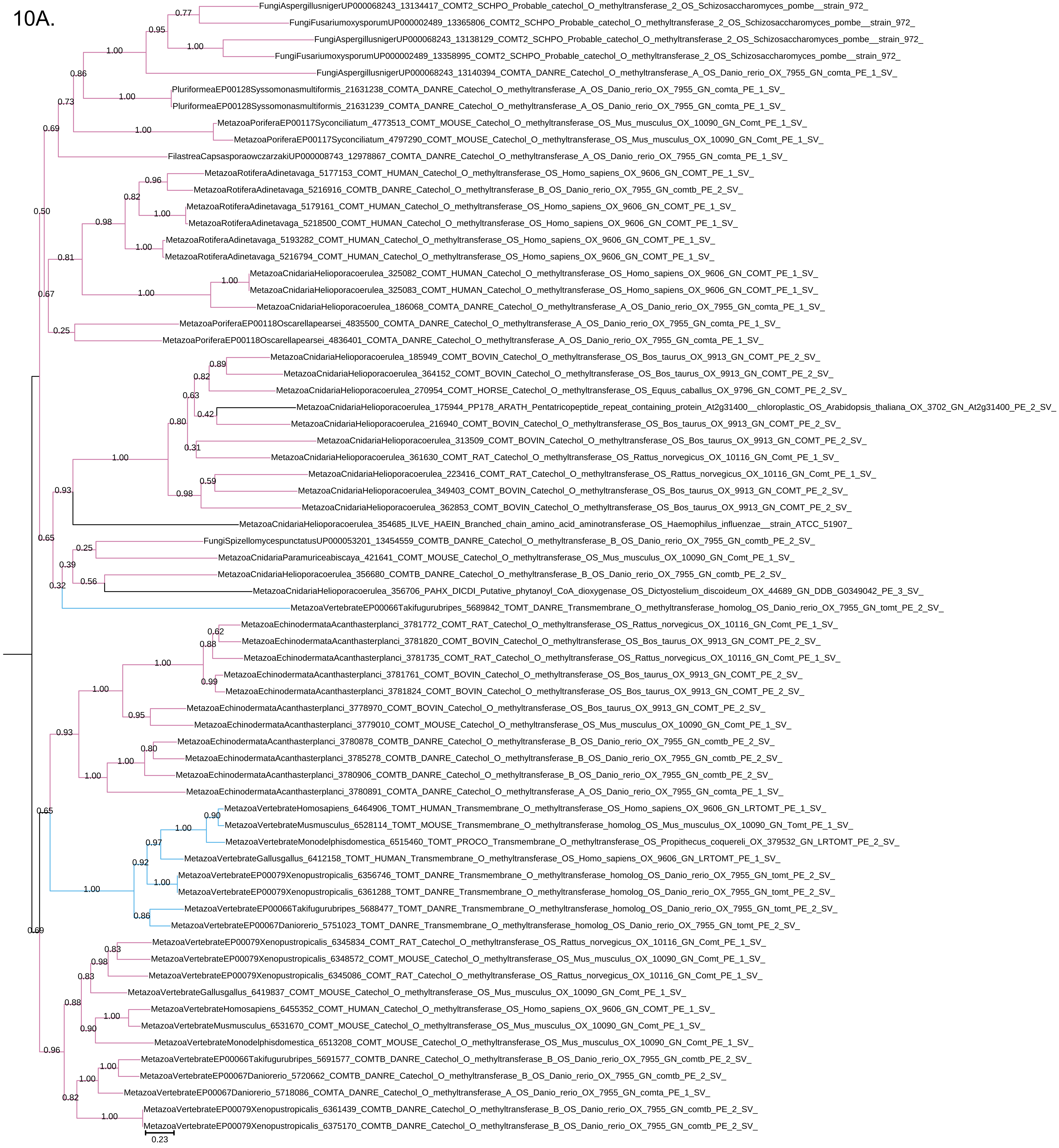
FungiAspergillusnigerUP000068243_13134417_COMT2_SCHPO_Probable_catechol_O_methyltransferase_2_OS_Schizosaccharomyces_pombe__strain_972_
FungiFusariumoxysporumUP000002489_13365806_COMT2_SCHPO_Probable_catechol_O_methyltransferase_2_OS_Schizosaccharomyces_pombe__strain_972_
FungiAspergillusnigerUP000068243_13138129_COMT2_SCHPO_Probable_catechol_O_methyltransferase_2_OS_Schizosaccharomyces_pombe__strain_972_
FungiFusariumoxysporumUP000002489_13358995_COMT2_SCHPO_Probable_catechol_O_methyltransferase_2_OS_Schizosaccharomyces_pombe__strain_972_
FungiAspergillusnigerUP000068243_13140394_COMTA_DANRE_Catechol_O_methyltransferase_A_OS_Danio_rerio_OX_7955_GN_comta_PE_1_SV_
PluriformeaEP00128Syssomonasmultiformis_21631238_COMTA_DANRE_Catechol_O_methyltransferase_A_OS_Danio_rerio_OX_7955_GN_comta_PE_1_SV_
PluriformeaEP00128Syssomonasmultiformis_21631239_COMTA_DANRE_Catechol_O_methyltransferase_A_OS_Danio_rerio_OX_7955_GN_comta_PE_1_SV_
MetazoaPoriferaEP00117Syconciliatum_4773513_COMT_MOUSE_Catechol_O_methyltransferase_OS_Mus_musculus_OX_10090_GN_Comt_PE_1_SV_
MetazoaPoriferaEP00117Syconciliatum_4797290_COMT_MOUSE_Catechol_O_methyltransferase_OS_Mus_musculus_OX_10090_GN_Comt_PE_1_SV_
FilastreaCapsasporaowczarzakiUP000008743_12978867_COMTA_DANRE_Catechol_O_methyltransferase_A_OS_Danio_rerio_OX_7955_GN_comta_PE_1_SV_
MetazoaRotiferaAdinetavaga_5177153_COMT_HUMAN_Catechol_O_methyltransferase_OS_Homo_sapiens_OX_9606_GN_COMT_PE_1_SV_
MetazoaRotiferaAdinetavaga_5216916_COMTB_DANRE_Catechol_O_methyltransferase_B_OS_Danio_rerio_OX_7955_GN_comtb_PE_2_SV_
MetazoaRotiferaAdinetavaga_5179161_COMT_HUMAN_Catechol_O_methyltransferase_OS_Homo_sapiens_OX_9606_GN_COMT_PE_1_SV_
MetazoaRotiferaAdinetavaga_5218500_COMT_HUMAN_Catechol_O_methyltransferase_OS_Homo_sapiens_OX_9606_GN_COMT_PE_1_SV_
MetazoaRotiferaAdinetavaga_5193282_COMT_HUMAN_Catechol_O_methyltransferase_OS_Homo_sapiens_OX_9606_GN_COMT_PE_1_SV_
MetazoaRotiferaAdinetavaga_5216794_COMT_HUMAN_Catechol_O_methyltransferase_OS_Homo_sapiens_OX_9606_GN_COMT_PE_1_SV_
MetazoaCnidariaHelioporacoerulea_325082_COMT_HUMAN_Catechol_O_methyltransferase_OS_Homo_sapiens_OX_9606_GN_COMT_PE_1_SV_
MetazoaCnidariaHelioporacoerulea_325083_COMT_HUMAN_Catechol_O_methyltransferase_OS_Homo_sapiens_OX_9606_GN_COMT_PE_1_SV_
MetazoaCnidariaHelioporacoerulea_186068_COMTA_DANRE_Catechol_O_methyltransferase_A_OS_Danio_rerio_OX_7955_GN_comta_PE_1_SV_
MetazoaPoriferaEP00118Oscarellapearsei_4835500_COMTA_DANRE_Catechol_O_methyltransferase_A_OS_Danio_rerio_OX_7955_GN_comta_PE_1_SV_
MetazoaPoriferaEP00118Oscarellapearsei_4836401_COMTA_DANRE_Catechol_O_methyltransferase_A_OS_Danio_rerio_OX_7955_GN_comta_PE_1_SV_
MetazoaCnidariaHelioporacoerulea_185949_COMT_BOVIN_Catechol_O_methyltransferase_OS_Bos_taurus_OX_9913_GN_COMT_PE_2_SV_
MetazoaCnidariaHelioporacoerulea_364152_COMT_BOVIN_Catechol_O_methyltransferase_OS_Bos_taurus_OX_9913_GN_COMT_PE_2_SV_
MetazoaCnidariaHelioporacoerulea_270954_COMT_HORSE_Catechol_O_methyltransferase_OS_Equus_caballus_OX_9796_GN_COMT_PE_2_SV_
MetazoaCnidariaHelioporacoerulea_175944_PP178_ARATH_Pentatricopeptide_repeat_containing_protein_At2g31400__chloroplastic_OS_Arabidopsis_thaliana_OX_3702_GN_At2g31400_PE_2_SV_
MetazoaCnidariaHelioporacoerulea_216940_COMT_BOVIN_Catechol_O_methyltransferase_OS_Bos_taurus_OX_9913_GN_COMT_PE_2_SV_
MetazoaCnidariaHelioporacoerulea_313509_COMT_BOVIN_Catechol_O_methyltransferase_OS_Bos_taurus_OX_9913_GN_COMT_PE_2_SV_
MetazoaCnidariaHelioporacoerulea_361630_COMT_RAT_Catechol_O_methyltransferase_OS_Rattus_norvegicus_OX_10116_GN_Comt_PE_1_SV_
MetazoaCnidariaHelioporacoerulea_223416_COMT_RAT_Catechol_O_methyltransferase_OS_Rattus_norvegicus_OX_10116_GN_Comt_PE_1_SV_
MetazoaCnidariaHelioporacoerulea_349403_COMT_BOVIN_Catechol_O_methyltransferase_OS_Bos_taurus_OX_9913_GN_COMT_PE_2_SV_
MetazoaCnidariaHelioporacoerulea_362853_COMT_BOVIN_Catechol_O_methyltransferase_OS_Bos_taurus_OX_9913_GN_COMT_PE_2_SV_
MetazoaCnidariaHelioporacoerulea_354685_ILVE_HAEIN_Branched_chain_amino_acid_aminotransferase_OS_Haemophilus_influenzae__strain_ATCC_51907_
FungiSpizellomycespunctatusUP000053201_13454559_COMTB_DANRE_Catechol_O_methyltransferase_B_OS_Danio_rerio_OX_7955_GN_comtb_PE_2_SV_
MetazoaCnidariaParamuriceabiscaya_421641_COMT_MOUSE_Catechol_O_methyltransferase_OS_Mus_musculus_OX_10090_GN_Comt_PE_1_SV_
MetazoaCnidariaHelioporacoerulea_356680_COMTB_DANRE_Catechol_O_methyltransferase_B_OS_Danio_rerio_OX_7955_GN_comtb_PE_2_SV_
MetazoaCnidariaHelioporacoerulea_356706_PAHX_DICDI_Putative_phytanoyl_CoA_dioxygenase_OS_Dictyostelium_discoideum_OX_44689_GN_DDB_G0349042_PE_3_SV_
MetazoaVertebrateEP00066Takifugurubripes_5689842_TOMT_DANRE_Transmembrane_O_methyltransferase_homolog_OS_Danio_rerio_OX_7955_GN_tomt_PE_2_SV_
MetazoaEchinodermataAcanthasterplanci_3781772_COMT_RAT_Catechol_O_methyltransferase_OS_Rattus_norvegicus_OX_10116_GN_Comt_PE_1_SV_
MetazoaEchinodermataAcanthasterplanci_3781820_COMT_BOVIN_Catechol_O_methyltransferase_OS_Bos_taurus_OX_9913_GN_COMT_PE_2_SV_
MetazoaEchinodermataAcanthasterplanci_3781735_COMT_RAT_Catechol_O_methyltransferase_OS_Rattus_norvegicus_OX_10116_GN_Comt_PE_1_SV_
MetazoaEchinodermataAcanthasterplanci_3781761_COMT_BOVIN_Catechol_O_methyltransferase_OS_Bos_taurus_OX_9913_GN_COMT_PE_2_SV_
MetazoaEchinodermataAcanthasterplanci_3781824_COMT_BOVIN_Catechol_O_methyltransferase_OS_Bos_taurus_OX_9913_GN_COMT_PE_2_SV_
MetazoaEchinodermataAcanthasterplanci_3778970_COMT_BOVIN_Catechol_O_methyltransferase_OS_Bos_taurus_OX_9913_GN_COMT_PE_2_SV_
MetazoaEchinodermataAcanthasterplanci_3779010_COMT_MOUSE_Catechol_O_methyltransferase_OS_Mus_musculus_OX_10090_GN_Comt_PE_1_SV_
MetazoaEchinodermataAcanthasterplanci_3780878_COMTB_DANRE_Catechol_O_methyltransferase_B_OS_Danio_rerio_OX_7955_GN_comtb_PE_2_SV_
MetazoaEchinodermataAcanthasterplanci_3785278_COMTB_DANRE_Catechol_O_methyltransferase_B_OS_Danio_rerio_OX_7955_GN_comtb_PE_2_SV_
MetazoaEchinodermataAcanthasterplanci_3780906_COMTB_DANRE_Catechol_O_methyltransferase_B_OS_Danio_rerio_OX_7955_GN_comtb_PE_2_SV_
MetazoaEchinodermataAcanthasterplanci_3780891_COMTA_DANRE_Catechol_O_methyltransferase_A_OS_Danio_rerio_OX_7955_GN_comta_PE_1_SV_
MetazoaVertebrateHomosapiens_6464906_TOMT_HUMAN_Transmembrane_O_methyltransferase_OS_Homo_sapiens_OX_9606_GN_LRTOMT_PE_1_SV_
MetazoaVertebrateMusmusculus_6528114_TOMT_MOUSE_Transmembrane_O_methyltransferase_homolog_OS_Mus_musculus_OX_10090_GN_Tomt_PE_1_SV_
MetazoaVertebrateMonodelphisdomestica_6515460_TOMT_PROCO_Transmembrane_O_methyltransferase_OS_Propithecus_coquereli_OX_379532_GN_LRTOMT_PE_2_SV_
MetazoaVertebrateGallusgallus_6412158_TOMT_HUMAN_Transmembrane_O_methyltransferase_OS_Homo_sapiens_OX_9606_GN_LRTOMT_PE_1_SV_
MetazoaVertebrateEP00079Xenopustropicalis_6356746_TOMT_DANRE_Transmembrane_O_methyltransferase_homolog_OS_Danio_rerio_OX_7955_GN_tomt_PE_2_SV_
MetazoaVertebrateEP00079Xenopustropicalis_6361288_TOMT_DANRE_Transmembrane_O_methyltransferase_homolog_OS_Danio_rerio_OX_7955_GN_tomt_PE_2_SV_
MetazoaVertebrateEP00066Takifugurubripes_5688477_TOMT_DANRE_Transmembrane_O_methyltransferase_homolog_OS_Danio_rerio_OX_7955_GN_tomt_PE_2_SV_
MetazoaVertebrateEP00067Daniorerio_5751023_TOMT_DANRE_Transmembrane_O_methyltransferase_homolog_OS_Danio_rerio_OX_7955_GN_tomt_PE_2_SV_
MetazoaVertebrateEP00079Xenopustropicalis_6345834_COMT_RAT_Catechol_O_methyltransferase_OS_Rattus_norvegicus_OX_10116_GN_Comt_PE_1_SV_
MetazoaVertebrateEP00079Xenopustropicalis_6348572_COMT_MOUSE_Catechol_O_methyltransferase_OS_Mus_musculus_OX_10090_GN_Comt_PE_1_SV_
MetazoaVertebrateEP00079Xenopustropicalis_6345086_COMT_RAT_Catechol_O_methyltransferase_OS_Rattus_norvegicus_OX_10116_GN_Comt_PE_1_SV_
MetazoaVertebrateGallusgallus_6419837_COMT_MOUSE_Catechol_O_methyltransferase_OS_Mus_musculus_OX_10090_GN_Comt_PE_1_SV_
MetazoaVertebrateHomosapiens_6455352_COMT_HUMAN_Catechol_O_methyltransferase_OS_Homo_sapiens_OX_9606_GN_COMT_PE_1_SV_
MetazoaVertebrateMusmusculus_6531670_COMT_MOUSE_Catechol_O_methyltransferase_OS_Mus_musculus_OX_10090_GN_Comt_PE_1_SV_
MetazoaVertebrateMonodelphisdomestica_6513208_COMT_MOUSE_Catechol_O_methyltransferase_OS_Mus_musculus_OX_10090_GN_Comt_PE_1_SV_
MetazoaVertebrateEP00066Takifugurubripes_5691577_COMTB_DANRE_Catechol_O_methyltransferase_B_OS_Danio_rerio_OX_7955_GN_comtb_PE_2_SV_
MetazoaVertebrateEP00067Daniorerio_5720662_COMTB_DANRE_Catechol_O_methyltransferase_B_OS_Danio_rerio_OX_7955_GN_comtb_PE_2_SV_
MetazoaVertebrateEP00067Daniorerio_5718086_COMTA_DANRE_Catechol_O_methyltransferase_A_OS_Danio_rerio_OX_7955_GN_comta_PE_1_SV_
MetazoaVertebrateEP00079Xenopustropicalis_6361439_COMTB_DANRE_Catechol_O_methyltransferase_B_OS_Danio_rerio_OX_7955_GN_comtb_PE_2_SV_
MetazoaVertebrateEP00079Xenopustropicalis_6375170_COMTB_DANRE_Catechol_O_methyltransferase_B_OS_Danio_rerio_OX_7955_GN_comtb_PE_2_SV_
0.23

10B.

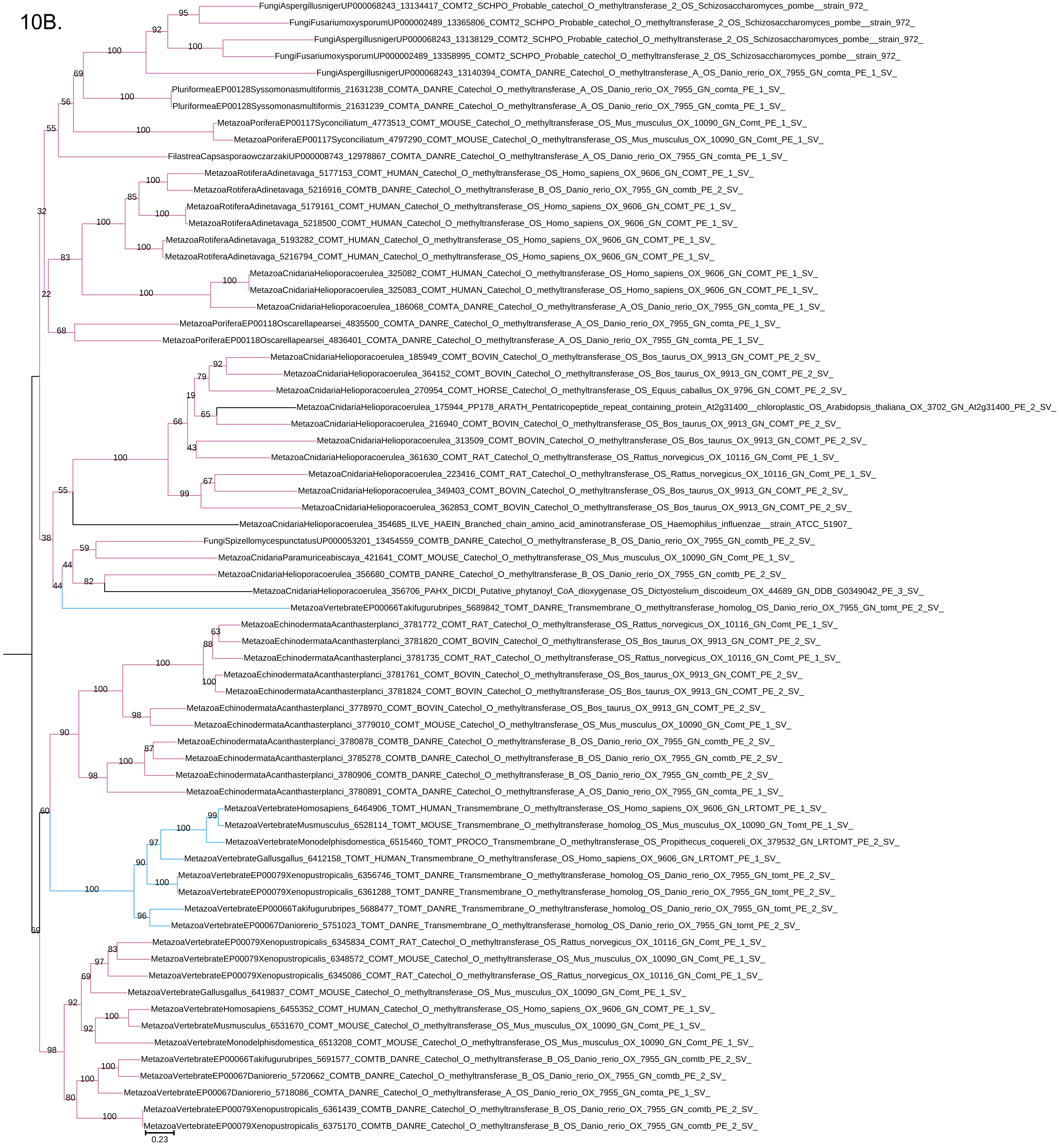
FungiAspergillusnigerUP000068243_13134417_COMT2_SCHPO_Probable_catechol_O_methyltransferase_2_OS_Schizosaccharomyces_pombe__strain_972_
FungiFusariumoxysporumUP000002489_13365806_COMT2_SCHPO_Probable_catechol_O_methyltransferase_2_OS_Schizosaccharomyces_pombe__strain_972_
FungiAspergillusnigerUP000068243_13138129_COMT2_SCHPO_Probable_catechol_O_methyltransferase_2_OS_Schizosaccharomyces_pombe__strain_972_
FungiFusariumoxysporumUP000002489_13358995_COMT2_SCHPO_Probable_catechol_O_methyltransferase_2_OS_Schizosaccharomyces_pombe__strain_972_
FungiAspergillusnigerUP000068243_13140394_COMTA_DANRE_Catechol_O_methyltransferase_A_OS_Danio_rerio_OX_7955_GN_comta_PE_1_SV_
PluriformeaEP00128Syssomonasmultiformis_21631238_COMTA_DANRE_Catechol_O_methyltransferase_A_OS_Danio_rerio_OX_7955_GN_comta_PE_1_SV_
PluriformeaEP00128Syssomonasmultiformis_21631239_COMTA_DANRE_Catechol_O_methyltransferase_A_OS_Danio_rerio_OX_7955_GN_comta_PE_1_SV_
MetazoaPoriferaEP00117Syconciliatum_4773513_COMT_MOUSE_Catechol_O_methyltransferase_OS_Mus_musculus_OX_10090_GN_Comt_PE_1_SV_
MetazoaPoriferaEP00117Syconciliatum_4797290_COMT_MOUSE_Catechol_O_methyltransferase_OS_Mus_musculus_OX_10090_GN_Comt_PE_1_SV_
FilastreaCapsasporaowczarzakiUP000008743_12978867_COMTA_DANRE_Catechol_O_methyltransferase_A_OS_Danio_rerio_OX_7955_GN_comta_PE_1_SV_
MetazoaRotiferaAdinetavaga_5177153_COMT_HUMAN_Catechol_O_methyltransferase_OS_Homo_sapiens_OX_9606_GN_COMT_PE_1_SV_
MetazoaRotiferaAdinetavaga_5216916_COMTB_DANRE_Catechol_O_methyltransferase_B_OS_Danio_rerio_OX_7955_GN_comtb_PE_2_SV_
MetazoaRotiferaAdinetavaga_5179161_COMT_HUMAN_Catechol_O_methyltransferase_OS_Homo_sapiens_OX_9606_GN_COMT_PE_1_SV_
MetazoaRotiferaAdinetavaga_5218500_COMT_HUMAN_Catechol_O_methyltransferase_OS_Homo_sapiens_OX_9606_GN_COMT_PE_1_SV_
MetazoaRotiferaAdinetavaga_5193282_COMT_HUMAN_Catechol_O_methyltransferase_OS_Homo_sapiens_OX_9606_GN_COMT_PE_1_SV_
MetazoaRotiferaAdinetavaga_5216794_COMT_HUMAN_Catechol_O_methyltransferase_OS_Homo_sapiens_OX_9606_GN_COMT_PE_1_SV_
MetazoaCnidariaHelioporacoerulea_325082_COMT_HUMAN_Catechol_O_methyltransferase_OS_Homo_sapiens_OX_9606_GN_COMT_PE_1_SV_
MetazoaCnidariaHelioporacoerulea_325083_COMT_HUMAN_Catechol_O_methyltransferase_OS_Homo_sapiens_OX_9606_GN_COMT_PE_1_SV_
MetazoaCnidariaHelioporacoerulea_186068_COMTA_DANRE_Catechol_O_methyltransferase_A_OS_Danio_rerio_OX_7955_GN_comta_PE_1_SV_
MetazoaPoriferaEP00118Oscarellapearsei_4835500_COMTA_DANRE_Catechol_O_methyltransferase_A_OS_Danio_rerio_OX_7955_GN_comta_PE_1_SV_
MetazoaPoriferaEP00118Oscarellapearsei_4836401_COMTA_DANRE_Catechol_O_methyltransferase_A_OS_Danio_rerio_OX_7955_GN_comta_PE_1_SV_
MetazoaCnidariaHelioporacoerulea_185949_COMT_BOVIN_Catechol_O_methyltransferase_OS_Bos_taurus_OX_9913_GN_COMT_PE_2_SV_
MetazoaCnidariaHelioporacoerulea_364152_COMT_BOVIN_Catechol_O_methyltransferase_OS_Bos_taurus_OX_9913_GN_COMT_PE_2_SV_
MetazoaCnidariaHelioporacoerulea_270954_COMT_HORSE_Catechol_O_methyltransferase_OS_Equus_caballus_OX_9796_GN_COMT_PE_2_SV_
MetazoaCnidariaHelioporacoerulea_175944_PP178_ARATH_Pentatricopeptide_repeat_containing_protein_At2g31400__chloroplastic_OS_Arabidopsis_thaliana_OX_3702_GN_At2g31400_PE_2_SV_
MetazoaCnidariaHelioporacoerulea_216940_COMT_BOVIN_Catechol_O_methyltransferase_OS_Bos_taurus_OX_9913_GN_COMT_PE_2_SV_
MetazoaCnidariaHelioporacoerulea_313509_COMT_BOVIN_Catechol_O_methyltransferase_OS_Bos_taurus_OX_9913_GN_COMT_PE_2_SV_
MetazoaCnidariaHelioporacoerulea_361630_COMT_RAT_Catechol_O_methyltransferase_OS_Rattus_norvegicus_OX_10116_GN_Comt_PE_1_SV_
MetazoaCnidariaHelioporacoerulea_223416_COMT_RAT_Catechol_O_methyltransferase_OS_Rattus_norvegicus_OX_10116_GN_Comt_PE_1_SV_
MetazoaCnidariaHelioporacoerulea_349403_COMT_BOVIN_Catechol_O_methyltransferase_OS_Bos_taurus_OX_9913_GN_COMT_PE_2_SV_
MetazoaCnidariaHelioporacoerulea_362853_COMT_BOVIN_Catechol_O_methyltransferase_OS_Bos_taurus_OX_9913_GN_COMT_PE_2_SV_
MetazoaCnidariaHelioporacoerulea_354685_ILVE_HAEIN_Branched_chain_amino_acid_aminotransferase_OS_Haemophilus_influenzae__strain_ATCC_51907_
FungiSpizellomycespunctatusUP000053201_13454559_COMTB_DANRE_Catechol_O_methyltransferase_B_OS_Danio_rerio_OX_7955_GN_comtb_PE_2_SV_
MetazoaCnidariaParamuriceabiscaya_421641_COMT_MOUSE_Catechol_O_methyltransferase_OS_Mus_musculus_OX_10090_GN_Comt_PE_1_SV_
MetazoaCnidariaHelioporacoerulea_356680_COMTB_DANRE_Catechol_O_methyltransferase_B_OS_Danio_rerio_OX_7955_GN_comtb_PE_2_SV_
MetazoaCnidariaHelioporacoerulea_356706_PAHX_DICDI_Putative_phytanoyl_CoA_dioxygenase_OS_Dictyostelium_discoideum_OX_44689_GN_DDB_G0349042_PE_3_SV_
MetazoaVertebrateEP00066Takifugurubripes_5689842_TOMT_DANRE_Transmembrane_O_methyltransferase_homolog_OS_Danio_rerio_OX_7955_GN_tomt_PE_2_SV_
MetazoaEchinodermataAcanthasterplanci_3781772_COMT_RAT_Catechol_O_methyltransferase_OS_Rattus_norvegicus_OX_10116_GN_Comt_PE_1_SV_
MetazoaEchinodermataAcanthasterplanci_3781820_COMT_BOVIN_Catechol_O_methyltransferase_OS_Bos_taurus_OX_9913_GN_COMT_PE_2_SV_
MetazoaEchinodermataAcanthasterplanci_3781735_COMT_RAT_Catechol_O_methyltransferase_OS_Rattus_norvegicus_OX_10116_GN_Comt_PE_1_SV_
MetazoaEchinodermataAcanthasterplanci_3781761_COMT_BOVIN_Catechol_O_methyltransferase_OS_Bos_taurus_OX_9913_GN_COMT_PE_2_SV_
MetazoaEchinodermataAcanthasterplanci_3781824_COMT_BOVIN_Catechol_O_methyltransferase_OS_Bos_taurus_OX_9913_GN_COMT_PE_2_SV_
MetazoaEchinodermataAcanthasterplanci_3778970_COMT_BOVIN_Catechol_O_methyltransferase_OS_Bos_taurus_OX_9913_GN_COMT_PE_2_SV_
MetazoaEchinodermataAcanthasterplanci_3779010_COMT_MOUSE_Catechol_O_methyltransferase_OS_Mus_musculus_OX_10090_GN_Comt_PE_1_SV_
MetazoaEchinodermataAcanthasterplanci_3780878_COMTB_DANRE_Catechol_O_methyltransferase_B_OS_Danio_rerio_OX_7955_GN_comtb_PE_2_SV_
MetazoaEchinodermataAcanthasterplanci_3785278_COMTB_DANRE_Catechol_O_methyltransferase_B_OS_Danio_rerio_OX_7955_GN_comtb_PE_2_SV_
MetazoaEchinodermataAcanthasterplanci_3780906_COMTB_DANRE_Catechol_O_methyltransferase_B_OS_Danio_rerio_OX_7955_GN_comtb_PE_2_SV_
MetazoaEchinodermataAcanthasterplanci_3780891_COMTA_DANRE_Catechol_O_methyltransferase_A_OS_Danio_rerio_OX_7955_GN_comta_PE_1_SV_
MetazoaVertebrateHomosapiens_6464906_TOMT_HUMAN_Transmembrane_O_methyltransferase_OS_Homo_sapiens_OX_9606_GN_LRTOMT_PE_1_SV_
MetazoaVertebrateMusmusculus_6528114_TOMT_MOUSE_Transmembrane_O_methyltransferase_homolog_OS_Mus_musculus_OX_10090_GN_Tomt_PE_1_SV_
MetazoaVertebrateMonodelphisdomestica_6515460_TOMT_PROCO_Transmembrane_O_methyltransferase_OS_Propithecus_coquereli_OX_379532_GN_LRTOMT_PE_2_SV_
MetazoaVertebrateGallusgallus_6412158_TOMT_HUMAN_Transmembrane_O_methyltransferase_OS_Homo_sapiens_OX_9606_GN_LRTOMT_PE_1_SV_
MetazoaVertebrateEP00079Xenopustropicalis_6356746_TOMT_DANRE_Transmembrane_O_methyltransferase_homolog_OS_Danio_rerio_OX_7955_GN_tomt_PE_2_SV_
MetazoaVertebrateEP00079Xenopustropicalis_6361288_TOMT_DANRE_Transmembrane_O_methyltransferase_homolog_OS_Danio_rerio_OX_7955_GN_tomt_PE_2_SV_
MetazoaVertebrateEP00066Takifugurubripes_5688477_TOMT_DANRE_Transmembrane_O_methyltransferase_homolog_OS_Danio_rerio_OX_7955_GN_tomt_PE_2_SV_
MetazoaVertebrateEP00067Daniorerio_5751023_TOMT_DANRE_Transmembrane_O_methyltransferase_homolog_OS_Danio_rerio_OX_7955_GN_tomt_PE_2_SV_
MetazoaVertebrateEP00079Xenopustropicalis_6345834_COMT_RAT_Catechol_O_methyltransferase_OS_Rattus_norvegicus_OX_10116_GN_Comt_PE_1_SV_
MetazoaVertebrateEP00079Xenopustropicalis_6348572_COMT_MOUSE_Catechol_O_methyltransferase_OS_Mus_musculus_OX_10090_GN_Comt_PE_1_SV_
MetazoaVertebrateEP00079Xenopustropicalis_6345086_COMT_RAT_Catechol_O_methyltransferase_OS_Rattus_norvegicus_OX_10116_GN_Comt_PE_1_SV_
MetazoaVertebrateGallusgallus_6419837_COMT_MOUSE_Catechol_O_methyltransferase_OS_Mus_musculus_OX_10090_GN_Comt_PE_1_SV_
MetazoaVertebrateHomosapiens_6455352_COMT_HUMAN_Catechol_O_methyltransferase_OS_Homo_sapiens_OX_9606_GN_COMT_PE_1_SV_
MetazoaVertebrateMusmusculus_6531670_COMT_MOUSE_Catechol_O_methyltransferase_OS_Mus_musculus_OX_10090_GN_Comt_PE_1_SV_
MetazoaVertebrateMonodelphisdomestica_6513208_COMT_MOUSE_Catechol_O_methyltransferase_OS_Mus_musculus_OX_10090_GN_Comt_PE_1_SV_
MetazoaVertebrateEP00066Takifugurubripes_5691577_COMTB_DANRE_Catechol_O_methyltransferase_B_OS_Danio_rerio_OX_7955_GN_comtb_PE_2_SV_
MetazoaVertebrateEP00067Daniorerio_5720662_COMTB_DANRE_Catechol_O_methyltransferase_B_OS_Danio_rerio_OX_7955_GN_comtb_PE_2_SV_
MetazoaVertebrateEP00067Daniorerio_5718086_COMTA_DANRE_Catechol_O_methyltransferase_A_OS_Danio_rerio_OX_7955_GN_comta_PE_1_SV_
MetazoaVertebrateEP00079Xenopustropicalis_6361439_COMTB_DANRE_Catechol_O_methyltransferase_B_OS_Danio_rerio_OX_7955_GN_comtb_PE_2_SV_
MetazoaVertebrateEP00079Xenopustropicalis_6375170_COMTB_DANRE_Catechol_O_methyltransferase_B_OS_Danio_rerio_OX_7955_GN_comtb_PE_2_SV_
0.23

10C.

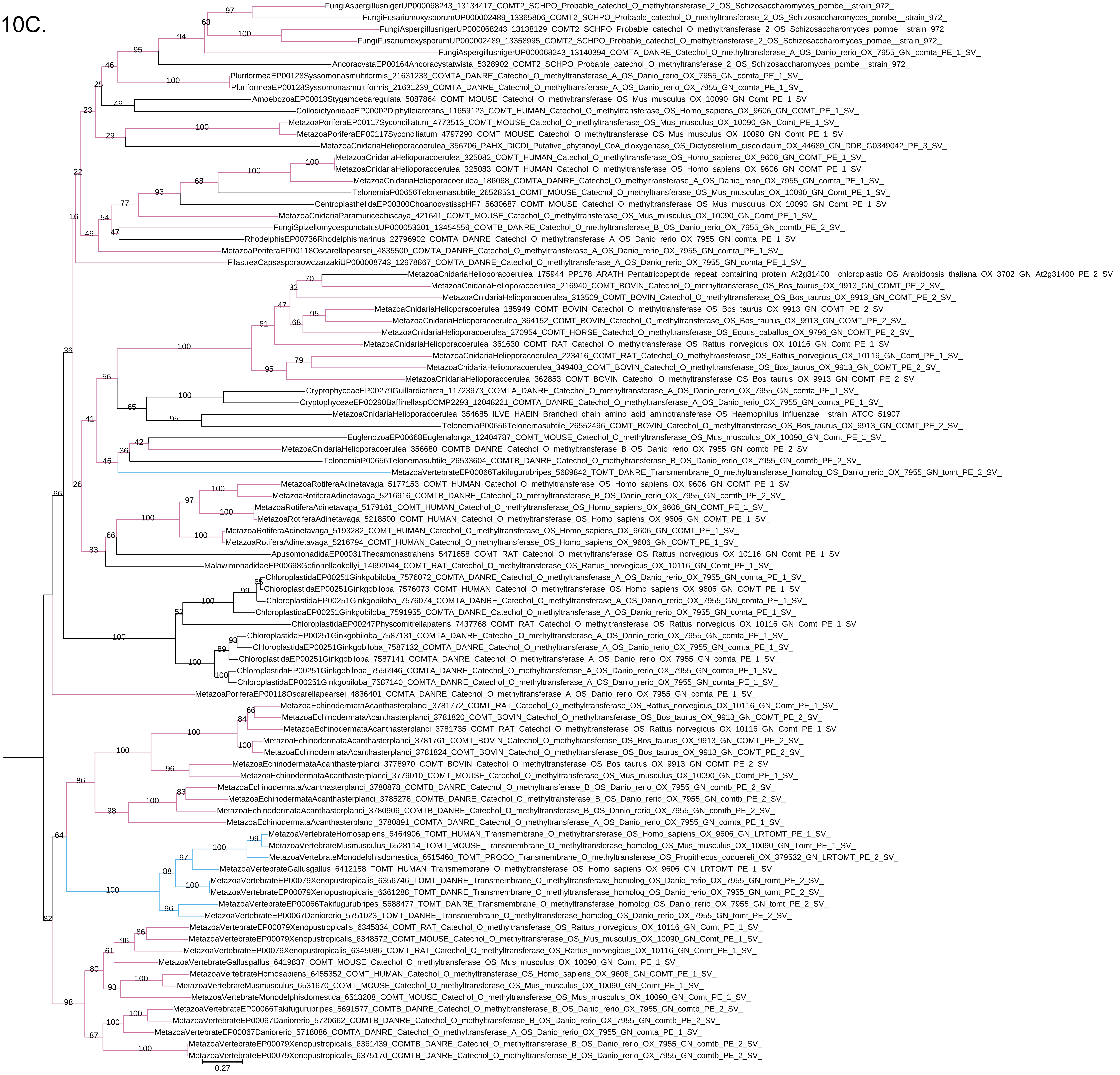
FungiAspergillusnigerUP000068243_13134417_COMT2_SCHPO_Probable_catechol_O_methyltransferase_2_OS_Schizosaccharomyces_pombe__strain_972_
FungiFusariumoxysporumUP000002489_13365806_COMT2_SCHPO_Probable_catechol_O_methyltransferase_2_OS_Schizosaccharomyces_pombe__strain_972_
FungiAspergillusnigerUP000068243_13138129_COMT2_SCHPO_Probable_catechol_O_methyltransferase_2_OS_Schizosaccharomyces_pombe__strain_972_
FungiFusariumoxysporumUP000002489_13358995_COMT2_SCHPO_Probable_catechol_O_methyltransferase_2_OS_Schizosaccharomyces_pombe__strain_972_
FungiAspergillusnigerUP000068243_13140394_COMTA_DANRE_Catechol_O_methyltransferase_A_OS_Danio_rerio_OX_7955_GN_comta_PE_1_SV_
AncoracystaEP00164Ancoracystatwista_5328902_COMT2_SCHPO_Probable_catechol_O_methyltransferase_2_OS_Schizosaccharomyces_pombe__strain_972_
PluriformeaEP00128Syssomonasmultiformis_21631238_COMTA_DANRE_Catechol_O_methyltransferase_A_OS_Danio_rerio_OX_7955_GN_comta_PE_1_SV_
PluriformeaEP00128Syssomonasmultiformis_21631239_COMTA_DANRE_Catechol_O_methyltransferase_A_OS_Danio_rerio_OX_7955_GN_comta_PE_1_SV_
AmoebozoaEP00013Stygamoebaregulata_5087864_COMT_MOUSE_Catechol_O_methyltransferase_OS_Mus_musculus_OX_10090_GN_Comt_PE_1_SV_
CollodictyonidaeEP00002Diphylleiarotans_11659123_COMT_HUMAN_Catechol_O_methyltransferase_OS_Homo_sapiens_OX_9606_GN_COMT_PE_1_SV_
MetazoaPoriferaEP00117Syconciliatum_4773513_COMT_MOUSE_Catechol_O_methyltransferase_OS_Mus_musculus_OX_10090_GN_Comt_PE_1_SV_
MetazoaPoriferaEP00117Syconciliatum_4797290_COMT_MOUSE_Catechol_O_methyltransferase_OS_Mus_musculus_OX_10090_GN_Comt_PE_1_SV_
MetazoaCnidariaHelioporacoerulea_356706_PAHX_DICDI_Putative_phytanoyl_CoA_dioxygenase_OS_Dictyostelium_discoideum_OX_44689_GN_DDB_G0349042_PE_3_SV_
MetazoaCnidariaHelioporacoerulea_325082_COMT_HUMAN_Catechol_O_methyltransferase_OS_Homo_sapiens_OX_9606_GN_COMT_PE_1_SV_
MetazoaCnidariaHelioporacoerulea_325083_COMT_HUMAN_Catechol_O_methyltransferase_OS_Homo_sapiens_OX_9606_GN_COMT_PE_1_SV_
MetazoaCnidariaHelioporacoerulea_186068_COMTA_DANRE_Catechol_O_methyltransferase_A_OS_Danio_rerio_OX_7955_GN_comta_PE_1_SV_
TelonemiaP00656Telonemasubtile_26528531_COMT_MOUSE_Catechol_O_methyltransferase_OS_Mus_musculus_OX_10090_GN_Comt_PE_1_SV_
CentroplasthelidaEP00300Choanocystissp HF7_5630687_COMT_MOUSE_Catechol_O_methyltransferase_OS_Mus_musculus_OX_10090_GN_Comt_PE_1_SV_
MetazoaCnidariaParamuriceabiscaya_421641_COMT_MOUSE_Catechol_O_methyltransferase_OS_Mus_musculus_OX_10090_GN_Comt_PE_1_SV_
FungiSpizellomycespunctatusUP000053201_13454559_COMTB_DANRE_Catechol_O_methyltransferase_B_OS_Danio_rerio_OX_7955_GN_comtb_PE_2_SV_
RhodelphisEP00736Rhodelphismarinus_22796902_COMTA_DANRE_Catechol_O_methyltransferase_A_OS_Danio_rerio_OX_7955_GN_comta_PE_1_SV_
MetazoaPoriferaEP00118Oscarellapearsei_4835500_COMTA_DANRE_Catechol_O_methyltransferase_A_OS_Danio_rerio_OX_7955_GN_comta_PE_1_SV_
FilastreaCapsasporaowczarzakiUP000008743_12978867_COMTA_DANRE_Catechol_O_methyltransferase_A_OS_Danio_rerio_OX_7955_GN_comta_PE_1_SV_
MetazoaCnidariaHelioporacoerulea_175944_PP178_ARATH_Pentatricopeptide_repeat_containing_protein_At2g31400__chloroplastic_OS_Arabidopsis_thaliana_OX_3702_GN_At2g31400_PE_2_SV_
MetazoaCnidariaHelioporacoerulea_216940_COMT_BOVIN_Catechol_O_methyltransferase_OS_Bos_taurus_OX_9913_GN_COMT_PE_2_SV_
MetazoaCnidariaHelioporacoerulea_313509_COMT_BOVIN_Catechol_O_methyltransferase_OS_Bos_taurus_OX_9913_GN_COMT_PE_2_SV_
MetazoaCnidariaHelioporacoerulea_185949_COMT_BOVIN_Catechol_O_methyltransferase_OS_Bos_taurus_OX_9913_GN_COMT_PE_2_SV_
MetazoaCnidariaHelioporacoerulea_364152_COMT_BOVIN_Catechol_O_methyltransferase_OS_Bos_taurus_OX_9913_GN_COMT_PE_2_SV_
MetazoaCnidariaHelioporacoerulea_270954_COMT_HORSE_Catechol_O_methyltransferase_OS_Equus_caballus_OX_9796_GN_COMT_PE_2_SV_
MetazoaCnidariaHelioporacoerulea_361630_COMT_RAT_Catechol_O_methyltransferase_OS_Rattus_norvegicus_OX_10116_GN_Comt_PE_1_SV_
MetazoaCnidariaHelioporacoerulea_223416_COMT_RAT_Catechol_O_methyltransferase_OS_Rattus_norvegicus_OX_10116_GN_Comt_PE_1_SV_
MetazoaCnidariaHelioporacoerulea_349403_COMT_BOVIN_Catechol_O_methyltransferase_OS_Bos_taurus_OX_9913_GN_COMT_PE_2_SV_
MetazoaCnidariaHelioporacoerulea_362853_COMT_BOVIN_Catechol_O_methyltransferase_OS_Bos_taurus_OX_9913_GN_COMT_PE_2_SV_
CryptophyceaeEP00279Guillardiatheta_11723973_COMTA_DANRE_Catechol_O_methyltransferase_A_OS_Danio_rerio_OX_7955_GN_comta_PE_1_SV_
CryptophyceaeEP00290BaffinellaspCCMP2293_12048221_COMTA_DANRE_Catechol_O_methyltransferase_A_OS_Danio_rerio_OX_7955_GN_comta_PE_1_SV_
MetazoaCnidariaHelioporacoerulea_354685_ILVE_HAEIN_Branched_chain_amino_acid_aminotransferase_OS_Haemophilus_influenzae__strain_ATCC_51907_
TelonemiaP00656Telonemasubtile_26552496_COMT_BOVIN_Catechol_O_methyltransferase_OS_Bos_taurus_OX_9913_GN_COMT_PE_2_SV_
EuglenozoaEP00668Euglenalonga_12404787_COMT_MOUSE_Catechol_O_methyltransferase_OS_Mus_musculus_OX_10090_GN_Comt_PE_1_SV_
MetazoaCnidariaHelioporacoerulea_356680_COMTB_DANRE_Catechol_O_methyltransferase_B_OS_Danio_rerio_OX_7955_GN_comtb_PE_2_SV_
TelonemiaP00656Telonemasubtile_26533604_COMTB_DANRE_Catechol_O_methyltransferase_B_OS_Danio_rerio_OX_7955_GN_comtb_PE_2_SV_
MetazoaVertebrateEP00066Takifugurubripes_5689842_TOMT_DANRE_Transmembrane_O_methyltransferase_homolog_OS_Danio_rerio_OX_7955_GN_tomt_PE_2_SV_
MetazoaRotiferaAdinetavaga_5177153_COMT_HUMAN_Catechol_O_methyltransferase_OS_Homo_sapiens_OX_9606_GN_COMT_PE_1_SV_
MetazoaRotiferaAdinetavaga_5216916_COMTB_DANRE_Catechol_O_methyltransferase_B_OS_Danio_rerio_OX_7955_GN_comtb_PE_2_SV_
MetazoaRotiferaAdinetavaga_5179161_COMT_HUMAN_Catechol_O_methyltransferase_OS_Homo_sapiens_OX_9606_GN_COMT_PE_1_SV_
MetazoaRotiferaAdinetavaga_5218500_COMT_HUMAN_Catechol_O_methyltransferase_OS_Homo_sapiens_OX_9606_GN_COMT_PE_1_SV_
MetazoaRotiferaAdinetavaga_5193282_COMT_HUMAN_Catechol_O_methyltransferase_OS_Homo_sapiens_OX_9606_GN_COMT_PE_1_SV_
MetazoaRotiferaAdinetavaga_5216794_COMT_HUMAN_Catechol_O_methyltransferase_OS_Homo_sapiens_OX_9606_GN_COMT_PE_1_SV_
ApusomonadidaEP00031Thecamonastrahens_5471658_COMT_RAT_Catechol_O_methyltransferase_OS_Rattus_norvegicus_OX_10116_GN_Comt_PE_1_SV_
MalawimonadidaeEP00698Gefionellaokellyi_14692044_COMT_RAT_Catechol_O_methyltransferase_OS_Rattus_norvegicus_OX_10116_GN_Comt_PE_1_SV_
ChloroplastidaEP00251Ginkgobiloba_7576072_COMTA_DANRE_Catechol_O_methyltransferase_A_OS_Danio_rerio_OX_7955_GN_comta_PE_1_SV_
ChloroplastidaEP00251Ginkgobiloba_7576073_COMT_HUMAN_Catechol_O_methyltransferase_OS_Homo_sapiens_OX_9606_GN_COMT_PE_1_SV_
ChloroplastidaEP00251Ginkgobiloba_7576074_COMTA_DANRE_Catechol_O_methyltransferase_A_OS_Danio_rerio_OX_7955_GN_comta_PE_1_SV_
ChloroplastidaEP00251Ginkgobiloba_7591955_COMTA_DANRE_Catechol_O_methyltransferase_A_OS_Danio_rerio_OX_7955_GN_comta_PE_1_SV_
ChloroplastidaEP00247Physcomitrellapatens_7437768_COMT_RAT_Catechol_O_methyltransferase_OS_Rattus_norvegicus_OX_10116_GN_Comt_PE_1_SV_
ChloroplastidaEP00251Ginkgobiloba_7587131_COMTA_DANRE_Catechol_O_methyltransferase_A_OS_Danio_rerio_OX_7955_GN_comta_PE_1_SV_
ChloroplastidaEP00251Ginkgobiloba_7587132_COMTA_DANRE_Catechol_O_methyltransferase_A_OS_Danio_rerio_OX_7955_GN_comta_PE_1_SV_
ChloroplastidaEP00251Ginkgobiloba_7587141_COMTA_DANRE_Catechol_O_methyltransferase_A_OS_Danio_rerio_OX_7955_GN_comta_PE_1_SV_
ChloroplastidaEP00251Ginkgobiloba_7556946_COMTA_DANRE_Catechol_O_methyltransferase_A_OS_Danio_rerio_OX_7955_GN_comta_PE_1_SV_
ChloroplastidaEP00251Ginkgobiloba_7587140_COMTA_DANRE_Catechol_O_methyltransferase_A_OS_Danio_rerio_OX_7955_GN_comta_PE_1_SV_
MetazoaPoriferaEP00118Oscarellapearsei_4836401_COMTA_DANRE_Catechol_O_methyltransferase_A_OS_Danio_rerio_OX_7955_GN_comta_PE_1_SV_
MetazoaEchinodermataAcanthasterplanci_3781772_COMT_RAT_Catechol_O_methyltransferase_OS_Rattus_norvegicus_OX_10116_GN_Comt_PE_1_SV_
MetazoaEchinodermataAcanthasterplanci_3781820_COMT_BOVIN_Catechol_O_methyltransferase_OS_Bos_taurus_OX_9913_GN_COMT_PE_2_SV_
MetazoaEchinodermataAcanthasterplanci_3781735_COMT_RAT_Catechol_O_methyltransferase_OS_Rattus_norvegicus_OX_10116_GN_Comt_PE_1_SV_
MetazoaEchinodermataAcanthasterplanci_3781761_COMT_BOVIN_Catechol_O_methyltransferase_OS_Bos_taurus_OX_9913_GN_COMT_PE_2_SV_
MetazoaEchinodermataAcanthasterplanci_3781824_COMT_BOVIN_Catechol_O_methyltransferase_OS_Bos_taurus_OX_9913_GN_COMT_PE_2_SV_
MetazoaEchinodermataAcanthasterplanci_3778970_COMT_BOVIN_Catechol_O_methyltransferase_OS_Bos_taurus_OX_9913_GN_COMT_PE_2_SV_
MetazoaEchinodermataAcanthasterplanci_3779010_COMT_MOUSE_Catechol_O_methyltransferase_OS_Mus_musculus_OX_10090_GN_Comt_PE_1_SV_
MetazoaEchinodermataAcanthasterplanci_3780878_COMTB_DANRE_Catechol_O_methyltransferase_B_OS_Danio_rerio_OX_7955_GN_comtb_PE_2_SV_
MetazoaEchinodermataAcanthasterplanci_3785278_COMTB_DANRE_Catechol_O_methyltransferase_B_OS_Danio_rerio_OX_7955_GN_comtb_PE_2_SV_
MetazoaEchinodermataAcanthasterplanci_3780906_COMTB_DANRE_Catechol_O_methyltransferase_B_OS_Danio_rerio_OX_7955_GN_comtb_PE_2_SV_
MetazoaEchinodermataAcanthasterplanci_3780891_COMTA_DANRE_Catechol_O_methyltransferase_A_OS_Danio_rerio_OX_7955_GN_comta_PE_1_SV_
MetazoaVertebrateHomosapiens_6464906_TOMT_HUMAN_Transmembrane_O_methyltransferase_OS_Homo_sapiens_OX_9606_GN_LRTOMT_PE_1_SV_
MetazoaVertebrateMusmusculus_6528114_TOMT_MOUSE_Transmembrane_O_methyltransferase_homolog_OS_Mus_musculus_OX_10090_GN_Tomt_PE_1_SV_
MetazoaVertebrateMonodelphisdomestica_6515460_TOMT_PROCO_Transmembrane_O_methyltransferase_OS_Propithecus_coquereli_OX_379532_GN_LRTOMT_PE_2_SV_
MetazoaVertebrateGallusgallus_6412158_TOMT_HUMAN_Transmembrane_O_methyltransferase_OS_Homo_sapiens_OX_9606_GN_LRTOMT_PE_1_SV_
MetazoaVertebrateEP00079Xenopustropicalis_6356746_TOMT_DANRE_Transmembrane_O_methyltransferase_homolog_OS_Danio_rerio_OX_7955_GN_tomt_PE_2_SV_
MetazoaVertebrateEP00079Xenopustropicalis_6361288_TOMT_DANRE_Transmembrane_O_methyltransferase_homolog_OS_Danio_rerio_OX_7955_GN_tomt_PE_2_SV_
MetazoaVertebrateEP00066Takifugurubripes_5688477_TOMT_DANRE_Transmembrane_O_methyltransferase_homolog_OS_Danio_rerio_OX_7955_GN_tomt_PE_2_SV_
MetazoaVertebrateEP00067Daniorerio_5751023_TOMT_DANRE_Transmembrane_O_methyltransferase_homolog_OS_Danio_rerio_OX_7955_GN_tomt_PE_2_SV_
MetazoaVertebrateEP00079Xenopustropicalis_6345834_COMT_RAT_Catechol_O_methyltransferase_OS_Rattus_norvegicus_OX_10116_GN_Comt_PE_1_SV_
MetazoaVertebrateEP00079Xenopustropicalis_6348572_COMT_MOUSE_Catechol_O_methyltransferase_OS_Mus_musculus_OX_10090_GN_Comt_PE_1_SV_
MetazoaVertebrateEP00079Xenopustropicalis_6345086_COMT_RAT_Catechol_O_methyltransferase_OS_Rattus_norvegicus_OX_10116_GN_Comt_PE_1_SV_
MetazoaVertebrateGallusgallus_6419837_COMT_MOUSE_Catechol_O_methyltransferase_OS_Mus_musculus_OX_10090_GN_Comt_PE_1_SV_
MetazoaVertebrateHomosapiens_6455352_COMT_HUMAN_Catechol_O_methyltransferase_OS_Homo_sapiens_OX_9606_GN_COMT_PE_1_SV_
MetazoaVertebrateMusmusculus_6531670_COMT_MOUSE_Catechol_O_methyltransferase_OS_Mus_musculus_OX_10090_GN_Comt_PE_1_SV_
MetazoaVertebrateMonodelphisdomestica_6513208_COMT_MOUSE_Catechol_O_methyltransferase_OS_Mus_musculus_OX_10090_GN_Comt_PE_1_SV_
MetazoaVertebrateEP00066Takifugurubripes_5691577_COMTB_DANRE_Catechol_O_methyltransferase_B_OS_Danio_rerio_OX_7955_GN_comtb_PE_2_SV_
MetazoaVertebrateEP00067Daniorerio_5720662_COMTB_DANRE_Catechol_O_methyltransferase_B_OS_Danio_rerio_OX_7955_GN_comtb_PE_2_SV_
MetazoaVertebrateEP00067Daniorerio_5718086_COMTA_DANRE_Catechol_O_methyltransferase_A_OS_Danio_rerio_OX_7955_GN_comta_PE_1_SV_
MetazoaVertebrateEP00079Xenopustropicalis_6361439_COMTB_DANRE_Catechol_O_methyltransferase_B_OS_Danio_rerio_OX_7955_GN_comtb_PE_2_SV_
MetazoaVertebrateEP00079Xenopustropicalis_6375170_COMTB_DANRE_Catechol_O_methyltransferase_B_OS_Danio_rerio_OX_7955_GN_comtb_PE_2_SV_
0.27

10D.

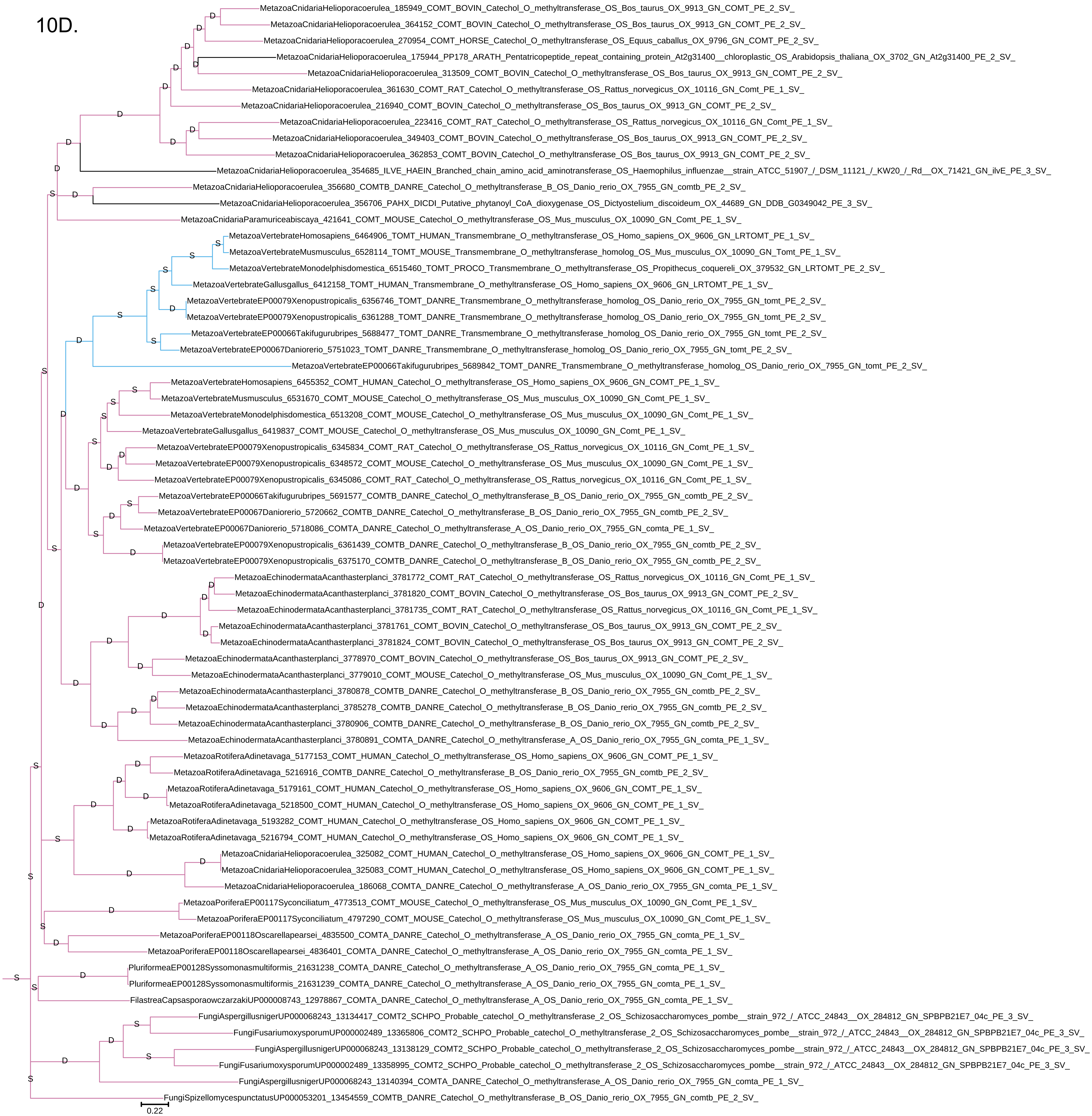
MetazoaCnidariaHelioporacoerulea_185949_COMT_BOVIN_Catechol_O_methyltransferase_OS_Bos_taurus_OX_9913_GN_COMT_PE_2_SV_
MetazoaCnidariaHelioporacoerulea_364152_COMT_BOVIN_Catechol_O_methyltransferase_OS_Bos_taurus_OX_9913_GN_COMT_PE_2_SV_
MetazoaCnidariaHelioporacoerulea_270954_COMT_HORSE_Catechol_O_methyltransferase_OS_Equus_caballus_OX_9796_GN_COMT_PE_2_SV_
MetazoaCnidariaHelioporacoerulea_175944_PP178_ARATH_Pentatricopeptide_repeat_containing_protein_At2g31400__chloroplastic_OS_Arabidopsis_thaliana_OX_3702_GN_At2g31400_PE_2_SV_
MetazoaCnidariaHelioporacoerulea_313509_COMT_BOVIN_Catechol_O_methyltransferase_OS_Bos_taurus_OX_9913_GN_COMT_PE_2_SV_
MetazoaCnidariaHelioporacoerulea_361630_COMT_RAT_Catechol_O_methyltransferase_OS_Rattus_norvegicus_OX_10116_GN_Comt_PE_1_SV_
MetazoaCnidariaHelioporacoerulea_216940_COMT_BOVIN_Catechol_O_methyltransferase_OS_Bos_taurus_OX_9913_GN_COMT_PE_2_SV_
MetazoaCnidariaHelioporacoerulea_223416_COMT_RAT_Catechol_O_methyltransferase_OS_Rattus_norvegicus_OX_10116_GN_Comt_PE_1_SV_
MetazoaCnidariaHelioporacoerulea_349403_COMT_BOVIN_Catechol_O_methyltransferase_OS_Bos_taurus_OX_9913_GN_COMT_PE_2_SV_
MetazoaCnidariaHelioporacoerulea_362853_COMT_BOVIN_Catechol_O_methyltransferase_OS_Bos_taurus_OX_9913_GN_COMT_PE_2_SV_
MetazoaCnidariaHelioporacoerulea_354685_ILVE_HAEIN_Branched_chain_amino_acid_aminotransferase_OS_Haemophilus_influenzae__strain_ATCC_51907_/_DSM_11121_/_KW20_/_Rd__OX_71421_GN_ilvE_PE_3_SV_
MetazoaCnidariaHelioporacoerulea_356680_COMTB_DANRE_Catechol_O_methyltransferase_B_OS_Danio_rerio_OX_7955_GN_comtb_PE_2_SV_
MetazoaCnidariaHelioporacoerulea_356706_PAHX_DICDI_Putative_phytanoyl_CoA_dioxygenase_OS_Dictyostelium_discoideum_OX_44689_GN_DDB_G0349042_PE_3_SV_
MetazoaCnidariaParamuriceabiscaya_421641_COMT_MOUSE_Catechol_O_methyltransferase_OS_Mus_musculus_OX_10090_GN_Comt_PE_1_SV_
MetazoaVertebrateHomosapiens_6464906_TOMT_HUMAN_Transmembrane_O_methyltransferase_OS_Homo_sapiens_OX_9606_GN_LRTOMT_PE_1_SV_
MetazoaVertebrateMusmusculus_6528114_TOMT_MOUSE_Transmembrane_O_methyltransferase_homolog_OS_Mus_musculus_OX_10090_GN_Tomt_PE_1_SV_
MetazoaVertebrateMonodelphisdomestica_6515460_TOMT_PROCO_Transmembrane_O_methyltransferase_OS_Propithecus_coquereli_OX_379532_GN_LRTOMT_PE_2_SV_
MetazoaVertebrateGallusgallus_6412158_TOMT_HUMAN_Transmembrane_O_methyltransferase_OS_Homo_sapiens_OX_9606_GN_LRTOMT_PE_1_SV_
MetazoaVertebrateEP00079Xenopustropicalis_6356746_TOMT_DANRE_Transmembrane_O_methyltransferase_homolog_OS_Danio_rerio_OX_7955_GN_tomt_PE_2_SV_
MetazoaVertebrateEP00079Xenopustropicalis_6361288_TOMT_DANRE_Transmembrane_O_methyltransferase_homolog_OS_Danio_rerio_OX_7955_GN_tomt_PE_2_SV_
MetazoaVertebrateEP00066Takifugurubripes_5688477_TOMT_DANRE_Transmembrane_O_methyltransferase_homolog_OS_Danio_rerio_OX_7955_GN_tomt_PE_2_SV_
MetazoaVertebrateEP00067Daniorerio_5751023_TOMT_DANRE_Transmembrane_O_methyltransferase_homolog_OS_Danio_rerio_OX_7955_GN_tomt_PE_2_SV_
MetazoaVertebrateEP00066Takifugurubripes_5689842_TOMT_DANRE_Transmembrane_O_methyltransferase_homolog_OS_Danio_rerio_OX_7955_GN_tomt_PE_2_SV_
MetazoaVertebrateHomosapiens_6455352_COMT_HUMAN_Catechol_O_methyltransferase_OS_Homo_sapiens_OX_9606_GN_COMT_PE_1_SV_
MetazoaVertebrateMusmusculus_6531670_COMT_MOUSE_Catechol_O_methyltransferase_OS_Mus_musculus_OX_10090_GN_Comt_PE_1_SV_
MetazoaVertebrateMonodelphisdomestica_6513208_COMT_MOUSE_Catechol_O_methyltransferase_OS_Mus_musculus_OX_10090_GN_Comt_PE_1_SV_
MetazoaVertebrateGallusgallus_6419837_COMT_MOUSE_Catechol_O_methyltransferase_OS_Mus_musculus_OX_10090_GN_Comt_PE_1_SV_
MetazoaVertebrateEP00079Xenopustropicalis_6345834_COMT_RAT_Catechol_O_methyltransferase_OS_Rattus_norvegicus_OX_10116_GN_Comt_PE_1_SV_
MetazoaVertebrateEP00079Xenopustropicalis_6348572_COMT_MOUSE_Catechol_O_methyltransferase_OS_Mus_musculus_OX_10090_GN_Comt_PE_1_SV_
MetazoaVertebrateEP00079Xenopustropicalis_6345086_COMT_RAT_Catechol_O_methyltransferase_OS_Rattus_norvegicus_OX_10116_GN_Comt_PE_1_SV_
MetazoaVertebrateEP00066Takifugurubripes_5691577_COMTB_DANRE_Catechol_O_methyltransferase_B_OS_Danio_rerio_OX_7955_GN_comtb_PE_2_SV_
MetazoaVertebrateEP00067Daniorerio_5720662_COMTB_DANRE_Catechol_O_methyltransferase_B_OS_Danio_rerio_OX_7955_GN_comtb_PE_2_SV_
MetazoaVertebrateEP00067Daniorerio_5718086_COMTA_DANRE_Catechol_O_methyltransferase_A_OS_Danio_rerio_OX_7955_GN_comta_PE_1_SV_
MetazoaVertebrateEP00079Xenopustropicalis_6361439_COMTB_DANRE_Catechol_O_methyltransferase_B_OS_Danio_rerio_OX_7955_GN_comtb_PE_2_SV_
MetazoaVertebrateEP00079Xenopustropicalis_6375170_COMTB_DANRE_Catechol_O_methyltransferase_B_OS_Danio_rerio_OX_7955_GN_comtb_PE_2_SV_
MetazoaEchinodermataAcanthasterplanci_3781772_COMT_RAT_Catechol_O_methyltransferase_OS_Rattus_norvegicus_OX_10116_GN_Comt_PE_1_SV_
MetazoaEchinodermataAcanthasterplanci_3781820_COMT_BOVIN_Catechol_O_methyltransferase_OS_Bos_taurus_OX_9913_GN_COMT_PE_2_SV_
MetazoaEchinodermataAcanthasterplanci_3781735_COMT_RAT_Catechol_O_methyltransferase_OS_Rattus_norvegicus_OX_10116_GN_Comt_PE_1_SV_
MetazoaEchinodermataAcanthasterplanci_3781761_COMT_BOVIN_Catechol_O_methyltransferase_OS_Bos_taurus_OX_9913_GN_COMT_PE_2_SV_
MetazoaEchinodermataAcanthasterplanci_3781824_COMT_BOVIN_Catechol_O_methyltransferase_OS_Bos_taurus_OX_9913_GN_COMT_PE_2_SV_
MetazoaEchinodermataAcanthasterplanci_3778970_COMT_BOVIN_Catechol_O_methyltransferase_OS_Bos_taurus_OX_9913_GN_COMT_PE_2_SV_
MetazoaEchinodermataAcanthasterplanci_3779010_COMT_MOUSE_Catechol_O_methyltransferase_OS_Mus_musculus_OX_10090_GN_Comt_PE_1_SV_
MetazoaEchinodermataAcanthasterplanci_3780878_COMTB_DANRE_Catechol_O_methyltransferase_B_OS_Danio_rerio_OX_7955_GN_comtb_PE_2_SV_
MetazoaEchinodermataAcanthasterplanci_3785278_COMTB_DANRE_Catechol_O_methyltransferase_B_OS_Danio_rerio_OX_7955_GN_comtb_PE_2_SV_
MetazoaEchinodermataAcanthasterplanci_3780906_COMTB_DANRE_Catechol_O_methyltransferase_B_OS_Danio_rerio_OX_7955_GN_comtb_PE_2_SV_
MetazoaEchinodermataAcanthasterplanci_3780891_COMTA_DANRE_Catechol_O_methyltransferase_A_OS_Danio_rerio_OX_7955_GN_comta_PE_1_SV_
MetazoaRotiferaAdinetavaga_5177153_COMT_HUMAN_Catechol_O_methyltransferase_OS_Homo_sapiens_OX_9606_GN_COMT_PE_1_SV_
MetazoaRotiferaAdinetavaga_5216916_COMTB_DANRE_Catechol_O_methyltransferase_B_OS_Danio_rerio_OX_7955_GN_comtb_PE_2_SV_
MetazoaRotiferaAdinetavaga_5179161_COMT_HUMAN_Catechol_O_methyltransferase_OS_Homo_sapiens_OX_9606_GN_COMT_PE_1_SV_
MetazoaRotiferaAdinetavaga_5218500_COMT_HUMAN_Catechol_O_methyltransferase_OS_Homo_sapiens_OX_9606_GN_COMT_PE_1_SV_
MetazoaRotiferaAdinetavaga_5193282_COMT_HUMAN_Catechol_O_methyltransferase_OS_Homo_sapiens_OX_9606_GN_COMT_PE_1_SV_
MetazoaRotiferaAdinetavaga_5216794_COMT_HUMAN_Catechol_O_methyltransferase_OS_Homo_sapiens_OX_9606_GN_COMT_PE_1_SV_
MetazoaCnidariaHelioporacoerulea_325082_COMT_HUMAN_Catechol_O_methyltransferase_OS_Homo_sapiens_OX_9606_GN_COMT_PE_1_SV_
MetazoaCnidariaHelioporacoerulea_325083_COMT_HUMAN_Catechol_O_methyltransferase_OS_Homo_sapiens_OX_9606_GN_COMT_PE_1_SV_
MetazoaCnidariaHelioporacoerulea_186068_COMTA_DANRE_Catechol_O_methyltransferase_A_OS_Danio_rerio_OX_7955_GN_comta_PE_1_SV_
MetazoaPoriferaEP00117Syconciliatum_4773513_COMT_MOUSE_Catechol_O_methyltransferase_OS_Mus_musculus_OX_10090_GN_Comt_PE_1_SV_
MetazoaPoriferaEP00117Syconciliatum_4797290_COMT_MOUSE_Catechol_O_methyltransferase_OS_Mus_musculus_OX_10090_GN_Comt_PE_1_SV_
MetazoaPoriferaEP00118Oscarellapearsei_4835500_COMTA_DANRE_Catechol_O_methyltransferase_A_OS_Danio_rerio_OX_7955_GN_comta_PE_1_SV_
MetazoaPoriferaEP00118Oscarellapearsei_4836401_COMTA_DANRE_Catechol_O_methyltransferase_A_OS_Danio_rerio_OX_7955_GN_comta_PE_1_SV_
PluriformeaEP00128Syssomonasmultiformis_21631238_COMTA_DANRE_Catechol_O_methyltransferase_A_OS_Danio_rerio_OX_7955_GN_comta_PE_1_SV_
PluriformeaEP00128Syssomonasmultiformis_21631239_COMTA_DANRE_Catechol_O_methyltransferase_A_OS_Danio_rerio_OX_7955_GN_comta_PE_1_SV_
FilastreaCapsasporaowczarzakiUP000008743_12978867_COMTA_DANRE_Catechol_O_methyltransferase_A_OS_Danio_rerio_OX_7955_GN_comta_PE_1_SV_
FungiAspergillusnigerUP000068243_13134417_COMT2_SCHPO_Probable_catechol_O_methyltransferase_2_OS_Schizosaccharomyces_pombe__strain_972_/_ATCC_24843__OX_284812_GN_SPBPB21E7_04c_PE_3_SV_
FungiFusariumoxysporumUP000002489_13365806_COMT2_SCHPO_Probable_catechol_O_methyltransferase_2_OS_Schizosaccharomyces_pombe__strain_972_/_ATCC_24843__OX_284812_GN_SPBPB21E7_04c_PE_3_SV_
FungiAspergillusnigerUP000068243_13138129_COMT2_SCHPO_Probable_catechol_O_methyltransferase_2_OS_Schizosaccharomyces_pombe__strain_972_/_ATCC_24843__OX_284812_GN_SPBPB21E7_04c_PE_3_SV_
FungiFusariumoxysporumUP000002489_13358995_COMT2_SCHPO_Probable_catechol_O_methyltransferase_2_OS_Schizosaccharomyces_pombe__strain_972_/_ATCC_24843__OX_284812_GN_SPBPB21E7_04c_PE_3_SV_
FungiAspergillusnigerUP000068243_13140394_COMTA_DANRE_Catechol_O_methyltransferase_A_OS_Danio_rerio_OX_7955_GN_comta_PE_1_SV_
FungiSpizellomycespunctatusUP000053201_13454559_COMTB_DANRE_Catechol_O_methyltransferase_B_OS_Danio_rerio_OX_7955_GN_comtb_PE_2_SV_
0.22

10E.

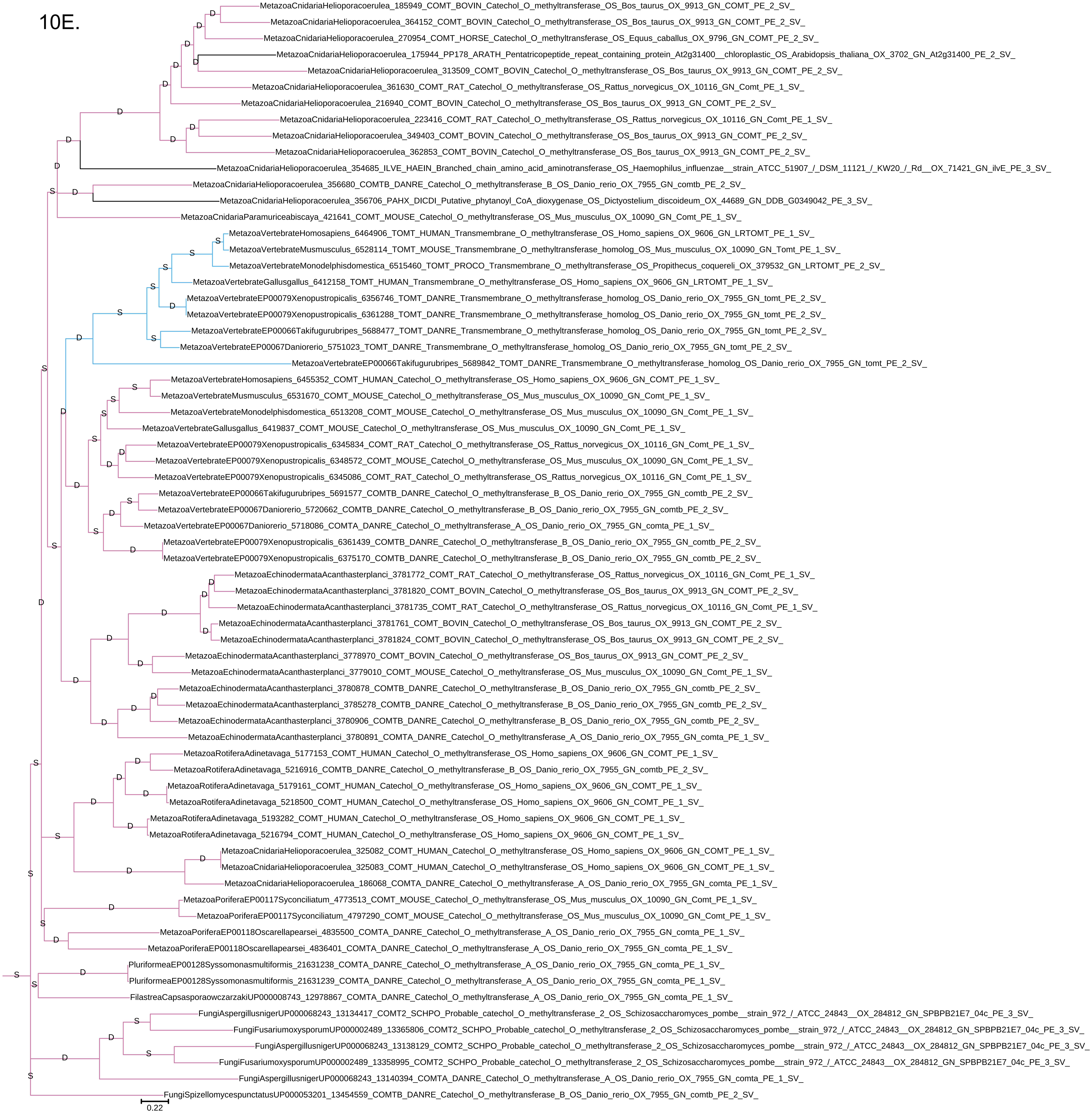

MetazoaCnidariaHelioporacoerulea_185949_COMT_BOVIN_Catechol_O_methyltransferase_OS_Bos_taurus_OX_9913_GN_COMT_PE_2_SV_
MetazoaCnidariaHelioporacoerulea_364152_COMT_BOVIN_Catechol_O_methyltransferase_OS_Bos_taurus_OX_9913_GN_COMT_PE_2_SV_
MetazoaCnidariaHelioporacoerulea_270954_COMT_HORSE_Catechol_O_methyltransferase_OS_Equus_caballus_OX_9796_GN_COMT_PE_2_SV_
MetazoaCnidariaHelioporacoerulea_175944_PP178_ARATH_Pentatricopeptide_repeat_containing_protein_At2g31400__chloroplastic_OS_Arabidopsis_thaliana_OX_3702_GN_At2g31400_PE_2_SV_
MetazoaCnidariaHelioporacoerulea_313509_COMT_BOVIN_Catechol_O_methyltransferase_OS_Bos_taurus_OX_9913_GN_COMT_PE_2_SV_
MetazoaCnidariaHelioporacoerulea_361630_COMT_RAT_Catechol_O_methyltransferase_OS_Rattus_norvegicus_OX_10116_GN_Comt_PE_1_SV_
MetazoaCnidariaHelioporacoerulea_216940_COMT_BOVIN_Catechol_O_methyltransferase_OS_Bos_taurus_OX_9913_GN_COMT_PE_2_SV_
MetazoaCnidariaHelioporacoerulea_223416_COMT_RAT_Catechol_O_methyltransferase_OS_Rattus_norvegicus_OX_10116_GN_Comt_PE_1_SV_
MetazoaCnidariaHelioporacoerulea_349403_COMT_BOVIN_Catechol_O_methyltransferase_OS_Bos_taurus_OX_9913_GN_COMT_PE_2_SV_
MetazoaCnidariaHelioporacoerulea_362853_COMT_BOVIN_Catechol_O_methyltransferase_OS_Bos_taurus_OX_9913_GN_COMT_PE_2_SV_
MetazoaCnidariaHelioporacoerulea_354685_ILVE_HAEIN_Branched_chain_amino_acid_aminotransferase_OS_Haemophilus_influenzae__strain_ATCC_51907_/_DSM_11121_/_KW20_/_Rd__OX_71421_GN_ilvE_PE_3_SV_
MetazoaCnidariaHelioporacoerulea_356680_COMTB_DANRE_Catechol_O_methyltransferase_B_OS_Danio_rerio_OX_7955_GN_comtb_PE_2_SV_
MetazoaCnidariaHelioporacoerulea_356706_PAHX_DICDI_Putative_phytanoyl_CoA_dioxygenase_OS_Dictyostelium_discoideum_OX_44689_GN_DDB_G0349042_PE_3_SV_
MetazoaCnidariaParamuriceabiscaya_421641_COMT_MOUSE_Catechol_O_methyltransferase_OS_Mus_musculus_OX_10090_GN_Comt_PE_1_SV_
MetazoaVertebrateHomosapiens_6464906_TOMT_HUMAN_Transmembrane_O_methyltransferase_OS_Homo_sapiens_OX_9606_GN_LRTOMT_PE_1_SV_
MetazoaVertebrateMusmusculus_6528114_TOMT_MOUSE_Transmembrane_O_methyltransferase_homolog_OS_Mus_musculus_OX_10090_GN_Tomt_PE_1_SV_
MetazoaVertebrateMonodelphisdomestica_6515460_TOMT_PROCO_Transmembrane_O_methyltransferase_OS_Propithecus_coquereli_OX_379532_GN_LRTOMT_PE_2_SV_
MetazoaVertebrateGallusgallus_6412158_TOMT_HUMAN_Transmembrane_O_methyltransferase_OS_Homo_sapiens_OX_9606_GN_LRTOMT_PE_1_SV_
MetazoaVertebrateEP00079Xenopustropicalis_6356746_TOMT_DANRE_Transmembrane_O_methyltransferase_homolog_OS_Danio_rerio_OX_7955_GN_tomt_PE_2_SV_
MetazoaVertebrateEP00079Xenopustropicalis_6361288_TOMT_DANRE_Transmembrane_O_methyltransferase_homolog_OS_Danio_rerio_OX_7955_GN_tomt_PE_2_SV_
MetazoaVertebrateEP00066Takifugurubripes_5688477_TOMT_DANRE_Transmembrane_O_methyltransferase_homolog_OS_Danio_rerio_OX_7955_GN_tomt_PE_2_SV_
MetazoaVertebrateEP00067Daniorerio_5751023_TOMT_DANRE_Transmembrane_O_methyltransferase_homolog_OS_Danio_rerio_OX_7955_GN_tomt_PE_2_SV_
MetazoaVertebrateEP00066Takifugurubripes_5689842_TOMT_DANRE_Transmembrane_O_methyltransferase_homolog_OS_Danio_rerio_OX_7955_GN_tomt_PE_2_SV_
MetazoaVertebrateHomosapiens_6455352_COMT_HUMAN_Catechol_O_methyltransferase_OS_Homo_sapiens_OX_9606_GN_COMT_PE_1_SV_
MetazoaVertebrateMusmusculus_6531670_COMT_MOUSE_Catechol_O_methyltransferase_OS_Mus_musculus_OX_10090_GN_Comt_PE_1_SV_
MetazoaVertebrateMonodelphisdomestica_6513208_COMT_MOUSE_Catechol_O_methyltransferase_OS_Mus_musculus_OX_10090_GN_Comt_PE_1_SV_
MetazoaVertebrateGallusgallus_6419837_COMT_MOUSE_Catechol_O_methyltransferase_OS_Mus_musculus_OX_10090_GN_Comt_PE_1_SV_
MetazoaVertebrateEP00079Xenopustropicalis_6345834_COMT_RAT_Catechol_O_methyltransferase_OS_Rattus_norvegicus_OX_10116_GN_Comt_PE_1_SV_
MetazoaVertebrateEP00079Xenopustropicalis_6348572_COMT_MOUSE_Catechol_O_methyltransferase_OS_Mus_musculus_OX_10090_GN_Comt_PE_1_SV_
MetazoaVertebrateEP00079Xenopustropicalis_6345086_COMT_RAT_Catechol_O_methyltransferase_OS_Rattus_norvegicus_OX_10116_GN_Comt_PE_1_SV_
MetazoaVertebrateEP00066Takifugurubripes_5691577_COMTB_DANRE_Catechol_O_methyltransferase_B_OS_Danio_rerio_OX_7955_GN_comtb_PE_2_SV_
MetazoaVertebrateEP00067Daniorerio_5720662_COMTB_DANRE_Catechol_O_methyltransferase_B_OS_Danio_rerio_OX_7955_GN_comtb_PE_2_SV_
MetazoaVertebrateEP00067Daniorerio_5718086_COMTA_DANRE_Catechol_O_methyltransferase_A_OS_Danio_rerio_OX_7955_GN_comta_PE_1_SV_
MetazoaVertebrateEP00079Xenopustropicalis_6361439_COMTB_DANRE_Catechol_O_methyltransferase_B_OS_Danio_rerio_OX_7955_GN_comtb_PE_2_SV_
MetazoaVertebrateEP00079Xenopustropicalis_6375170_COMTB_DANRE_Catechol_O_methyltransferase_B_OS_Danio_rerio_OX_7955_GN_comtb_PE_2_SV_
MetazoaEchinodermataAcanthasterplanci_3781772_COMT_RAT_Catechol_O_methyltransferase_OS_Rattus_norvegicus_OX_10116_GN_Comt_PE_1_SV_
MetazoaEchinodermataAcanthasterplanci_3781820_COMT_BOVIN_Catechol_O_methyltransferase_OS_Bos_taurus_OX_9913_GN_COMT_PE_2_SV_
MetazoaEchinodermataAcanthasterplanci_3781735_COMT_RAT_Catechol_O_methyltransferase_OS_Rattus_norvegicus_OX_10116_GN_Comt_PE_1_SV_
MetazoaEchinodermataAcanthasterplanci_3781761_COMT_BOVIN_Catechol_O_methyltransferase_OS_Bos_taurus_OX_9913_GN_COMT_PE_2_SV_
MetazoaEchinodermataAcanthasterplanci_3781824_COMT_BOVIN_Catechol_O_methyltransferase_OS_Bos_taurus_OX_9913_GN_COMT_PE_2_SV_
MetazoaEchinodermataAcanthasterplanci_3778970_COMT_BOVIN_Catechol_O_methyltransferase_OS_Bos_taurus_OX_9913_GN_COMT_PE_2_SV_
MetazoaEchinodermataAcanthasterplanci_3779010_COMT_MOUSE_Catechol_O_methyltransferase_OS_Mus_musculus_OX_10090_GN_Comt_PE_1_SV_
MetazoaEchinodermataAcanthasterplanci_3780878_COMTB_DANRE_Catechol_O_methyltransferase_B_OS_Danio_rerio_OX_7955_GN_comtb_PE_2_SV_
MetazoaEchinodermataAcanthasterplanci_3785278_COMTB_DANRE_Catechol_O_methyltransferase_B_OS_Danio_rerio_OX_7955_GN_comtb_PE_2_SV_
MetazoaEchinodermataAcanthasterplanci_3780906_COMTB_DANRE_Catechol_O_methyltransferase_B_OS_Danio_rerio_OX_7955_GN_comtb_PE_2_SV_
MetazoaEchinodermataAcanthasterplanci_3780891_COMTA_DANRE_Catechol_O_methyltransferase_A_OS_Danio_rerio_OX_7955_GN_comta_PE_1_SV_
MetazoaRotiferaAdinetavaga_5177153_COMT_HUMAN_Catechol_O_methyltransferase_OS_Homo_sapiens_OX_9606_GN_COMT_PE_1_SV_
MetazoaRotiferaAdinetavaga_5216916_COMTB_DANRE_Catechol_O_methyltransferase_B_OS_Danio_rerio_OX_7955_GN_comtb_PE_2_SV_
MetazoaRotiferaAdinetavaga_5179161_COMT_HUMAN_Catechol_O_methyltransferase_OS_Homo_sapiens_OX_9606_GN_COMT_PE_1_SV_
MetazoaRotiferaAdinetavaga_5218500_COMT_HUMAN_Catechol_O_methyltransferase_OS_Homo_sapiens_OX_9606_GN_COMT_PE_1_SV_
MetazoaRotiferaAdinetavaga_5193282_COMT_HUMAN_Catechol_O_methyltransferase_OS_Homo_sapiens_OX_9606_GN_COMT_PE_1_SV_
MetazoaRotiferaAdinetavaga_5216794_COMT_HUMAN_Catechol_O_methyltransferase_OS_Homo_sapiens_OX_9606_GN_COMT_PE_1_SV_
MetazoaCnidariaHelioporacoerulea_325082_COMT_HUMAN_Catechol_O_methyltransferase_OS_Homo_sapiens_OX_9606_GN_COMT_PE_1_SV_
MetazoaCnidariaHelioporacoerulea_325083_COMT_HUMAN_Catechol_O_methyltransferase_OS_Homo_sapiens_OX_9606_GN_COMT_PE_1_SV_
MetazoaCnidariaHelioporacoerulea_186068_COMTA_DANRE_Catechol_O_methyltransferase_A_OS_Danio_rerio_OX_7955_GN_comta_PE_1_SV_
MetazoaPoriferaEP00117Syconciliatum_4773513_COMT_MOUSE_Catechol_O_methyltransferase_OS_Mus_musculus_OX_10090_GN_Comt_PE_1_SV_
MetazoaPoriferaEP00117Syconciliatum_4797290_COMT_MOUSE_Catechol_O_methyltransferase_OS_Mus_musculus_OX_10090_GN_Comt_PE_1_SV_
MetazoaPoriferaEP00118Oscarellapearsei_4835500_COMTA_DANRE_Catechol_O_methyltransferase_A_OS_Danio_rerio_OX_7955_GN_comta_PE_1_SV_
MetazoaPoriferaEP00118Oscarellapearsei_4836401_COMTA_DANRE_Catechol_O_methyltransferase_A_OS_Danio_rerio_OX_7955_GN_comta_PE_1_SV_
PluriformeaEP00128Syssomonasmultiformis_21631238_COMTA_DANRE_Catechol_O_methyltransferase_A_OS_Danio_rerio_OX_7955_GN_comta_PE_1_SV_
PluriformeaEP00128Syssomonasmultiformis_21631239_COMTA_DANRE_Catechol_O_methyltransferase_A_OS_Danio_rerio_OX_7955_GN_comta_PE_1_SV_
FilastreaCapsasporaowczarzakiUP000008743_12978867_COMTA_DANRE_Catechol_O_methyltransferase_A_OS_Danio_rerio_OX_7955_GN_comta_PE_1_SV_
FungiAspergillusnigerUP000068243_13134417_COMT2_SCHPO_Probable_catechol_O_methyltransferase_2_OS_Schizosaccharomyces_pombe__strain_972_/_ATCC_24843__OX_284812_GN_SPBPB21E7_04c_PE_3_SV_
FungiFusariumoxysporumUP000002489_13365806_COMT2_SCHPO_Probable_catechol_O_methyltransferase_2_OS_Schizosaccharomyces_pombe__strain_972_/_ATCC_24843__OX_284812_GN_SPBPB21E7_04c_PE_3_SV_
FungiAspergillusnigerUP000068243_13138129_COMT2_SCHPO_Probable_catechol_O_methyltransferase_2_OS_Schizosaccharomyces_pombe__strain_972_/_ATCC_24843__OX_284812_GN_SPBPB21E7_04c_PE_3_SV_
FungiFusariumoxysporumUP000002489_13358995_COMT2_SCHPO_Probable_catechol_O_methyltransferase_2_OS_Schizosaccharomyces_pombe__strain_972_/_ATCC_24843__OX_284812_GN_SPBPB21E7_04c_PE_3_SV_
FungiAspergillusnigerUP000068243_13140394_COMTA_DANRE_Catechol_O_methyltransferase_A_OS_Danio_rerio_OX_7955_GN_comta_PE_1_SV_
FungiSpizellomycespunctatusUP000053201_13454559_COMTB_DANRE_Catechol_O_methyltransferase_B_OS_Danio_rerio_OX_7955_GN_comtb_PE_2_SV_
0.22

10F.

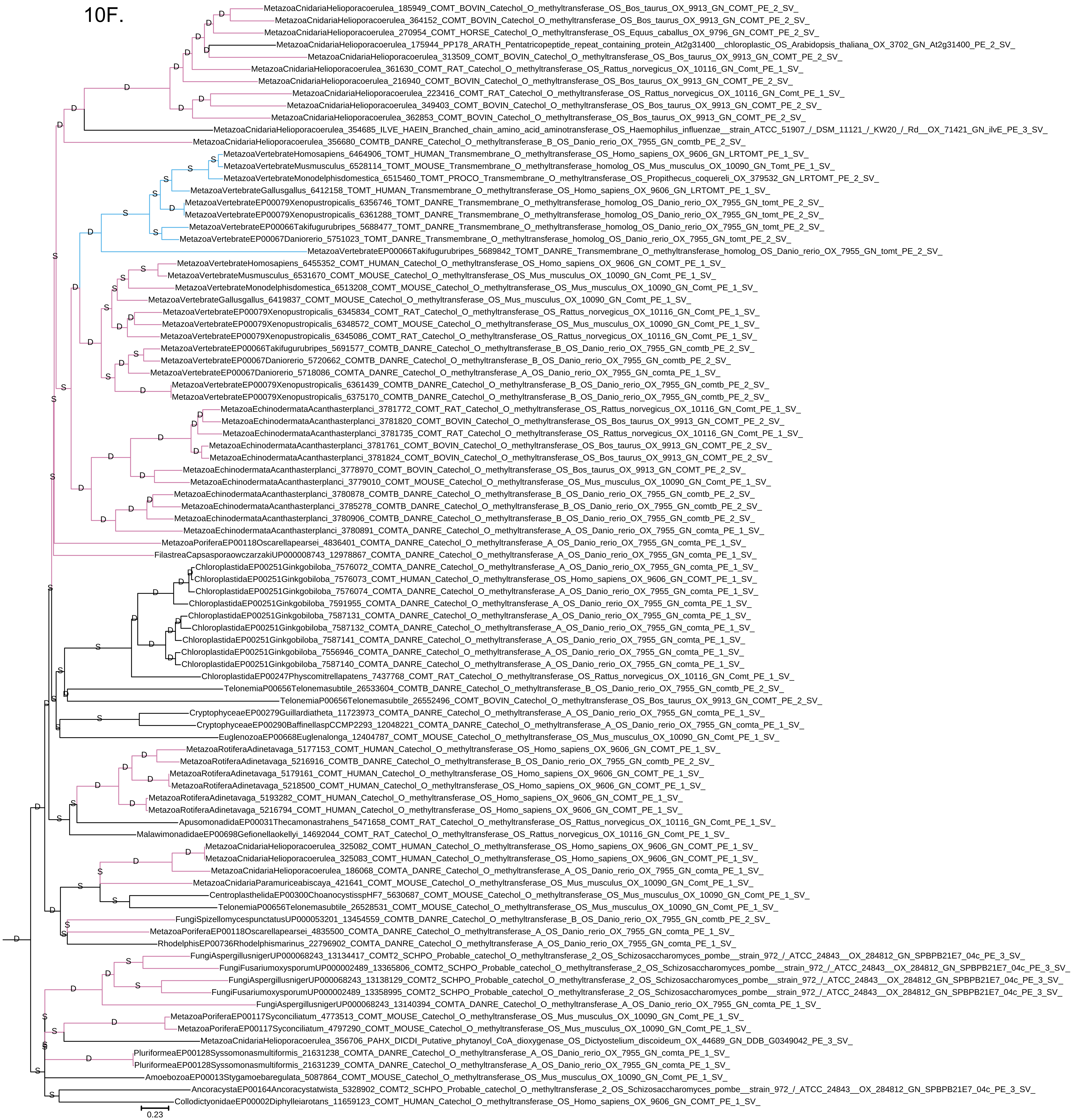

MetazoaCnidariaHelioporacoerulea_185949_COMT_BOVIN_Catechol_O_methyltransferase_OS_Bos_taurus_OX_9913_GN_COMT_PE_2_SV_
MetazoaCnidariaHelioporacoerulea_364152_COMT_BOVIN_Catechol_O_methyltransferase_OS_Bos_taurus_OX_9913_GN_COMT_PE_2_SV_
MetazoaCnidariaHelioporacoerulea_270954_COMT_HORSE_Catechol_O_methyltransferase_OS_Equus_caballus_OX_9796_GN_COMT_PE_2_SV_
MetazoaCnidariaHelioporacoerulea_175944_PP178_ARATH_Pentatricopeptide_repeat_containing_protein_At2g31400__chloroplastic_OS_Arabidopsis_thaliana_OX_3702_GN_At2g31400_PE_2_SV_
MetazoaCnidariaHelioporacoerulea_313509_COMT_BOVIN_Catechol_O_methyltransferase_OS_Bos_taurus_OX_9913_GN_COMT_PE_2_SV_
MetazoaCnidariaHelioporacoerulea_361630_COMT_RAT_Catechol_O_methyltransferase_OS_Rattus_norvegicus_OX_10116_GN_Comt_PE_1_SV_
MetazoaCnidariaHelioporacoerulea_216940_COMT_BOVIN_Catechol_O_methyltransferase_OS_Bos_taurus_OX_9913_GN_COMT_PE_2_SV_
MetazoaCnidariaHelioporacoerulea_223416_COMT_RAT_Catechol_O_methyltransferase_OS_Rattus_norvegicus_OX_10116_GN_Comt_PE_1_SV_
MetazoaCnidariaHelioporacoerulea_349403_COMT_BOVIN_Catechol_O_methyltransferase_OS_Bos_taurus_OX_9913_GN_COMT_PE_2_SV_
MetazoaCnidariaHelioporacoerulea_362853_COMT_BOVIN_Catechol_O_methyltransferase_OS_Bos_taurus_OX_9913_GN_COMT_PE_2_SV_
MetazoaCnidariaHelioporacoerulea_354685_ILVE_HAEIN_Branched_chain_amino_acid_aminotransferase_OS_Haemophilus_influenzae__strain_ATCC_51907_/_DSM_11121_/_KW20_/_Rd__OX_71421_GN_ilvE_PE_3_SV_
MetazoaCnidariaHelioporacoerulea_356680_COMTB_DANRE_Catechol_O_methyltransferase_B_OS_Danio_rerio_OX_7955_GN_comtb_PE_2_SV_
MetazoaVertebrateHomosapiens_6464906_TOMT_HUMAN_Transmembrane_O_methyltransferase_OS_Homo_sapiens_OX_9606_GN_LRTOMT_PE_1_SV_
MetazoaVertebrateMusmusculus_6528114_TOMT_MOUSE_Transmembrane_O_methyltransferase_homolog_OS_Mus_musculus_OX_10090_GN_Tomt_PE_1_SV_
MetazoaVertebrateMonodelphisdomestica_6515460_TOMT_PROCO_Transmembrane_O_methyltransferase_OS_Propithecus_coquereli_OX_379532_GN_LRTOMT_PE_2_SV_
MetazoaVertebrateGallusgallus_6412158_TOMT_HUMAN_Transmembrane_O_methyltransferase_OS_Homo_sapiens_OX_9606_GN_LRTOMT_PE_1_SV_
MetazoaVertebrateEP00079Xenopustropicalis_6356746_TOMT_DANRE_Transmembrane_O_methyltransferase_homolog_OS_Danio_rerio_OX_7955_GN_tomt_PE_2_SV_
MetazoaVertebrateEP00079Xenopustropicalis_6361288_TOMT_DANRE_Transmembrane_O_methyltransferase_homolog_OS_Danio_rerio_OX_7955_GN_tomt_PE_2_SV_
MetazoaVertebrateEP00066Takifugurubripes_5688477_TOMT_DANRE_Transmembrane_O_methyltransferase_homolog_OS_Danio_rerio_OX_7955_GN_tomt_PE_2_SV_
MetazoaVertebrateEP00067Daniorerio_5751023_TOMT_DANRE_Transmembrane_O_methyltransferase_homolog_OS_Danio_rerio_OX_7955_GN_tomt_PE_2_SV_
MetazoaVertebrateEP00066Takifugurubripes_5689842_TOMT_DANRE_Transmembrane_O_methyltransferase_homolog_OS_Danio_rerio_OX_7955_GN_tomt_PE_2_SV_
MetazoaVertebrateHomosapiens_6455352_COMT_HUMAN_Catechol_O_methyltransferase_OS_Homo_sapiens_OX_9606_GN_COMT_PE_1_SV_
MetazoaVertebrateMusmusculus_6531670_COMT_MOUSE_Catechol_O_methyltransferase_OS_Mus_musculus_OX_10090_GN_Comt_PE_1_SV_
MetazoaVertebrateMonodelphisdomestica_6513208_COMT_MOUSE_Catechol_O_methyltransferase_OS_Mus_musculus_OX_10090_GN_Comt_PE_1_SV_
MetazoaVertebrateGallusgallus_6419837_COMT_MOUSE_Catechol_O_methyltransferase_OS_Mus_musculus_OX_10090_GN_Comt_PE_1_SV_
MetazoaVertebrateEP00079Xenopustropicalis_6345834_COMT_RAT_Catechol_O_methyltransferase_OS_Rattus_norvegicus_OX_10116_GN_Comt_PE_1_SV_
MetazoaVertebrateEP00079Xenopustropicalis_6348572_COMT_MOUSE_Catechol_O_methyltransferase_OS_Mus_musculus_OX_10090_GN_Comt_PE_1_SV_
MetazoaVertebrateEP00079Xenopustropicalis_6345086_COMT_RAT_Catechol_O_methyltransferase_OS_Rattus_norvegicus_OX_10116_GN_Comt_PE_1_SV_
MetazoaVertebrateEP00066Takifugurubripes_5691577_COMTB_DANRE_Catechol_O_methyltransferase_B_OS_Danio_rerio_OX_7955_GN_comtb_PE_2_SV_
MetazoaVertebrateEP00067Daniorerio_5720662_COMTB_DANRE_Catechol_O_methyltransferase_B_OS_Danio_rerio_OX_7955_GN_comtb_PE_2_SV_
MetazoaVertebrateEP00067Daniorerio_5718086_COMTA_DANRE_Catechol_O_methyltransferase_A_OS_Danio_rerio_OX_7955_GN_comta_PE_1_SV_
MetazoaVertebrateEP00079Xenopustropicalis_6361439_COMTB_DANRE_Catechol_O_methyltransferase_B_OS_Danio_rerio_OX_7955_GN_comtb_PE_2_SV_
MetazoaVertebrateEP00079Xenopustropicalis_6375170_COMTB_DANRE_Catechol_O_methyltransferase_B_OS_Danio_rerio_OX_7955_GN_comtb_PE_2_SV_
MetazoaEchinodermataAcanthasterplanci_3781772_COMT_RAT_Catechol_O_methyltransferase_OS_Rattus_norvegicus_OX_10116_GN_Comt_PE_1_SV_
MetazoaEchinodermataAcanthasterplanci_3781820_COMT_BOVIN_Catechol_O_methyltransferase_OS_Bos_taurus_OX_9913_GN_COMT_PE_2_SV_
MetazoaEchinodermataAcanthasterplanci_3781735_COMT_RAT_Catechol_O_methyltransferase_OS_Rattus_norvegicus_OX_10116_GN_Comt_PE_1_SV_
MetazoaEchinodermataAcanthasterplanci_3781761_COMT_BOVIN_Catechol_O_methyltransferase_OS_Bos_taurus_OX_9913_GN_COMT_PE_2_SV_
MetazoaEchinodermataAcanthasterplanci_3781824_COMT_BOVIN_Catechol_O_methyltransferase_OS_Bos_taurus_OX_9913_GN_COMT_PE_2_SV_
MetazoaEchinodermataAcanthasterplanci_3778970_COMT_BOVIN_Catechol_O_methyltransferase_OS_Bos_taurus_OX_9913_GN_COMT_PE_2_SV_
MetazoaEchinodermataAcanthasterplanci_3779010_COMT_MOUSE_Catechol_O_methyltransferase_OS_Mus_musculus_OX_10090_GN_Comt_PE_1_SV_
MetazoaEchinodermataAcanthasterplanci_3780878_COMTB_DANRE_Catechol_O_methyltransferase_B_OS_Danio_rerio_OX_7955_GN_comtb_PE_2_SV_
MetazoaEchinodermataAcanthasterplanci_3785278_COMTB_DANRE_Catechol_O_methyltransferase_B_OS_Danio_rerio_OX_7955_GN_comtb_PE_2_SV_
MetazoaEchinodermataAcanthasterplanci_3780906_COMTB_DANRE_Catechol_O_methyltransferase_B_OS_Danio_rerio_OX_7955_GN_comtb_PE_2_SV_
MetazoaEchinodermataAcanthasterplanci_3780891_COMTA_DANRE_Catechol_O_methyltransferase_A_OS_Danio_rerio_OX_7955_GN_comta_PE_1_SV_
MetazoaPoriferaEP00118Oscarellapearsei_4836401_COMTA_DANRE_Catechol_O_methyltransferase_A_OS_Danio_rerio_OX_7955_GN_comta_PE_1_SV_
FilastreaCapsasporaowczarzakiUP000008743_12978867_COMTA_DANRE_Catechol_O_methyltransferase_A_OS_Danio_rerio_OX_7955_GN_comta_PE_1_SV_
ChloroplastidaEP00251Ginkgobiloba_7576072_COMTA_DANRE_Catechol_O_methyltransferase_A_OS_Danio_rerio_OX_7955_GN_comta_PE_1_SV_
ChloroplastidaEP00251Ginkgobiloba_7576073_COMT_HUMAN_Catechol_O_methyltransferase_OS_Homo_sapiens_OX_9606_GN_COMT_PE_1_SV_
ChloroplastidaEP00251Ginkgobiloba_7576074_COMTA_DANRE_Catechol_O_methyltransferase_A_OS_Danio_rerio_OX_7955_GN_comta_PE_1_SV_
ChloroplastidaEP00251Ginkgobiloba_7591955_COMTA_DANRE_Catechol_O_methyltransferase_A_OS_Danio_rerio_OX_7955_GN_comta_PE_1_SV_
ChloroplastidaEP00251Ginkgobiloba_7587131_COMTA_DANRE_Catechol_O_methyltransferase_A_OS_Danio_rerio_OX_7955_GN_comta_PE_1_SV_
ChloroplastidaEP00251Ginkgobiloba_7587132_COMTA_DANRE_Catechol_O_methyltransferase_A_OS_Danio_rerio_OX_7955_GN_comta_PE_1_SV_
ChloroplastidaEP00251Ginkgobiloba_7587141_COMTA_DANRE_Catechol_O_methyltransferase_A_OS_Danio_rerio_OX_7955_GN_comta_PE_1_SV_
ChloroplastidaEP00251Ginkgobiloba_7556946_COMTA_DANRE_Catechol_O_methyltransferase_A_OS_Danio_rerio_OX_7955_GN_comta_PE_1_SV_
ChloroplastidaEP00251Ginkgobiloba_7587140_COMTA_DANRE_Catechol_O_methyltransferase_A_OS_Danio_rerio_OX_7955_GN_comta_PE_1_SV_
ChloroplastidaEP00247Physcomitrellapatens_7437768_COMT_RAT_Catechol_O_methyltransferase_OS_Rattus_norvegicus_OX_10116_GN_Comt_PE_1_SV_
TelonemiaP00656Telonemasubtile_26533604_COMTB_DANRE_Catechol_O_methyltransferase_B_OS_Danio_rerio_OX_7955_GN_comtb_PE_2_SV_
TelonemiaP00656Telonemasubtile_26552496_COMT_BOVIN_Catechol_O_methyltransferase_OS_Bos_taurus_OX_9913_GN_COMT_PE_2_SV_
CryptophyceaeEP00279Guillardiatheta_11723973_COMTA_DANRE_Catechol_O_methyltransferase_A_OS_Danio_rerio_OX_7955_GN_comta_PE_1_SV_
CryptophyceaeEP00290BaffinellaspCCMP2293_12048221_COMTA_DANRE_Catechol_O_methyltransferase_A_OS_Danio_rerio_OX_7955_GN_comta_PE_1_SV_
EuglenozoaEP00668Euglenalonga_12404787_COMT_MOUSE_Catechol_O_methyltransferase_OS_Mus_musculus_OX_10090_GN_Comt_PE_1_SV_
MetazoaRotiferaAdinetavaga_5177153_COMT_HUMAN_Catechol_O_methyltransferase_OS_Homo_sapiens_OX_9606_GN_COMT_PE_1_SV_
MetazoaRotiferaAdinetavaga_5216916_COMTB_DANRE_Catechol_O_methyltransferase_B_OS_Danio_rerio_OX_7955_GN_comtb_PE_2_SV_
MetazoaRotiferaAdinetavaga_5179161_COMT_HUMAN_Catechol_O_methyltransferase_OS_Homo_sapiens_OX_9606_GN_COMT_PE_1_SV_
MetazoaRotiferaAdinetavaga_5218500_COMT_HUMAN_Catechol_O_methyltransferase_OS_Homo_sapiens_OX_9606_GN_COMT_PE_1_SV_
MetazoaRotiferaAdinetavaga_5193282_COMT_HUMAN_Catechol_O_methyltransferase_OS_Homo_sapiens_OX_9606_GN_COMT_PE_1_SV_
MetazoaRotiferaAdinetavaga_5216794_COMT_HUMAN_Catechol_O_methyltransferase_OS_Homo_sapiens_OX_9606_GN_COMT_PE_1_SV_
ApusomonadidaEP00031Thecamonastrahens_5471658_COMT_RAT_Catechol_O_methyltransferase_OS_Rattus_norvegicus_OX_10116_GN_Comt_PE_1_SV_
MalawimonadidaeEP00698Gefionellaokellyi_14692044_COMT_RAT_Catechol_O_methyltransferase_OS_Rattus_norvegicus_OX_10116_GN_Comt_PE_1_SV_
MetazoaCnidariaHelioporacoerulea_325082_COMT_HUMAN_Catechol_O_methyltransferase_OS_Homo_sapiens_OX_9606_GN_COMT_PE_1_SV_
MetazoaCnidariaHelioporacoerulea_325083_COMT_HUMAN_Catechol_O_methyltransferase_OS_Homo_sapiens_OX_9606_GN_COMT_PE_1_SV_
MetazoaCnidariaHelioporacoerulea_186068_COMTA_DANRE_Catechol_O_methyltransferase_A_OS_Danio_rerio_OX_7955_GN_comta_PE_1_SV_
MetazoaCnidariaParamuriceabiscaya_421641_COMT_MOUSE_Catechol_O_methyltransferase_OS_Mus_musculus_OX_10090_GN_Comt_PE_1_SV_
CentroplasthelidaEP00300ChoanocystisspHF7_5630687_COMT_MOUSE_Catechol_O_methyltransferase_OS_Mus_musculus_OX_10090_GN_Comt_PE_1_SV_
TelonemiaP00656Telonemasubtile_26528531_COMT_MOUSE_Catechol_O_methyltransferase_OS_Mus_musculus_OX_10090_GN_Comt_PE_1_SV_
FungiSpizellomycespunctatusUP000053201_13454559_COMTB_DANRE_Catechol_O_methyltransferase_B_OS_Danio_rerio_OX_7955_GN_comtb_PE_2_SV_
MetazoaPoriferaEP00118Oscarellapearsei_4835500_COMTA_DANRE_Catechol_O_methyltransferase_A_OS_Danio_rerio_OX_7955_GN_comta_PE_1_SV_
RhodelphisEP00736Rhodelphismarinus_22796902_COMTA_DANRE_Catechol_O_methyltransferase_A_OS_Danio_rerio_OX_7955_GN_comta_PE_1_SV_
FungiAspergillusnigerUP000068243_13134417_COMT2_SCHPO_Probable_catechol_O_methyltransferase_2_OS_Schizosaccharomyces_pombe__strain_972_/_ATCC_24843__OX_284812_GN_SPBPB21E7_04c_PE_3_SV_
FungiFusariumoxysporumUP000002489_13365806_COMT2_SCHPO_Probable_catechol_O_methyltransferase_2_OS_Schizosaccharomyces_pombe__strain_972_/_ATCC_24843__OX_284812_GN_SPBPB21E7_04c_PE_3_SV_
FungiAspergillusnigerUP000068243_13138129_COMT2_SCHPO_Probable_catechol_O_methyltransferase_2_OS_Schizosaccharomyces_pombe__strain_972_/_ATCC_24843__OX_284812_GN_SPBPB21E7_04c_PE_3_SV_
FungiFusariumoxysporumUP000002489_13358995_COMT2_SCHPO_Probable_catechol_O_methyltransferase_2_OS_Schizosaccharomyces_pombe__strain_972_/_ATCC_24843__OX_284812_GN_SPBPB21E7_04c_PE_3_SV_
FungiAspergillusnigerUP000068243_13140394_COMTA_DANRE_Catechol_O_methyltransferase_A_OS_Danio_rerio_OX_7955_GN_comta_PE_1_SV_
MetazoaPoriferaEP00117Syconciliatum_4773513_COMT_MOUSE_Catechol_O_methyltransferase_OS_Mus_musculus_OX_10090_GN_Comt_PE_1_SV_
MetazoaPoriferaEP00117Syconciliatum_4797290_COMT_MOUSE_Catechol_O_methyltransferase_OS_Mus_musculus_OX_10090_GN_Comt_PE_1_SV_
MetazoaCnidariaHelioporacoerulea_356706_PAHX_DICDI_Putative_phytanoyl_CoA_dioxygenase_OS_Dictyostelium_discoideum_OX_44689_GN_DDB_G0349042_PE_3_SV_
PluriformeaEP00128Syssomonasmultiformis_21631238_COMTA_DANRE_Catechol_O_methyltransferase_A_OS_Danio_rerio_OX_7955_GN_comta_PE_1_SV_
PluriformeaEP00128Syssomonasmultiformis_21631239_COMTA_DANRE_Catechol_O_methyltransferase_A_OS_Danio_rerio_OX_7955_GN_comta_PE_1_SV_
AmoebozoaEP00013Stygamoebaregulata_5087864_COMT_MOUSE_Catechol_O_methyltransferase_OS_Mus_musculus_OX_10090_GN_Comt_PE_1_SV_
AncoracystaEP00164Ancoracystatwista_5328902_COMT2_SCHPO_Probable_catechol_O_methyltransferase_2_OS_Schizosaccharomyces_pombe__strain_972_/_ATCC_24843__OX_284812_GN_SPBPB21E7_04c_PE_3_SV_
CollodictyonidaeEP00002Diphylleiarotans_11659123_COMT_HUMAN_Catechol_O_methyltransferase_OS_Homo_sapiens_OX_9606_GN_COMT_PE_1_SV_
0.23

Supplementary Figure 11: Evolution of HNMTs. Simplified illustration of the gene tree to species tree reconciliation. Silhouettes obtained from Phylopic.org. Silhouette images are by Andrew R. Gehrke (*Hofstenia miamia*); Daniel Jaron (*Mus musculus*); Mali'o Kodis, photograph by Ching (http://www.flickr.com/photos/36302473@N03/) (*Chrysaora fuscescens*); Mario Quevedo (Asteriidae); Oliver Voigt (*Trichoplax adhaerens*); Ramiro Morales-Hojas (*Drosophila americana*); Steven Haddock, Jellywatch.org (*Hormiphora californensis*); and Tess Linden (*Salpingoeca rosetta*). HNMT = histamine-N-methyltransferase.

11.

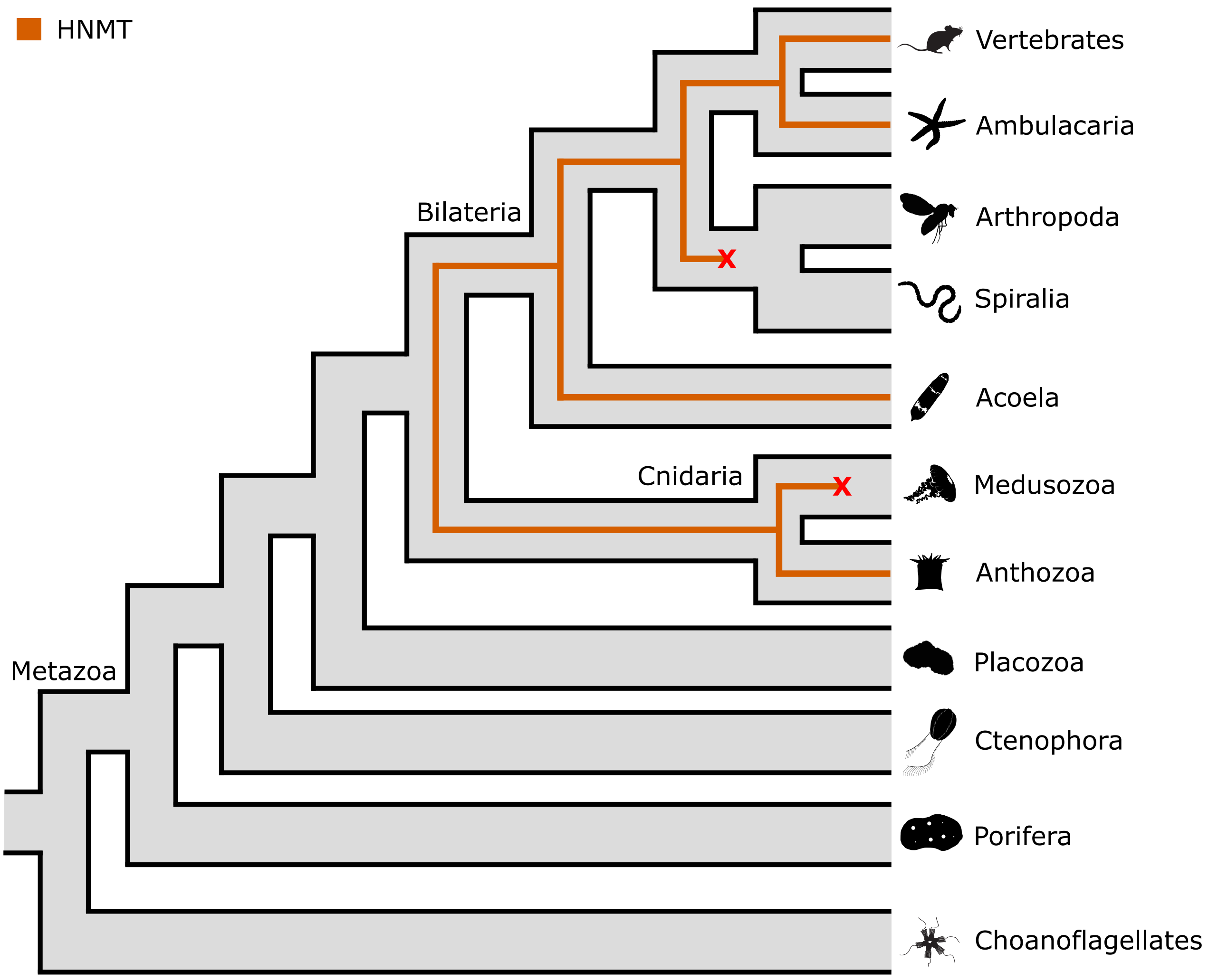
HNMT
Bilateria
Cnidaria
Metazoa
Vertebrates
Ambulacaria
Arthropoda
Spiralia
Acoela
Medusozoa
Anthozoa
Placozoa
Ctenophora
Porifera
Choanoflagellates

Supplementary Figure 12: Analysis of HNMTs. A) TBE tree with TBE score supports. B) Maximum likelihood tree with ultrafast bootstrap proportion supports. C) Maximum likelihood tree including all eukaryotic sequences with ultrafast bootstrap proportion supports. D) Reconciliation using sponge-first hypothesis. E) Reconciliation using ctenophore-first hypothesis. F) Reconciliation of the UFB tree including all eukaryotic sequences. Nodes in reconciled trees indicated as speciation events (S) or duplication events (D). All trees shown as rooted, non-reconciled trees rooted arbitrarily for visualisation. HNMT = histamine-N-methyltransferase, TBE = transfer bootstrap expectation, UFB = ultrafast bootstrap. Branches colour coded by sequence annotation or clade; orange = histamine-N-methyltransferase. Source Data are available at [https://figshare.le.ac.uk/articles/dataset/Monoamine_Neuromodulation_is_a_Bilaterian_Innovation_Results/20391477]; in Results/GeneTrees labelled OG_HNMT_TBE.tbe.tree and OG_HNMT_UFB.treefile; in Results/GeneTrees/Eukaryotes_GeneTrees labelled OG_HNMT_EUKIQ.treefile; in Results/Reconciliation/SpongeFirst labelled OG_HNMT; in Results/Reconciliation/CtenophoreFirst labelled OG_HNMT; and in Results/Reconciliation/Eukaryote labelled OG_HNMT.

12A.

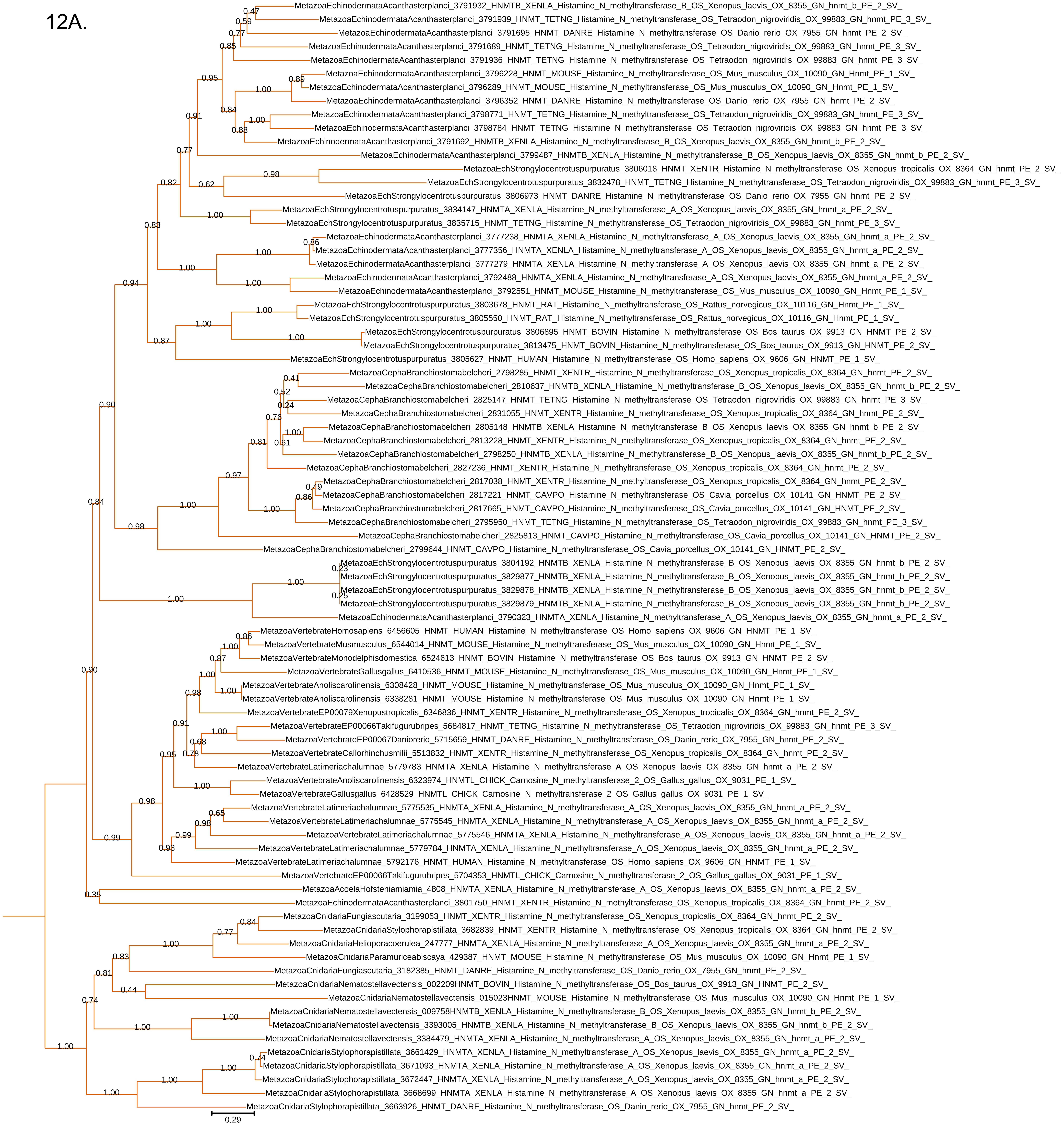
MetazoaEchinodermataAcanthasterplanci_3791932_HNMTB_XENLA_Histamine_N_methyltransferase_B_OS_Xenopus_laevis_OX_8355_GN_hnmt_b_PE_2_SV_
MetazoaEchinodermataAcanthasterplanci_3791939_HNMT_TETNG_Histamine_N_methyltransferase_OS_Tetraodon_nigroviridis_OX_99883_GN_hnmt_PE_3_SV_
MetazoaEchinodermataAcanthasterplanci_3791695_HNMT_DANRE_Histamine_N_methyltransferase_OS_Danio_rerio_OX_7955_GN_hnmt_PE_2_SV_
MetazoaEchinodermataAcanthasterplanci_3791689_HNMT_TETNG_Histamine_N_methyltransferase_OS_Tetraodon_nigroviridis_OX_99883_GN_hnmt_PE_3_SV_
MetazoaEchinodermataAcanthasterplanci_3791936_HNMT_TETNG_Histamine_N_methyltransferase_OS_Tetraodon_nigroviridis_OX_99883_GN_hnmt_PE_3_SV_
MetazoaEchinodermataAcanthasterplanci_3796228_HNMT_MOUSE_Histamine_N_methyltransferase_OS_Mus_musculus_OX_10090_GN_Hnmt_PE_1_SV_
MetazoaEchinodermataAcanthasterplanci_3796289_HNMT_MOUSE_Histamine_N_methyltransferase_OS_Mus_musculus_OX_10090_GN_Hnmt_PE_1_SV_
MetazoaEchinodermataAcanthasterplanci_3796352_HNMT_DANRE_Histamine_N_methyltransferase_OS_Danio_rerio_OX_7955_GN_hnmt_PE_2_SV_
MetazoaEchinodermataAcanthasterplanci_3798771_HNMT_TETNG_Histamine_N_methyltransferase_OS_Tetraodon_nigroviridis_OX_99883_GN_hnmt_PE_3_SV_
MetazoaEchinodermataAcanthasterplanci_3798784_HNMT_TETNG_Histamine_N_methyltransferase_OS_Tetraodon_nigroviridis_OX_99883_GN_hnmt_PE_3_SV_
MetazoaEchinodermataAcanthasterplanci_3791692_HNMTB_XENLA_Histamine_N_methyltransferase_B_OS_Xenopus_laevis_OX_8355_GN_hnmt_b_PE_2_SV_
MetazoaEchinodermataAcanthasterplanci_3799487_HNMTB_XENLA_Histamine_N_methyltransferase_B_OS_Xenopus_laevis_OX_8355_GN_hnmt_b_PE_2_SV_
MetazoaEchStrongylocentrotuspurpuratus_3806018_HNMT_XENTR_Histamine_N_methyltransferase_OS_Xenopus_tropicalis_OX_8364_GN_hnmt_PE_2_SV_
MetazoaEchStrongylocentrotuspurpuratus_3832478_HNMT_TETNG_Histamine_N_methyltransferase_OS_Tetraodon_nigroviridis_OX_99883_GN_hnmt_PE_3_SV_
MetazoaEchStrongylocentrotuspurpuratus_3806973_HNMT_DANRE_Histamine_N_methyltransferase_OS_Danio_rerio_OX_7955_GN_hnmt_PE_2_SV_
MetazoaEchStrongylocentrotuspurpuratus_3834147_HNMTA_XENLA_Histamine_N_methyltransferase_A_OS_Xenopus_laevis_OX_8355_GN_hnmt_a_PE_2_SV_
MetazoaEchStrongylocentrotuspurpuratus_3835715_HNMT_TETNG_Histamine_N_methyltransferase_OS_Tetraodon_nigroviridis_OX_99883_GN_hnmt_PE_3_SV_
MetazoaEchinodermataAcanthasterplanci_3777238_HNMTA_XENLA_Histamine_N_methyltransferase_A_OS_Xenopus_laevis_OX_8355_GN_hnmt_a_PE_2_SV_
MetazoaEchinodermataAcanthasterplanci_3777356_HNMTA_XENLA_Histamine_N_methyltransferase_A_OS_Xenopus_laevis_OX_8355_GN_hnmt_a_PE_2_SV_
MetazoaEchinodermataAcanthasterplanci_3777279_HNMTA_XENLA_Histamine_N_methyltransferase_A_OS_Xenopus_laevis_OX_8355_GN_hnmt_a_PE_2_SV_
MetazoaEchinodermataAcanthasterplanci_3792488_HNMTA_XENLA_Histamine_N_methyltransferase_A_OS_Xenopus_laevis_OX_8355_GN_hnmt_a_PE_2_SV_
MetazoaEchinodermataAcanthasterplanci_3792551_HNMT_MOUSE_Histamine_N_methyltransferase_OS_Mus_musculus_OX_10090_GN_Hnmt_PE_1_SV_
MetazoaEchStrongylocentrotuspurpuratus_3803678_HNMT_RAT_Histamine_N_methyltransferase_OS_Rattus_norvegicus_OX_10116_GN_Hnmt_PE_1_SV_
MetazoaEchStrongylocentrotuspurpuratus_3805550_HNMT_RAT_Histamine_N_methyltransferase_OS_Rattus_norvegicus_OX_10116_GN_Hnmt_PE_1_SV_
MetazoaEchStrongylocentrotuspurpuratus_3806895_HNMT_BOVIN_Histamine_N_methyltransferase_OS_Bos_taurus_OX_9913_GN_HNMT_PE_2_SV_
MetazoaEchStrongylocentrotuspurpuratus_3813475_HNMT_BOVIN_Histamine_N_methyltransferase_OS_Bos_taurus_OX_9913_GN_HNMT_PE_2_SV_
MetazoaEchStrongylocentrotuspurpuratus_3805627_HNMT_HUMAN_Histamine_N_methyltransferase_OS_Homo_sapiens_OX_9606_GN_HNMT_PE_1_SV_
MetazoaCephaBranchiostomabelcheri_2798285_HNMT_XENTR_Histamine_N_methyltransferase_OS_Xenopus_tropicalis_OX_8364_GN_hnmt_PE_2_SV_
MetazoaCephaBranchiostomabelcheri_2810637_HNMTB_XENLA_Histamine_N_methyltransferase_B_OS_Xenopus_laevis_OX_8355_GN_hnmt_b_PE_2_SV_
MetazoaCephaBranchiostomabelcheri_2825147_HNMT_TETNG_Histamine_N_methyltransferase_OS_Tetraodon_nigroviridis_OX_99883_GN_hnmt_PE_3_SV_
MetazoaCephaBranchiostomabelcheri_2831055_HNMT_XENTR_Histamine_N_methyltransferase_OS_Xenopus_tropicalis_OX_8364_GN_hnmt_PE_2_SV_
MetazoaCephaBranchiostomabelcheri_2805148_HNMTB_XENLA_Histamine_N_methyltransferase_B_OS_Xenopus_laevis_OX_8355_GN_hnmt_b_PE_2_SV_
MetazoaCephaBranchiostomabelcheri_2813228_HNMT_XENTR_Histamine_N_methyltransferase_OS_Xenopus_tropicalis_OX_8364_GN_hnmt_PE_2_SV_
MetazoaCephaBranchiostomabelcheri_2798250_HNMTB_XENLA_Histamine_N_methyltransferase_B_OS_Xenopus_laevis_OX_8355_GN_hnmt_b_PE_2_SV_
MetazoaCephaBranchiostomabelcheri_2827236_HNMT_XENTR_Histamine_N_methyltransferase_OS_Xenopus_tropicalis_OX_8364_GN_hnmt_PE_2_SV_
MetazoaCephaBranchiostomabelcheri_2817038_HNMT_XENTR_Histamine_N_methyltransferase_OS_Xenopus_tropicalis_OX_8364_GN_hnmt_PE_2_SV_
MetazoaCephaBranchiostomabelcheri_2817221_HNMT_CAVPO_Histamine_N_methyltransferase_OS_Cavia_porcellus_OX_10141_GN_HNMT_PE_2_SV_
MetazoaCephaBranchiostomabelcheri_2817665_HNMT_CAVPO_Histamine_N_methyltransferase_OS_Cavia_porcellus_OX_10141_GN_HNMT_PE_2_SV_
MetazoaCephaBranchiostomabelcheri_2795950_HNMT_TETNG_Histamine_N_methyltransferase_OS_Tetraodon_nigroviridis_OX_99883_GN_hnmt_PE_3_SV_
MetazoaCephaBranchiostomabelcheri_2825813_HNMT_CAVPO_Histamine_N_methyltransferase_OS_Cavia_porcellus_OX_10141_GN_HNMT_PE_2_SV_
MetazoaCephaBranchiostomabelcheri_2799644_HNMT_CAVPO_Histamine_N_methyltransferase_OS_Cavia_porcellus_OX_10141_GN_HNMT_PE_2_SV_
MetazoaEchStrongylocentrotuspurpuratus_3804192_HNMTB_XENLA_Histamine_N_methyltransferase_B_OS_Xenopus_laevis_OX_8355_GN_hnmt_b_PE_2_SV_
MetazoaEchStrongylocentrotuspurpuratus_3829877_HNMTB_XENLA_Histamine_N_methyltransferase_B_OS_Xenopus_laevis_OX_8355_GN_hnmt_b_PE_2_SV_
MetazoaEchStrongylocentrotuspurpuratus_3829878_HNMTB_XENLA_Histamine_N_methyltransferase_B_OS_Xenopus_laevis_OX_8355_GN_hnmt_b_PE_2_SV_
MetazoaEchStrongylocentrotuspurpuratus_3829879_HNMTB_XENLA_Histamine_N_methyltransferase_B_OS_Xenopus_laevis_OX_8355_GN_hnmt_b_PE_2_SV_
MetazoaEchinodermataAcanthasterplanci_3790323_HNMTA_XENLA_Histamine_N_methyltransferase_A_OS_Xenopus_laevis_OX_8355_GN_hnmt_a_PE_2_SV_
MetazoaVertebrateHomosapiens_6456605_HNMT_HUMAN_Histamine_N_methyltransferase_OS_Homo_sapiens_OX_9606_GN_HNMT_PE_1_SV_
MetazoaVertebrateMusmusculus_6544014_HNMT_MOUSE_Histamine_N_methyltransferase_OS_Mus_musculus_OX_10090_GN_Hnmt_PE_1_SV_
MetazoaVertebrateMonodelphisdomestica_6524613_HNMT_BOVIN_Histamine_N_methyltransferase_OS_Bos_taurus_OX_9913_GN_HNMT_PE_2_SV_
MetazoaVertebrateGallusgallus_6410536_HNMT_MOUSE_Histamine_N_methyltransferase_OS_Mus_musculus_OX_10090_GN_Hnmt_PE_1_SV_
MetazoaVertebrateAnoliscarolinensis_6308428_HNMT_MOUSE_Histamine_N_methyltransferase_OS_Mus_musculus_OX_10090_GN_Hnmt_PE_1_SV_
MetazoaVertebrateAnoliscarolinensis_6338281_HNMT_MOUSE_Histamine_N_methyltransferase_OS_Mus_musculus_OX_10090_GN_Hnmt_PE_1_SV_
MetazoaVertebrateEP00079Xenopustropicalis_6346836_HNMT_XENTR_Histamine_N_methyltransferase_OS_Xenopus_tropicalis_OX_8364_GN_hnmt_PE_2_SV_
MetazoaVertebrateEP00066Takifugurubripes_5684817_HNMT_TETNG_Histamine_N_methyltransferase_OS_Tetraodon_nigroviridis_OX_99883_GN_hnmt_PE_3_SV_
MetazoaVertebrateEP00067Daniorerio_5715659_HNMT_DANRE_Histamine_N_methyltransferase_OS_Danio_rerio_OX_7955_GN_hnmt_PE_2_SV_
MetazoaVertebrateCallorhinchusmilii_5513832_HNMT_XENTR_Histamine_N_methyltransferase_OS_Xenopus_tropicalis_OX_8364_GN_hnmt_PE_2_SV_
MetazoaVertebrateLatimeriachalumnae_5779783_HNMTA_XENLA_Histamine_N_methyltransferase_A_OS_Xenopus_laevis_OX_8355_GN_hnmt_a_PE_2_SV_
MetazoaVertebrateAnoliscarolinensis_6323974_HNMTL_CHICK_Carnosine_N_methyltransferase_2_OS_Gallus_gallus_OX_9031_PE_1_SV_
MetazoaVertebrateGallusgallus_6428529_HNMTL_CHICK_Carnosine_N_methyltransferase_2_OS_Gallus_gallus_OX_9031_PE_1_SV_
MetazoaVertebrateLatimeriachalumnae_5775535_HNMTA_XENLA_Histamine_N_methyltransferase_A_OS_Xenopus_laevis_OX_8355_GN_hnmt_a_PE_2_SV_
MetazoaVertebrateLatimeriachalumnae_5775545_HNMTA_XENLA_Histamine_N_methyltransferase_A_OS_Xenopus_laevis_OX_8355_GN_hnmt_a_PE_2_SV_
MetazoaVertebrateLatimeriachalumnae_5775546_HNMTA_XENLA_Histamine_N_methyltransferase_A_OS_Xenopus_laevis_OX_8355_GN_hnmt_a_PE_2_SV_
MetazoaVertebrateLatimeriachalumnae_5779784_HNMTA_XENLA_Histamine_N_methyltransferase_A_OS_Xenopus_laevis_OX_8355_GN_hnmt_a_PE_2_SV_
MetazoaVertebrateLatimeriachalumnae_5792176_HNMT_HUMAN_Histamine_N_methyltransferase_OS_Homo_sapiens_OX_9606_GN_HNMT_PE_1_SV_
MetazoaVertebrateEP00066Takifugurubripes_5704353_HNMTL_CHICK_Carnosine_N_methyltransferase_2_OS_Gallus_gallus_OX_9031_PE_1_SV_
MetazoaAcoelaHofsteniamiamia_4808_HNMTA_XENLA_Histamine_N_methyltransferase_A_OS_Xenopus_laevis_OX_8355_GN_hnmt_a_PE_2_SV_
MetazoaEchinodermataAcanthasterplanci_3801750_HNMT_XENTR_Histamine_N_methyltransferase_OS_Xenopus_tropicalis_OX_8364_GN_hnmt_PE_2_SV_
MetazoaCnidariaFungiascutaria_3199053_HNMT_XENTR_Histamine_N_methyltransferase_OS_Xenopus_tropicalis_OX_8364_GN_hnmt_PE_2_SV_
MetazoaCnidariaStylophorapistillata_3682839_HNMT_XENTR_Histamine_N_methyltransferase_OS_Xenopus_tropicalis_OX_8364_GN_hnmt_PE_2_SV_
MetazoaCnidariaHelioporacoerulea_247777_HNMTA_XENLA_Histamine_N_methyltransferase_A_OS_Xenopus_laevis_OX_8355_GN_hnmt_a_PE_2_SV_
MetazoaCnidariaParamuriceabiscaya_429387_HNMT_MOUSE_Histamine_N_methyltransferase_OS_Mus_musculus_OX_10090_GN_Hnmt_PE_1_SV_
MetazoaCnidariaFungiascutaria_3182385_HNMT_DANRE_Histamine_N_methyltransferase_OS_Danio_rerio_OX_7955_GN_hnmt_PE_2_SV_
MetazoaCnidariaNematostellavectensis_002209HNMT_BOVIN_Histamine_N_methyltransferase_OS_Bos_taurus_OX_9913_GN_HNMT_PE_2_SV_
MetazoaCnidariaNematostellavectensis_015023HNMT_MOUSE_Histamine_N_methyltransferase_OS_Mus_musculus_OX_10090_GN_Hnmt_PE_1_SV_
MetazoaCnidariaNematostellavectensis_009758HNMTB_XENLA_Histamine_N_methyltransferase_B_OS_Xenopus_laevis_OX_8355_GN_hnmt_b_PE_2_SV_
MetazoaCnidariaNematostellavectensis_3393005_HNMTB_XENLA_Histamine_N_methyltransferase_B_OS_Xenopus_laevis_OX_8355_GN_hnmt_b_PE_2_SV_
MetazoaCnidariaNematostellavectensis_3384479_HNMTA_XENLA_Histamine_N_methyltransferase_A_OS_Xenopus_laevis_OX_8355_GN_hnmt_a_PE_2_SV_
MetazoaCnidariaStylophorapistillata_3661429_HNMTA_XENLA_Histamine_N_methyltransferase_A_OS_Xenopus_laevis_OX_8355_GN_hnmt_a_PE_2_SV_
MetazoaCnidariaStylophorapistillata_3671093_HNMTA_XENLA_Histamine_N_methyltransferase_A_OS_Xenopus_laevis_OX_8355_GN_hnmt_a_PE_2_SV_
MetazoaCnidariaStylophorapistillata_3672447_HNMTA_XENLA_Histamine_N_methyltransferase_A_OS_Xenopus_laevis_OX_8355_GN_hnmt_a_PE_2_SV_
MetazoaCnidariaStylophorapistillata_3668699_HNMTA_XENLA_Histamine_N_methyltransferase_A_OS_Xenopus_laevis_OX_8355_GN_hnmt_a_PE_2_SV_
MetazoaCnidariaStylophorapistillata_3663926_HNMT_DANRE_Histamine_N_methyltransferase_OS_Danio_rerio_OX_7955_GN_hnmt_PE_2_SV_
0.29

12B.

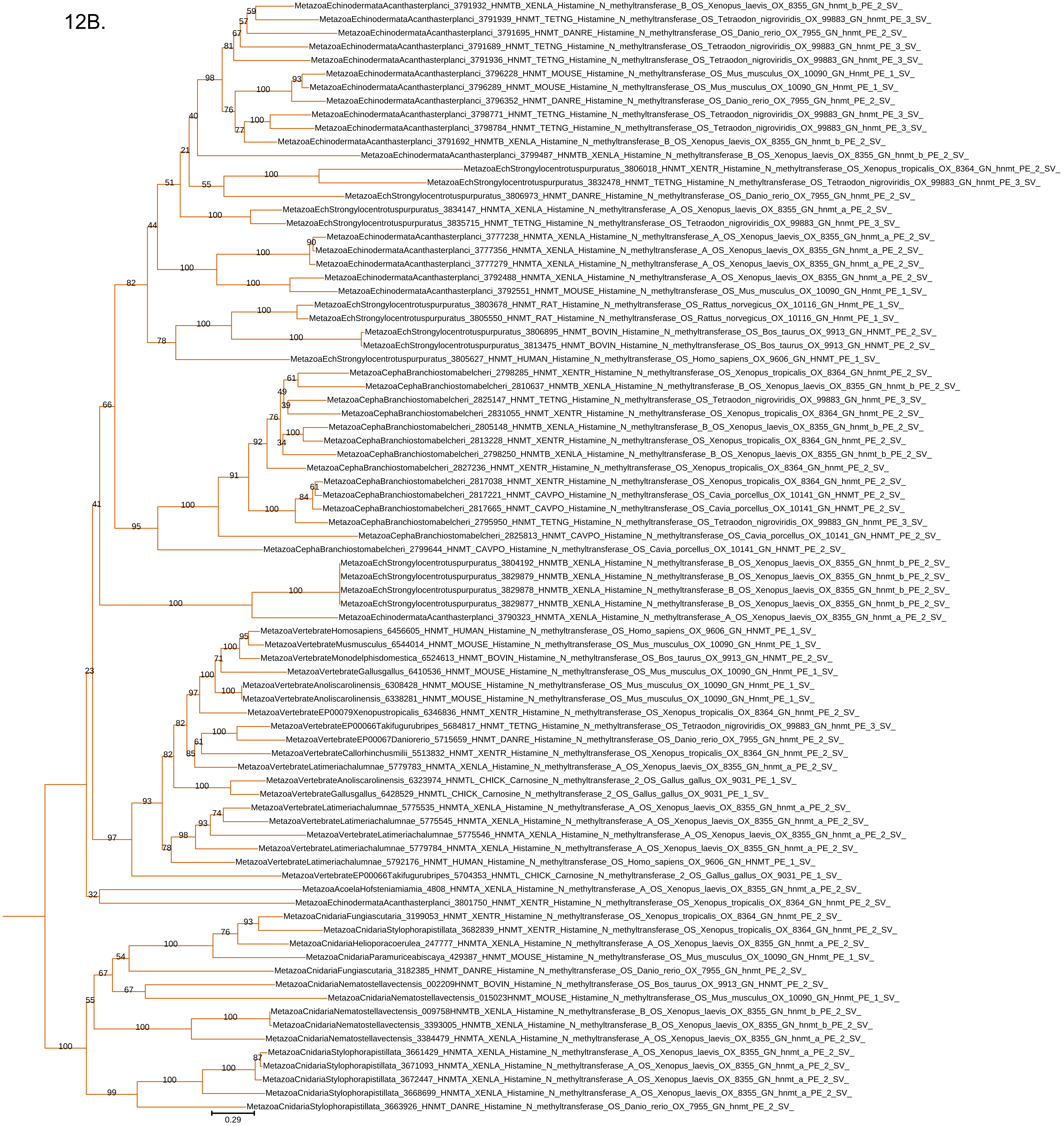
MetazoaEchinodermataAcanthasterplanci_3791932_HNMTB_XENLA_Histamine_N_methyltransferase_B_OS_Xenopus_laevis_OX_8355_GN_hnmt_b_PE_2_SV_
MetazoaEchinodermataAcanthasterplanci_3791939_HNMT_TETNG_Histamine_N_methyltransferase_OS_Tetraodon_nigroviridis_OX_99883_GN_hnmt_PE_3_SV_
MetazoaEchinodermataAcanthasterplanci_3791695_HNMT_DANRE_Histamine_N_methyltransferase_OS_Danio_rerio_OX_7955_GN_hnmt_PE_2_SV_
MetazoaEchinodermataAcanthasterplanci_3791689_HNMT_TETNG_Histamine_N_methyltransferase_OS_Tetraodon_nigroviridis_OX_99883_GN_hnmt_PE_3_SV_
MetazoaEchinodermataAcanthasterplanci_3791936_HNMT_TETNG_Histamine_N_methyltransferase_OS_Tetraodon_nigroviridis_OX_99883_GN_hnmt_PE_3_SV_
MetazoaEchinodermataAcanthasterplanci_3796228_HNMT_MOUSE_Histamine_N_methyltransferase_OS_Mus_musculus_OX_10090_GN_Hnmt_PE_1_SV_
MetazoaEchinodermataAcanthasterplanci_3796289_HNMT_MOUSE_Histamine_N_methyltransferase_OS_Mus_musculus_OX_10090_GN_Hnmt_PE_1_SV_
MetazoaEchinodermataAcanthasterplanci_3796352_HNMT_DANRE_Histamine_N_methyltransferase_OS_Danio_rerio_OX_7955_GN_hnmt_PE_2_SV_
MetazoaEchinodermataAcanthasterplanci_3798771_HNMT_TETNG_Histamine_N_methyltransferase_OS_Tetraodon_nigroviridis_OX_99883_GN_hnmt_PE_3_SV_
MetazoaEchinodermataAcanthasterplanci_3798784_HNMT_TETNG_Histamine_N_methyltransferase_OS_Tetraodon_nigroviridis_OX_99883_GN_hnmt_PE_3_SV_
MetazoaEchinodermataAcanthasterplanci_3791692_HNMTB_XENLA_Histamine_N_methyltransferase_B_OS_Xenopus_laevis_OX_8355_GN_hnmt_b_PE_2_SV_
MetazoaEchinodermataAcanthasterplanci_3799487_HNMTB_XENLA_Histamine_N_methyltransferase_B_OS_Xenopus_laevis_OX_8355_GN_hnmt_b_PE_2_SV_
MetazoaEchStrongylocentrotuspurpuratus_3806018_HNMT_XENTR_Histamine_N_methyltransferase_OS_Xenopus_tropicalis_OX_8364_GN_hnmt_PE_2_SV_
MetazoaEchStrongylocentrotuspurpuratus_3832478_HNMT_TETNG_Histamine_N_methyltransferase_OS_Tetraodon_nigroviridis_OX_99883_GN_hnmt_PE_3_SV_
MetazoaEchStrongylocentrotuspurpuratus_3806973_HNMT_DANRE_Histamine_N_methyltransferase_OS_Danio_rerio_OX_7955_GN_hnmt_PE_2_SV_
MetazoaEchStrongylocentrotuspurpuratus_3834147_HNMTA_XENLA_Histamine_N_methyltransferase_A_OS_Xenopus_laevis_OX_8355_GN_hnmt_a_PE_2_SV_
MetazoaEchStrongylocentrotuspurpuratus_3835715_HNMT_TETNG_Histamine_N_methyltransferase_OS_Tetraodon_nigroviridis_OX_99883_GN_hnmt_PE_3_SV_
MetazoaEchinodermataAcanthasterplanci_3777238_HNMTA_XENLA_Histamine_N_methyltransferase_A_OS_Xenopus_laevis_OX_8355_GN_hnmt_a_PE_2_SV_
MetazoaEchinodermataAcanthasterplanci_3777356_HNMTA_XENLA_Histamine_N_methyltransferase_A_OS_Xenopus_laevis_OX_8355_GN_hnmt_a_PE_2_SV_
MetazoaEchinodermataAcanthasterplanci_3777279_HNMTA_XENLA_Histamine_N_methyltransferase_A_OS_Xenopus_laevis_OX_8355_GN_hnmt_a_PE_2_SV_
MetazoaEchinodermataAcanthasterplanci_3792488_HNMTA_XENLA_Histamine_N_methyltransferase_A_OS_Xenopus_laevis_OX_8355_GN_hnmt_a_PE_2_SV_
MetazoaEchinodermataAcanthasterplanci_3792551_HNMT_MOUSE_Histamine_N_methyltransferase_OS_Mus_musculus_OX_10090_GN_Hnmt_PE_1_SV_
MetazoaEchStrongylocentrotuspurpuratus_3803678_HNMT_RAT_Histamine_N_methyltransferase_OS_Rattus_norvegicus_OX_10116_GN_Hnmt_PE_1_SV_
MetazoaEchStrongylocentrotuspurpuratus_3805550_HNMT_RAT_Histamine_N_methyltransferase_OS_Rattus_norvegicus_OX_10116_GN_Hnmt_PE_1_SV_
MetazoaEchStrongylocentrotuspurpuratus_3806895_HNMT_BOVIN_Histamine_N_methyltransferase_OS_Bos_taurus_OX_9913_GN_HNMT_PE_2_SV_
MetazoaEchStrongylocentrotuspurpuratus_3813475_HNMT_BOVIN_Histamine_N_methyltransferase_OS_Bos_taurus_OX_9913_GN_HNMT_PE_2_SV_
MetazoaEchStrongylocentrotuspurpuratus_3805627_HNMT_HUMAN_Histamine_N_methyltransferase_OS_Homo_sapiens_OX_9606_GN_HNMT_PE_1_SV_
MetazoaCephaBranchiostomabelcheri_2798285_HNMT_XENTR_Histamine_N_methyltransferase_OS_Xenopus_tropicalis_OX_8364_GN_hnmt_PE_2_SV_
MetazoaCephaBranchiostomabelcheri_2810637_HNMTB_XENLA_Histamine_N_methyltransferase_B_OS_Xenopus_laevis_OX_8355_GN_hnmt_b_PE_2_SV_
MetazoaCephaBranchiostomabelcheri_2825147_HNMT_TETNG_Histamine_N_methyltransferase_OS_Tetraodon_nigroviridis_OX_99883_GN_hnmt_PE_3_SV_
MetazoaCephaBranchiostomabelcheri_2831055_HNMT_XENTR_Histamine_N_methyltransferase_OS_Xenopus_tropicalis_OX_8364_GN_hnmt_PE_2_SV_
MetazoaCephaBranchiostomabelcheri_2805148_HNMTB_XENLA_Histamine_N_methyltransferase_B_OS_Xenopus_laevis_OX_8355_GN_hnmt_b_PE_2_SV_
MetazoaCephaBranchiostomabelcheri_2813228_HNMT_XENTR_Histamine_N_methyltransferase_OS_Xenopus_tropicalis_OX_8364_GN_hnmt_PE_2_SV_
MetazoaCephaBranchiostomabelcheri_2798250_HNMTB_XENLA_Histamine_N_methyltransferase_B_OS_Xenopus_laevis_OX_8355_GN_hnmt_b_PE_2_SV_
MetazoaCephaBranchiostomabelcheri_2827236_HNMT_XENTR_Histamine_N_methyltransferase_OS_Xenopus_tropicalis_OX_8364_GN_hnmt_PE_2_SV_
MetazoaCephaBranchiostomabelcheri_2817038_HNMT_XENTR_Histamine_N_methyltransferase_OS_Xenopus_tropicalis_OX_8364_GN_hnmt_PE_2_SV_
MetazoaCephaBranchiostomabelcheri_2817221_HNMT_CAVPO_Histamine_N_methyltransferase_OS_Cavia_porcellus_OX_10141_GN_HNMT_PE_2_SV_
MetazoaCephaBranchiostomabelcheri_2817665_HNMT_CAVPO_Histamine_N_methyltransferase_OS_Cavia_porcellus_OX_10141_GN_HNMT_PE_2_SV_
MetazoaCephaBranchiostomabelcheri_2795950_HNMT_TETNG_Histamine_N_methyltransferase_OS_Tetraodon_nigroviridis_OX_99883_GN_hnmt_PE_3_SV_
MetazoaCephaBranchiostomabelcheri_2825813_HNMT_CAVPO_Histamine_N_methyltransferase_OS_Cavia_porcellus_OX_10141_GN_HNMT_PE_2_SV_
MetazoaCephaBranchiostomabelcheri_2799644_HNMT_CAVPO_Histamine_N_methyltransferase_OS_Cavia_porcellus_OX_10141_GN_HNMT_PE_2_SV_
MetazoaEchStrongylocentrotuspurpuratus_3804192_HNMTB_XENLA_Histamine_N_methyltransferase_B_OS_Xenopus_laevis_OX_8355_GN_hnmt_b_PE_2_SV_
MetazoaEchStrongylocentrotuspurpuratus_3829879_HNMTB_XENLA_Histamine_N_methyltransferase_B_OS_Xenopus_laevis_OX_8355_GN_hnmt_b_PE_2_SV_
MetazoaEchStrongylocentrotuspurpuratus_3829878_HNMTB_XENLA_Histamine_N_methyltransferase_B_OS_Xenopus_laevis_OX_8355_GN_hnmt_b_PE_2_SV_
MetazoaEchStrongylocentrotuspurpuratus_3829877_HNMTB_XENLA_Histamine_N_methyltransferase_B_OS_Xenopus_laevis_OX_8355_GN_hnmt_b_PE_2_SV_
MetazoaEchinodermataAcanthasterplanci_3790323_HNMTA_XENLA_Histamine_N_methyltransferase_A_OS_Xenopus_laevis_OX_8355_GN_hnmt_a_PE_2_SV_
MetazoaVertebrateHomosapiens_6456605_HNMT_HUMAN_Histamine_N_methyltransferase_OS_Homo_sapiens_OX_9606_GN_HNMT_PE_1_SV_
MetazoaVertebrateMusmusculus_6544014_HNMT_MOUSE_Histamine_N_methyltransferase_OS_Mus_musculus_OX_10090_GN_Hnmt_PE_1_SV_
MetazoaVertebrateMonodelphisdomestica_6524613_HNMT_BOVIN_Histamine_N_methyltransferase_OS_Bos_taurus_OX_9913_GN_HNMT_PE_2_SV_
MetazoaVertebrateGallusgallus_6410536_HNMT_MOUSE_Histamine_N_methyltransferase_OS_Mus_musculus_OX_10090_GN_Hnmt_PE_1_SV_
MetazoaVertebrateAnoliscarolinensis_6308428_HNMT_MOUSE_Histamine_N_methyltransferase_OS_Mus_musculus_OX_10090_GN_Hnmt_PE_1_SV_
MetazoaVertebrateAnoliscarolinensis_6338281_HNMT_MOUSE_Histamine_N_methyltransferase_OS_Mus_musculus_OX_10090_GN_Hnmt_PE_1_SV_
MetazoaVertebrateEP00079Xenopustropicalis_6346836_HNMT_XENTR_Histamine_N_methyltransferase_OS_Xenopus_tropicalis_OX_8364_GN_hnmt_PE_2_SV_
MetazoaVertebrateEP00066Takifugurubripes_5684817_HNMT_TETNG_Histamine_N_methyltransferase_OS_Tetraodon_nigroviridis_OX_99883_GN_hnmt_PE_3_SV_
MetazoaVertebrateEP00067Daniorerio_5715659_HNMT_DANRE_Histamine_N_methyltransferase_OS_Danio_rerio_OX_7955_GN_hnmt_PE_2_SV_
MetazoaVertebrateCallorhinchusmilii_5513832_HNMT_XENTR_Histamine_N_methyltransferase_OS_Xenopus_tropicalis_OX_8364_GN_hnmt_PE_2_SV_
MetazoaVertebrateLatimeriachalumnae_5779783_HNMTA_XENLA_Histamine_N_methyltransferase_A_OS_Xenopus_laevis_OX_8355_GN_hnmt_a_PE_2_SV_
MetazoaVertebrateAnoliscarolinensis_6323974_HNMTL_CHICK_Carnosine_N_methyltransferase_2_OS_Gallus_gallus_OX_9031_PE_1_SV_
MetazoaVertebrateGallusgallus_6428529_HNMTL_CHICK_Carnosine_N_methyltransferase_2_OS_Gallus_gallus_OX_9031_PE_1_SV_
MetazoaVertebrateLatimeriachalumnae_5775535_HNMTA_XENLA_Histamine_N_methyltransferase_A_OS_Xenopus_laevis_OX_8355_GN_hnmt_a_PE_2_SV_
MetazoaVertebrateLatimeriachalumnae_5775545_HNMTA_XENLA_Histamine_N_methyltransferase_A_OS_Xenopus_laevis_OX_8355_GN_hnmt_a_PE_2_SV_
MetazoaVertebrateLatimeriachalumnae_5775546_HNMTA_XENLA_Histamine_N_methyltransferase_A_OS_Xenopus_laevis_OX_8355_GN_hnmt_a_PE_2_SV_
MetazoaVertebrateLatimeriachalumnae_5779784_HNMTA_XENLA_Histamine_N_methyltransferase_A_OS_Xenopus_laevis_OX_8355_GN_hnmt_a_PE_2_SV_
MetazoaVertebrateLatimeriachalumnae_5792176_HNMT_HUMAN_Histamine_N_methyltransferase_OS_Homo_sapiens_OX_9606_GN_HNMT_PE_1_SV_
MetazoaVertebrateEP00066Takifugurubripes_5704353_HNMTL_CHICK_Carnosine_N_methyltransferase_2_OS_Gallus_gallus_OX_9031_PE_1_SV_
MetazoaAcoelaHofsteniamiamia_4808_HNMTA_XENLA_Histamine_N_methyltransferase_A_OS_Xenopus_laevis_OX_8355_GN_hnmt_a_PE_2_SV_
MetazoaEchinodermataAcanthasterplanci_3801750_HNMT_XENTR_Histamine_N_methyltransferase_OS_Xenopus_tropicalis_OX_8364_GN_hnmt_PE_2_SV_
MetazoaCnidariaFungiascutaria_3199053_HNMT_XENTR_Histamine_N_methyltransferase_OS_Xenopus_tropicalis_OX_8364_GN_hnmt_PE_2_SV_
MetazoaCnidariaStylophorapistillata_3682839_HNMT_XENTR_Histamine_N_methyltransferase_OS_Xenopus_tropicalis_OX_8364_GN_hnmt_PE_2_SV_
MetazoaCnidariaHelioporacoerulea_247777_HNMTA_XENLA_Histamine_N_methyltransferase_A_OS_Xenopus_laevis_OX_8355_GN_hnmt_a_PE_2_SV_
MetazoaCnidariaParamuriceabiscaya_429387_HNMT_MOUSE_Histamine_N_methyltransferase_OS_Mus_musculus_OX_10090_GN_Hnmt_PE_1_SV_
MetazoaCnidariaFungiascutaria_3182385_HNMT_DANRE_Histamine_N_methyltransferase_OS_Danio_rerio_OX_7955_GN_hnmt_PE_2_SV_
MetazoaCnidariaNematostellavectensis_002209HNMT_BOVIN_Histamine_N_methyltransferase_OS_Bos_taurus_OX_9913_GN_HNMT_PE_2_SV_
MetazoaCnidariaNematostellavectensis_015023HNMT_MOUSE_Histamine_N_methyltransferase_OS_Mus_musculus_OX_10090_GN_Hnmt_PE_1_SV_
MetazoaCnidariaNematostellavectensis_009758HNMTB_XENLA_Histamine_N_methyltransferase_B_OS_Xenopus_laevis_OX_8355_GN_hnmt_b_PE_2_SV_
MetazoaCnidariaNematostellavectensis_3393005_HNMTB_XENLA_Histamine_N_methyltransferase_B_OS_Xenopus_laevis_OX_8355_GN_hnmt_b_PE_2_SV_
MetazoaCnidariaNematostellavectensis_3384479_HNMTA_XENLA_Histamine_N_methyltransferase_A_OS_Xenopus_laevis_OX_8355_GN_hnmt_a_PE_2_SV_
MetazoaCnidariaStylophorapistillata_3661429_HNMTA_XENLA_Histamine_N_methyltransferase_A_OS_Xenopus_laevis_OX_8355_GN_hnmt_a_PE_2_SV_
MetazoaCnidariaStylophorapistillata_3671093_HNMTA_XENLA_Histamine_N_methyltransferase_A_OS_Xenopus_laevis_OX_8355_GN_hnmt_a_PE_2_SV_
MetazoaCnidariaStylophorapistillata_3672447_HNMTA_XENLA_Histamine_N_methyltransferase_A_OS_Xenopus_laevis_OX_8355_GN_hnmt_a_PE_2_SV_
MetazoaCnidariaStylophorapistillata_3668699_HNMTA_XENLA_Histamine_N_methyltransferase_A_OS_Xenopus_laevis_OX_8355_GN_hnmt_a_PE_2_SV_
MetazoaCnidariaStylophorapistillata_3663926_HNMT_DANRE_Histamine_N_methyltransferase_OS_Danio_rerio_OX_7955_GN_hnmt_PE_2_SV_
0.29

12C.

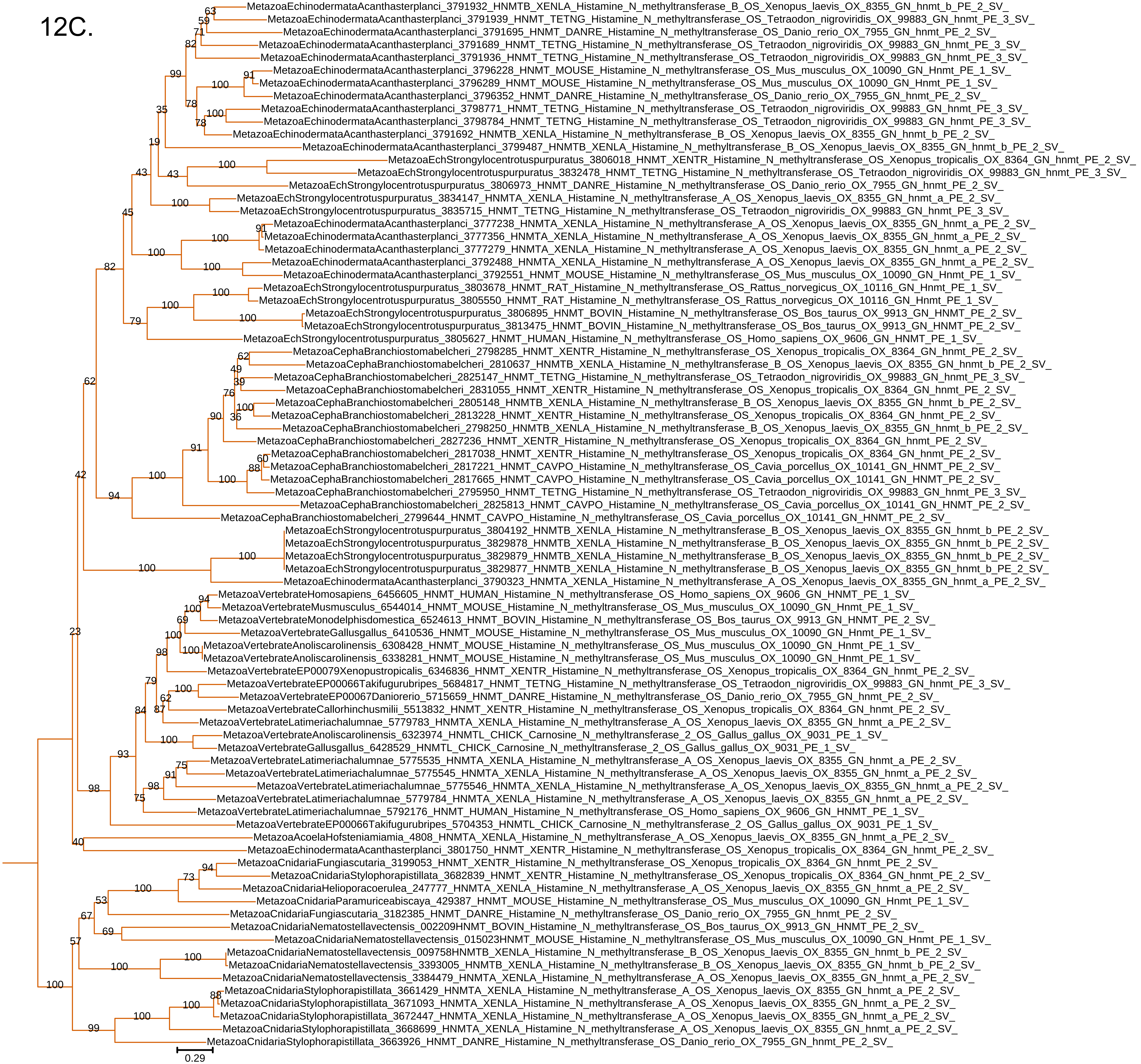
MetazoaEchinodermataAcanthasterplanci_3791932_HNMTB_XENLA_Histamine_N_methyltransferase_B_OS_Xenopus_laevis_OX_8355_GN_hnmt_b_PE_2_SV_
MetazoaEchinodermataAcanthasterplanci_3791939_HNMT_TETNG_Histamine_N_methyltransferase_OS_Tetraodon_nigroviridis_OX_99883_GN_hnmt_PE_3_SV_
MetazoaEchinodermataAcanthasterplanci_3791695_HNMT_DANRE_Histamine_N_methyltransferase_OS_Danio_rerio_OX_7955_GN_hnmt_PE_2_SV_
MetazoaEchinodermataAcanthasterplanci_3791689_HNMT_TETNG_Histamine_N_methyltransferase_OS_Tetraodon_nigroviridis_OX_99883_GN_hnmt_PE_3_SV_
MetazoaEchinodermataAcanthasterplanci_3791936_HNMT_TETNG_Histamine_N_methyltransferase_OS_Tetraodon_nigroviridis_OX_99883_GN_hnmt_PE_3_SV_
MetazoaEchinodermataAcanthasterplanci_3796228_HNMT_MOUSE_Histamine_N_methyltransferase_OS_Mus_musculus_OX_10090_GN_Hnmt_PE_1_SV_
MetazoaEchinodermataAcanthasterplanci_3796289_HNMT_MOUSE_Histamine_N_methyltransferase_OS_Mus_musculus_OX_10090_GN_Hnmt_PE_1_SV_
MetazoaEchinodermataAcanthasterplanci_3796352_HNMT_DANRE_Histamine_N_methyltransferase_OS_Danio_rerio_OX_7955_GN_hnmt_PE_2_SV_
MetazoaEchinodermataAcanthasterplanci_3798771_HNMT_TETNG_Histamine_N_methyltransferase_OS_Tetraodon_nigroviridis_OX_99883_GN_hnmt_PE_3_SV_
MetazoaEchinodermataAcanthasterplanci_3798784_HNMT_TETNG_Histamine_N_methyltransferase_OS_Tetraodon_nigroviridis_OX_99883_GN_hnmt_PE_3_SV_
MetazoaEchinodermataAcanthasterplanci_3791692_HNMTB_XENLA_Histamine_N_methyltransferase_B_OS_Xenopus_laevis_OX_8355_GN_hnmt_b_PE_2_SV_
MetazoaEchinodermataAcanthasterplanci_3799487_HNMTB_XENLA_Histamine_N_methyltransferase_B_OS_Xenopus_laevis_OX_8355_GN_hnmt_b_PE_2_SV_
MetazoaEchStrongylocentrotuspurpuratus_3806018_HNMT_XENTR_Histamine_N_methyltransferase_OS_Xenopus_tropicalis_OX_8364_GN_hnmt_PE_2_SV_
MetazoaEchStrongylocentrotuspurpuratus_3832478_HNMT_TETNG_Histamine_N_methyltransferase_OS_Tetraodon_nigroviridis_OX_99883_GN_hnmt_PE_3_SV_
MetazoaEchStrongylocentrotuspurpuratus_3806973_HNMT_DANRE_Histamine_N_methyltransferase_OS_Danio_rerio_OX_7955_GN_hnmt_PE_2_SV_
MetazoaEchStrongylocentrotuspurpuratus_3834147_HNMTA_XENLA_Histamine_N_methyltransferase_A_OS_Xenopus_laevis_OX_8355_GN_hnmt_a_PE_2_SV_
MetazoaEchStrongylocentrotuspurpuratus_3835715_HNMT_TETNG_Histamine_N_methyltransferase_OS_Tetraodon_nigroviridis_OX_99883_GN_hnmt_PE_3_SV_
MetazoaEchinodermataAcanthasterplanci_3777238_HNMTA_XENLA_Histamine_N_methyltransferase_A_OS_Xenopus_laevis_OX_8355_GN_hnmt_a_PE_2_SV_
MetazoaEchinodermataAcanthasterplanci_3777356_HNMTA_XENLA_Histamine_N_methyltransferase_A_OS_Xenopus_laevis_OX_8355_GN_hnmt_a_PE_2_SV_
MetazoaEchinodermataAcanthasterplanci_3777279_HNMTA_XENLA_Histamine_N_methyltransferase_A_OS_Xenopus_laevis_OX_8355_GN_hnmt_a_PE_2_SV_
MetazoaEchinodermataAcanthasterplanci_3792488_HNMTA_XENLA_Histamine_N_methyltransferase_A_OS_Xenopus_laevis_OX_8355_GN_hnmt_a_PE_2_SV_
MetazoaEchinodermataAcanthasterplanci_3792551_HNMT_MOUSE_Histamine_N_methyltransferase_OS_Mus_musculus_OX_10090_GN_Hnmt_PE_1_SV_
MetazoaEchStrongylocentrotuspurpuratus_3803678_HNMT_RAT_Histamine_N_methyltransferase_OS_Rattus_norvegicus_OX_10116_GN_Hnmt_PE_1_SV_
MetazoaEchStrongylocentrotuspurpuratus_3805550_HNMT_RAT_Histamine_N_methyltransferase_OS_Rattus_norvegicus_OX_10116_GN_Hnmt_PE_1_SV_
MetazoaEchStrongylocentrotuspurpuratus_3806895_HNMT_BOVIN_Histamine_N_methyltransferase_OS_Bos_taurus_OX_9913_GN_HNMT_PE_2_SV_
MetazoaEchStrongylocentrotuspurpuratus_3813475_HNMT_BOVIN_Histamine_N_methyltransferase_OS_Bos_taurus_OX_9913_GN_HNMT_PE_2_SV_
MetazoaEchStrongylocentrotuspurpuratus_3805627_HNMT_HUMAN_Histamine_N_methyltransferase_OS_Homo_sapiens_OX_9606_GN_HNMT_PE_1_SV_
MetazoaCephaBranchiostomabelcheri_2798285_HNMT_XENTR_Histamine_N_methyltransferase_OS_Xenopus_tropicalis_OX_8364_GN_hnmt_PE_2_SV_
MetazoaCephaBranchiostomabelcheri_2810637_HNMTB_XENLA_Histamine_N_methyltransferase_B_OS_Xenopus_laevis_OX_8355_GN_hnmt_b_PE_2_SV_
MetazoaCephaBranchiostomabelcheri_2825147_HNMT_TETNG_Histamine_N_methyltransferase_OS_Tetraodon_nigroviridis_OX_99883_GN_hnmt_PE_3_SV_
MetazoaCephaBranchiostomabelcheri_2831055_HNMT_XENTR_Histamine_N_methyltransferase_OS_Xenopus_tropicalis_OX_8364_GN_hnmt_PE_2_SV_
MetazoaCephaBranchiostomabelcheri_2805148_HNMTB_XENLA_Histamine_N_methyltransferase_B_OS_Xenopus_laevis_OX_8355_GN_hnmt_b_PE_2_SV_
MetazoaCephaBranchiostomabelcheri_2813228_HNMT_XENTR_Histamine_N_methyltransferase_OS_Xenopus_tropicalis_OX_8364_GN_hnmt_PE_2_SV_
MetazoaCephaBranchiostomabelcheri_2798250_HNMTB_XENLA_Histamine_N_methyltransferase_B_OS_Xenopus_laevis_OX_8355_GN_hnmt_b_PE_2_SV_
MetazoaCephaBranchiostomabelcheri_2827236_HNMT_XENTR_Histamine_N_methyltransferase_OS_Xenopus_tropicalis_OX_8364_GN_hnmt_PE_2_SV_
MetazoaCephaBranchiostomabelcheri_2817038_HNMT_XENTR_Histamine_N_methyltransferase_OS_Xenopus_tropicalis_OX_8364_GN_hnmt_PE_2_SV_
MetazoaCephaBranchiostomabelcheri_2817221_HNMT_CAVPO_Histamine_N_methyltransferase_OS_Cavia_porcellus_OX_10141_GN_HNMT_PE_2_SV_
MetazoaCephaBranchiostomabelcheri_2817665_HNMT_CAVPO_Histamine_N_methyltransferase_OS_Cavia_porcellus_OX_10141_GN_HNMT_PE_2_SV_
MetazoaCephaBranchiostomabelcheri_2795950_HNMT_TETNG_Histamine_N_methyltransferase_OS_Tetraodon_nigroviridis_OX_99883_GN_hnmt_PE_3_SV_
MetazoaCephaBranchiostomabelcheri_2825813_HNMT_CAVPO_Histamine_N_methyltransferase_OS_Cavia_porcellus_OX_10141_GN_HNMT_PE_2_SV_
MetazoaCephaBranchiostomabelcheri_2799644_HNMT_CAVPO_Histamine_N_methyltransferase_OS_Cavia_porcellus_OX_10141_GN_HNMT_PE_2_SV_
MetazoaEchStrongylocentrotuspurpuratus_3804192_HNMTB_XENLA_Histamine_N_methyltransferase_B_OS_Xenopus_laevis_OX_8355_GN_hnmt_b_PE_2_SV_
MetazoaEchStrongylocentrotuspurpuratus_3829878_HNMTB_XENLA_Histamine_N_methyltransferase_B_OS_Xenopus_laevis_OX_8355_GN_hnmt_b_PE_2_SV_
MetazoaEchStrongylocentrotuspurpuratus_3829879_HNMTB_XENLA_Histamine_N_methyltransferase_B_OS_Xenopus_laevis_OX_8355_GN_hnmt_b_PE_2_SV_
MetazoaEchStrongylocentrotuspurpuratus_3829877_HNMTB_XENLA_Histamine_N_methyltransferase_B_OS_Xenopus_laevis_OX_8355_GN_hnmt_b_PE_2_SV_
MetazoaEchinodermataAcanthasterplanci_3790323_HNMTA_XENLA_Histamine_N_methyltransferase_A_OS_Xenopus_laevis_OX_8355_GN_hnmt_a_PE_2_SV_
MetazoaVertebrateHomosapiens_6456605_HNMT_HUMAN_Histamine_N_methyltransferase_OS_Homo_sapiens_OX_9606_GN_HNMT_PE_1_SV_
MetazoaVertebrateMusmusculus_6544014_HNMT_MOUSE_Histamine_N_methyltransferase_OS_Mus_musculus_OX_10090_GN_Hnmt_PE_1_SV_
MetazoaVertebrateMonodelphisdomestica_6524613_HNMT_BOVIN_Histamine_N_methyltransferase_OS_Bos_taurus_OX_9913_GN_HNMT_PE_2_SV_
MetazoaVertebrateGallusgallus_6410536_HNMT_MOUSE_Histamine_N_methyltransferase_OS_Mus_musculus_OX_10090_GN_Hnmt_PE_1_SV_
MetazoaVertebrateAnoliscarolinensis_6308428_HNMT_MOUSE_Histamine_N_methyltransferase_OS_Mus_musculus_OX_10090_GN_Hnmt_PE_1_SV_
MetazoaVertebrateAnoliscarolinensis_6338281_HNMT_MOUSE_Histamine_N_methyltransferase_OS_Mus_musculus_OX_10090_GN_Hnmt_PE_1_SV_
MetazoaVertebrateEP00079Xenopustropicalis_6346836_HNMT_XENTR_Histamine_N_methyltransferase_OS_Xenopus_tropicalis_OX_8364_GN_hnmt_PE_2_SV_
MetazoaVertebrateEP00066Takifugurubripes_5684817_HNMT_TETNG_Histamine_N_methyltransferase_OS_Tetraodon_nigroviridis_OX_99883_GN_hnmt_PE_3_SV_
MetazoaVertebrateEP00067Daniorerio_5715659_HNMT_DANRE_Histamine_N_methyltransferase_OS_Danio_rerio_OX_7955_GN_hnmt_PE_2_SV_
MetazoaVertebrateCallorhinchusmilii_5513832_HNMT_XENTR_Histamine_N_methyltransferase_OS_Xenopus_tropicalis_OX_8364_GN_hnmt_PE_2_SV_
MetazoaVertebrateLatimeriachalumnae_5779783_HNMTA_XENLA_Histamine_N_methyltransferase_A_OS_Xenopus_laevis_OX_8355_GN_hnmt_a_PE_2_SV_
MetazoaVertebrateAnoliscarolinensis_6323974_HNMTL_CHICK_Carnosine_N_methyltransferase_2_OS_Gallus_gallus_OX_9031_PE_1_SV_
MetazoaVertebrateGallusgallus_6428529_HNMTL_CHICK_Carnosine_N_methyltransferase_2_OS_Gallus_gallus_OX_9031_PE_1_SV_
MetazoaVertebrateLatimeriachalumnae_5775535_HNMTA_XENLA_Histamine_N_methyltransferase_A_OS_Xenopus_laevis_OX_8355_GN_hnmt_a_PE_2_SV_
MetazoaVertebrateLatimeriachalumnae_5775545_HNMTA_XENLA_Histamine_N_methyltransferase_A_OS_Xenopus_laevis_OX_8355_GN_hnmt_a_PE_2_SV_
MetazoaVertebrateLatimeriachalumnae_5775546_HNMTA_XENLA_Histamine_N_methyltransferase_A_OS_Xenopus_laevis_OX_8355_GN_hnmt_a_PE_2_SV_
MetazoaVertebrateLatimeriachalumnae_5779784_HNMTA_XENLA_Histamine_N_methyltransferase_A_OS_Xenopus_laevis_OX_8355_GN_hnmt_a_PE_2_SV_
MetazoaVertebrateLatimeriachalumnae_5792176_HNMT_HUMAN_Histamine_N_methyltransferase_OS_Homo_sapiens_OX_9606_GN_HNMT_PE_1_SV_
MetazoaVertebrateEP00066Takifugurubripes_5704353_HNMTL_CHICK_Carnosine_N_methyltransferase_2_OS_Gallus_gallus_OX_9031_PE_1_SV_
MetazoaAcoelaHofsteniamiamia_4808_HNMTA_XENLA_Histamine_N_methyltransferase_A_OS_Xenopus_laevis_OX_8355_GN_hnmt_a_PE_2_SV_
MetazoaEchinodermataAcanthasterplanci_3801750_HNMT_XENTR_Histamine_N_methyltransferase_OS_Xenopus_tropicalis_OX_8364_GN_hnmt_PE_2_SV_
MetazoaCnidariaFungiascutaria_3199053_HNMT_XENTR_Histamine_N_methyltransferase_OS_Xenopus_tropicalis_OX_8364_GN_hnmt_PE_2_SV_
MetazoaCnidariaStylophorapistillata_3682839_HNMT_XENTR_Histamine_N_methyltransferase_OS_Xenopus_tropicalis_OX_8364_GN_hnmt_PE_2_SV_
MetazoaCnidariaHelioporacoerulea_247777_HNMTA_XENLA_Histamine_N_methyltransferase_A_OS_Xenopus_laevis_OX_8355_GN_hnmt_a_PE_2_SV_
MetazoaCnidariaParamuriceabiscaya_429387_HNMT_MOUSE_Histamine_N_methyltransferase_OS_Mus_musculus_OX_10090_GN_Hnmt_PE_1_SV_
MetazoaCnidariaFungiascutaria_3182385_HNMT_DANRE_Histamine_N_methyltransferase_OS_Danio_rerio_OX_7955_GN_hnmt_PE_2_SV_
MetazoaCnidariaNematostellavectensis_002209HNMT_BOVIN_Histamine_N_methyltransferase_OS_Bos_taurus_OX_9913_GN_HNMT_PE_2_SV_
MetazoaCnidariaNematostellavectensis_015023HNMT_MOUSE_Histamine_N_methyltransferase_OS_Mus_musculus_OX_10090_GN_Hnmt_PE_1_SV_
MetazoaCnidariaNematostellavectensis_009758HNMTB_XENLA_Histamine_N_methyltransferase_B_OS_Xenopus_laevis_OX_8355_GN_hnmt_b_PE_2_SV_
MetazoaCnidariaNematostellavectensis_3393005_HNMTB_XENLA_Histamine_N_methyltransferase_B_OS_Xenopus_laevis_OX_8355_GN_hnmt_b_PE_2_SV_
MetazoaCnidariaNematostellavectensis_3384479_HNMTA_XENLA_Histamine_N_methyltransferase_A_OS_Xenopus_laevis_OX_8355_GN_hnmt_a_PE_2_SV_
MetazoaCnidariaStylophorapistillata_3661429_HNMTA_XENLA_Histamine_N_methyltransferase_A_OS_Xenopus_laevis_OX_8355_GN_hnmt_a_PE_2_SV_
MetazoaCnidariaStylophorapistillata_3671093_HNMTA_XENLA_Histamine_N_methyltransferase_A_OS_Xenopus_laevis_OX_8355_GN_hnmt_a_PE_2_SV_
MetazoaCnidariaStylophorapistillata_3672447_HNMTA_XENLA_Histamine_N_methyltransferase_A_OS_Xenopus_laevis_OX_8355_GN_hnmt_a_PE_2_SV_
MetazoaCnidariaStylophorapistillata_3668699_HNMTA_XENLA_Histamine_N_methyltransferase_A_OS_Xenopus_laevis_OX_8355_GN_hnmt_a_PE_2_SV_
MetazoaCnidariaStylophorapistillata_3663926_HNMT_DANRE_Histamine_N_methyltransferase_OS_Danio_rerio_OX_7955_GN_hnmt_PE_2_SV_
0.29

12D.

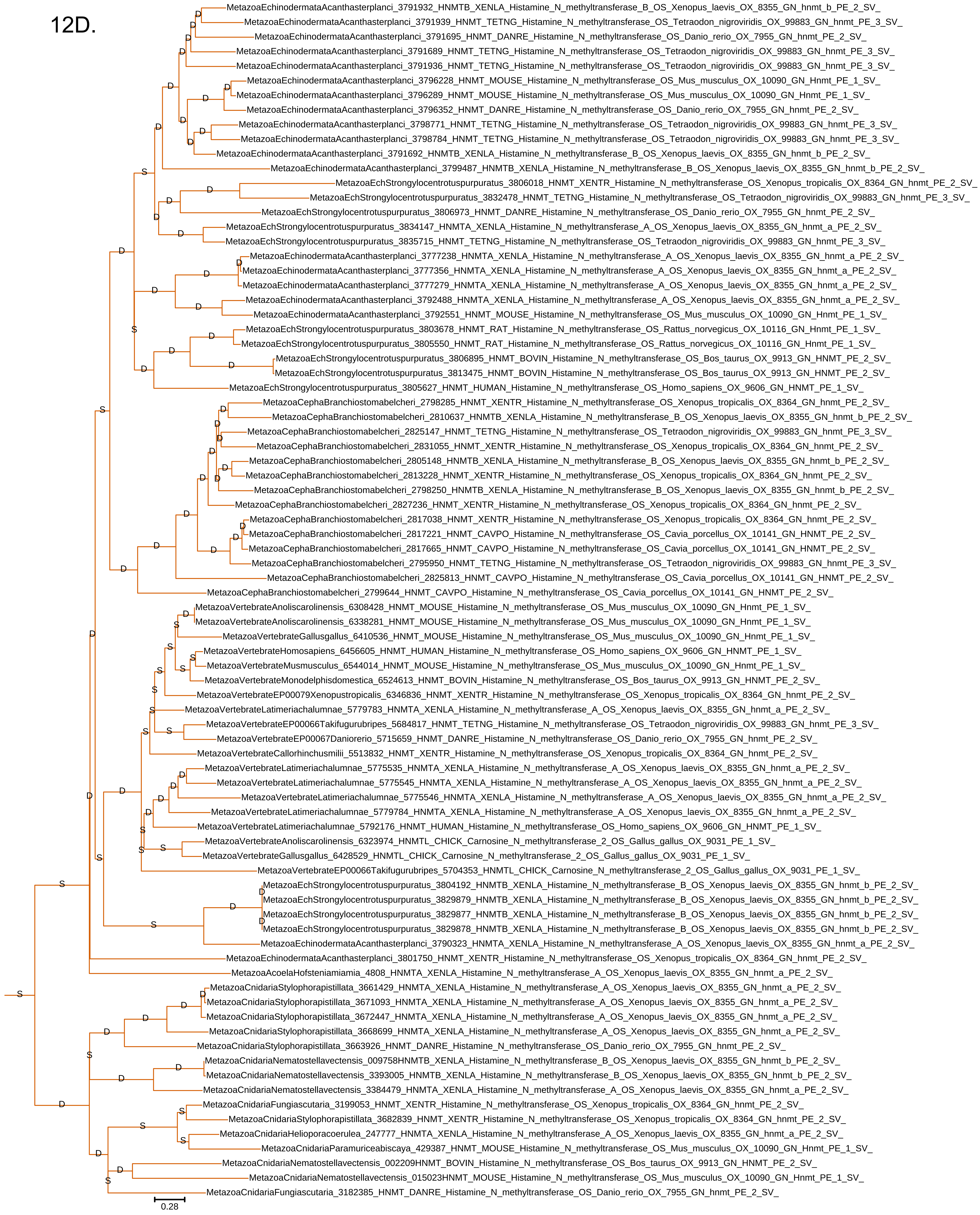
MetazoaEchinodermataAcanthasterplanci_3791932_HNMTB_XENLA_Histamine_N_methyltransferase_B_OS_Xenopus_laevis_OX_8355_GN_hnmt_b_PE_2_SV_
MetazoaEchinodermataAcanthasterplanci_3791939_HNMT_TETNG_Histamine_N_methyltransferase_OS_Tetraodon_nigroviridis_OX_99883_GN_hnmt_PE_3_SV_
MetazoaEchinodermataAcanthasterplanci_3791695_HNMT_DANRE_Histamine_N_methyltransferase_OS_Danio_rerio_OX_7955_GN_hnmt_PE_2_SV_
MetazoaEchinodermataAcanthasterplanci_3791689_HNMT_TETNG_Histamine_N_methyltransferase_OS_Tetraodon_nigroviridis_OX_99883_GN_hnmt_PE_3_SV_
MetazoaEchinodermataAcanthasterplanci_3791936_HNMT_TETNG_Histamine_N_methyltransferase_OS_Tetraodon_nigroviridis_OX_99883_GN_hnmt_PE_3_SV_
MetazoaEchinodermataAcanthasterplanci_3796228_HNMT_MOUSE_Histamine_N_methyltransferase_OS_Mus_musculus_OX_10090_GN_Hnmt_PE_1_SV_
MetazoaEchinodermataAcanthasterplanci_3796289_HNMT_MOUSE_Histamine_N_methyltransferase_OS_Mus_musculus_OX_10090_GN_Hnmt_PE_1_SV_
MetazoaEchinodermataAcanthasterplanci_3796352_HNMT_DANRE_Histamine_N_methyltransferase_OS_Danio_rerio_OX_7955_GN_hnmt_PE_2_SV_
MetazoaEchinodermataAcanthasterplanci_3798771_HNMT_TETNG_Histamine_N_methyltransferase_OS_Tetraodon_nigroviridis_OX_99883_GN_hnmt_PE_3_SV_
MetazoaEchinodermataAcanthasterplanci_3798784_HNMT_TETNG_Histamine_N_methyltransferase_OS_Tetraodon_nigroviridis_OX_99883_GN_hnmt_PE_3_SV_
MetazoaEchinodermataAcanthasterplanci_3791692_HNMTB_XENLA_Histamine_N_methyltransferase_B_OS_Xenopus_laevis_OX_8355_GN_hnmt_b_PE_2_SV_
MetazoaEchinodermataAcanthasterplanci_3799487_HNMTB_XENLA_Histamine_N_methyltransferase_B_OS_Xenopus_laevis_OX_8355_GN_hnmt_b_PE_2_SV_
MetazoaEchStrongylocentrotuspurpuratus_3806018_HNMT_XENTR_Histamine_N_methyltransferase_OS_Xenopus_tropicalis_OX_8364_GN_hnmt_PE_2_SV_
MetazoaEchStrongylocentrotuspurpuratus_3832478_HNMT_TETNG_Histamine_N_methyltransferase_OS_Tetraodon_nigroviridis_OX_99883_GN_hnmt_PE_3_SV_
MetazoaEchStrongylocentrotuspurpuratus_3806973_HNMT_DANRE_Histamine_N_methyltransferase_OS_Danio_rerio_OX_7955_GN_hnmt_PE_2_SV_
MetazoaEchStrongylocentrotuspurpuratus_3834147_HNMTA_XENLA_Histamine_N_methyltransferase_A_OS_Xenopus_laevis_OX_8355_GN_hnmt_a_PE_2_SV_
MetazoaEchStrongylocentrotuspurpuratus_3835715_HNMT_TETNG_Histamine_N_methyltransferase_OS_Tetraodon_nigroviridis_OX_99883_GN_hnmt_PE_3_SV_
MetazoaEchinodermataAcanthasterplanci_3777238_HNMTA_XENLA_Histamine_N_methyltransferase_A_OS_Xenopus_laevis_OX_8355_GN_hnmt_a_PE_2_SV_
MetazoaEchinodermataAcanthasterplanci_3777356_HNMTA_XENLA_Histamine_N_methyltransferase_A_OS_Xenopus_laevis_OX_8355_GN_hnmt_a_PE_2_SV_
MetazoaEchinodermataAcanthasterplanci_3777279_HNMTA_XENLA_Histamine_N_methyltransferase_A_OS_Xenopus_laevis_OX_8355_GN_hnmt_a_PE_2_SV_
MetazoaEchinodermataAcanthasterplanci_3792488_HNMTA_XENLA_Histamine_N_methyltransferase_A_OS_Xenopus_laevis_OX_8355_GN_hnmt_a_PE_2_SV_
MetazoaEchinodermataAcanthasterplanci_3792551_HNMT_MOUSE_Histamine_N_methyltransferase_OS_Mus_musculus_OX_10090_GN_Hnmt_PE_1_SV_
MetazoaEchStrongylocentrotuspurpuratus_3803678_HNMT_RAT_Histamine_N_methyltransferase_OS_Rattus_norvegicus_OX_10116_GN_Hnmt_PE_1_SV_
MetazoaEchStrongylocentrotuspurpuratus_3805550_HNMT_RAT_Histamine_N_methyltransferase_OS_Rattus_norvegicus_OX_10116_GN_Hnmt_PE_1_SV_
MetazoaEchStrongylocentrotuspurpuratus_3806895_HNMT_BOVIN_Histamine_N_methyltransferase_OS_Bos_taurus_OX_9913_GN_HNMT_PE_2_SV_
MetazoaEchStrongylocentrotuspurpuratus_3813475_HNMT_BOVIN_Histamine_N_methyltransferase_OS_Bos_taurus_OX_9913_GN_HNMT_PE_2_SV_
MetazoaEchStrongylocentrotuspurpuratus_3805627_HNMT_HUMAN_Histamine_N_methyltransferase_OS_Homo_sapiens_OX_9606_GN_HNMT_PE_1_SV_
MetazoaCephaBranchiostomabelcheri_2798285_HNMT_XENTR_Histamine_N_methyltransferase_OS_Xenopus_tropicalis_OX_8364_GN_hnmt_PE_2_SV_
MetazoaCephaBranchiostomabelcheri_2810637_HNMTB_XENLA_Histamine_N_methyltransferase_B_OS_Xenopus_laevis_OX_8355_GN_hnmt_b_PE_2_SV_
MetazoaCephaBranchiostomabelcheri_2825147_HNMT_TETNG_Histamine_N_methyltransferase_OS_Tetraodon_nigroviridis_OX_99883_GN_hnmt_PE_3_SV_
MetazoaCephaBranchiostomabelcheri_2831055_HNMT_XENTR_Histamine_N_methyltransferase_OS_Xenopus_tropicalis_OX_8364_GN_hnmt_PE_2_SV_
MetazoaCephaBranchiostomabelcheri_2805148_HNMTB_XENLA_Histamine_N_methyltransferase_B_OS_Xenopus_laevis_OX_8355_GN_hnmt_b_PE_2_SV_
MetazoaCephaBranchiostomabelcheri_2813228_HNMT_XENTR_Histamine_N_methyltransferase_OS_Xenopus_tropicalis_OX_8364_GN_hnmt_PE_2_SV_
MetazoaCephaBranchiostomabelcheri_2798250_HNMTB_XENLA_Histamine_N_methyltransferase_B_OS_Xenopus_laevis_OX_8355_GN_hnmt_b_PE_2_SV_
MetazoaCephaBranchiostomabelcheri_2827236_HNMT_XENTR_Histamine_N_methyltransferase_OS_Xenopus_tropicalis_OX_8364_GN_hnmt_PE_2_SV_
MetazoaCephaBranchiostomabelcheri_2817038_HNMT_XENTR_Histamine_N_methyltransferase_OS_Xenopus_tropicalis_OX_8364_GN_hnmt_PE_2_SV_
MetazoaCephaBranchiostomabelcheri_2817221_HNMT_CAVPO_Histamine_N_methyltransferase_OS_Cavia_porcellus_OX_10141_GN_HNMT_PE_2_SV_
MetazoaCephaBranchiostomabelcheri_2817665_HNMT_CAVPO_Histamine_N_methyltransferase_OS_Cavia_porcellus_OX_10141_GN_HNMT_PE_2_SV_
MetazoaCephaBranchiostomabelcheri_2795950_HNMT_TETNG_Histamine_N_methyltransferase_OS_Tetraodon_nigroviridis_OX_99883_GN_hnmt_PE_3_SV_
MetazoaCephaBranchiostomabelcheri_2825813_HNMT_CAVPO_Histamine_N_methyltransferase_OS_Cavia_porcellus_OX_10141_GN_HNMT_PE_2_SV_
MetazoaCephaBranchiostomabelcheri_2799644_HNMT_CAVPO_Histamine_N_methyltransferase_OS_Cavia_porcellus_OX_10141_GN_HNMT_PE_2_SV_
MetazoaVertebrateAnoliscarolinensis_6308428_HNMT_MOUSE_Histamine_N_methyltransferase_OS_Mus_musculus_OX_10090_GN_Hnmt_PE_1_SV_
MetazoaVertebrateAnoliscarolinensis_6338281_HNMT_MOUSE_Histamine_N_methyltransferase_OS_Mus_musculus_OX_10090_GN_Hnmt_PE_1_SV_
MetazoaVertebrateGallusgallus_6410536_HNMT_MOUSE_Histamine_N_methyltransferase_OS_Mus_musculus_OX_10090_GN_Hnmt_PE_1_SV_
MetazoaVertebrateHomosapiens_6456605_HNMT_HUMAN_Histamine_N_methyltransferase_OS_Homo_sapiens_OX_9606_GN_HNMT_PE_1_SV_
MetazoaVertebrateMusmusculus_6544014_HNMT_MOUSE_Histamine_N_methyltransferase_OS_Mus_musculus_OX_10090_GN_Hnmt_PE_1_SV_
MetazoaVertebrateMonodelphisdomestica_6524613_HNMT_BOVIN_Histamine_N_methyltransferase_OS_Bos_taurus_OX_9913_GN_HNMT_PE_2_SV_
MetazoaVertebrateEP00079Xenopustropicalis_6346836_HNMT_XENTR_Histamine_N_methyltransferase_OS_Xenopus_tropicalis_OX_8364_GN_hnmt_PE_2_SV_
MetazoaVertebrateLatimeriachalumnae_5779783_HNMTA_XENLA_Histamine_N_methyltransferase_A_OS_Xenopus_laevis_OX_8355_GN_hnmt_a_PE_2_SV_
MetazoaVertebrateEP00066Takifugurubripes_5684817_HNMT_TETNG_Histamine_N_methyltransferase_OS_Tetraodon_nigroviridis_OX_99883_GN_hnmt_PE_3_SV_
MetazoaVertebrateEP00067Daniorerio_5715659_HNMT_DANRE_Histamine_N_methyltransferase_OS_Danio_rerio_OX_7955_GN_hnmt_PE_2_SV_
MetazoaVertebrateCallorhinchusmilii_5513832_HNMT_XENTR_Histamine_N_methyltransferase_OS_Xenopus_tropicalis_OX_8364_GN_hnmt_PE_2_SV_
MetazoaVertebrateLatimeriachalumnae_5775535_HNMTA_XENLA_Histamine_N_methyltransferase_A_OS_Xenopus_laevis_OX_8355_GN_hnmt_a_PE_2_SV_
MetazoaVertebrateLatimeriachalumnae_5775545_HNMTA_XENLA_Histamine_N_methyltransferase_A_OS_Xenopus_laevis_OX_8355_GN_hnmt_a_PE_2_SV_
MetazoaVertebrateLatimeriachalumnae_5775546_HNMTA_XENLA_Histamine_N_methyltransferase_A_OS_Xenopus_laevis_OX_8355_GN_hnmt_a_PE_2_SV_
MetazoaVertebrateLatimeriachalumnae_5779784_HNMTA_XENLA_Histamine_N_methyltransferase_A_OS_Xenopus_laevis_OX_8355_GN_hnmt_a_PE_2_SV_
MetazoaVertebrateLatimeriachalumnae_5792176_HNMT_HUMAN_Histamine_N_methyltransferase_OS_Homo_sapiens_OX_9606_GN_HNMT_PE_1_SV_
MetazoaVertebrateAnoliscarolinensis_6323974_HNMTL_CHICK_Carnosine_N_methyltransferase_2_OS_Gallus_gallus_OX_9031_PE_1_SV_
MetazoaVertebrateGallusgallus_6428529_HNMTL_CHICK_Carnosine_N_methyltransferase_2_OS_Gallus_gallus_OX_9031_PE_1_SV_
MetazoaVertebrateEP00066Takifugurubripes_5704353_HNMTL_CHICK_Carnosine_N_methyltransferase_2_OS_Gallus_gallus_OX_9031_PE_1_SV_
MetazoaEchStrongylocentrotuspurpuratus_3804192_HNMTB_XENLA_Histamine_N_methyltransferase_B_OS_Xenopus_laevis_OX_8355_GN_hnmt_b_PE_2_SV_
MetazoaEchStrongylocentrotuspurpuratus_3829879_HNMTB_XENLA_Histamine_N_methyltransferase_B_OS_Xenopus_laevis_OX_8355_GN_hnmt_b_PE_2_SV_
MetazoaEchStrongylocentrotuspurpuratus_3829877_HNMTB_XENLA_Histamine_N_methyltransferase_B_OS_Xenopus_laevis_OX_8355_GN_hnmt_b_PE_2_SV_
MetazoaEchStrongylocentrotuspurpuratus_3829878_HNMTB_XENLA_Histamine_N_methyltransferase_B_OS_Xenopus_laevis_OX_8355_GN_hnmt_b_PE_2_SV_
MetazoaEchinodermataAcanthasterplanci_3790323_HNMTA_XENLA_Histamine_N_methyltransferase_A_OS_Xenopus_laevis_OX_8355_GN_hnmt_a_PE_2_SV_
MetazoaEchinodermataAcanthasterplanci_3801750_HNMT_XENTR_Histamine_N_methyltransferase_OS_Xenopus_tropicalis_OX_8364_GN_hnmt_PE_2_SV_
MetazoaAcoelaHofsteniamiamia_4808_HNMTA_XENLA_Histamine_N_methyltransferase_A_OS_Xenopus_laevis_OX_8355_GN_hnmt_a_PE_2_SV_
MetazoaCnidariaStylophorapistillata_3661429_HNMTA_XENLA_Histamine_N_methyltransferase_A_OS_Xenopus_laevis_OX_8355_GN_hnmt_a_PE_2_SV_
MetazoaCnidariaStylophorapistillata_3671093_HNMTA_XENLA_Histamine_N_methyltransferase_A_OS_Xenopus_laevis_OX_8355_GN_hnmt_a_PE_2_SV_
MetazoaCnidariaStylophorapistillata_3672447_HNMTA_XENLA_Histamine_N_methyltransferase_A_OS_Xenopus_laevis_OX_8355_GN_hnmt_a_PE_2_SV_
MetazoaCnidariaStylophorapistillata_3668699_HNMTA_XENLA_Histamine_N_methyltransferase_A_OS_Xenopus_laevis_OX_8355_GN_hnmt_a_PE_2_SV_
MetazoaCnidariaStylophorapistillata_3663926_HNMT_DANRE_Histamine_N_methyltransferase_OS_Danio_rerio_OX_7955_GN_hnmt_PE_2_SV_
MetazoaCnidariaNematostellavectensis_009758HNMTB_XENLA_Histamine_N_methyltransferase_B_OS_Xenopus_laevis_OX_8355_GN_hnmt_b_PE_2_SV_
MetazoaCnidariaNematostellavectensis_3393005_HNMTB_XENLA_Histamine_N_methyltransferase_B_OS_Xenopus_laevis_OX_8355_GN_hnmt_b_PE_2_SV_
MetazoaCnidariaNematostellavectensis_3384479_HNMTA_XENLA_Histamine_N_methyltransferase_A_OS_Xenopus_laevis_OX_8355_GN_hnmt_a_PE_2_SV_
MetazoaCnidariaFungiascutaria_3199053_HNMT_XENTR_Histamine_N_methyltransferase_OS_Xenopus_tropicalis_OX_8364_GN_hnmt_PE_2_SV_
MetazoaCnidariaStylophorapistillata_3682839_HNMT_XENTR_Histamine_N_methyltransferase_OS_Xenopus_tropicalis_OX_8364_GN_hnmt_PE_2_SV_
MetazoaCnidariaHelioporacoerulea_247777_HNMTA_XENLA_Histamine_N_methyltransferase_A_OS_Xenopus_laevis_OX_8355_GN_hnmt_a_PE_2_SV_
MetazoaCnidariaParamuriceabiscaya_429387_HNMT_MOUSE_Histamine_N_methyltransferase_OS_Mus_musculus_OX_10090_GN_Hnmt_PE_1_SV_
MetazoaCnidariaNematostellavectensis_002209HNMT_BOVIN_Histamine_N_methyltransferase_OS_Bos_taurus_OX_9913_GN_HNMT_PE_2_SV_
MetazoaCnidariaNematostellavectensis_015023HNMT_MOUSE_Histamine_N_methyltransferase_OS_Mus_musculus_OX_10090_GN_Hnmt_PE_1_SV_
MetazoaCnidariaFungiascutaria_3182385_HNMT_DANRE_Histamine_N_methyltransferase_OS_Danio_rerio_OX_7955_GN_hnmt_PE_2_SV_
0.28

12E.

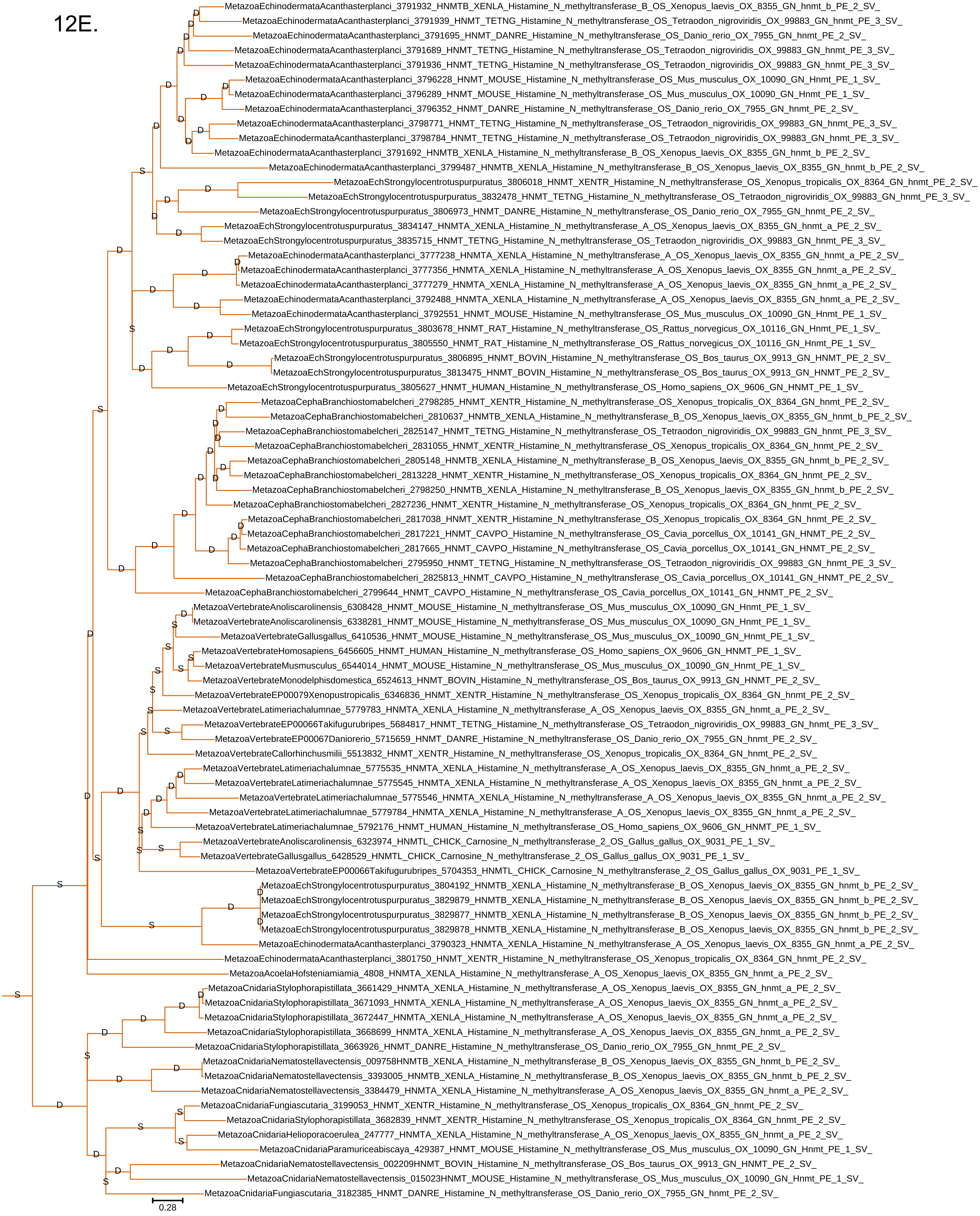
MetazoaEchinodermataAcanthasterplanci_3791932_HNMTB_XENLA_Histamine_N_methyltransferase_B_OS_Xenopus_laevis_OX_8355_GN_hnmt_b_PE_2_SV_
MetazoaEchinodermataAcanthasterplanci_3791939_HNMT_TETNG_Histamine_N_methyltransferase_OS_Tetraodon_nigroviridis_OX_99883_GN_hnmt_PE_3_SV_
MetazoaEchinodermataAcanthasterplanci_3791695_HNMT_DANRE_Histamine_N_methyltransferase_OS_Danio_rerio_OX_7955_GN_hnmt_PE_2_SV_
MetazoaEchinodermataAcanthasterplanci_3791689_HNMT_TETNG_Histamine_N_methyltransferase_OS_Tetraodon_nigroviridis_OX_99883_GN_hnmt_PE_3_SV_
MetazoaEchinodermataAcanthasterplanci_3791936_HNMT_TETNG_Histamine_N_methyltransferase_OS_Tetraodon_nigroviridis_OX_99883_GN_hnmt_PE_3_SV_
MetazoaEchinodermataAcanthasterplanci_3796228_HNMT_MOUSE_Histamine_N_methyltransferase_OS_Mus_musculus_OX_10090_GN_Hnmt_PE_1_SV_
MetazoaEchinodermataAcanthasterplanci_3796289_HNMT_MOUSE_Histamine_N_methyltransferase_OS_Mus_musculus_OX_10090_GN_Hnmt_PE_1_SV_
MetazoaEchinodermataAcanthasterplanci_3796352_HNMT_DANRE_Histamine_N_methyltransferase_OS_Danio_rerio_OX_7955_GN_hnmt_PE_2_SV_
MetazoaEchinodermataAcanthasterplanci_3798771_HNMT_TETNG_Histamine_N_methyltransferase_OS_Tetraodon_nigroviridis_OX_99883_GN_hnmt_PE_3_SV_
MetazoaEchinodermataAcanthasterplanci_3798784_HNMT_TETNG_Histamine_N_methyltransferase_OS_Tetraodon_nigroviridis_OX_99883_GN_hnmt_PE_3_SV_
MetazoaEchinodermataAcanthasterplanci_3791692_HNMTB_XENLA_Histamine_N_methyltransferase_B_OS_Xenopus_laevis_OX_8355_GN_hnmt_b_PE_2_SV_
MetazoaEchinodermataAcanthasterplanci_3799487_HNMTB_XENLA_Histamine_N_methyltransferase_B_OS_Xenopus_laevis_OX_8355_GN_hnmt_b_PE_2_SV_
MetazoaEchStrongylocentrotuspurpuratus_3806018_HNMT_XENTR_Histamine_N_methyltransferase_OS_Xenopus_tropicalis_OX_8364_GN_hnmt_PE_2_SV_
MetazoaEchStrongylocentrotuspurpuratus_3832478_HNMT_TETNG_Histamine_N_methyltransferase_OS_Tetraodon_nigroviridis_OX_99883_GN_hnmt_PE_3_SV_
MetazoaEchStrongylocentrotuspurpuratus_3806973_HNMT_DANRE_Histamine_N_methyltransferase_OS_Danio_rerio_OX_7955_GN_hnmt_PE_2_SV_
MetazoaEchStrongylocentrotuspurpuratus_3834147_HNMTA_XENLA_Histamine_N_methyltransferase_A_OS_Xenopus_laevis_OX_8355_GN_hnmt_a_PE_2_SV_
MetazoaEchStrongylocentrotuspurpuratus_3835715_HNMT_TETNG_Histamine_N_methyltransferase_OS_Tetraodon_nigroviridis_OX_99883_GN_hnmt_PE_3_SV_
MetazoaEchinodermataAcanthasterplanci_3777238_HNMTA_XENLA_Histamine_N_methyltransferase_A_OS_Xenopus_laevis_OX_8355_GN_hnmt_a_PE_2_SV_
MetazoaEchinodermataAcanthasterplanci_3777356_HNMTA_XENLA_Histamine_N_methyltransferase_A_OS_Xenopus_laevis_OX_8355_GN_hnmt_a_PE_2_SV_
MetazoaEchinodermataAcanthasterplanci_3777279_HNMTA_XENLA_Histamine_N_methyltransferase_A_OS_Xenopus_laevis_OX_8355_GN_hnmt_a_PE_2_SV_
MetazoaEchinodermataAcanthasterplanci_3792488_HNMTA_XENLA_Histamine_N_methyltransferase_A_OS_Xenopus_laevis_OX_8355_GN_hnmt_a_PE_2_SV_
MetazoaEchinodermataAcanthasterplanci_3792551_HNMT_MOUSE_Histamine_N_methyltransferase_OS_Mus_musculus_OX_10090_GN_Hnmt_PE_1_SV_
MetazoaEchStrongylocentrotuspurpuratus_3803678_HNMT_RAT_Histamine_N_methyltransferase_OS_Rattus_norvegicus_OX_10116_GN_Hnmt_PE_1_SV_
MetazoaEchStrongylocentrotuspurpuratus_3805550_HNMT_RAT_Histamine_N_methyltransferase_OS_Rattus_norvegicus_OX_10116_GN_Hnmt_PE_1_SV_
MetazoaEchStrongylocentrotuspurpuratus_3806895_HNMT_BOVIN_Histamine_N_methyltransferase_OS_Bos_taurus_OX_9913_GN_HNMT_PE_2_SV_
MetazoaEchStrongylocentrotuspurpuratus_3813475_HNMT_BOVIN_Histamine_N_methyltransferase_OS_Bos_taurus_OX_9913_GN_HNMT_PE_2_SV_
MetazoaEchStrongylocentrotuspurpuratus_3805627_HNMT_HUMAN_Histamine_N_methyltransferase_OS_Homo_sapiens_OX_9606_GN_HNMT_PE_1_SV_
MetazoaCephaBranchiostomabelcheri_2798285_HNMT_XENTR_Histamine_N_methyltransferase_OS_Xenopus_tropicalis_OX_8364_GN_hnmt_PE_2_SV_
MetazoaCephaBranchiostomabelcheri_2810637_HNMTB_XENLA_Histamine_N_methyltransferase_B_OS_Xenopus_laevis_OX_8355_GN_hnmt_b_PE_2_SV_
MetazoaCephaBranchiostomabelcheri_2825147_HNMT_TETNG_Histamine_N_methyltransferase_OS_Tetraodon_nigroviridis_OX_99883_GN_hnmt_PE_3_SV_
MetazoaCephaBranchiostomabelcheri_2831055_HNMT_XENTR_Histamine_N_methyltransferase_OS_Xenopus_tropicalis_OX_8364_GN_hnmt_PE_2_SV_
MetazoaCephaBranchiostomabelcheri_2805148_HNMTB_XENLA_Histamine_N_methyltransferase_B_OS_Xenopus_laevis_OX_8355_GN_hnmt_b_PE_2_SV_
MetazoaCephaBranchiostomabelcheri_2813228_HNMT_XENTR_Histamine_N_methyltransferase_OS_Xenopus_tropicalis_OX_8364_GN_hnmt_PE_2_SV_
MetazoaCephaBranchiostomabelcheri_2798250_HNMTB_XENLA_Histamine_N_methyltransferase_B_OS_Xenopus_laevis_OX_8355_GN_hnmt_b_PE_2_SV_
MetazoaCephaBranchiostomabelcheri_2827236_HNMT_XENTR_Histamine_N_methyltransferase_OS_Xenopus_tropicalis_OX_8364_GN_hnmt_PE_2_SV_
MetazoaCephaBranchiostomabelcheri_2817038_HNMT_XENTR_Histamine_N_methyltransferase_OS_Xenopus_tropicalis_OX_8364_GN_hnmt_PE_2_SV_
MetazoaCephaBranchiostomabelcheri_2817221_HNMT_CAVPO_Histamine_N_methyltransferase_OS_Cavia_porcellus_OX_10141_GN_HNMT_PE_2_SV_
MetazoaCephaBranchiostomabelcheri_2817665_HNMT_CAVPO_Histamine_N_methyltransferase_OS_Cavia_porcellus_OX_10141_GN_HNMT_PE_2_SV_
MetazoaCephaBranchiostomabelcheri_2795950_HNMT_TETNG_Histamine_N_methyltransferase_OS_Tetraodon_nigroviridis_OX_99883_GN_hnmt_PE_3_SV_
MetazoaCephaBranchiostomabelcheri_2825813_HNMT_CAVPO_Histamine_N_methyltransferase_OS_Cavia_porcellus_OX_10141_GN_HNMT_PE_2_SV_
MetazoaCephaBranchiostomabelcheri_2799644_HNMT_CAVPO_Histamine_N_methyltransferase_OS_Cavia_porcellus_OX_10141_GN_HNMT_PE_2_SV_
MetazoaVertebrateAnoliscarolinensis_6308428_HNMT_MOUSE_Histamine_N_methyltransferase_OS_Mus_musculus_OX_10090_GN_Hnmt_PE_1_SV_
MetazoaVertebrateAnoliscarolinensis_6338281_HNMT_MOUSE_Histamine_N_methyltransferase_OS_Mus_musculus_OX_10090_GN_Hnmt_PE_1_SV_
MetazoaVertebrateGallusgallus_6410536_HNMT_MOUSE_Histamine_N_methyltransferase_OS_Mus_musculus_OX_10090_GN_Hnmt_PE_1_SV_
MetazoaVertebrateHomosapiens_6456605_HNMT_HUMAN_Histamine_N_methyltransferase_OS_Homo_sapiens_OX_9606_GN_HNMT_PE_1_SV_
MetazoaVertebrateMusmusculus_6544014_HNMT_MOUSE_Histamine_N_methyltransferase_OS_Mus_musculus_OX_10090_GN_Hnmt_PE_1_SV_
MetazoaVertebrateMonodelphisdomestica_6524613_HNMT_BOVIN_Histamine_N_methyltransferase_OS_Bos_taurus_OX_9913_GN_HNMT_PE_2_SV_
MetazoaVertebrateEP00079Xenopustropicalis_6346836_HNMT_XENTR_Histamine_N_methyltransferase_OS_Xenopus_tropicalis_OX_8364_GN_hnmt_PE_2_SV_
MetazoaVertebrateLatimeriachalumnae_5779783_HNMTA_XENLA_Histamine_N_methyltransferase_A_OS_Xenopus_laevis_OX_8355_GN_hnmt_a_PE_2_SV_
MetazoaVertebrateEP00066Takifugurubripes_5684817_HNMT_TETNG_Histamine_N_methyltransferase_OS_Tetraodon_nigroviridis_OX_99883_GN_hnmt_PE_3_SV_
MetazoaVertebrateEP00067Daniorerio_5715659_HNMT_DANRE_Histamine_N_methyltransferase_OS_Danio_rerio_OX_7955_GN_hnmt_PE_2_SV_
MetazoaVertebrateCallorhinchusmilii_5513832_HNMT_XENTR_Histamine_N_methyltransferase_OS_Xenopus_tropicalis_OX_8364_GN_hnmt_PE_2_SV_
MetazoaVertebrateLatimeriachalumnae_5775535_HNMTA_XENLA_Histamine_N_methyltransferase_A_OS_Xenopus_laevis_OX_8355_GN_hnmt_a_PE_2_SV_
MetazoaVertebrateLatimeriachalumnae_5775545_HNMTA_XENLA_Histamine_N_methyltransferase_A_OS_Xenopus_laevis_OX_8355_GN_hnmt_a_PE_2_SV_
MetazoaVertebrateLatimeriachalumnae_5775546_HNMTA_XENLA_Histamine_N_methyltransferase_A_OS_Xenopus_laevis_OX_8355_GN_hnmt_a_PE_2_SV_
MetazoaVertebrateLatimeriachalumnae_5779784_HNMTA_XENLA_Histamine_N_methyltransferase_A_OS_Xenopus_laevis_OX_8355_GN_hnmt_a_PE_2_SV_
MetazoaVertebrateLatimeriachalumnae_5792176_HNMT_HUMAN_Histamine_N_methyltransferase_OS_Homo_sapiens_OX_9606_GN_HNMT_PE_1_SV_
MetazoaVertebrateAnoliscarolinensis_6323974_HNMTL_CHICK_Carnosine_N_methyltransferase_2_OS_Gallus_gallus_OX_9031_PE_1_SV_
MetazoaVertebrateGallusgallus_6428529_HNMTL_CHICK_Carnosine_N_methyltransferase_2_OS_Gallus_gallus_OX_9031_PE_1_SV_
MetazoaVertebrateEP00066Takifugurubripes_5704353_HNMTL_CHICK_Carnosine_N_methyltransferase_2_OS_Gallus_gallus_OX_9031_PE_1_SV_
MetazoaEchStrongylocentrotuspurpuratus_3804192_HNMTB_XENLA_Histamine_N_methyltransferase_B_OS_Xenopus_laevis_OX_8355_GN_hnmt_b_PE_2_SV_
MetazoaEchStrongylocentrotuspurpuratus_3829879_HNMTB_XENLA_Histamine_N_methyltransferase_B_OS_Xenopus_laevis_OX_8355_GN_hnmt_b_PE_2_SV_
MetazoaEchStrongylocentrotuspurpuratus_3829877_HNMTB_XENLA_Histamine_N_methyltransferase_B_OS_Xenopus_laevis_OX_8355_GN_hnmt_b_PE_2_SV_
MetazoaEchStrongylocentrotuspurpuratus_3829878_HNMTB_XENLA_Histamine_N_methyltransferase_B_OS_Xenopus_laevis_OX_8355_GN_hnmt_b_PE_2_SV_
MetazoaEchinodermataAcanthasterplanci_3790323_HNMTA_XENLA_Histamine_N_methyltransferase_A_OS_Xenopus_laevis_OX_8355_GN_hnmt_a_PE_2_SV_
MetazoaEchinodermataAcanthasterplanci_3801750_HNMT_XENTR_Histamine_N_methyltransferase_OS_Xenopus_tropicalis_OX_8364_GN_hnmt_PE_2_SV_
MetazoaAcoelaHofsteniamiamia_4808_HNMTA_XENLA_Histamine_N_methyltransferase_A_OS_Xenopus_laevis_OX_8355_GN_hnmt_a_PE_2_SV_
MetazoaCnidariaStylophorapistillata_3661429_HNMTA_XENLA_Histamine_N_methyltransferase_A_OS_Xenopus_laevis_OX_8355_GN_hnmt_a_PE_2_SV_
MetazoaCnidariaStylophorapistillata_3671093_HNMTA_XENLA_Histamine_N_methyltransferase_A_OS_Xenopus_laevis_OX_8355_GN_hnmt_a_PE_2_SV_
MetazoaCnidariaStylophorapistillata_3672447_HNMTA_XENLA_Histamine_N_methyltransferase_A_OS_Xenopus_laevis_OX_8355_GN_hnmt_a_PE_2_SV_
MetazoaCnidariaStylophorapistillata_3668699_HNMTA_XENLA_Histamine_N_methyltransferase_A_OS_Xenopus_laevis_OX_8355_GN_hnmt_a_PE_2_SV_
MetazoaCnidariaStylophorapistillata_3663926_HNMT_DANRE_Histamine_N_methyltransferase_OS_Danio_rerio_OX_7955_GN_hnmt_PE_2_SV_
MetazoaCnidariaNematostellavectensis_009758HNMTB_XENLA_Histamine_N_methyltransferase_B_OS_Xenopus_laevis_OX_8355_GN_hnmt_b_PE_2_SV_
MetazoaCnidariaNematostellavectensis_3393005_HNMTB_XENLA_Histamine_N_methyltransferase_B_OS_Xenopus_laevis_OX_8355_GN_hnmt_b_PE_2_SV_
MetazoaCnidariaNematostellavectensis_3384479_HNMTA_XENLA_Histamine_N_methyltransferase_A_OS_Xenopus_laevis_OX_8355_GN_hnmt_a_PE_2_SV_
MetazoaCnidariaFungiascutaria_3199053_HNMT_XENTR_Histamine_N_methyltransferase_OS_Xenopus_tropicalis_OX_8364_GN_hnmt_PE_2_SV_
MetazoaCnidariaStylophorapistillata_3682839_HNMT_XENTR_Histamine_N_methyltransferase_OS_Xenopus_tropicalis_OX_8364_GN_hnmt_PE_2_SV_
MetazoaCnidariaHelioporacoerulea_247777_HNMTA_XENLA_Histamine_N_methyltransferase_A_OS_Xenopus_laevis_OX_8355_GN_hnmt_a_PE_2_SV_
MetazoaCnidariaParamuriceabiscaya_429387_HNMT_MOUSE_Histamine_N_methyltransferase_OS_Mus_musculus_OX_10090_GN_Hnmt_PE_1_SV_
MetazoaCnidariaNematostellavectensis_002209HNMT_BOVIN_Histamine_N_methyltransferase_OS_Bos_taurus_OX_9913_GN_HNMT_PE_2_SV_
MetazoaCnidariaNematostellavectensis_015023HNMT_MOUSE_Histamine_N_methyltransferase_OS_Mus_musculus_OX_10090_GN_Hnmt_PE_1_SV_
MetazoaCnidariaFungiascutaria_3182385_HNMT_DANRE_Histamine_N_methyltransferase_OS_Danio_rerio_OX_7955_GN_hnmt_PE_2_SV_
0.28

12F.

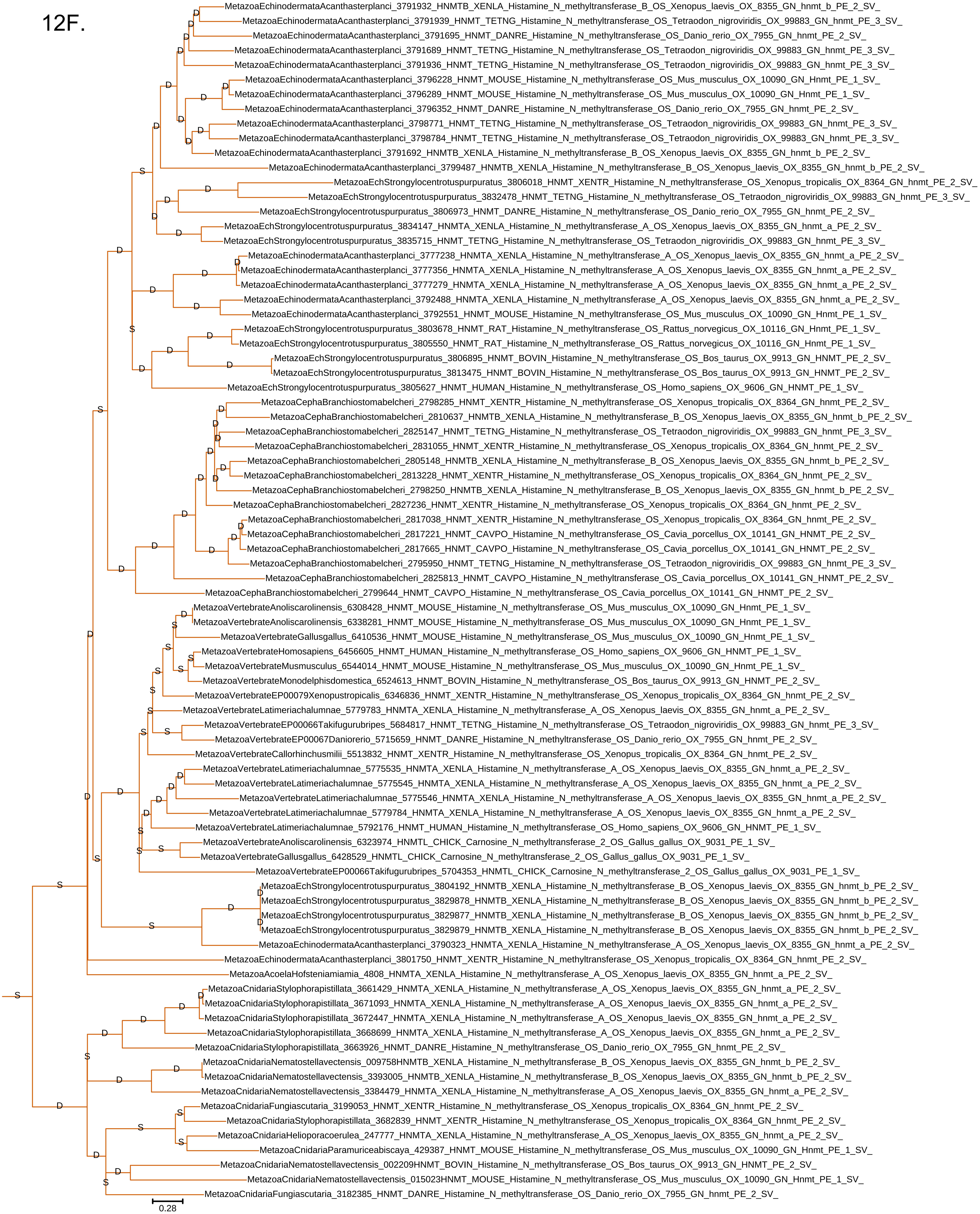
MetazoaEchinodermataAcanthasterplanci_3791932_HNMTB_XENLA_Histamine_N_methyltransferase_B_OS_Xenopus_laevis_OX_8355_GN_hnmt_b_PE_2_SV_
MetazoaEchinodermataAcanthasterplanci_3791939_HNMT_TETNG_Histamine_N_methyltransferase_OS_Tetraodon_nigroviridis_OX_99883_GN_hnmt_PE_3_SV_
MetazoaEchinodermataAcanthasterplanci_3791695_HNMT_DANRE_Histamine_N_methyltransferase_OS_Danio_rerio_OX_7955_GN_hnmt_PE_2_SV_
MetazoaEchinodermataAcanthasterplanci_3791689_HNMT_TETNG_Histamine_N_methyltransferase_OS_Tetraodon_nigroviridis_OX_99883_GN_hnmt_PE_3_SV_
MetazoaEchinodermataAcanthasterplanci_3791936_HNMT_TETNG_Histamine_N_methyltransferase_OS_Tetraodon_nigroviridis_OX_99883_GN_hnmt_PE_3_SV_
MetazoaEchinodermataAcanthasterplanci_3796228_HNMT_MOUSE_Histamine_N_methyltransferase_OS_Mus_musculus_OX_10090_GN_Hnmt_PE_1_SV_
MetazoaEchinodermataAcanthasterplanci_3796289_HNMT_MOUSE_Histamine_N_methyltransferase_OS_Mus_musculus_OX_10090_GN_Hnmt_PE_1_SV_
MetazoaEchinodermataAcanthasterplanci_3796352_HNMT_DANRE_Histamine_N_methyltransferase_OS_Danio_rerio_OX_7955_GN_hnmt_PE_2_SV_
MetazoaEchinodermataAcanthasterplanci_3798771_HNMT_TETNG_Histamine_N_methyltransferase_OS_Tetraodon_nigroviridis_OX_99883_GN_hnmt_PE_3_SV_
MetazoaEchinodermataAcanthasterplanci_3798784_HNMT_TETNG_Histamine_N_methyltransferase_OS_Tetraodon_nigroviridis_OX_99883_GN_hnmt_PE_3_SV_
MetazoaEchinodermataAcanthasterplanci_3791692_HNMTB_XENLA_Histamine_N_methyltransferase_B_OS_Xenopus_laevis_OX_8355_GN_hnmt_b_PE_2_SV_
MetazoaEchinodermataAcanthasterplanci_3799487_HNMTB_XENLA_Histamine_N_methyltransferase_B_OS_Xenopus_laevis_OX_8355_GN_hnmt_b_PE_2_SV_
MetazoaEchStrongylocentrotuspurpuratus_3806018_HNMT_XENTR_Histamine_N_methyltransferase_OS_Xenopus_tropicalis_OX_8364_GN_hnmt_PE_2_SV_
MetazoaEchStrongylocentrotuspurpuratus_3832478_HNMT_TETNG_Histamine_N_methyltransferase_OS_Tetraodon_nigroviridis_OX_99883_GN_hnmt_PE_3_SV_
MetazoaEchStrongylocentrotuspurpuratus_3806973_HNMT_DANRE_Histamine_N_methyltransferase_OS_Danio_rerio_OX_7955_GN_hnmt_PE_2_SV_
MetazoaEchStrongylocentrotuspurpuratus_3834147_HNMTA_XENLA_Histamine_N_methyltransferase_A_OS_Xenopus_laevis_OX_8355_GN_hnmt_a_PE_2_SV_
MetazoaEchStrongylocentrotuspurpuratus_3835715_HNMT_TETNG_Histamine_N_methyltransferase_OS_Tetraodon_nigroviridis_OX_99883_GN_hnmt_PE_3_SV_
MetazoaEchinodermataAcanthasterplanci_3777238_HNMTA_XENLA_Histamine_N_methyltransferase_A_OS_Xenopus_laevis_OX_8355_GN_hnmt_a_PE_2_SV_
MetazoaEchinodermataAcanthasterplanci_3777356_HNMTA_XENLA_Histamine_N_methyltransferase_A_OS_Xenopus_laevis_OX_8355_GN_hnmt_a_PE_2_SV_
MetazoaEchinodermataAcanthasterplanci_3777279_HNMTA_XENLA_Histamine_N_methyltransferase_A_OS_Xenopus_laevis_OX_8355_GN_hnmt_a_PE_2_SV_
MetazoaEchinodermataAcanthasterplanci_3792488_HNMTA_XENLA_Histamine_N_methyltransferase_A_OS_Xenopus_laevis_OX_8355_GN_hnmt_a_PE_2_SV_
MetazoaEchinodermataAcanthasterplanci_3792551_HNMT_MOUSE_Histamine_N_methyltransferase_OS_Mus_musculus_OX_10090_GN_Hnmt_PE_1_SV_
MetazoaEchStrongylocentrotuspurpuratus_3803678_HNMT_RAT_Histamine_N_methyltransferase_OS_Rattus_norvegicus_OX_10116_GN_Hnmt_PE_1_SV_
MetazoaEchStrongylocentrotuspurpuratus_3805550_HNMT_RAT_Histamine_N_methyltransferase_OS_Rattus_norvegicus_OX_10116_GN_Hnmt_PE_1_SV_
MetazoaEchStrongylocentrotuspurpuratus_3806895_HNMT_BOVIN_Histamine_N_methyltransferase_OS_Bos_taurus_OX_9913_GN_HNMT_PE_2_SV_
MetazoaEchStrongylocentrotuspurpuratus_3813475_HNMT_BOVIN_Histamine_N_methyltransferase_OS_Bos_taurus_OX_9913_GN_HNMT_PE_2_SV_
MetazoaEchStrongylocentrotuspurpuratus_3805627_HNMT_HUMAN_Histamine_N_methyltransferase_OS_Homo_sapiens_OX_9606_GN_HNMT_PE_1_SV_
MetazoaCephaBranchiostomabelcheri_2798285_HNMT_XENTR_Histamine_N_methyltransferase_OS_Xenopus_tropicalis_OX_8364_GN_hnmt_PE_2_SV_
MetazoaCephaBranchiostomabelcheri_2810637_HNMTB_XENLA_Histamine_N_methyltransferase_B_OS_Xenopus_laevis_OX_8355_GN_hnmt_b_PE_2_SV_
MetazoaCephaBranchiostomabelcheri_2825147_HNMT_TETNG_Histamine_N_methyltransferase_OS_Tetraodon_nigroviridis_OX_99883_GN_hnmt_PE_3_SV_
MetazoaCephaBranchiostomabelcheri_2831055_HNMT_XENTR_Histamine_N_methyltransferase_OS_Xenopus_tropicalis_OX_8364_GN_hnmt_PE_2_SV_
MetazoaCephaBranchiostomabelcheri_2805148_HNMTB_XENLA_Histamine_N_methyltransferase_B_OS_Xenopus_laevis_OX_8355_GN_hnmt_b_PE_2_SV_
MetazoaCephaBranchiostomabelcheri_2813228_HNMT_XENTR_Histamine_N_methyltransferase_OS_Xenopus_tropicalis_OX_8364_GN_hnmt_PE_2_SV_
MetazoaCephaBranchiostomabelcheri_2798250_HNMTB_XENLA_Histamine_N_methyltransferase_B_OS_Xenopus_laevis_OX_8355_GN_hnmt_b_PE_2_SV_
MetazoaCephaBranchiostomabelcheri_2827236_HNMT_XENTR_Histamine_N_methyltransferase_OS_Xenopus_tropicalis_OX_8364_GN_hnmt_PE_2_SV_
MetazoaCephaBranchiostomabelcheri_2817038_HNMT_XENTR_Histamine_N_methyltransferase_OS_Xenopus_tropicalis_OX_8364_GN_hnmt_PE_2_SV_
MetazoaCephaBranchiostomabelcheri_2817221_HNMT_CAVPO_Histamine_N_methyltransferase_OS_Cavia_porcellus_OX_10141_GN_HNMT_PE_2_SV_
MetazoaCephaBranchiostomabelcheri_2817665_HNMT_CAVPO_Histamine_N_methyltransferase_OS_Cavia_porcellus_OX_10141_GN_HNMT_PE_2_SV_
MetazoaCephaBranchiostomabelcheri_2795950_HNMT_TETNG_Histamine_N_methyltransferase_OS_Tetraodon_nigroviridis_OX_99883_GN_hnmt_PE_3_SV_
MetazoaCephaBranchiostomabelcheri_2825813_HNMT_CAVPO_Histamine_N_methyltransferase_OS_Cavia_porcellus_OX_10141_GN_HNMT_PE_2_SV_
MetazoaCephaBranchiostomabelcheri_2799644_HNMT_CAVPO_Histamine_N_methyltransferase_OS_Cavia_porcellus_OX_10141_GN_HNMT_PE_2_SV_
MetazoaVertebrateAnoliscarolinensis_6308428_HNMT_MOUSE_Histamine_N_methyltransferase_OS_Mus_musculus_OX_10090_GN_Hnmt_PE_1_SV_
MetazoaVertebrateAnoliscarolinensis_6338281_HNMT_MOUSE_Histamine_N_methyltransferase_OS_Mus_musculus_OX_10090_GN_Hnmt_PE_1_SV_
MetazoaVertebrateGallusgallus_6410536_HNMT_MOUSE_Histamine_N_methyltransferase_OS_Mus_musculus_OX_10090_GN_Hnmt_PE_1_SV_
MetazoaVertebrateHomosapiens_6456605_HNMT_HUMAN_Histamine_N_methyltransferase_OS_Homo_sapiens_OX_9606_GN_HNMT_PE_1_SV_
MetazoaVertebrateMusmusculus_6544014_HNMT_MOUSE_Histamine_N_methyltransferase_OS_Mus_musculus_OX_10090_GN_Hnmt_PE_1_SV_
MetazoaVertebrateMonodelphisdomestica_6524613_HNMT_BOVIN_Histamine_N_methyltransferase_OS_Bos_taurus_OX_9913_GN_HNMT_PE_2_SV_
MetazoaVertebrateEP00079Xenopustropicalis_6346836_HNMT_XENTR_Histamine_N_methyltransferase_OS_Xenopus_tropicalis_OX_8364_GN_hnmt_PE_2_SV_
MetazoaVertebrateLatimeriachalumnae_5779783_HNMTA_XENLA_Histamine_N_methyltransferase_A_OS_Xenopus_laevis_OX_8355_GN_hnmt_a_PE_2_SV_
MetazoaVertebrateEP00066Takifugurubripes_5684817_HNMT_TETNG_Histamine_N_methyltransferase_OS_Tetraodon_nigroviridis_OX_99883_GN_hnmt_PE_3_SV_
MetazoaVertebrateEP00067Daniorerio_5715659_HNMT_DANRE_Histamine_N_methyltransferase_OS_Danio_rerio_OX_7955_GN_hnmt_PE_2_SV_
MetazoaVertebrateCallorhinchusmilii_5513832_HNMT_XENTR_Histamine_N_methyltransferase_OS_Xenopus_tropicalis_OX_8364_GN_hnmt_PE_2_SV_
MetazoaVertebrateLatimeriachalumnae_5775535_HNMTA_XENLA_Histamine_N_methyltransferase_A_OS_Xenopus_laevis_OX_8355_GN_hnmt_a_PE_2_SV_
MetazoaVertebrateLatimeriachalumnae_5775545_HNMTA_XENLA_Histamine_N_methyltransferase_A_OS_Xenopus_laevis_OX_8355_GN_hnmt_a_PE_2_SV_
MetazoaVertebrateLatimeriachalumnae_5775546_HNMTA_XENLA_Histamine_N_methyltransferase_A_OS_Xenopus_laevis_OX_8355_GN_hnmt_a_PE_2_SV_
MetazoaVertebrateLatimeriachalumnae_5779784_HNMTA_XENLA_Histamine_N_methyltransferase_A_OS_Xenopus_laevis_OX_8355_GN_hnmt_a_PE_2_SV_
MetazoaVertebrateLatimeriachalumnae_5792176_HNMT_HUMAN_Histamine_N_methyltransferase_OS_Homo_sapiens_OX_9606_GN_HNMT_PE_1_SV_
MetazoaVertebrateAnoliscarolinensis_6323974_HNMTL_CHICK_Carnosine_N_methyltransferase_2_OS_Gallus_gallus_OX_9031_PE_1_SV_
MetazoaVertebrateGallusgallus_6428529_HNMTL_CHICK_Carnosine_N_methyltransferase_2_OS_Gallus_gallus_OX_9031_PE_1_SV_
MetazoaVertebrateEP00066Takifugurubripes_5704353_HNMTL_CHICK_Carnosine_N_methyltransferase_2_OS_Gallus_gallus_OX_9031_PE_1_SV_
MetazoaEchStrongylocentrotuspurpuratus_3804192_HNMTB_XENLA_Histamine_N_methyltransferase_B_OS_Xenopus_laevis_OX_8355_GN_hnmt_b_PE_2_SV_
MetazoaEchStrongylocentrotuspurpuratus_3829878_HNMTB_XENLA_Histamine_N_methyltransferase_B_OS_Xenopus_laevis_OX_8355_GN_hnmt_b_PE_2_SV_
MetazoaEchStrongylocentrotuspurpuratus_3829877_HNMTB_XENLA_Histamine_N_methyltransferase_B_OS_Xenopus_laevis_OX_8355_GN_hnmt_b_PE_2_SV_
MetazoaEchStrongylocentrotuspurpuratus_3829879_HNMTB_XENLA_Histamine_N_methyltransferase_B_OS_Xenopus_laevis_OX_8355_GN_hnmt_b_PE_2_SV_
MetazoaEchinodermataAcanthasterplanci_3790323_HNMTA_XENLA_Histamine_N_methyltransferase_A_OS_Xenopus_laevis_OX_8355_GN_hnmt_a_PE_2_SV_
MetazoaEchinodermataAcanthasterplanci_3801750_HNMT_XENTR_Histamine_N_methyltransferase_OS_Xenopus_tropicalis_OX_8364_GN_hnmt_PE_2_SV_
MetazoaAcoelaHofsteniamiamia_4808_HNMTA_XENLA_Histamine_N_methyltransferase_A_OS_Xenopus_laevis_OX_8355_GN_hnmt_a_PE_2_SV_
MetazoaCnidariaStylophorapistillata_3661429_HNMTA_XENLA_Histamine_N_methyltransferase_A_OS_Xenopus_laevis_OX_8355_GN_hnmt_a_PE_2_SV_
MetazoaCnidariaStylophorapistillata_3671093_HNMTA_XENLA_Histamine_N_methyltransferase_A_OS_Xenopus_laevis_OX_8355_GN_hnmt_a_PE_2_SV_
MetazoaCnidariaStylophorapistillata_3672447_HNMTA_XENLA_Histamine_N_methyltransferase_A_OS_Xenopus_laevis_OX_8355_GN_hnmt_a_PE_2_SV_
MetazoaCnidariaStylophorapistillata_3668699_HNMTA_XENLA_Histamine_N_methyltransferase_A_OS_Xenopus_laevis_OX_8355_GN_hnmt_a_PE_2_SV_
MetazoaCnidariaStylophorapistillata_3663926_HNMT_DANRE_Histamine_N_methyltransferase_OS_Danio_rerio_OX_7955_GN_hnmt_PE_2_SV_
MetazoaCnidariaNematostellavectensis_009758HNMTB_XENLA_Histamine_N_methyltransferase_B_OS_Xenopus_laevis_OX_8355_GN_hnmt_b_PE_2_SV_
MetazoaCnidariaNematostellavectensis_3393005_HNMTB_XENLA_Histamine_N_methyltransferase_B_OS_Xenopus_laevis_OX_8355_GN_hnmt_b_PE_2_SV_
MetazoaCnidariaNematostellavectensis_3384479_HNMTA_XENLA_Histamine_N_methyltransferase_A_OS_Xenopus_laevis_OX_8355_GN_hnmt_a_PE_2_SV_
MetazoaCnidariaFungiascutaria_3199053_HNMT_XENTR_Histamine_N_methyltransferase_OS_Xenopus_tropicalis_OX_8364_GN_hnmt_PE_2_SV_
MetazoaCnidariaStylophorapistillata_3682839_HNMT_XENTR_Histamine_N_methyltransferase_OS_Xenopus_tropicalis_OX_8364_GN_hnmt_PE_2_SV_
MetazoaCnidariaHelioporacoerulea_247777_HNMTA_XENLA_Histamine_N_methyltransferase_A_OS_Xenopus_laevis_OX_8355_GN_hnmt_a_PE_2_SV_
MetazoaCnidariaParamuriceabiscaya_429387_HNMT_MOUSE_Histamine_N_methyltransferase_OS_Mus_musculus_OX_10090_GN_Hnmt_PE_1_SV_
MetazoaCnidariaNematostellavectensis_002209HNMT_BOVIN_Histamine_N_methyltransferase_OS_Bos_taurus_OX_9913_GN_HNMT_PE_2_SV_
MetazoaCnidariaNematostellavectensis_015023HNMT_MOUSE_Histamine_N_methyltransferase_OS_Mus_musculus_OX_10090_GN_Hnmt_PE_1_SV_
MetazoaCnidariaFungiascutaria_3182385_HNMT_DANRE_Histamine_N_methyltransferase_OS_Danio_rerio_OX_7955_GN_hnmt_PE_2_SV_
0.28

Supplementary Figure 13: Evolution of MAOs. Simplified illustration of the gene tree to species tree reconciliation. Silhouettes obtained from Phylopic.org. Silhouette images are by Andrew R. Gehrke (*Hofstenia miamia*); Daniel Jaron (*Mus musculus*); Mali'o Kodis, photograph by Ching (http://www.flickr.com/photos/36302473@N03/) (*Chrysaora fuscescens*); Mario Quevedo (Asteriidae); Oliver Voigt (*Trichoplax adhaerens*); Ramiro Morales-Hojas (*Drosophila americana*); Steven Haddock, Jellywatch.org (*Hormiphora californensis*); and Tess Linden (*Salpingoeca rosetta*). MAO = monoamine oxidase.

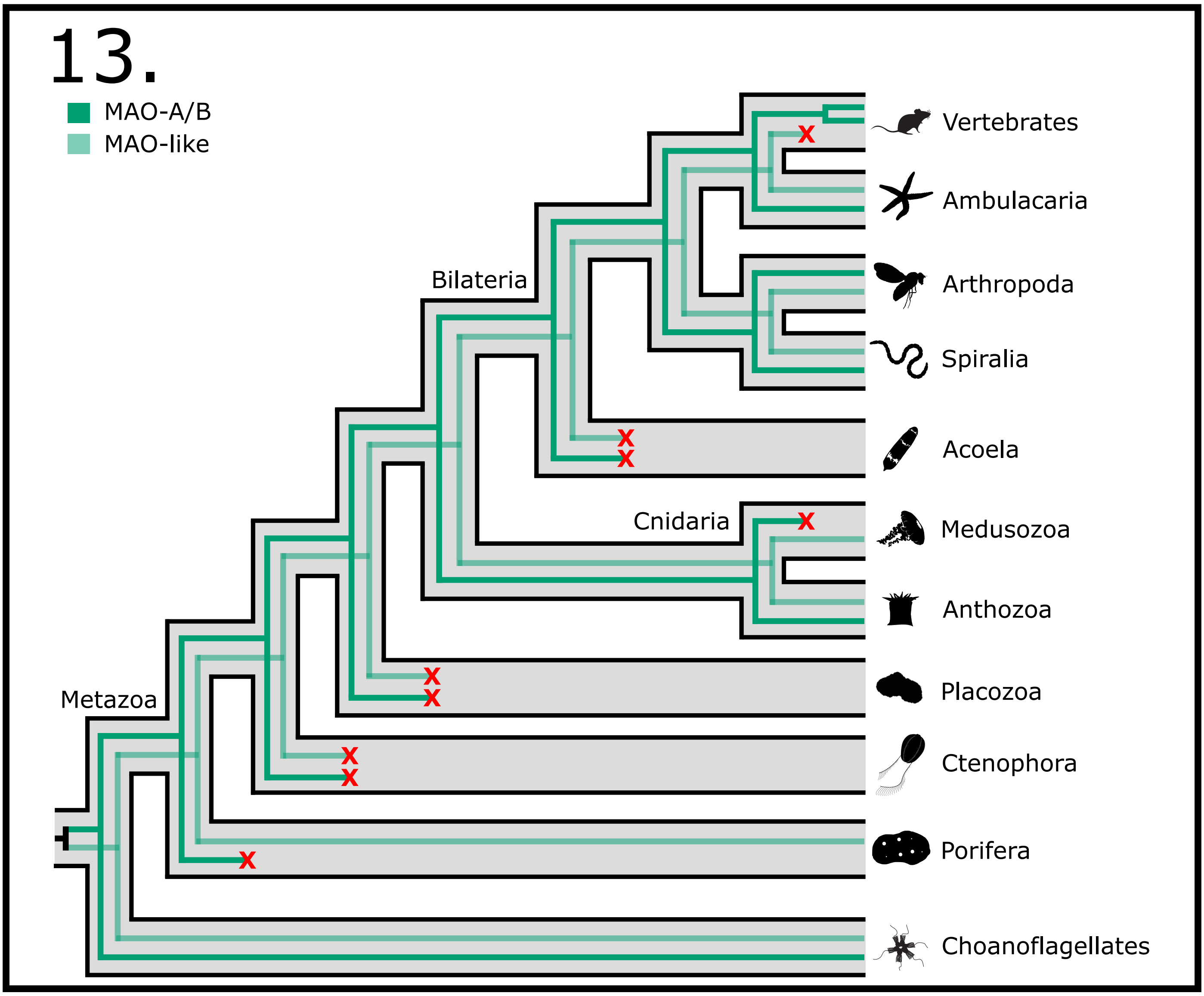
13.
MAO-A/B
MAO-like
Vertebrates
Ambulacaria
Arthropoda
Spiralia
Acoela
Medusozoa
Anthozoa
Placozoa
Ctenophora
Porifera
Choanoflagellates
Bilateria
Cnidaria
Metazoa

Supplementary Figure 14: Analysis of MAOs. A) TBE tree with TBE score supports. B) Maximum likelihood tree with ultrafast bootstrap proportion supports. C) Maximum likelihood tree including all eukaryotic sequences with ultrafast bootstrap proportion supports. D) Reconciliation using sponge-first hypothesis. E) Reconciliation using ctenophore-first hypothesis. F) Reconciliation of the UFB tree including all eukaryotic sequences. Nodes in reconciled trees indicated as speciation events (S) or duplication events (D). All trees shown as rooted, non-reconciled trees rooted arbitrarily for visualisation. MAO = monoamine oxidase, TBE = transfer bootstrap expectation, UFB = ultrafast bootstrap. Branches colour coded by clade; dark green = monoamine oxidase; light green = monoamine oxidase-like; and black = others and non-opithokont sequences. Source Data are available at [https://figshare.le.ac.uk/articles/dataset/Monoamine_Neuromodulation_is_a_Bilaterian_Innovation_Results/20391477]; in Results/GeneTrees labelled OG_MAO_TBE.tbe.tree and OG_MAO_UFB.treefile; in Results/GeneTrees/Eukaryotes_GeneTrees labelled OG_MAO_EUKIQ.treefile; in Results/Reconciliation/SpongeFirst labelled OG_MAO; in Results/Reconciliation/CtenophoreFirst labelled OG_MAO; and in Results/Reconciliation/Eukaryote labelled OG_MAO.

14A.

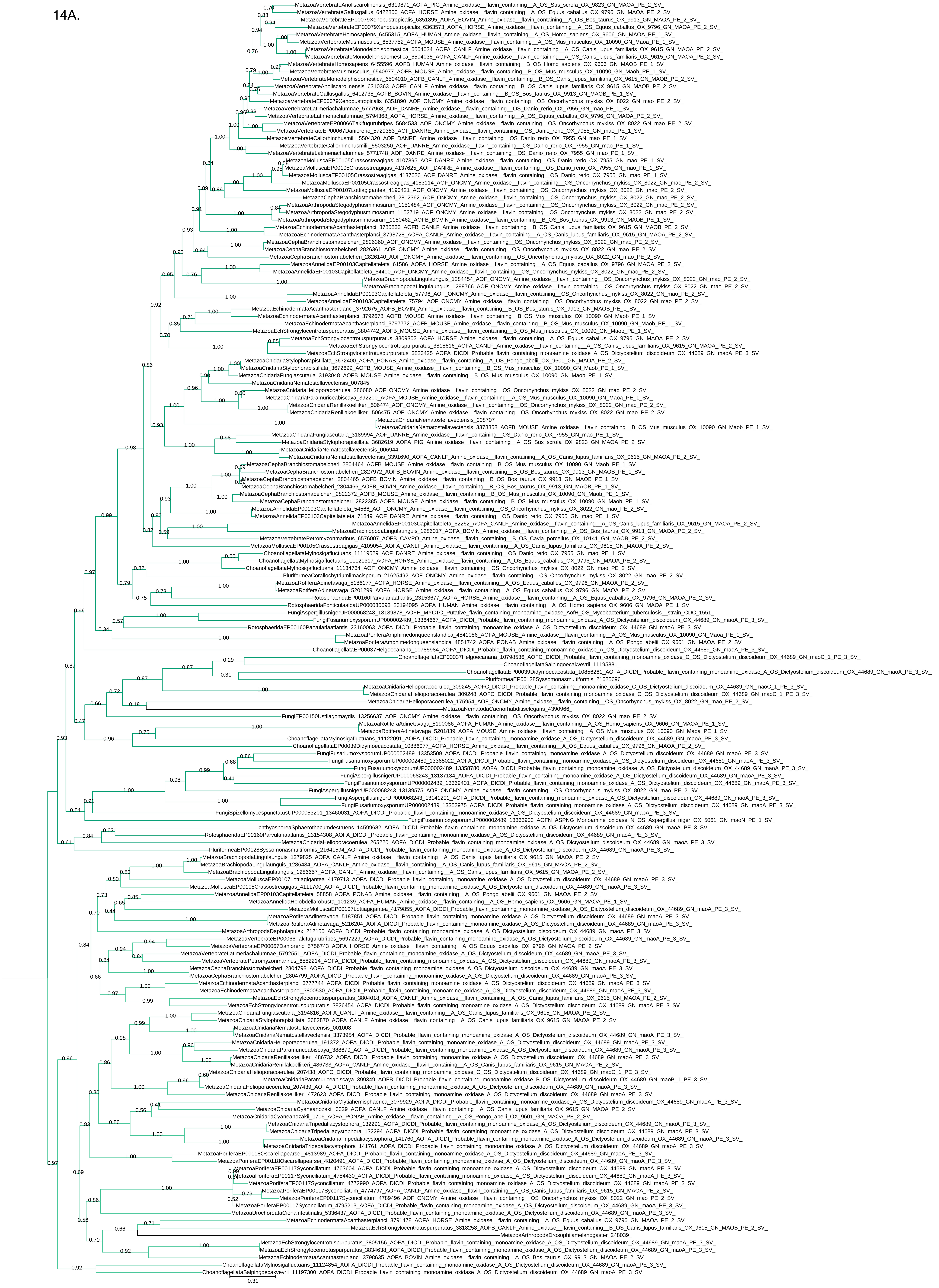

14B.

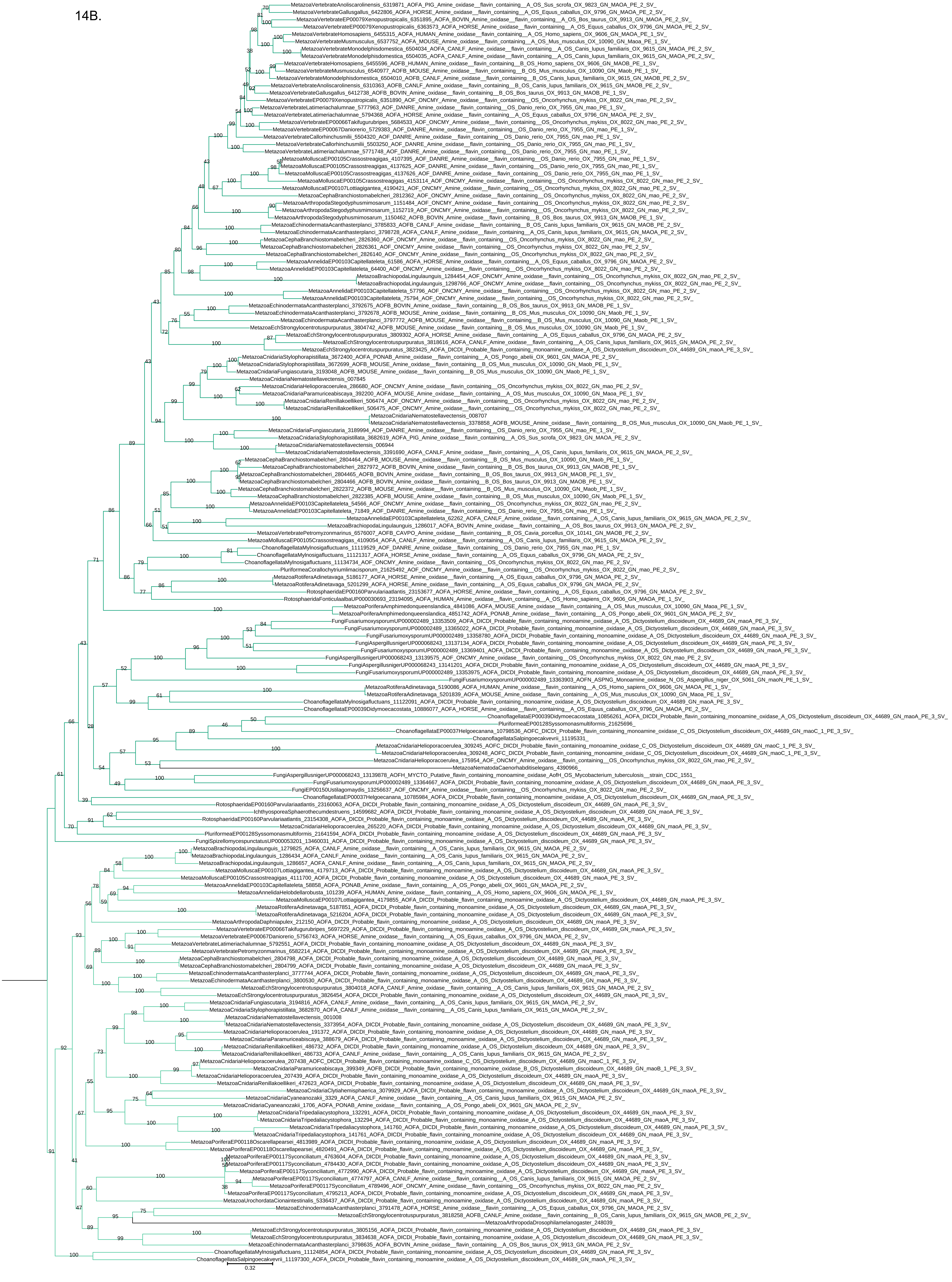
MetazoaVertebrateAnoliscarolinensis_6319871_AOFA_PIG_Amine_oxidase__flavin_containing__A_OS_Sus_scrofa_OX_9823_GN_MAOA_PE_2_SV_
MetazoaVertebrateGallusgallus_6422806_AOFA_HORSE_Amine_oxidase__flavin_containing__A_OS_Equus_caballus_OX_9796_GN_MAOA_PE_2_SV_
MetazoaVertebrateEP00079Xenopustropicalis_6351895_AOFA_BOVIN_Amine_oxidase__flavin_containing__A_OS_Bos_taurus_OX_9913_GN_MAOA_PE_2_SV_
MetazoaVertebrateEP00079Xenopustropicalis_6363573_AOFA_HORSE_Amine_oxidase__flavin_containing__A_OS_Equus_caballus_OX_9796_GN_MAOA_PE_2_SV_
MetazoaVertebrateHomosapiens_6455315_AOFA_HUMAN_Amine_oxidase__flavin_containing__A_OS_Homo_sapiens_OX_9606_GN_MAOA_PE_1_SV_
MetazoaVertebrateMusmusculus_6537752_AOFA_MOUSE_Amine_oxidase__flavin_containing__A_OS_Mus_musculus_OX_10090_GN_Maoa_PE_1_SV_
MetazoaVertebrateMonodelphisdomestica_6504034_AOFA_CANLF_Amine_oxidase__flavin_containing__A_OS_Canis_lupus_familiaris_OX_9615_GN_MAOA_PE_2_SV_
MetazoaVertebrateMonodelphisdomestica_6504035_AOFA_CANLF_Amine_oxidase__flavin_containing__A_OS_Canis_lupus_familiaris_OX_9615_GN_MAOA_PE_2_SV_
MetazoaVertebrateHomosapiens_6455596_AOFB_HUMAN_Amine_oxidase__flavin_containing__B_OS_Homo_sapiens_OX_9606_GN_MAOB_PE_1_SV_
MetazoaVertebrateMusmusculus_6540977_AOFB_MOUSE_Amine_oxidase__flavin_containing__B_OS_Mus_musculus_OX_10090_GN_Maob_PE_1_SV_
MetazoaVertebrateMonodelphisdomestica_6504010_AOFB_CANLF_Amine_oxidase__flavin_containing__B_OS_Canis_lupus_familiaris_OX_9615_GN_MAOB_PE_2_SV_
MetazoaVertebrateAnoliscarolinensis_6310363_AOFB_CANLF_Amine_oxidase__flavin_containing__B_OS_Canis_lupus_familiaris_OX_9615_GN_MAOB_PE_2_SV_
MetazoaVertebrateGallusgallus_6412738_AOFB_BOVIN_Amine_oxidase__flavin_containing__B_OS_Bos_taurus_OX_9913_GN_MAOB_PE_1_SV_
MetazoaVertebrateEP00079Xenopustropicalis_6351890_AOF_ONCMY_Amine_oxidase__flavin_containing__OS_Oncorhynchus_mykiss_OX_8022_GN_mao_PE_2_SV_
MetazoaVertebrateLatimeriachalumnae_5777963_AOF_DANRE_Amine_oxidase__flavin_containing__OS_Danio_rerio_OX_7955_GN_mao_PE_1_SV_
MetazoaVertebrateLatimeriachalumnae_5794368_AOFA_HORSE_Amine_oxidase__flavin_containing__A_OS_Equus_caballus_OX_9796_GN_MAOA_PE_2_SV_
MetazoaVertebrateEP00066Takifugurubripes_5684533_AOF_ONCMY_Amine_oxidase__flavin_containing__OS_Oncorhynchus_mykiss_OX_8022_GN_mao_PE_2_SV_
MetazoaVertebrateEP00067Daniorerio_5729383_AOF_DANRE_Amine_oxidase__flavin_containing__OS_Danio_rerio_OX_7955_GN_mao_PE_1_SV_
MetazoaVertebrateCallorhinchusmilii_5504320_AOF_DANRE_Amine_oxidase__flavin_containing__OS_Danio_rerio_OX_7955_GN_mao_PE_1_SV_
MetazoaVertebrateCallorhinchusmilii_5503250_AOF_DANRE_Amine_oxidase__flavin_containing__OS_Danio_rerio_OX_7955_GN_mao_PE_1_SV_
MetazoaVertebrateLatimeriachalumnae_5771748_AOF_DANRE_Amine_oxidase__flavin_containing__OS_Danio_rerio_OX_7955_GN_mao_PE_1_SV_
MetazoaMolluscaEP00105Crassostreagigas_4107395_AOF_DANRE_Amine_oxidase__flavin_containing__OS_Danio_rerio_OX_7955_GN_mao_PE_1_SV_
MetazoaMolluscaEP00105Crassostreagigas_4137625_AOF_DANRE_Amine_oxidase__flavin_containing__OS_Danio_rerio_OX_7955_GN_mao_PE_1_SV_
MetazoaMolluscaEP00105Crassostreagigas_4137626_AOF_DANRE_Amine_oxidase__flavin_containing__OS_Danio_rerio_OX_7955_GN_mao_PE_1_SV_
MetazoaMolluscaEP00105Crassostreagigas_4153114_AOF_ONCMY_Amine_oxidase__flavin_containing__OS_Oncorhynchus_mykiss_OX_8022_GN_mao_PE_2_SV_
MetazoaMolluscaEP00107Lottiagigantea_4190421_AOF_ONCMY_Amine_oxidase__flavin_containing__OS_Oncorhynchus_mykiss_OX_8022_GN_mao_PE_2_SV_
MetazoaCephaBranchiostomabelcheri_2812362_AOF_ONCMY_Amine_oxidase__flavin_containing__OS_Oncorhynchus_mykiss_OX_8022_GN_mao_PE_2_SV_
MetazoaArthropodaStegodyphusmimosarum_1151484_AOF_ONCMY_Amine_oxidase__flavin_containing__OS_Oncorhynchus_mykiss_OX_8022_GN_mao_PE_2_SV_
MetazoaArthropodaStegodyphusmimosarum_1152719_AOF_ONCMY_Amine_oxidase__flavin_containing__OS_Oncorhynchus_mykiss_OX_8022_GN_mao_PE_2_SV_
MetazoaArthropodaStegodyphusmimosarum_1150462_AOFB_BOVIN_Amine_oxidase__flavin_containing__B_OS_Bos_taurus_OX_9913_GN_MAOB_PE_1_SV_
MetazoaEchinodermataAcanthasterplanci_3785833_AOFB_CANLF_Amine_oxidase__flavin_containing__B_OS_Canis_lupus_familiaris_OX_9615_GN_MAOB_PE_2_SV_
MetazoaEchinodermataAcanthasterplanci_3798728_AOFA_CANLF_Amine_oxidase__flavin_containing__A_OS_Canis_lupus_familiaris_OX_9615_GN_MAOA_PE_2_SV_
MetazoaCephaBranchiostomabelcheri_2826360_AOF_ONCMY_Amine_oxidase__flavin_containing__OS_Oncorhynchus_mykiss_OX_8022_GN_mao_PE_2_SV_
MetazoaCephaBranchiostomabelcheri_2826361_AOF_ONCMY_Amine_oxidase__flavin_containing__OS_Oncorhynchus_mykiss_OX_8022_GN_mao_PE_2_SV_
MetazoaCephaBranchiostomabelcheri_2826140_AOF_ONCMY_Amine_oxidase__flavin_containing__OS_Oncorhynchus_mykiss_OX_8022_GN_mao_PE_2_SV_
MetazoaAnnelidaEP00103Capitellateleta_61586_AOFA_HORSE_Amine_oxidase__flavin_containing__A_OS_Equus_caballus_OX_9796_GN_MAOA_PE_2_SV_
MetazoaAnnelidaEP00103Capitellateleta_64400_AOF_ONCMY_Amine_oxidase__flavin_containing__OS_Oncorhynchus_mykiss_OX_8022_GN_mao_PE_2_SV_
MetazoaBrachiopodaLingulaunguis_1284454_AOF_ONCMY_Amine_oxidase__flavin_containing__OS_Oncorhynchus_mykiss_OX_8022_GN_mao_PE_2_SV_
MetazoaBrachiopodaLingulaunguis_1298766_AOF_ONCMY_Amine_oxidase__flavin_containing__OS_Oncorhynchus_mykiss_OX_8022_GN_mao_PE_2_SV_
MetazoaAnnelidaEP00103Capitellateleta_57796_AOF_ONCMY_Amine_oxidase__flavin_containing__OS_Oncorhynchus_mykiss_OX_8022_GN_mao_PE_2_SV_
MetazoaAnnelidaEP00103Capitellateleta_75794_AOF_ONCMY_Amine_oxidase__flavin_containing__OS_Oncorhynchus_mykiss_OX_8022_GN_mao_PE_2_SV_
MetazoaEchinodermataAcanthasterplanci_3792675_AOFB_BOVIN_Amine_oxidase__flavin_containing__B_OS_Bos_taurus_OX_9913_GN_MAOB_PE_1_SV_
MetazoaEchinodermataAcanthasterplanci_3792678_AOFB_MOUSE_Amine_oxidase__flavin_containing__B_OS_Mus_musculus_OX_10090_GN_Maob_PE_1_SV_
MetazoaEchinodermataAcanthasterplanci_3797772_AOFB_MOUSE_Amine_oxidase__flavin_containing__B_OS_Mus_musculus_OX_10090_GN_Maob_PE_1_SV_
MetazoaEchStrongylocentrotuspurpuratus_3804742_AOFB_MOUSE_Amine_oxidase__flavin_containing__B_OS_Mus_musculus_OX_10090_GN_Maob_PE_1_SV_
MetazoaEchStrongylocentrotuspurpuratus_3809302_AOFA_HORSE_Amine_oxidase__flavin_containing__A_OS_Equus_caballus_OX_9796_GN_MAOA_PE_2_SV_
MetazoaEchStrongylocentrotuspurpuratus_3818616_AOFA_CANLF_Amine_oxidase__flavin_containing__A_OS_Canis_lupus_familiaris_OX_9615_GN_MAOA_PE_2_SV_
MetazoaEchStrongylocentrotuspurpuratus_3823425_AOFA_DICDI_Probable_flavin_containing_monoamine_oxidase_A_OS_Dictyostelium_discoideum_OX_44689_GN_maoA_PE_3_SV_
MetazoaCnidariaStylophorapistillata_3672400_AOFA_PONAB_Amine_oxidase__flavin_containing__A_OS_Pongo_abelii_OX_9601_GN_MAOA_PE_2_SV_
MetazoaCnidariaStylophorapistillata_3672699_AOFB_MOUSE_Amine_oxidase__flavin_containing__B_OS_Mus_musculus_OX_10090_GN_Maob_PE_1_SV_
MetazoaCnidariaFungiascutaria_3193048_AOFB_MOUSE_Amine_oxidase__flavin_containing__B_OS_Mus_musculus_OX_10090_GN_Maob_PE_1_SV_
MetazoaCnidariaNematostellavectensis_007845
MetazoaCnidariaHelioporacoerulea_286680_AOF_ONCMY_Amine_oxidase__flavin_containing__OS_Oncorhynchus_mykiss_OX_8022_GN_mao_PE_2_SV_
MetazoaCnidariaParamuriceabiscaya_392200_AOFA_MOUSE_Amine_oxidase__flavin_containing__A_OS_Mus_musculus_OX_10090_GN_Maoa_PE_1_SV_
MetazoaCnidariaRenillakoellikeri_506474_AOF_ONCMY_Amine_oxidase__flavin_containing__OS_Oncorhynchus_mykiss_OX_8022_GN_mao_PE_2_SV_
MetazoaCnidariaRenillakoellikeri_506475_AOF_ONCMY_Amine_oxidase__flavin_containing__OS_Oncorhynchus_mykiss_OX_8022_GN_mao_PE_2_SV_
MetazoaCnidariaNematostellavectensis_008707
MetazoaCnidariaNematostellavectensis_3378858_AOFB_MOUSE_Amine_oxidase__flavin_containing__B_OS_Mus_musculus_OX_10090_GN_Maob_PE_1_SV_
MetazoaCnidariaFungiascutaria_3189994_AOF_DANRE_Amine_oxidase__flavin_containing__OS_Danio_rerio_OX_7955_GN_mao_PE_1_SV_
MetazoaCnidariaStylophorapistillata_3682619_AOFA_PIG_Amine_oxidase__flavin_containing__A_OS_Sus_scrofa_OX_9823_GN_MAOA_PE_2_SV_
MetazoaCnidariaNematostellavectensis_006944
MetazoaCnidariaNematostellavectensis_3391690_AOFA_CANLF_Amine_oxidase__flavin_containing__A_OS_Canis_lupus_familiaris_OX_9615_GN_MAOA_PE_2_SV_
MetazoaCephaBranchiostomabelcheri_2804464_AOFB_MOUSE_Amine_oxidase__flavin_containing__B_OS_Mus_musculus_OX_10090_GN_Maob_PE_1_SV_
MetazoaCephaBranchiostomabelcheri_2827972_AOFB_BOVIN_Amine_oxidase__flavin_containing__B_OS_Bos_taurus_OX_9913_GN_MAOB_PE_1_SV_
MetazoaCephaBranchiostomabelcheri_2804465_AOFB_BOVIN_Amine_oxidase__flavin_containing__B_OS_Bos_taurus_OX_9913_GN_MAOB_PE_1_SV_
MetazoaCephaBranchiostomabelcheri_2804466_AOFB_BOVIN_Amine_oxidase__flavin_containing__B_OS_Bos_taurus_OX_9913_GN_MAOB_PE_1_SV_
MetazoaCephaBranchiostomabelcheri_2822372_AOFB_MOUSE_Amine_oxidase__flavin_containing__B_OS_Mus_musculus_OX_10090_GN_Maob_PE_1_SV_
MetazoaCephaBranchiostomabelcheri_2822385_AOFB_MOUSE_Amine_oxidase__flavin_containing__B_OS_Mus_musculus_OX_10090_GN_Maob_PE_1_SV_
MetazoaAnnelidaEP00103Capitellateleta_54566_AOF_ONCMY_Amine_oxidase__flavin_containing__OS_Oncorhynchus_mykiss_OX_8022_GN_mao_PE_2_SV_
MetazoaAnnelidaEP00103Capitellateleta_71849_AOF_DANRE_Amine_oxidase__flavin_containing__OS_Danio_rerio_OX_7955_GN_mao_PE_1_SV_
MetazoaAnnelidaEP00103Capitellateleta_62262_AOFA_CANLF_Amine_oxidase__flavin_containing__A_OS_Canis_lupus_familiaris_OX_9615_GN_MAOA_PE_2_SV_
MetazoaBrachiopodaLingulaunguis_1286017_AOFA_BOVIN_Amine_oxidase__flavin_containing__A_OS_Bos_taurus_OX_9913_GN_MAOA_PE_2_SV_
MetazoaVertebratePetromyzonmarinus_6576007_AOFB_CAVPO_Amine_oxidase__flavin_containing__B_OS_Cavia_porcellus_OX_10141_GN_MAOB_PE_2_SV_
MetazoaMolluscaEP00105Crassostreagigas_4109054_AOFA_CANLF_Amine_oxidase__flavin_containing__A_OS_Canis_lupus_familiaris_OX_9615_GN_MAOA_PE_2_SV_
ChoanoflagellataMylnosigafluctuans_11119529_AOF_DANRE_Amine_oxidase__flavin_containing__OS_Danio_rerio_OX_7955_GN_mao_PE_1_SV_
ChoanoflagellataMylnosigafluctuans_11121317_AOFA_HORSE_Amine_oxidase__flavin_containing__A_OS_Equus_caballus_OX_9796_GN_MAOA_PE_2_SV_
ChoanoflagellataMylnosigafluctuans_11134734_AOF_ONCMY_Amine_oxidase__flavin_containing__OS_Oncorhynchus_mykiss_OX_8022_GN_mao_PE_2_SV_
PluriformeaCorallochytriumlimacisporum_21625492_AOF_ONCMY_Amine_oxidase__flavin_containing__OS_Oncorhynchus_mykiss_OX_8022_GN_mao_PE_2_SV_
MetazoaRotiferaAdinetavaga_5186177_AOFA_HORSE_Amine_oxidase__flavin_containing__A_OS_Equus_caballus_OX_9796_GN_MAOA_PE_2_SV_
MetazoaRotiferaAdinetavaga_5201299_AOFA_HORSE_Amine_oxidase__flavin_containing__A_OS_Equus_caballus_OX_9796_GN_MAOA_PE_2_SV_
RotosphaeridaEP00160Parvulariaatlantis_23153677_AOFA_HORSE_Amine_oxidase__flavin_containing__A_OS_Equus_caballus_OX_9796_GN_MAOA_PE_2_SV_
RotosphaeridaFonticulaalbaUP000030693_23194095_AOFA_HUMAN_Amine_oxidase__flavin_containing__A_OS_Homo_sapiens_OX_9606_GN_MAOA_PE_1_SV_
MetazoaPoriferaAmphimedonqueenslandica_4841086_AOFA_MOUSE_Amine_oxidase__flavin_containing__A_OS_Mus_musculus_OX_10090_GN_Maoa_PE_1_SV_
MetazoaPoriferaAmphimedonqueenslandica_4851742_AOFA_PONAB_Amine_oxidase__flavin_containing__A_OS_Pongo_abelii_OX_9601_GN_MAOA_PE_2_SV_
FungiFusariumoxysporumUP000002489_13353509_AOFA_DICDI_Probable_flavin_containing_monoamine_oxidase_A_OS_Dictyostelium_discoideum_OX_44689_GN_maoA_PE_3_SV_
FungiFusariumoxysporumUP000002489_13365022_AOFA_DICDI_Probable_flavin_containing_monoamine_oxidase_A_OS_Dictyostelium_discoideum_OX_44689_GN_maoA_PE_3_SV_
FungiFusariumoxysporumUP000002489_13358780_AOFA_DICDI_Probable_flavin_containing_monoamine_oxidase_A_OS_Dictyostelium_discoideum_OX_44689_GN_maoA_PE_3_SV_
FungiAspergillusnigerUP000068243_13137134_AOFA_DICDI_Probable_flavin_containing_monoamine_oxidase_A_OS_Dictyostelium_discoideum_OX_44689_GN_maoA_PE_3_SV_
FungiFusariumoxysporumUP000002489_13369401_AOFA_DICDI_Probable_flavin_containing_monoamine_oxidase_A_OS_Dictyostelium_discoideum_OX_44689_GN_maoA_PE_3_SV_
FungiAspergillusnigerUP000068243_13139575_AOF_ONCMY_Amine_oxidase__flavin_containing__OS_Oncorhynchus_mykiss_OX_8022_GN_mao_PE_2_SV_
FungiAspergillusnigerUP000068243_13141201_AOFA_DICDI_Probable_flavin_containing_monoamine_oxidase_A_OS_Dictyostelium_discoideum_OX_44689_GN_maoA_PE_3_SV_
FungiFusariumoxysporumUP000002489_13353975_AOFA_DICDI_Probable_flavin_containing_monoamine_oxidase_A_OS_Dictyostelium_discoideum_OX_44689_GN_maoA_PE_3_SV_
FungiFusariumoxysporumUP000002489_13363903_AOFN_ASPNG_Monoamine_oxidase_N_OS_Aspergillus_niger_OX_5061_GN_maoN_PE_1_SV_
MetazoaRotiferaAdinetavaga_5190086_AOFA_HUMAN_Amine_oxidase__flavin_containing__A_OS_Homo_sapiens_OX_9606_GN_MAOA_PE_1_SV_
MetazoaRotiferaAdinetavaga_5201839_AOFA_MOUSE_Amine_oxidase__flavin_containing__A_OS_Mus_musculus_OX_10090_GN_Maoa_PE_1_SV_
ChoanoflagellataMylnosigafluctuans_11122091_AOFA_DICDI_Probable_flavin_containing_monoamine_oxidase_A_OS_Dictyostelium_discoideum_OX_44689_GN_maoA_PE_3_SV_
ChoanoflagellataEP00039Didymoecacostata_10886077_AOFA_HORSE_Amine_oxidase__flavin_containing__A_OS_Equus_caballus_OX_9796_GN_MAOA_PE_2_SV_
ChoanoflagellataEP00039Didymoecacostata_10856261_AOFA_DICDI_Probable_flavin_containing_monoamine_oxidase_A_OS_Dictyostelium_discoideum_OX_44689_GN_maoA_PE_3_SV_
PluriformeaEP00128Syssomonasmultiformis_21625696_
ChoanoflagellataEP00037Helgoecanana_10798536_AOFC_DICDI_Probable_flavin_containing_monoamine_oxidase_C_OS_Dictyostelium_discoideum_OX_44689_GN_maoC_1_PE_3_SV_
ChoanoflagellataSalpingoecakvevrii_11195331_
MetazoaCnidariaHelioporacoerulea_309245_AOFC_DICDI_Probable_flavin_containing_monoamine_oxidase_C_OS_Dictyostelium_discoideum_OX_44689_GN_maoC_1_PE_3_SV_
MetazoaCnidariaHelioporacoerulea_309248_AOFC_DICDI_Probable_flavin_containing_monoamine_oxidase_C_OS_Dictyostelium_discoideum_OX_44689_GN_maoC_1_PE_3_SV_
MetazoaCnidariaHelioporacoerulea_175954_AOF_ONCMY_Amine_oxidase__flavin_containing__OS_Oncorhynchus_mykiss_OX_8022_GN_mao_PE_2_SV_
MetazoaNematodaCaenorhabditiselegans_4390966_
FungiAspergillusnigerUP000068243_13139878_AOFH_MYCTO_Putative_flavin_containing_monoamine_oxidase_AofH_OS_Mycobacterium_tuberculosis__strain_CDC_1551_
FungiFusariumoxysporumUP000002489_13364667_AOFA_DICDI_Probable_flavin_containing_monoamine_oxidase_A_OS_Dictyostelium_discoideum_OX_44689_GN_maoA_PE_3_SV_
FungiEP00150Ustilagomaydis_13256637_AOF_ONCMY_Amine_oxidase__flavin_containing__OS_Oncorhynchus_mykiss_OX_8022_GN_mao_PE_2_SV_
ChoanoflagellataEP00037Helgoecanana_10785984_AOFA_DICDI_Probable_flavin_containing_monoamine_oxidase_A_OS_Dictyostelium_discoideum_OX_44689_GN_maoA_PE_3_SV_
RotosphaeridaEP00160Parvulariaatlantis_23160063_AOFA_DICDI_Probable_flavin_containing_monoamine_oxidase_A_OS_Dictyostelium_discoideum_OX_44689_GN_maoA_PE_3_SV_
IchthyosporeaSphaerothecumdestruens_14599682_AOFA_DICDI_Probable_flavin_containing_monoamine_oxidase_A_OS_Dictyostelium_discoideum_OX_44689_GN_maoA_PE_3_SV_
RotosphaeridaEP00160Parvulariaatlantis_23154308_AOFA_DICDI_Probable_flavin_containing_monoamine_oxidase_A_OS_Dictyostelium_discoideum_OX_44689_GN_maoA_PE_3_SV_
MetazoaCnidariaHelioporacoerulea_265220_AOFA_DICDI_Probable_flavin_containing_monoamine_oxidase_A_OS_Dictyostelium_discoideum_OX_44689_GN_maoA_PE_3_SV_
PluriformeaEP00128Syssomonasmultiformis_21641594_AOFA_DICDI_Probable_flavin_containing_monoamine_oxidase_A_OS_Dictyostelium_discoideum_OX_44689_GN_maoA_PE_3_SV_
FungiSpizellomycespunctatusUP000053201_13460031_AOFA_DICDI_Probable_flavin_containing_monoamine_oxidase_A_OS_Dictyostelium_discoideum_OX_44689_GN_maoA_PE_3_SV_
MetazoaBrachiopodaLingulaunguis_1279825_AOFA_CANLF_Amine_oxidase__flavin_containing__A_OS_Canis_lupus_familiaris_OX_9615_GN_MAOA_PE_2_SV_
MetazoaBrachiopodaLingulaunguis_1286434_AOFA_CANLF_Amine_oxidase__flavin_containing__A_OS_Canis_lupus_familiaris_OX_9615_GN_MAOA_PE_2_SV_
MetazoaBrachiopodaLingulaunguis_1286657_AOFA_CANLF_Amine_oxidase__flavin_containing__A_OS_Canis_lupus_familiaris_OX_9615_GN_MAOA_PE_2_SV_
MetazoaMolluscaEP00107Lottiagigantea_4179713_AOFA_DICDI_Probable_flavin_containing_monoamine_oxidase_A_OS_Dictyostelium_discoideum_OX_44689_GN_maoA_PE_3_SV_
MetazoaMolluscaEP00105Crassostreagigas_4111700_AOFA_DICDI_Probable_flavin_containing_monoamine_oxidase_A_OS_Dictyostelium_discoideum_OX_44689_GN_maoA_PE_3_SV_
MetazoaAnnelidaEP00103Capitellateleta_58858_AOFA_PONAB_Amine_oxidase__flavin_containing__A_OS_Pongo_abelii_OX_9601_GN_MAOA_PE_2_SV_
MetazoaAnnelidaHelobdellarobusta_101239_AOFA_HUMAN_Amine_oxidase__flavin_containing__A_OS_Homo_sapiens_OX_9606_GN_MAOA_PE_1_SV_
MetazoaMolluscaEP00107Lottiagigantea_4179855_AOFA_DICDI_Probable_flavin_containing_monoamine_oxidase_A_OS_Dictyostelium_discoideum_OX_44689_GN_maoA_PE_3_SV_
MetazoaRotiferaAdinetavaga_5187851_AOFA_DICDI_Probable_flavin_containing_monoamine_oxidase_A_OS_Dictyostelium_discoideum_OX_44689_GN_maoA_PE_3_SV_
MetazoaRotiferaAdinetavaga_5216204_AOFA_DICDI_Probable_flavin_containing_monoamine_oxidase_A_OS_Dictyostelium_discoideum_OX_44689_GN_maoA_PE_3_SV_
MetazoaArthropodaDaphniapulex_212150_AOFA_DICDI_Probable_flavin_containing_monoamine_oxidase_A_OS_Dictyostelium_discoideum_OX_44689_GN_maoA_PE_3_SV_
MetazoaVertebrateEP00066Takifugurubripes_5697229_AOFA_DICDI_Probable_flavin_containing_monoamine_oxidase_A_OS_Dictyostelium_discoideum_OX_44689_GN_maoA_PE_3_SV_
MetazoaVertebrateEP00067Daniorerio_5756743_AOFA_HORSE_Amine_oxidase__flavin_containing__A_OS_Equus_caballus_OX_9796_GN_MAOA_PE_2_SV_
MetazoaVertebrateLatimeriachalumnae_5792551_AOFA_DICDI_Probable_flavin_containing_monoamine_oxidase_A_OS_Dictyostelium_discoideum_OX_44689_GN_maoA_PE_3_SV_
MetazoaVertebratePetromyzonmarinus_6582214_AOFA_DICDI_Probable_flavin_containing_monoamine_oxidase_A_OS_Dictyostelium_discoideum_OX_44689_GN_maoA_PE_3_SV_
MetazoaCephaBranchiostomabelcheri_2804798_AOFA_DICDI_Probable_flavin_containing_monoamine_oxidase_A_OS_Dictyostelium_discoideum_OX_44689_GN_maoA_PE_3_SV_
MetazoaCephaBranchiostomabelcheri_2804799_AOFA_DICDI_Probable_flavin_containing_monoamine_oxidase_A_OS_Dictyostelium_discoideum_OX_44689_GN_maoA_PE_3_SV_
MetazoaEchinodermataAcanthasterplanci_3777744_AOFA_DICDI_Probable_flavin_containing_monoamine_oxidase_A_OS_Dictyostelium_discoideum_OX_44689_GN_maoA_PE_3_SV_
MetazoaEchinodermataAcanthasterplanci_3800530_AOFA_DICDI_Probable_flavin_containing_monoamine_oxidase_A_OS_Dictyostelium_discoideum_OX_44689_GN_maoA_PE_3_SV_
MetazoaEchStrongylocentrotuspurpuratus_3804018_AOFA_CANLF_Amine_oxidase__flavin_containing__A_OS_Canis_lupus_familiaris_OX_9615_GN_MAOA_PE_2_SV_
MetazoaEchStrongylocentrotuspurpuratus_3826454_AOFA_DICDI_Probable_flavin_containing_monoamine_oxidase_A_OS_Dictyostelium_discoideum_OX_44689_GN_maoA_PE_3_SV_
MetazoaCnidariaFungiascutaria_3194816_AOFA_CANLF_Amine_oxidase__flavin_containing__A_OS_Canis_lupus_familiaris_OX_9615_GN_MAOA_PE_2_SV_
MetazoaCnidariaStylophorapistillata_3682870_AOFA_CANLF_Amine_oxidase__flavin_containing__A_OS_Canis_lupus_familiaris_OX_9615_GN_MAOA_PE_2_SV_
MetazoaCnidariaNematostellavectensis_001008
MetazoaCnidariaNematostellavectensis_3373954_AOFA_DICDI_Probable_flavin_containing_monoamine_oxidase_A_OS_Dictyostelium_discoideum_OX_44689_GN_maoA_PE_3_SV_
MetazoaCnidariaHelioporacoerulea_191372_AOFA_DICDI_Probable_flavin_containing_monoamine_oxidase_A_OS_Dictyostelium_discoideum_OX_44689_GN_maoA_PE_3_SV_
MetazoaCnidariaParamuriceabiscaya_388679_AOFA_DICDI_Probable_flavin_containing_monoamine_oxidase_A_OS_Dictyostelium_discoideum_OX_44689_GN_maoA_PE_3_SV_
MetazoaCnidariaRenillakoellikeri_486732_AOFA_DICDI_Probable_flavin_containing_monoamine_oxidase_A_OS_Dictyostelium_discoideum_OX_44689_GN_maoA_PE_3_SV_
MetazoaCnidariaRenillakoellikeri_486733_AOFA_CANLF_Amine_oxidase__flavin_containing__A_OS_Canis_lupus_familiaris_OX_9615_GN_MAOA_PE_2_SV_
MetazoaCnidariaHelioporacoerulea_207438_AOFC_DICDI_Probable_flavin_containing_monoamine_oxidase_C_OS_Dictyostelium_discoideum_OX_44689_GN_maoC_1_PE_3_SV_
MetazoaCnidariaParamuriceabiscaya_399349_AOFB_DICDI_Probable_flavin_containing_monoamine_oxidase_B_OS_Dictyostelium_discoideum_OX_44689_GN_maoB_1_PE_3_SV_
MetazoaCnidariaHelioporacoerulea_207439_AOFA_DICDI_Probable_flavin_containing_monoamine_oxidase_A_OS_Dictyostelium_discoideum_OX_44689_GN_maoA_PE_3_SV_
MetazoaCnidariaRenillakoellikeri_472623_AOFA_DICDI_Probable_flavin_containing_monoamine_oxidase_A_OS_Dictyostelium_discoideum_OX_44689_GN_maoA_PE_3_SV_
MetazoaCnidariaClytiahemisphaerica_3079929_AOFA_DICDI_Probable_flavin_containing_monoamine_oxidase_A_OS_Dictyostelium_discoideum_OX_44689_GN_maoA_PE_3_SV_
MetazoaCnidariaCyaneanozakii_3329_AOFA_CANLF_Amine_oxidase__flavin_containing__A_OS_Canis_lupus_familiaris_OX_9615_GN_MAOA_PE_2_SV_
MetazoaCnidariaCyaneanozakii_1706_AOFA_PONAB_Amine_oxidase__flavin_containing__A_OS_Pongo_abelii_OX_9601_GN_MAOA_PE_2_SV_
MetazoaCnidariaTripedaliacystophora_132291_AOFA_DICDI_Probable_flavin_containing_monoamine_oxidase_A_OS_Dictyostelium_discoideum_OX_44689_GN_maoA_PE_3_SV_
MetazoaCnidariaTripedaliacystophora_132294_AOFA_DICDI_Probable_flavin_containing_monoamine_oxidase_A_OS_Dictyostelium_discoideum_OX_44689_GN_maoA_PE_3_SV_
MetazoaCnidariaTripedaliacystophora_141760_AOFA_DICDI_Probable_flavin_containing_monoamine_oxidase_A_OS_Dictyostelium_discoideum_OX_44689_GN_maoA_PE_3_SV_
MetazoaCnidariaTripedaliacystophora_141761_AOFA_DICDI_Probable_flavin_containing_monoamine_oxidase_A_OS_Dictyostelium_discoideum_OX_44689_GN_maoA_PE_3_SV_
MetazoaPoriferaEP00118Oscarellapearsei_4813989_AOFA_DICDI_Probable_flavin_containing_monoamine_oxidase_A_OS_Dictyostelium_discoideum_OX_44689_GN_maoA_PE_3_SV_
MetazoaPoriferaEP00118Oscarellapearsei_4820491_AOFA_DICDI_Probable_flavin_containing_monoamine_oxidase_A_OS_Dictyostelium_discoideum_OX_44689_GN_maoA_PE_3_SV_
MetazoaPoriferaEP00117Syconciliatum_4763604_AOFA_DICDI_Probable_flavin_containing_monoamine_oxidase_A_OS_Dictyostelium_discoideum_OX_44689_GN_maoA_PE_3_SV_
MetazoaPoriferaEP00117Syconciliatum_4784430_AOFA_DICDI_Probable_flavin_containing_monoamine_oxidase_A_OS_Dictyostelium_discoideum_OX_44689_GN_maoA_PE_3_SV_
MetazoaPoriferaEP00117Syconciliatum_4772990_AOFA_DICDI_Probable_flavin_containing_monoamine_oxidase_A_OS_Dictyostelium_discoideum_OX_44689_GN_maoA_PE_3_SV_
MetazoaPoriferaEP00117Syconciliatum_4774797_AOFA_CANLF_Amine_oxidase__flavin_containing__A_OS_Canis_lupus_familiaris_OX_9615_GN_MAOA_PE_2_SV_
MetazoaPoriferaEP00117Syconciliatum_4789496_AOF_ONCMY_Amine_oxidase__flavin_containing__OS_Oncorhynchus_mykiss_OX_8022_GN_mao_PE_2_SV_
MetazoaPoriferaEP00117Syconciliatum_4795213_AOFA_DICDI_Probable_flavin_containing_monoamine_oxidase_A_OS_Dictyostelium_discoideum_OX_44689_GN_maoA_PE_3_SV_
MetazoaUrochordataCionaintestinalis_5336437_AOFA_DICDI_Probable_flavin_containing_monoamine_oxidase_A_OS_Dictyostelium_discoideum_OX_44689_GN_maoA_PE_3_SV_
MetazoaEchinodermataAcanthasterplanci_3791478_AOFA_HORSE_Amine_oxidase__flavin_containing__A_OS_Equus_caballus_OX_9796_GN_MAOA_PE_2_SV_
MetazoaEchStrongylocentrotuspurpuratus_3818258_AOFB_CANLF_Amine_oxidase__flavin_containing__B_OS_Canis_lupus_familiaris_OX_9615_GN_MAOB_PE_2_SV_
MetazoaArthropodaDrosophilamelanogaster_248039_
MetazoaEchStrongylocentrotuspurpuratus_3805156_AOFA_DICDI_Probable_flavin_containing_monoamine_oxidase_A_OS_Dictyostelium_discoideum_OX_44689_GN_maoA_PE_3_SV_
MetazoaEchStrongylocentrotuspurpuratus_3834638_AOFA_DICDI_Probable_flavin_containing_monoamine_oxidase_A_OS_Dictyostelium_discoideum_OX_44689_GN_maoA_PE_3_SV_
MetazoaEchinodermataAcanthasterplanci_3798635_AOFA_BOVIN_Amine_oxidase__flavin_containing__A_OS_Bos_taurus_OX_9913_GN_MAOA_PE_2_SV_
ChoanoflagellataMylnosigafluctuans_11124854_AOFA_DICDI_Probable_flavin_containing_monoamine_oxidase_A_OS_Dictyostelium_discoideum_OX_44689_GN_maoA_PE_3_SV_
ChoanoflagellataSalpingoecakvevrii_11197300_AOFA_DICDI_Probable_flavin_containing_monoamine_oxidase_A_OS_Dictyostelium_discoideum_OX_44689_GN_maoA_PE_3_SV_
0.32

14C.

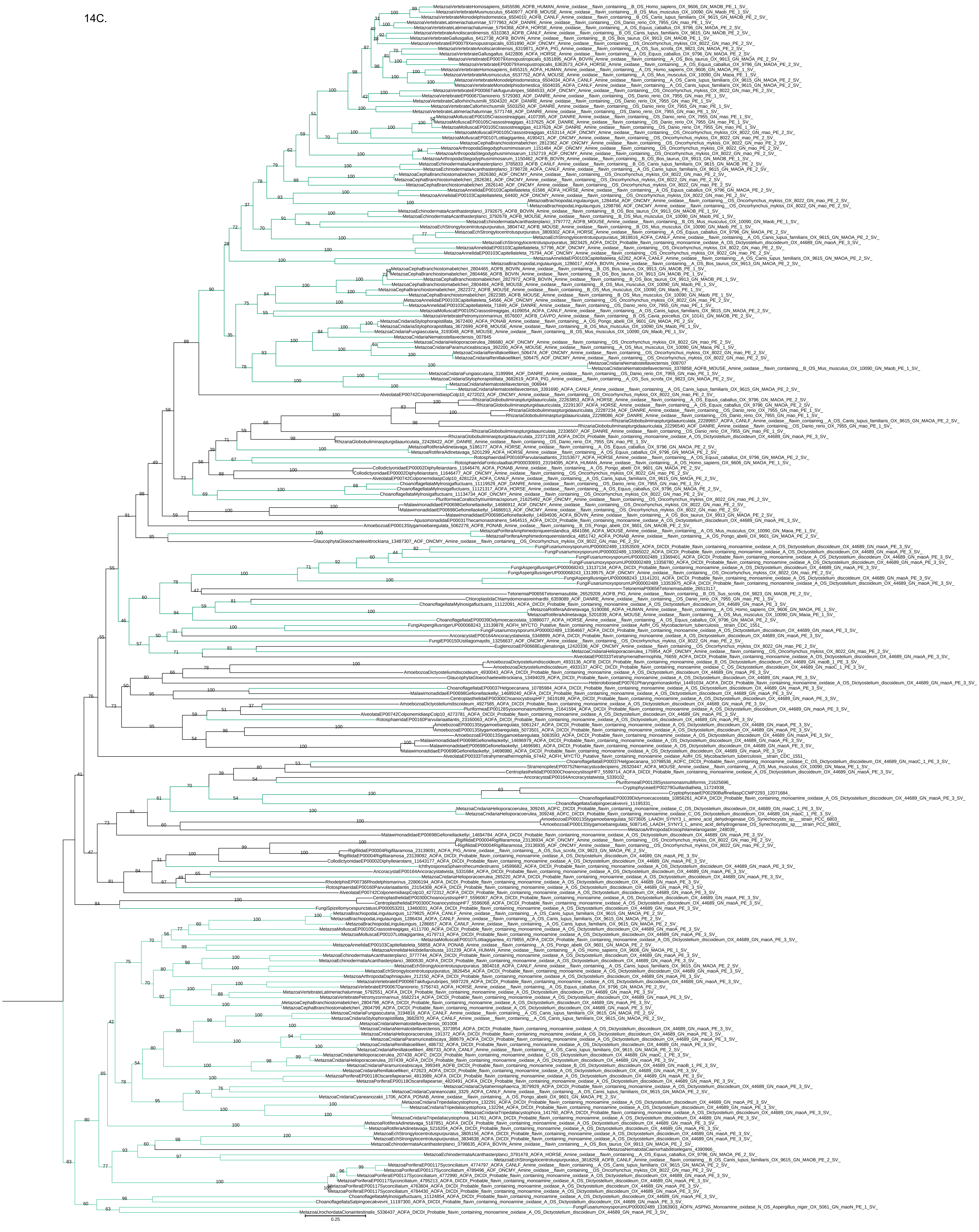

14D.

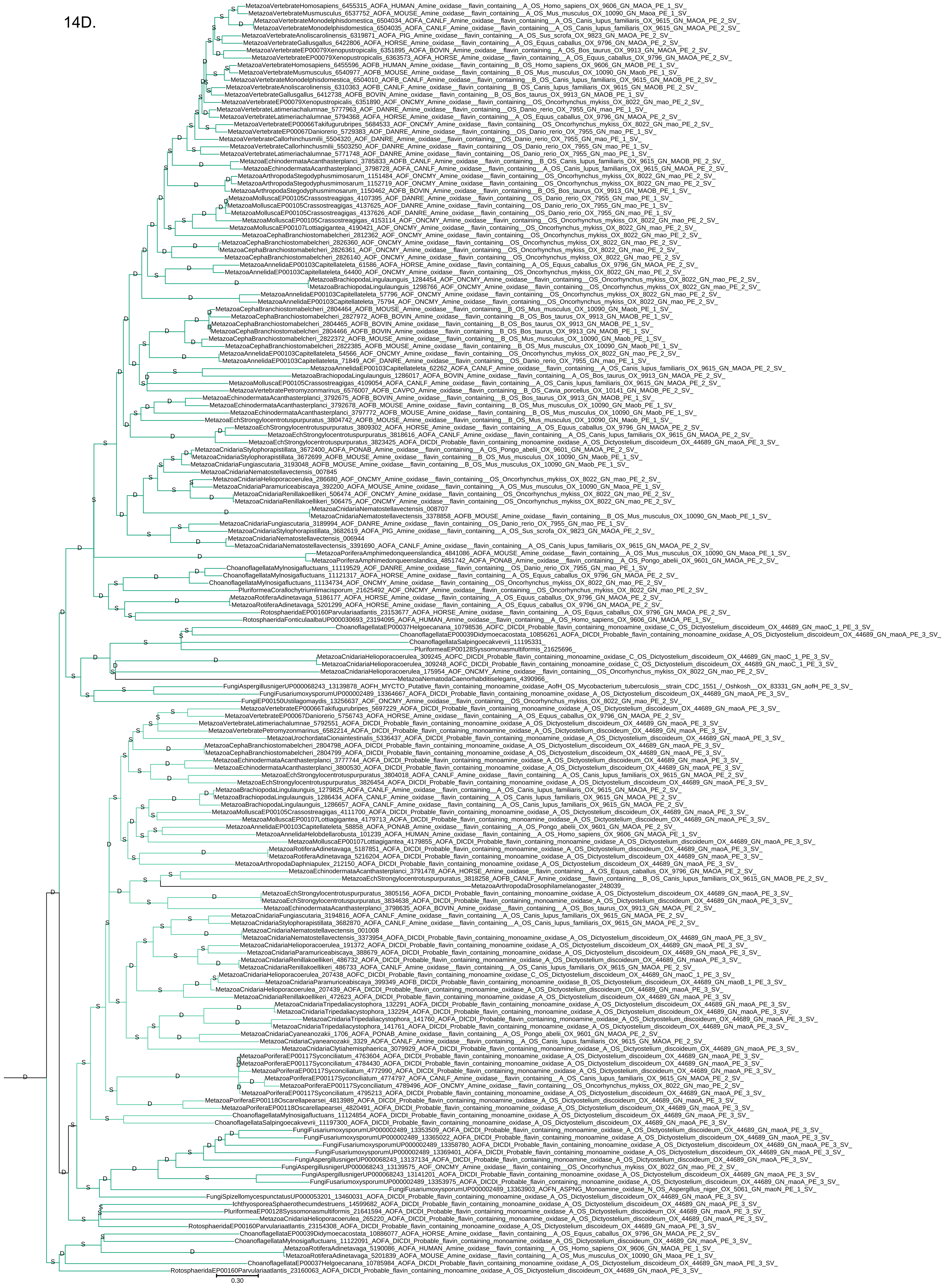

14E.

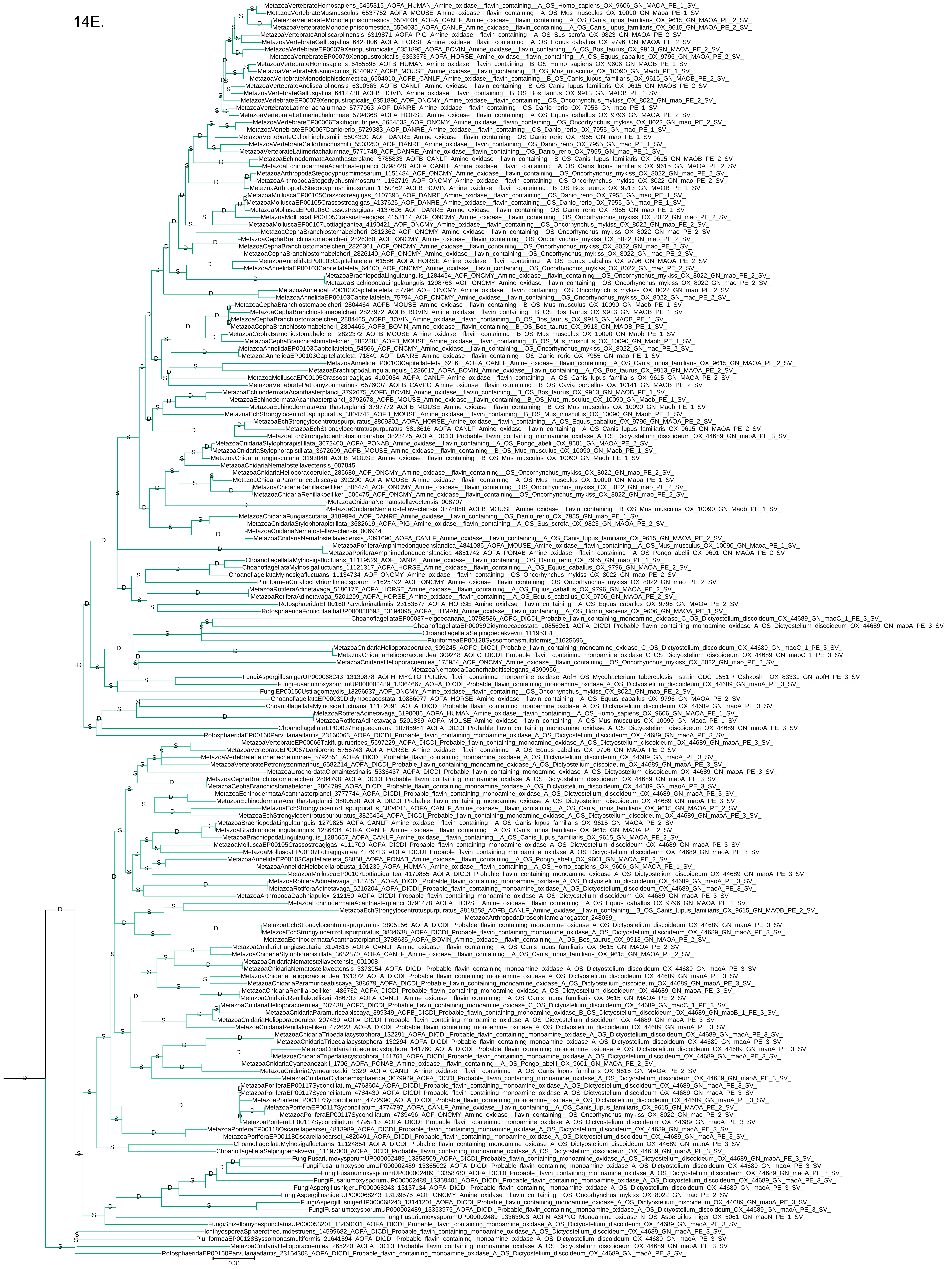

14F.

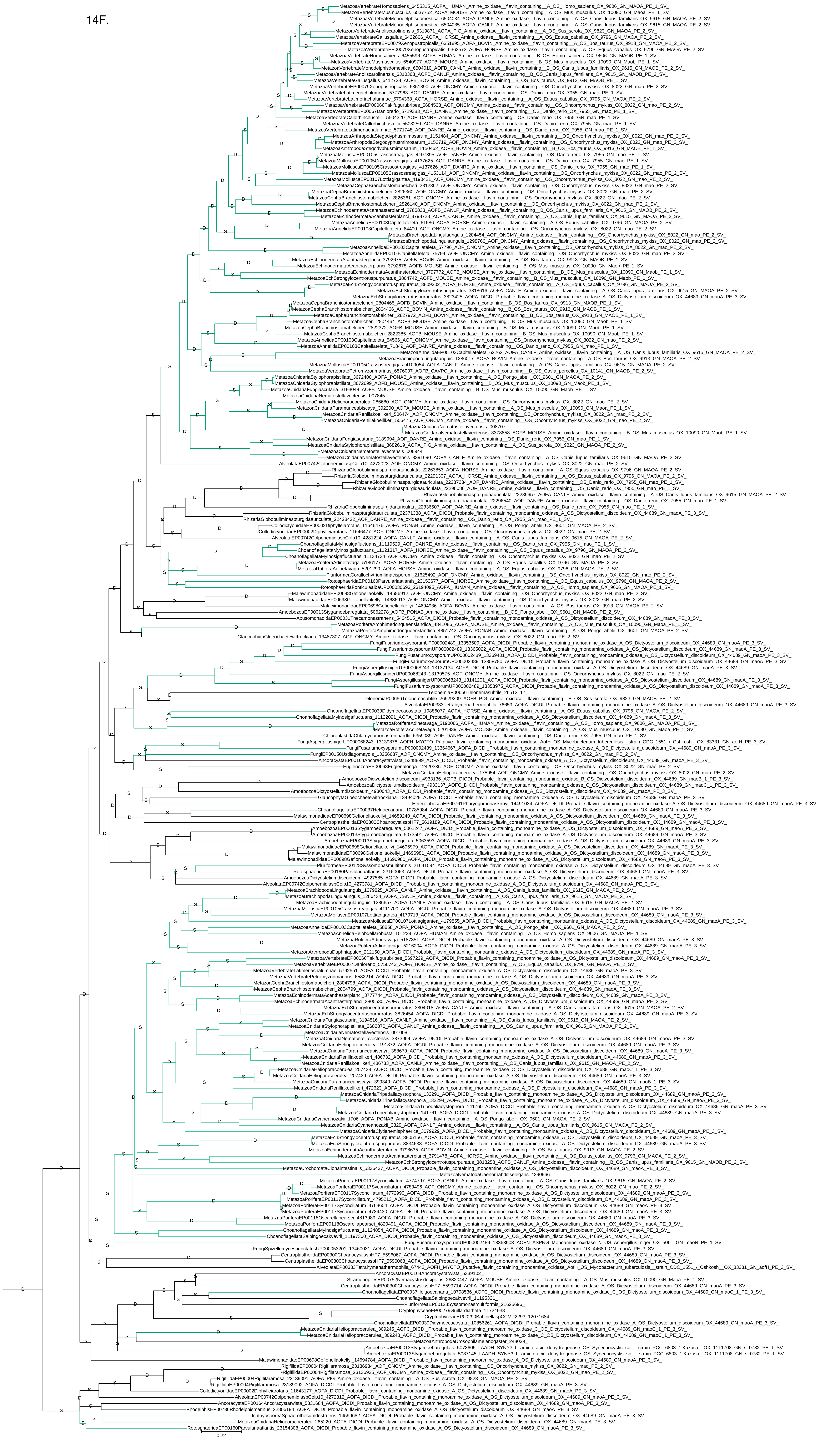

Supplementary Figure 15: Evolution of 5HT3Rs. Simplified illustration of the gene tree to species tree reconciliation. Silhouettes obtained from Phylopic.org. Silhouette images are by Andrew R. Gehrke (*Hofstenia miamia*); Daniel Jaron (*Mus musculus*); Mali'o Kodis, photograph by Ching (http://www.flickr.com/photos/36302473@N03/) (*Chrysaora fuscescens*); Mario Quevedo (Asteriidae); Oliver Voigt (*Trichoplax adhaerens*); Ramiro Morales-Hojas (*Drosophila americana*); Steven Haddock, Jellywatch.org (*Hormiphora californensis*); and Tess Linden (*Salpingoeca rosetta*). 5HT3R = 5-hydroxytryptamine receptor 3.

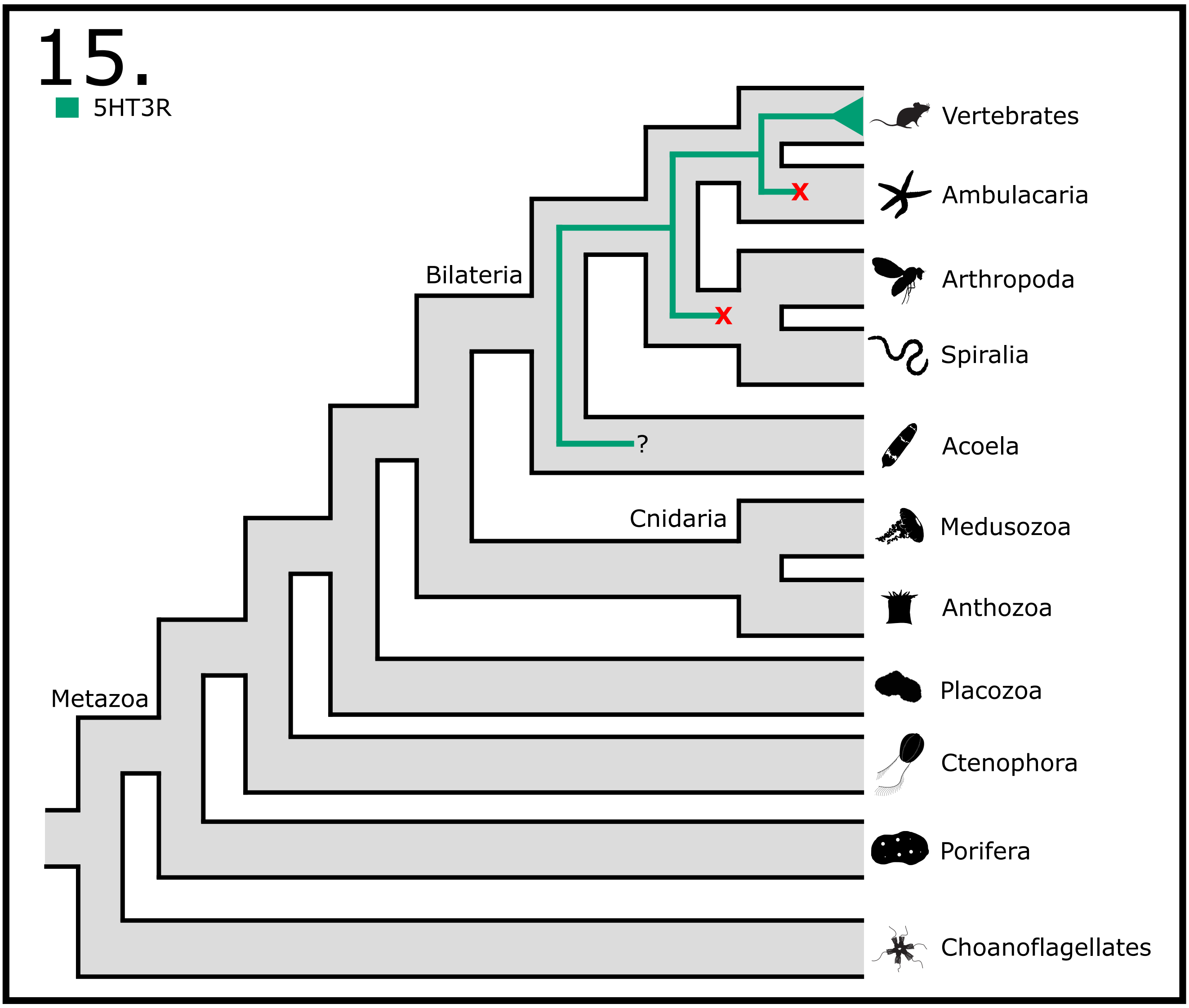
15.
5HT3R
Vertebrates
Ambulacaria
Arthropoda
Spiralia
Acoela
Medusozoa
Anthozoa
Placozoa
Ctenophora
Porifera
Choanoflagellates
Bilateria
Cnidaria
Metazoa
?

Supplementary Figure 16: Analysis of 5HT3Rs. A) TBE tree with TBE score supports. B) Maximum likelihood tree with ultrafast bootstrap proportion supports. C) Maximum likelihood tree including all eukaryotic sequences with ultrafast bootstrap proportion supports. D) Reconciliation using sponge-first hypothesis. E) Reconciliation using ctenophore-first hypothesis. F) Reconciliation of the UFB tree including all eukaryotic sequences. Nodes in reconciled trees indicated as speciation events (S) or duplication events (D). All trees shown as rooted, non-reconciled trees rooted arbitrarily for visualisation. 5HT3R = 5-hydroxytryptamine receptor 3, TBE = transfer bootstrap expectation, UFB = ultrafast bootstrap. Branches colour coded by sequence annotation or clade; green = 5-hydroxytryptamine receptor 3; and black = others. Source Data are available at [https://figshare.le.ac.uk/articles/dataset/Monoamine_Neuromodulation_is_a_Bilaterian_Innovation_Results/20391477]; in Results/GeneTrees labelled OG_5HT3R_TBE.tbe.tree and OG_5HT3R_UFB.treefile; in Results/GeneTrees/Eukaryotes_GeneTrees labelled OG_5HT3R_EUKIQ.treefile; in Results/Reconciliation/SpongeFirst labelled OG_5HT3R; in Results/Reconciliation/CtenophoreFirst labelled OG_5HT3R; and in Results/Reconciliation/Eukaryote labelled OG_5HT3R.

16A.

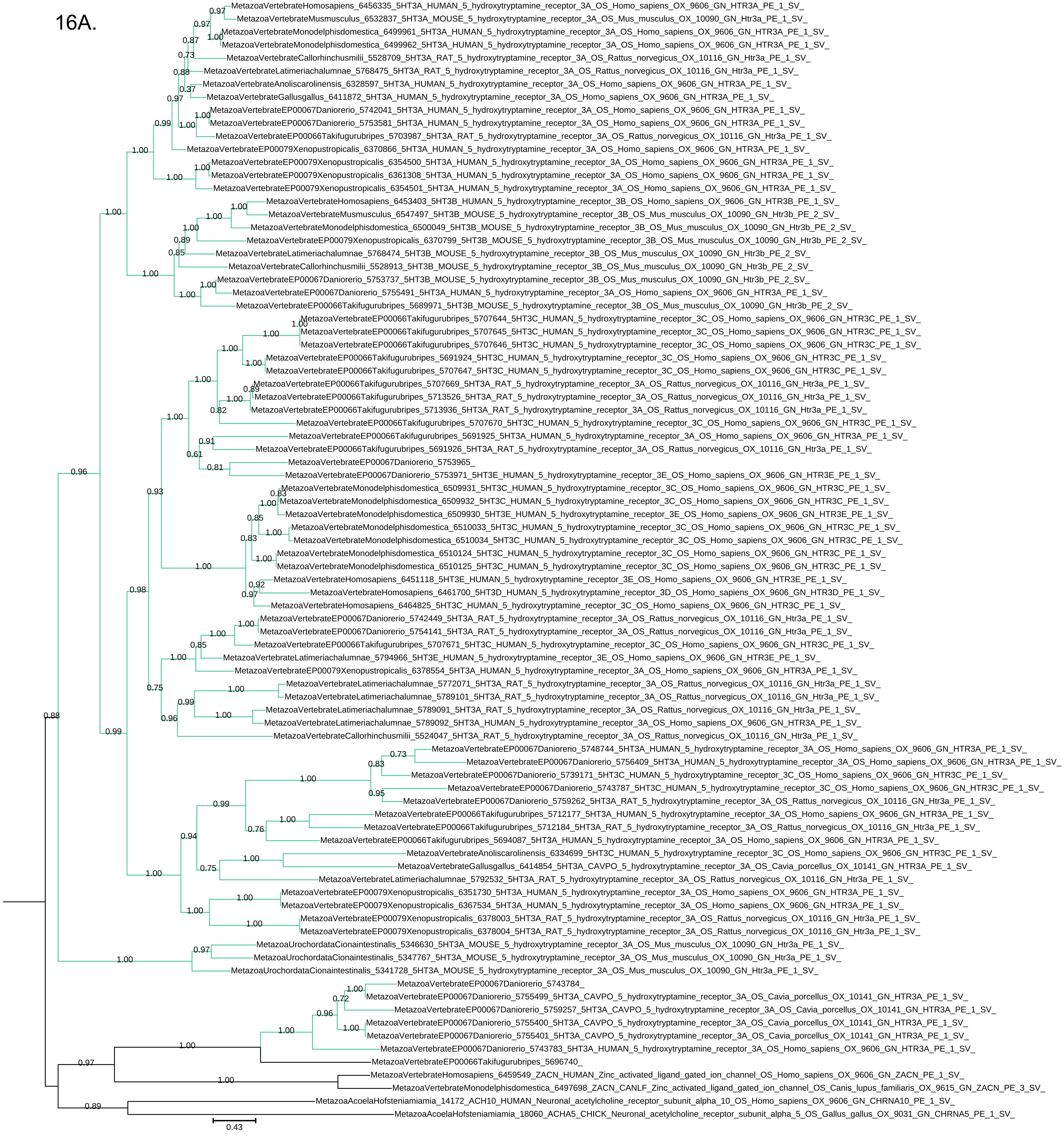
MetazoaVertebrateHomosapiens_6456335_5HT3A_HUMAN_5_hydroxytryptamine_receptor_3A_OS_Homo_sapiens_OX_9606_GN_HTR3A_PE_1_SV_
MetazoaVertebrateMusmusculus_6532837_5HT3A_MOUSE_5_hydroxytryptamine_receptor_3A_OS_Mus_musculus_OX_10090_GN_Htr3a_PE_1_SV_
MetazoaVertebrateMonodelphisdomestica_6499961_5HT3A_HUMAN_5_hydroxytryptamine_receptor_3A_OS_Homo_sapiens_OX_9606_GN_HTR3A_PE_1_SV_
MetazoaVertebrateMonodelphisdomestica_6499962_5HT3A_HUMAN_5_hydroxytryptamine_receptor_3A_OS_Homo_sapiens_OX_9606_GN_HTR3A_PE_1_SV_
MetazoaVertebrateCallorhinchusmilii_5528709_5HT3A_RAT_5_hydroxytryptamine_receptor_3A_OS_Rattus_norvegicus_OX_10116_GN_Htr3a_PE_1_SV_
MetazoaVertebrateLatimeriachalumnae_5768475_5HT3A_RAT_5_hydroxytryptamine_receptor_3A_OS_Rattus_norvegicus_OX_10116_GN_Htr3a_PE_1_SV_
MetazoaVertebrateAnoliscarolinensis_6328597_5HT3A_HUMAN_5_hydroxytryptamine_receptor_3A_OS_Homo_sapiens_OX_9606_GN_HTR3A_PE_1_SV_
MetazoaVertebrateGallusgallus_6411872_5HT3A_HUMAN_5_hydroxytryptamine_receptor_3A_OS_Homo_sapiens_OX_9606_GN_HTR3A_PE_1_SV_
MetazoaVertebrateEP00067Daniorerio_5742041_5HT3A_HUMAN_5_hydroxytryptamine_receptor_3A_OS_Homo_sapiens_OX_9606_GN_HTR3A_PE_1_SV_
MetazoaVertebrateEP00067Daniorerio_5753581_5HT3A_HUMAN_5_hydroxytryptamine_receptor_3A_OS_Homo_sapiens_OX_9606_GN_HTR3A_PE_1_SV_
MetazoaVertebrateEP00066Takifugurubripes_5703987_5HT3A_RAT_5_hydroxytryptamine_receptor_3A_OS_Rattus_norvegicus_OX_10116_GN_Htr3a_PE_1_SV_
MetazoaVertebrateEP00079Xenopustropicalis_6370866_5HT3A_HUMAN_5_hydroxytryptamine_receptor_3A_OS_Homo_sapiens_OX_9606_GN_HTR3A_PE_1_SV_
MetazoaVertebrateEP00079Xenopustropicalis_6354500_5HT3A_HUMAN_5_hydroxytryptamine_receptor_3A_OS_Homo_sapiens_OX_9606_GN_HTR3A_PE_1_SV_
MetazoaVertebrateEP00079Xenopustropicalis_6361308_5HT3A_HUMAN_5_hydroxytryptamine_receptor_3A_OS_Homo_sapiens_OX_9606_GN_HTR3A_PE_1_SV_
MetazoaVertebrateEP00079Xenopustropicalis_6354501_5HT3A_HUMAN_5_hydroxytryptamine_receptor_3A_OS_Homo_sapiens_OX_9606_GN_HTR3A_PE_1_SV_
MetazoaVertebrateHomosapiens_6453403_5HT3B_HUMAN_5_hydroxytryptamine_receptor_3B_OS_Homo_sapiens_OX_9606_GN_HTR3B_PE_1_SV_
MetazoaVertebrateMusmusculus_6547497_5HT3B_MOUSE_5_hydroxytryptamine_receptor_3B_OS_Mus_musculus_OX_10090_GN_Htr3b_PE_2_SV_
MetazoaVertebrateMonodelphisdomestica_6500049_5HT3B_MOUSE_5_hydroxytryptamine_receptor_3B_OS_Mus_musculus_OX_10090_GN_Htr3b_PE_2_SV_
MetazoaVertebrateEP00079Xenopustropicalis_6370799_5HT3B_MOUSE_5_hydroxytryptamine_receptor_3B_OS_Mus_musculus_OX_10090_GN_Htr3b_PE_2_SV_
MetazoaVertebrateLatimeriachalumnae_5768474_5HT3B_MOUSE_5_hydroxytryptamine_receptor_3B_OS_Mus_musculus_OX_10090_GN_Htr3b_PE_2_SV_
MetazoaVertebrateCallorhinchusmilii_5528913_5HT3B_MOUSE_5_hydroxytryptamine_receptor_3B_OS_Mus_musculus_OX_10090_GN_Htr3b_PE_2_SV_
MetazoaVertebrateEP00067Daniorerio_5753737_5HT3B_MOUSE_5_hydroxytryptamine_receptor_3B_OS_Mus_musculus_OX_10090_GN_Htr3b_PE_2_SV_
MetazoaVertebrateEP00067Daniorerio_5755491_5HT3A_HUMAN_5_hydroxytryptamine_receptor_3A_OS_Homo_sapiens_OX_9606_GN_HTR3A_PE_1_SV_
MetazoaVertebrateEP00066Takifugurubripes_5689971_5HT3B_MOUSE_5_hydroxytryptamine_receptor_3B_OS_Mus_musculus_OX_10090_GN_Htr3b_PE_2_SV_
MetazoaVertebrateEP00066Takifugurubripes_5707644_5HT3C_HUMAN_5_hydroxytryptamine_receptor_3C_OS_Homo_sapiens_OX_9606_GN_HTR3C_PE_1_SV_
MetazoaVertebrateEP00066Takifugurubripes_5707645_5HT3C_HUMAN_5_hydroxytryptamine_receptor_3C_OS_Homo_sapiens_OX_9606_GN_HTR3C_PE_1_SV_
MetazoaVertebrateEP00066Takifugurubripes_5707646_5HT3C_HUMAN_5_hydroxytryptamine_receptor_3C_OS_Homo_sapiens_OX_9606_GN_HTR3C_PE_1_SV_
MetazoaVertebrateEP00066Takifugurubripes_5691924_5HT3C_HUMAN_5_hydroxytryptamine_receptor_3C_OS_Homo_sapiens_OX_9606_GN_HTR3C_PE_1_SV_
MetazoaVertebrateEP00066Takifugurubripes_5707647_5HT3C_HUMAN_5_hydroxytryptamine_receptor_3C_OS_Homo_sapiens_OX_9606_GN_HTR3C_PE_1_SV_
MetazoaVertebrateEP00066Takifugurubripes_5707669_5HT3A_RAT_5_hydroxytryptamine_receptor_3A_OS_Rattus_norvegicus_OX_10116_GN_Htr3a_PE_1_SV_
MetazoaVertebrateEP00066Takifugurubripes_5713526_5HT3A_RAT_5_hydroxytryptamine_receptor_3A_OS_Rattus_norvegicus_OX_10116_GN_Htr3a_PE_1_SV_
MetazoaVertebrateEP00066Takifugurubripes_5713936_5HT3A_RAT_5_hydroxytryptamine_receptor_3A_OS_Rattus_norvegicus_OX_10116_GN_Htr3a_PE_1_SV_
MetazoaVertebrateEP00066Takifugurubripes_5707670_5HT3C_HUMAN_5_hydroxytryptamine_receptor_3C_OS_Homo_sapiens_OX_9606_GN_HTR3C_PE_1_SV_
MetazoaVertebrateEP00066Takifugurubripes_5691925_5HT3A_HUMAN_5_hydroxytryptamine_receptor_3A_OS_Homo_sapiens_OX_9606_GN_HTR3A_PE_1_SV_
MetazoaVertebrateEP00066Takifugurubripes_5691926_5HT3A_RAT_5_hydroxytryptamine_receptor_3A_OS_Rattus_norvegicus_OX_10116_GN_Htr3a_PE_1_SV_
MetazoaVertebrateEP00067Daniorerio_5753965_
MetazoaVertebrateEP00067Daniorerio_5753971_5HT3E_HUMAN_5_hydroxytryptamine_receptor_3E_OS_Homo_sapiens_OX_9606_GN_HTR3E_PE_1_SV_
MetazoaVertebrateMonodelphisdomestica_6509931_5HT3C_HUMAN_5_hydroxytryptamine_receptor_3C_OS_Homo_sapiens_OX_9606_GN_HTR3C_PE_1_SV_
MetazoaVertebrateMonodelphisdomestica_6509932_5HT3C_HUMAN_5_hydroxytryptamine_receptor_3C_OS_Homo_sapiens_OX_9606_GN_HTR3C_PE_1_SV_
MetazoaVertebrateMonodelphisdomestica_6509930_5HT3E_HUMAN_5_hydroxytryptamine_receptor_3E_OS_Homo_sapiens_OX_9606_GN_HTR3E_PE_1_SV_
MetazoaVertebrateMonodelphisdomestica_6510033_5HT3C_HUMAN_5_hydroxytryptamine_receptor_3C_OS_Homo_sapiens_OX_9606_GN_HTR3C_PE_1_SV_
MetazoaVertebrateMonodelphisdomestica_6510034_5HT3C_HUMAN_5_hydroxytryptamine_receptor_3C_OS_Homo_sapiens_OX_9606_GN_HTR3C_PE_1_SV_
MetazoaVertebrateMonodelphisdomestica_6510124_5HT3C_HUMAN_5_hydroxytryptamine_receptor_3C_OS_Homo_sapiens_OX_9606_GN_HTR3C_PE_1_SV_
MetazoaVertebrateMonodelphisdomestica_6510125_5HT3C_HUMAN_5_hydroxytryptamine_receptor_3C_OS_Homo_sapiens_OX_9606_GN_HTR3C_PE_1_SV_
MetazoaVertebrateHomosapiens_6451118_5HT3E_HUMAN_5_hydroxytryptamine_receptor_3E_OS_Homo_sapiens_OX_9606_GN_HTR3E_PE_1_SV_
MetazoaVertebrateHomosapiens_6461700_5HT3D_HUMAN_5_hydroxytryptamine_receptor_3D_OS_Homo_sapiens_OX_9606_GN_HTR3D_PE_1_SV_
MetazoaVertebrateHomosapiens_6464825_5HT3C_HUMAN_5_hydroxytryptamine_receptor_3C_OS_Homo_sapiens_OX_9606_GN_HTR3C_PE_1_SV_
MetazoaVertebrateEP00067Daniorerio_5742449_5HT3A_RAT_5_hydroxytryptamine_receptor_3A_OS_Rattus_norvegicus_OX_10116_GN_Htr3a_PE_1_SV_
MetazoaVertebrateEP00067Daniorerio_5754141_5HT3A_RAT_5_hydroxytryptamine_receptor_3A_OS_Rattus_norvegicus_OX_10116_GN_Htr3a_PE_1_SV_
MetazoaVertebrateEP00066Takifugurubripes_5707671_5HT3C_HUMAN_5_hydroxytryptamine_receptor_3C_OS_Homo_sapiens_OX_9606_GN_HTR3C_PE_1_SV_
MetazoaVertebrateLatimeriachalumnae_5794966_5HT3E_HUMAN_5_hydroxytryptamine_receptor_3E_OS_Homo_sapiens_OX_9606_GN_HTR3E_PE_1_SV_
MetazoaVertebrateEP00079Xenopustropicalis_6378554_5HT3A_HUMAN_5_hydroxytryptamine_receptor_3A_OS_Homo_sapiens_OX_9606_GN_HTR3A_PE_1_SV_
MetazoaVertebrateLatimeriachalumnae_5772071_5HT3A_RAT_5_hydroxytryptamine_receptor_3A_OS_Rattus_norvegicus_OX_10116_GN_Htr3a_PE_1_SV_
MetazoaVertebrateLatimeriachalumnae_5789101_5HT3A_RAT_5_hydroxytryptamine_receptor_3A_OS_Rattus_norvegicus_OX_10116_GN_Htr3a_PE_1_SV_
MetazoaVertebrateLatimeriachalumnae_5789091_5HT3A_RAT_5_hydroxytryptamine_receptor_3A_OS_Rattus_norvegicus_OX_10116_GN_Htr3a_PE_1_SV_
MetazoaVertebrateLatimeriachalumnae_5789092_5HT3A_HUMAN_5_hydroxytryptamine_receptor_3A_OS_Homo_sapiens_OX_9606_GN_HTR3A_PE_1_SV_
MetazoaVertebrateCallorhinchusmilii_5524047_5HT3A_RAT_5_hydroxytryptamine_receptor_3A_OS_Rattus_norvegicus_OX_10116_GN_Htr3a_PE_1_SV_
MetazoaVertebrateEP00067Daniorerio_5748744_5HT3A_HUMAN_5_hydroxytryptamine_receptor_3A_OS_Homo_sapiens_OX_9606_GN_HTR3A_PE_1_SV_
MetazoaVertebrateEP00067Daniorerio_5756409_5HT3A_HUMAN_5_hydroxytryptamine_receptor_3A_OS_Homo_sapiens_OX_9606_GN_HTR3A_PE_1_SV_
MetazoaVertebrateEP00067Daniorerio_5739171_5HT3C_HUMAN_5_hydroxytryptamine_receptor_3C_OS_Homo_sapiens_OX_9606_GN_HTR3C_PE_1_SV_
MetazoaVertebrateEP00067Daniorerio_5743787_5HT3C_HUMAN_5_hydroxytryptamine_receptor_3C_OS_Homo_sapiens_OX_9606_GN_HTR3C_PE_1_SV_
MetazoaVertebrateEP00067Daniorerio_5759262_5HT3A_RAT_5_hydroxytryptamine_receptor_3A_OS_Rattus_norvegicus_OX_10116_GN_Htr3a_PE_1_SV_
MetazoaVertebrateEP00066Takifugurubripes_5712177_5HT3A_HUMAN_5_hydroxytryptamine_receptor_3A_OS_Homo_sapiens_OX_9606_GN_HTR3A_PE_1_SV_
MetazoaVertebrateEP00066Takifugurubripes_5712184_5HT3A_RAT_5_hydroxytryptamine_receptor_3A_OS_Rattus_norvegicus_OX_10116_GN_Htr3a_PE_1_SV_
MetazoaVertebrateEP00066Takifugurubripes_5694087_5HT3A_HUMAN_5_hydroxytryptamine_receptor_3A_OS_Homo_sapiens_OX_9606_GN_HTR3A_PE_1_SV_
MetazoaVertebrateAnoliscarolinensis_6334699_5HT3C_HUMAN_5_hydroxytryptamine_receptor_3C_OS_Homo_sapiens_OX_9606_GN_HTR3C_PE_1_SV_
MetazoaVertebrateGallusgallus_6414854_5HT3A_CAVPO_5_hydroxytryptamine_receptor_3A_OS_Cavia_porcellus_OX_10141_GN_HTR3A_PE_1_SV_
MetazoaVertebrateLatimeriachalumnae_5792532_5HT3A_RAT_5_hydroxytryptamine_receptor_3A_OS_Rattus_norvegicus_OX_10116_GN_Htr3a_PE_1_SV_
MetazoaVertebrateEP00079Xenopustropicalis_6351730_5HT3A_HUMAN_5_hydroxytryptamine_receptor_3A_OS_Homo_sapiens_OX_9606_GN_HTR3A_PE_1_SV_
MetazoaVertebrateEP00079Xenopustropicalis_6367534_5HT3A_HUMAN_5_hydroxytryptamine_receptor_3A_OS_Homo_sapiens_OX_9606_GN_HTR3A_PE_1_SV_
MetazoaVertebrateEP00079Xenopustropicalis_6378003_5HT3A_RAT_5_hydroxytryptamine_receptor_3A_OS_Rattus_norvegicus_OX_10116_GN_Htr3a_PE_1_SV_
MetazoaVertebrateEP00079Xenopustropicalis_6378004_5HT3A_RAT_5_hydroxytryptamine_receptor_3A_OS_Rattus_norvegicus_OX_10116_GN_Htr3a_PE_1_SV_
MetazoaUrochordataCionaintestinalis_5346630_5HT3A_MOUSE_5_hydroxytryptamine_receptor_3A_OS_Mus_musculus_OX_10090_GN_Htr3a_PE_1_SV_
MetazoaUrochordataCionaintestinalis_5347767_5HT3A_MOUSE_5_hydroxytryptamine_receptor_3A_OS_Mus_musculus_OX_10090_GN_Htr3a_PE_1_SV_
MetazoaUrochordataCionaintestinalis_5341728_5HT3A_MOUSE_5_hydroxytryptamine_receptor_3A_OS_Mus_musculus_OX_10090_GN_Htr3a_PE_1_SV_
MetazoaVertebrateEP00067Daniorerio_5743784_
MetazoaVertebrateEP00067Daniorerio_5755499_5HT3A_CAVPO_5_hydroxytryptamine_receptor_3A_OS_Cavia_porcellus_OX_10141_GN_HTR3A_PE_1_SV_
MetazoaVertebrateEP00067Daniorerio_5759257_5HT3A_CAVPO_5_hydroxytryptamine_receptor_3A_OS_Cavia_porcellus_OX_10141_GN_HTR3A_PE_1_SV_
MetazoaVertebrateEP00067Daniorerio_5755400_5HT3A_CAVPO_5_hydroxytryptamine_receptor_3A_OS_Cavia_porcellus_OX_10141_GN_HTR3A_PE_1_SV_
MetazoaVertebrateEP00067Daniorerio_5755401_5HT3A_CAVPO_5_hydroxytryptamine_receptor_3A_OS_Cavia_porcellus_OX_10141_GN_HTR3A_PE_1_SV_
MetazoaVertebrateEP00067Daniorerio_5743783_5HT3A_HUMAN_5_hydroxytryptamine_receptor_3A_OS_Homo_sapiens_OX_9606_GN_HTR3A_PE_1_SV_
MetazoaVertebrateEP00066Takifugurubripes_5696740_
MetazoaVertebrateHomosapiens_6459549_ZACN_HUMAN_Zinc_activated_ligand_gated_ion_channel_OS_Homo_sapiens_OX_9606_GN_ZACN_PE_1_SV_
MetazoaVertebrateMonodelphisdomestica_6497698_ZACN_CANLF_Zinc_activated_ligand_gated_ion_channel_OS_Canis_lupus_familiaris_OX_9615_GN_ZACN_PE_3_SV_
MetazoaAcoelaHofsteniamiamia_14172_ACH10_HUMAN_Neuronal_acetylcholine_receptor_subunit_alpha_10_OS_Homo_sapiens_OX_9606_GN_CHRNA10_PE_1_SV_
MetazoaAcoelaHofsteniamiamia_18060_ACHA5_CHICK_Neuronal_acetylcholine_receptor_subunit_alpha_5_OS_Gallus_gallus_OX_9031_GN_CHRNA5_PE_1_SV_
0.43

16B.

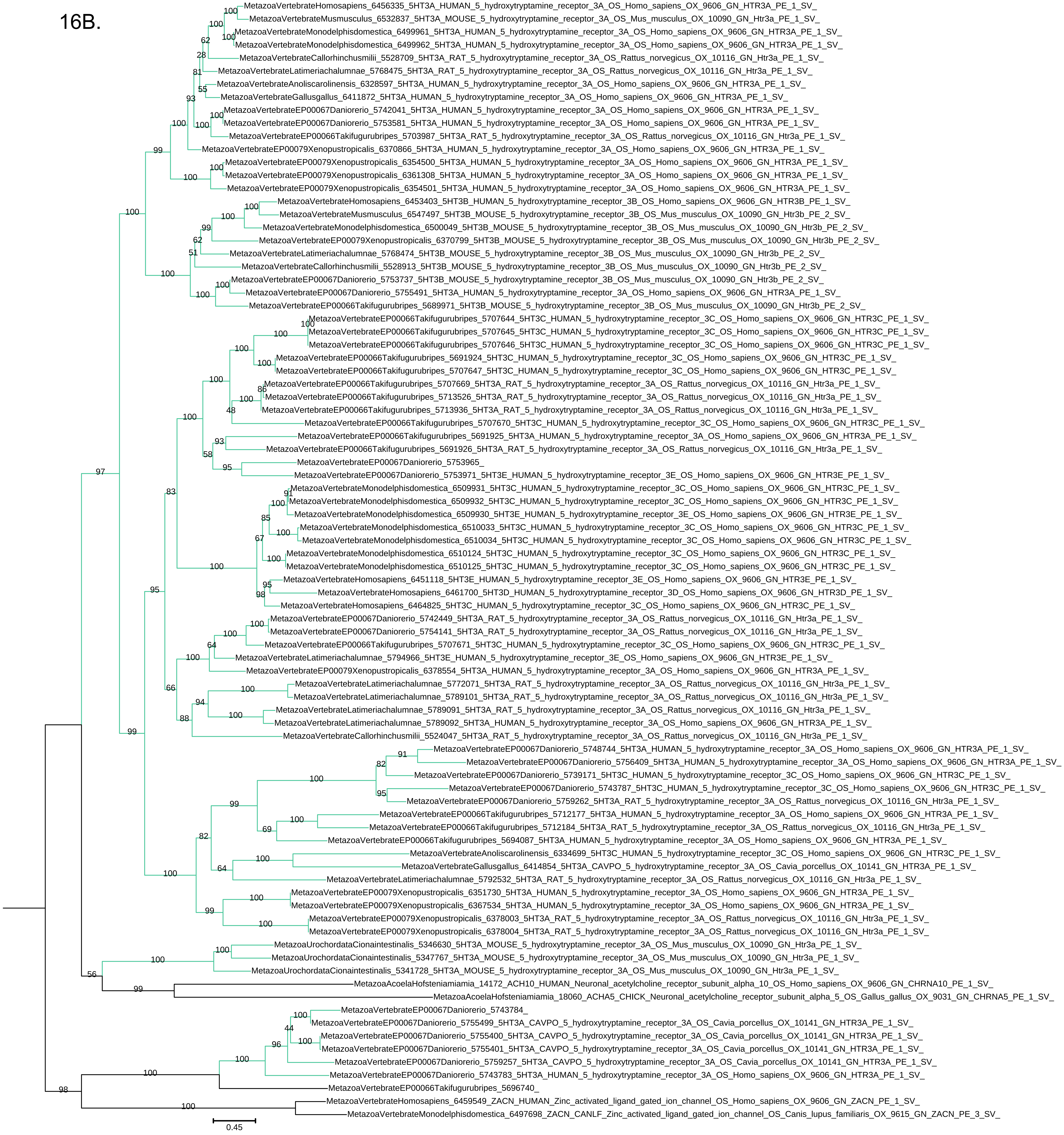
MetazoaVertebrateHomosapiens_6456335_5HT3A_HUMAN_5_hydroxytryptamine_receptor_3A_OS_Homo_sapiens_OX_9606_GN_HTR3A_PE_1_SV_
MetazoaVertebrateMusmusculus_6532837_5HT3A_MOUSE_5_hydroxytryptamine_receptor_3A_OS_Mus_musculus_OX_10090_GN_Htr3a_PE_1_SV_
MetazoaVertebrateMonodelphisdomestica_6499961_5HT3A_HUMAN_5_hydroxytryptamine_receptor_3A_OS_Homo_sapiens_OX_9606_GN_HTR3A_PE_1_SV_
MetazoaVertebrateMonodelphisdomestica_6499962_5HT3A_HUMAN_5_hydroxytryptamine_receptor_3A_OS_Homo_sapiens_OX_9606_GN_HTR3A_PE_1_SV_
MetazoaVertebrateCallorhinchusmilii_5528709_5HT3A_RAT_5_hydroxytryptamine_receptor_3A_OS_Rattus_norvegicus_OX_10116_GN_Htr3a_PE_1_SV_
MetazoaVertebrateLatimeriachalumnae_5768475_5HT3A_RAT_5_hydroxytryptamine_receptor_3A_OS_Rattus_norvegicus_OX_10116_GN_Htr3a_PE_1_SV_
MetazoaVertebrateAnoliscarolinensis_6328597_5HT3A_HUMAN_5_hydroxytryptamine_receptor_3A_OS_Homo_sapiens_OX_9606_GN_HTR3A_PE_1_SV_
MetazoaVertebrateGallusgallus_6411872_5HT3A_HUMAN_5_hydroxytryptamine_receptor_3A_OS_Homo_sapiens_OX_9606_GN_HTR3A_PE_1_SV_
MetazoaVertebrateEP00067Daniorerio_5742041_5HT3A_HUMAN_5_hydroxytryptamine_receptor_3A_OS_Homo_sapiens_OX_9606_GN_HTR3A_PE_1_SV_
MetazoaVertebrateEP00067Daniorerio_5753581_5HT3A_HUMAN_5_hydroxytryptamine_receptor_3A_OS_Homo_sapiens_OX_9606_GN_HTR3A_PE_1_SV_
MetazoaVertebrateEP00066Takifugurubripes_5703987_5HT3A_RAT_5_hydroxytryptamine_receptor_3A_OS_Rattus_norvegicus_OX_10116_GN_Htr3a_PE_1_SV_
MetazoaVertebrateEP00079Xenopustropicalis_6370866_5HT3A_HUMAN_5_hydroxytryptamine_receptor_3A_OS_Homo_sapiens_OX_9606_GN_HTR3A_PE_1_SV_
MetazoaVertebrateEP00079Xenopustropicalis_6354500_5HT3A_HUMAN_5_hydroxytryptamine_receptor_3A_OS_Homo_sapiens_OX_9606_GN_HTR3A_PE_1_SV_
MetazoaVertebrateEP00079Xenopustropicalis_6361308_5HT3A_HUMAN_5_hydroxytryptamine_receptor_3A_OS_Homo_sapiens_OX_9606_GN_HTR3A_PE_1_SV_
MetazoaVertebrateEP00079Xenopustropicalis_6354501_5HT3A_HUMAN_5_hydroxytryptamine_receptor_3A_OS_Homo_sapiens_OX_9606_GN_HTR3A_PE_1_SV_
MetazoaVertebrateHomosapiens_6453403_5HT3B_HUMAN_5_hydroxytryptamine_receptor_3B_OS_Homo_sapiens_OX_9606_GN_HTR3B_PE_1_SV_
MetazoaVertebrateMusmusculus_6547497_5HT3B_MOUSE_5_hydroxytryptamine_receptor_3B_OS_Mus_musculus_OX_10090_GN_Htr3b_PE_2_SV_
MetazoaVertebrateMonodelphisdomestica_6500049_5HT3B_MOUSE_5_hydroxytryptamine_receptor_3B_OS_Mus_musculus_OX_10090_GN_Htr3b_PE_2_SV_
MetazoaVertebrateEP00079Xenopustropicalis_6370799_5HT3B_MOUSE_5_hydroxytryptamine_receptor_3B_OS_Mus_musculus_OX_10090_GN_Htr3b_PE_2_SV_
MetazoaVertebrateLatimeriachalumnae_5768474_5HT3B_MOUSE_5_hydroxytryptamine_receptor_3B_OS_Mus_musculus_OX_10090_GN_Htr3b_PE_2_SV_
MetazoaVertebrateCallorhinchusmilii_5528913_5HT3B_MOUSE_5_hydroxytryptamine_receptor_3B_OS_Mus_musculus_OX_10090_GN_Htr3b_PE_2_SV_
MetazoaVertebrateEP00067Daniorerio_5753737_5HT3B_MOUSE_5_hydroxytryptamine_receptor_3B_OS_Mus_musculus_OX_10090_GN_Htr3b_PE_2_SV_
MetazoaVertebrateEP00067Daniorerio_5755491_5HT3A_HUMAN_5_hydroxytryptamine_receptor_3A_OS_Homo_sapiens_OX_9606_GN_HTR3A_PE_1_SV_
MetazoaVertebrateEP00066Takifugurubripes_5689971_5HT3B_MOUSE_5_hydroxytryptamine_receptor_3B_OS_Mus_musculus_OX_10090_GN_Htr3b_PE_2_SV_
MetazoaVertebrateEP00066Takifugurubripes_5707644_5HT3C_HUMAN_5_hydroxytryptamine_receptor_3C_OS_Homo_sapiens_OX_9606_GN_HTR3C_PE_1_SV_
MetazoaVertebrateEP00066Takifugurubripes_5707645_5HT3C_HUMAN_5_hydroxytryptamine_receptor_3C_OS_Homo_sapiens_OX_9606_GN_HTR3C_PE_1_SV_
MetazoaVertebrateEP00066Takifugurubripes_5707646_5HT3C_HUMAN_5_hydroxytryptamine_receptor_3C_OS_Homo_sapiens_OX_9606_GN_HTR3C_PE_1_SV_
MetazoaVertebrateEP00066Takifugurubripes_5691924_5HT3C_HUMAN_5_hydroxytryptamine_receptor_3C_OS_Homo_sapiens_OX_9606_GN_HTR3C_PE_1_SV_
MetazoaVertebrateEP00066Takifugurubripes_5707647_5HT3C_HUMAN_5_hydroxytryptamine_receptor_3C_OS_Homo_sapiens_OX_9606_GN_HTR3C_PE_1_SV_
MetazoaVertebrateEP00066Takifugurubripes_5707669_5HT3A_RAT_5_hydroxytryptamine_receptor_3A_OS_Rattus_norvegicus_OX_10116_GN_Htr3a_PE_1_SV_
MetazoaVertebrateEP00066Takifugurubripes_5713526_5HT3A_RAT_5_hydroxytryptamine_receptor_3A_OS_Rattus_norvegicus_OX_10116_GN_Htr3a_PE_1_SV_
MetazoaVertebrateEP00066Takifugurubripes_5713936_5HT3A_RAT_5_hydroxytryptamine_receptor_3A_OS_Rattus_norvegicus_OX_10116_GN_Htr3a_PE_1_SV_
MetazoaVertebrateEP00066Takifugurubripes_5707670_5HT3C_HUMAN_5_hydroxytryptamine_receptor_3C_OS_Homo_sapiens_OX_9606_GN_HTR3C_PE_1_SV_
MetazoaVertebrateEP00066Takifugurubripes_5691925_5HT3A_HUMAN_5_hydroxytryptamine_receptor_3A_OS_Homo_sapiens_OX_9606_GN_HTR3A_PE_1_SV_
MetazoaVertebrateEP00066Takifugurubripes_5691926_5HT3A_RAT_5_hydroxytryptamine_receptor_3A_OS_Rattus_norvegicus_OX_10116_GN_Htr3a_PE_1_SV_
MetazoaVertebrateEP00067Daniorerio_5753965_
MetazoaVertebrateEP00067Daniorerio_5753971_5HT3E_HUMAN_5_hydroxytryptamine_receptor_3E_OS_Homo_sapiens_OX_9606_GN_HTR3E_PE_1_SV_
MetazoaVertebrateMonodelphisdomestica_6509931_5HT3C_HUMAN_5_hydroxytryptamine_receptor_3C_OS_Homo_sapiens_OX_9606_GN_HTR3C_PE_1_SV_
MetazoaVertebrateMonodelphisdomestica_6509932_5HT3C_HUMAN_5_hydroxytryptamine_receptor_3C_OS_Homo_sapiens_OX_9606_GN_HTR3C_PE_1_SV_
MetazoaVertebrateMonodelphisdomestica_6509930_5HT3E_HUMAN_5_hydroxytryptamine_receptor_3E_OS_Homo_sapiens_OX_9606_GN_HTR3E_PE_1_SV_
MetazoaVertebrateMonodelphisdomestica_6510033_5HT3C_HUMAN_5_hydroxytryptamine_receptor_3C_OS_Homo_sapiens_OX_9606_GN_HTR3C_PE_1_SV_
MetazoaVertebrateMonodelphisdomestica_6510034_5HT3C_HUMAN_5_hydroxytryptamine_receptor_3C_OS_Homo_sapiens_OX_9606_GN_HTR3C_PE_1_SV_
MetazoaVertebrateMonodelphisdomestica_6510124_5HT3C_HUMAN_5_hydroxytryptamine_receptor_3C_OS_Homo_sapiens_OX_9606_GN_HTR3C_PE_1_SV_
MetazoaVertebrateMonodelphisdomestica_6510125_5HT3C_HUMAN_5_hydroxytryptamine_receptor_3C_OS_Homo_sapiens_OX_9606_GN_HTR3C_PE_1_SV_
MetazoaVertebrateHomosapiens_6451118_5HT3E_HUMAN_5_hydroxytryptamine_receptor_3E_OS_Homo_sapiens_OX_9606_GN_HTR3E_PE_1_SV_
MetazoaVertebrateHomosapiens_6461700_5HT3D_HUMAN_5_hydroxytryptamine_receptor_3D_OS_Homo_sapiens_OX_9606_GN_HTR3D_PE_1_SV_
MetazoaVertebrateHomosapiens_6464825_5HT3C_HUMAN_5_hydroxytryptamine_receptor_3C_OS_Homo_sapiens_OX_9606_GN_HTR3C_PE_1_SV_
MetazoaVertebrateEP00067Daniorerio_5742449_5HT3A_RAT_5_hydroxytryptamine_receptor_3A_OS_Rattus_norvegicus_OX_10116_GN_Htr3a_PE_1_SV_
MetazoaVertebrateEP00067Daniorerio_5754141_5HT3A_RAT_5_hydroxytryptamine_receptor_3A_OS_Rattus_norvegicus_OX_10116_GN_Htr3a_PE_1_SV_
MetazoaVertebrateEP00066Takifugurubripes_5707671_5HT3C_HUMAN_5_hydroxytryptamine_receptor_3C_OS_Homo_sapiens_OX_9606_GN_HTR3C_PE_1_SV_
MetazoaVertebrateLatimeriachalumnae_5794966_5HT3E_HUMAN_5_hydroxytryptamine_receptor_3E_OS_Homo_sapiens_OX_9606_GN_HTR3E_PE_1_SV_
MetazoaVertebrateEP00079Xenopustropicalis_6378554_5HT3A_HUMAN_5_hydroxytryptamine_receptor_3A_OS_Homo_sapiens_OX_9606_GN_HTR3A_PE_1_SV_
MetazoaVertebrateLatimeriachalumnae_5772071_5HT3A_RAT_5_hydroxytryptamine_receptor_3A_OS_Rattus_norvegicus_OX_10116_GN_Htr3a_PE_1_SV_
MetazoaVertebrateLatimeriachalumnae_5789101_5HT3A_RAT_5_hydroxytryptamine_receptor_3A_OS_Rattus_norvegicus_OX_10116_GN_Htr3a_PE_1_SV_
MetazoaVertebrateLatimeriachalumnae_5789091_5HT3A_RAT_5_hydroxytryptamine_receptor_3A_OS_Rattus_norvegicus_OX_10116_GN_Htr3a_PE_1_SV_
MetazoaVertebrateLatimeriachalumnae_5789092_5HT3A_HUMAN_5_hydroxytryptamine_receptor_3A_OS_Homo_sapiens_OX_9606_GN_HTR3A_PE_1_SV_
MetazoaVertebrateCallorhinchusmilii_5524047_5HT3A_RAT_5_hydroxytryptamine_receptor_3A_OS_Rattus_norvegicus_OX_10116_GN_Htr3a_PE_1_SV_
MetazoaVertebrateEP00067Daniorerio_5748744_5HT3A_HUMAN_5_hydroxytryptamine_receptor_3A_OS_Homo_sapiens_OX_9606_GN_HTR3A_PE_1_SV_
MetazoaVertebrateEP00067Daniorerio_5756409_5HT3A_HUMAN_5_hydroxytryptamine_receptor_3A_OS_Homo_sapiens_OX_9606_GN_HTR3A_PE_1_SV_
MetazoaVertebrateEP00067Daniorerio_5739171_5HT3C_HUMAN_5_hydroxytryptamine_receptor_3C_OS_Homo_sapiens_OX_9606_GN_HTR3C_PE_1_SV_
MetazoaVertebrateEP00067Daniorerio_5743787_5HT3C_HUMAN_5_hydroxytryptamine_receptor_3C_OS_Homo_sapiens_OX_9606_GN_HTR3C_PE_1_SV_
MetazoaVertebrateEP00067Daniorerio_5759262_5HT3A_RAT_5_hydroxytryptamine_receptor_3A_OS_Rattus_norvegicus_OX_10116_GN_Htr3a_PE_1_SV_
MetazoaVertebrateEP00066Takifugurubripes_5712177_5HT3A_HUMAN_5_hydroxytryptamine_receptor_3A_OS_Homo_sapiens_OX_9606_GN_HTR3A_PE_1_SV_
MetazoaVertebrateEP00066Takifugurubripes_5712184_5HT3A_RAT_5_hydroxytryptamine_receptor_3A_OS_Rattus_norvegicus_OX_10116_GN_Htr3a_PE_1_SV_
MetazoaVertebrateEP00066Takifugurubripes_5694087_5HT3A_HUMAN_5_hydroxytryptamine_receptor_3A_OS_Homo_sapiens_OX_9606_GN_HTR3A_PE_1_SV_
MetazoaVertebrateAnoliscarolinensis_6334699_5HT3C_HUMAN_5_hydroxytryptamine_receptor_3C_OS_Homo_sapiens_OX_9606_GN_HTR3C_PE_1_SV_
MetazoaVertebrateGallusgallus_6414854_5HT3A_CAVPO_5_hydroxytryptamine_receptor_3A_OS_Cavia_porcellus_OX_10141_GN_HTR3A_PE_1_SV_
MetazoaVertebrateLatimeriachalumnae_5792532_5HT3A_RAT_5_hydroxytryptamine_receptor_3A_OS_Rattus_norvegicus_OX_10116_GN_Htr3a_PE_1_SV_
MetazoaVertebrateEP00079Xenopustropicalis_6351730_5HT3A_HUMAN_5_hydroxytryptamine_receptor_3A_OS_Homo_sapiens_OX_9606_GN_HTR3A_PE_1_SV_
MetazoaVertebrateEP00079Xenopustropicalis_6367534_5HT3A_HUMAN_5_hydroxytryptamine_receptor_3A_OS_Homo_sapiens_OX_9606_GN_HTR3A_PE_1_SV_
MetazoaVertebrateEP00079Xenopustropicalis_6378003_5HT3A_RAT_5_hydroxytryptamine_receptor_3A_OS_Rattus_norvegicus_OX_10116_GN_Htr3a_PE_1_SV_
MetazoaVertebrateEP00079Xenopustropicalis_6378004_5HT3A_RAT_5_hydroxytryptamine_receptor_3A_OS_Rattus_norvegicus_OX_10116_GN_Htr3a_PE_1_SV_
MetazoaUrochordataCionaintestinalis_5346630_5HT3A_MOUSE_5_hydroxytryptamine_receptor_3A_OS_Mus_musculus_OX_10090_GN_Htr3a_PE_1_SV_
MetazoaUrochordataCionaintestinalis_5347767_5HT3A_MOUSE_5_hydroxytryptamine_receptor_3A_OS_Mus_musculus_OX_10090_GN_Htr3a_PE_1_SV_
MetazoaUrochordataCionaintestinalis_5341728_5HT3A_MOUSE_5_hydroxytryptamine_receptor_3A_OS_Mus_musculus_OX_10090_GN_Htr3a_PE_1_SV_
MetazoaAcoelaHofsteniamiamia_14172_ACH10_HUMAN_Neuronal_acetylcholine_receptor_subunit_alpha_10_OS_Homo_sapiens_OX_9606_GN_CHRNA10_PE_1_SV_
MetazoaAcoelaHofsteniamiamia_18060_ACHA5_CHICK_Neuronal_acetylcholine_receptor_subunit_alpha_5_OS_Gallus_gallus_OX_9031_GN_CHRNA5_PE_1_SV_
MetazoaVertebrateEP00067Daniorerio_5743784_
MetazoaVertebrateEP00067Daniorerio_5755499_5HT3A_CAVPO_5_hydroxytryptamine_receptor_3A_OS_Cavia_porcellus_OX_10141_GN_HTR3A_PE_1_SV_
MetazoaVertebrateEP00067Daniorerio_5755400_5HT3A_CAVPO_5_hydroxytryptamine_receptor_3A_OS_Cavia_porcellus_OX_10141_GN_HTR3A_PE_1_SV_
MetazoaVertebrateEP00067Daniorerio_5755401_5HT3A_CAVPO_5_hydroxytryptamine_receptor_3A_OS_Cavia_porcellus_OX_10141_GN_HTR3A_PE_1_SV_
MetazoaVertebrateEP00067Daniorerio_5759257_5HT3A_CAVPO_5_hydroxytryptamine_receptor_3A_OS_Cavia_porcellus_OX_10141_GN_HTR3A_PE_1_SV_
MetazoaVertebrateEP00067Daniorerio_5743783_5HT3A_HUMAN_5_hydroxytryptamine_receptor_3A_OS_Homo_sapiens_OX_9606_GN_HTR3A_PE_1_SV_
MetazoaVertebrateEP00066Takifugurubripes_5696740_
MetazoaVertebrateHomosapiens_6459549_ZACN_HUMAN_Zinc_activated_ligand_gated_ion_channel_OS_Homo_sapiens_OX_9606_GN_ZACN_PE_1_SV_
MetazoaVertebrateMonodelphisdomestica_6497698_ZACN_CANLF_Zinc_activated_ligand_gated_ion_channel_OS_Canis_lupus_familiaris_OX_9615_GN_ZACN_PE_3_SV_
0.45

16C.

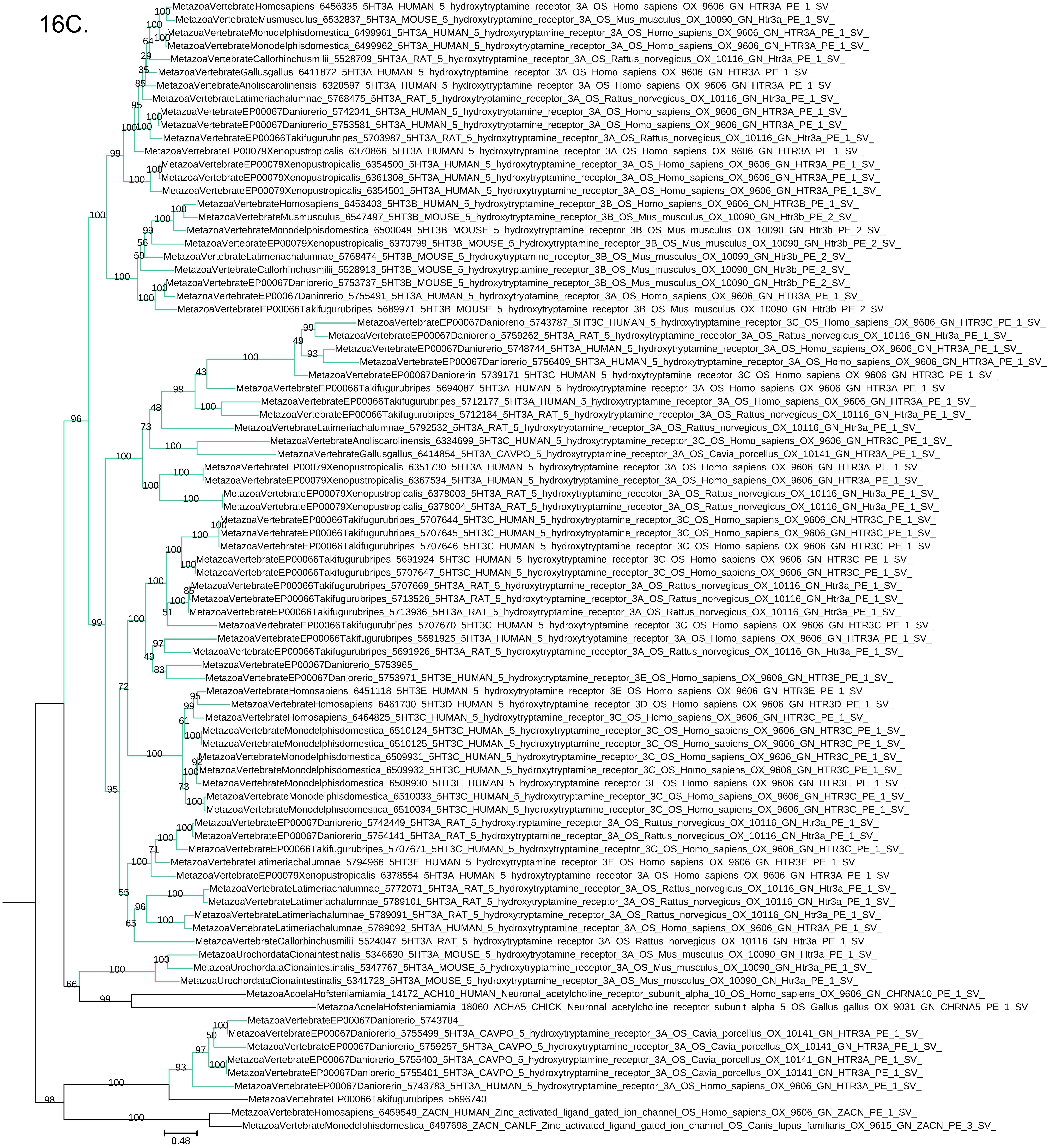
MetazoaVertebrateHomosapiens_6456335_5HT3A_HUMAN_5_hydroxytryptamine_receptor_3A_OS_Homo_sapiens_OX_9606_GN_HTR3A_PE_1_SV_
MetazoaVertebrateMusmusculus_6532837_5HT3A_MOUSE_5_hydroxytryptamine_receptor_3A_OS_Mus_musculus_OX_10090_GN_Htr3a_PE_1_SV_
MetazoaVertebrateMonodelphisdomestica_6499961_5HT3A_HUMAN_5_hydroxytryptamine_receptor_3A_OS_Homo_sapiens_OX_9606_GN_HTR3A_PE_1_SV_
MetazoaVertebrateMonodelphisdomestica_6499962_5HT3A_HUMAN_5_hydroxytryptamine_receptor_3A_OS_Homo_sapiens_OX_9606_GN_HTR3A_PE_1_SV_
MetazoaVertebrateCallorhinchusmilii_5528709_5HT3A_RAT_5_hydroxytryptamine_receptor_3A_OS_Rattus_norvegicus_OX_10116_GN_Htr3a_PE_1_SV_
MetazoaVertebrateGallusgallus_6411872_5HT3A_HUMAN_5_hydroxytryptamine_receptor_3A_OS_Homo_sapiens_OX_9606_GN_HTR3A_PE_1_SV_
MetazoaVertebrateAnoliscarolinensis_6328597_5HT3A_HUMAN_5_hydroxytryptamine_receptor_3A_OS_Homo_sapiens_OX_9606_GN_HTR3A_PE_1_SV_
MetazoaVertebrateLatimeriachalumnae_5768475_5HT3A_RAT_5_hydroxytryptamine_receptor_3A_OS_Rattus_norvegicus_OX_10116_GN_Htr3a_PE_1_SV_
MetazoaVertebrateEP00067Daniorerio_5742041_5HT3A_HUMAN_5_hydroxytryptamine_receptor_3A_OS_Homo_sapiens_OX_9606_GN_HTR3A_PE_1_SV_
MetazoaVertebrateEP00067Daniorerio_5753581_5HT3A_HUMAN_5_hydroxytryptamine_receptor_3A_OS_Homo_sapiens_OX_9606_GN_HTR3A_PE_1_SV_
MetazoaVertebrateEP00066Takifugurubripes_5703987_5HT3A_RAT_5_hydroxytryptamine_receptor_3A_OS_Rattus_norvegicus_OX_10116_GN_Htr3a_PE_1_SV_
MetazoaVertebrateEP00079Xenopustropicalis_6370866_5HT3A_HUMAN_5_hydroxytryptamine_receptor_3A_OS_Homo_sapiens_OX_9606_GN_HTR3A_PE_1_SV_
MetazoaVertebrateEP00079Xenopustropicalis_6354500_5HT3A_HUMAN_5_hydroxytryptamine_receptor_3A_OS_Homo_sapiens_OX_9606_GN_HTR3A_PE_1_SV_
MetazoaVertebrateEP00079Xenopustropicalis_6361308_5HT3A_HUMAN_5_hydroxytryptamine_receptor_3A_OS_Homo_sapiens_OX_9606_GN_HTR3A_PE_1_SV_
MetazoaVertebrateEP00079Xenopustropicalis_6354501_5HT3A_HUMAN_5_hydroxytryptamine_receptor_3A_OS_Homo_sapiens_OX_9606_GN_HTR3A_PE_1_SV_
MetazoaVertebrateHomosapiens_6453403_5HT3B_HUMAN_5_hydroxytryptamine_receptor_3B_OS_Homo_sapiens_OX_9606_GN_HTR3B_PE_1_SV_
MetazoaVertebrateMusmusculus_6547497_5HT3B_MOUSE_5_hydroxytryptamine_receptor_3B_OS_Mus_musculus_OX_10090_GN_Htr3b_PE_2_SV_
MetazoaVertebrateMonodelphisdomestica_6500049_5HT3B_MOUSE_5_hydroxytryptamine_receptor_3B_OS_Mus_musculus_OX_10090_GN_Htr3b_PE_2_SV_
MetazoaVertebrateEP00079Xenopustropicalis_6370799_5HT3B_MOUSE_5_hydroxytryptamine_receptor_3B_OS_Mus_musculus_OX_10090_GN_Htr3b_PE_2_SV_
MetazoaVertebrateLatimeriachalumnae_5768474_5HT3B_MOUSE_5_hydroxytryptamine_receptor_3B_OS_Mus_musculus_OX_10090_GN_Htr3b_PE_2_SV_
MetazoaVertebrateCallorhinchusmilii_5528913_5HT3B_MOUSE_5_hydroxytryptamine_receptor_3B_OS_Mus_musculus_OX_10090_GN_Htr3b_PE_2_SV_
MetazoaVertebrateEP00067Daniorerio_5753737_5HT3B_MOUSE_5_hydroxytryptamine_receptor_3B_OS_Mus_musculus_OX_10090_GN_Htr3b_PE_2_SV_
MetazoaVertebrateEP00067Daniorerio_5755491_5HT3A_HUMAN_5_hydroxytryptamine_receptor_3A_OS_Homo_sapiens_OX_9606_GN_HTR3A_PE_1_SV_
MetazoaVertebrateEP00066Takifugurubripes_5689971_5HT3B_MOUSE_5_hydroxytryptamine_receptor_3B_OS_Mus_musculus_OX_10090_GN_Htr3b_PE_2_SV_
MetazoaVertebrateEP00067Daniorerio_5743787_5HT3C_HUMAN_5_hydroxytryptamine_receptor_3C_OS_Homo_sapiens_OX_9606_GN_HTR3C_PE_1_SV_
MetazoaVertebrateEP00067Daniorerio_5759262_5HT3A_RAT_5_hydroxytryptamine_receptor_3A_OS_Rattus_norvegicus_OX_10116_GN_Htr3a_PE_1_SV_
MetazoaVertebrateEP00067Daniorerio_5748744_5HT3A_HUMAN_5_hydroxytryptamine_receptor_3A_OS_Homo_sapiens_OX_9606_GN_HTR3A_PE_1_SV_
MetazoaVertebrateEP00067Daniorerio_5756409_5HT3A_HUMAN_5_hydroxytryptamine_receptor_3A_OS_Homo_sapiens_OX_9606_GN_HTR3A_PE_1_SV_
MetazoaVertebrateEP00067Daniorerio_5739171_5HT3C_HUMAN_5_hydroxytryptamine_receptor_3C_OS_Homo_sapiens_OX_9606_GN_HTR3C_PE_1_SV_
MetazoaVertebrateEP00066Takifugurubripes_5694087_5HT3A_HUMAN_5_hydroxytryptamine_receptor_3A_OS_Homo_sapiens_OX_9606_GN_HTR3A_PE_1_SV_
MetazoaVertebrateEP00066Takifugurubripes_5712177_5HT3A_HUMAN_5_hydroxytryptamine_receptor_3A_OS_Homo_sapiens_OX_9606_GN_HTR3A_PE_1_SV_
MetazoaVertebrateEP00066Takifugurubripes_5712184_5HT3A_RAT_5_hydroxytryptamine_receptor_3A_OS_Rattus_norvegicus_OX_10116_GN_Htr3a_PE_1_SV_
MetazoaVertebrateLatimeriachalumnae_5792532_5HT3A_RAT_5_hydroxytryptamine_receptor_3A_OS_Rattus_norvegicus_OX_10116_GN_Htr3a_PE_1_SV_
MetazoaVertebrateAnoliscarolinensis_6334699_5HT3C_HUMAN_5_hydroxytryptamine_receptor_3C_OS_Homo_sapiens_OX_9606_GN_HTR3C_PE_1_SV_
MetazoaVertebrateGallusgallus_6414854_5HT3A_CAVPO_5_hydroxytryptamine_receptor_3A_OS_Cavia_porcellus_OX_10141_GN_HTR3A_PE_1_SV_
MetazoaVertebrateEP00079Xenopustropicalis_6351730_5HT3A_HUMAN_5_hydroxytryptamine_receptor_3A_OS_Homo_sapiens_OX_9606_GN_HTR3A_PE_1_SV_
MetazoaVertebrateEP00079Xenopustropicalis_6367534_5HT3A_HUMAN_5_hydroxytryptamine_receptor_3A_OS_Homo_sapiens_OX_9606_GN_HTR3A_PE_1_SV_
MetazoaVertebrateEP00079Xenopustropicalis_6378003_5HT3A_RAT_5_hydroxytryptamine_receptor_3A_OS_Rattus_norvegicus_OX_10116_GN_Htr3a_PE_1_SV_
MetazoaVertebrateEP00079Xenopustropicalis_6378004_5HT3A_RAT_5_hydroxytryptamine_receptor_3A_OS_Rattus_norvegicus_OX_10116_GN_Htr3a_PE_1_SV_
MetazoaVertebrateEP00066Takifugurubripes_5707644_5HT3C_HUMAN_5_hydroxytryptamine_receptor_3C_OS_Homo_sapiens_OX_9606_GN_HTR3C_PE_1_SV_
MetazoaVertebrateEP00066Takifugurubripes_5707645_5HT3C_HUMAN_5_hydroxytryptamine_receptor_3C_OS_Homo_sapiens_OX_9606_GN_HTR3C_PE_1_SV_
MetazoaVertebrateEP00066Takifugurubripes_5707646_5HT3C_HUMAN_5_hydroxytryptamine_receptor_3C_OS_Homo_sapiens_OX_9606_GN_HTR3C_PE_1_SV_
MetazoaVertebrateEP00066Takifugurubripes_5691924_5HT3C_HUMAN_5_hydroxytryptamine_receptor_3C_OS_Homo_sapiens_OX_9606_GN_HTR3C_PE_1_SV_
MetazoaVertebrateEP00066Takifugurubripes_5707647_5HT3C_HUMAN_5_hydroxytryptamine_receptor_3C_OS_Homo_sapiens_OX_9606_GN_HTR3C_PE_1_SV_
MetazoaVertebrateEP00066Takifugurubripes_5707669_5HT3A_RAT_5_hydroxytryptamine_receptor_3A_OS_Rattus_norvegicus_OX_10116_GN_Htr3a_PE_1_SV_
MetazoaVertebrateEP00066Takifugurubripes_5713526_5HT3A_RAT_5_hydroxytryptamine_receptor_3A_OS_Rattus_norvegicus_OX_10116_GN_Htr3a_PE_1_SV_
MetazoaVertebrateEP00066Takifugurubripes_5713936_5HT3A_RAT_5_hydroxytryptamine_receptor_3A_OS_Rattus_norvegicus_OX_10116_GN_Htr3a_PE_1_SV_
MetazoaVertebrateEP00066Takifugurubripes_5707670_5HT3C_HUMAN_5_hydroxytryptamine_receptor_3C_OS_Homo_sapiens_OX_9606_GN_HTR3C_PE_1_SV_
MetazoaVertebrateEP00066Takifugurubripes_5691925_5HT3A_HUMAN_5_hydroxytryptamine_receptor_3A_OS_Homo_sapiens_OX_9606_GN_HTR3A_PE_1_SV_
MetazoaVertebrateEP00066Takifugurubripes_5691926_5HT3A_RAT_5_hydroxytryptamine_receptor_3A_OS_Rattus_norvegicus_OX_10116_GN_Htr3a_PE_1_SV_
MetazoaVertebrateEP00067Daniorerio_5753965_
MetazoaVertebrateEP00067Daniorerio_5753971_5HT3E_HUMAN_5_hydroxytryptamine_receptor_3E_OS_Homo_sapiens_OX_9606_GN_HTR3E_PE_1_SV_
MetazoaVertebrateHomosapiens_6451118_5HT3E_HUMAN_5_hydroxytryptamine_receptor_3E_OS_Homo_sapiens_OX_9606_GN_HTR3E_PE_1_SV_
MetazoaVertebrateHomosapiens_6461700_5HT3D_HUMAN_5_hydroxytryptamine_receptor_3D_OS_Homo_sapiens_OX_9606_GN_HTR3D_PE_1_SV_
MetazoaVertebrateHomosapiens_6464825_5HT3C_HUMAN_5_hydroxytryptamine_receptor_3C_OS_Homo_sapiens_OX_9606_GN_HTR3C_PE_1_SV_
MetazoaVertebrateMonodelphisdomestica_6510124_5HT3C_HUMAN_5_hydroxytryptamine_receptor_3C_OS_Homo_sapiens_OX_9606_GN_HTR3C_PE_1_SV_
MetazoaVertebrateMonodelphisdomestica_6510125_5HT3C_HUMAN_5_hydroxytryptamine_receptor_3C_OS_Homo_sapiens_OX_9606_GN_HTR3C_PE_1_SV_
MetazoaVertebrateMonodelphisdomestica_6509931_5HT3C_HUMAN_5_hydroxytryptamine_receptor_3C_OS_Homo_sapiens_OX_9606_GN_HTR3C_PE_1_SV_
MetazoaVertebrateMonodelphisdomestica_6509932_5HT3C_HUMAN_5_hydroxytryptamine_receptor_3C_OS_Homo_sapiens_OX_9606_GN_HTR3C_PE_1_SV_
MetazoaVertebrateMonodelphisdomestica_6509930_5HT3E_HUMAN_5_hydroxytryptamine_receptor_3E_OS_Homo_sapiens_OX_9606_GN_HTR3E_PE_1_SV_
MetazoaVertebrateMonodelphisdomestica_6510033_5HT3C_HUMAN_5_hydroxytryptamine_receptor_3C_OS_Homo_sapiens_OX_9606_GN_HTR3C_PE_1_SV_
MetazoaVertebrateMonodelphisdomestica_6510034_5HT3C_HUMAN_5_hydroxytryptamine_receptor_3C_OS_Homo_sapiens_OX_9606_GN_HTR3C_PE_1_SV_
MetazoaVertebrateEP00067Daniorerio_5742449_5HT3A_RAT_5_hydroxytryptamine_receptor_3A_OS_Rattus_norvegicus_OX_10116_GN_Htr3a_PE_1_SV_
MetazoaVertebrateEP00067Daniorerio_5754141_5HT3A_RAT_5_hydroxytryptamine_receptor_3A_OS_Rattus_norvegicus_OX_10116_GN_Htr3a_PE_1_SV_
MetazoaVertebrateEP00066Takifugurubripes_5707671_5HT3C_HUMAN_5_hydroxytryptamine_receptor_3C_OS_Homo_sapiens_OX_9606_GN_HTR3C_PE_1_SV_
MetazoaVertebrateLatimeriachalumnae_5794966_5HT3E_HUMAN_5_hydroxytryptamine_receptor_3E_OS_Homo_sapiens_OX_9606_GN_HTR3E_PE_1_SV_
MetazoaVertebrateEP00079Xenopustropicalis_6378554_5HT3A_HUMAN_5_hydroxytryptamine_receptor_3A_OS_Homo_sapiens_OX_9606_GN_HTR3A_PE_1_SV_
MetazoaVertebrateLatimeriachalumnae_5772071_5HT3A_RAT_5_hydroxytryptamine_receptor_3A_OS_Rattus_norvegicus_OX_10116_GN_Htr3a_PE_1_SV_
MetazoaVertebrateLatimeriachalumnae_5789101_5HT3A_RAT_5_hydroxytryptamine_receptor_3A_OS_Rattus_norvegicus_OX_10116_GN_Htr3a_PE_1_SV_
MetazoaVertebrateLatimeriachalumnae_5789091_5HT3A_RAT_5_hydroxytryptamine_receptor_3A_OS_Rattus_norvegicus_OX_10116_GN_Htr3a_PE_1_SV_
MetazoaVertebrateLatimeriachalumnae_5789092_5HT3A_HUMAN_5_hydroxytryptamine_receptor_3A_OS_Homo_sapiens_OX_9606_GN_HTR3A_PE_1_SV_
MetazoaVertebrateCallorhinchusmilii_5524047_5HT3A_RAT_5_hydroxytryptamine_receptor_3A_OS_Rattus_norvegicus_OX_10116_GN_Htr3a_PE_1_SV_
MetazoaUrochordataCionaintestinalis_5346630_5HT3A_MOUSE_5_hydroxytryptamine_receptor_3A_OS_Mus_musculus_OX_10090_GN_Htr3a_PE_1_SV_
MetazoaUrochordataCionaintestinalis_5347767_5HT3A_MOUSE_5_hydroxytryptamine_receptor_3A_OS_Mus_musculus_OX_10090_GN_Htr3a_PE_1_SV_
MetazoaUrochordataCionaintestinalis_5341728_5HT3A_MOUSE_5_hydroxytryptamine_receptor_3A_OS_Mus_musculus_OX_10090_GN_Htr3a_PE_1_SV_
MetazoaAcoelaHofsteniamiamia_14172_ACH10_HUMAN_Neuronal_acetylcholine_receptor_subunit_alpha_10_OS_Homo_sapiens_OX_9606_GN_CHRNA10_PE_1_SV_
MetazoaAcoelaHofsteniamiamia_18060_ACHA5_CHICK_Neuronal_acetylcholine_receptor_subunit_alpha_5_OS_Gallus_gallus_OX_9031_GN_CHRNA5_PE_1_SV_
MetazoaVertebrateEP00067Daniorerio_5743784_
MetazoaVertebrateEP00067Daniorerio_5755499_5HT3A_CAVPO_5_hydroxytryptamine_receptor_3A_OS_Cavia_porcellus_OX_10141_GN_HTR3A_PE_1_SV_
MetazoaVertebrateEP00067Daniorerio_5759257_5HT3A_CAVPO_5_hydroxytryptamine_receptor_3A_OS_Cavia_porcellus_OX_10141_GN_HTR3A_PE_1_SV_
MetazoaVertebrateEP00067Daniorerio_5755400_5HT3A_CAVPO_5_hydroxytryptamine_receptor_3A_OS_Cavia_porcellus_OX_10141_GN_HTR3A_PE_1_SV_
MetazoaVertebrateEP00067Daniorerio_5755401_5HT3A_CAVPO_5_hydroxytryptamine_receptor_3A_OS_Cavia_porcellus_OX_10141_GN_HTR3A_PE_1_SV_
MetazoaVertebrateEP00067Daniorerio_5743783_5HT3A_HUMAN_5_hydroxytryptamine_receptor_3A_OS_Homo_sapiens_OX_9606_GN_HTR3A_PE_1_SV_
MetazoaVertebrateEP00066Takifugurubripes_5696740_
MetazoaVertebrateHomosapiens_6459549_ZACN_HUMAN_Zinc_activated_ligand_gated_ion_channel_OS_Homo_sapiens_OX_9606_GN_ZACN_PE_1_SV_
MetazoaVertebrateMonodelphisdomestica_6497698_ZACN_CANLF_Zinc_activated_ligand_gated_ion_channel_OS_Canis_lupus_familiaris_OX_9615_GN_ZACN_PE_3_SV_
0.48

16D.

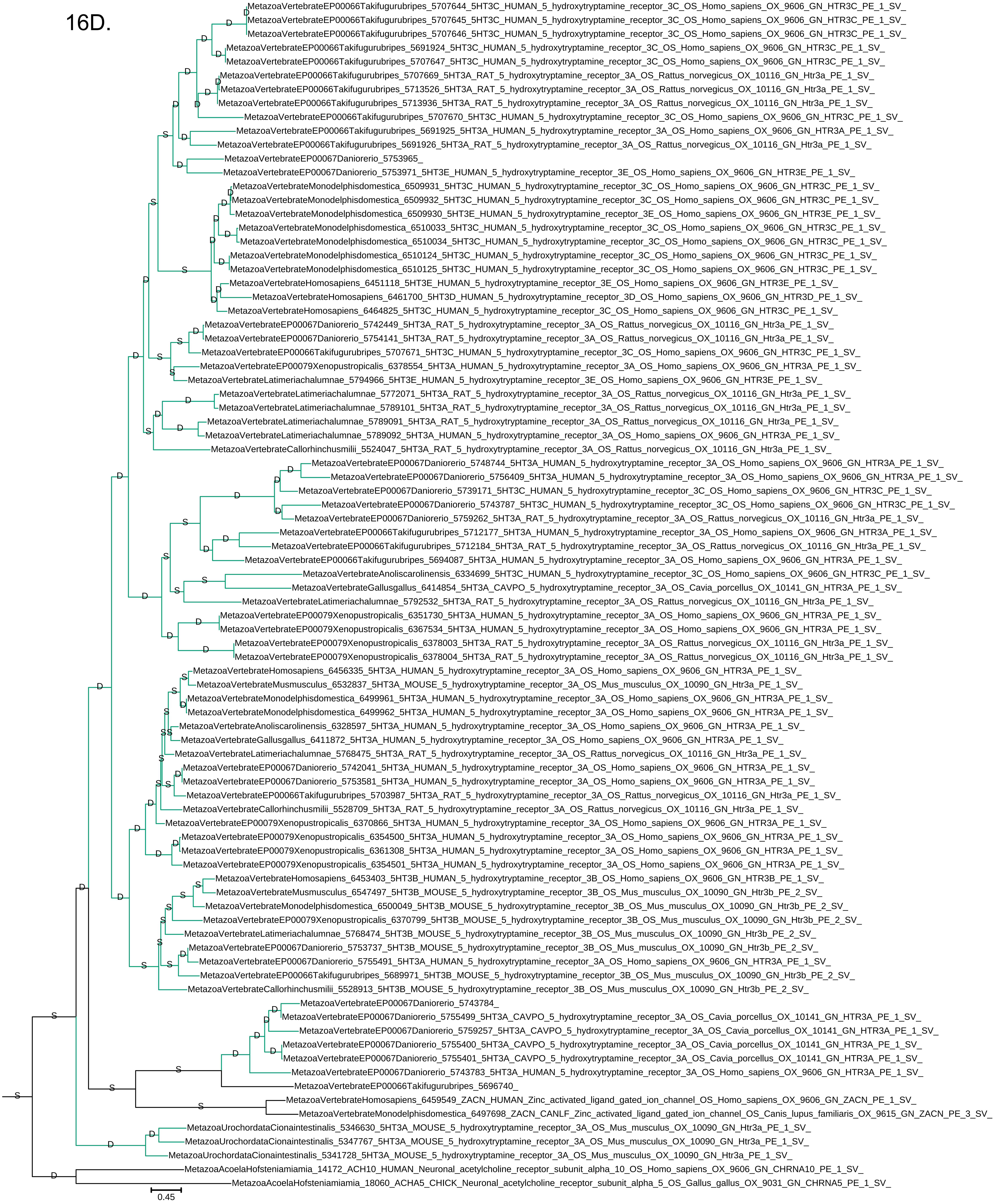

MetazoaVertebrateEP00066Takifugurubripes_5707644_5HT3C_HUMAN_5_hydroxytryptamine_receptor_3C_OS_Homo_sapiens_OX_9606_GN_HTR3C_PE_1_SV_
MetazoaVertebrateEP00066Takifugurubripes_5707645_5HT3C_HUMAN_5_hydroxytryptamine_receptor_3C_OS_Homo_sapiens_OX_9606_GN_HTR3C_PE_1_SV_
MetazoaVertebrateEP00066Takifugurubripes_5707646_5HT3C_HUMAN_5_hydroxytryptamine_receptor_3C_OS_Homo_sapiens_OX_9606_GN_HTR3C_PE_1_SV_
MetazoaVertebrateEP00066Takifugurubripes_5691924_5HT3C_HUMAN_5_hydroxytryptamine_receptor_3C_OS_Homo_sapiens_OX_9606_GN_HTR3C_PE_1_SV_
MetazoaVertebrateEP00066Takifugurubripes_5707647_5HT3C_HUMAN_5_hydroxytryptamine_receptor_3C_OS_Homo_sapiens_OX_9606_GN_HTR3C_PE_1_SV_
MetazoaVertebrateEP00066Takifugurubripes_5707669_5HT3A_RAT_5_hydroxytryptamine_receptor_3A_OS_Rattus_norvegicus_OX_10116_GN_Htr3a_PE_1_SV_
MetazoaVertebrateEP00066Takifugurubripes_5713526_5HT3A_RAT_5_hydroxytryptamine_receptor_3A_OS_Rattus_norvegicus_OX_10116_GN_Htr3a_PE_1_SV_
MetazoaVertebrateEP00066Takifugurubripes_5713936_5HT3A_RAT_5_hydroxytryptamine_receptor_3A_OS_Rattus_norvegicus_OX_10116_GN_Htr3a_PE_1_SV_
MetazoaVertebrateEP00066Takifugurubripes_5707670_5HT3C_HUMAN_5_hydroxytryptamine_receptor_3C_OS_Homo_sapiens_OX_9606_GN_HTR3C_PE_1_SV_
MetazoaVertebrateEP00066Takifugurubripes_5691925_5HT3A_HUMAN_5_hydroxytryptamine_receptor_3A_OS_Homo_sapiens_OX_9606_GN_HTR3A_PE_1_SV_
MetazoaVertebrateEP00066Takifugurubripes_5691926_5HT3A_RAT_5_hydroxytryptamine_receptor_3A_OS_Rattus_norvegicus_OX_10116_GN_Htr3a_PE_1_SV_
MetazoaVertebrateEP00067Daniorerio_5753965_
MetazoaVertebrateEP00067Daniorerio_5753971_5HT3E_HUMAN_5_hydroxytryptamine_receptor_3E_OS_Homo_sapiens_OX_9606_GN_HTR3E_PE_1_SV_
MetazoaVertebrateMonodelphisdomestica_6509931_5HT3C_HUMAN_5_hydroxytryptamine_receptor_3C_OS_Homo_sapiens_OX_9606_GN_HTR3C_PE_1_SV_
MetazoaVertebrateMonodelphisdomestica_6509932_5HT3C_HUMAN_5_hydroxytryptamine_receptor_3C_OS_Homo_sapiens_OX_9606_GN_HTR3C_PE_1_SV_
MetazoaVertebrateMonodelphisdomestica_6509930_5HT3E_HUMAN_5_hydroxytryptamine_receptor_3E_OS_Homo_sapiens_OX_9606_GN_HTR3E_PE_1_SV_
MetazoaVertebrateMonodelphisdomestica_6510033_5HT3C_HUMAN_5_hydroxytryptamine_receptor_3C_OS_Homo_sapiens_OX_9606_GN_HTR3C_PE_1_SV_
MetazoaVertebrateMonodelphisdomestica_6510034_5HT3C_HUMAN_5_hydroxytryptamine_receptor_3C_OS_Homo_sapiens_OX_9606_GN_HTR3C_PE_1_SV_
MetazoaVertebrateMonodelphisdomestica_6510124_5HT3C_HUMAN_5_hydroxytryptamine_receptor_3C_OS_Homo_sapiens_OX_9606_GN_HTR3C_PE_1_SV_
MetazoaVertebrateMonodelphisdomestica_6510125_5HT3C_HUMAN_5_hydroxytryptamine_receptor_3C_OS_Homo_sapiens_OX_9606_GN_HTR3C_PE_1_SV_
MetazoaVertebrateHomosapiens_6451118_5HT3E_HUMAN_5_hydroxytryptamine_receptor_3E_OS_Homo_sapiens_OX_9606_GN_HTR3E_PE_1_SV_
MetazoaVertebrateHomosapiens_6461700_5HT3D_HUMAN_5_hydroxytryptamine_receptor_3D_OS_Homo_sapiens_OX_9606_GN_HTR3D_PE_1_SV_
MetazoaVertebrateHomosapiens_6464825_5HT3C_HUMAN_5_hydroxytryptamine_receptor_3C_OS_Homo_sapiens_OX_9606_GN_HTR3C_PE_1_SV_
MetazoaVertebrateEP00067Daniorerio_5742449_5HT3A_RAT_5_hydroxytryptamine_receptor_3A_OS_Rattus_norvegicus_OX_10116_GN_Htr3a_PE_1_SV_
MetazoaVertebrateEP00067Daniorerio_5754141_5HT3A_RAT_5_hydroxytryptamine_receptor_3A_OS_Rattus_norvegicus_OX_10116_GN_Htr3a_PE_1_SV_
MetazoaVertebrateEP00066Takifugurubripes_5707671_5HT3C_HUMAN_5_hydroxytryptamine_receptor_3C_OS_Homo_sapiens_OX_9606_GN_HTR3C_PE_1_SV_
MetazoaVertebrateEP00079Xenopustropicalis_6378554_5HT3A_HUMAN_5_hydroxytryptamine_receptor_3A_OS_Homo_sapiens_OX_9606_GN_HTR3A_PE_1_SV_
MetazoaVertebrateLatimeriachalumnae_5794966_5HT3E_HUMAN_5_hydroxytryptamine_receptor_3E_OS_Homo_sapiens_OX_9606_GN_HTR3E_PE_1_SV_
MetazoaVertebrateLatimeriachalumnae_5772071_5HT3A_RAT_5_hydroxytryptamine_receptor_3A_OS_Rattus_norvegicus_OX_10116_GN_Htr3a_PE_1_SV_
MetazoaVertebrateLatimeriachalumnae_5789101_5HT3A_RAT_5_hydroxytryptamine_receptor_3A_OS_Rattus_norvegicus_OX_10116_GN_Htr3a_PE_1_SV_
MetazoaVertebrateLatimeriachalumnae_5789091_5HT3A_RAT_5_hydroxytryptamine_receptor_3A_OS_Rattus_norvegicus_OX_10116_GN_Htr3a_PE_1_SV_
MetazoaVertebrateLatimeriachalumnae_5789092_5HT3A_HUMAN_5_hydroxytryptamine_receptor_3A_OS_Homo_sapiens_OX_9606_GN_HTR3A_PE_1_SV_
MetazoaVertebrateCallorhinchusmilii_5524047_5HT3A_RAT_5_hydroxytryptamine_receptor_3A_OS_Rattus_norvegicus_OX_10116_GN_Htr3a_PE_1_SV_
MetazoaVertebrateEP00067Daniorerio_5748744_5HT3A_HUMAN_5_hydroxytryptamine_receptor_3A_OS_Homo_sapiens_OX_9606_GN_HTR3A_PE_1_SV_
MetazoaVertebrateEP00067Daniorerio_5756409_5HT3A_HUMAN_5_hydroxytryptamine_receptor_3A_OS_Homo_sapiens_OX_9606_GN_HTR3A_PE_1_SV_
MetazoaVertebrateEP00067Daniorerio_5739171_5HT3C_HUMAN_5_hydroxytryptamine_receptor_3C_OS_Homo_sapiens_OX_9606_GN_HTR3C_PE_1_SV_
MetazoaVertebrateEP00067Daniorerio_5743787_5HT3C_HUMAN_5_hydroxytryptamine_receptor_3C_OS_Homo_sapiens_OX_9606_GN_HTR3C_PE_1_SV_
MetazoaVertebrateEP00067Daniorerio_5759262_5HT3A_RAT_5_hydroxytryptamine_receptor_3A_OS_Rattus_norvegicus_OX_10116_GN_Htr3a_PE_1_SV_
MetazoaVertebrateEP00066Takifugurubripes_5712177_5HT3A_HUMAN_5_hydroxytryptamine_receptor_3A_OS_Homo_sapiens_OX_9606_GN_HTR3A_PE_1_SV_
MetazoaVertebrateEP00066Takifugurubripes_5712184_5HT3A_RAT_5_hydroxytryptamine_receptor_3A_OS_Rattus_norvegicus_OX_10116_GN_Htr3a_PE_1_SV_
MetazoaVertebrateEP00066Takifugurubripes_5694087_5HT3A_HUMAN_5_hydroxytryptamine_receptor_3A_OS_Homo_sapiens_OX_9606_GN_HTR3A_PE_1_SV_
MetazoaVertebrateAnoliscarolinensis_6334699_5HT3C_HUMAN_5_hydroxytryptamine_receptor_3C_OS_Homo_sapiens_OX_9606_GN_HTR3C_PE_1_SV_
MetazoaVertebrateGallusgallus_6414854_5HT3A_CAVPO_5_hydroxytryptamine_receptor_3A_OS_Cavia_porcellus_OX_10141_GN_HTR3A_PE_1_SV_
MetazoaVertebrateLatimeriachalumnae_5792532_5HT3A_RAT_5_hydroxytryptamine_receptor_3A_OS_Rattus_norvegicus_OX_10116_GN_Htr3a_PE_1_SV_
MetazoaVertebrateEP00079Xenopustropicalis_6351730_5HT3A_HUMAN_5_hydroxytryptamine_receptor_3A_OS_Homo_sapiens_OX_9606_GN_HTR3A_PE_1_SV_
MetazoaVertebrateEP00079Xenopustropicalis_6367534_5HT3A_HUMAN_5_hydroxytryptamine_receptor_3A_OS_Homo_sapiens_OX_9606_GN_HTR3A_PE_1_SV_
MetazoaVertebrateEP00079Xenopustropicalis_6378003_5HT3A_RAT_5_hydroxytryptamine_receptor_3A_OS_Rattus_norvegicus_OX_10116_GN_Htr3a_PE_1_SV_
MetazoaVertebrateEP00079Xenopustropicalis_6378004_5HT3A_RAT_5_hydroxytryptamine_receptor_3A_OS_Rattus_norvegicus_OX_10116_GN_Htr3a_PE_1_SV_
MetazoaVertebrateHomosapiens_6456335_5HT3A_HUMAN_5_hydroxytryptamine_receptor_3A_OS_Homo_sapiens_OX_9606_GN_HTR3A_PE_1_SV_
MetazoaVertebrateMusmusculus_6532837_5HT3A_MOUSE_5_hydroxytryptamine_receptor_3A_OS_Mus_musculus_OX_10090_GN_Htr3a_PE_1_SV_
MetazoaVertebrateMonodelphisdomestica_6499961_5HT3A_HUMAN_5_hydroxytryptamine_receptor_3A_OS_Homo_sapiens_OX_9606_GN_HTR3A_PE_1_SV_
MetazoaVertebrateMonodelphisdomestica_6499962_5HT3A_HUMAN_5_hydroxytryptamine_receptor_3A_OS_Homo_sapiens_OX_9606_GN_HTR3A_PE_1_SV_
MetazoaVertebrateAnoliscarolinensis_6328597_5HT3A_HUMAN_5_hydroxytryptamine_receptor_3A_OS_Homo_sapiens_OX_9606_GN_HTR3A_PE_1_SV_
MetazoaVertebrateGallusgallus_6411872_5HT3A_HUMAN_5_hydroxytryptamine_receptor_3A_OS_Homo_sapiens_OX_9606_GN_HTR3A_PE_1_SV_
MetazoaVertebrateLatimeriachalumnae_5768475_5HT3A_RAT_5_hydroxytryptamine_receptor_3A_OS_Rattus_norvegicus_OX_10116_GN_Htr3a_PE_1_SV_
MetazoaVertebrateEP00067Daniorerio_5742041_5HT3A_HUMAN_5_hydroxytryptamine_receptor_3A_OS_Homo_sapiens_OX_9606_GN_HTR3A_PE_1_SV_
MetazoaVertebrateEP00067Daniorerio_5753581_5HT3A_HUMAN_5_hydroxytryptamine_receptor_3A_OS_Homo_sapiens_OX_9606_GN_HTR3A_PE_1_SV_
MetazoaVertebrateEP00066Takifugurubripes_5703987_5HT3A_RAT_5_hydroxytryptamine_receptor_3A_OS_Rattus_norvegicus_OX_10116_GN_Htr3a_PE_1_SV_
MetazoaVertebrateCallorhinchusmilii_5528709_5HT3A_RAT_5_hydroxytryptamine_receptor_3A_OS_Rattus_norvegicus_OX_10116_GN_Htr3a_PE_1_SV_
MetazoaVertebrateEP00079Xenopustropicalis_6370866_5HT3A_HUMAN_5_hydroxytryptamine_receptor_3A_OS_Homo_sapiens_OX_9606_GN_HTR3A_PE_1_SV_
MetazoaVertebrateEP00079Xenopustropicalis_6354500_5HT3A_HUMAN_5_hydroxytryptamine_receptor_3A_OS_Homo_sapiens_OX_9606_GN_HTR3A_PE_1_SV_
MetazoaVertebrateEP00079Xenopustropicalis_6361308_5HT3A_HUMAN_5_hydroxytryptamine_receptor_3A_OS_Homo_sapiens_OX_9606_GN_HTR3A_PE_1_SV_
MetazoaVertebrateEP00079Xenopustropicalis_6354501_5HT3A_HUMAN_5_hydroxytryptamine_receptor_3A_OS_Homo_sapiens_OX_9606_GN_HTR3A_PE_1_SV_
MetazoaVertebrateHomosapiens_6453403_5HT3B_HUMAN_5_hydroxytryptamine_receptor_3B_OS_Homo_sapiens_OX_9606_GN_HTR3B_PE_1_SV_
MetazoaVertebrateMusmusculus_6547497_5HT3B_MOUSE_5_hydroxytryptamine_receptor_3B_OS_Mus_musculus_OX_10090_GN_Htr3b_PE_2_SV_
MetazoaVertebrateMonodelphisdomestica_6500049_5HT3B_MOUSE_5_hydroxytryptamine_receptor_3B_OS_Mus_musculus_OX_10090_GN_Htr3b_PE_2_SV_
MetazoaVertebrateEP00079Xenopustropicalis_6370799_5HT3B_MOUSE_5_hydroxytryptamine_receptor_3B_OS_Mus_musculus_OX_10090_GN_Htr3b_PE_2_SV_
MetazoaVertebrateLatimeriachalumnae_5768474_5HT3B_MOUSE_5_hydroxytryptamine_receptor_3B_OS_Mus_musculus_OX_10090_GN_Htr3b_PE_2_SV_
MetazoaVertebrateEP00067Daniorerio_5753737_5HT3B_MOUSE_5_hydroxytryptamine_receptor_3B_OS_Mus_musculus_OX_10090_GN_Htr3b_PE_2_SV_
MetazoaVertebrateEP00067Daniorerio_5755491_5HT3A_HUMAN_5_hydroxytryptamine_receptor_3A_OS_Homo_sapiens_OX_9606_GN_HTR3A_PE_1_SV_
MetazoaVertebrateEP00066Takifugurubripes_5689971_5HT3B_MOUSE_5_hydroxytryptamine_receptor_3B_OS_Mus_musculus_OX_10090_GN_Htr3b_PE_2_SV_
MetazoaVertebrateCallorhinchusmilii_5528913_5HT3B_MOUSE_5_hydroxytryptamine_receptor_3B_OS_Mus_musculus_OX_10090_GN_Htr3b_PE_2_SV_
MetazoaVertebrateEP00067Daniorerio_5743784_
MetazoaVertebrateEP00067Daniorerio_5755499_5HT3A_CAVPO_5_hydroxytryptamine_receptor_3A_OS_Cavia_porcellus_OX_10141_GN_HTR3A_PE_1_SV_
MetazoaVertebrateEP00067Daniorerio_5759257_5HT3A_CAVPO_5_hydroxytryptamine_receptor_3A_OS_Cavia_porcellus_OX_10141_GN_HTR3A_PE_1_SV_
MetazoaVertebrateEP00067Daniorerio_5755400_5HT3A_CAVPO_5_hydroxytryptamine_receptor_3A_OS_Cavia_porcellus_OX_10141_GN_HTR3A_PE_1_SV_
MetazoaVertebrateEP00067Daniorerio_5755401_5HT3A_CAVPO_5_hydroxytryptamine_receptor_3A_OS_Cavia_porcellus_OX_10141_GN_HTR3A_PE_1_SV_
MetazoaVertebrateEP00067Daniorerio_5743783_5HT3A_HUMAN_5_hydroxytryptamine_receptor_3A_OS_Homo_sapiens_OX_9606_GN_HTR3A_PE_1_SV_
MetazoaVertebrateEP00066Takifugurubripes_5696740_
MetazoaVertebrateHomosapiens_6459549_ZACN_HUMAN_Zinc_activated_ligand_gated_ion_channel_OS_Homo_sapiens_OX_9606_GN_ZACN_PE_1_SV_
MetazoaVertebrateMonodelphisdomestica_6497698_ZACN_CANLF_Zinc_activated_ligand_gated_ion_channel_OS_Canis_lupus_familiaris_OX_9615_GN_ZACN_PE_3_SV_
MetazoaUrochordataCionaintestinalis_5346630_5HT3A_MOUSE_5_hydroxytryptamine_receptor_3A_OS_Mus_musculus_OX_10090_GN_Htr3a_PE_1_SV_
MetazoaUrochordataCionaintestinalis_5347767_5HT3A_MOUSE_5_hydroxytryptamine_receptor_3A_OS_Mus_musculus_OX_10090_GN_Htr3a_PE_1_SV_
MetazoaUrochordataCionaintestinalis_5341728_5HT3A_MOUSE_5_hydroxytryptamine_receptor_3A_OS_Mus_musculus_OX_10090_GN_Htr3a_PE_1_SV_
MetazoaAcoelaHofsteniamiamia_14172_ACH10_HUMAN_Neuronal_acetylcholine_receptor_subunit_alpha_10_OS_Homo_sapiens_OX_9606_GN_CHRNA10_PE_1_SV_
MetazoaAcoelaHofsteniamiamia_18060_ACHA5_CHICK_Neuronal_acetylcholine_receptor_subunit_alpha_5_OS_Gallus_gallus_OX_9031_GN_CHRNA5_PE_1_SV_
0.45

16E.

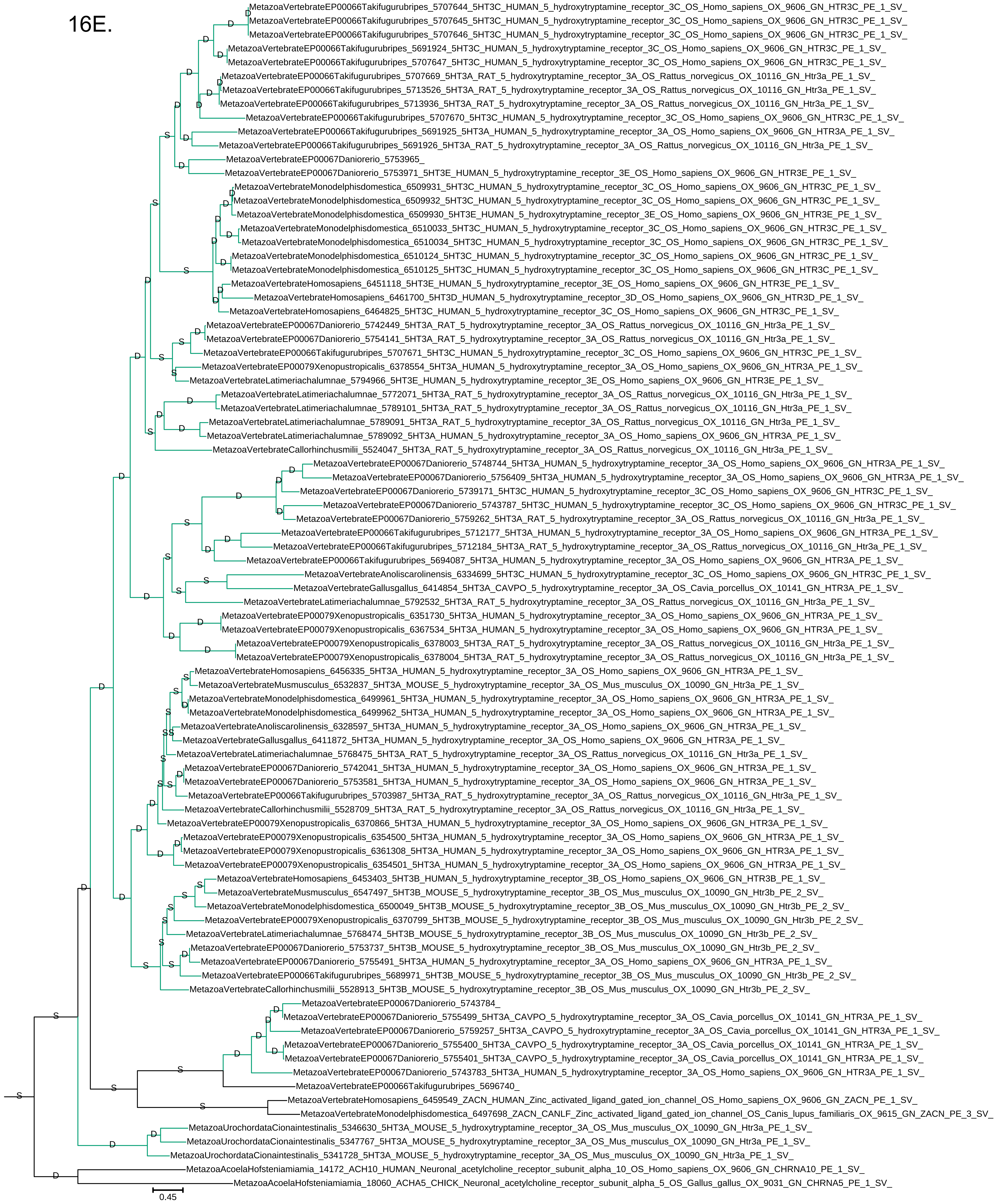
MetazoaVertebrateEP00066Takifugurubripes_5707644_5HT3C_HUMAN_5_hydroxytryptamine_receptor_3C_OS_Homo_sapiens_OX_9606_GN_HTR3C_PE_1_SV_
MetazoaVertebrateEP00066Takifugurubripes_5707645_5HT3C_HUMAN_5_hydroxytryptamine_receptor_3C_OS_Homo_sapiens_OX_9606_GN_HTR3C_PE_1_SV_
MetazoaVertebrateEP00066Takifugurubripes_5707646_5HT3C_HUMAN_5_hydroxytryptamine_receptor_3C_OS_Homo_sapiens_OX_9606_GN_HTR3C_PE_1_SV_
MetazoaVertebrateEP00066Takifugurubripes_5691924_5HT3C_HUMAN_5_hydroxytryptamine_receptor_3C_OS_Homo_sapiens_OX_9606_GN_HTR3C_PE_1_SV_
MetazoaVertebrateEP00066Takifugurubripes_5707647_5HT3C_HUMAN_5_hydroxytryptamine_receptor_3C_OS_Homo_sapiens_OX_9606_GN_HTR3C_PE_1_SV_
MetazoaVertebrateEP00066Takifugurubripes_5707669_5HT3A_RAT_5_hydroxytryptamine_receptor_3A_OS_Rattus_norvegicus_OX_10116_GN_Htr3a_PE_1_SV_
MetazoaVertebrateEP00066Takifugurubripes_5713526_5HT3A_RAT_5_hydroxytryptamine_receptor_3A_OS_Rattus_norvegicus_OX_10116_GN_Htr3a_PE_1_SV_
MetazoaVertebrateEP00066Takifugurubripes_5713936_5HT3A_RAT_5_hydroxytryptamine_receptor_3A_OS_Rattus_norvegicus_OX_10116_GN_Htr3a_PE_1_SV_
MetazoaVertebrateEP00066Takifugurubripes_5707670_5HT3C_HUMAN_5_hydroxytryptamine_receptor_3C_OS_Homo_sapiens_OX_9606_GN_HTR3C_PE_1_SV_
MetazoaVertebrateEP00066Takifugurubripes_5691925_5HT3A_HUMAN_5_hydroxytryptamine_receptor_3A_OS_Homo_sapiens_OX_9606_GN_HTR3A_PE_1_SV_
MetazoaVertebrateEP00066Takifugurubripes_5691926_5HT3A_RAT_5_hydroxytryptamine_receptor_3A_OS_Rattus_norvegicus_OX_10116_GN_Htr3a_PE_1_SV_
MetazoaVertebrateEP00067Daniorerio_5753965_
MetazoaVertebrateEP00067Daniorerio_5753971_5HT3E_HUMAN_5_hydroxytryptamine_receptor_3E_OS_Homo_sapiens_OX_9606_GN_HTR3E_PE_1_SV_
MetazoaVertebrateMonodelphisdomestica_6509931_5HT3C_HUMAN_5_hydroxytryptamine_receptor_3C_OS_Homo_sapiens_OX_9606_GN_HTR3C_PE_1_SV_
MetazoaVertebrateMonodelphisdomestica_6509932_5HT3C_HUMAN_5_hydroxytryptamine_receptor_3C_OS_Homo_sapiens_OX_9606_GN_HTR3C_PE_1_SV_
MetazoaVertebrateMonodelphisdomestica_6509930_5HT3E_HUMAN_5_hydroxytryptamine_receptor_3E_OS_Homo_sapiens_OX_9606_GN_HTR3E_PE_1_SV_
MetazoaVertebrateMonodelphisdomestica_6510033_5HT3C_HUMAN_5_hydroxytryptamine_receptor_3C_OS_Homo_sapiens_OX_9606_GN_HTR3C_PE_1_SV_
MetazoaVertebrateMonodelphisdomestica_6510034_5HT3C_HUMAN_5_hydroxytryptamine_receptor_3C_OS_Homo_sapiens_OX_9606_GN_HTR3C_PE_1_SV_
MetazoaVertebrateMonodelphisdomestica_6510124_5HT3C_HUMAN_5_hydroxytryptamine_receptor_3C_OS_Homo_sapiens_OX_9606_GN_HTR3C_PE_1_SV_
MetazoaVertebrateMonodelphisdomestica_6510125_5HT3C_HUMAN_5_hydroxytryptamine_receptor_3C_OS_Homo_sapiens_OX_9606_GN_HTR3C_PE_1_SV_
MetazoaVertebrateHomosapiens_6451118_5HT3E_HUMAN_5_hydroxytryptamine_receptor_3E_OS_Homo_sapiens_OX_9606_GN_HTR3E_PE_1_SV_
MetazoaVertebrateHomosapiens_6461700_5HT3D_HUMAN_5_hydroxytryptamine_receptor_3D_OS_Homo_sapiens_OX_9606_GN_HTR3D_PE_1_SV_
MetazoaVertebrateHomosapiens_6464825_5HT3C_HUMAN_5_hydroxytryptamine_receptor_3C_OS_Homo_sapiens_OX_9606_GN_HTR3C_PE_1_SV_
MetazoaVertebrateEP00067Daniorerio_5742449_5HT3A_RAT_5_hydroxytryptamine_receptor_3A_OS_Rattus_norvegicus_OX_10116_GN_Htr3a_PE_1_SV_
MetazoaVertebrateEP00067Daniorerio_5754141_5HT3A_RAT_5_hydroxytryptamine_receptor_3A_OS_Rattus_norvegicus_OX_10116_GN_Htr3a_PE_1_SV_
MetazoaVertebrateEP00066Takifugurubripes_5707671_5HT3C_HUMAN_5_hydroxytryptamine_receptor_3C_OS_Homo_sapiens_OX_9606_GN_HTR3C_PE_1_SV_
MetazoaVertebrateEP00079Xenopustropicalis_6378554_5HT3A_HUMAN_5_hydroxytryptamine_receptor_3A_OS_Homo_sapiens_OX_9606_GN_HTR3A_PE_1_SV_
MetazoaVertebrateLatimeriachalumnae_5794966_5HT3E_HUMAN_5_hydroxytryptamine_receptor_3E_OS_Homo_sapiens_OX_9606_GN_HTR3E_PE_1_SV_
MetazoaVertebrateLatimeriachalumnae_5772071_5HT3A_RAT_5_hydroxytryptamine_receptor_3A_OS_Rattus_norvegicus_OX_10116_GN_Htr3a_PE_1_SV_
MetazoaVertebrateLatimeriachalumnae_5789101_5HT3A_RAT_5_hydroxytryptamine_receptor_3A_OS_Rattus_norvegicus_OX_10116_GN_Htr3a_PE_1_SV_
MetazoaVertebrateLatimeriachalumnae_5789091_5HT3A_RAT_5_hydroxytryptamine_receptor_3A_OS_Rattus_norvegicus_OX_10116_GN_Htr3a_PE_1_SV_
MetazoaVertebrateLatimeriachalumnae_5789092_5HT3A_HUMAN_5_hydroxytryptamine_receptor_3A_OS_Homo_sapiens_OX_9606_GN_HTR3A_PE_1_SV_
MetazoaVertebrateCallorhinchusmilii_5524047_5HT3A_RAT_5_hydroxytryptamine_receptor_3A_OS_Rattus_norvegicus_OX_10116_GN_Htr3a_PE_1_SV_
MetazoaVertebrateEP00067Daniorerio_5748744_5HT3A_HUMAN_5_hydroxytryptamine_receptor_3A_OS_Homo_sapiens_OX_9606_GN_HTR3A_PE_1_SV_
MetazoaVertebrateEP00067Daniorerio_5756409_5HT3A_HUMAN_5_hydroxytryptamine_receptor_3A_OS_Homo_sapiens_OX_9606_GN_HTR3A_PE_1_SV_
MetazoaVertebrateEP00067Daniorerio_5739171_5HT3C_HUMAN_5_hydroxytryptamine_receptor_3C_OS_Homo_sapiens_OX_9606_GN_HTR3C_PE_1_SV_
MetazoaVertebrateEP00067Daniorerio_5743787_5HT3C_HUMAN_5_hydroxytryptamine_receptor_3C_OS_Homo_sapiens_OX_9606_GN_HTR3C_PE_1_SV_
MetazoaVertebrateEP00067Daniorerio_5759262_5HT3A_RAT_5_hydroxytryptamine_receptor_3A_OS_Rattus_norvegicus_OX_10116_GN_Htr3a_PE_1_SV_
MetazoaVertebrateEP00066Takifugurubripes_5712177_5HT3A_HUMAN_5_hydroxytryptamine_receptor_3A_OS_Homo_sapiens_OX_9606_GN_HTR3A_PE_1_SV_
MetazoaVertebrateEP00066Takifugurubripes_5712184_5HT3A_RAT_5_hydroxytryptamine_receptor_3A_OS_Rattus_norvegicus_OX_10116_GN_Htr3a_PE_1_SV_
MetazoaVertebrateEP00066Takifugurubripes_5694087_5HT3A_HUMAN_5_hydroxytryptamine_receptor_3A_OS_Homo_sapiens_OX_9606_GN_HTR3A_PE_1_SV_
MetazoaVertebrateAnoliscarolinensis_6334699_5HT3C_HUMAN_5_hydroxytryptamine_receptor_3C_OS_Homo_sapiens_OX_9606_GN_HTR3C_PE_1_SV_
MetazoaVertebrateGallusgallus_6414854_5HT3A_CAVPO_5_hydroxytryptamine_receptor_3A_OS_Cavia_porcellus_OX_10141_GN_HTR3A_PE_1_SV_
MetazoaVertebrateLatimeriachalumnae_5792532_5HT3A_RAT_5_hydroxytryptamine_receptor_3A_OS_Rattus_norvegicus_OX_10116_GN_Htr3a_PE_1_SV_
MetazoaVertebrateEP00079Xenopustropicalis_6351730_5HT3A_HUMAN_5_hydroxytryptamine_receptor_3A_OS_Homo_sapiens_OX_9606_GN_HTR3A_PE_1_SV_
MetazoaVertebrateEP00079Xenopustropicalis_6367534_5HT3A_HUMAN_5_hydroxytryptamine_receptor_3A_OS_Homo_sapiens_OX_9606_GN_HTR3A_PE_1_SV_
MetazoaVertebrateEP00079Xenopustropicalis_6378003_5HT3A_RAT_5_hydroxytryptamine_receptor_3A_OS_Rattus_norvegicus_OX_10116_GN_Htr3a_PE_1_SV_
MetazoaVertebrateEP00079Xenopustropicalis_6378004_5HT3A_RAT_5_hydroxytryptamine_receptor_3A_OS_Rattus_norvegicus_OX_10116_GN_Htr3a_PE_1_SV_
MetazoaVertebrateHomosapiens_6456335_5HT3A_HUMAN_5_hydroxytryptamine_receptor_3A_OS_Homo_sapiens_OX_9606_GN_HTR3A_PE_1_SV_
MetazoaVertebrateMusmusculus_6532837_5HT3A_MOUSE_5_hydroxytryptamine_receptor_3A_OS_Mus_musculus_OX_10090_GN_Htr3a_PE_1_SV_
MetazoaVertebrateMonodelphisdomestica_6499961_5HT3A_HUMAN_5_hydroxytryptamine_receptor_3A_OS_Homo_sapiens_OX_9606_GN_HTR3A_PE_1_SV_
MetazoaVertebrateMonodelphisdomestica_6499962_5HT3A_HUMAN_5_hydroxytryptamine_receptor_3A_OS_Homo_sapiens_OX_9606_GN_HTR3A_PE_1_SV_
MetazoaVertebrateAnoliscarolinensis_6328597_5HT3A_HUMAN_5_hydroxytryptamine_receptor_3A_OS_Homo_sapiens_OX_9606_GN_HTR3A_PE_1_SV_
MetazoaVertebrateGallusgallus_6411872_5HT3A_HUMAN_5_hydroxytryptamine_receptor_3A_OS_Homo_sapiens_OX_9606_GN_HTR3A_PE_1_SV_
MetazoaVertebrateLatimeriachalumnae_5768475_5HT3A_RAT_5_hydroxytryptamine_receptor_3A_OS_Rattus_norvegicus_OX_10116_GN_Htr3a_PE_1_SV_
MetazoaVertebrateEP00067Daniorerio_5742041_5HT3A_HUMAN_5_hydroxytryptamine_receptor_3A_OS_Homo_sapiens_OX_9606_GN_HTR3A_PE_1_SV_
MetazoaVertebrateEP00067Daniorerio_5753581_5HT3A_HUMAN_5_hydroxytryptamine_receptor_3A_OS_Homo_sapiens_OX_9606_GN_HTR3A_PE_1_SV_
MetazoaVertebrateEP00066Takifugurubripes_5703987_5HT3A_RAT_5_hydroxytryptamine_receptor_3A_OS_Rattus_norvegicus_OX_10116_GN_Htr3a_PE_1_SV_
MetazoaVertebrateCallorhinchusmilii_5528709_5HT3A_RAT_5_hydroxytryptamine_receptor_3A_OS_Rattus_norvegicus_OX_10116_GN_Htr3a_PE_1_SV_
MetazoaVertebrateEP00079Xenopustropicalis_6370866_5HT3A_HUMAN_5_hydroxytryptamine_receptor_3A_OS_Homo_sapiens_OX_9606_GN_HTR3A_PE_1_SV_
MetazoaVertebrateEP00079Xenopustropicalis_6354500_5HT3A_HUMAN_5_hydroxytryptamine_receptor_3A_OS_Homo_sapiens_OX_9606_GN_HTR3A_PE_1_SV_
MetazoaVertebrateEP00079Xenopustropicalis_6361308_5HT3A_HUMAN_5_hydroxytryptamine_receptor_3A_OS_Homo_sapiens_OX_9606_GN_HTR3A_PE_1_SV_
MetazoaVertebrateEP00079Xenopustropicalis_6354501_5HT3A_HUMAN_5_hydroxytryptamine_receptor_3A_OS_Homo_sapiens_OX_9606_GN_HTR3A_PE_1_SV_
MetazoaVertebrateHomosapiens_6453403_5HT3B_HUMAN_5_hydroxytryptamine_receptor_3B_OS_Homo_sapiens_OX_9606_GN_HTR3B_PE_1_SV_
MetazoaVertebrateMusmusculus_6547497_5HT3B_MOUSE_5_hydroxytryptamine_receptor_3B_OS_Mus_musculus_OX_10090_GN_Htr3b_PE_2_SV_
MetazoaVertebrateMonodelphisdomestica_6500049_5HT3B_MOUSE_5_hydroxytryptamine_receptor_3B_OS_Mus_musculus_OX_10090_GN_Htr3b_PE_2_SV_
MetazoaVertebrateEP00079Xenopustropicalis_6370799_5HT3B_MOUSE_5_hydroxytryptamine_receptor_3B_OS_Mus_musculus_OX_10090_GN_Htr3b_PE_2_SV_
MetazoaVertebrateLatimeriachalumnae_5768474_5HT3B_MOUSE_5_hydroxytryptamine_receptor_3B_OS_Mus_musculus_OX_10090_GN_Htr3b_PE_2_SV_
MetazoaVertebrateEP00067Daniorerio_5753737_5HT3B_MOUSE_5_hydroxytryptamine_receptor_3B_OS_Mus_musculus_OX_10090_GN_Htr3b_PE_2_SV_
MetazoaVertebrateEP00067Daniorerio_5755491_5HT3A_HUMAN_5_hydroxytryptamine_receptor_3A_OS_Homo_sapiens_OX_9606_GN_HTR3A_PE_1_SV_
MetazoaVertebrateEP00066Takifugurubripes_5689971_5HT3B_MOUSE_5_hydroxytryptamine_receptor_3B_OS_Mus_musculus_OX_10090_GN_Htr3b_PE_2_SV_
MetazoaVertebrateCallorhinchusmilii_5528913_5HT3B_MOUSE_5_hydroxytryptamine_receptor_3B_OS_Mus_musculus_OX_10090_GN_Htr3b_PE_2_SV_
MetazoaVertebrateEP00067Daniorerio_5743784_
MetazoaVertebrateEP00067Daniorerio_5755499_5HT3A_CAVPO_5_hydroxytryptamine_receptor_3A_OS_Cavia_porcellus_OX_10141_GN_HTR3A_PE_1_SV_
MetazoaVertebrateEP00067Daniorerio_5759257_5HT3A_CAVPO_5_hydroxytryptamine_receptor_3A_OS_Cavia_porcellus_OX_10141_GN_HTR3A_PE_1_SV_
MetazoaVertebrateEP00067Daniorerio_5755400_5HT3A_CAVPO_5_hydroxytryptamine_receptor_3A_OS_Cavia_porcellus_OX_10141_GN_HTR3A_PE_1_SV_
MetazoaVertebrateEP00067Daniorerio_5755401_5HT3A_CAVPO_5_hydroxytryptamine_receptor_3A_OS_Cavia_porcellus_OX_10141_GN_HTR3A_PE_1_SV_
MetazoaVertebrateEP00067Daniorerio_5743783_5HT3A_HUMAN_5_hydroxytryptamine_receptor_3A_OS_Homo_sapiens_OX_9606_GN_HTR3A_PE_1_SV_
MetazoaVertebrateEP00066Takifugurubripes_5696740_
MetazoaVertebrateHomosapiens_6459549_ZACN_HUMAN_Zinc_activated_ligand_gated_ion_channel_OS_Homo_sapiens_OX_9606_GN_ZACN_PE_1_SV_
MetazoaVertebrateMonodelphisdomestica_6497698_ZACN_CANLF_Zinc_activated_ligand_gated_ion_channel_OS_Canis_lupus_familiaris_OX_9615_GN_ZACN_PE_3_SV_
MetazoaUrochordataCionaintestinalis_5346630_5HT3A_MOUSE_5_hydroxytryptamine_receptor_3A_OS_Mus_musculus_OX_10090_GN_Htr3a_PE_1_SV_
MetazoaUrochordataCionaintestinalis_5347767_5HT3A_MOUSE_5_hydroxytryptamine_receptor_3A_OS_Mus_musculus_OX_10090_GN_Htr3a_PE_1_SV_
MetazoaUrochordataCionaintestinalis_5341728_5HT3A_MOUSE_5_hydroxytryptamine_receptor_3A_OS_Mus_musculus_OX_10090_GN_Htr3a_PE_1_SV_
MetazoaAcoelaHofsteniamiamia_14172_ACH10_HUMAN_Neuronal_acetylcholine_receptor_subunit_alpha_10_OS_Homo_sapiens_OX_9606_GN_CHRNA10_PE_1_SV_
MetazoaAcoelaHofsteniamiamia_18060_ACHA5_CHICK_Neuronal_acetylcholine_receptor_subunit_alpha_5_OS_Gallus_gallus_OX_9031_GN_CHRNA5_PE_1_SV_
0.45

16F.

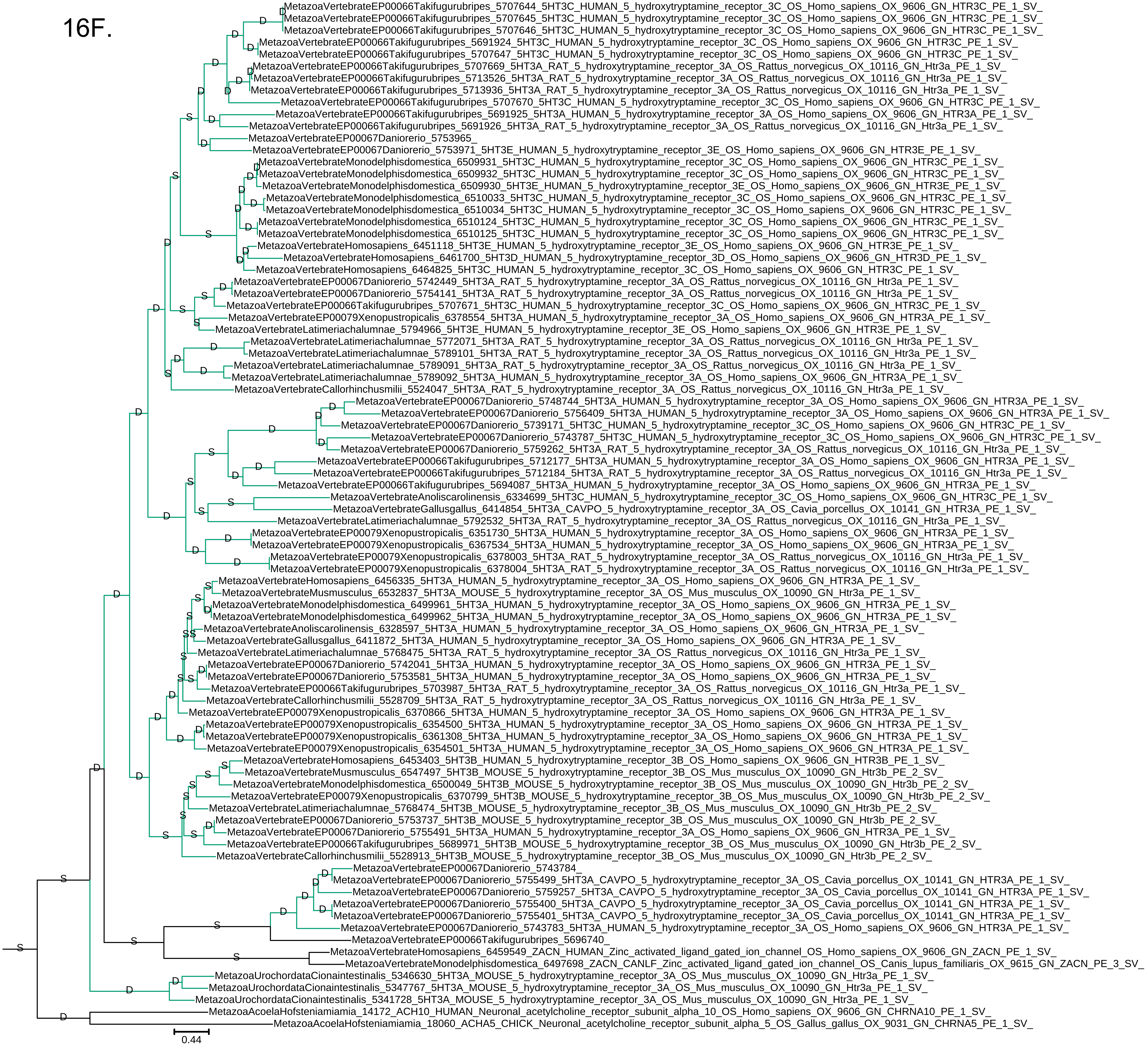
MetazoaVertebrateEP00066Takifugurubripes_5707644_5HT3C_HUMAN_5_hydroxytryptamine_receptor_3C_OS_Homo_sapiens_OX_9606_GN_HTR3C_PE_1_SV_
MetazoaVertebrateEP00066Takifugurubripes_5707645_5HT3C_HUMAN_5_hydroxytryptamine_receptor_3C_OS_Homo_sapiens_OX_9606_GN_HTR3C_PE_1_SV_
MetazoaVertebrateEP00066Takifugurubripes_5707646_5HT3C_HUMAN_5_hydroxytryptamine_receptor_3C_OS_Homo_sapiens_OX_9606_GN_HTR3C_PE_1_SV_
MetazoaVertebrateEP00066Takifugurubripes_5691924_5HT3C_HUMAN_5_hydroxytryptamine_receptor_3C_OS_Homo_sapiens_OX_9606_GN_HTR3C_PE_1_SV_
MetazoaVertebrateEP00066Takifugurubripes_5707647_5HT3C_HUMAN_5_hydroxytryptamine_receptor_3C_OS_Homo_sapiens_OX_9606_GN_HTR3C_PE_1_SV_
MetazoaVertebrateEP00066Takifugurubripes_5707669_5HT3A_RAT_5_hydroxytryptamine_receptor_3A_OS_Rattus_norvegicus_OX_10116_GN_Htr3a_PE_1_SV_
MetazoaVertebrateEP00066Takifugurubripes_5713526_5HT3A_RAT_5_hydroxytryptamine_receptor_3A_OS_Rattus_norvegicus_OX_10116_GN_Htr3a_PE_1_SV_
MetazoaVertebrateEP00066Takifugurubripes_5713936_5HT3A_RAT_5_hydroxytryptamine_receptor_3A_OS_Rattus_norvegicus_OX_10116_GN_Htr3a_PE_1_SV_
MetazoaVertebrateEP00066Takifugurubripes_5707670_5HT3C_HUMAN_5_hydroxytryptamine_receptor_3C_OS_Homo_sapiens_OX_9606_GN_HTR3C_PE_1_SV_
MetazoaVertebrateEP00066Takifugurubripes_5691925_5HT3A_HUMAN_5_hydroxytryptamine_receptor_3A_OS_Homo_sapiens_OX_9606_GN_HTR3A_PE_1_SV_
MetazoaVertebrateEP00066Takifugurubripes_5691926_5HT3A_RAT_5_hydroxytryptamine_receptor_3A_OS_Rattus_norvegicus_OX_10116_GN_Htr3a_PE_1_SV_
MetazoaVertebrateEP00067Daniorerio_5753965_
MetazoaVertebrateEP00067Daniorerio_5753971_5HT3E_HUMAN_5_hydroxytryptamine_receptor_3E_OS_Homo_sapiens_OX_9606_GN_HTR3E_PE_1_SV_
MetazoaVertebrateMonodelphisdomestica_6509931_5HT3C_HUMAN_5_hydroxytryptamine_receptor_3C_OS_Homo_sapiens_OX_9606_GN_HTR3C_PE_1_SV_
MetazoaVertebrateMonodelphisdomestica_6509932_5HT3C_HUMAN_5_hydroxytryptamine_receptor_3C_OS_Homo_sapiens_OX_9606_GN_HTR3C_PE_1_SV_
MetazoaVertebrateMonodelphisdomestica_6509930_5HT3E_HUMAN_5_hydroxytryptamine_receptor_3E_OS_Homo_sapiens_OX_9606_GN_HTR3E_PE_1_SV_
MetazoaVertebrateMonodelphisdomestica_6510033_5HT3C_HUMAN_5_hydroxytryptamine_receptor_3C_OS_Homo_sapiens_OX_9606_GN_HTR3C_PE_1_SV_
MetazoaVertebrateMonodelphisdomestica_6510034_5HT3C_HUMAN_5_hydroxytryptamine_receptor_3C_OS_Homo_sapiens_OX_9606_GN_HTR3C_PE_1_SV_
MetazoaVertebrateMonodelphisdomestica_6510124_5HT3C_HUMAN_5_hydroxytryptamine_receptor_3C_OS_Homo_sapiens_OX_9606_GN_HTR3C_PE_1_SV_
MetazoaVertebrateMonodelphisdomestica_6510125_5HT3C_HUMAN_5_hydroxytryptamine_receptor_3C_OS_Homo_sapiens_OX_9606_GN_HTR3C_PE_1_SV_
MetazoaVertebrateHomosapiens_6451118_5HT3E_HUMAN_5_hydroxytryptamine_receptor_3E_OS_Homo_sapiens_OX_9606_GN_HTR3E_PE_1_SV_
MetazoaVertebrateHomosapiens_6461700_5HT3D_HUMAN_5_hydroxytryptamine_receptor_3D_OS_Homo_sapiens_OX_9606_GN_HTR3D_PE_1_SV_
MetazoaVertebrateHomosapiens_6464825_5HT3C_HUMAN_5_hydroxytryptamine_receptor_3C_OS_Homo_sapiens_OX_9606_GN_HTR3C_PE_1_SV_
MetazoaVertebrateEP00067Daniorerio_5742449_5HT3A_RAT_5_hydroxytryptamine_receptor_3A_OS_Rattus_norvegicus_OX_10116_GN_Htr3a_PE_1_SV_
MetazoaVertebrateEP00067Daniorerio_5754141_5HT3A_RAT_5_hydroxytryptamine_receptor_3A_OS_Rattus_norvegicus_OX_10116_GN_Htr3a_PE_1_SV_
MetazoaVertebrateEP00066Takifugurubripes_5707671_5HT3C_HUMAN_5_hydroxytryptamine_receptor_3C_OS_Homo_sapiens_OX_9606_GN_HTR3C_PE_1_SV_
MetazoaVertebrateEP00079Xenopustropicalis_6378554_5HT3A_HUMAN_5_hydroxytryptamine_receptor_3A_OS_Homo_sapiens_OX_9606_GN_HTR3A_PE_1_SV_
MetazoaVertebrateLatimeriachalumnae_5794966_5HT3E_HUMAN_5_hydroxytryptamine_receptor_3E_OS_Homo_sapiens_OX_9606_GN_HTR3E_PE_1_SV_
MetazoaVertebrateLatimeriachalumnae_5772071_5HT3A_RAT_5_hydroxytryptamine_receptor_3A_OS_Rattus_norvegicus_OX_10116_GN_Htr3a_PE_1_SV_
MetazoaVertebrateLatimeriachalumnae_5789101_5HT3A_RAT_5_hydroxytryptamine_receptor_3A_OS_Rattus_norvegicus_OX_10116_GN_Htr3a_PE_1_SV_
MetazoaVertebrateLatimeriachalumnae_5789091_5HT3A_RAT_5_hydroxytryptamine_receptor_3A_OS_Rattus_norvegicus_OX_10116_GN_Htr3a_PE_1_SV_
MetazoaVertebrateLatimeriachalumnae_5789092_5HT3A_HUMAN_5_hydroxytryptamine_receptor_3A_OS_Homo_sapiens_OX_9606_GN_HTR3A_PE_1_SV_
MetazoaVertebrateCallorhinchusmilii_5524047_5HT3A_RAT_5_hydroxytryptamine_receptor_3A_OS_Rattus_norvegicus_OX_10116_GN_Htr3a_PE_1_SV_
MetazoaVertebrateEP00067Daniorerio_5748744_5HT3A_HUMAN_5_hydroxytryptamine_receptor_3A_OS_Homo_sapiens_OX_9606_GN_HTR3A_PE_1_SV_
MetazoaVertebrateEP00067Daniorerio_5756409_5HT3A_HUMAN_5_hydroxytryptamine_receptor_3A_OS_Homo_sapiens_OX_9606_GN_HTR3A_PE_1_SV_
MetazoaVertebrateEP00067Daniorerio_5739171_5HT3C_HUMAN_5_hydroxytryptamine_receptor_3C_OS_Homo_sapiens_OX_9606_GN_HTR3C_PE_1_SV_
MetazoaVertebrateEP00067Daniorerio_5743787_5HT3C_HUMAN_5_hydroxytryptamine_receptor_3C_OS_Homo_sapiens_OX_9606_GN_HTR3C_PE_1_SV_
MetazoaVertebrateEP00067Daniorerio_5759262_5HT3A_RAT_5_hydroxytryptamine_receptor_3A_OS_Rattus_norvegicus_OX_10116_GN_Htr3a_PE_1_SV_
MetazoaVertebrateEP00066Takifugurubripes_5712177_5HT3A_HUMAN_5_hydroxytryptamine_receptor_3A_OS_Homo_sapiens_OX_9606_GN_HTR3A_PE_1_SV_
MetazoaVertebrateEP00066Takifugurubripes_5712184_5HT3A_RAT_5_hydroxytryptamine_receptor_3A_OS_Rattus_norvegicus_OX_10116_GN_Htr3a_PE_1_SV_
MetazoaVertebrateEP00066Takifugurubripes_5694087_5HT3A_HUMAN_5_hydroxytryptamine_receptor_3A_OS_Homo_sapiens_OX_9606_GN_HTR3A_PE_1_SV_
MetazoaVertebrateAnoliscarolinensis_6334699_5HT3C_HUMAN_5_hydroxytryptamine_receptor_3C_OS_Homo_sapiens_OX_9606_GN_HTR3C_PE_1_SV_
MetazoaVertebrateGallusgallus_6414854_5HT3A_CAVPO_5_hydroxytryptamine_receptor_3A_OS_Cavia_porcellus_OX_10141_GN_HTR3A_PE_1_SV_
MetazoaVertebrateLatimeriachalumnae_5792532_5HT3A_RAT_5_hydroxytryptamine_receptor_3A_OS_Rattus_norvegicus_OX_10116_GN_Htr3a_PE_1_SV_
MetazoaVertebrateEP00079Xenopustropicalis_6351730_5HT3A_HUMAN_5_hydroxytryptamine_receptor_3A_OS_Homo_sapiens_OX_9606_GN_HTR3A_PE_1_SV_
MetazoaVertebrateEP00079Xenopustropicalis_6367534_5HT3A_HUMAN_5_hydroxytryptamine_receptor_3A_OS_Homo_sapiens_OX_9606_GN_HTR3A_PE_1_SV_
MetazoaVertebrateEP00079Xenopustropicalis_6378003_5HT3A_RAT_5_hydroxytryptamine_receptor_3A_OS_Rattus_norvegicus_OX_10116_GN_Htr3a_PE_1_SV_
MetazoaVertebrateEP00079Xenopustropicalis_6378004_5HT3A_RAT_5_hydroxytryptamine_receptor_3A_OS_Rattus_norvegicus_OX_10116_GN_Htr3a_PE_1_SV_
MetazoaVertebrateHomosapiens_6456335_5HT3A_HUMAN_5_hydroxytryptamine_receptor_3A_OS_Homo_sapiens_OX_9606_GN_HTR3A_PE_1_SV_
MetazoaVertebrateMusmusculus_6532837_5HT3A_MOUSE_5_hydroxytryptamine_receptor_3A_OS_Mus_musculus_OX_10090_GN_Htr3a_PE_1_SV_
MetazoaVertebrateMonodelphisdomestica_6499961_5HT3A_HUMAN_5_hydroxytryptamine_receptor_3A_OS_Homo_sapiens_OX_9606_GN_HTR3A_PE_1_SV_
MetazoaVertebrateMonodelphisdomestica_6499962_5HT3A_HUMAN_5_hydroxytryptamine_receptor_3A_OS_Homo_sapiens_OX_9606_GN_HTR3A_PE_1_SV_
MetazoaVertebrateAnoliscarolinensis_6328597_5HT3A_HUMAN_5_hydroxytryptamine_receptor_3A_OS_Homo_sapiens_OX_9606_GN_HTR3A_PE_1_SV_
MetazoaVertebrateGallusgallus_6411872_5HT3A_HUMAN_5_hydroxytryptamine_receptor_3A_OS_Homo_sapiens_OX_9606_GN_HTR3A_PE_1_SV_
MetazoaVertebrateLatimeriachalumnae_5768475_5HT3A_RAT_5_hydroxytryptamine_receptor_3A_OS_Rattus_norvegicus_OX_10116_GN_Htr3a_PE_1_SV_
MetazoaVertebrateEP00067Daniorerio_5742041_5HT3A_HUMAN_5_hydroxytryptamine_receptor_3A_OS_Homo_sapiens_OX_9606_GN_HTR3A_PE_1_SV_
MetazoaVertebrateEP00067Daniorerio_5753581_5HT3A_HUMAN_5_hydroxytryptamine_receptor_3A_OS_Homo_sapiens_OX_9606_GN_HTR3A_PE_1_SV_
MetazoaVertebrateEP00066Takifugurubripes_5703987_5HT3A_RAT_5_hydroxytryptamine_receptor_3A_OS_Rattus_norvegicus_OX_10116_GN_Htr3a_PE_1_SV_
MetazoaVertebrateCallorhinchusmilii_5528709_5HT3A_RAT_5_hydroxytryptamine_receptor_3A_OS_Rattus_norvegicus_OX_10116_GN_Htr3a_PE_1_SV_
MetazoaVertebrateEP00079Xenopustropicalis_6370866_5HT3A_HUMAN_5_hydroxytryptamine_receptor_3A_OS_Homo_sapiens_OX_9606_GN_HTR3A_PE_1_SV_
MetazoaVertebrateEP00079Xenopustropicalis_6354500_5HT3A_HUMAN_5_hydroxytryptamine_receptor_3A_OS_Homo_sapiens_OX_9606_GN_HTR3A_PE_1_SV_
MetazoaVertebrateEP00079Xenopustropicalis_6361308_5HT3A_HUMAN_5_hydroxytryptamine_receptor_3A_OS_Homo_sapiens_OX_9606_GN_HTR3A_PE_1_SV_
MetazoaVertebrateEP00079Xenopustropicalis_6354501_5HT3A_HUMAN_5_hydroxytryptamine_receptor_3A_OS_Homo_sapiens_OX_9606_GN_HTR3A_PE_1_SV_
MetazoaVertebrateHomosapiens_6453403_5HT3B_HUMAN_5_hydroxytryptamine_receptor_3B_OS_Homo_sapiens_OX_9606_GN_HTR3B_PE_1_SV_
MetazoaVertebrateMusmusculus_6547497_5HT3B_MOUSE_5_hydroxytryptamine_receptor_3B_OS_Mus_musculus_OX_10090_GN_Htr3b_PE_2_SV_
MetazoaVertebrateMonodelphisdomestica_6500049_5HT3B_MOUSE_5_hydroxytryptamine_receptor_3B_OS_Mus_musculus_OX_10090_GN_Htr3b_PE_2_SV_
MetazoaVertebrateEP00079Xenopustropicalis_6370799_5HT3B_MOUSE_5_hydroxytryptamine_receptor_3B_OS_Mus_musculus_OX_10090_GN_Htr3b_PE_2_SV_
MetazoaVertebrateLatimeriachalumnae_5768474_5HT3B_MOUSE_5_hydroxytryptamine_receptor_3B_OS_Mus_musculus_OX_10090_GN_Htr3b_PE_2_SV_
MetazoaVertebrateEP00067Daniorerio_5753737_5HT3B_MOUSE_5_hydroxytryptamine_receptor_3B_OS_Mus_musculus_OX_10090_GN_Htr3b_PE_2_SV_
MetazoaVertebrateEP00067Daniorerio_5755491_5HT3A_HUMAN_5_hydroxytryptamine_receptor_3A_OS_Homo_sapiens_OX_9606_GN_HTR3A_PE_1_SV_
MetazoaVertebrateEP00066Takifugurubripes_5689971_5HT3B_MOUSE_5_hydroxytryptamine_receptor_3B_OS_Mus_musculus_OX_10090_GN_Htr3b_PE_2_SV_
MetazoaVertebrateCallorhinchusmilii_5528913_5HT3B_MOUSE_5_hydroxytryptamine_receptor_3B_OS_Mus_musculus_OX_10090_GN_Htr3b_PE_2_SV_
MetazoaVertebrateEP00067Daniorerio_5743784_
MetazoaVertebrateEP00067Daniorerio_5755499_5HT3A_CAVPO_5_hydroxytryptamine_receptor_3A_OS_Cavia_porcellus_OX_10141_GN_HTR3A_PE_1_SV_
MetazoaVertebrateEP00067Daniorerio_5759257_5HT3A_CAVPO_5_hydroxytryptamine_receptor_3A_OS_Cavia_porcellus_OX_10141_GN_HTR3A_PE_1_SV_
MetazoaVertebrateEP00067Daniorerio_5755400_5HT3A_CAVPO_5_hydroxytryptamine_receptor_3A_OS_Cavia_porcellus_OX_10141_GN_HTR3A_PE_1_SV_
MetazoaVertebrateEP00067Daniorerio_5755401_5HT3A_CAVPO_5_hydroxytryptamine_receptor_3A_OS_Cavia_porcellus_OX_10141_GN_HTR3A_PE_1_SV_
MetazoaVertebrateEP00067Daniorerio_5743783_5HT3A_HUMAN_5_hydroxytryptamine_receptor_3A_OS_Homo_sapiens_OX_9606_GN_HTR3A_PE_1_SV_
MetazoaVertebrateEP00066Takifugurubripes_5696740_
MetazoaVertebrateHomosapiens_6459549_ZACN_HUMAN_Zinc_activated_ligand_gated_ion_channel_OS_Homo_sapiens_OX_9606_GN_ZACN_PE_1_SV_
MetazoaVertebrateMonodelphisdomestica_6497698_ZACN_CANLF_Zinc_activated_ligand_gated_ion_channel_OS_Canis_lupus_familiaris_OX_9615_GN_ZACN_PE_3_SV_
MetazoaUrochordataCionaintestinalis_5346630_5HT3A_MOUSE_5_hydroxytryptamine_receptor_3A_OS_Mus_musculus_OX_10090_GN_Htr3a_PE_1_SV_
MetazoaUrochordataCionaintestinalis_5347767_5HT3A_MOUSE_5_hydroxytryptamine_receptor_3A_OS_Mus_musculus_OX_10090_GN_Htr3a_PE_1_SV_
MetazoaUrochordataCionaintestinalis_5341728_5HT3A_MOUSE_5_hydroxytryptamine_receptor_3A_OS_Mus_musculus_OX_10090_GN_Htr3a_PE_1_SV_
MetazoaAcoelaHofsteniamiamia_14172_ACH10_HUMAN_Neuronal_acetylcholine_receptor_subunit_alpha_10_OS_Homo_sapiens_OX_9606_GN_CHRNA10_PE_1_SV_
MetazoaAcoelaHofsteniamiamia_18060_ACHA5_CHICK_Neuronal_acetylcholine_receptor_subunit_alpha_5_OS_Gallus_gallus_OX_9031_GN_CHRNA5_PE_1_SV_
0.44

Supplementary Figure 17: Analysis of OG1. A) TBE tree with TBE score supports. B) Maximum likelihood tree with ultrafast bootstrap proportion supports. C) Maximum likelihood tree including all eukaryotic sequences with ultrafast bootstrap proportion supports. D) Reconciliation using sponge-first hypothesis. E) Reconciliation using ctenophore-first hypothesis. F) Reconciliation of the UFB tree including all eukaryotic sequences. Nodes in reconciled trees indicated as speciation events (S) or duplication events (D). All trees shown as rooted, non-reconciled trees rooted arbitrarily for visualisation. OG = orthogroup, TBE = transfer bootstrap expectation, UFB = ultrafast bootstrap. Branches colour coded by clade; purple = bilaterian specific clade of monoamine receptors; dark orange = cnidarian specific clade; and black = others. Source Data are available at [https://figshare.le.ac.uk/articles/dataset/Monoamine_Neuromodulation_is_a_Bilaterian_Innovation_Results/20391477]; in Results/GeneTrees labelled OG_1_TBE.tbe.tree and OG_1_UFB.treefile; in Results/GeneTrees/Eukaryotes_GeneTrees labelled OG_1_EUKIQ.treefile; in Results/Reconciliation/SpongeFirst labelled OG_1; in Results/Reconciliation/CtenophoreFirst labelled OG_1; and in Results/Reconciliation/Eukaryote labelled OG_1.

17A.

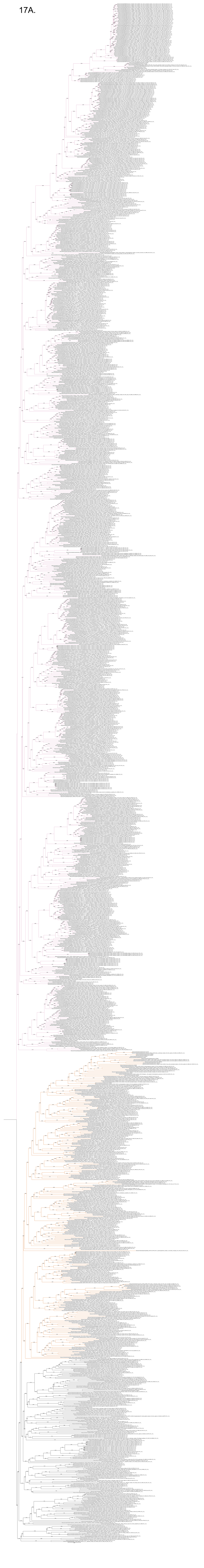

17B.

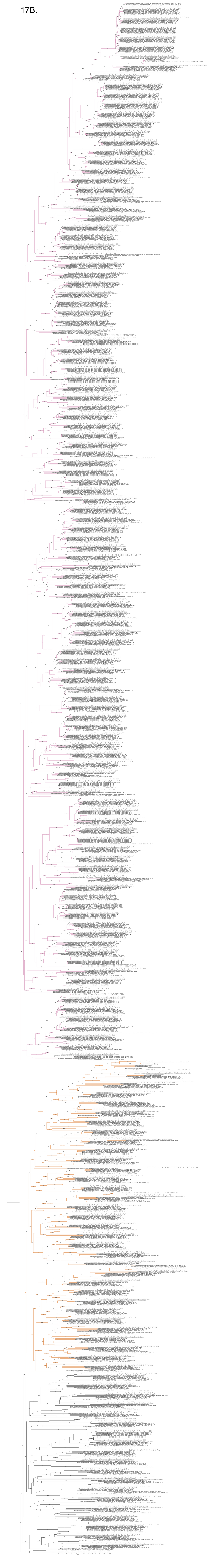

17C.

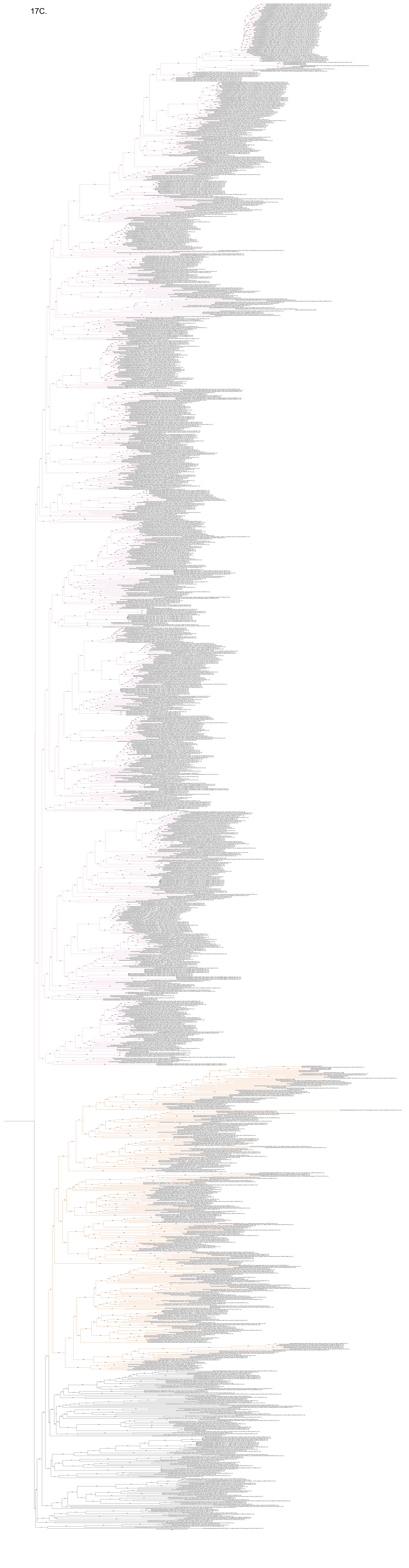

17D.

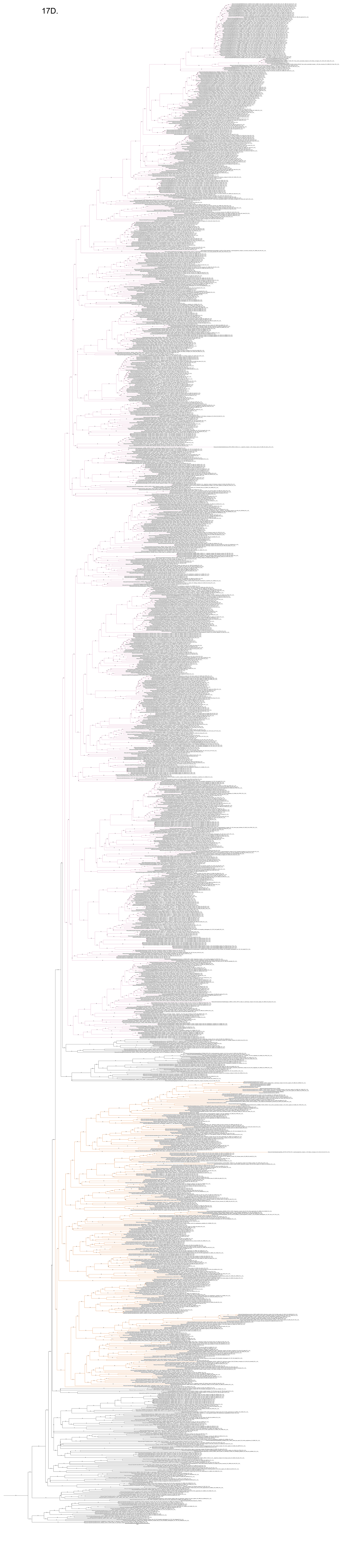

17E.

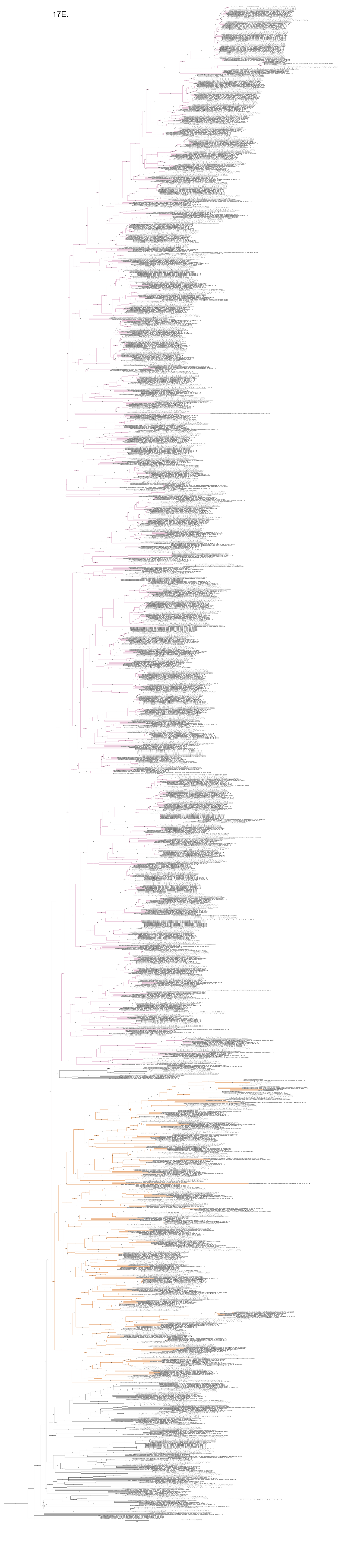

17F.

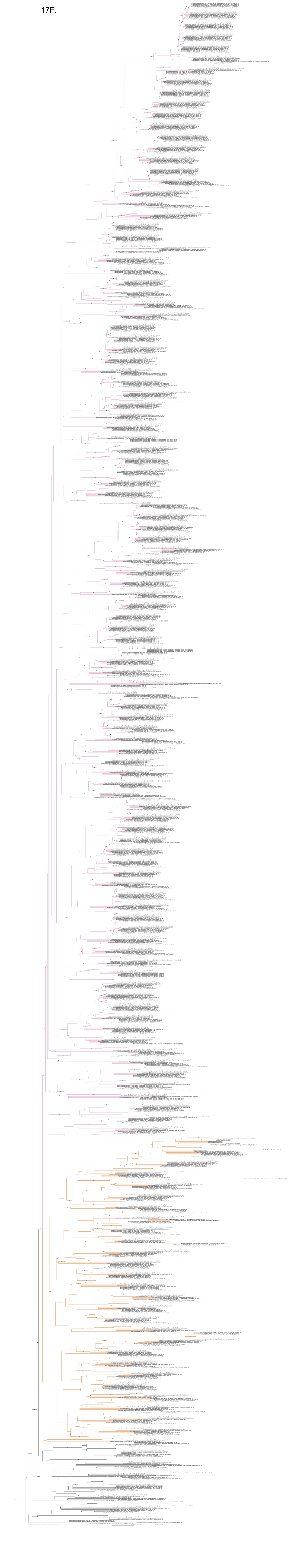

Supplementary Figure 18: Analysis of OG30. A) TBE tree with TBE score supports. B) Maximum likelihood tree with ultrafast bootstrap proportion supports. C) Maximum likelihood tree including all eukaryotic sequences with ultrafast bootstrap proportion supports. D) Reconciliation using sponge-first hypothesis. E) Reconciliation using ctenophore-first hypothesis. F) Reconciliation of the UFB tree including all eukaryotic sequences. Nodes in reconciled trees indicated as speciation events (S) or duplication events (D). All trees shown as rooted, non-reconciled trees rooted arbitrarily for visualisation. OG = orthogroup, TBE = transfer bootstrap expectation, UFB = ultrafast bootstrap. Branches colour coded by; green = clade of histamine and acetylcholine muscarinic receptors; and black = others. Source Data are available at [https://figshare.le.ac.uk/articles/dataset/Monoamine_Neuromodulation_is_a_Bilaterian_Innovation_Results/20391477]; in Results/GeneTrees labelled OG_30_TBE.tbe.tree and OG_30_UFB.treefile; in Results/GeneTrees/Eukaryotes_GeneTrees labelled OG_30_EUKIQ.treefile; in Results/Reconciliation/SpongeFirst labelled OG_30; in Results/Reconciliation/CtenophoreFirst labelled OG_30; and in Results/Reconciliation/Eukaryote labelled OG_30.

18A.

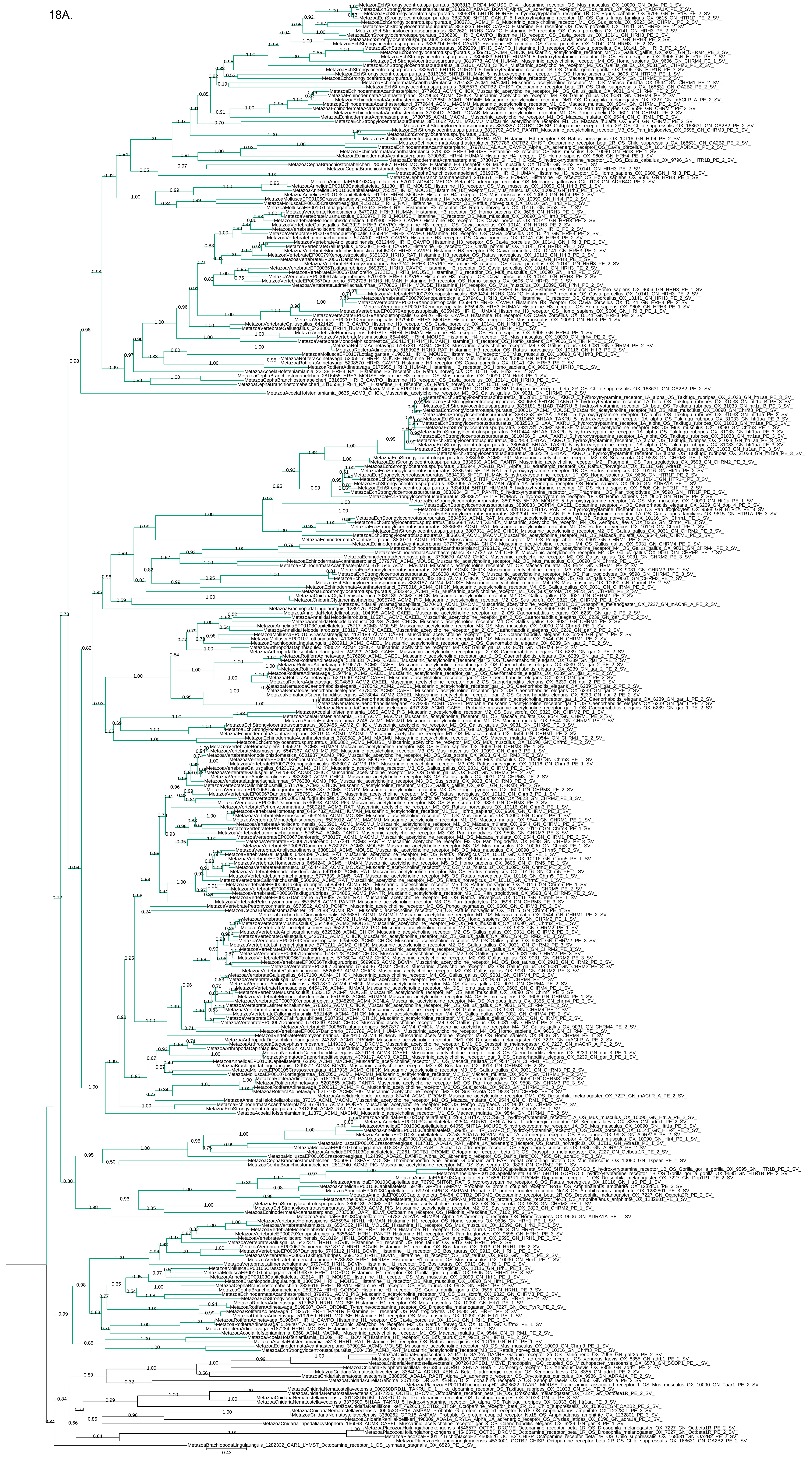

18B.

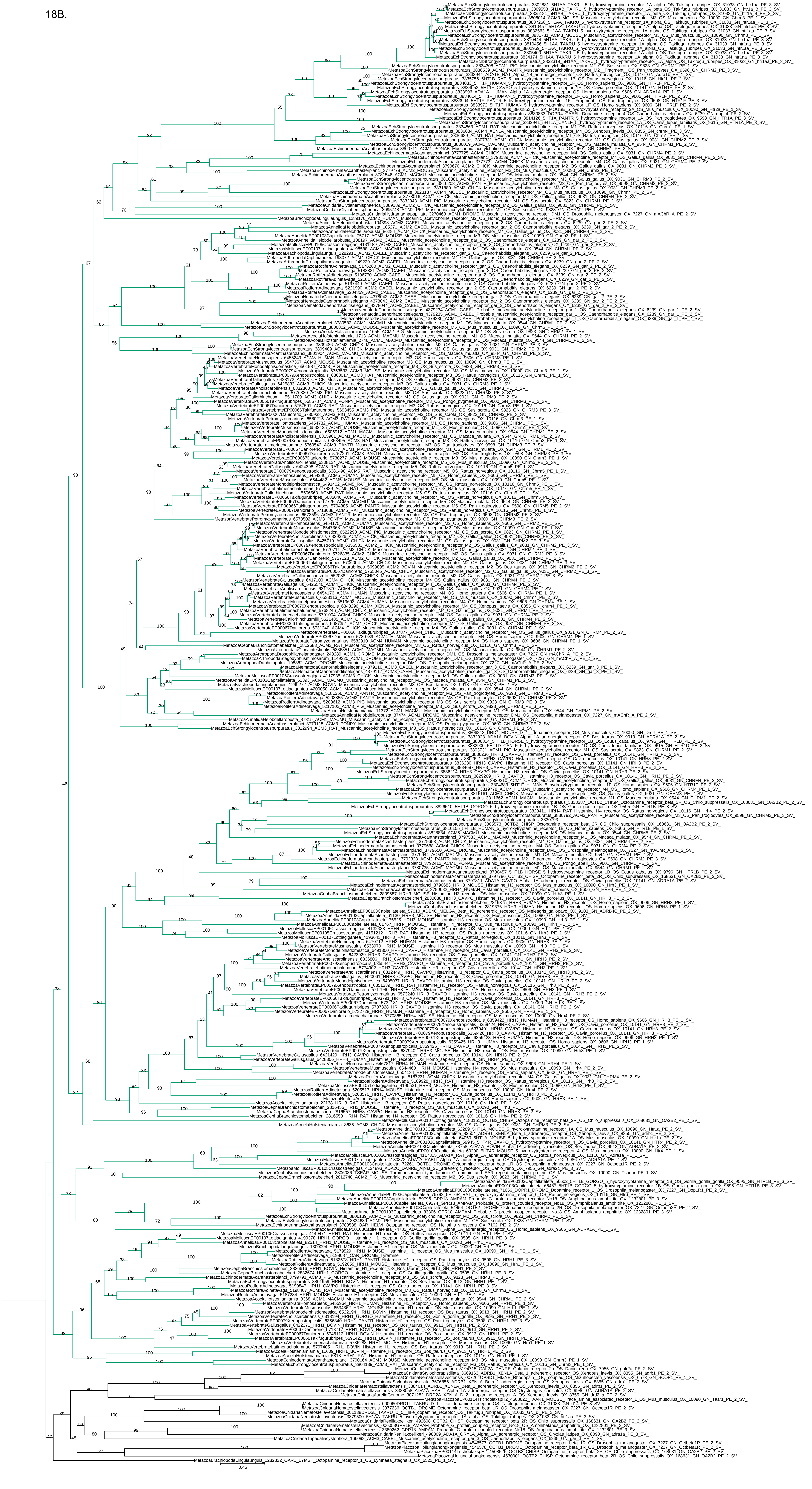

18C.

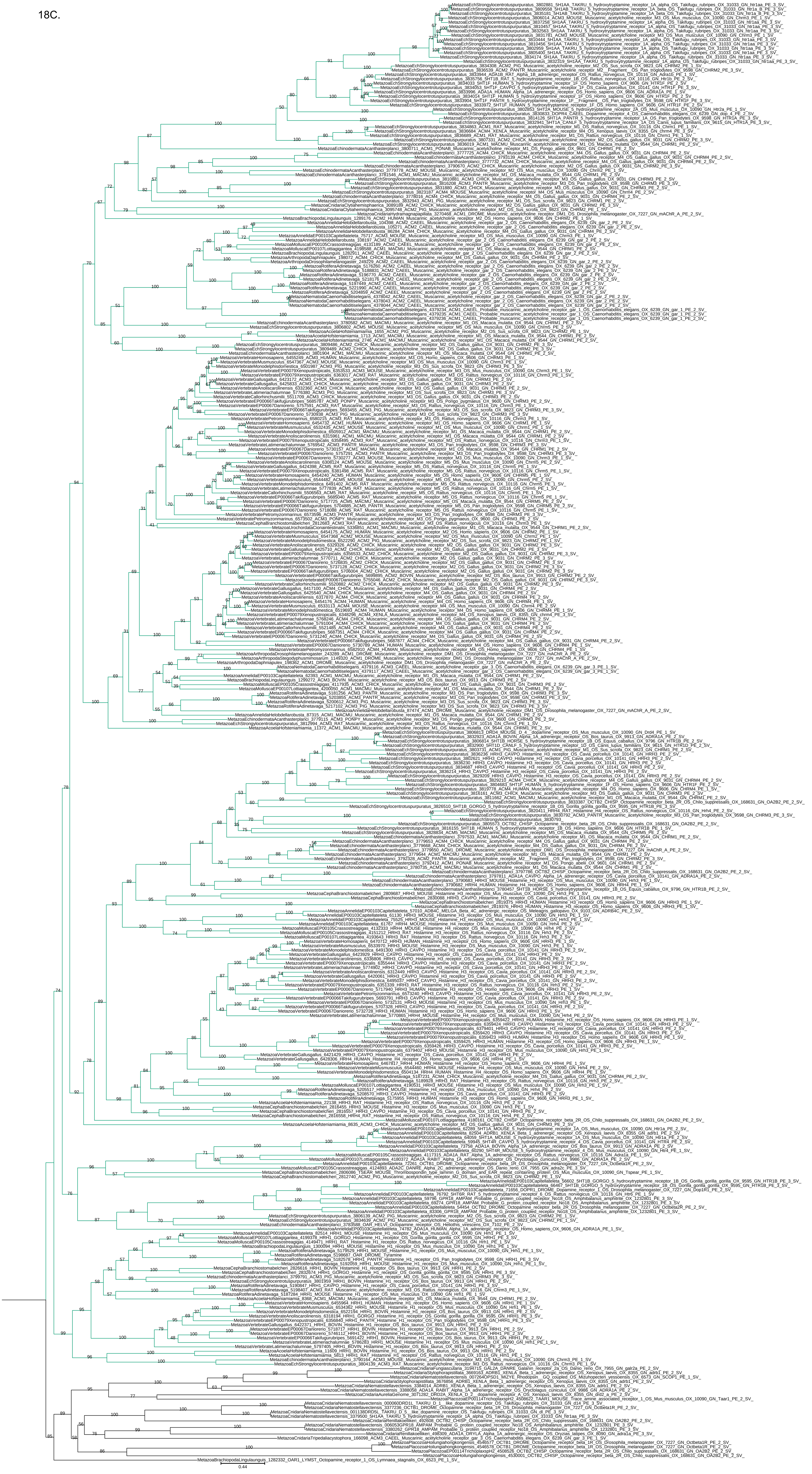

18D.

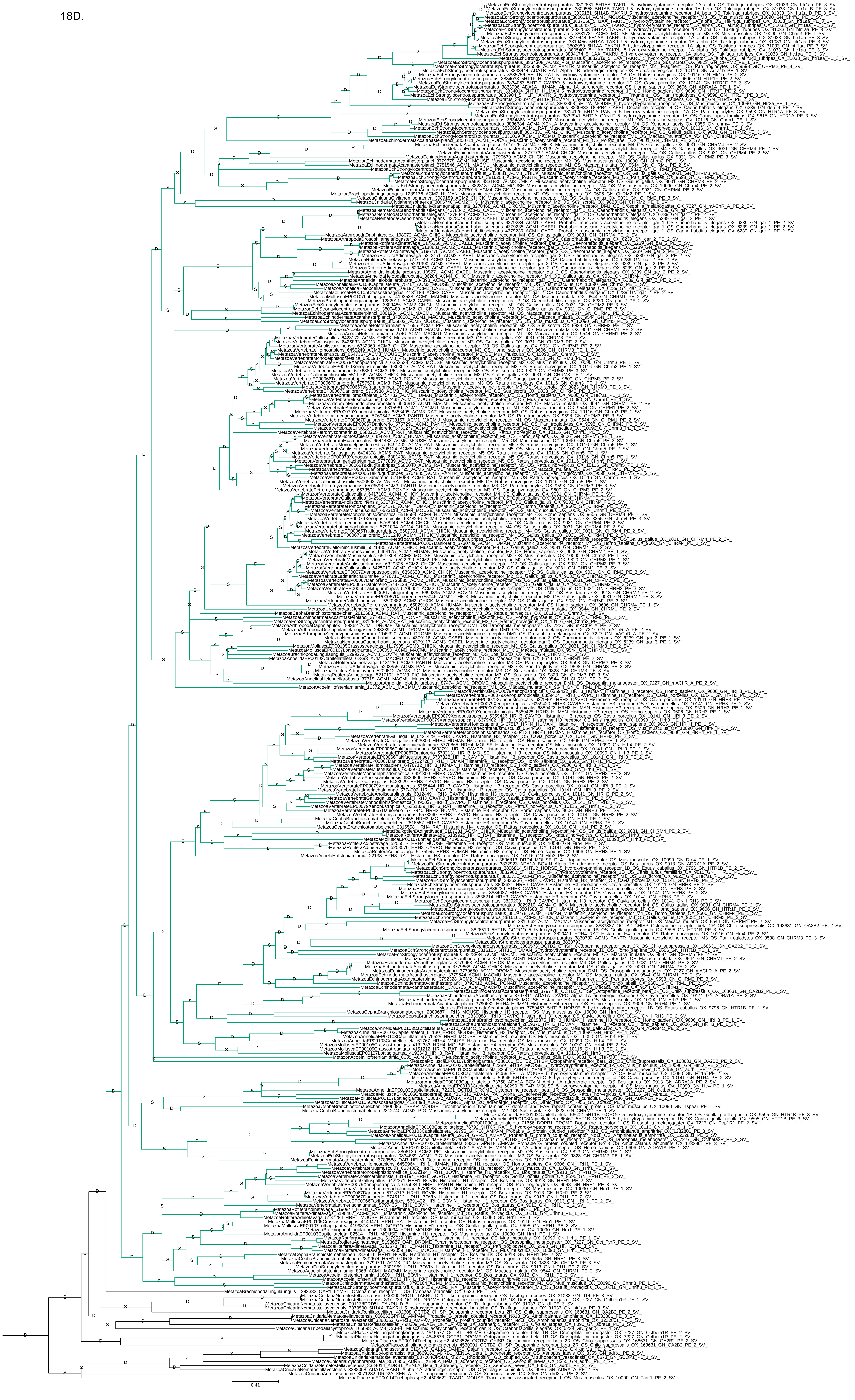

18E.

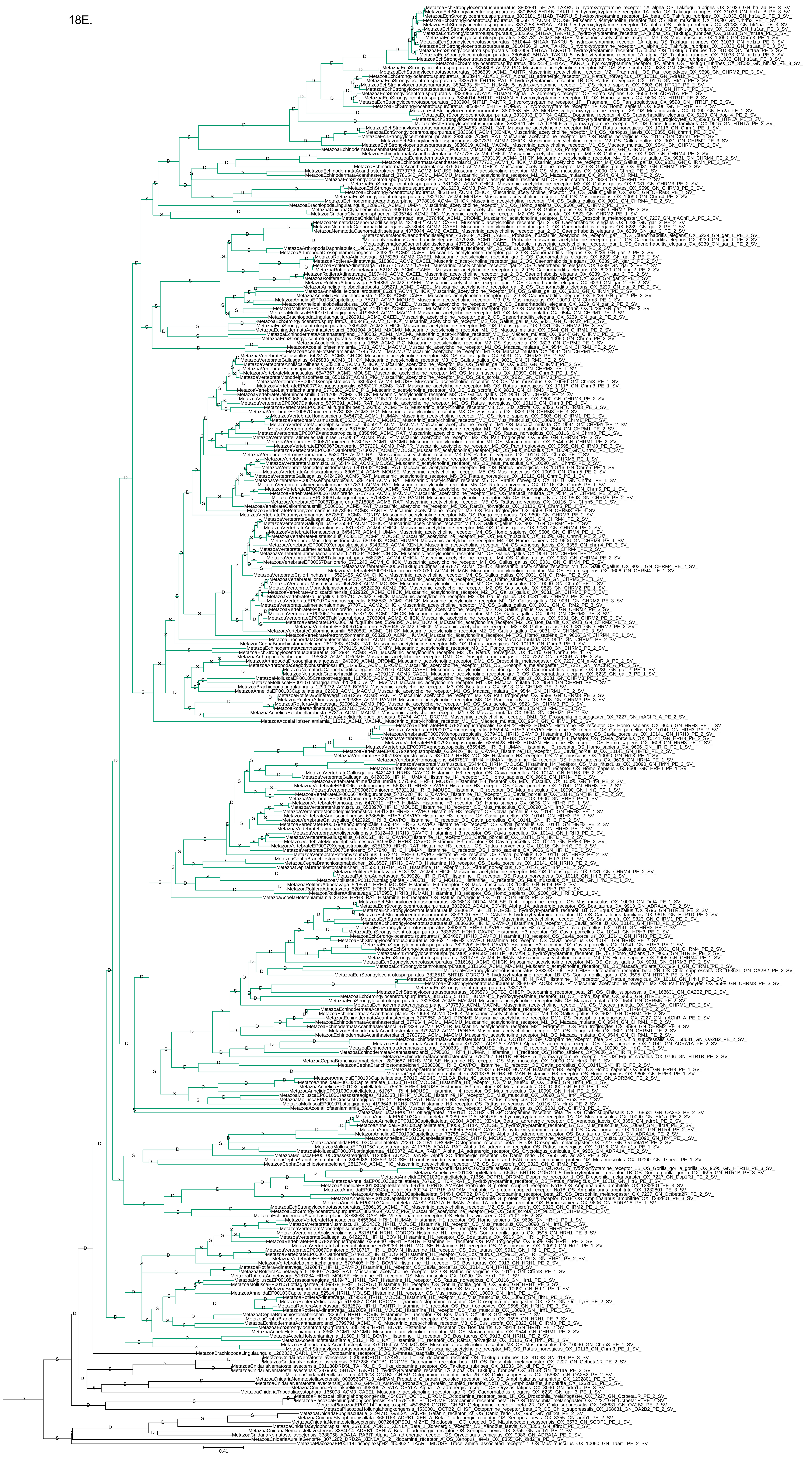

18F.

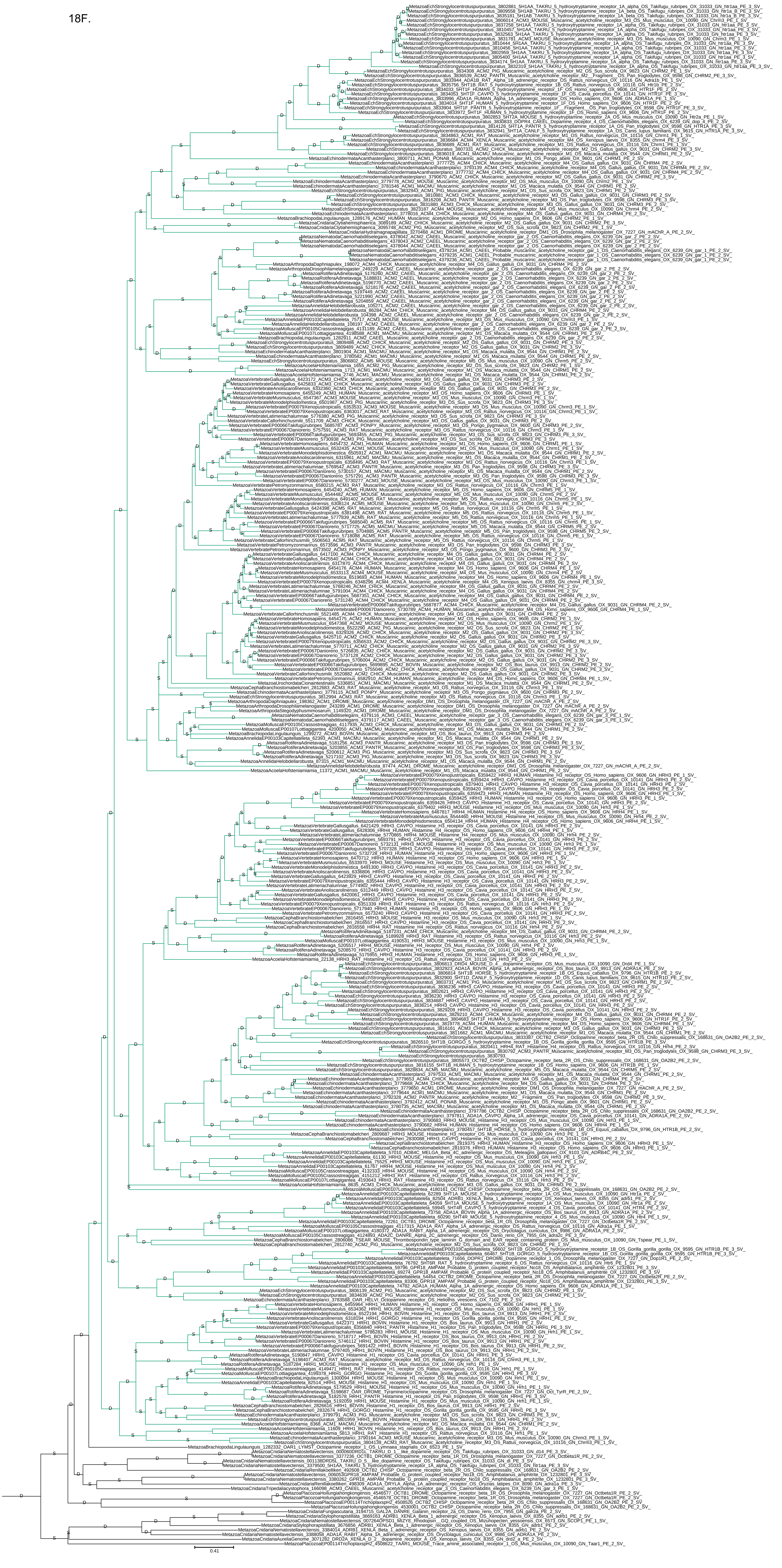

Supplementary Figure 19: CLANS analysis of GPCRs. A) Clusters obtained using a p-value < 1e-100. B) Clusters obtained using a p-value < 1e-42. C) Clusters obtained using a p-value < 1e-42 and the connection obtained with a p-value < 1e-40. Each dot represents a sequence, lines indicate similarity between two sequences at or above the p-value threshold. Key clusters were coloured according to sequences from *Homo sapiens*, *Mus musculus* and *Drosophila melanogaster*. Purple and green indicate bilaterian sequences from OG1 and OG30 respectively and red triangles indicate other sequences included in broccoli OG1 and OG30. Other dots colour coded by sequence annotations: dark blue = adenosine receptors; light blue = melatonin receptors; orange = opsins; and yellow = other GPCR groups. GPCR = g-protein coupled receptor, OG = orthogroup.

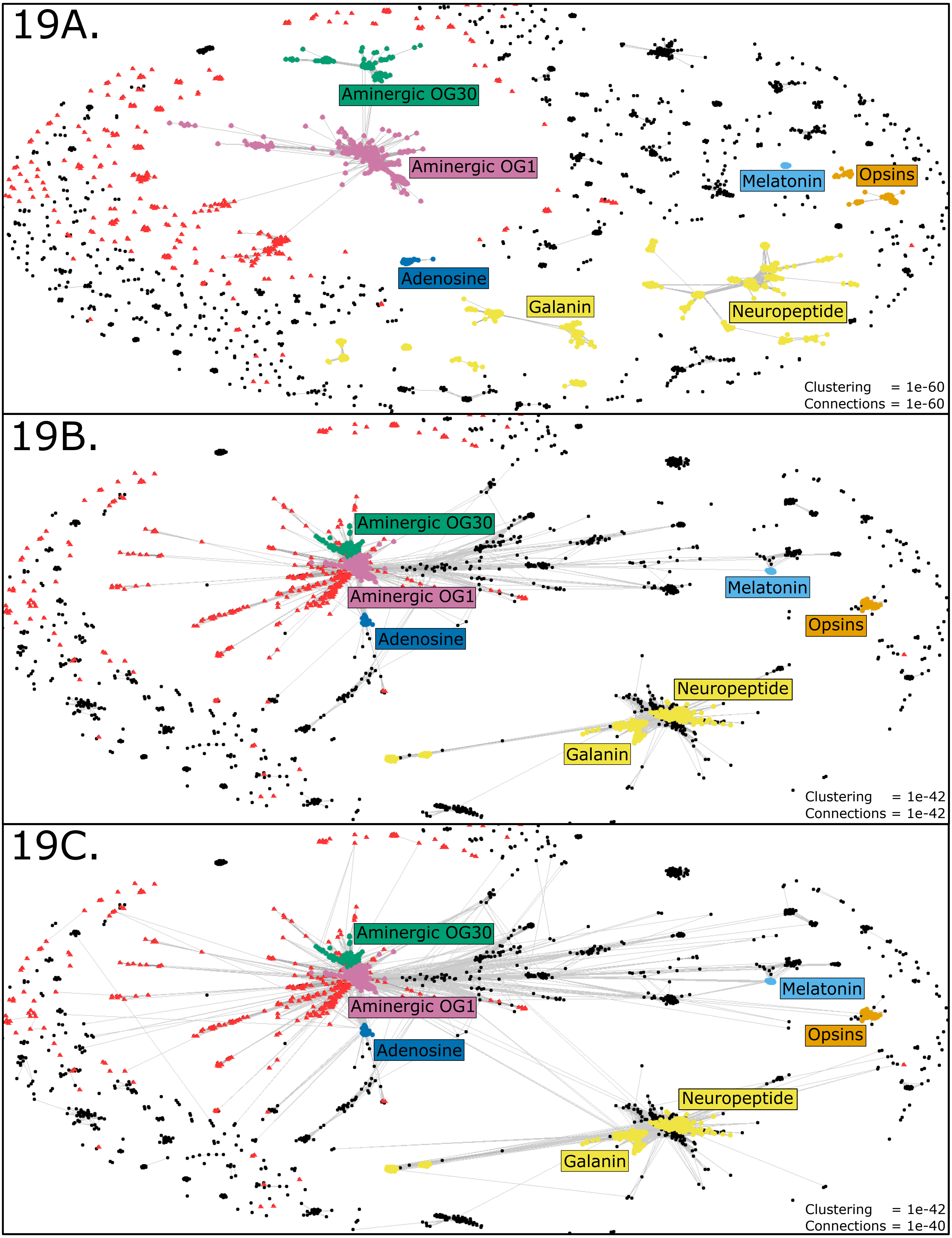
19A.
Aminergic OG30
Aminergic OG1
Melatonin
Opsins
Adenosine
Galanin
Neuropeptide
Clustering = 1e-60
Connections = 1e-60
19B.
Aminergic OG30
Aminergic OG1
Melatonin
Opsins
Adenosine
Neuropeptide
Galanin
Clustering = 1e-42
Connections = 1e-42
19C.
Aminergic OG30
Aminergic OG1
Melatonin
Opsins
Adenosine
Neuropeptide
Galanin
Clustering = 1e-42
Connections = 1e-40

Supplementary Figure 20: Analysis of GPCRs. A) TBE tree with TBE score supports. B) Maximum likelihood tree with ultrafast bootstrap proportion supports. C) TBE tree reconciliation using sponge-first hypothesis. D) UFB tree reconciliation using sponge-first hypothesis. E) TBE tree reconciliation using ctenophore-first hypothesis. F) UFB tree reconciliation using ctenophore-first hypothesis. Nodes in reconciled trees indicated as speciation events (S) or duplication events (D). All trees shown as rooted, non-reconciled trees rooted arbitrarily for visualisation. GPCR = g-protein coupled receptor, TBE = transfer bootstrap expectation, UFB = ultrafast bootstrap, OG = orthogroup. Branches colour coded by sequence annotation or clade; purple = bilaterian specific OG1 clade; green = OG30 clade; dark orange = cnidarian specific clade; dark blue = adenosine receptors; light blue = melatonin receptors; orange = opsins; and black = others. Source Data are available at [https://figshare.le.ac.uk/articles/dataset/Monoamine_Neuromodulation_is_a_Bilaterian_Innovation_Results/20391477]; in Results/GPCRAnalysis/GeneTrees labelled GPCR_CLANs_TBE.tbe.tree and GPCR_CLANs_UFB.treefile; in Results/Reconciliation/SpongeFirst labelled GPCR_CLANs_TBE and GPCR_CLANs_UFB; and in Results/Reconciliation/CtenophoreFirst labelled GPCR_CLANs_TBE and GPCR_CLANs_UFB.

20A.

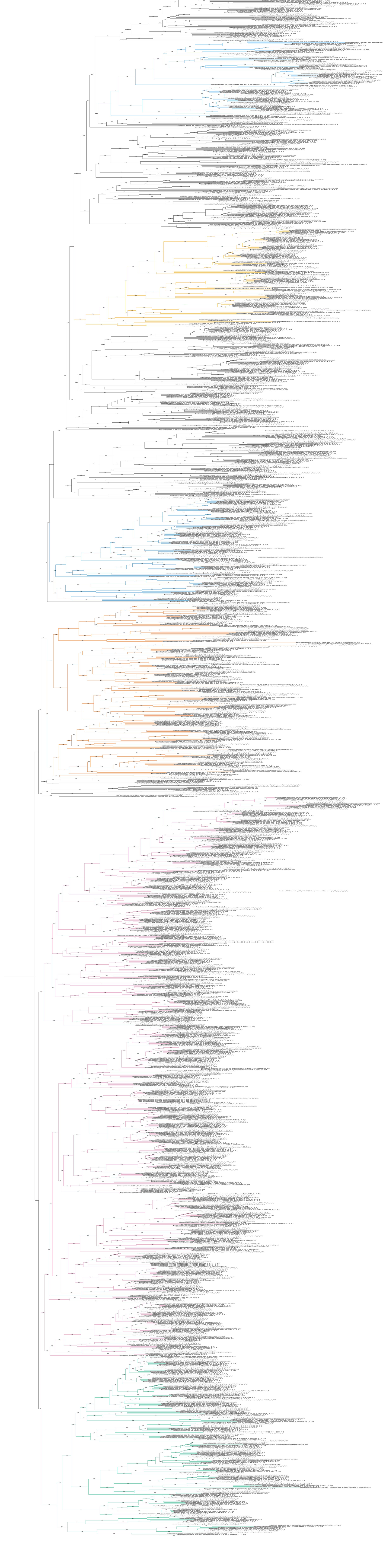

20B.

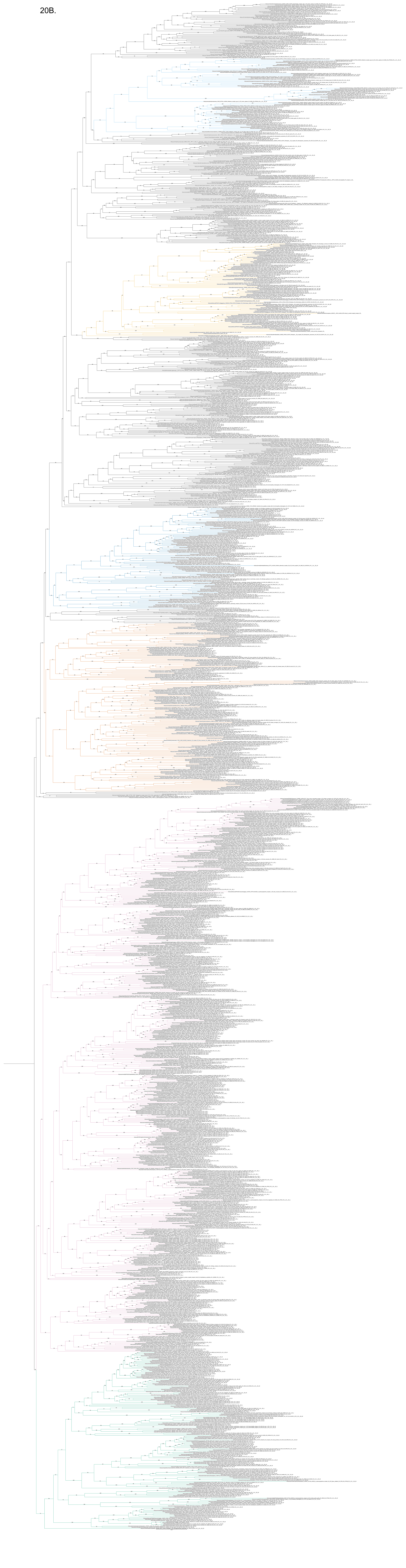

20C.

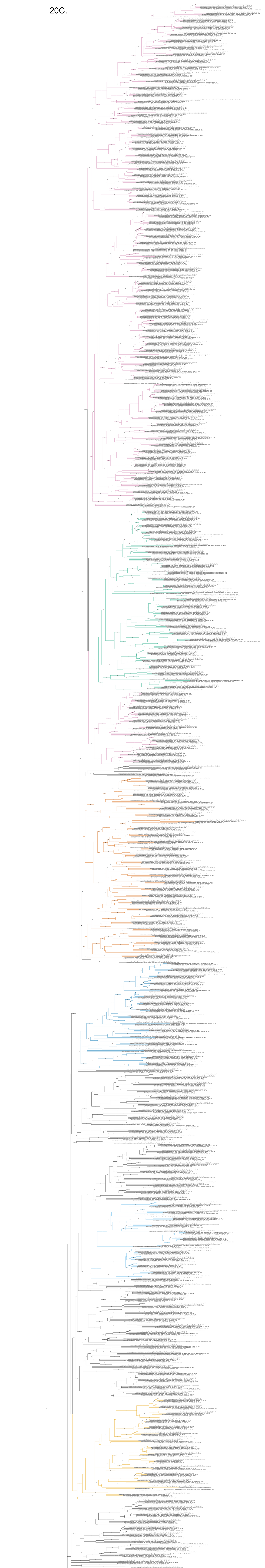

20D.

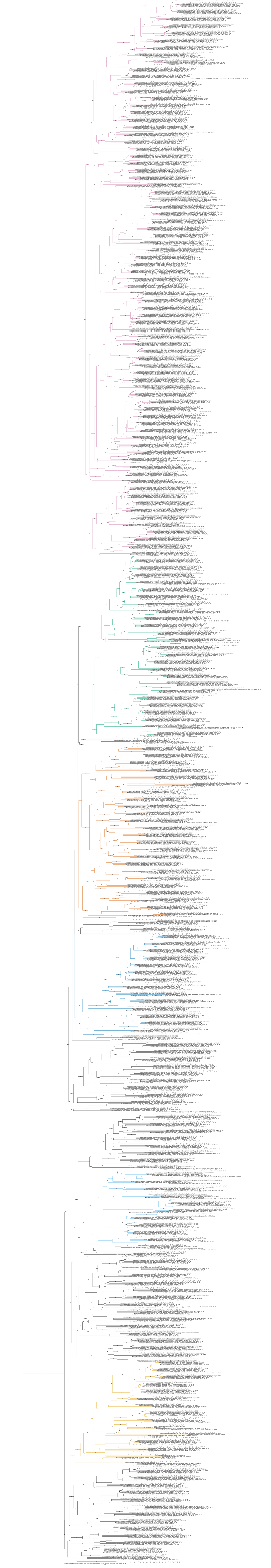

20E.

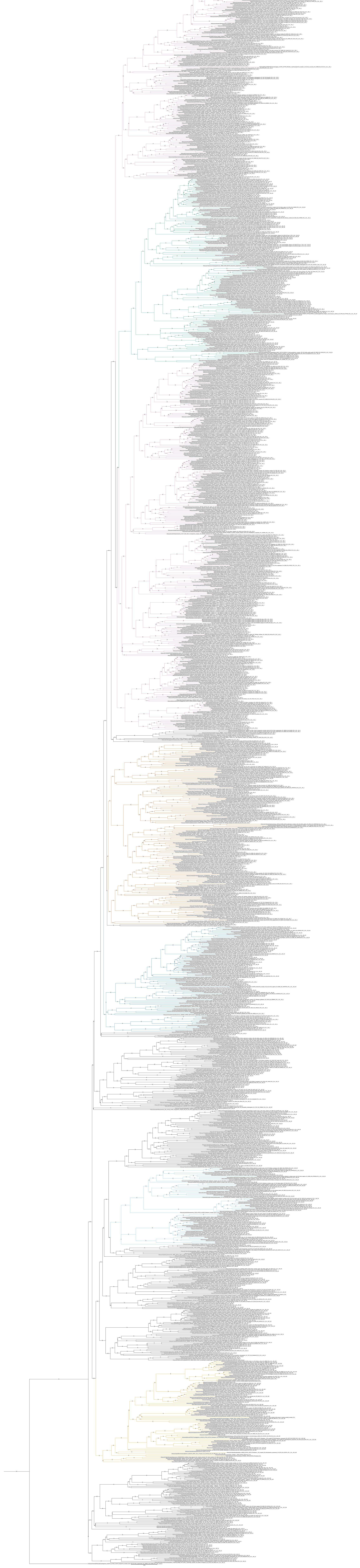

20F.

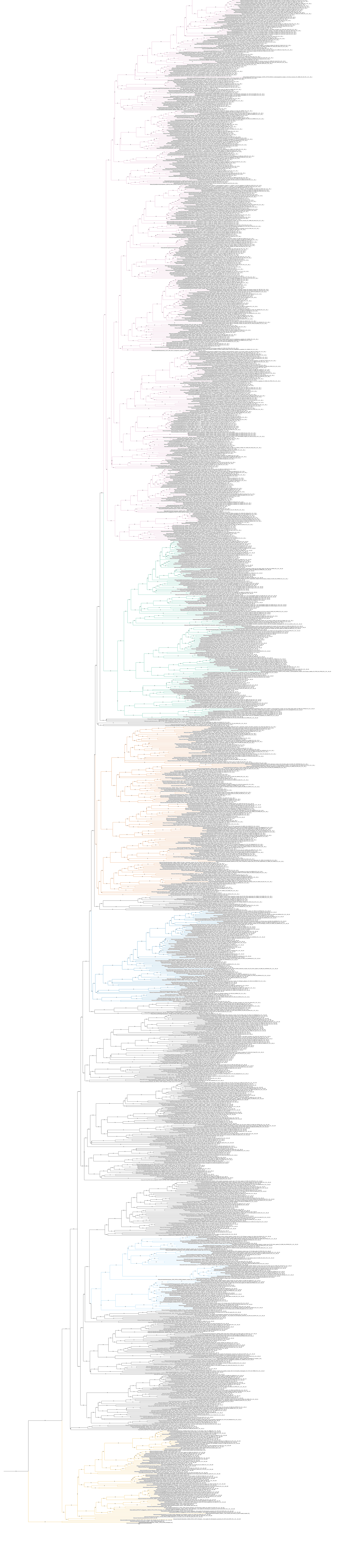

Supplement: Supplementary file 1 — Supplementary information [file 41467_2023_39030_MOESM1_ESM.pdf]
