# Supplementary material for: The monoaminergic system is a bilaterian innovation
Source: Nat Commun. 2023 Jun 6;14:3284. doi: 10.1038/s41467-023-39030-2 (PMC10244343; doi:10.1038/s41467-023-39030-2)
Supplement: Supplementary file 4 — Reporting Summary [file 41467_2023_39030_MOESM4_ESM.pdf]

nature portfolio

Corresponding author(s): Roberto Feuda

Last updated by author(s): May 5, 2023

Reporting Summary

Nature Portfolio wishes to improve the reproducibility of the work that we publish. This form provides structure for consistency and transparency in reporting. For further information on Nature Portfolio policies, see our Editorial Policies and the Editorial Policy Checklist.

Statistics

For all statistical analyses, confirm that the following items are present in the figure legend, table legend, main text, or Methods section.

n/a	Confirmed	
☒	☐	The exact sample size (*n*) for each experimental group/condition, given as a discrete number and unit of measurement
☒	☐	A statement on whether measurements were taken from distinct samples or whether the same sample was measured repeatedly
☒	☐	The statistical test(s) used AND whether they are one- or two-sided<br>*Only common tests should be described solely by name; describe more complex techniques in the Methods section.*
☒	☐	A description of all covariates tested
☒	☐	A description of any assumptions or corrections, such as tests of normality and adjustment for multiple comparisons
☒	☐	A full description of the statistical parameters including central tendency (e.g. means) or other basic estimates (e.g. regression coefficient) AND variation (e.g. standard deviation) or associated estimates of uncertainty (e.g. confidence intervals)
☒	☐	For null hypothesis testing, the test statistic (e.g. *F*, *t*, *r*) with confidence intervals, effect sizes, degrees of freedom and *P* value noted<br>*Give P values as exact values whenever suitable.*
☒	☐	For Bayesian analysis, information on the choice of priors and Markov chain Monte Carlo settings
☒	☐	For hierarchical and complex designs, identification of the appropriate level for tests and full reporting of outcomes
☒	☐	Estimates of effect sizes (e.g. Cohen's *d*, Pearson's *r*), indicating how they were calculated

Our web collection on statistics for biologists contains articles on many of the points above.

Software and code

Policy information about availability of computer code

Data collection	No software was used for the data collection

Data analysis	Trinity v2.8.4<br>Transdecoder<br>BUSCO v4.0.5<br>KEGG<br>EggNOG v5.0<br>BLASTP v2.9.0<br>CD-HIT v4.6.6<br>Broccoli v1.1<br>InterProScan v5.46.81<br>Phobius v1.01<br>CLANS<br>MAFFT v7.49<br>PhyKIT v1.11.7<br>TrimAl v1.4.1<br>IQ-TREE2 v2.1.4_beta/v2.1.3<br>Generax v2.0.1<br>recphyloXML v1.0.7<br>RogueNaRok v1.0

For manuscripts utilizing custom algorithms or software that are central to the research but not yet described in published literature, software must be made available to editors and reviewers. We strongly encourage code deposition in a community repository (e.g. GitHub). See the Nature Portfolio guidelines for submitting code & software for further information.

Data

Policy information about availability of data

All manuscripts must include a data availability statement. This statement should provide the following information, where applicable:

- Accession codes, unique identifiers, or web links for publicly available datasets
- A description of any restrictions on data availability
- For clinical datasets or third party data, please ensure that the statement adheres to our policy

Data underlying this study is provided in the figshare repositories with accessions: .

Research involving human participants, their data, or biological material

Policy information about studies with human participants or human data. See also policy information about sex, gender (identity/presentation), and sexual orientation and race, ethnicity and racism.

Reporting on sex and gender	N/A
Reporting on race, ethnicity, or other socially relevant groupings	N/A
Population characteristics	N/A
Recruitment	N/A
Ethics oversight	N/A

Note that full information on the approval of the study protocol must also be provided in the manuscript.

Field-specific reporting

Please select the one below that is the best fit for your research. If you are not sure, read the appropriate sections before making your selection.

☐ Life sciences ☐ Behavioural & social sciences ☒ Ecological, evolutionary & environmental sciences

For a reference copy of the document with all sections, see nature.com/documents/nr-reporting-summary-flat.pdf

Ecological, evolutionary & environmental sciences study design

All studies must disclose on these points even when the disclosure is negative.

Study description	We present a phylogenomic investigation of the genes involved in the monoaminergic system in animals
Research sample	Our study aimed to investigate the evolutionary history of the monoaminergic system in animals. To achieve this, we used a large taxonomical sampling including 47 animals' species (including 21 non-bilaterian species), 18 opisthokonts, and 34 other eukaryotes.

	The genomic data were obtained from NCBI and ENSMBL and was carefully curated to ensure that it was phylogenetically representative and comprehensive
Sampling strategy	We manually selected species with publicly available genomes prioritizing those from different phylogenetic groups (particularly focusing on non-bilaterian animals). We only chose genomes with a with high level of completeness (above 75%) according to BUSCO, exceptions were made for species of interest with no alternatives or closely related species. To further increase sampling of non-bilaterian animals we included de novo assembled transcription data for several cnidarian species. We limited sampling to 101 species as more would prove too large for timely computational analyses.
Data collection	R.F. and M.G. manually downloaded NCBI and ENSMBL accessions to retrieve the sequence data (see Supplementary Data 2)
Timing and spatial scale	Transcriptomic and genomic samples were retrieved from public repositories during 2019, 2020 and 2021
Data exclusions	No data were excluded from the analysis.
Reproducibility	Not applicable as this was a phylogenomic study
Randomization	Not applicable as this was a phylogenomic study
Blinding	Not applicable as this was a phylogenomic study

Did the study involve field work? ☐ Yes ☒ No

Reporting for specific materials, systems and methods

We require information from authors about some types of materials, experimental systems and methods used in many studies. Here, indicate whether each material, system or method listed is relevant to your study. If you are not sure if a list item applies to your research, read the appropriate section before selecting a response.

Materials & experimental systems

n/a	Involved in the study
☒	☐ Antibodies
☒	☐ Eukaryotic cell lines
☒	☐ Palaeontology and archaeology
☒	☐ Animals and other organisms
☒	☐ Clinical data
☒	☐ Dual use research of concern
☒	☐ Plants

Methods

n/a	Involved in the study
☒	☐ ChIP-seq
☒	☐ Flow cytometry
☒	☐ MRI-based neuroimaging
