# Supplementary material for: The monoaminergic system is a bilaterian innovation
Source: Nat Commun. 2023 Jun 6;14:3284. doi: 10.1038/s41467-023-39030-2 (PMC10244343; doi:10.1038/s41467-023-39030-2)
Supplement: Supplementary file 2 — Description of Additional Supplementary Files [file 41467_2023_39030_MOESM2_ESM.pdf]

Description of Additional Supplementary files

Supplementary Data 1: Literature Evidence for Monoamines in Non-bilaterians.

A table detailing the literature sources of chemical evidence for monoamines used in figure 1C. Colour coded by clade: dark blue = Porifera; light blue = Ctenophora; yellow = Cnidaria; dark orange = other eukaryotes. Shapes match figure 1C for evidence type: Circle = HPLC (high pressure liquid chromatography)/MS (mass spectroscopy); triangle = stainings; diamond = pharmacology. GC = gas chromatography; LC = liquid chromatography; UV = ultraviolet. Findings colour coded: present = black; ambiguous = grey; absence = red.

Supplementary Data 2: Species Data and BUSCO Scores.

A table detailing the species sample used in this study and the sources of sequence data. BUSCO (benchmarking universal single copy orthologues) metrics for each species data shown. Sp = species.

Supplementary Data 3: Broccoli Orthogroups of Interest Presence/absence.

A table detailing the genes present from each species in the broccoli orthogroups of interest. OG = orthogroups; GPCR = g-protein coupled receptor; 5HT3R = 5-hydroxytryptamine receptor 3; HRH/ACM = histamine receptor/acetylcholine muscarinic receptor; SLC = solute ligand carrier; DBH/TBH/MOXD = dopamine beta hydroxylase/tyramine beta hydroxylase/monooxygenase DBH-like; MAO = monoamine oxygenase; VMAT/VACHT = vesicular monoamine transporter/vesicular acetylcholine transporter; DDC/HDC/TDC = dopa decarboxylase/histidine decarboxylase/tyrosine decarboxylase; TPH/TH/PAH = tryptophan hydroxylase/tyrosine hydroxylase/phenylalanine hydroxylase; COMT = catechol-O-methyltransferase; GCH = GTP cyclo-hydrolase; HNMT = histamine-N-methyltransferase; PNMT = phenylethanolamine-N-methyltransferase.

Supplementary Data 4: Methodological Information.

A table detailing methodological information for phylogenetic analyses including the source of query sequences, the numbers of sequences in orthogroups/alignments, InterProScan motif codes counted as matches to annotations and substitution models used in phylogenetic analyses. OG = orthogroup; AAAH = aromatic amino acid hydroxylase; AADC = aromatic amine decarboxylase; BH = beta hydroxylase; PNMT = phenylethanolamine-N-methyltransferase; VMAT = vesicular monoamine transporter; SLC = solute ligand carrier; GCH = GTP cyclo-hydrolase; MAO = monoamine oxidase; COMT = catechol-O-methyltransferase; HNMT = histamine-N-methyltransferase; GPCR = g-protein coupled receptor; HRH = histamine receptor; ACM = acetylcholine muscarinic receptor; 5HT3R = 5-hydroxytryptamine receptor 3; PAH = phenylalanine hydroxylase; TPH = tryptophan hydroxylase; TH = tyrosine hydroxylase; DDC = dopa decarboxylase; TDC = tyrosine decarboxylase; HDC = histidine decarboxylase; DBH = dopamine beta hydroxylase; TBH = tyramine beta hydroxylase; MOXD = monooxygenase DBH-like; NNMT = nicotinamide-N-methyltransferase; INMT = indolethylamine-N-methyltransferase; VACHT = vesicular acetylcholine transporter; SAM-MT = S-adenosyl methionine methyltransferase; SERT = serotonin transporter; IDAT = invertebrate dopamine transporter; DAT = dopamine

transporter; NET = nor-epinephrine transporter; 5HTR = 5-hydroxytryptamine receptor; DRD = dopamine receptor; ARDB = beta adrenergic receptor; ADRA = alpha adrenergic receptor; Tyr = tyraminergic receptor; OctR = octopaminergic receptor; TAAR = trace amine-associated receptor.

Supplementary Data 5: Alignment Statistics.

A table detailing various statistics for the alignments used in phylogenetic analyses. Seq No = number of sequences; min = minimum pairwise similarity; max = maximum pairwise similarity; mean = mean pairwise similarity; SD = standard deviation; RCV = relative composition variability; OG = orthogroup; AAAH = aromatic amino acid hydroxylase; AADC = aromatic amine decarboxylase; BH = beta hydroxylase; PNMT = phenylethanolamine-N-methyltransferase; VMAT = vesicular monoamine transporter; SLC = solute ligand carrier; GCH = GTP cyclo-hydrolase; MAO = monoamine oxidase; COMT = catechol-O-methyltransferase; HNMT = histamine-N-methyltransferase; GPCR = g-protein coupled receptor; 5HT3R = 5-hydroxytryptamine receptor 3.

Supplementary Data 6: Rogue Taxa Statistics.

A table detailing rouge taxa scores for all sequences. OG = orthogroup; AAAH = aromatic amino acid hydroxylase; AADC = aromatic amine decarboxylase; BH = beta hydroxylase; PNMT = phenylethanolamine-N-methyltransferase; VMAT = vesicular monoamine transporter; SLC = solute ligand carrier; GCH = GTP cyclo-hydrolase; MAO = monoamine oxidase; COMT = catechol-O-methyltransferase; HNMT = histamine-N-methyltransferase; 5HT3R = 5-hydroxytryptamine receptor 3; GPCR = g-protein coupled receptor; TBE = transfer bootstrap expectation.
